# Identification and expression profiles of olfactory-related genes in the antennal transcriptome of *Graphosoma rubrolineatum* (Hemiptera: Pentatomidae)

**DOI:** 10.1371/journal.pone.0306986

**Published:** 2024-08-06

**Authors:** Shibao Guo, Panjing Liu, Yin Tang, Junhua Chen, Tao Zhang, Hongmin Liu

**Affiliations:** 1 College of Agronomy, Xinyang Agriculture and Forestry University, Xinyang, China; 2 Plant Protection Institute, HAAFS/Key Laboratory of IPM on Crops in Northern Region of North China, Ministry of Agriculture and Rural Affairs, Baoding, P. R. China; 3 IPM Innovation Center of Hebei Province/International Science and Technology Joint Research Center on IPM of Hebei Province, Baoding, China; 4 CAS Center for Excellence in Molecular Plant Sciences, Shanghai Institute of Plant Physiology and Ecology, Chinese Academy of Sciences, Shanghai, China; Universita degli Studi della Basilicata, ITALY

## Abstract

*Graphosoma rubrolineatum* (Hemiptera: Pentatomidae) is an important pest of vegetables and herbs (e.g., Umbelliferae and Cruciferae) in China, Siberia, Korea, and Japan. Insects are highly dependent on their olfactory system to detect odorants. However, no molecular-mediated olfactory genes in *G*. *rubrolineatum* have yet been identified. In this study, we first established the antennal transcriptome of *G*. *rubrolineatum* and identified 189 candidate olfactory genes, including 31 odorant-binding proteins (OBPs), 15 chemosensory proteins (CSPs), four sensory neuron membrane proteins (SNMPs),94 odorant receptors (ORs), 23 ionotropic receptors (IRs), and 22 gustatory receptors (GRs). Additionally, phylogenetic trees were constructed for olfactory genes between *G*. *rubrolineatum* and other hemipteran insects. We also detected the expression profiles of ten OBPs, five CSPs, two SNMPs, five ORs, four IRs, and four GRs by real-time quantitative PCR. The results revealed that most genes (*GrubOBP1/11/31*, *GrubCSP3/8*, *GrubSNMP1a/1b*, *GrubOrco/OR9/11/13*, *GrubGR1/4/22*, *GrubIR25/75h/76b/GluR1*) were highly expressed in the antennae, *GrubOBP13/31* and G*rubCSP4/11/12* were highly expressed in the legs, while *GrubOBP20* and *GrubGR19* were highly expressed in the wings. Our results will enrich the gene inventory of *G*. *rubrolineatum* and provide further insight into the molecular chemosensory mechanisms of *G*. *rubrolineatum*.

## Introduction

Insect chemosensory systems are critical for detecting volatiles to locate mates, oviposition sites, and food, as well as to avoid predators or hazardous substances [1, 2]. The recognition of chemical cues by insects is a remarkably complicated process that relies on the involvement of olfactory receptor neurons (ORNs) in chemosensory organs, where chemical stimuli are transformed into electrical signals and conveyed to the brain [3–5]. The antenna is a vital organ that plays an indispensable role in chemoreception. Increasing evidence suggests that the chemosensory system involves diverse olfactory genes, including odorant-binding proteins (OBPs), chemosensory proteins (CSPs), odorant receptors (ORs), ionotropic receptors (IRs), gustatory receptors (GRs), and sensory neuron membrane proteins (SNMPs) [4, 6, 7].

OBPs, which are abundant mainly in the antennae, are small, soluble proteins that bind and solubilize hydrophobic odorant molecules from the external environment and transfer them to chemosensory receptors embedded in ORNs [8, 9]. Most chemosensory OBPs contain 130–150 amino acids and have a common folding style of six α-helical domains [4, 10]. The classic OBP has a conserved pattern of six cysteines that form three disulfide bridges; minus-C OBPs lack one or two cysteines (C2 and C5), while plus-C OBPs have two extra cysteines and a distinctive proline following the sixth cysteine [11–13]. CSPs consist of 100–120 amino acids and four conserved cysteines that form two disulfide bridges [14]. Unlike OBPs, CSPs have a wider tissue distribution, and certain members are found in nonsensory organs where they play a variety of roles, including development and differentiation [15–17]. SNMPs (comprising 519–525 amino acids) are members of the CD36 family with two transmembrane domains (TMDs); and they were initially identified in olfactory receptor neurons of Lepidoptera and are involved in pheromone perception in *Drosophila* [18–20].

In addition to olfactory proteins, the insect olfactory system involves three types of chemosensory receptors: ORs, IRs, and GRs [4, 21]. Insect ORs are seven-transmembrane proteins with a structure opposite to that of typical G-protein-coupled receptors (GPCRs; the N-terminus is intracellular, and the C-terminus is extracellular) [22]. Generally, ORs can be divided into two subtypes: the conventional ORx (highly diverse in insects) and the olfactory coreceptor (Orco; significantly conserved in insect species) [23]. ORx and Orco form a special heterodimer and participate in the signal transduction of odorant molecules [24]. In addition, ORx plays a crucial role in specificity, while Orco assists ORx in achieving location accuracy, which can enhance the sensitivity to odorant molecules [22, 25]. IRs are a novel type of olfactory receptor that belongs to a subfamily of ancient and highly conserved ionotropic glutamate receptors (iGluRs) [6]. Insect IRs can be categorized into two subgroups: conserved “antennal IRs,” which are involved in olfaction in ORNs in antennae, and species-specific “divergent” IRs, which are involved in taste in gustatory organs [26–28]. GRs are essential for recognizing various materials, such as salts, CO_2_, sugars, and organic compounds, and are involved in the feeding activities of insects [29–32]. Moreover, GRs are mainly expressed in gustatory receptor neurons in taste organs and are also present in other tissues such as antennae, wings, heads, ovaries, and testes [33, 34]. This suggests that the functions of GRs are both conserved and divergent.

*Graphosoma rubrolineatum* (Hemiptera: Pentatomidae) is a significant pest of vegetables and herbs (e.g., Umbelliferae and Cruciferae) in China, Siberia, Korea, and Japan [35, 36]. Adults and nymphs of this species mainly inhabit the leaves, flower buds, and young pods of host plants to absorb sap. Even worse, this can deform seed pods and reduce seed yield [35, 37]. However, the molecular-mediated chemosensory systems in *G*. *rubrolineatum* have not been yet defined. In this study, we focused on the antennal transcriptome of *G*. *rubrolineatum* and identified candidate olfactory genes. Furthermore, phylogenetic analysis and expression profiling of these identified olfactory genes were also conducted to further elucidate their potential functions.

## Materials and methods

### Insect rearing and sample collection

*G*. *rubrolineatum* nymphs were obtained from an experimental field with radish plants in July 2022 in Baoding, China (38.96°N, 115.46°E) and reared in an insectary at 28°C ± 1°C, L: D = 12:12, and 80% relative humidity. Nymphs were fed fresh radish leaves until adult eclosion. The different adult tissues (female antennae, male antennae, heads, legs, thoraxes, abdomens, and wings) of adults were collected and rapidly frozen in liquid nitrogen. Then the samples were stored at -80°C until use. Three biological replicates were established for each tissue sample, with each antennal sample containing 50 pairs of antennae. In addition, other tissues were collected from 10 to 20 adults.

### RNA extraction, sequencing, assembly, and annotation

TRIzol reagent (TransGen, China) was used for total RNA extraction. To ensure the use of qualified samples for transcriptome sequencing, the purity, concentration, and integrity of the RNA samples were evaluated using advanced molecular biology equipment. For cDNA library construction, one microgram of RNA per sample was used with the NEBNext® Ultra™ RNA Library Prep Kit for Illumina® (NEB, USA). The library preparations were then sequenced on an Illumina HiSeq 2000 platform and paired-end reads were generated (BioMaker, Beijing, China). Finally, the raw sequence transcriptome data from the antennae libraries of *G*. *rubrolineatum* have been were uploaded to the Sequence Read Archive (SRA) database (BioSample number: SAMN39436715-SAMN39436720).

Transcriptome assembly was completed using Trinity (v2.5.1, with the parameter min_kmer_cov 2) to generate transcripts, based on high-quality clean data [38]. The longest transcript was selected as the unigene. All unigenes obtained from *G*. *rubrolineatum* were annotated against the NCBI non-redundant protein database (NR), Swiss-Prot, Clusters of Orthologous Groups (COG), EuKaryotic Orthologous Groups (KOG), eggNOG4.5, and Kyoto Encyclopedia of Genes and Genomes (KEGG) using DI-AMOND software [39] and the Gene Ontology (GO) results of the genes were analyzed using InterProScan [40]. Sunsequently, HMMER software [41] was used for comparison with the Pfam database [42] to obtain annotation information for the unigenes.

### Differential expression analysis

The abundance of unigenes was calculated using the fragments per kilobase per million mapped reads (FPKM) method [43]. The DESeq R package (version 1.10.1) was used to perform differential expression analysis of the two groups. DESeq provides statistical routines to determine differential expression in digital gene expression data using a model based on the negative binomial distribution. To control the false discovery rate, the resulting p- values were adjusted using Hochberg’s and Benjamini approach. Genes with an adjusted p-value of <0.05, as identified by DESeq, were considered differentially expressed genes [44].

### Identification of olfactory-related genes

To identify candidate OBPs, CSPs, SNMPs, ORs, IRs, and GRs, known protein sequences from other hemipteran species (*Adelphocoris lineolatus*, *Apolygus lucorum*, *Ad*. *suturalis*, *Arma chinensis*, *Cyrtorhinus lividipennis*, *Halyomorpha halys*, *Nezara viridula*, *Tessaratoma papillosa*) were used as queries to search the antennal transcriptomes of *G*. *rubrolineatum*. The HMM software package (hmmsearch) was used to identify the putative olfactory proteins with an e-value < 1e−5. To further verify all candidate genes, we conducted a BLAST search against the NR database to remove genes with low identity (< 30%). Moreover, the presence of definitive domains in these putative proteins was also predicted using the InterProScan tool plug-in in Geneious (version: 9.1.3) [45].

### Sequence analysis and phylogenetic analysis

Putative N-terminal signal peptides of GrubOBPs and GrubCSPs were predicted using SignalP 6.0 (https://services.healthtech.dtu.dk/services/SignalP-6.0/). The TMHMM Server Version 2.0 (https://services.healthtech.dtu.dk/services/TMHMM-2.0/) was used to predict the TMDs of GrubORs, GrubIRs, and GrubGRs. Sequence alignments of CSPs, OBPs, and SNMPs were performed using ClustalX-2.1, and the results were visualized using GeneDoc software (http://nrbsc.org/gfx/genedoc). Evolutionary analysis was conducted in MEGA7 using the neighbor-joining method with a bootstrap test (1000 replicates) [46]. Finally, phylogenetic trees were visualized and edited using the iTOL online tools (https://itol.embl.de/) [47].

### Expression analysis

Based on log-transformed FPKM values, the expression levels of the olfactory genes in *G*. *rubrolineatum* antennae were visualized using a heatmap in TBtools (version 1.098728) [48]. Quantitative real-time PCR (qRT‒PCR) was then used to explore the expression profiles of the olfactory genes. One microgram of total RNA was reverse transcribed into cDNA using All-in-One First-Strand cDNA Synthesis SuperMix (TransGen, China). Gene-specific primers (S1 Table) were designed using Premier 6.0, and the *GrubEFIa* and *GrubRPS18* were selected as reference genes. qRT‒PCR was performed on an Applied Biosystems QuantStudio 6 Real-Time PCR System (Applied Biosystems, Foster City, CA, United States of America). The primer amplification efficiency was 92–95% as determined by the pre-experiment. Then, expression data analyses were performed using the 2^^-ΔΔCT^ method [49].

### Statistical analysis

SPSS (version 25.0) was used for the statistical analysis. The qRT‒PCR data from six samples were analyzed using one-way analysis of variance (ANOVA) with Tukey’s multiple comparison test in SPSS (version 25.0). The data are presented as the mean + standard error. Statistical significance between samples was determined with a significance level of p < 0.05. Finally, the results were visualized using GraphPad Prism 8 software.

## Results

### Overview of the *Graphosoma rubrolineatum* antennal transcriptome

**Fig 1:**
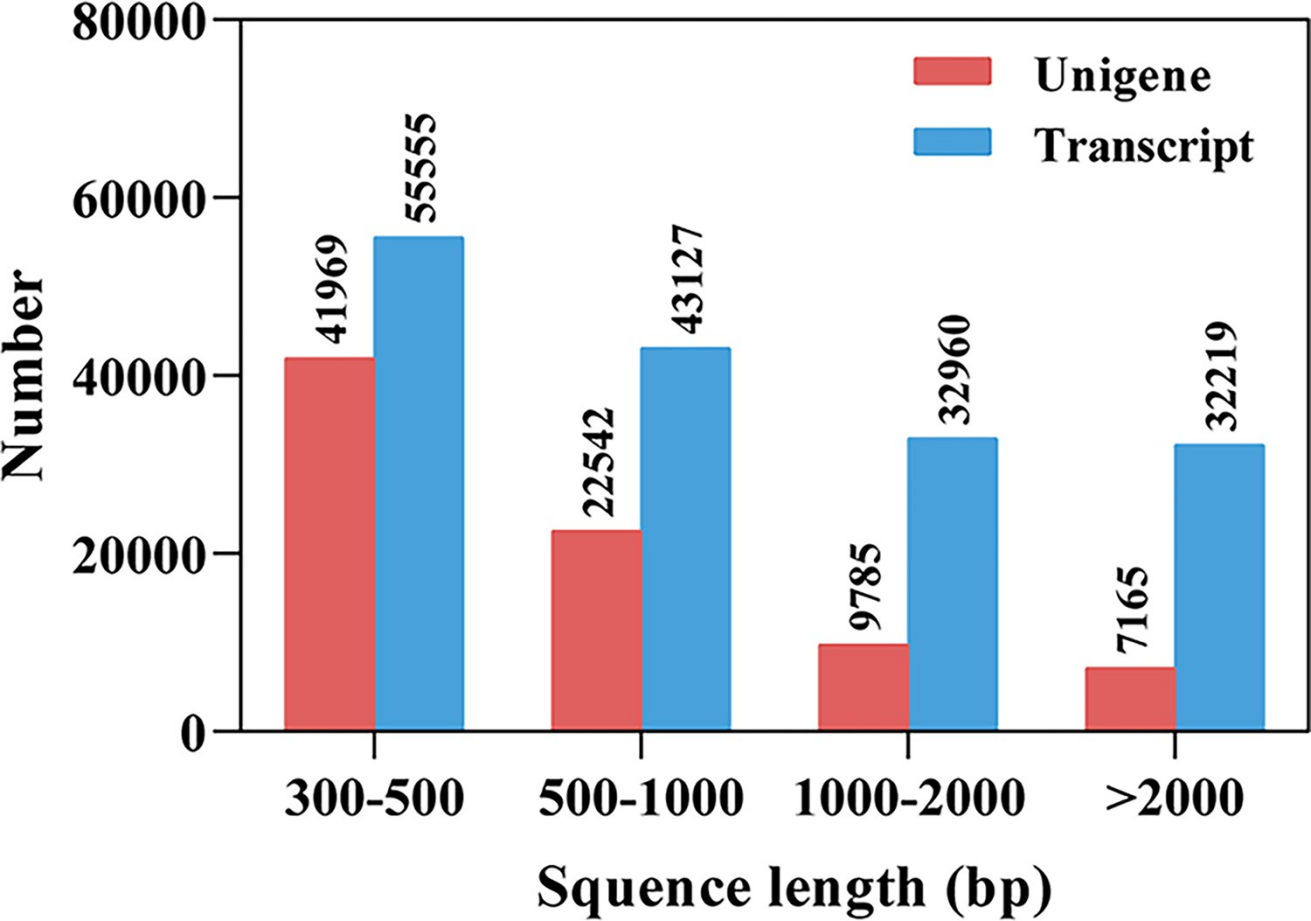
Distribution of transcripts and unigenes size in the *Graphosoma rubrolineatum* transcriptome.

We sequenced the transcriptomes of female antennae (FA) and male antennae (MA) in three independent biological replicates. A total of 23,277,222 (FA-1), 22,550,200 (FA-2), 21,112,206 (FA-3), 21,261,590 (MA-1), 21,326,206 (MA-2) and 20,900,550 (MA-3) clean reads were obtained. The quality of the transcriptome sequences was high, with Q30 percentages of 93.74%, 93.30%, 93.43%, 94.05%, 93.39%, and 92.93% for these replicates (S2 Table). A total of 163,865 transcripts and 81,462 unigenes were generated with N50 lengths of 2,165 bp and 1,193 bp, respectively. Among those unigenes, 48.48% (39,492) were longer than 500 base pairs (bp), and 20.81% (16,950) were longer than 1,000 bp (Fig 1 and S3 Table).

**Fig 2:**
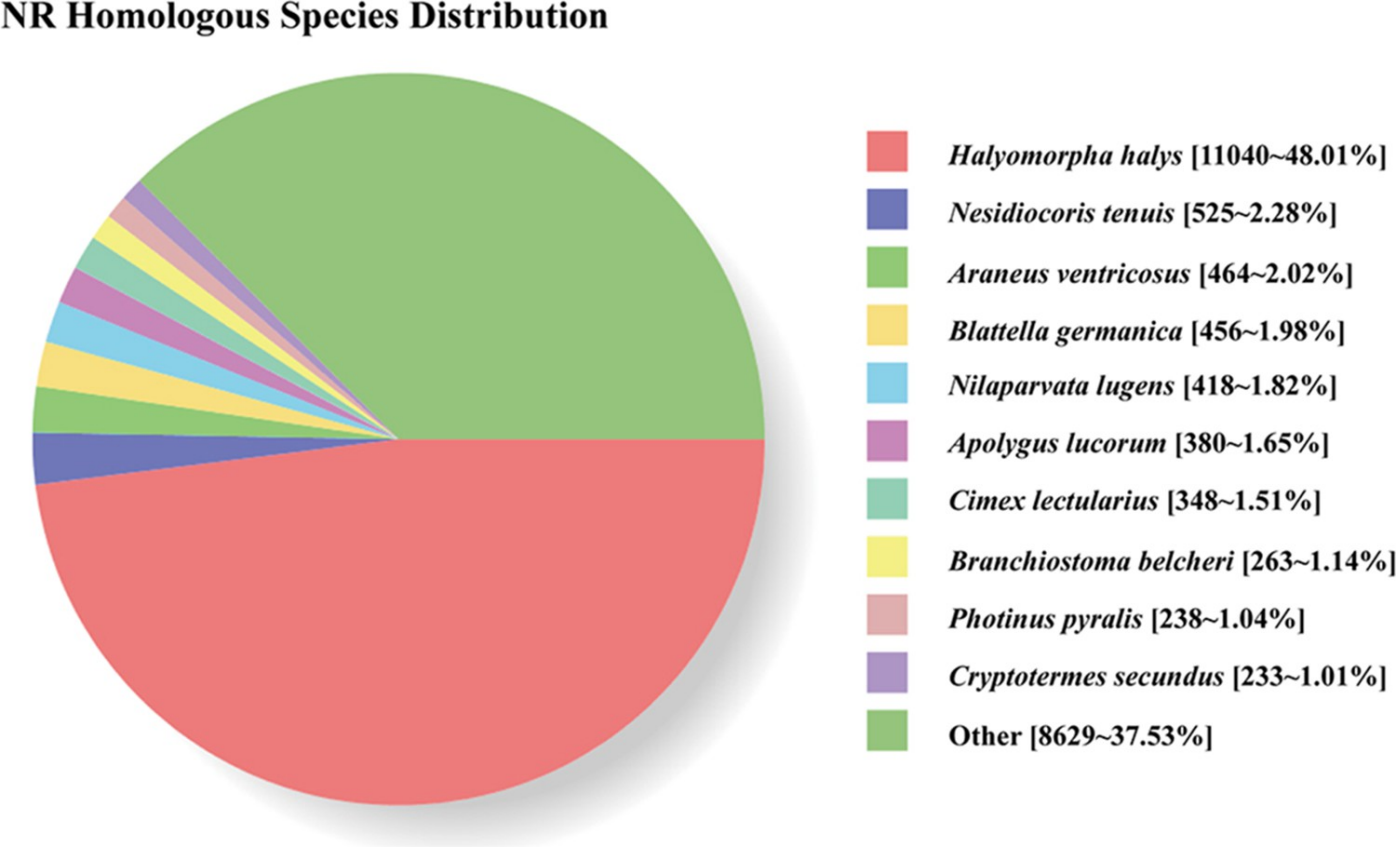
Percentage of homologous hits of the *Graphosoma rubrolineatum* unigenes to other species. Blastx searched the *G*. *rubrolineatum* unigenes against the non-redundancy protein database with a cut-off E-value of 10–5.

Nine databases were used to obtain comprehensive information on gene function. Of the total unigenes, 22,994 (28.23%) had significant matches in the NR database, 22,491 (27.61%) in the TrEMBL database, 17,731 (21.77%) in the GO database, 16,345 (20.06%) in the eggNOG database and 14,680 (18.02%) in the Pfam database (Table 1). Among the NR-hit unigenes, the highest match percentage (48.01%) was found for sequences related to *H*. *halys*, followed by sequences from *Nesidiocoris tenuis* (2.28%) (Fig 2). The GO-hit unigenes could be assigned to three GO terms: biological processes, molecular functions, and cellular components. In the biological process category, the unigenes were mainly enriched in the cellular process (11,426), metabolic process (9,665), and biological regulation (3,195) terms. In the molecular function category, the annotations were mainly enriched in the binding (9,140) and catalytic activity (8,049) terms. Among the cellular components, the largest group was the cellular anatomical entity process (8,133), followed by the intracellular process (11,785) (S1 Fig). A total of 12,151 unigenes were classified into 25 clusters of KOG categories. The largest category was the group focused on general function prediction (22.01%), followed by the group centered on signal transduction mechanisms (11.19%) (S2 Fig).

**Table 1:** Summary for the annotation of the *Graphosoma rubrolineatum* transcriptome.

Databases	Number of Unigenes	Percentage (%)
Annotated in NR	22,994	28.23
Annotated in TrEMBL	22,491	27.61
Annotated in GO	17,731	21.77
Annotated in eggNOG	16,345	20.06
Annotated in Pfam	14,680	18.02
Annotated in KEGG	13,815	16.96
Annotated in KOG	12,151	14.92
Annotated in SwissProt	8,937	10.97
Annotated in COG	4,723	5.80
Annotated in all Databases	25,586	31.41

#### Candidate odorant binding proteins (OBPs)

We identified 31 OBPs (GrubOBP1-31) from *G*. *rubrolineatum* antennal transcriptome dataset. Twenty-three GrubOBPs had full-length open reading frames (ORFs) ranging from 129 to 241 amino acids. Among the complete OBPs, 20 OBPs contained signal peptide sequences in addition to the three proteins (GrbOBP5, GrbOBP6, and GrbOBP17) (S4 Table). Furthermore, 22 GrubOBPs (GrubOBP5, GrubOBP7-27) were classic OBPs with six cysteines (C1-X_22-37_-C2-X_3_-C3-X_41-43_-C4-X_8-21_-C5-X_8_-C6), while five GrubOBPs (GrubOBP1-4, GrubOBP6) were plus-C OBPs with additional cysteine residues and a conserved proline after the sixth cysteine (S3 Fig).

**Fig 3:**
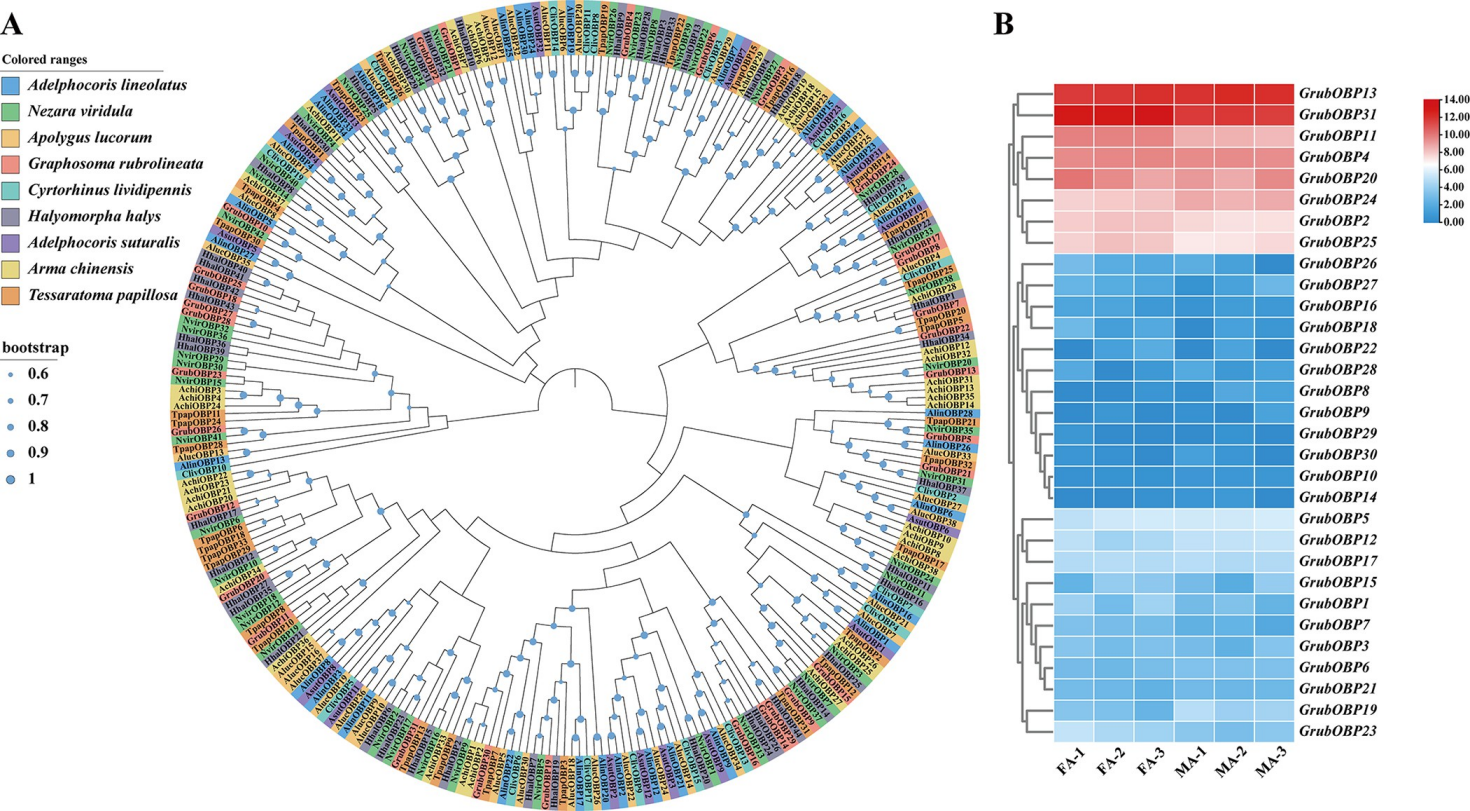
Analyses of candidate odorant-binding proteins (OBPs). (A) Neighborhood-joining method tree of OBPs in *Graphosoma rubrolineatum* and other hemipteran insects. The amino acid sequences used in this analysis are listed in S5 Table. (B) FPKM values of *GrubOBPs* based on antennal transcriptome data in *G*. *rubrolineatum*. FA: female antennae, MA: male antennae.

The phylogenetic tree constructed based on eight other hemipteran species (*Ad*. *lineolatus*, *Ap*. *lucorum*, *Ad*. *suturalis*, *Ar*. *chinensis*, *C*. *lividipennis*, *H*. *halys*, *N*. *viridula*,*Tp*. *papillosa*) showed that GrubOBPs were supported by high bootstrap values with *N*. *viridula*, *H*. *halys*, and*Te*. *papillosa*. As depicted in Fig 3A, five GrubOBPs (GrubOBP1-4, GrubOBP6) were clustered on the same branch along with NvirOBP21, HhalOBP31, NvirOBP27, HhalOBP9, and NvirOBP22. All 31 GrubOBPs could be clustered into three groups (high, mid, and low expression) based on their FPKM values in the antennae. Among these genes, *GrubOBP13* and *GrubOBP31* were highly expressed in both female and male antennae. *GrubOBP5*, *GrubOBP12*, and *GrubOBP17* were moderately expressed, while *GrubOBP9*, *GrubOBP22*, and *GrubOBP29* had relatively low expression (S4 Table). In particular, *GrubOBP11* exhibited greater expression in female antennae than in male antennae, while *GrubOBP24* was preferentially expressed in male antennae (Fig 3B).

#### Candidate chemosensory proteins (CSPs)

A total of 15 candidate CSPs were identified from the dataset of the *G*. *rubrolineatum* antennal transcriptome. Among these, 11 GrubCSPs had intact ORFs that encoded proteins with 98 to 142 amino acid residues, along with a signal peptide. The sequence identity of the GrubCSPs ranged from 46.24% to 96.33% in *C*. *lividipennis*, *N*. *viridula*, *Riptortus pedestris*, and *Tropidothorax elegans* (S4 Table). The majority of the 15 CSPs contained four conserved cysteine residues with the common cysteine sequence motif of C1-X_6-8_-C2-X_18_-C3-X_2_-C4, whereas GrubCSP14 lacked the third cysteine (C3) (S4 Fig).

**Fig 4:**
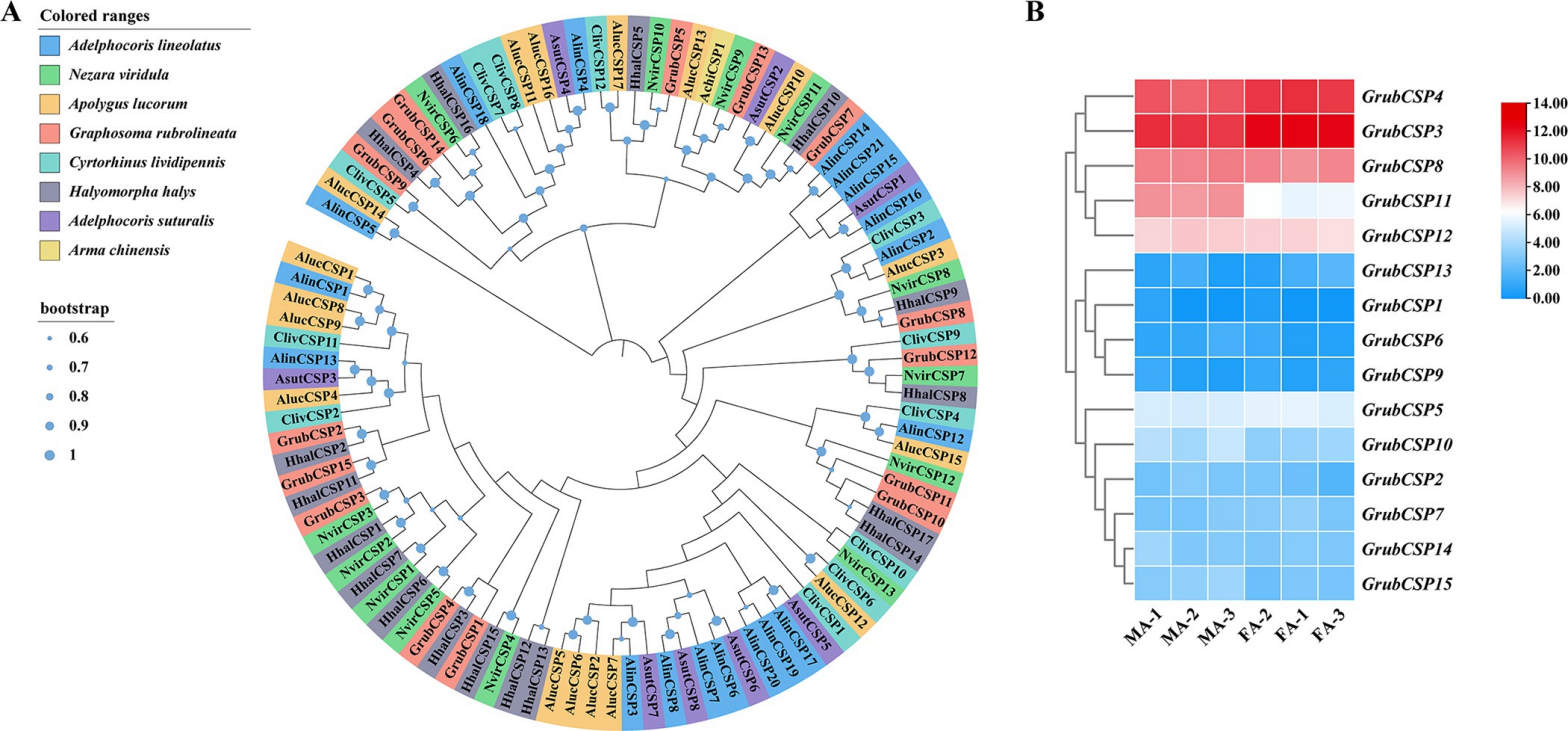
Analyses of candidate chemosensory proteins (CSPs). (A) Neighborhood-joining method tree of CSPs in *Graphosoma rubrolineatum* and other hemipteran insects. The amino acid sequences used in this analysis are listed in S5 Table. (B) FPKM values of *GrubCSPs* based on antennae transcriptome data in *G*. *rubrolineatum*. FA: female antennae, MA: male antennae.

All 15 GrubCSPs were used to construct the phylogenetic tree with seven hemipteran species (*Ar*. *chinensis*, *Ad*. *lineolatus*, *Ap*. *lucorum*, *Ad*. *suturalis*, *C*. *lividipennis*, *H*. *halys*, *N*. *viridula*). The results showed that all GrubCSPs were distributed on various branches and were homologous to those of *H*. *halys* and *N*. *viridula* (Fig 4A). According to the FPKM values, only five *GrubCSPs* (*GrubCSP3*, *GrubCSP4*, *GrubCSP8*, *GrubCSP11*, and *GrubCSP12*) exhibited relatively high expression in the antennae. Furthermore, there was a strong preference for *GrubCSP11* expression in male antennae, which was eightfold greater than that in female antennae (Fig 4B).

#### Candidate sensory neuron membrane proteins (SNMPs)

Four putative SNMP genes were identified from the antennal transcriptome, among which *GrubSNMP1a* and *GrubSNMP1b* had full-length ORFs. The sequence identities of the two GrubSNMPs were 58.45% and 89.65% with those of *Ad*. *lineolatus* and *H*. *haly*, respectively (S4 Table). According to the phylogenetic tree, these SNMPs were divided into two subfamilies: GrubSNMP1a/1b belonged to the SNMP1 family, while GrubSNMP2a and GrubSNMP2b were classified into the SNMP2 family. According to the FPKM values from the antennal transcriptome, *GrubSNMP1a*, and GrubSNMP1b were highly expressed in both female and male antennae, while the expression of *GrubSNMP2a* and *GrubSNMP2b* was negligible in the antennae (S5 Fig).

#### Candidate odorant receptors (ORs)

A total of 94 putative OR genes were identified by screening the antennal transcriptome of *G*. *rubrolineatum*. Among these ORs, 79 encode full-length proteins ranging from 270 to 475 amino acids in length that contain 3 to 8 TMDs according to the TMHMM prediction. The sequence identity of these ORs ranged from 30.10% to 97.89% compared to that of homologous ORs in other species. Furthermore, the sequence identity of GrubOrco with the conserved insect Orco in *Plautia stali* was 97.89% (S4 Table).

**Fig 5:**
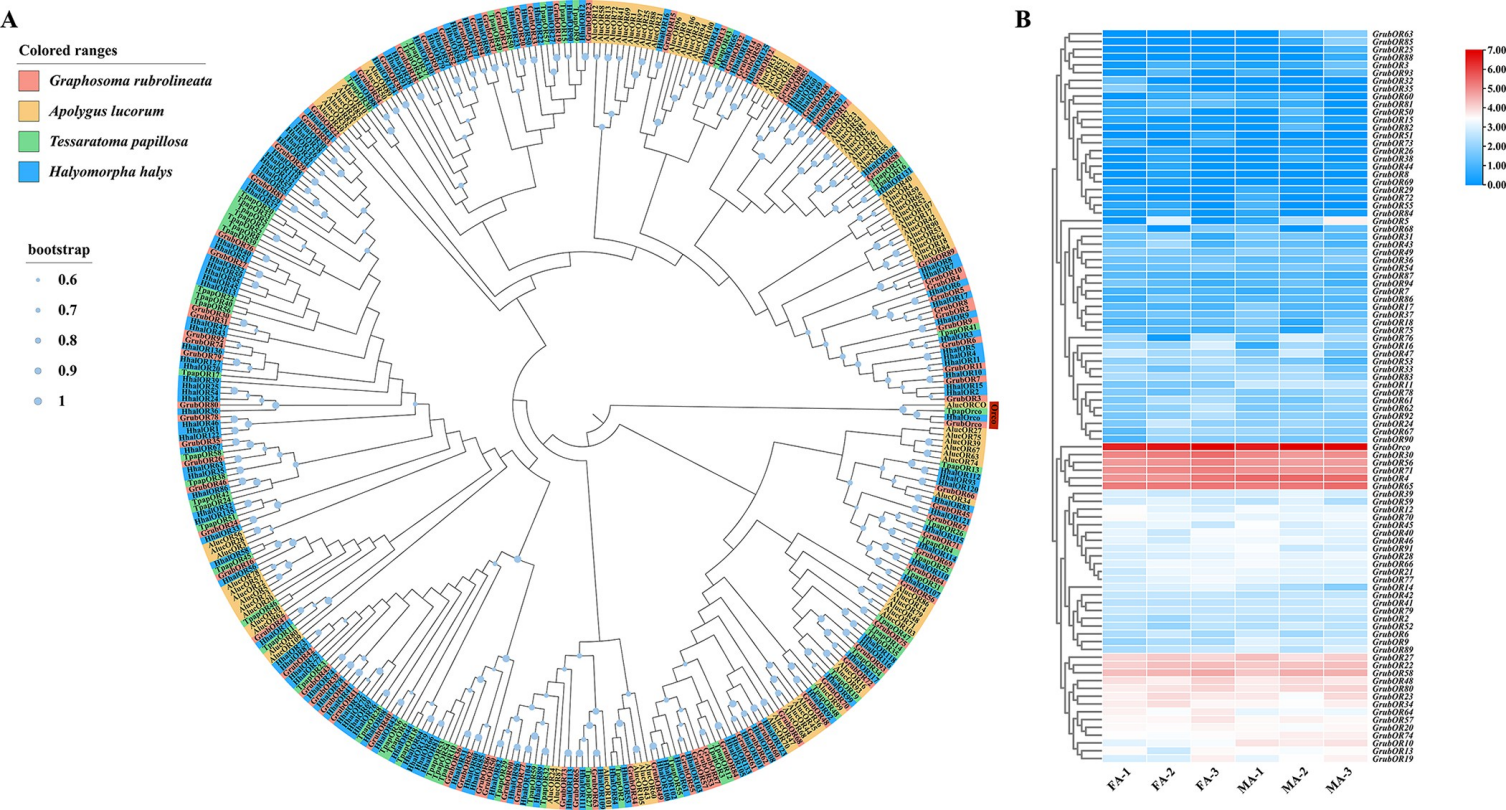
Analyses of candidate odorant receptors (ORs). (A) Neighborhood-joining method tree of ORs in *Graphosoma rubrolineatum* and other hemipteran insects. The amino acid sequences used in this analysis are listed in S5 Table. (B) FPKM values of *GrubORs* based on antennae transcriptome data in *G*. *rubrolineatum*. FA: female antennae, MA: male antennae.

Phylogenetic analysis was conducted using 94 ORs in *G*. *rubrolineatum* and ORs from other hemipteran species. The results indicated that the GrubORs were highly homologous to those of *H*. *haly*, and GrubOrco was clustered in the Orco family, which is highly conserved in insects (Fig 5A). The FPKM values from the antennal transcriptome of 94 *GrubORs* were visualized using a heatmap. The results revealed that *GrubOrco* exhibited the highest expression in both female and male antennae (FA: 101.69, MA: 110.34), followed by *GrubOR4*, *GrubOR30*, *GrubOR56*, *GrubOR65*, *and GrubOR71* (S4 Table, Fig 5B). In addition, *GrubOR64* showed bias toward female antennae expression, while *GrubOR10* and *GrubOR19* were more highly expressed in male antennae than in female (Fig 5B).

#### Candidate ionotropic receptors (IRs)

**Fig 6:**
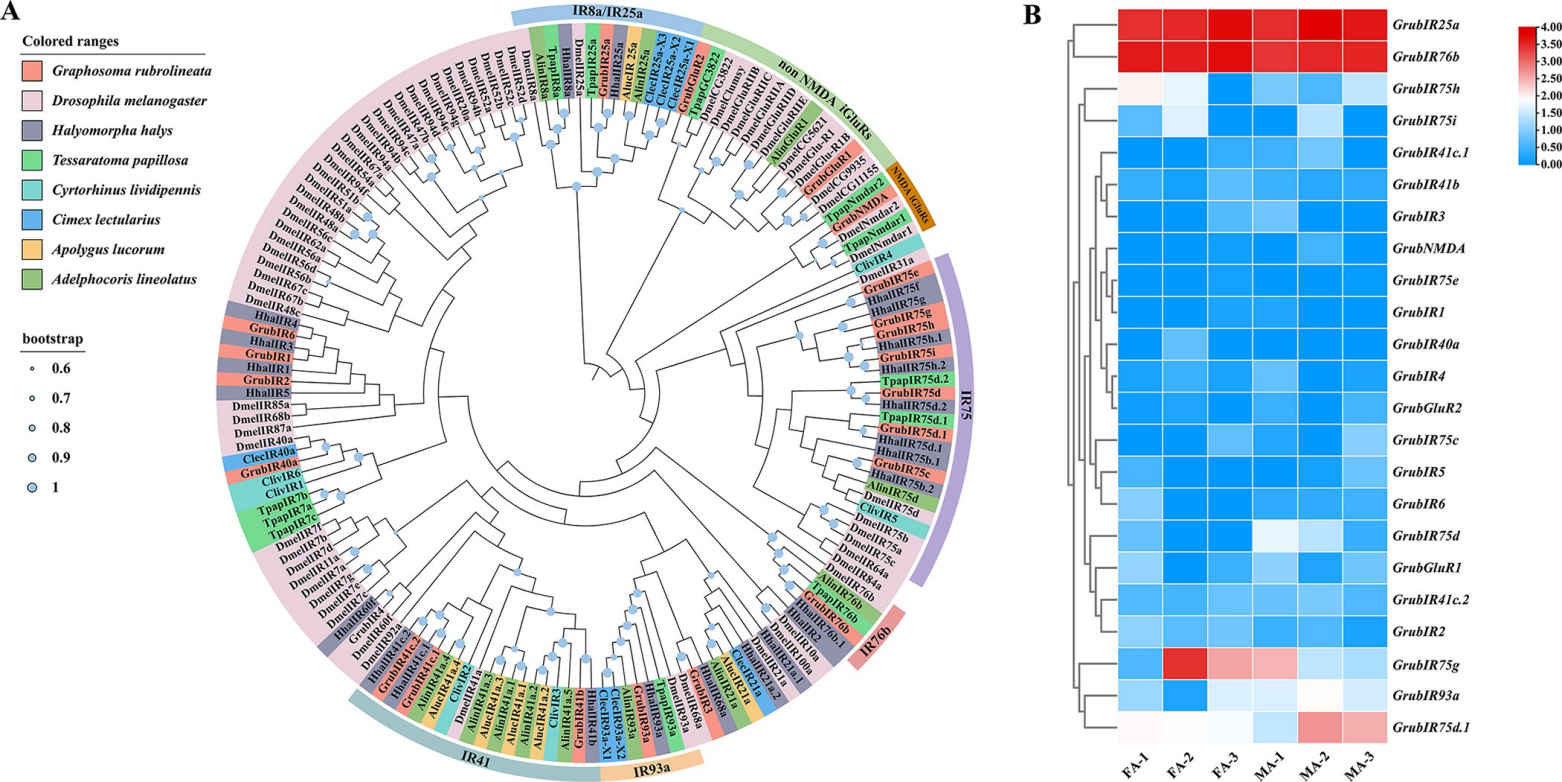
Analyses of putative of candidate ionotropic receptors (IRs). (A) Neighborhood-joining method tree of IRs in *Graphosoma rubrolineatum* and other hemipteran insects. The amino acid sequences used in this analysis are listed in S5 Table. (B) FPKM values of *GrubIRs* based on antennae transcriptome data in *G*. *rubrolineatum*. FA: female antennae, MA: male antennae.

Twenty-three IRs were identified from the antennae transcriptome data in *G*. *rubrolineatum*, and twelve IRs encode full-length proteins ranging from 465 to 932 amino acids. All GrubIRs showed high levels of identity (> 72%) with other reported insect IRs based on the BLASTX results, indicating that these IRs were highly conserved in hemipteran insects (S4 Table). As shown in Fig 6A, all the identified IRs can be classified into different subgroups, such as NMDA iGluRs, non-NMDA iGluRs, IR8a/IR25a, IR76b, and IR93a. GrubGluR1 and GrubGluR2 belong to the non-NMDA iGluR family, and GrubIR93a was clustered on the subbranches of IR93a with a high bootstrap value. In addition, GrubIR25a was clustered on the same branch as HhalIR25a, with the bootstrap value of 0.99, and GrubIR76b and HhalIR76b.1 were clustered together on the same branch, with the bootstrap value of 0.99. The heatmap results showed that *GrubIR25a* exhibited the highest expression value (FA: 10.92, MA: 11.53, mean FPKM), followed by *GrubIR76b* (FA: 11.52, MA: 10.27, mean FPKM) (S4 Table and Fig 6B). *GrubIR75g* was significantly highly expressed in female antennae, while *GrubIR75d*.*1* was notably expressed in male antennae (Fig 6B).

#### Candidate gustatory receptors (GRs)

**Fig 7:**
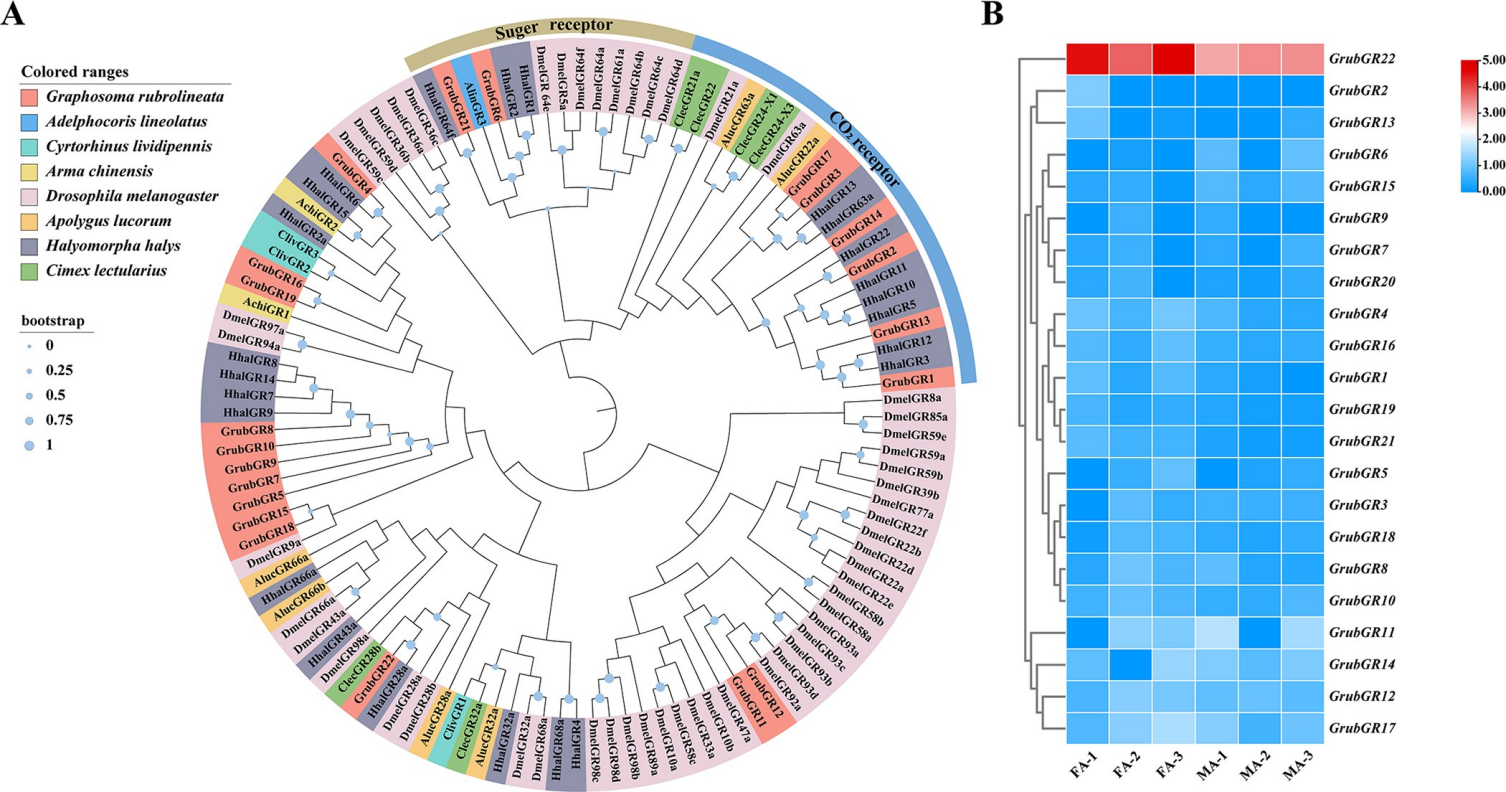
Analyses of putative of candidate gustatory receptors (GRs). (A) Neighborhood-joining method tree of GRs in *Graphosoma rubrolineatum* and other hemipteran insects. The amino acid sequences used in this analysis are listed in S5 Table. (B) FPKM values of *GrubGRs* based on antennae transcriptome data in *G*. *rubrolineatum*. FA: female antennae, MA: male antennae.

Twenty-two unigenes encoding candidate GRs were identified in *G*. *rubrolineatum* from the antennal transcriptome (S4 Table). Eleven GRs had full-length ORFs encoding 242–429 amino acids, each containing 4–8 predicted transmembrane domains. Phylogenetic analysis revealed that GrubGR6 and GrubGR21 belong to the sugar receptor subfamily, while six GrubGRs (GrubGR1, GrubGR2, GrubGR3, GrubGR13, GrubGR14, and GrubGR17) were classified as members of the CO_2_ receptor subfamily. GrubGR4 and HhalGR6 were clustered on the same branch, while GrubGR22 clustered with HhalGR28a (Fig 7A). The FPKM values indicated that most *GrubGRs* were significantly less highly expressed in antennae than were *GrubORs*. Only *GrubGR22* was highly expressed in both male and female antennae (FA: 19.85, MA: 8.84, mean FPKM) (S4 Table and Fig 7B).

#### Tissue expression profiles of olfactory genes in *Graphosoma rubrolineatum*

**Fig 8:**
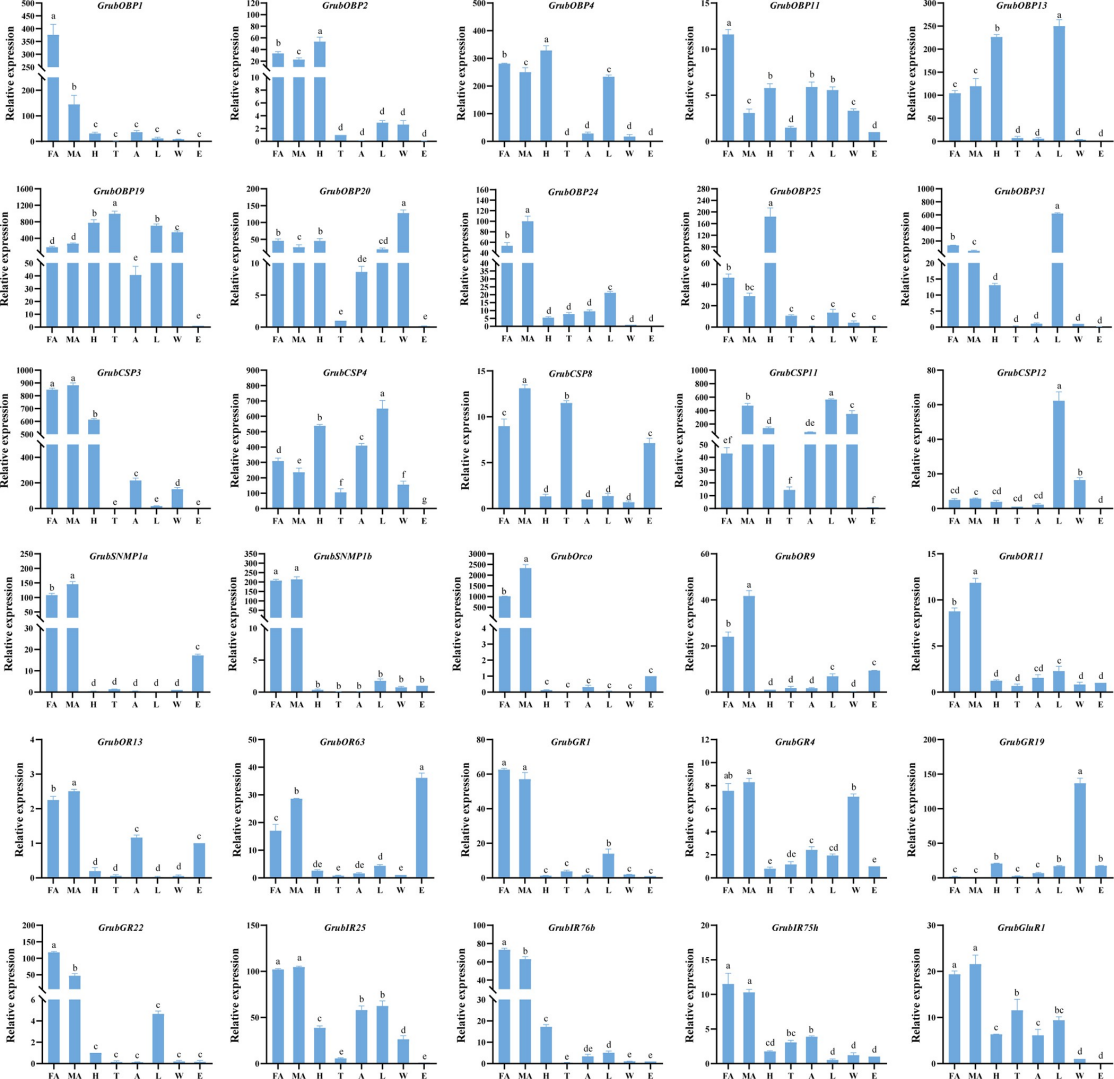
Tissue expression profiles of olfactory genes in *Graphosoma rubrolineatum*. FA: female antennae, MA: male antennae, H: heads without antennae, T: thoraxes, A: abdomens, L: legs, W: wings, E: eggs. The expression levels were estimated using the 2^^-ΔΔCT^ method. Relative expression levels are indicated as the mean + standard error (SE). The standard error is represented by the error bar, and different letters indicate a statistically significant difference between tissues (p < 0.05, ANOVA).

To further investigate the role of olfactory receptors in olfactory recognition in *G*. *rubrolineatum*, we utilized fluorescence qRT‒PCR to analyze the expression patterns of olfactory genes, including ten OBPs, five CSPs, five ORs, two SNMPs, four GRs, and four IRs, in different tissues (female antennae, male antennae, heads, legs, thoraxes, abdomens, and wings) (Fig 8). The results showed that all these genes were expressed diversely in all tissues but had lower expression in eggs. Among the olfactory genes examined, most were expressed mainly in the antennae. The differences in gene expression between male and female antennae were consistent with the transcriptome results. For instance, *GrubOBP11* was highly expressed in female antennae (male/female: 3.09/11.59), whereas *GrubOBP24* was highly expressed in male antennae (male/female: 100.15/53.42). Several genes were also highly expressed in other tissues. For example, *GrubOBP2*, *GrubOBP4*, and *GrubOBP25* exhibited high expression in the heads, while *GrubOBP13*, *GrubOBP31*, *GrubCSP4*, and *GrubCSP12* exhibited high expression in the legs. *GrubOR63* had the highest expression in the eggs, while *GrubOBP20* and *GrubGR19* were highly expressed in the wings (Fig 8). The diverse expression of these genes in different tissues may indicate that they perform various functions.

## Discussion

Insect antennae, as important olfactory organs, have been used as pivotal research material for constructing transcriptome libraries to obtain olfactory genes [50–52]. Although research on the olfactory genes of insect species has been developing rapidly, there has been little research on the olfactory mechanisms of *G*. *rubrolineatum*. In this study, we established the antennal transcriptome and identified 189 putative olfactory genes, including 31 OBPs, 15 CSPs, 4 SNMPs, 94 ORs, 22 GRs, and 23 IRs. We also examined their expression patterns in different tissues to gain a better understanding of their functions in *G*. *rubrolineatum*. The number of these genes varies among different species, including Hemiptera insects. The number of olfactory genes in *G*. *rubrolineatum* (31 OBPs, 15 CSPs, 94 ORs) was lower than that in *H*. *halys* (44 OBPs, 17 CSPs, 138 ORs) [53], *Ap*. *lucorum* (38 OBPs, 17 CSPs, 110 ORs) [51, 54], and *Ad*. *lineolatus* (34 OBPs, 19 CSPs, 88 ORs) [55–57], but higher than that in *Tr*. *elegans* (19 OBPs, 7 CSPs, 121 ORs) [58, 59] and *Ad*. *suturalis* (16 OBPs, 12 CSPs, 15 ORs) [60]. The differences in gene number among species may be due to complex environmental changes or the diversity of gene functions. In addition, the antennal cDNA library has difficulty fully representing the number of genes, particularly those with low expression [53]. Moreover, the number of olfactory genes in the antennal transcriptome (8 OBPs and 11 CSPs, 188 ORs) [61] of *R*. *pedestris* is lower than the number of genes identified in the genome (43 OBPs and 17 CSPs, 237 ORs) [62, 63]. This result indicates that more genes may be identified from the genome or multiple transcriptomes [64].

OBPs play a significant role in binding to odorants, which is considered the first critical step in olfactory signal transduction pathways [14, 65]. Many studies have shown that insect OBPs contain six cysteine sites, which form three disulfide bonds to maintain structural stability [66]. Classic OBPs have a conserved pattern of six cysteines that form three disulfide bridges. Minus-C OBPs lack one or two cysteines (C2 and C5), while plus-C OBPs have two extra cysteines and a characteristic proline after the sixth cysteine [11–13]. In our study, we identified 22 classic OBPs and five plus-C OBPs (GrubOBP1-4 and GrubOBP6). The remaining seven genes had incomplete sequences (5’ partial or 3’ partial), making it impossible to accurately determine their type. In addition, only classical OBPs and plus-OBPs have been described in other Hemiptera, whereas one minus-C OBP has been identified in *N*. *viridula* [67]. Moreover, the number of plus-C OBPs varies even within the same order; for example, *Ap*. *lucorum* (6), *Ad*. *suturalis* (4), *Ad*. *lineolatus* (2),*Tr*. *elegans* (5), and *N*. *viridula* (9) [51, 55, 59, 60, 67].

Expression profiling of olfactory genes is crucial for exploring the function and olfactory recognition mechanism of insects. OBPs in the antenna play critical roles in olfaction, such as recognizing sex pheromones or plant volatile components, and guiding normal behaviors such as feeding, mating, or oviposition [9, 68–70]. A heatmap based on the FPKM values suggested that eight OBPs were highly expressed in the antenna, indicating a possible role in olfaction (Fig 3B). Several OBPs have been reported to exhibit sex-biased transcript accumulation in other insects [54, 71]. In our study, the qRT‒PCR results indicated that seven OBPs (*GrubOBP1*, *GrubOBP2*, *GrubOBP4*, *GrubOBP11*, *GrubOBP20*, *GrubOBP24*, and *GrubOBP31*) exhibited sex-biased expression. *GrubOBP24* exhibited highly abundant expression in male antennae, indicating that these OBPs may play an important role in detecting pheromones released by females, although further verification is needed. The four genes (*GrubOBP1*, *GrubOBP2*, *GrubOBP4*, *GrubOBP11*) that are more highly expressed in the female antennae might be involved in oviposition site selection or other female-specific functions [67, 72]. OBPs are not restricted to olfactory tissues and have been proposed to be involved in other non-sensory functions [73–76]. Here, two OBPs (*GrubOBP13* and *GrubOBP31*) were highly expressed in the legs, suggesting their potential involvement in oviposition site preference and taste recognition [77–79]. Previous research has reported that CSPs are widely expressed in non-olfactory tissues as well as olfactory tissues, such as the head, legs, wings, gut, and pheromone gland [34, 80–82]. *BmorCSP2* was the most abundant genes in *Bombyx mori* tissues and exhibited a high affinity for most of the compounds tested, including both aliphatic and aromatic compounds [80]. In our study, *GrubCSP4* and *GrubCSP11* were also widely expressed in all investigated tissues (except for eggs), indicating that they may be involved in various physiological behaviors and binding to multiple compounds, although experimental verification is still needed (Fig 8).

In the insect olfactory system, ORs play crucial roles in detecting and recognizing odors [3, 25, 83]. Most functional insect ORs belong to the GPCR superfamily, which has seven transmembrane domains, especially in Drosophila [22]. In recent years, with the increasing use of insect transcriptome sequencing in gene identification, the number of transmembrane domains identified for ORs in many insects has mostly been 4–8 according to software predictions. For instance, 47 full-length AlucORs had 3–8 predicted TMDs in *Ap*. *lucorum* [54], while 93 HhalORs were full-length with 5–8 predicted TMDs in *H*. *halys* [53]. In our study, 79 GrubORs with full-length ORFs also contained 3–8 predicted TMDs (S4 Table). The varying number of TMDs may be attributed to alternative splicing during transcriptome sequencing [84]. Although ORs show great diversity in number and sequence, Orco is highly homologous among different insect species with seven transmembrane domains, and coexpressed with ORs to form heterodimers, which assist in olfactory recognition [25, 85]. As shown in Fig 5, Orcos genes are highly conserved among hemipteran insects and are clustered on one branch. Moreover, the greater expression of GrubOrco in the antennae than in the other tissues was essential for OR-mediated odor detection (Fig 8). Furthermore, the FPKM values revealed that six GrubORs displayed significantly high expression levels in the antennae of both sexes, indicating their potential role in odorant recognition.

## Supporting information

S1 Fig (TIF)

S2 Fig (TIF)

S3 Fig (TIF)

S4 Fig (TIF)

S5 Fig (TIF)

S1 Table Primer sequences for chemosensory genes used in qRT-PCR. (XLSX)

S2 Table Summary of the *Graphosoma rubrolineatum* antennal transcriptome. (XLSX)

S3 Table Summary of de novo assembly of the *Graphosoma rubrolineatum* antennal transcriptome. (XLSX)

S4 Table Summary of olfactory genes in *Graphosoma rubrolineatum* antennal transcriptome. (XLSX)

S5 Table Sequences used in phylogenetic trees. (DOCX)
