# Supplementary figures and images for: Identification and expression profiles of olfactory-related genes in the antennal transcriptome of Graphosoma rubrolineatum (Hemiptera: Pentatomidae)
Source: PLoS One. 2024 Aug 6;19(8):e0306986. doi: 10.1371/journal.pone.0306986 (PMC11302851; doi:10.1371/journal.pone.0306986)

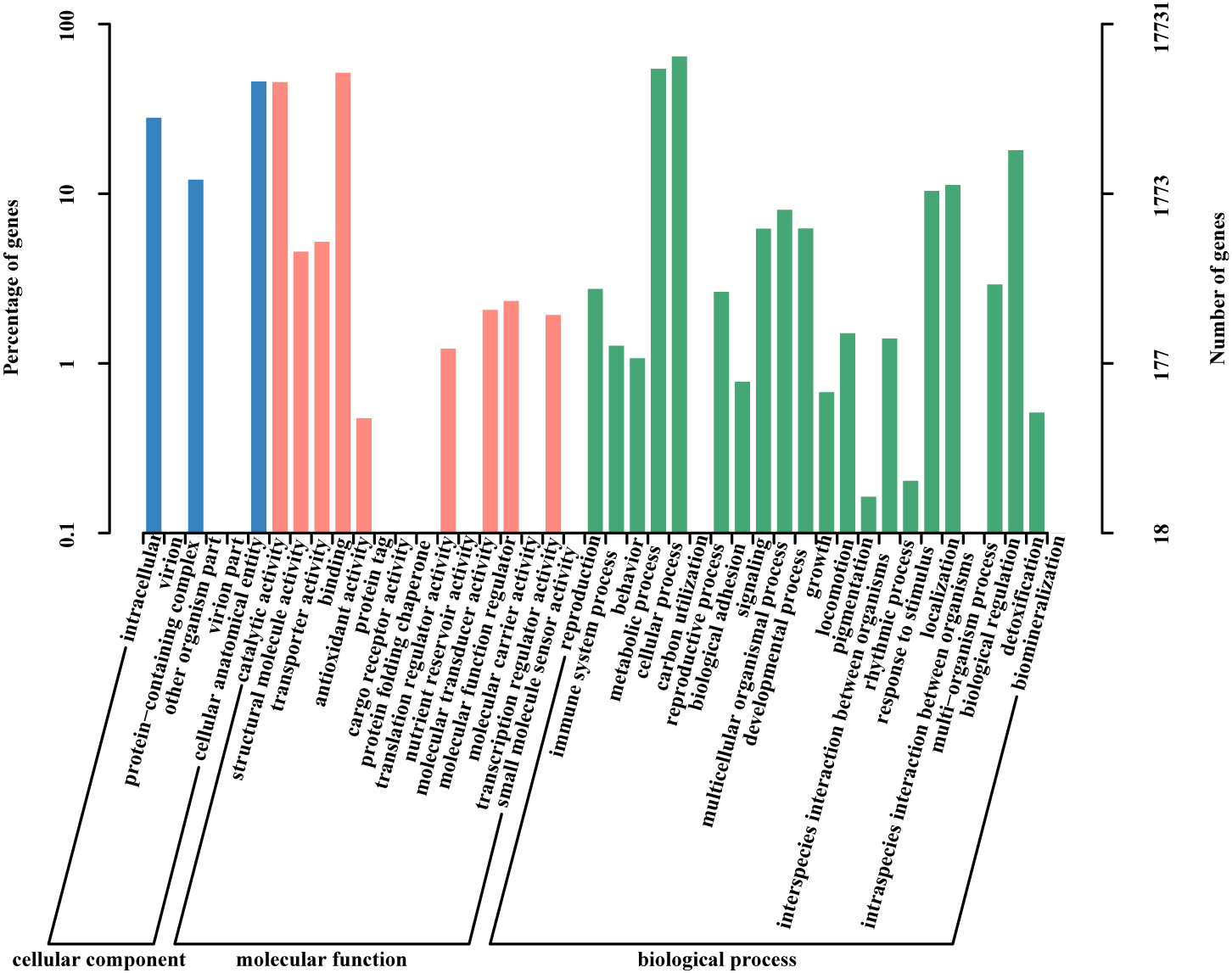

Supplement: S1 Fig — (TIF) [file pone.0306986.s001.tif]

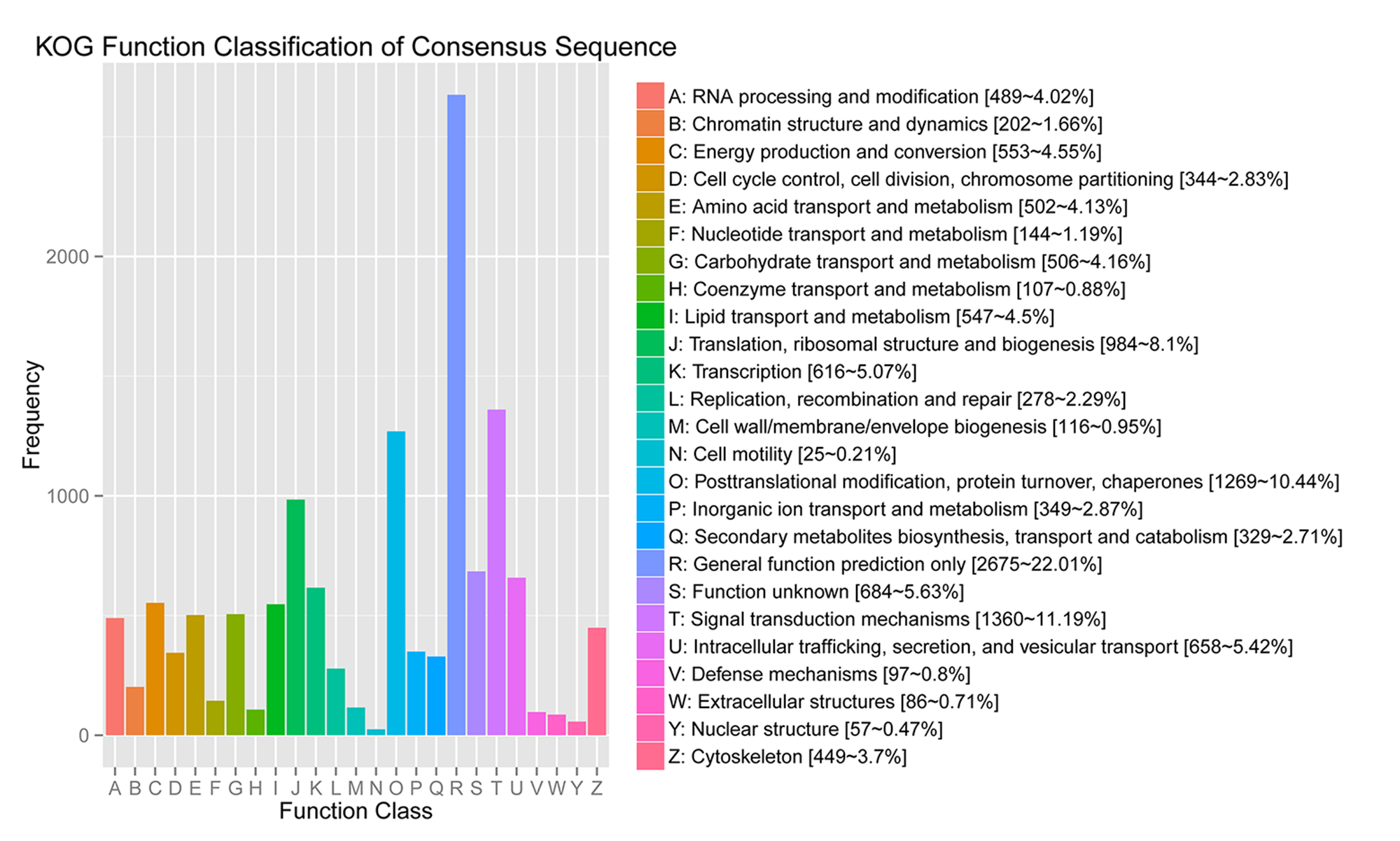

Supplement: S2 Fig — (TIF) [file pone.0306986.s002.tif]

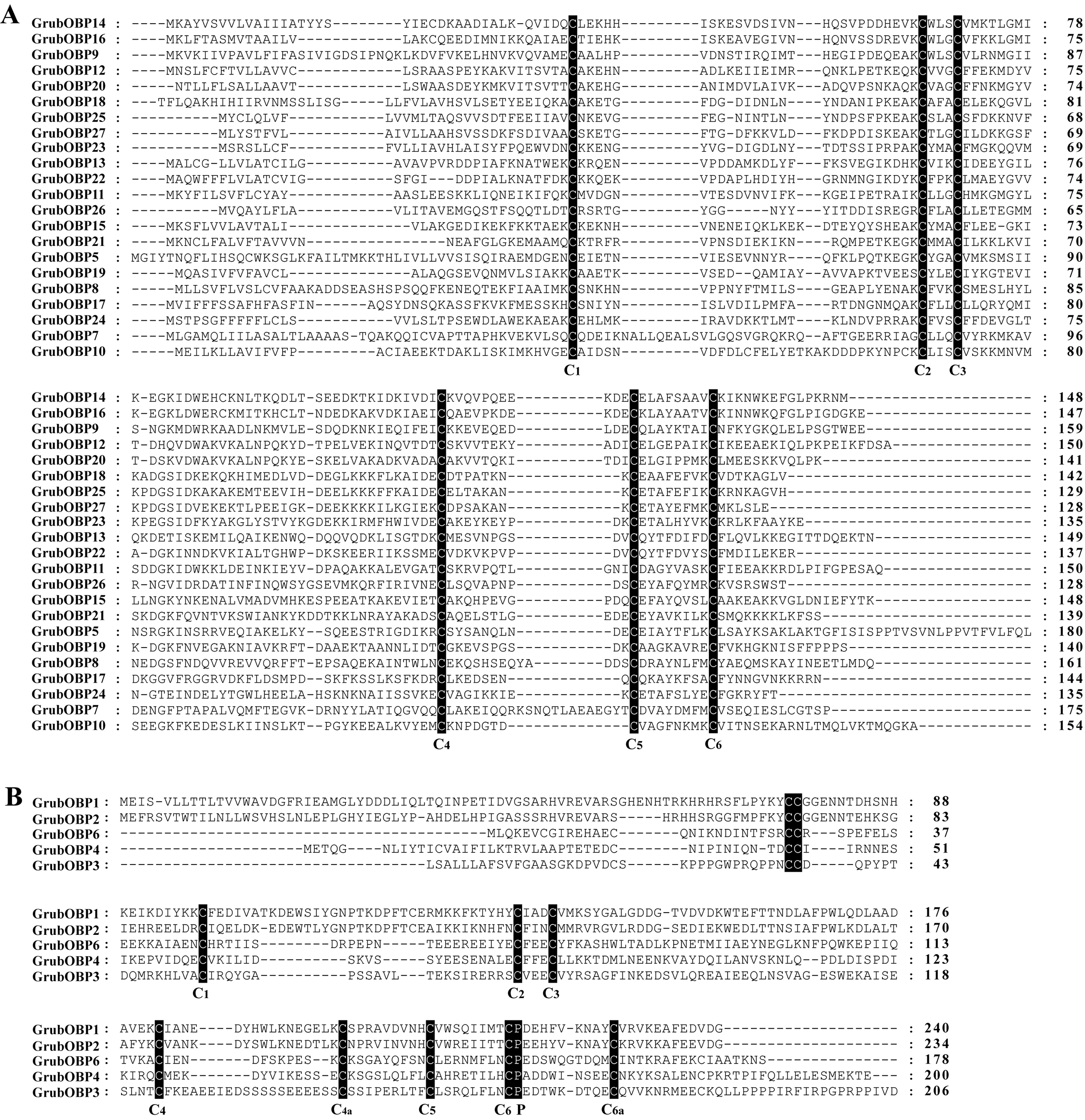

Supplement: S3 Fig — (TIF) [file pone.0306986.s003.tif]

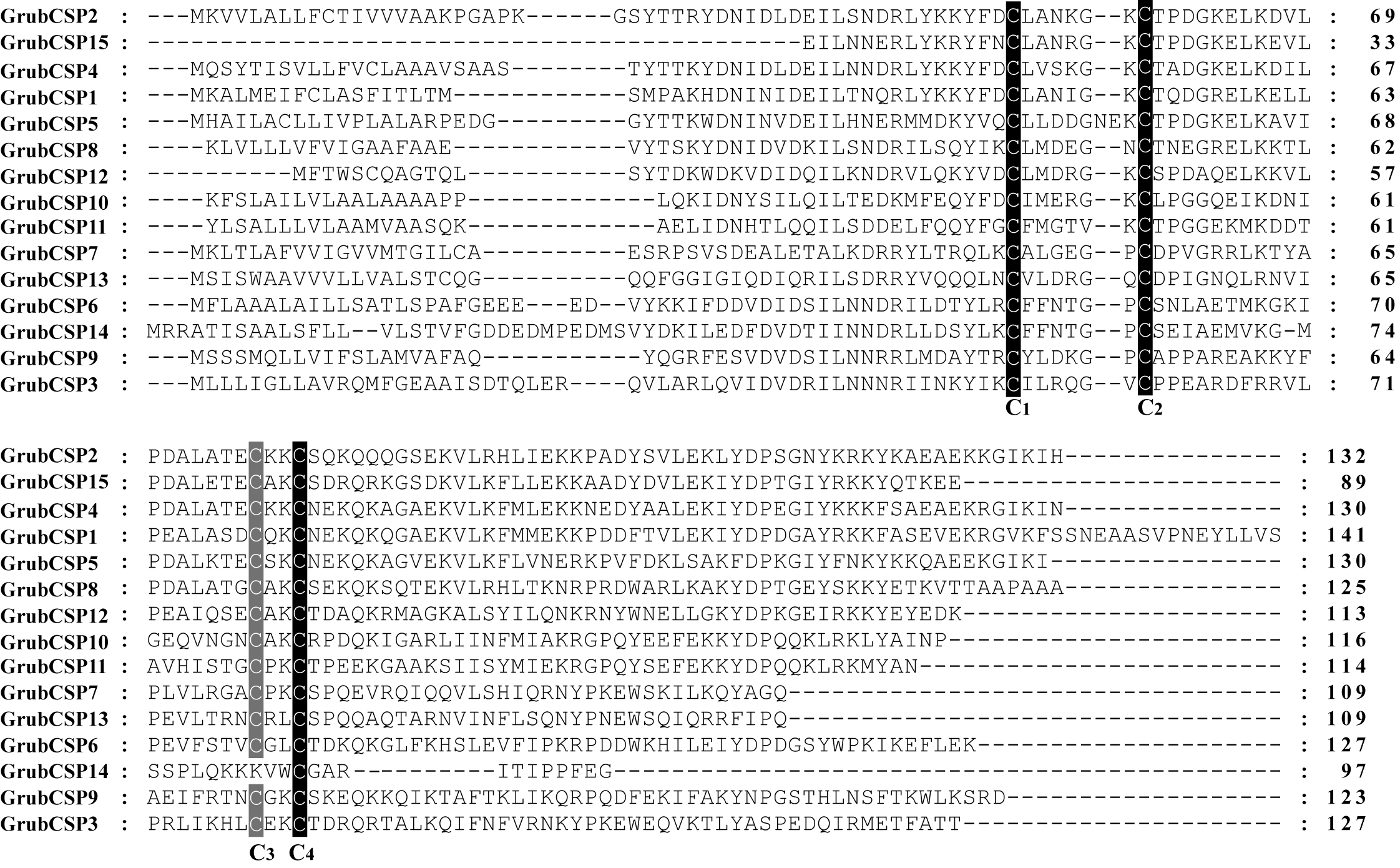

Supplement: S4 Fig — (TIF) [file pone.0306986.s004.tif]

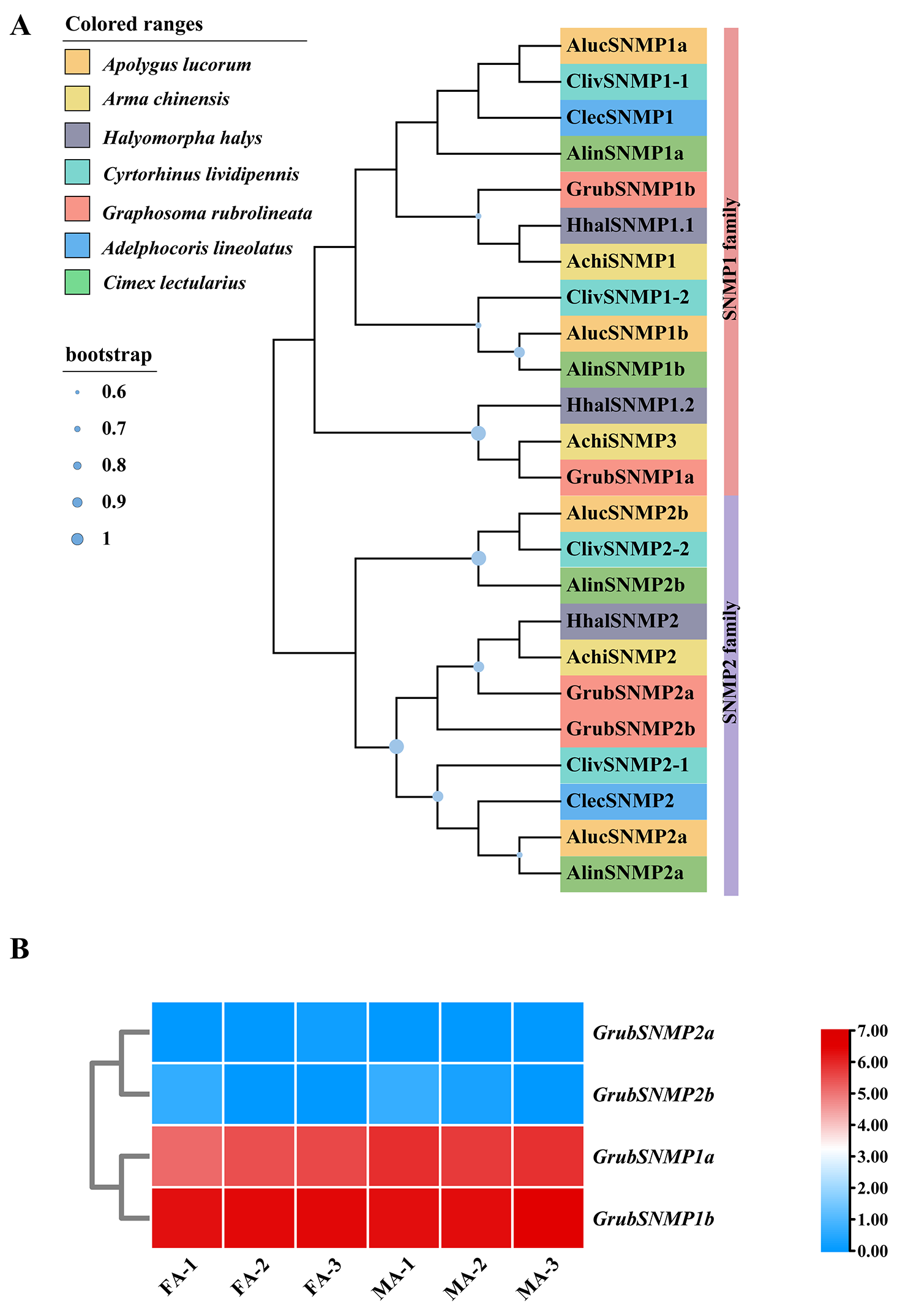

Supplement: S5 Fig — (TIF) [file pone.0306986.s005.tif]
