# Supplementary material for: Identification and expression profiles of olfactory-related genes in the antennal transcriptome of Graphosoma rubrolineatum (Hemiptera: Pentatomidae)
Source: PLoS One. 2024 Aug 6;19(8):e0306986. doi: 10.1371/journal.pone.0306986 (PMC11302851; doi:10.1371/journal.pone.0306986)
Supplement: S5 Table — (DOCX) [file pone.0306986.s010.docx]

**S5 Table. Sequences used in phylogenetic trees**

**OBPs**

>GrubOBP1

MEISVLLTTLTVVWAVDGFRIEAMGLYDDDLIQLTQINPETIDVGSARHVREVARSGHENHTRKHRHRSFLPYKYCCGGENNTDHSNHKEIKDIYKKCFEDIVATKDEWSIYGNPTKDPFTCERMKKFKTYHYCIADCVMKSYGALGDDGTVDVDKWTEFTTNDLAFPWLQDLAADAVEKCIANEDYHWLKNEGELKCSPRAVDVNHCVWSQIIMTCPDEHFVKNAYCVRVKEAFEDVDG*

>GrubOBP2

MEFRSVTWTILNLLWSVHSLNLEPLGHYIEGLYPAHDELHPIGASSSRHVREVARSHRHHSRGGFMPFKYCCGGENNTEHKSGIEHREELDRCIQELDKEDEWTLYGNPTKDPFTCEAIKKIKNHFNCFINCMMRVRGVLRDDGSEDIEKWEDLTTNSIAFPWLKDLALTAFYKCVANKDYSWLKNEDTLKCNPRVINVNHCVWREIITTCPEEHYVKNAYCKRVKKAFEEVDG*

>GrubOBP3

LSALLLAFSVFGAASGKDPVDCSKPPPGWPRQPPNCCDQPYPTDQMRKHLVACIRQYGAPSSAVLTEKSIRERRSCVEECVYRSAGFINKEDSVLQREAIEEQLNSVAGESWEKAISESLNTCFKEAEEIEDSSSSSEEEESSCSSIPERLTFCLSRQLFLNCPEDTWKDTQECQVVKNRMEECKQLLPPPPIRFIRPGPRPPIVDQ*

>GrubOBP4

METQGNLIYTICVAIFILKTRVLAAPTETEDCNIPINIQNTDCCIIRNNESIKEPVIDQECVKILIDSKVSSYEESENALECFFECLLKKTDMLNEENKVAYDQILANVSKNLQPDLDISPDIKIRQCMEKDYVIKESSECKSGSLQLFLCAHRETILHCPADDWINSEECNKYKSALENCPKRTPIFQLLELESMEKTE*

>GrubOBP5

MGIYTNQFLIHSQCWKSGLKFAILTMKKTHLIVLLVVSISQIRAEMDGENCEIETNVIESEVNNYRQFKLPQTKEGKCYGACVMKSMSIINSRGKINSRRVEQIAKELKYSQEESTRIGDIKRCSYSANQLNDECEIAYTFLKCLSAYKSAKLAKTGFISISPPTVSVNLPPVTFVLFQLG*

>GrubOBP6

MLQKEVCGIREHAECQNIKNDINTFSRCCRSPEFELSEEKKAIAENCHRTIISDRPEPNTEEEREEIYECFEECYFKASHWLTADLKPNETMIIAEYNEGLKNFPQWKEPIIQTVKACIENDFSKPESKCKSGAYQFSNCLERNMFLNCPEDSWQGTDQMCINTKRAFEKCIAATKNS*

>GrubOBP7

MLGAMQLIILASALTLAAAASTQAKQQICVAPTTAPHKVEKVLSQCQDEIKNALLQEALSVLGQSVGRQKRQAFTGEERRIAGCLLQCVYRKMKAVDENGFPTAPALVQMFTEGVKDRNYYLATIQGVQQCLAKEIQQRKSNQTLAEAEGYTCDVAYDMFMCVSEQIESLCGTSP*

>GrubOBP8

MLLSVFLVSLCVFAAKADDSEASHSPSQQFKENEQTEKFIAAIMKCSNKHNVPPNYFTMILSGEAPLYENAKCFVKCSMESLHYLNEDGSFNDQVVREVVQRFFTEPSAQEKAINTWLNCEKQSHSEQYADDSCDRAYNLFMCYAEQMSKAYINEETLMDQ*

>GrubOBP9

MKVKIIVPAVLFIFASIVIGDSIPNQKLKDVFVKELHNVKVQVAMECAALHPVDNSTIRQIMTHEGIPDEQEAKCWLSCVLRNMGIISNGKMDWRKAADLNKMVLESDQDKNKIEQIFEICKKEVEQEDLDECQLAYKTAICNFKYGKQLELPSGTWEE*

>GrubOBP10

MEILKLLAVIFVFPACIAEEKTDAKLISKIMKHVGECAIDSNVDFDLCFELYETKAKDDDPKYNPCKCLISCVSKKMNVMSEEGKFKEDESLKIINSLKTPGYKEEALKVYEMCKNPDGTDCVAGFNKMKCVITNSEKARNLTMQLVKTMQGKA*

>GrubOBP11

MKYFILSVFLCYAYAASLEESKKLIQNEIKIFQKCMVDGNVTESDVNVIFKKGEIPETRAIKCLLGCHMKGMGYLSDDGKIDWKKLDEINKIEYVDPAQAKKALEVGATCSKRVPQTLGNICDAGYVASKCFIEEAKKRDLPIFGPESAQ*

>GrubOBP12

MNSLFCFTVLLAVVCLSRAASPEYKAKVITSVTACAKEHNADLKEIIEIMRQNKLPETKEQKCVVGCFFEKMDYVTDHQVDWAKVKALNPQKYDTPELVEKINQVTDTCSKVVTEKYADICELGEPAIKCIKEEAEKIQLPKPEIKFDSA*

>GrubOBP13

MALCGLLVLATCILGAVAVPVRDDPIAFKNATWEKCKRQENVPDDAMKDLYFFKSVEGIKDHKCVIKCIDEEYGILQKDETISKEMILQAIKENWQDQQVQDKLISGTDKCMESVNPGSDVCQYTFDIFDCFLQVLKKEGITTDQEKTN*

>GrubOBP14

MKAYVSVVLVAIIIATYYSYIECDKAADIALKQVIDQCLEKHHISKESVDSIVNHQSVPDDHEVKCWLSCVMKTLGMIKEGKIDWEHCKNLTKQDLTSEEDKTKIDKIVDICKVQVPQEEKDECELAFSAAVCKIKNWKEFGLPKRNM*

>GrubOBP15

MKSFLVVLAVTALIVLAKGEDIKEKFKKTAEKCKEKNHVNENEIQKLKEKDTEYQYSHEAKCYMACFLEEGKILLNGKYNKENALVMADVMHKESPEEATKAKEVIETCAKQHPEVGPDQCEFAYQVSLCAAKEAKKVGLDNIEFYTK*

>GrubOBP16

MKLFTASMVTAAILVLAKCQEEDIMNIKKQAIAECTIEHKISKEAVEGIVNHQNVSSDREVKCWLGCVFKKLGMIKEGKLDWERCKMITKHCLTNDEDKAKVDKIAEICQAEVPKDEKDECKLAYAATVCKINNWKQFGLPIGDGKE*

>GrubOBP17

MVIFFFSSAFHFASFINAQSYDNSQKASSFKVKFMESSKHCSNIYNISLVDILPMFARTDNGNMQAKCFLLCLLQRYQMIDKGGVFRGGRVDKFLDSMPDSKFKSSLKSFKDRCLKEDSENQCQKAYKFSACFYNNGVNKKRRN*

>GrubOBP18

TFLQAKHIHIIRVNMSSLISGLLFVLAVHSVLSETYEEIQKACAKETGFDGDIDNLNYNDANIPKEAKCAFACELEKQGVLKADGSIDKEKQKHIMEDLVDDEGLKKKFLKAIDECDTPATKNKCEAAFEFVKCVDTKAGLV*

>GrubOBP19

MQASIVFVFAVCLALAQGSEVQNMVLSIAKKCAAETKVSEDQAMIAYAVVAPKTVEESCYLECIYKGTEVIKDGKFNVEGAKNIAVKRFTDAAEKTAANNLIDTCGKEVSPGSDKCAAGKAVRECFVKHGKNISFFPPPS*

>GrubOBP20

NTLLFLSALLAAVTLSWAASDEYKMKVITSVTTCAKEHGANIMDVLAIVKADQVPSNKAQKCVAGCFFNKMGYVTDSKVDWAKVKALNPQKYESKELVAKADKVADACAKVVTQKITDICELGIPPMKCLMEESKKVQLPK

>GrubOBP21

MKNCLFALVFTAVVVNNEAFGLGKEMAAMQCKTRFRVPNSDIEKIKNRQMPETKEGKCMMACILKKLKVISKDGKFQVNTVKSWIANKYKDDTKKLNRAYAKADSCAQELSTLGEDECEYAVKILKCSMQKKKKLKFSS*

>GrubOBP22

MAQWFFFLVLATCVIGSFGIDDPIALKNATFDKCKKQEKVPDAPLHDIYHGRNMNGIKDYKCFPKCLMAEYGVVADGKINNDKVKIALTGHWPDKSKEERIIKSSMECVDKVKPVPDVCQYTFDVYSCFMDILEKER

>GrubOBP23

MSRSLLCFFVLLIAVHLAISYFPQEWVDNCKKENGYVGDIGDLNYTDTSSIPRPAKCYMACFMGKQQVMKPEGSIDFKYAKGLYSTVYKGDEKKIRMFHWIVDECAKEYKEYPDKCETALHYVKCKRLKFAAYKE*

>GrubOBP24

MSTPSGFFFFFLCLSVVLSLTPSEWDLAWEKAEAKCEHLMKIRAVDKKTLMTKLNDVPRRAKCFVSCFFDEVGLTNGTEINDELYTGWLHEELAHSKNKNAIISSVKECVAGIKKIEKCETAFSLYECFGKRYFT*

>GrubOBP25

MYCLQLVFLVVMLTAQSVVSDTFEEIIAVCNKEVGFEGNINTLNYNDPSFPKEAKCSLACSFDKKNVFKPDGSIDKAKAKEMTEEVIHDEELKKKFFKAIDECELTAKANKCETAFEFIKCKRNKAGVH*

>GrubOBP26

MVQAYLFLAVLITAVEMGQSTFSQQTLDTCRSRTGYGGNYYYITDDISREGRCFLACLLETEGMMRNGVIDRDATINFINQWSYGSEVMKQRFIRIVNECLSQVAPNPDSCEYAFQYMRCKVSRSWST*

>GrubOBP27

MLYSTFVLAIVLLAAHSVSSDKFSDIVAACSKETGFTGDFKKVLDFKDPDISKEAKCTLGCILDKKGSFKPDGSIDVEKEKTLPEEIGKDEEKKKKILKGIEKCDPSAKANKCETAYEFMKCMKLSLE*

>GrubOBP28

MFYSQLVLAIGLLATHSVWSETFNDIEAACNKETGFTGNVEKINFSDSSIPKEAKCTLACILEKKGAFKPDGSIDKDIEKQIPEEIVKDAELKKKYMKGIEKCDASAKANKCD

>GrubOBP29

MLIVTDAIKDIMNHIQVAEDEEVKCWISCVMNELAIMKERAIDWVRFEDILTSGLADEEETKVHQLVEACHSQVDQEDKNECQVAYSLADCKIKHWNELGLPKGNWEDLAA*

>GrubOBP30

MYNGLNITKDGKFVEIAAKGLAQHRFANSPDELTKANNMIEACTKEAVVKDANEKCAIGRLVRECFVKNGSKVIILIPSMYDNNIMNNN*

>GrubOBP31

MACVMEKMGLLKDGKVDMDKTLEMNKQKFKNPEDLEKANEIARRCANGKLYYQYYFMLFPQIIL*

**CSPs**

>GrubCSP1

MKALMEIFCLASFITLTMSMPAKHDNINIDEILTNQRLYKKYFDCLANIGKCTQDGRELKELLPEALASDCQKCNEKQKQGAEKVLKFMMEKKPDDFTVLEKIYDPDGAYRKKFASEVEKRGVKFSSNEAASVPNEYLLVS*

>GrubCSP2

MKVVLALLFCTIVVVAAKPGAPKGSYTTRYDNIDLDEILSNDRLYKKYFDCLANKGKCTPDGKELKDVLPDALATECKKCSQKQQQGSEKVLRHLIEKKPADYSVLEKLYDPSGNYKRKYKAEAEKKGIKIH*

>GrubCSP5

MLLLIGLLAVRQMFGEAAISDTQLERQVLARLQVIDVDRILNNNRIINKYIKCILRQGVCPPEARDFRRVLPRLIKHLCEKCTDRQRTALKQIFNFVRNKYPKEWEQVKTLYASPEDQIRMETFATT*

>GrubCSP4

MQSYTISVLLFVCLAAAVSAASTYTTKYDNIDLDEILNNDRLYKKYFDCLVSKGKCTADGKELKDILPDALATECKKCNEKQKAGAEKVLKFMLEKKNEDYAALEKIYDPEGIYKKKFSAEAEKRGIKIN*

>GrubCSP3

MHAILACLLIVPLALARPEDGGYTTKWDNINVDEILHNERMMDKYVQCLLDDGNEKCTPDGKELKAVIPDALKTECSKCNEKQKAGVEKVLKFLVNERKPVFDKLSAKFDPKGIYFNKYKKQAEEKGIKI*

>GrubCSP6

MFLAAALAILLSATLSPAFGEEEEDVYKKIFDDVDIDSILNNDRILDTYLRCFFNTGPCSNLAETMKGKIPEVFSTVCGLCTDKQKGLFKHSLEVFIPKRPDDWKHILEIYDPDGSYWPKIKEFLEK*

>GrubCSP7

MKLTLAFVVIGVVMTGILCAESRPSVSDEALETALKDRRYLTRQLKCALGEGPCDPVGRRLKTYAPLVLRGACPKCSPQEVRQIQQVLSHIQRNYPKEWSKILKQYAGQ*

>GrubCSP8

KLVLLLVFVIGAAFAAEVYTSKYDNIDVDKILSNDRILSQYIKCLMDEGNCTNEGRELKKTLPDALATGCAKCSEKQKSQTEKVLRHLTKNRPRDWARLKAKYDPTGEYSKKYETKVTTAAPAAA*

>GrubCSP9

MSSSMQLLVIFSLAMVAFAQYQGRFESVDVDSILNNRRLMDAYTRCYLDKGPCAPPAREAKKYFAEIFRTNCGKCSKEQKKQIKTAFTKLIKQRPQDFEKIFAKYNPGSTHLNSFTKWLKSRD*

>GrubCSP10

KFSLAILVLAALAAAAPPLQKIDNYSILQILTEDKMFEQYFDCIMERGKCLPGGQEIKDNIGEQVNGNCAKCRPDQKIGARLIINFMIAKRGPQYEEFEKKYDPQQKLRKLYAINP*

>GrubCSP11

YLSALLLVLAAMVAASQKAELIDNHTLQQILSDDELFQQYFGCFMGTVKCTPGGEKMKDDTAVHISTGCPKCTPEEKGAAKSIISYMIEKRGPQYSEFEKKYDPQQKLRKMYAN*

>GrubCSP12

MFTWSCQAGTQLSYTDKWDKVDIDQILKNDRVLQKYVDCLMDRGKCSPDAQELKKVLPEAIQSECAKCTDAQKRMAGKALSYILQNKRNYWNELLGKYDPKGEIRKKYEYEDK*

>GrubCSP13

MSISWAAVVVLLVALSTCQGQQFGGIGIQDIQRILSDRRYVQQQLNCVLDRGQCDPIGNQLRNVIPEVLTRNCRLCSPQQAQTARNVINFLSQNYPNEWSQIQRRFIPQ*

>GrubCSP14

MRRATISAALSFLLVLSTVFGDDEDMPEDMSVYDKILEDFDVDTIINNDRLLDSYLKCFFNTGPCSEIAEMVKGMSSPLQKKKVWCGARITIPPFEG*

>GrubCSP15

EILNNERLYKRYFNCLANRGKCTPDGKELKEVLPDALETECAKCSDRQRKGSDKVLKFLLEKKAADYDVLEKIYDPTGIYRKKYQTKEE*

>HhalCSP1

MRLIISCLLLIGLALAKPDTTKTDNKKEGYTTKYDNIDLEEILNNIRLLDKYFNCLIEKGKCTTDGKELKEIVPDALKTGCSKCNEKQKAGVEQVLRYLIEKKRDYFDELAKKYDPEGIYLKKYEAEAEKRGIKL

>HhalCSP2

MKVVLALLICTTVLAAKPGAHKGSYTTRYDNIDLEEILSNERLYKKYFDCLANKGKCTPDGKELKDVLPDALATECKKCSHKQQQGTEKVLRHLIEKRPVDYSVLEKLYDPTGTYKRKYKAEAEKRGIKIH

>HhalCSP3

MQSYTISALLFVCLAAAVSAATTYTTKYDNIDLDEILSNDRLYKKYFDCLISKGKCTADGKELKDILPDALATECKKCNEKQKAGAEKVLKFMLEKKAEDYAALEKIYDPEGAYKKKYAAEAEKRGIKIN

>HhalCSP4

MFIAAALAILLSATLTPSFGDDQEDIYKKIFDDVDIDGILNNDRILDTYLRCFFNSGPCSNLADTIRGKIPEVFSTVCGLCTEKQKGLFKHSLDIFIPKRPDDWKHILEIYDPDGSYWPKIKEFLETY

>HhalCSP5

MLLLIGLLAVRQMFGEAAVSDTQLERQVLARLQVIDVERILNNNRIINKYLKCMLRQGVCPPEARDFRRVLPKLIKHLCEKCTDRQRTALKQIFTFVRTKFPKEWEQMKILYATNPEDQIRMEKFAAT

>HhalCSP6

MRVILAVILFAGVALARPDGYTTKYDNINLDEILNNDRLYQKYFQCLTNKGKCTPDGKQLKDILPDALKSKCAKCNERQRKGAEKVFKYLLDKKPNDYKVLEKIYDPQGVYRAQYKSEAEKRGIKI

>HhalCSP7

MRVILACLLFVGLVFAKPDGYTTKYDNIDLEEILHNERLYKKYYDCLANKGTCTPDGKELKDIVPDALKTGCKKCNEKQKKGVEKVLKYVLENKRADYDYLEKIYDPQGIYRKKYQAEAEKHGIKI

>HhalCSP8

MLRTLLLLAPLVLACFCQAAATQQSYTDKWDRIDVDQILKNDRILKKYVDCLMDRGKCSPDAQELRKVLPEAIQTECAKCTDSQKRMAGKALSYILQNKRNYWNELIGKYDPKGEFRKKYEYEEDK

>HhalCSP9

MKLVLLLVFVIGAAFAAEVYTSKYDNIDVDKILSNDRILSQYIKCLMEEGNCTNEGRELKKTLPDALATGCAKCSEKQKAQTEKVLRHLTKNRPRDWARLKTKYDPTGEYSKKYEPKATTAAPAAA

>HhalCSP10

HDSLSARINHYVGMNPTLVFVVIGVVMTGILCAESRPSVSDEALETALKDRRYLTRQLKCALGEGPCDPVGRRLKTYAPLVLRGTCPKCSPQEVRQIQQVLSHIQRNYPKEWSKILKQYAGQ

>HhalCSP11

MKAVLALALFAAVAVARPGDKYTTKYDNIDLDEILNNERLYKRYFNCLANRGKCTPDGKELKAVLPDALETECAKCSDRQKKGSDKVLKFLLEKKAADYDVLEKIYDPTGIYRKKFTMKKE

>HhalCSP12

MKVVIALLLFAAVAIAKPADMYTTKYDNIDIDEILNNERLYKKYIDCVMDKGDCTPDGKELRKNIPDAIATDCAKCSEHQKTGTDKVLNYMLKNKKADYDELEKKFDPKGEYRKRHNIKA

>HhalCSP13

MKLVILLLVVLAAVASADKYTTQYDNIDIDEILSNDRLYKKYYDCLMGKGKCTPDGQELKKNMPDAITTDCSKCSEKQKVGSQKVLKFMLDKKESDYTNLEKVFDPTGTYRKKHAQS

>HhalCSP14

VIVIGLFILTILAPAAPLELLPPKVDGHDVTQILTDDKLFQQYFDCVMGRTKCTPGGQIVKDGIPAQLKDGCANCPPIRRIGAQIIVRFMIATRCPQYEEFEKKYDPQQKLRKLYAA

>HhalCSP15

MMLVLSLLMVSVAAALPADTYTTKYDNLDVGEILKNDRLYQKYNECLSNTGTCTPDGKELKDILGEIIKTDCKKCSEKQKKNIVKFLKQILEEKPEDFVKLEKIYDPDQVFRKKYAS

>HhalCSP16

MSSKAPSPLCDTVAMRRTSFLVAVCMLFSLSAVLADEEDIDVYEKILEDFDVDTIINNDRLLDSYLKCFFNTGPCSDIAEMVKGKIPEVFSTVCGLCTEKQKGLFKHSLDIFIPKRP

>HhalCSP17

MRFTVALFAFVVFAAAAPQSAKIDKYDIEQIFNDEKLFQQYFNCVMGRGQCTPGGQKLKDSIADHVKSGCANCPPERKARAQKMVKFMIAKKAPQYEEFEKMYDPEQKLRKLYA

>NvirCSP1

MRLILACLLFVGLVFAKPDGYTTKYDNIDLDEILSNERLYKKYFDCLANKGPCTPDGKELKDVIPDALKTGCKKCNEKQKKGAEKVFRYILKNKRADYDVLEKIYDPEGIYRKKYEAEAEKHGIKL

>NvirCSP2

MRVIFACLLFVGLALAMPDTSKTDTKKDGYTTKYDNIDLDEILNNTRLLDKYFNCLVEKDKCTTDGKELKDIVPDALKTACSKCNEKQKGGVEKVLRFLIEKKRDYYDELSKKYDPEGIYLKKYEAEAQKRGIKI

>NvirCSP3

MRAILACLLFVSLALARPDGYTTKWDNIDVDEILHNDRMMENYEKCLLEEGNEKCTPDGKELKAIIPDALKTGCSKCNEKQKAGVEKVLKYLVKERRPLFDKLAAKFDPKGFYLKKFKDEADKHGIKY

>NvirCSP4

MELVLALLVVSVAAAIPADTYTTKYDNLDVGEILKNERLYKKYQECLYDTGTCSPDGKELKDVLAEIIKTDCKKCSEKQKNNIVKYLKVVLTEKPDDYLKLEKIYDPDQIFRKKYSS

>NvirCSP5

MRVILAVILFAGVALARPDGYTTKYDNIDLDEILNNDRLYQKYFQCLTNKGKCTPDGKQLKDILPDALKSKCAKCNERQKKGAEKVFKHLLDKKPNDYKTLEKIYDPQGTYRAQYKSEAEKKGIKI

>NvirCSP6

MRRASFFAVVCSLLAVSTVFADEEEEEPPYDVYDKILEDFDVDTIINNDRLLDSYLKCFFNTGPCSEIAEMVKGKIPEVFSTVCGLCTDKQKGLFKHSLDIFIPKRPDDWKHILEIYDPDGSYWPKIKEFLETH

>NvirCSP7

MLRTLLLLAPLLLAWSCQAATAPSYTTRWDTIDIDQILKNDRILKKYIDCLMDRGKCSPDAQELKKVLPEAMQTECAKCTDAQKRLAGKALSYILQYKRNYWNELLGKYDPKGEFRKKYEYEDK

>NvirCSP8

MKLLLLFVVVVGTVFAAEVYTSKYDNIDVDKILSNDRILSQYIKCLMDEGNCTNEGRELKKTLPDALATGCAKCSEKQRSQTEKVLRHLTKNRPRDWARLKTKYDPTGEYSKKYEPKATTAAPAAA

>NvirCSP9

MRISSAAVLVLLVALNSCGGQQLGSIGPLDIQKILSDKVYVQQQLNCVLDQGQCDAVGNQLRRVIPEVLERNCRFCTPQQAQNARNVVNYISQNYPDEWSQIQRRFSRQ

>NvirCSP10

MLLLIGLLAVRQMFGEAAISDTQLERQVLARLQVVDVDRILNNNRIINKYIKCILRQGVCPPEARDFRRVLPRLIKHLCEKCTDRQRTALKQIFTFVQSKFPKEWEQVKTLYASPEDQIRMEKFATT

>NvirCSP11

MKLTLAFVVIGVVLTGILCAESRPAVSDEALETALKDRRYLTRQLKCALGEGPCDPVGRRLKTYAPLVLRGTCPKCSPQEVRQIQQVLSHIQRNYPKEWSKMLKQYAGQ

>NvirCSP12

MKFASALVVLAAFAAADAQSSFKLDGHEIQQVLADDNLYHQYFNCVMDKGKCTPDGQDLKDHIPDHLNGGCANCTPERKERAKTLVRFMVTKKNHDFEEFEKKYDPKHMMRKQYGQ

>NvirCSP13

DLDEILNNDRLYEKYFKCIMGKGKCTPDGKELKNDIPDAIKTDCSKCSDRQKEGTDKVLKFMLANKKADYAVLEKTYDPA

>AchiCSP1

TSWAIGVVLLVALESCKGDLLTQGQKQRINSADVKRMLTDRNYVLKQINCVLHDVECDEIGLHLKRVIPDVLIKNCASCSPQEAQTARNIINYIRGKYPNEWAEIQS

>AlucCSP1

MLKVLVLLAAVVCCVSAAATYTSKYDNIDLDEILSNTRLYKKYFDCLANKGKCTPDGKELKESLPDALKTNCAKCTKKQQEGTDKVLRHVLKNKPNDYKVLESIYDPTGIYRKKYEIEAEKRGIKLPGSH

>AlucCSP2

MASKLSVVLLIGAVGMVLAADKYTDKYDNIDLDEILGNQRLYQKYFDCIQGKGKCTPDGAELKETIPEALKTECAKCSDKQKAGVEKVLRHLIREKPDDYKVLEDQFDPEGVYRKKYEDLKKKVEEGKPIEY

>AlucCSP3

MKVAVLVLLCVGAALSAEVYTSKYDNIDVDKILSNDRILTQYIKCLMEEGNCTNEGKELKKTLPDALASGCTKCSEKQKAQTEKVLRHLSKNRPRDWNRLKNKYDPKGEYSKKYEKEAKAISA

>AlucCSP4

MKFVAALFVASVAVLAVEAADQYTTKYDNIDLDDILKNQRLYKKYFECLTNKGKCTPDGKELKEHLPDALKTGCSKCSEKQRAGSEKVIKHLLKNKPSDYAILEKIYDPQGSYKKKYEAEAKKLGINV

>AlucCSP5

MVGKLSVVLLIGAVGMVLAAELYTDKYDNIDVDEILGNQRLYQKYFDCIQGKGKCTPDGAELKKNIPEALQTDCAKCSEKQKAGVEKVLRHLINEKPEDYKVLEEQFDPEGVYRKKYEHLKKKVEEGKPV

>AlucCSP6

MVSKLSIVLLIGALADVWASELYTDKYDNIDVDEILGNQRLYQKYFDCIQGKGKCTPDGAELKKNIPEALQTDCAKCSEKQKAGVEKVLRHLINEKPEDYKVLEEQFDPEGVYRKKYEHLKKKVEEGKPIEY

>AlucCSP7

MVSKLSIVLLLGALADVWAAELYTDKYDNIDIDEILNNDRMYKNYFNCVMGNGKCTPDGLELKAKIPEALQTECAKCSDKQKKGAEKVLRFIINQKKDDYKLLEEKFDPEGVYRKKYEAQKKLAEEGKPIEY

>AlucCSP8

MLKVLVLLANAASTYTTKYDNIDLDEILSNQRLYKKYYDCLANKGKCTPDGKELKEALPDALKTNCSKCSKKQQEGTDKVLRYVLKNKPNDYKVLENIYDPSGNYRKRYEDEASKRGIKLPGSH

>AlucCSP9

MLKIFVLLAAAVCLANAASTYTTKYDNIDLDEILSNQRLYKKYYDCLANKGKCTPDGKELKEALPDALKTNCSKCSKKQQEGTDKVLRYVLKNKPNDYKVLENIYDPSGNYRKRYEDEASKRGIKLPGGH

>AlucCSP10

MNTSALLKVGILLGCIAACLAAETRSSVSDEALEAALKDKRYLTRQLKCALGEGACDPVGRRLKTYAPLVLRGACPKCTPSEVRQIQQVLSHIQRHYPKEWAKILKQYAGQ

>AlucCSP11

MVWLLLCFLLFAAGRAEDMTQEDMEFYTRVFEEVDPDFILDNERILTSYLKCFYSEIECNAHAEVVKKSIPNVLATVCGRCSDKQKGIFKYSLNKFIPAHPKDWERILSIYDPTGEAWPKVKEFIES

>AlucCSP12

MKIFLFSLLLVSVATVVLCAEVYPDKYDSIDLDEILSNQRLYQKYFDCVMGKGKCTPDGTELKDKIPEALQNECAKCSEKQKKGAEKVLRFLITEKKDDFKLLEEKFDPEGKYRKKFGELKKKIEEGKPVTV

>AlucCSP13

MKILLVLGALVAAVASQGGLPFLTPSEVNRLISDSNYVKNQINCILGKAKCDSLGNQLKLAIPEVLGRNCKNCDAQQAANARKVTDFMRNNFPAEFGQILKRYRIRQSHSYGR

>AlucCSP14

MRRGEGTMIRLTVVLLTIASVARTGVCTRLPYSTHYDYIDVDQVLNNSRLYTKYFECLMGQGKCTPEAKELRDKLPEALQTNCGICSERQAKESHKVIRFLINQRPEDFKMLEAKYDPSGLYMKRYQEEMKLNGAFS

>AlucCSP15

MLIFVVFGVSSLFVGLEGAPLQYSDTRYDDVELTSILNNDELYIKLFQCLIGRGKCTPDWEILKDAVPGALLDNCEKCTAKEKFGTKTLLSHLVHEKPSDMRILEGEFDPDGSYRKELEKEEKETNDINRKRSALKEEVEFLNKVKRLIK

>AlucCSP16

MRIIYAFLVVLACGLASSEMTEEEFYEKVFEEVDPDFILDNERILTTYLKCFYNEIECNVHAEVVKKSIPNVLATVCGRCSDKQKGIFKYSLNKFIPAHPKDWERILSIYDPTGEAWPKVKEFIES

>AlucCSP17

MLMRIVITIAAVLTVSLALVTREMREREFFRQLEGINVDSILINRRLIDKYIKCLLKTGKCDPTMKDLRIALPLILEHLCETRCSERERQNLRKLFLYIRTNRAQEWERLAKLYDPKGAYKANVDAFIENRPRPTMITSSAIRETVAPTIFTTTTTTTTTTTRAPTAPTPQRQSIRATRNALYIAMRRFAMYATTPRKRNRHNLTTPVI

>AlinCSP6

MFYKLSVVVLMGILAGVWAADKYTDKYDNIDIDEILTNERLYKKYFDCIQGTGKCTPDGIELKEKIPEALKTECAKCNEKQKAGVEKVMRYLITKKPEDFKILEDKFDPEGVYRKKYEAQRKLVEEGKPVEY

>AlinCSP5

MGHLTIVLLAAAFEVLTGSRAYTTHYDYIDVDQVLNNTRLYTKYVECLLGQGKCTPEARELRDKLPEALQTNCARCSERQASESHRVIRFLIQNRQEDFKLLEAKYDPSGLYFKRFEEETKRNVSLS

>AlinCSP4

MRIILSAFLVAMACSLATCEMTEEEFYTKVFEEVDPDFILDNERILTSYLKCFYNEIECNAHAEVVKKSIPDVLATVCGRCSDKQKSIFKYSLNKFIPAHPKDWEKILSIYDPSGEAWPKVKAFIES

>AlinCSP1

MLKVLVLLAAVVCCVSAAATYTSKYDNIDLDEILSNTRLYKKYFDCLANKGKCTPDGKELKESLPDALKTNCAKCTKKQQEGTDKVFRHVLKNKPNDYKVLESIYDPPGIYRKKYEAEAEKRGIKLPGSH

>AlinCSP2

MKVAVLVLLCVGAALSAEVYTSKYDNIDVDKILSNDRILTRYIKCLMEEGNCTNEGKELKKTLPDALASGCTKCSEKQKAQTEKVLRHLSKNRPRDWALLKTKYDPKGEYSKKYEKEAKALTA

>AlinCSP3

MISKLSMVLLIGAFADVWAAEQYTDKYDNIDIDEILNNDRMYKNYFHCVMGNGKCTPDGLELKAKIPEALQTECAKCTDKQKKEVEKVLRFIINQKKDDYKLLEEKFDPEGVYRKKYEAQKKLVEEGKPIEY

>AlinCSP18

MIWILLVAVSMTTSLAEEESIDYYRVFEEIDPDLILDNERILQTYLKCFYGEGPCNTHAQLAKESIPDVLA

>AlinCSP21

KELKEHIKEALENECGKCTEAQKKGTRRVIGHLINHEADFWNELTAKYDPERKYTTKYEKELKEVKA

>AlinCSP20

PDGLELKEAIPDALKTDCSKCSDKQKAGVEKVLKFLLTKKPEDYKILESQFDSEGVYKKKYEALRKQ

>AlinCSP19

MVYKSSVVFLLMGTVAYVWGEKYTEQYDDINLDNILTNERLYRIYFKCILSKGKCTPEGEVLKKAIPDALK

>AlinCSP17

MDYKFLVVMQMGVISSVCAAGPYTDKYDNVNLDEVLNNERLYRNYFNCLQGKGKCTLDGAILKEIIPSALKTDCALCSVRQKKGAEKVLIFLITKKPDDFKILEDKF

>AlinCSP16

MLPFYVFSLCAVFVACQETYTSKYDNVNVEDALKNDRLYKAYFNCLADRGPCTREGNMLKEALPDGLRNNCSLCTDPQRRGTHQVIRFLFKYRPEDMKLLEEIYDPEGIYKTKYAEERKKLME

>AlinCSP15

MKLIVAVALLCVVAESWAASTYTDKWDNINVDEILESQRLLKAYVDCLLDRGRCTPDGKALKETLPDALENECSKCTDKQKSGSDKVIRHLVNKRPEMWKELSAKYDPNNIYQDRYKDKIEAVKGQ

>AlinCSP14

MNSAIVLCVVALAGMVLARPDDTYTTKYDNVDLDEILGNDRLLVPYIKCTLDEGKCAPDAKELKEHIREALENGCAKCTDKQKEGTRRVIAHLIKHKNADWQKLKAKYDPEGKYTHKYEKELEEVQH

>AlinCSP13

MKFVAALLVASVAVLAVEAANQYTTKYDNIDLDDILKNQRLYKKYFECLTGNGKCTPDGKELKEHLPDALKTGCSKCSEKQRAGSEKVIKHLLKNKPQDYAVLEKIYDPSGIYKKKYEAEAKKLGINV

>AlinCSP12

MMIIIVFGISALLVVVEGAPLQYSDTRYDDVELTTILSNDELYIKLFQCLIGRGKCTPDWEILKDALPGALLDNCSECSNKQKFGTKTLLAHLVHERPSDMRLLEGEFDPDGSYRKELEKEEKESNDINRKRSANLEEVEILDKIKRIIK

>AlinCSP7

MNYKLSVILLIGVLASVWAASTYTDKYDNIDLDEILTNERLYKKYFDCIQGKGKCTPDGTELKEAIPDALKTECAKCNAKQKAGVEKVLRHLLTKKAEDYKILEDKFDPEGVYRKKYEAQKKLADEGKPIVL

>AlinCSP8

MDYKLSVMLLMGVLACAWAADKYTDKYDNIDIDEILNNERLYKKYFDCILGNGKCTPDGTELKETIPDALKTACAKCNDKQKAGVEKVLRHLLTKKAEDYKILEAKFDPEGVYRKKYEAQKKLAEEGKPIAL

>AsutCSP8

MDYKLSVMLVMGVLACAWAADMYTDQYDNIDIEEILTNERLYKKYFDCIIGNGKCTPDGTELKETIPDALKTACAKCNDKQKAGVEKVLRHLLTKKAEDYKILEAKFDPEGVYRKKYEAQKKLAEEGKPIVL

>AsutCSP7

MVSKLSMVLLIGALADVWASELYTDKYDSIDIDEILNNDRMYKNYFNCVMGNGKCTPDGTELKAKIPEALQTECAKCSDKQKKGVEKVLRFLIKEKKDDYKLLEEKFDPEGVYRKKYEAQKKLVEEGKPIEY

>AsutCSP6

MVCKLFAVVLMGILAGVWAADKYTDKYDNIDIDEILTNERLYKKYFDCIQGIGKCTPDGIELKEKIPEALKTECAKCNEKQKAGVEKVMRYLITKKPEDFKILEDKFDPEGVYRKKYEAQRKLVEEGKPVEY

>AsutCSP5

MDYKFFVVMQIGVISSVCAAGTYTDKYDNVNLDEVLNNERLYRNYFNCLQGKGKCTLDGAILKEVIPSALKTDCALCSVRQKKGAEKVLIFLITKKPDDFKILEDKFDPEGVYRKKYEAQRKLVEEGKPIH

>AsutCSP4

MRIILSAFLVAMACSLATCEMTEEEFYTKVFEEVDPDFILDNERILTSYLKCFYSEIECNAHAEVVKKSIPDVLATVCGRCSDKQKSIFKYSLNKFIPAHPKDWEKILSIYDPSGEAWPKVKAFIES

>AsutCSP3

MKFVAALLVASVAVLAVEAANQYTTKYDNIDLDDILKNQRLYKKYFECLTGKGKCTPDGKELKEHLPDALKTGCSKCSEKQRAGSEKVIKHLLKNKPQDYAVLEKIYDPSGIYKKKYEAEAKKLGINV

>AsutCSP2

MGHFPPVFSLSPVLLVASLHTMNTSTLLKIAFLLGCVAACLAAETRSSVSDEALEAALKDKRYLTRQLKCALGEGACDPVGRRLKTYAPLVLRGACPKCTPSEVRQIQQVLSHIQRHYPKEWAKILKQYAGQ

>AsutCSP1

MLPFYVFSLCAVFVACQETYTSKYDNVNVEDALKNDRLYKAYFNCLADRGPCTREGNMLKEALPDGLRNNCSLCTDPQRRGTHQVIRFLFKYRPEDMKLLEEIYDPEGIYKTKYAEERKKLME

>ClivCSP5

MRPLLWTALVLFLSSVVLGYESKSGLPFNIDVEAILNNKRLLDAYSRCYLDEGPCPGPSREAKKYLGEIFRTNCAKCTKDQKKQTRIAFRKLKEKRPQDFARVFAKYNPGNTHLASFNAWLKSHEP

>ClivCSP4

MFVAVVLCAFGGALGAPVQYSDTRFDGLELSSILKNDELYIQLFQCLIGRGKCSPDWELLKDAVPGALLDNCVDCSEKQKISMKTLLAHLVHHKPEDMRLLEGEFDPDGSYRKSLEKLDKDEEEKQTNVIQTKRAARLKDVEILEKVKRIK

>ClivCSP3

MKVAALFLLCVGVALSADVYTSKYDNIDVDKILSNDRILTQYIKCLMEEGNCTNEGKELKKTLPDALASGCTKCSEKQKAQTEKVLRHLSKNRPRDWARLKNKYDPKGEFSKKYEGKSLTA

>ClivCSP2

MKIALILFVLAAVVSCALAADQYTTRYDNIDLDDILKNQRLYKKYYECLVGKGKCTPDGKELKEHLPDALKTGCSKCSEKQRSGSEKVIKYLLKNKPQDYAALEKIYDPQGSYKKKYEAEAKKTRHPSVNLLTFVPAILLLFEIV

>ClivCSP1

MLATWTTLLLLGAFGITFGEQYTDKYDNIDLDSILSNDRLFKKYFECMTGEGKCTPDGAELKAVIPDALKTECAKCNEKQRKGTEKVLRFLITKKADEYKILEDQFDPEGIYRKKYEAQKKNVLEGKPIEIPGTS

>ClivCSP12

MEGQTMRFALLVASIFTVAAAIVTREMREREFFRHLEAIDVDGITNNQRLVDKYIKCLLKTGKCDPAMKDLRISLPLILEHMCETRCSERERTNLRKLFMYIRTNRPLEWDRLSKLYDPKGTYKPRVDAFVENRPRISTTTQLPTINRSTTILPSPR

>ClivCSP11

MKILVVAVCFIAVASAASTYTTKYDNIDLEEILTNQRLYKKYFDCLTNKGKCTPDGKELKDALPDALKTSCSKCTKKQQEGTDKVIRYMLKNKVQDYKMLESLYDPSGNYRKKYDEEAQKRGIKLPK

>ClivCSP10

MKFVILLVVASIAMCLGEDKYSDEYDSVDLDEVLNNKRLYANYISCILGKGKCSADAKYLKETIPDALQTGCTKCSEVQKKRVGKMLKFVKENHSDDYSSLLEKYDPEGQYKDLYKDLVA

>ClivCSP9

MVPSCVTLMLCVLFTLISIVSSAENKYTSKYDKVDVDAIIKNERILKRYVDCLMDRSSCTPDAKLLKALLPDALQTNCAKCTDAQKIMAGKVLGHLLQFKRPYWDELTKKYDPDGSFRKRQGYDEDPDDIDYSNYDDSSRP

>ClivCSP8

MLLLYGLCAFVALNLSAGAPQEDLSFYEQVFEEVDPDMILDNERILQSYLKCFYSEIECNPHAAVVKKSIPEVLSTVCGKCSDKQKSIFKYSLNKFIPAHPKDWDRILSIYDPSGEAWPKVKAFMES

>ClivCSP7

MKSLFCALVSVALFVAVKGMTTDEFYSKVFEEVDPDFILDNERILRSYLNCFYSESECNSHAAVVKESIPEVLSTVCGKCSDKQKSIFKYSLNKFIPAHPKDWDRILSIYDPSGEAWPKVKAFMES

>ClivCSP6

MKLFAFAVLLVCAVFVTGQESYPDRYDNIDVDEILSNQRLYQKYFDCVMGKGKCTPDGAELKDKIPEALKNECAQCNEKQKKGAEKVLRFLITQKKDDFKLLEEKYDPEGIYRKKYEEQRKALEEGKDVKV

**SNMPs**

>GrubSNMP1a

MSAKKAQFSDVVLERIKAVPQRLKEAPPKKYGKLGAALVLGGIGFGWLVFPYILSFAVGKIVTLDDGSDVRNIWKKIPQYLDFNIYIFNITNPMEVQNGGKPVLQEIGPYRYIEWKEKVHLVDDVAEDTITYSNLNTWYFLKEKSLPLTGDELVTIPHLPLLSVLLVAEKSFPMPALTIVDAAVPDIYGKLGSVFLTAKVKELLFDGILIDCTARNIVPKAICIAIKQNSKALVKLGKNKYLFSFFGIRNATPEETRITVKKGVQDVYSIGKVVAMNGNTQNVVWSGGECRRFSGTDSTIFPPFRKPDNYSIVAFSPEICRTMSGTYVGEAEYQGVRGYRYEVSLGDMKRNPGEMCFCPSPDKCLGKGTTDLAKCQGAPLIGSLPHFYDAEEEYLNGVVGLKPDKDKHEITFIMEPISGVPLLARKRLQFNIHLHPVRFVDLTKKLTPTLVPLFWIEENLDLGDELMGFLEANLLTNLRLVDVVKWMLIVVGAGVCVAGVVVYRMKKRAQDKTNSNEDLLPPGNDDVEILDPSRLSNSNSTASLVPSLTVPTAPVTHTGWDTNRSPSLQLPAVETITASPGISSSAPIEPRTRASAEQHSRLSTGRLRKF*

>GrubSNMP1b

MAAPLRLGVAGAVLSFIGIIFGFWGFHKFLAYKINQNVALKKGNEMRATWSKFPIPVEFRIYIFNVTNPEEVHTGQKPKMQEVGPYFFDEWKEKVNLEDDPAEDTVSFNQRTAWVFQESRSEGLTGEEMLTIPHPALLSMVLTVEKQKPGALAMISKALPALFNNPSTVFLTAKAMDILFRGIPINCSSTEFGPKAICTMLRANPKGLKKLNDDIFLFSFFGTKNYTIDEGRFTVKRGVRDAKEVGKVVKFNGKETQDIWSGPECNALRGTDSTIFPPFIEDSDEIVSFAPDLCRSLGAKFQHKMVYKGIPGNYYTADLGDMSSNPEEKCFCPTPTTCLKKGAFDISKCVGAPIVLTLPHFYSTDPSYLEGVEGLHPDREKHQIFLNFEPMTATPLGARKRLQFNLPIHPIKKVALMKELPEALVPLFWVEEGLELDQKFIDLLDAKLFRTIRIVGVGKWVIIVLGFAMIGGGVGLHYYRKNKIKGPVNKVSPPPSKF*

>GrubSNMP2a

MKGLLELQGLSRVVYTGSAGCLIIVLSIYFGIAGFPYLLQQQIQSRIALNNGSEGMKAWENLPLPLEFKIFIFNVTNPDEVSNGEQPVVQELGPYVYDQYRRKVDIEFTEDDTISYKIEKKFYFNKNKSGCHKESDVVVVPNIPLIATAYGIEERFPMGLVFINTSAPLLFPGIKNIFITTTIGDLLFNGVRIKCDYLKGPAMPVCQGIKRNRPPSLKEIPSSGDLAFSYFSAANTSVSGVFKVYRGNKNVYDLGEIVKYDNHTDLPMWDKNTTCSVLKGTDSTILPPLQNKDQEIYIFLPEVCLSLKAVSYGETSMYGIDVYKYMASPHNFDSGITNPSNICRCKKQEDEPKAPPMCLKDGAIDASKCQGAPVVFTYPHMLFADPEYQNYVEGYKRDHGKHQTEVFIEPRTGVPLAAFKRIQMNIFLRRLEDVDIFANVSEGLFPLLWLEEALTEELVQTYLPDMKEMISTTHIIMSVTGLLIGVGIFCLLVALILYLKHRKIACIQENQVVSNISLIGAGIATRKDPDTVEKRIGYDLPNTVGSDRITIQKF*

>GrubSNMP2b

TDVVTVLNAPLLVMAYLFENEFPENMSYVNMAIPHLFPEKQDLCIEVVVKDLLFDGIRISCGDDNVHTKNICDEIRRKEIPSIRQIPNSEDPAFSYFYSYNTSRSKFYKITRGNINILELGYIVEYDNQTYLSMWPNDSICSNIKGTSDWEFFPPLQENPHPKIHLFFPEMCLSMYAFESDEEMFGMKLIGYFVSSHNFNSDEVKCLCKENRQNGTSESCTGRGV

>HhalSNMP1.1

MAAPLRLGVAGAVLSLVGIIFGFWGFHKFLAYKINQNVALKKGNDMRAAWSKFPIPVEFKIFLFNVTNPQEVHTGQKPKLQEIGPYFFDEWKEKVKLEDDPAEDTVSFNQRTAWIFQESRSEGLTGEEMITIPHPALLSMVLTVEKQKPGALPMISKALPALFNSPSTVFLTAKAMDILFRGVPINCSSNDFGPKAICTMIRANPKGLKKLNDDIFLFSFFGEKNYTIDEGRFTVKRGVRDAKEVGTVVKFNGKEIQDVWSGPECNALRGTDSTIFPPFIDDSEDIVSFAPDLCRSLGAKFKHKIVYKGIPGNHYTADLGDMSSNPEEKCFCPTPDTCLKKGAFDISKCVGAPIVLTLPHFYETDPSYLATVEGLHPDKEKHQIFLNFEPMTGTPLGARKRLQFNIPIHPIKKVALMKELPDALVPLFWVEEGLELDQKFIDLLDSQLFRALRIVGVSKWVIIVLGLAMIGGGVGLHYYRKNKMNSPTVTQVSPPPNKF

>HhalSNMP1.2

SPQYFHCSEYFIRQSRYPPLQSVLSMSAKKAQFSDVVLERIKAVPQKLKEAPPKKYGKVGAALVLGGVGFGWLIFPYILSFAVGKIVTLDDGSDVRKIWKKIPQYLDFNIYLFNITNPMEVQNGGKPILQEVGPYRYIEWKEKVNLVDDIGEDTITYSNLNTWYFLKEKSLPLTGDEMVTIPHLPLLSVLLVAEQDFPTPMLTIVNAAVPHIYGKLGSVFMTAKVRELLFDGVLIDCTAKNIVPKAICIAIKQNSKALVKLGKNKYLFSFFGIRNATPEDVRITVKKGVQDVYSIGKVVAMNGNTENVVWSGGECRRFSGTDSTIFPPFRKPDNYSIVAFSPEICRTMSGSYVGEGAYQGVRGYRYVVSLGDMKRNPGEMCFCPSPDRCLGKGTTDLTKCQGAPLIGSLPHFYDAEEEYLNGVVGMKPDKDKHEITFIMEPISGVPLLARKRLQFNIHLHPVRFVNLTKKLTPTLVPIFWLEENLDLGDELMGFLEANLLTNLRLVDVVKWMLIVVGAGVCVAGVVLYRMKKEANKKTNSREDLLPPGNDDVEVLDPSRLSGSTASLVRSLVQPTAPVTHTAWDVVPSPSLQLPAIETIGASPGISSSAPLEPRARSTAEALHSSLSTGRLRKF

>HhalSNMP2

MKGLMELRGLSRVVYTGSAGCFIIVLSIYFGIAGFPYLLQQQIKSKIVLNNGSEGMEAWESLPLPLEFKVFIFNVTNPDEVSKGMQPVVQELGPYVYDQYRRKVNIEFTEDDTISYRIEKKFYFNKNKSGCYRESDVVVVPNVPLIGTAYRVEERFPMGLIFINSSASLLFPGIKNLFLTTTVGDLFFNGVRIKCDYLKGPAMPVCQGIKRNLPPSLKEIPLSRDFAFSYFSDANSSVSGVFKTYRGNKNVYDLGRIIKYDNNTHLTMWDKNTTCSELKGTDSTILAPIQNKDQDIYIFLPEVCLSLKAVFSRATNMYGIDVYQYMASHHNFDSEKRNPSHICRCKKQEDEPNAPPMCLKDGAIDASKCQGAPVVFTYPHMLFADPEYQNYVKGYKGDYEKHQTEVFIEPRTGVPLAAFKRIQMNIFLRRLEDVDLFANISEGLFPLIWIEEALTEELVQTYLSDMKELMSTTRIIMSVTGLLIGVGIFCLLVALILYIKHRNVACMKENQVVSNISLIGHGIVTRKDPDTAEKRIAYDLPNSLSSDRLTVQKF

>AchiSNMP1

SDMAAPLRLGVAGAVLTFLGIIFGFWGFHKFLAYKINQNVALKKGNDMRAAWSKFPIPLEFRIYLFNVTNPQEVHAGQKPKLQEVGPYFFDEWKEKIKLEDDPAEDTVSFNQRAAWVFQESRSEGLTGEEVITIPHPALLSMVLTVEKQKPGALPMISKALPALFNSPSTVFLTAKVMDILFRGVPINCSSSDFGPKAICTMIRANPKGLKQLNDDIFLFSFFGVKNNTMEEGRFTVRRGIRDAKEVGSMVKFNGKEMQDVWSGPECNALRGTDSTIFPPFIDDSEDIVSFAPDLCRSLGAKFRHKIVYKGIPGNHYTADLGDMSSNPEEKCFCPTPTTCLKKGAFDISKCVGAPIVLTLPHFYETDQSYLDTVDGLHPEKEKHQIFLNFEPMTGTPLGARKRLQFNIPIHAIKKVALMKELPDALIPLFWVEEGLELDQKFIDILDAQLFRALRIVGIGRWVMVVLGLAMIGGGVGLHYYRKNKMIPPMVTQVSPPPNKY*

>AchiSNMP2

KAQIALNNGSEGMKAWENLPLPLEFKVFIFNITNPDEVSKGMKPVVQELGPYVYDQYRRKVDIEFNEDDTISYRIEKKFYFNRNKSGCHRESDVIVVPNLPLIGTAYGIEERFPMGLVFINSTAHLLFPGIKNLFLTTTVGDLLFNGVRIKCDYMKGPAMPVCQGIKRNLPPSLKEIPSSGDFAFSYFSDANSSVTGVFKAYRGNENVYDLGRIVEYNNHSDLIMWDKNTTCSELRGTDSTILPPIQNKDQDIYIFLPELCLSLKAVFSKETTMYGIDVYHYIASHHNFDSEKTNPSNICRCKKQEDDPTSPPTCLKDGAIDASRCQGAPVVFTYPHMLYADPEYQNFVEGYHSDYGKHQTEVFIEPRTGVPLAAFKRVQMNIFLRRLNDVDLFANISEGLIPLIWIEEALTEELVQTYLPDMKEMMSITRIIMSVTGLLIGIGIFCLLVALILYLKHRKVECIQENQVVSNISLIGTGKATRNDPDAMGKRISYDLPNTVGNDRVTVQKL*

>AchiSNMP3

YIYGKLGSVFMTAKVRELLFDGVLIDCTAKNIVPKAICIAIKQNSKALVKLGNNKYLFSFFGIRNATPEEARITVKKGVEDVYSIGKVVAMNGNPENVVWSGGECRRFSGTDSTIFPPFRKPDNYSIIAFSPEICRTMTGVYVGDGEYQGVRGYKYEVSLGDMKRNPGEMCFCPSPDRCLGKGTTDLTKCQGAPLIGSLPHFYDAEEDYLNGVEGMNPIKEKHEISFIMEPISGVPLLARKRLQFNIHLHPVRFINLTRKLTPTLVPIFWLEENLDLGDELMGFLEANLLTNLKLVDVVKWMLIVVGAGVCVAGVVLYRMKKEASKKADSKEDLLPPGSD

>AlucSNMP1a

MGAPLRLGVTGGVLFLFGSIFGFWGFNKFLNSQIAQTVQLKKGNEMRDNWAKFPIALEFKIYLFNLTNPDEVQEGGKPKVQEVGPYFFYEWKSKGKLEDDPSDDTVSFNMKAVWYFQKDKSEGLTGDEIINIPHPVVFSMIMTVERDKPGALPMLAKALPALFNNLTSPFVSARAMDILFDGLPINCASKEFGPKAVCTLINANPKGLVKKSPELFLFSFFGPKNGTLDEGRFTVKRGINDPKEVGMMVKFNNKTKLDVWSTDECNQLTGTDSTIFPPFIDDSEDIVSFSPDLCRSLGAKFRYKITYKGVPGNHYTADLGDMSANEDEKCYCPTPTTCLKKGALDITKCAGAPIVLTLPHYYLADPSYLDEVEGLHPEEEKHQIFLNFEPITGTPLGARKRLQFNIKSHAVKKIPFMKTLPTTMIPVMWIEEGVELDQKFIDILNANLFRVMKIVGVSKWVMMLLGIGMGGFGAFLYYKRRGAAGGSEKPPTPKTVQVESISSGKF

>AlucSNMP2b

MNVGILRFRNDSLSETFEVYRGNKDFNKIGQIVSIDGKRSLNHWYGEGCNKVAGSYDESLLQPFLTQDSISNVYGSDICTSLPMSVVGSMSYKGVDCLKFSPDKKFLGSVVDYPENFCYCPGSIEGITLGQSCLRTGAMEFAACQAVPVVLGFPHFYRASSRYQNAVGGLSPHQDKHESFVALEPTTGIPIEGAKRIQINFQVKGTPAMTMTKNAPDTLMPFLWLDEQVELGDEQLSMIKDTLLKMLKIFNIVNWVLIAVGSLMVFVGCLMSFLSARRERSHPD

>AlucSNMP1b

MTSHKSESRMSKMSESKMSQFPRSTKLSREQQGSRAKSPVFMNLMERMREMPTKIKEAPPKKFGKFGAAMVAGGVGFGWVAFPYILSFAISKMVNLAPGGEIHDIWKDIPQSLDFNFWVWNVTNPMEVQRGAKPVLQEVGPYRYIEWKKKVDLVDNPADDEITYSSLNTWYFQKEKSYPLTGDEIVTIPHLPIMSMLLVAEQDFPPAMLTLLNAAIPKIYGRMDSIFLNIKVRDLLFDGYPIDCTSRDLIGRTVCVAVKANSKPLVKNGRNKYLFSVLGTKNGTPEDVRLTVKKGTMNTFDIGKVVKINGSPLNTVWKDECNVLDGTDATIFPPYRSADNISIVAYATDICRSIRGSYIGEGTYNGVKGHQYVVNLGDMSKNPKDACFCVKKCYKKGTVDLTKCQGAPLIGTLPHFYLADESYLDGVIGLKPEPEKHQITFIMEPITGVPLLARKRFQFNVDMHPVQFVNLTKNLRPTLFPVLWVEEALDLGPELMGFLQARLLTNLTLVDIVKWTLIVVGCGIGIMGLIKHQMEKEQQKKHERGASVSPAPSNASQEQLIGQSAFRSDSEFSFKSNEMLMDPSQLTGMSKTTPPPIIPHPHMPTPPEVFTLERSLQERLSPEVGGIPPVEIPPSRLSIVRDMSPINESSVPGVPSEPNPVSGKSRKSMK>AlucSNMP2aMQRNGWATVDFKMGNISINRVMYLAGFGAIVFLIGLFFATSGTDMMINAKIKKSIVLEEDSEGLKRFQKMPFPLEFKVFLFNITNPDDVMMGGKPILTEMGPYTYDLYKEKPDLKFVKDGMIEYNMTYQFHFNHQKSHGSESDVVTALNVPLLGTAVMVEQTFPMGLGFLNNAIPFLWPNVTDIFMSVTVADLLFNGVLIQCNYTSGPAMPICNGLKGRAPPTIWREEDTKHFRFAMFRHKNKTTEGPYKVKTGKDDISEVGQIVEYKHRNTLKNWDKNSSCTVIRGTDTTIFGPPKNPHDNLYIFVPDVCLSFGASYVNTTVQYGIPLNKYTSDEKNMASAARDPDNLCRCAKDDDGVRQCLKDGVIDASPCQGAPVIVSNPHFLDADPEYRDGVVGLNAIEDKHKTFVLMEPRTGAPVEGRKRMQMNLRVKKVASITLLENITERVIPLLWIEEGTKLEGPLLQELQKLYHIVGFMGTFSWVLLAAGLVILVISGALYLKVRRLFCFSGTQLVAPVDSSGVGAQRMNTFGVTNQGADDYQEHGYPGATIYPQLGGSQEKNGDMPR

>ClivSNMP2-2

MRKCVNWLFLAGAGLLLVVVILIFHYVILPYIIHKQVAGTLALNNGTDAYDRFVKLPVPLYFSVYFFNLVNPEGVFEGQKPIVQEVGPWVYKETREKFNVEFDDEKDTAKYQQRTNFEFDPERSHPRTENDLVHPGNIIPQVIMRIGERISGLDFLTELVEFGLPEVMGKDYSLILNRTVGDILFNGIDMHCSANASLGAQAVCSMVRHFPGINSLKQKPNGDLSAGILSFRNGSSSDVFEIYRGNKDFSKIGIITSLNGKTSVTNWFGEECNQVAGSYDESILQPFLSEGSSFKVYGSDICTTTPLASVGETTALDVDCLRFAPGDNFLGSVTDHPENYCYCPGSIHDVTSPQACMKKGVLEFSTCQGVPIVLSFPHFYRADTLYQNAVGGLNPNAESHETYIDIEPTTGVPVKAAKRIQVNVQFKGSPALKITGKARDVLLPFMWIDESVQLEGEQLALIKDQLIKMLRIANIIKWVVIAAGLLMLLVGGLMAYVSVRAEHRHPD

>ClivSNMP2-1

MGSISGNKILYLAGFGAVLFFLGVFFGCAGIDLLINYQIKKSLVLEEGSPGLATFKKTPFPLEFKIYLFNITNPEEIMNGGKPVVAEVGPYVYDLWKEKPDLVFLPDGTLEYNLTSRLFFNQEKSGNLRTTDVFLALNIPLLGTATTVEQTFPMGLVFLNNAIPFLFPNITDIFMTATVGEILFDGVTIKCNYSSGPAMPICNGLKGRAPATMWRLDTKDYKFALFRPKNTSVEGPFKVLSGRDDISQVGRIIEYKNKNKLTAWDKNSSCATIDGTDSTIFGPQKTPHEEIQMFSPDLCLTFKTTYINSSTINKVPVDLFYAAESNMASSQKEPANECRCVKDDDGIKHCLKDGVIDASPCQGAQVIFSNPHFLDADPEYQNGVDGLKPDATKHTTFVYQEPKTGAPLEGRKRMQMSLKVKKIEGITILENITERLIPILWIEEGTRLEGPLLGELHQLYHIIGLLETLKWLLMAAGLLIVAIAIVLYLKVRGLACFSGTMLVTPVDNVPGQKMNTFGVTNQGSDDYEGGPNFGIYPKLAASQNKNGDIVSPTSHPQQIR

>ClivSNMP1-2

MPSSRPKMSKTGSKMSKTRSKLKHSSDSGISSSLPPMPPKYRQYPPMTVIPEKEKRNDSPVFTNIMERLKEVPTRIKEAPPKKFGKFGAVLAVGGAAFGWIIFPYVLYRSLTGVMNLEPGGEVYEKWKVVPFALDFNMYIFNVTNPDEVMNGAKPILQQVGPYRYEEWKTKEGIVDDPANDEMTYSNVNTFYFKKQETLPLTGDELVTIPHLPMMVSNAGTKITKYDCRVCD

>ClivSNMP1-1

MAAPLNLGITGGVLFFVGALFGFWGFNKFLNYQISQTVQLRNGNEMRDNWAKFPISLEFRIYLFNLTNPEEVFKGGKPKVQEVGPYFFEEWKSKGNFEDDSSDDTVSFNMKAVWYFKPERSEGLTGNEMVTIPHPVLFGLVDSWSQLPNILCSKDGCYDFKFTWGRKKKRKFERLRNDPNITETVILNWPFMSMVMTVEQTKPGALPMLAKALPPLLNNISTPFVTARAMDIMFDGIPINCSSKEFGPKTVCTLINADPKGLVKKSPELFLFSFFGPKNDTFEPGKFTVKRGINDPLEVGLVVKYNDKPEMSTWPTPECNKLTGTDSTIFPPFIQDSDDIVSFSPDLCRSLPAKFRHKVTFKGIPGNHYTADLGDMSANEDEKCYCPTPTTCLKKGAFDITKCAGAPIVLTLPHYYLADPSYLDEVEGLHPDEEKHQIFLNFEPITGTPLAARKRLQFNIKSHPVKKIPLMKELPTGMIPIMWIEEGVELPQDFIDLLEANLFRAIRMVGVGRWIIMILGLGLIGGGVFLYYKRKQAPTNGSVSPKTVQVETVSAKY

>AlinSNMP2b

MPSLNFVNELLELGFPELLRDNYAMRLNRTVDELLFSGITTHCPPNASLSAATVCSILRHFPGLKSLQKYPNGDMNVGIMRFKNDTLSDTYEVYRGNHDFDKIGQIVTLNGQQSVDNWYGDECNKVAGSYGETLLKPFLTEDSTMKVYGSDLCSSLPVGFKETSSYEGVDSFKFGPQKKFLGSVVDYPENYCYCPGSIDGITLGQGCMKAGAMEFSACQAVPVVLSFPHFYKASSHFQNAVGGLDPDSDKHESYIHLEPITGIPLKGVKRIQINFQMKGTPAMKITKNARDTLIPFLWVEEVAALGDDQVNLLKDMLLKMLKILSIVRWVLIAVGSLMVLVGCVMSFLSARKEHRHQY

>AlinSNMP2a

MMRNGWTSVDLRMGNIHINRVLYLGAFGAVIFIIGLFFATSGTDMMINSKIKKGIVLEEGSEGLKRFQKTPFPLEFKVFLFNITNTDDVMMGGKPVLTEMGPYTYDLYKEKPELKFLKDGMIEYNMTYQFHFNAQKSRGSESDMVTGLNVPLLGTATMVEQTFPMGLGFLNNAIPFLFPNITDIFVTTTVKDLLFDGILLRCNYTSGPAMPICNGLKGRAPPTIWREEETKNYRFAMFRHKNKTSEGPYKVKTGKGDVTEVGQIVEYQHRQTLKNWDKNSSCTIIKGTDTTIFGPLKNPHDDLYIFVPDVCLSFTANYVNTSIQNGIPLNKYFAAEKNMASYSKDPDNLCRCAKDDEGVRHCLKDGVIDASPCQGAPVIMSNPHFLDADAEYQNAVVGLKPIEEKHKTFVMLEPKTGAPVEGRKRMQMNLKVKKVNSITLLENVTERIIPLLWIEEGTRLEGPLLQELQKLYHVMGLLGTFSWVLLVAGLVIMGIAGVLYLKVRHLFCFAGTQIVAPVDSSIGGAQKMNTFGVTNQGSDDYQEHGYPGTAIYPQLGDGQGKNGDLVHTVAHPQAR

>AlinSNMP1b

MPSQKSESRNSESKMSQYPRVSQVSKPTKGSRATSPVFSNLMERMREMPTKIKEAPPRQFGKFGAAMVAGGVGFGWVAFPYILSFAISKMVNLAPGGEIHDIWKDIPQSLDFNIWIWNVTNPMEVQNGGKAVLQEVGPYRYIEWKKKVDLIDNPADDEITYSSLNTWYFQKDRSYPLTGDEIVTIPHLPLMSMLLVAEQDFPPAMMTVLNAAIPRIYGKLDSVFMQIKAKDLLFDGYPIDCTSRDLIGRTVCVAVKANSKPLVKNGRNKYLFSVLGTKNATPEDVRITVKKGTVNTYDIGKVVKVNGNPMNSVWKDECNVLDGTDATIFPPYRSADNVSIVAYATDICRSIRGTYIGEGSYNGVRGHQYAVDLGDMSSNPKDVCYCIKKCYKKGTVDLTKCQGAPLVGTLPHFYLADESYLDGVIGMKPDREKHQITFIMEPITGVPLLARKRFQFNVDMHPIRFVNVTKNIRPTLFPILWVEEALDLGPELMGFLQARLLTNLTLVDIVKWTLIVVGAGIGIMGIVKHQMEKEQRKKHERGASVSPAPSNASQERLVGQSAFRSDSEFSFKSSEMLMDPARLTGASKTTPPPLIPHPHIPTPPQVFTLERSLQERLSPEVEGIPPVEVPPSRLSVVTSVTPVEESAPAAGAQPGSKPASGKSKK

>AlinSNMP1a

MGAPLRLGVAGGALFLFGSVFGFWGFHKFLNSQIAQTVQLKKGNEMRDTWATFPVALEFKVYLFNLTNPEEVQNGGKPKVQEVGPYFFDEWKSKGNFEDDSAEDTVSFNMKAVWYFQKDRSEGLTGDEMITIPHPVVFSMIAQVERDKPGALPMLAKALPALFNNLTSPFIAARAMDILFDGLPINCSSKEFGPKAVCTLINANPKGLIKKSPELFLFSFFGPKNGTLDEGRFTVKRGINDPKEVGLMVKYNNKTKLDVWAGPECNTLSGTDSTIFPPFIDDSEDIVSFSPDLCRSLGAKFRYKITYKGVPGNHYTADLGDMSANEDEKCYCPTPTTCLKKGAMDITKCAGAPIILTLPHYYLADPSYLDEVEGLHPEEEKHQIFLNFEPITGTPLGARKRLQFNIKSHPVKKIPFMKSLPTTMIPLMWIEEGLELDQKFIDILNANLFRVMKIVGVSKWVMMLLGLGMGGFGAFLYYKRKGEAGQPSEKSPTPKTVQVESISGKF

>ClecSNMP1

MSDRRGSKVPSRFSHPGTGMTSKMSKKITDDFPQLANLMERVKQVPDQISEAPPAKFGKFGAGLILGGIGFGWVALPYIISFAVQQIVHLEEGSDIRKIWNNIPQAFDFKIWVFNITNPDEVQKGGIPVVAEIGPYYYKEWKGKVDLVDDFEEDTITYSNKNTWYFMEKESYPLTGEEIVTIPHVPLFSMLLIAEADFPAPMLTVINAAIPKIFGKLKNVFMKAKVRELLFDGIYIDCRARDVVGRTVCVALKQNSRPLVKLPNNQYLFSVFGVKNATPEDVRITVKRGVKDSKQIGSVVKLNGKSENSVWLGEECNKLTGTDSTIFPPFRGPDNMSIIAYSPEICRSLYGRYEKEGEYKGIKGHIYTVNLGDMINNPKESCYCPRTGCLKKGVTDLTKCQGAPLVGSLPHFYLGDESYQKGVVGLRPEKSEHEITFMMEPISGTPLVAKKRLQFNLPVHSVRHVNLTRKLKSTLIPIFWVEEGLELEGELMNFLEANLLTSLRLADGVKWTLIVVGSGLCIGGIVQRQRKKTKKNPRVSPSPNNSQAELVRSVSPMSLNAGLLNSLKSSTEMLLPSEHPHIPSPPMQPVDVYTFSERLYERLSPNAPPLEKSHSRLSVVKEDEKPPSRTDEKEESTANQPHGESPHM

>ClecSNMP2

MGALTVKRILYVAAFGAFFVILGGYFGWYGFEYITMRKIHKDLSLDNDTKGFTNWKNLPVLIKFSMYLFNVTNPEEIQLGGKPILQETGPYVFEIQKSRTNISWWPDGTIQYYDKSIYRYAQDTDVQNSLDEEITLLNVGLLGAAILVERIFPFGLSLVNEAIPLLFPNSTDMFIRATVREILFDGVMIQCNYTSGSAMPICNGLKAKAPPTIIRDEVTKHYSFAMLRHKNNTLSGPYRVNTGSKNVSELGKIVEVKNKMYIPFWDKNTTCSEIKGTDSTIFPPLTEPKNDIYIYVPDVCISMSASFVNKSYLDSKITAYHYESSKNNFASVEEWPENKCKCSKLEDSHKEPFCYKRGAFEAFDCQGSPIVFSPPHFLDADPSYLNYVEGLKPDREKHLTYVSIEPLTGVPISGRKRIQMNMFLKKIKGITLLTNVTEGLFPIGWAEEGADLHSGGITELAKLYYLLETFKVIRWILIILGVILMVIALLLYLKMKHLLCFHNNQVTAEFEYGVNQWRAKEGEPQKLSTFNTDKVSGVEDAPQTISTIYPPLGNKGDVPTSITSTTVPR

**ORs**

>GrubOrco

MQKIKMHGLVGDLWPNIRLMQLTGHWLLEYHEETGGMVRLIRLGYCWLTTFLVVMQFAFLACFLILDTYDADQMAAATITTLFFLHSVTKFGYFAVRSKYFYRTFGAWNQVNSHPLFAESNARHRATALSRMRKLLMIIGIITILSVMAWTTVTFLGDPHREITDPEDVNATITVEMPQLMVDAWYPWDARSGFCYFATFVYQLYWLFISLSHANLLDILFCSFVIFACEQLKHLKEILQPLMELSATLDSVVPNSGDLFRAGSGGSNMPLVENDGNDFDIRGIYSNRGDFSGFGQTGVSTIHTNGNGIGPNGLTKKQELLVRSAIKYWVERHKHVVKFVSSIGDAYGSALLLHMLTSTVTLTLLAYQATKIEGVDVYASTVIGYLLYTLGQVFVFCIHGNELIEESSSVMEAAYSCHWYDGSEEAKTFVQIVCQQCQKSLTVSGAKFFTVSLDLFASVLGAVVTYFMVLVQLK*

>GrubOR2

MLKIFIRYESIFFYTSPKMGTSPPKMHVLRSDRDIEIDEEIDRMYHKLTTISAVHPVFDKKRPLLCVMNLLIFILHSLSLNVMNICLVLTAIELYNINLVPFFHGMHITFMGFLSNASLWHSWLKRSTVSRLHRLILQDFFDYNEELEDKKKKLWDEMMKEKKWHIFYIITIGVAVSIVLILIPNVNHQFGTFQYNSTVHNVNFDLPVPLGYPFLGNEPLKLLTGSLLSFLSGFSFTLTNCAKSLMIINCNLHLQMQLKFLLYQIENIEYRAGRLHMQLYGVQPKNTGLKLYDAEFMKCYDICLRRSVQHHQIIVRAAEEFNEVFSFFVFFFYLTGAADIAMVLLSTSATEEFPGNTIGAVVMCAVEVGFVFLFAHMGQRITDLSVELREVIYNMPWYRCDQGIKRTMRILQIATLKPLSFSYYDLLHINYDSFATVINSAYSYYNLVNALRD*

>GrubOR3

MVFGSSVGKWLDDKLIELKDYINDEIDEKMYAFIDKEYFHLMQISCLYVKLDRRNRLISFVQIIFYLSVLFLHYFVLSSSTVLMIDINMVLFCQNIHFTLLVQLTIILLVFFQNKHRQMTLFHRLLSCNFFDYQEPTVDGEDALRSSMVRERRMLSVIPIAVALAAGAVLIIAPIVDKQAGLFDFENLSRVFSTHLPYPYAKYTFNNREGFNYYFALGGQVVLGILLTGIIGSGGFLFLNMSQNLSLQLQLLQNSLDKIETRAEVMYLKLYGKPKGDSTAMYDDHHFIYCYNVCLNKNFKHHQIILRAFQYTAEIISIPVFLAYMTGTIVIALSLISAGSADELPGTTLASMVLCGVEVGYMFLFSVFGQRIGDLNMELRFKIYNIKWYLCSRRIKMNLMVFQERTMKPLTMMAGGIVPANMETFATVSCRKLTDTRMQ*

>GrubOR4

SGKLTLKLQSFVDHLNQGFDESINDFMDSTYIRLTTLSGVFPTLKRDRPFLSLLKLSIMISICSSIMILWCIGTKGALYFSGSNFVLLMNQFHFMALGLMSLEVVRHSWMNRPEEGALHKLMLNNFFDYEEELSGTWTSLKEAMKKQRRRYIQSVILIGVAVSSTITLIPVLNSLNSESHDKTLPGIHMDVPIPVPYLINDNKSLSHFLAFLLEFVAATGVGLVLSTKCMMCIECNLYIQMHLKLLLFQFQNIETRATTLYRQLFGTLPNLTGVKLYRDIKFSNCIDECLKKNVKHHQIIIKAVRKLNTVYGCYVFNYYLLSTAAIALSILSTSAKDEFPGTAFGAVIICIIEVFFVALYSHFGQTLTDLGEDLRGEIYNVKWYYLNKKTRNTIRIIQIMASKGLYFSFHNILQINYDSFSKVLNSAYSYYNLVKANEK*

>GrubOR5

MAGIFSRRFGDVIDFLNTGCDEKTDLFIEKTFKNLTTISAIYPILDKQKPLLWSRNLAVFLVHSTVLIYAFFLTSKTSTILFYINFVPFVHEVHIAFLALLSIAILWHSWLKRPNVAKLYRFIERDLFDYEEELEEQMILLRKEKIKERKRYTLCVIMIGFAASVAVILIPSVNKLGTFVYNATLYDVNFELAVPLTHPFTSMEPIPFFFENAYIMLTSTIIALVNCTKSLMAIESNLSLQMHLKLLLHQIENIEVRSVRLYRQVYGVKPKLGGFGSYSKNFLKCYSICLRKTIQHHHVIIEAVREFNEVFGFYVFFYYLTGTIDIAMSMLATSSTDEFPGTTIGAMIICLVEVCFVALFAHMGQRLTDLSLDLREMIYNTPWYRCDQGIRSNIKILQIATLKPISFSFYSLLNINYESFATVMHSAYSYYNLVNAYK*

>GrubOR6

MLPTGKVDYLVKKSNIEFDESVEKFIDKTYHFLAQFSCHYLKMGKRYRFWSTLQFLVYYSVMGFHFAVLIHTSFILLSINFIIFVHNSHIAFLTFLVATVVLDFQRHRSAFVRLHRMIATDFYDYHEPELEVFSKHREDILRQRRRLAIVPVSACIAGGATLILANVLDRLGTFDFNKTDGLVSESLPYGFGVYPYNAKDGLGYYISYLLQFMLAIVLASGVGMTGWIYIILTQNIMLQLKILTSSLESIEERSKNMCERLFPGRLSDSSQKNFYIEEFIYCYNKCLNKNFEHHQVIISAFSCLKELFSVPVFLSYMIGTIVIALSLLSTGSGIELPGTTTASVLLCFAEVGFMYLFSVFSQYITDLSLGLRFTIYSTKWYYCNKSIAQSMAIFQTMTLKPMTLTAGGIVPANMDTFGNVMNSAYSYYNLVNAFNITV*

>GrubOR7

MWLFESTEEAEERVQVETFIDQQYYRLMQVSSLYLKMGPRDRLVSLIQLSIYVTILASHWLLFVNATFAIYDINMVLFSQSVHFSLLIQLSLLLPICFRIKHQKMYQFHKMLSYNFFDYHDPPIIGEESLRSAMADENRRMFLIPVGVAAATGSVLIIAPIVDHNAGVFDFNRTANIFSDNLPYRYLIYSFPIKDGFGHYFALIWQMLCGILITLIIGGGGFLFLNVAQNVCLQLKILISSLDHIEERTEHLVIKLFGNKYKSKNSSYRDKNYASCYSICLRKNFEHHQIIVSAFRILEELASVPVFLAYMTGTIVIALSLISAGSAKELPGTTVASMILCGVEVGYMFLFSVFGQRLSDLSCELRFKIYNIKWYLCDKGLKANMKLFQEMTLKPLTMTVGGIVPANMETFSTVMNSAYSYYNLVNAFDG*

>GrubOR8

MGASSSWINVLSSDNDIETDEEINKMYHRLPFLSAIHPVMDRNRPMLCLLNFLLFILISSSLSLMASFMIKASLEIYTINFVPFIHEVHITFIGFLSMASLWHSWLKRPNVVLLHRIIAQDFFDYKEELEDKMKKLRHEMMKERKLHIYVIISIGIGVGVVFFLIPAVNKFANFNYNSTVHRVNFNLPIPLGYPFLGNELLELLPGYAICAASSIGVVLTNSTKTFMMINCFLHIQMQLKLLLHQIENIEDRAGRLYMQRYGVKPRNTGLKPYGTKFMKCFDICLRRSVIHHQIIVRATGEFNEIFSFYVFFYYLTGTLDIALCLLTTSSWKEFPGHTIGAPMICIVEVGFVFIIARMGQIITDLSEELREVIYGMPWNRCDQGIKKTIRILQIATLKPLGFSFYDLLHFNYDSFASVMNSAYSFYNLVNAKNQDQ

>GrubOR9

MTLMEKIEDFFRSIDTSDEATDRFIDEQYKGLLQICLLYAKFDKKSLPMSCIQFFFYGTIILYHWIFTIKGLIDIKGINFVLYSKCAYYYMFIQMTGIGMFTLTMNRHALVRFHRSMSKNFYDYQENGTEFIERLKEEMNKEIKLFSLVPISTVIGALTAIVISPLLDKYGTYIVEDVSLPLNLNLPAPMVAYQFSTRTNIGFALAYMIQICLGNMVLFIIGGGGYLYIVMVQNVRYQLKILIHSIEKLEQRADDYYYQLSGFKIKYKGQKKFADKLYDFCYNECLKKNFEHHQIILSSFDCVRRFFSIPVFMAYLTGSVVIALSLLSNNIWELPGTSLTSSVLGCIEVGYMAFISFLGQTIIDLNDELRVKMYESRWYYGSNRVKNSIQIFQQMTLKPLEIMSASIVPINLDTFAVVMNSAYSYYNLINAFSSK*

>GrubOR10

MGIAGRMTALLQDWNQMSDEYSDIMIQKEYRGFLHLFGLYPNVNKVGPFWSIFRALLVGFLLLFHSVGLMVSAVLLYDINYVYFSSALHYVLIVGIAASYVAHFNTNRQFIATFHRKMYFNFYDYHEGTRVEIKDLIDTEEKRKKRFTILPMFAALAAVCILLLAPILDKYGTFNFDIDTPINFNLPLPMVYVFDSSSGGGYLVALTHQLSSAMLCGLVLGTTGYTFIVMATSVSVQMRILIHMIEHIESRAKALFKRKYGELPILSLQELYRDKRFSDCMDICLRKCVEHHNIILRNFSEMNSFYTLPLFTIFIIAAFIIALAMVGSSNWKVLPGNTVASLVLCGAECSFLGTLCSLGQSITDLNEDLRTAIYGIKWYHCNKKFCMNIRILLIKTLKPVIYNPCGLIKISHATYANIINSAYSYYNLASATSS*

>GrubOR11

MWQYHEDYETNRPIDAYISKHYKSVLRISGLYLKMDRRNRFLSIFQVVIYVLTLLYQIAIFINSTIIIYGFNMTLFSLNAHLALLMQVSLIVLLSVFWNNFKVFIIHKLLSDDVYDYQEPPVAGEELLRSKMKAELSKLILIPIGVALAAGVILSVSPIIDFKAGTFDFNSTAWMFDYHLPYPYSKCPYNVTDGSGYYFAMLEQVACGFVLSAIIGGGGFLFINTSENLCLQLMILGNSLDNIESRIEHLHAKLFGTMDGDSMESIQHDKRYAYCYNQCLRKNFEHHQVILKVFKLLEDIFSIPVGCAYLTGTLVIALSMVSAGSAKELPGTTAASILMCAVEVGYMFLFSVMGQRINDLSAELRYKIYNTKWYICSKEVKSTLMIFQEMTLKPMTMTVGKIVPANMETFTTVMNAAYSYYNLVYAVDKK*

>GrubOR12

MEGGAKHQVVDREVVYDFDKEREIQMIISCFKISNSSKLKRFLCLLYVTSYWWFLFYETCMGFYSLYLTMDDKAQMLETLHLTLLTIYAMVHFSNKLLSDFDGVLEIINKGFYKYDEKPDERHYEIRRKHVKQIRVVNKWFRMIIISSGLSFLLFNTAKKYIESLYKNKPSDIPINPYFPIPFYVPFDTTSVGAFAIVYLTNVIIMNSICMVTIGIEEIFISLMEQLKAQFEILNLSISNIEERALQRYLNRNVGPKPKVESLYELKEFQDCLLQCLKENIRHHQVLLRFTELIRNYVQRTFFHFVGTSCLMLASAVLFITKSKLSGEELLGVGTFMMMLLTEIMFDLQFCLYGEDLMNESGRVFFTLWSTPWWHFDKRVKSVLSIMMLNTKKPIILKSPFLNISTSLESFSGIISSAYHVGNVMLNL*

>GrubOR13

MTFEKEGLDKEFFYDFRETRFWYKTFAGSSMSQQMKPSLFLETYRIFAFLFLIMMTFMFFITALLTTKYLADFSEAFHYCCILLTMAIYIPAERLAKNDIDKTFQKLKKCFYVYNGELNESQKDIKRKTIAKIKFTDKVFFWVLIFVCVLYSILTPLKDYFYPHLRLERSEIIDRKLPIPFYVPFEDRYNIIFIIVFLMECCCNMMVHSLITSIHEAYISLTGQIYGEMKLLNYSLSHIEERAIKLYLSKDLKRVKSIRSIYKLTAFKKCFEECLKEDLIHHKVLLSCIKLVRPFTGRIIISVFTLCSIVWAVGIYVISQMIEQGTAMDKVLEFLLMLFTEGVYVFDMCLFNEKIKSEMDELFIQIYSIPWYNCDRKFCQNVHLMLMNTMKPTQLNKNLFNVSASFETLMPIISAAFSYFNVLRNIRN*

>GrubOR14

MDSIELPAEDIPIDRQITHDYETDRKNLMYMSGFKVNYDSKLKYILSKIYVYTIMFFYTFLVSMGSVAAILIHDKKLKLEAVHWTFLNLVGLTFMIIRYTYALDEPLKMIKIGFFTYENEAVDERYFERKKYHVRRIRNVSNMFLGLAISNGILLSFIKQVKKLPEAGDYGKSINTGLPIPIFLPFDTTTTIGFVLGYLIDVVAFFYLVVITSSAQQIFLSFMEQGIAQFEILNISISNLEKRAYFMYSKGKIYTEGITMDELYRSRKFQECVFQCLRQNVRHHQTLLRFRNNIKTYVEAVFVLLILASIVVFAAIMFVILELQDLSEASTLFWLFSTEIVYVFVVCIYGELFSQESAKMFYVLWDIPWWKFDKHNRLILLIMMSNSLNPVVINAPFFSSNLSLESFGDIMARAYQFMSVVRNVDRK*

>GrubOR15

MHLTDKDYLLKGYKGSQCFLLRIGAMYVLQDENEIYNWIFVGIFHMHYYVFLFWILPYDVGHTIPMGHMTATLQALHYYCTFLGYSIVNYLYHFNRKSVNYCLTLIGKDFFKYEEGETELEMKIAQENDKKQKEYIKKLTRYVMVIVFATAFAVLMLPTFAELLTRTEPIPDDEVMNPYLPIPLVLPWDSFTVGGYILIYVLLFLVSYNITTELLALSMGYTSFLIIYTSQFKILNKSVLKIEERATNRYRKVAVKQLKGMEKFDDPIFQECMNSCLKQNIKHHQILLKWMDEACHFLGWGVLCTIFTTSFLLAASGFLITMESDSSLLKSLILVQVQILELLHAYLFCWWGENLATESAKLYHSLYKTPWFYCGKRFNRLVQIMMSNASKPVIPKDPLFKINASLEVYMSILSTGYSYFNLLRSMN*

>GrubOR16

MDEPEDFDFINSTYWKMFSYSHLNVFLDKHPTGDVSFWKLKRYITYFFLYLYFPVWLVIEVSGMFLGRQGDLAQVAFDLSYVAHIVQLVIKMGYFLYHIKDIRSICLRFERFHTSKHRPIFSRRLLGERSQFLRRLASIYYTVIYMNFLFWIVPPLFIQPIIYGLTQTGFIVATGPQNIIPKFFPVRYPFDETSTRNQIIIACMEFTVLSAGFIYFIPIDQFFVSVIIMICSEINVICKSLMASGELRRELDRDYSVLLGSGDIKERINLKLFVEDHQRALRTTKKISEVMNPILGLVVGNCMVLLCTLALVITRKMKTASSFSEVFREVFGFIIVMTTSLITLYLYSWMCGELKSSEEAVFRAVYSSDWYNRNKKYRDNVLIVMRQSYASRPLRMMSMGDMDKETFIKGLKGIYTYYNFLTHFE*

>GrubOR17

MLGKIKAALKGKAEKAIGKTLYENQGIHMVLSGVYPVNVFYTLTAWVCQLGTLSCFIYTCIYFYYYEYNLTMFSEAAHFVVVVFCSASFYVIFWFRKDKIDNITKLLEQEIFKYKWTVDPETKREIEDMTQRMKDRKNKISNVMTITYILCGVCGVILLPIVFRKSGISVKEERILLTVPIATYFPVRGDLGWYLCSFNQYLLTFFIVSALNGSDVAFSCYCEEGCNQLWILGHTIRSSLKRARHLHKKILGKEPTSLRDETFSVCLKMCLNESVMHHYTVIRYFKELQSLFSWCVMIVMMAGATMLCLSGIYFLFDIVGFGTKCSFIFYLSGELMHTFIYAWYGQQISEMSAEIREVLYEIDWEECSKTVKPYILIMQAYTNNPIKLKGGDFMEYNLNTFGSVCSSAYSYFNLMSAAVPSSS*

>GrubOR18

MDLSFSELIRSEVLWRCQKASGMGMIWLENIKESLRRTRIFLSLSGMVVVFNRRLAYASCIIAYNLLALSLLIYSIYYYLDQIEKVSNIVHHSILILDACVGSAVGYFYHNQFDAILSDACSSYNYESQMMFKKMEEVKGVICMNYSAMFKYMLMSMIYLLLLMNTLAISQRYLNGEDILLLFPCYFPFSLDYYPIHIAVIIWQELIVLNIGVLVFSSLAVLYCVYSHVKSEIDILKFAMNNIEQRAYEMATNQKYYHDQNIHPEILSRCYVKCTRMCAEHHSDIIRYFNNGEIVIEIIYFLVFLTGLLVCTCTGFALISENTSLKIKFVGITLIQIIYLYIISWIAEDIAGQSQSVGDLVYVMEWYRLPKECQTILIIMRIRAGKPLLMRMLTGQKVDMAAFMALIKASYSYFNMMLATTQ*

>GrubOR19

MISGKKIKDFFYAKKSNDDMGFINNEARFLLTGISIWKTHSRFVIPSATVGFFGVLMLLSNLLYTILHYDSLNWINKIVDYGWTLVYLTVTYIIINNKEEIISLSEEVDRWWKYTYLEEEKVQLKIKSQNWMRTFNKYYVTCSTISWSFYVLPPVAKYFYKDRKQTMDALIFAAWTPFPLDIAWGYAITYGLEVLIMAFAQCLYSQLFLLLMTFAIVIGHQMRLIGTAFLTIVKRIDPMMKDITFETRREFYEVRDILLAKELRNGVRHFQHLYKCSYRLSRIFSSITNVTYHGGMWVMCSIAVKAATEMTFVVMLQAFMMISIVIISQYMYSFINECIIEEVQKLRNVVYDSPWYEMSTKIRKEFHIFQTMLDSDRFPTLRTIMGSQTNMENLSKVINASYCYFSMLITMKSRYETTTLE*

>GrubOR20

MDFCSAPRSFLRKYVAAVIKDEPRGYVFTSCVTIRYGIAVMEMRKNEYLVVRYPLLMLFSITTILWILIDHILEFRDARWFTKILDYNYCGGSLLFAYTLKVKRAEITVFTQVLDQWWPYTFLDREQEELKDNLQRKLKKFNHFYNRFMVTMGIGFGLMPLGRYFSETKKSSTNLLLQRCWSPFPLDTWWGFSTTYWVELTSMLSLFYCWTYIVSFLITVMETIGKQLQLIGISLVTVEERFLKTMLLFKMTDKKERYELYAGMLQKELANSVKHYQKMQRMSEEACSVFSMPINICFSSGILAMVVSCAKLATERDNKFVIFQAVVMVVNVLVNQFIVSCINQILTDQMDNLRITIYDTPWYKFPIPCRKTIHMMQLMLLNPPAIRTVFGTKTDMEFFATVVNTSYFYLGALLSMNVKL*

>GrubOR21

MGFKTELNSFREKYIFKVIEEDSKDFVYKKSVIIRHGITIWNKGKSYWVIRYPINLAICFIIFVWVFVHHVINFYRYSNVTKMFDYAYPGSAILYATTIYSQQEQIGKVSKILDQWYGYPFLEKTMKDLKQNAENKVEKFYDIYYNFMKLGSVAYCLMPIWRYYQNVEKDASYLLLFPCWSPFPLTSWWGFSITYLLELWTVINVFFTFGYIITYTTAVAITVGYQCRLISISLITIDERVSKKMEFQNIESGKDWTEARVHKMKKELRNSIIHYQRIYGLTKEVCGFFTGPISICYYTGIFAVVLCCGRLATERENMFILFQATFMASAVLVNQYIIAFINQVISDEYEKLRLAVYDSPWYEMRITCRKMIHILQSMVLSKPNIKTFLGSKADMEFFSTVINASYCYLSALLSMNIKY*

>GrubOR22

MGYQAVIKDSDVIEGLSIRFLKFFGMWNAVNEYRNTGKRSLIIKIYIFGSFLLTVPAAIFQTQSIFTIEFDLQKATFMYMHTLPAISLCCRIIVFWFRMDGQCKLYDLMRSDFFVIPERLREKVREVYKETNKISNKCCLVPFVWNIGIQIWFIAFPGVSVEYIQHRTGSMSAVTTGKKKILSGWYPFPIEEYPYYQIVYVYECLCLLWACNVMAMYYAFFYQILMCLHAQFVVLAFRLTNLKVDSTNGDVRRYRIPGHNGNSKINEEMKEIFKDHQKLLRYTEELRSIYNPLVTMTLGIGILVLIIGAAQILLGKTSDPSFLFQLFQIFSFQFIEMALFCFGSSLIETASSDLHYAVYFSEWYRADVEFGKAVQMLMIRAKKRINLTAITIYPVNMETLGSICQFTYSTAALMAGMVE*

>GrubOR23

MFGQEKATSFLKKYFTIEKNREASLASGALRRFIHGLAIYDNYVIPLPFILVVNSIMLSLLLTENMMKIANINWFDKFVEYNLCYPAVLYSILVTKNRRDLIYISESIDVWWNYQFLEKKTEMVKKRADAWLKRFTNAYRTFMIMGYLCVFFQPIGKYIWNGGSDMKDLMLFKCWSPVPLDKTWGLMMILVLEIVAQFFPFFMYSAIACYMITMTILFYHQTHLICEAFATIEERVWKMVWQTIETPNGAYLIYTEILQQEITNCARNYQNLYKYLKKMCKIFNITTNILYYTGMFLLVSSGLHLVTKKTNSLMLFQAVMLISSILVNQYLNSFITETLQEATARLRISVYSCNWYNMPKKCKINIHLMQTLVSYSPKLTTILGARCDKEFLSKVINASYCYFNALYSMTVERNENLH*

>GrubOR24

MSDPIRDSDIFDGQTIFYMKFVGIWKIVNTYRTSGKMSLVFRLEWYLTLLLSVPFQVLQVISPYYIEVDLEKATILILNTVSFLHMVAKHGTFWWNIKGHAELFRLMTKDVLSSIPQYKRTEAKKIYKDATKRCNFYCKMIITVTYSVWSMWTFNPSVKSDYILFHTGNMKDVTTGPKKILGGWYPVPFSESPWTEIVYIYEAVLLLWCAVIVSIFDTVVTQEVIGLYAHMSVLNFHISTLTKEEVIFHSKKEIYSEQEAEDLMLKEFIAIIRDHQYLLRCGKILKDSYNTYITALLLAGGNLMIITVFQFFYGKKDIPTTINFVFYLSYGVMEISLLCWTTTLLETASTNIAFSIYSSDWYTYNTKLRNIGRMMMLRSEKPLSLAGFKVYHVNLETLMNIMQFTYSSSALMSRMVE*

>GrubOR25

MELTTGDDIKEDEYYIRKCMKDNYGLEFWLKAGGLFAIGPKARFIYLISGLYMAVWFILFPLLLMTLYQETGGRNVEIVCEAVHFTMFTILEVVFLASAIANNKVLAKTFVLMGKGFYDYQNTLDKECLEFIAQEKEEATKRKALIAKLFIGVVMCACVSITIIRPIMKFLLGEHLLGSPDDGLLRILPVTMWTPFSKDKWYVILIVWLSEYVIAYVTPGIVFGNTLYIVYTCQDVGTQLNILGYTLKSVVRRAKALNMPGEQALRLCFTHSINHHLKILTCVKSLENLLYFPGLGLLGGSTILMCMSGFIFVSDEFSFSSKIVFCLFLLSELFLIFLICWCGEHVQSTSTRVFDMVYSSEWPDNMSSMNYYLLIIQAYTMRPIKINFGGVMGASLETYSNIVSSAFSYFNILLAMN*

>GrubOR26

PRRALDKSNTLEGLATFHLKLYGFWHFINVTRTTGKISWLFVLSCASILLVVTTYVIFQTIYLFRINFDLKKMAFIYLNAAPCIQDFNKVIFFLAKMQEFCHIYDTLQEDFLKSIPKHKIPAAKEIYRNTARTSNLVCTYAYSSFFLTGIIWLIVPGYDTDDPSSGRKKVLNGWYPFRFSESPNYELVFTYEFLIIAWGGVWYCIFENAIFIPMICLAGHFDVLSYHIATLSKEDMIHVLGRNSTSRVDINAFLNAQLKHIINDYNKLIRDLVWKGEEERYSETMNKTYNLVITFVLGLEIGNLTMIALHLIFEEKDAMFIVKTGTFFFAQLIEVILICYSSNLMGEASYGIREALYCNEWYTADRKFATSQQLMMIRASVPLTLTAVKMYPVNLETLLSIFQFIYSTAALLSKMK*

>GrubOR27

MSDHTPITDSEIIDGLSIRYLKFFGLWKVINDYRTTGRKNAIIKIQLAITLMYTVPYISCQLLSFFVINMDLQKVTFITLHTLGDIQICCKVLVIWFRLDSQCRLANLMKKDFLQLPEHKKPAARKIFKKIAWQSNLLCIAALTINTSYYIVSITYPDTSVDYILYHTGNMFEVNTGRMKIMGGWYPLPIDRSPYYEMIYVFEASLFVWVGTFLAVYVSLFYQTLMCLYAQFSVLGVKLSTLSSEDEGENNEKYRKSDSEMYDELYAIIKYHQKLLSYADEMRSIYNPLVTMILGTGVFVLILAYFQFLFGTTATTIFIVKSLIFLPYQAVEVCMFCFASSYLETASSDIQFAIYSSDWYKANIKFRKAAQKMMLRAKKGETLKAVGMYPINVETMVSIFQLIYGVSAMMMERIK*

>GrubOR28

MFSWLSRKNPTQHENHDKNTEKCYIKKAYEENYGFWMVHGGFYPVLGIETCLYIPAAVLLTITFSTTPFCISKSEIAIKSETVHFAVFLAIELTAMIVFMYHRKTIDEIYLAMGQRFYDYENTLDEECYEVIASAYRAGRSRKKIFHDLFVGCSMSTLLTAVIVRPLISYFKGDPDPNDGILRLLPVPTWTPFKTKSWYVTLIFFLAEDIVAYVTPGIVFGCILFIVCASEDVGAQLIILGHTLKSVVRRAEGLNLPKDEALKLCFNHSIKHHQMLLKFIKSLEMILNIPGFVLLFGSTILMCMSGFIFVSKEVPFISKIEFFLFLLSEMTIIFLICWCGEFIQKTSNQICDMVYFSEWPDNMESMKSYILIIQLRSIKSIKLNLGGFMVASLETFGHICSSAFSYFNLMLAVN*

>GrubOR29

MSYLPEIEESNVIEGLDIRFLIKSGMMRFINDNRTTGKRNYTIKIHLIGTIFITLPYIVCQALSLFEVQYDIKKGTFVILHPTAALQIYCRILVLWFRTESQSRLYNMIQKDFLNIPKELSAGISQLYRKENRTSNVCCMATFIWNASIELLYIFFPGVSVYYIQNRSKSKGAVATGRNKILSGWYPVPMSEYPYYEIIYLYETLGLLWSSTLLGLYFCMYFQLLMCLCTQYVALGYRISKLKIDLVKYKRDQKYKSSIYQELCQIVKDHQKLLRYTDELRSVYDPLVTMTIGLGITVLIIGAIQFLLGKTNDPGIIFKLIQMFSFRTFFEVSMFCFSSSRIEEASSALQDAVYSSDWYKADLKFRMAAQMIMIRARKRVNLTAFGMYPVNLATLGSIVQFTYSCSALMSRMAE*

>GrubOR30

MGKQQAITDEDVIDGLSIRYLKLTGLWSFLNEYRTTGNKNAIMKLKIFFTIFISSPYIVFQYLSYFMIKVDLEKATFLNLHAWPGVQVTFKIMVFWFRIDNISRLCDLMRKDFLTLPEHKRDEAKRIYIKITRFFNILVKTSFILGFSTVIPYISQPSVSVDYLLYHTGNMADVKGGRYKILHGWYPLPIDKSPYYEAVFVYEALLLTWNDIIFSVLDSLYYQILMCLYAQFLVLGHQLSTLKIPDSQNPKIRFNENNSSIYQELCQIIKDHQKLLSYTNEIRRIYNPLLTIVLGMGICVFIIGVFQFLFGKTTPIFTFLNFMFLSYEATEIALFCFGSSLIEKASSDLQFAIYSSDWYIADKKFRKAAQMLMTRSQKAETLTALKMYPINVETMMSIMQFTYSVATLMPSMVQ*

>GrubOR31

MGRQQAIKDEDVIDGLSIRYLKLTGVWSYLNEYRTTGKRNWIMDFKIFFTLLISSPYFFCQYVSYFVIDVDLQKATFLNLHAWPGAQVCFKVLVFWFRIENISRLSNLMSKDFLTLPKHQVGGAKRTYMKITRIFNMFVNTSFIVCLISITSYLSQPSVSVDYILYHTGNMADVKGGRYKILHGWYPLPMNKSPYFEAIFVYEAFLICWNAIAFPLFDSLYYQLLMCLYAQFSVLGLQLSTLKIHDNKNPKTNDSNSPIYNELYQIIKDHQKLLSYANEIRRIYNPLLTIILGMGICVFIIAVFQIKFGKTTPIFTFLSFLFLGYEAIEVTMFCFASSFIEQASSELQFAIYSSDWYKADINFRKAAQMMMVRSTKKETLTAIRMYPVNVQTMMSIFQFTYSTATLMSGMTE*

>GrubOR32

MDKLDDLIVDERKTRKLLRLVCCLKHIQGENSISNLNLLYIISVYILLSIVVLQGIVLAYSTDDIVEKVESIHYVLVTIIIISYMSNELYNNSKLDKAWKLINLAYNKFSENREEDRQIHIEINVESTKTNKLFCILMSVSCMGYLLFAPLRQLYTEEELIARKLPLPLYIPFDTNNNLGFYLGMAWEAISLFYICGVSTSIHQSFRGLMGRLRGELKILNNSIKRIDDRASKKYQGKVDEIEKLEPHFQSLVYNCLVEDIVHHQMLLEYYSLTKIYLGTILLLFIFLSSIILGAVGFLITKPNSHTEDIIKFLAIVSAELFFVYQLCWEGEKVAIESGQIFNNLFNIPWIRYDRNVKGCIIIMLCRTIKPIRLKTSIFNVEASLETYNWVITTAFQYFNLLRNIKNG*

>GrubOR33

MWLLDLFSTGPPRDDKGMCYEQSRMFRHGIAVSKKRFRFALPLPLAMLMVFSVLSGIVVDTIINIDTIDWATKVGDFCLRCNGLGHCIAAYLWTDEIDRLSDAVDVWVSYRLLEEEGEKLKKESKHWMDSFNYYFSNFMNFGQLSLILLPFTKLLGGADFSDLYIFKMWSPLPTEKWWATLIVYFYQICTNMINFVIYEGLTAYVMSVSVTIGHQVRLIGLSYTTIQERAMELASMRQEKKTDPARWHEIYCTELNRELSFSAKNYQHLYRNAQEMCRIFSGLVDLVFYSGMSMVIMFGLKIATEKENKIIVISTTLFVCVVVSNQYLFTIINDSIADEVTALQFAIYNSPWHQLPPRCLKTIHIMQTMVRKMPTLSNIMGHHVDREMFLKVINATYCYISTLLSMDL*

>GrubOR34

DLEAGACLSGFDNLMSLFSGMRGPLDQPAQGKWYRHLYTAYVTFINASIFFNFLTIAPVFLSPGVSIEVRCFTGFPTVAMFLALFNRTDMYLKREKCKDLTAHYLSLYGDSSELNAEVKRYGKIIKNISRILFLLTALPMVTIDLIPAFVALRGGPRALGVPAMYPFDTTEFILVFCFLCFFQSAAACQSPLSVVVFNNTFNMFAFRQLALTRHLSRELLRILSIARVDRKGVVTFQTQDGAQISKEEASQKILTDLRQWVKNHQQSIRMVNDLQDIYSVALFVEFVFAGSVLCMCAFVIANVVGGVVKVFFCGLYVFGLMMELLITCLLGNLILHESNSLESMIEGTHIYTLNSNVYKKWLRIILTKAKVPTRLVAVGVFPLDVETFKSLMMTTYSFFTLLKTLK*

>GrubOR35

MVHQERLKKSDLFHGLSVIHLQFFGLWDGINDFRSTGKTSRIFKINIALSALFIFPFIIFQCLCIFLISVDLKMASFVYMIGAAAAQVFIKIVVFWYRFKDQCALVDLLKVNFLSSIPDCKKNHINKIYQQSSFWCNIFTVLAFTGNIITIITWTSLPGFDTGNGRKKILSGWYPVPYSQSPWYEIVFWYETILIFWHGCLVSLYESFLLMLLIVLQSHFVALNYHLSTLKKNNKNIKVSTAESDAESDEVLNMELREIMMDYDKLLRYSNLLSSTYNPIVTITIGLDIGVLVFTILIIIFASSDALSLIKMLMYFSFALIEITLLCVSSSIVGSASTAIQESAYSSDWYLADKKFATTVQMIMIRAMRPVSLTALKMYPVNMETLIAIFRFIYSAVAVLSKLKE*

>GrubOR36

MSLQFETTHSNLIYLLRRCGLRLPWVKYQSRWSHFCYSTYDIFLFTIGLYQFVCSACSIILVPTFQDMCTLGIVTSVLATGGSITLFYFFYQNRLEKFTDNWNLLNEKILTSSSDSKEFFRQLFLQVAKSNESFGKTILFFVFWTPIIYCSPVPIIDAIKNSYRTNLPLPILYPYDDRQAGVYEITFLLHMMGLVISVMKKFGNDCFFLSLFKIHNAYLRYISVLIKKEGEKFKKNNNRIFKQKLISWIKIHQQIVKNSQDLIKLYTPIIIIYYVNLICIVVFGLFTQIKNDRDSSVQRFGTAMFCTVNIFQLYMQCSSAEQLSTEAEKVAEEVYNTPWNEVDECNAEIIRLVLKIANRPVEVTAFKAPTFLLNKQTFITFVVNTIRAFMTFSKMSDLRDQASLL*

>GrubOR37

MNTKKHIKESFNKIGFVEETSQLFLHGLRRYKSKSPISIPISLAVAVGNVMVCSMITGAILNYTKTDWVQLFINLILIPVSVIVGTMILRYQKSFSKVSKEIDCWWNYTFLEEKTDEMKKNAVEWMAWFGRYYYIGMYFSLSMNLVPFIKCHIWGKSKDPMNNLMYPCWTPLDLGTWWGFGMTYMFQFTSMYLSYFTFGNTCFYLTTAYVSIGYQAQLIGMALLSVEQRCEKLSEAYKDISKRKIMYETYVKQEIVSCIKHYQQLQRSAKELSKIYSTMAILAYNIGIIVLSISGIRLTTENDKLMLVNSFMNAFVTLGNQFIISFICELLTEEVEKLRNIIYYSSWVSMPVSCQKYIYTFLMMVDYMPTPTTLTGMKTNYENFSKVVNTSYCYFSLMLSMRIK*

>GrubOR38

MKVVDVLGQRRVYRWFLNFLAVACFFIDREWIWKKYYVFMSIFSAFGCGVMLYSGTLYINDIDSLSVIMHHGVVAIDLIVGLQVLCIRRNHVSDLVKDCSKSFDYESQIVSKFLNDLQDKREKSMEKVFKYNVFTSFFMIINVMIFGMVEKYLRNLEFMLLFPCWFPFELSYLPYYLMAYFWQCMLVASLCLTICGAMAIAYIVYSHLTSQITLLKFAIDKLKARAYECAYSTTKEDIKVSFEDRLRQSFIKGTKQCVEHYSMIIDYYTKAKNIFKIMYFMVFLTGIILLTCTGFALISENTSLKIKFIGVNTVQMIYLYIFCWLAEKISDLGESIRLSIYGIEWYEMPRECQSSLILMVERTLRPLNFCTLTGQKVDLENYMDLVKASYSYFNMMLATQQK*

>GrubOR39

MDPPRFFDHYRVLITCIRRSGLPTPWIEKPHTIAKVLYLIYDSIIVIMLLYSVVCYMYSIMTATISFQDLCSLGLSGGCFVCALLINVCQIQFRTELKRVTDTMDSIAKRIIESELADKDLFEQEYKNNAAFINNLIKYTLMSMMTTPFLYFLSLPVFEWYAGNYKAHFPVPIENFFNDRLPGVYELIVITIAASISYSSAQKASNDCLFISLFKIQTSFLRYLTQSKKVIEKELLSGNSVKSQKKLLIWVELHQEIIKNIKELVLIFSPILIVYYVMQIEIVVCGAFVELKKDNDNMVQSISVGSYVTLSIIYYYILSNTADELTTEAQKLVFMEYNLPWYKMDKRNASMVKMIMTMCNVPVEITAYYGPTFLLNRENFAGFMFATLSAFLTLCQMKDVYG*

>GrubOR40

MDPPCFAIHFRPLLVGLRRCGMPTPWLKKSSSIWRSAYLFYDMFLMALVAYILSCYVYTIMTMSIPFQDLCGLGVSTSNYLSGFLATIHQSLFRARLKRLTDKLDNIAKEIIFSGLGEEHSFLRLYNKNAKLMTTLVNKSVILGAMAPPIYCLSVPAMDWFAGQYRSHFPVLIESPFDERAPVVYEVVVLLVAACMSASIAKKVITDCLFISLFQIEIAFLKYLSLSLTNMKKEFLKGDGALIDKKLKLWIKLHQSVLRSINELILISSPVVIFYYVTVICIVVCGTFVQIMKDNDNIFQSLSISVFISITLVYYFLLANTADQLTVEGQNLAHAAYDTPWYQMKKRHSSLIRMIITISSRPIQLTAYSAPVFLLNRENFAGFVVSAISAFVTFCQMKALYG*

>GrubOR41

MDAPKFAHHYRALLQGLRKVGLATPWVEKPSLLSRVPLLLYDSLLIAIVVYMLCCYLYSVTTITIPFQELCGLGVSTSNFICALLVTFYQIRYSKDLKRITDNMDRIAERILDSDLTGADHFLQLYQRTSKLMAILTNHSIFFSFTLPLVYCFPVPLMDWMEGHYRSRQPFRIANPFNDKLPGVYELIWLVMTCSISYSTSKKAATDCLFVTLFSIQSDFLKYLSTAMTELQKELKFENSTQVRIKLVLWFNLHQDILRNNQELVEAFSPVVIIYYMTTIGIVVCGAFVQSMKENELIIQSISIGGYIVITLVYYFLLSNTADELTSEAQNLAFVVYSIPWYDMVKKNADLLRLAITISTRRIEITAYRAPTFLLNRETFAKFIVTAISAFVTLCQMKMVYE*

>GrubOR42

MDPPRFVDHYRPVLSWLRICGLSTPWEEKPSFIWRQIITCYNSFLVIMVCYVIFSYIYPITKNSIPFAELCLLGIGIVLFICGLAITLYMVLFKGKLMEIMDDMDRIASNIYKNELRGAEFLQEMYKKQAKFSMVLTKNSICFGFLTPVVFCWSVPMIDWISGSYRATLPIAIDSPFDIHAPVVYELMVLLLSSSLAISTMKKAATDCFFMSLFNTQITFLKNLSVTKRYFEKVFQNGNNAFIKRKLIIWISLHQEINLNIQQLIAIFSPLVIIYSVVIIMVVVCGAFVQIMNDNNNLIQSMSIGTYVAVTFLYQFLMFRTADELNNEAKKLAFFAYDLPWYEMKKADADMVRMVILRANRTIQVTAYCAPIFMLNRETFRGFMVTSISAFVTFCQIKDRYG*

>GrubOR43

MDPPRFVQQYRGILTWLRVCGMPTPWLEHSDIISKYLFVIYGIFLAAMVSYVTICYAYTIMSTSMTFQDLCVFGISGGCYICGLLVTLYLLQFRIRLKKITDDIDSITKRIIESELGQKEFLEKEHYKNSKLMAVLTDCSLYLSFTTPIFYCVSTPVIEWYTGSYRSNLPLPTISFFDEKAPGLYELMVFFISYSIAISTSKKSANDCLFIALFKIQTIFLKYLSVSKVALEKELMTDDTYKGQRKLMAWVRLHQDIMKNVEELILYFSPVVIIYYVIVVEIVVCGAFVELKKDNDNIIQSISVGCYVATTILYYFLLSNTADELTDEAQKLAFVEYCLPWYKMKKNNISMVKIILTMCNKPIKITAYKAPAFLLNRETFAGFMISAISAFVTFCRMKETYG*

>GrubOR44

MEPPRYVEQYGELFKWQRRCGFSTPWLEKPYFYFRFLDVAYDTITICMVLYILLDYTYTVLTTSVSFQELCLFGIGFGSSACSMFITCYQILYVHRLKEITDEMDSIGKKIMENDLGGKEFFKEEYTKNAKFLSLFTRCSLTSIFTTPFSYFLSVPVVEWFEGNYREHLPLPLANIFDDRQPVVYEMVVFVLSAGISIATAKKAALDSLFISFLSIQTTFLKYLSEVKGELSKKRRFTNDKQSHMKLLTWVKLHQEIVKNIEDLVQYFSPIVVVYYIAVVEIVVCGAFVELKKDNDNLVQSISVGSYVMLTVIFYYLLSNKADELTAEVQKMVFAEYNLPWYAMKKTDVNIIKFILMMCNKPIHITAYRAPAFRLNRETFSQFVVRAISALVTFFQMKDIFG*

>GrubOR45

MFDPVKIYFNLLIKSFSLVTGEETMSTWTFHKGLLRLLGNDWLVRKGEISLLRIIWLVLYPIMYIMSMVSITTLTIKYVLQREHPTMQEYTRAMNGVVACIAFVYAICKSLVLKLLGKNLRLLMDMTDDLGPVDEVATPHRDRSVRHATLYLGLLCLIPTTWTICSMFYMHNIPFPTDWPWGDHTPFRYFISFSIDFVAATYCAVTHSTYDTIFPVCAGAICGHIASISAKMEKLGTTGDKEKDKKIITECYRLHVALLRISDHINNTFGLVFLIQSIYTVLHACVIIYQVMKVSDITLAVLNTAPILASSYAQFLLYCYYGELLTDFFERLRFAFYNNRWYQCDMELKKMLVIMTLAANRTVRLESYGITFAGHKTYVSGLQDSISYYLILKTVTTDT*

>GrubOR46

MRPPIDESQVLEGMSIALLKITGLWNAINTNLTTGRRTIGLNILTAYSIFYAFPYVLFQLISMFVIRVNVEKLTFLFLNSFPCIQVFLKVSVFWYRIKEQSDIFNLLKQDFLTCIPPHKMSKVRDIYRFWARLSNIACVMAFASMILCMSSWIIVPGIDGVDDTGAVSKKILGGWYPFPFSRAPWNEIVFYYEMILMTTHGSLISLFECVMIQPLLCLCAHFTVLGYHISTLKVVDVAYSKTKNPSEYMNSELRAILLDYDVLLRYTAVMQDILNLLVTAILGTGIVILIIGVLQFKFGDLDAMFIFHFLTFLSYQATEVFLICTSSSALHSASSDICFAIYSSDWYLADREYARTAQMIMVRTCKPLTLTAIKMYPVSVEILVGLFQFTYTAAMVLSK*

>GrubOR47

MKEVDIDSRYFRAIGLWQFVVGYEATSWVLFNFFLGCVFLVNISVQLMNTLTGGYEFSILTEKLSVNLTVMESVIKIIYYCAKRSRLYSLSQCFRRDFLICKTHDLEAGDVVLNNGFSRVNTVTKGFVIMIFTTVGLWNSFPFLRCLTGECSKWNIMPSWYPQFIDDLPAIIYIFEFFIMVFCASLLYNVNCFFSALALSLSSQFQLLTKSISSIEKSADRRKGCKSANMDVLLRECLIDHQRLLRYSSAYNWWLAKEMEDMYNPIFLFQMLTSTFTICLVLVQLNDSTSKGEMPVAMVCKFFMYLMFGSMELLVYSWGGQILYDETAEVHRSLYESGWASASHNFRKNVLIAQIRTISPEYLTAGKFYAVNLASFTQIIKASYSYFTFLHGSGGSSR*

>GrubOR48

MNTERAKSLVYLLKIGGLWINFKNHRFGPLITSFQIFRLIYLVVGWILMINVIFLKGVKFLLTPSAVFIPLGINVVVTSVLFFFKIRKMEKLILECDKNLSSYNEPWERKLIQTDTEKVKYIADCFSYGLTGFGFVYTIMHFMVNIVRAITGNPERYTVPLPFDGYLDDTELRNFNFYIYTLLSDLWLAFGVPNTFAFQCTIFYIVSCTTTEINILKEYLRKLQVRENNNANNLPDWNLNQIILKHSQIIRFNELQNQSITLPTLVQCRTIFTLSICFVMFLILQLFGKNTFVMLLAAVFILILFVLGLVLCSAGEYLEEKSDELFYAVCSLPWYQQSLKVQKTYLLLLLQTSKTIVFDYAFTSSLNLRCYMQLVNNSFSYFMIMKSLSPADADHE*

>GrubOR49

MVKILRSLLQELPGVLEITFVISKSPFRRFIQAFVTITNVLMSIANAVSLYFLGLERSLGGTASFAAYGIIICIKHAIYYFRENEIKQLLDFFVKIEKGHKKGWEKEMFEKISKDAWIVVYKYWMIILSYEILYINVLTLLDFVIGFVIPTFPSVRVDVPCEGFIEFFEPRTLKRLAVTVPILAWTMSSMTVHLGSETLVFISIIYTKIELRIIKQKLEMIKNYLEKKGDNYKIKSEKMLWQVIAQHQRTLEVLDVMKSTVGLPVAIHNTAISVTLCVIFYCLMTFDERGSLSIKFHGITLIMCCGSLVLGLCYFGESLEEEFVFQNNKVIDSIYDLPWYNEGKNFRRTVAIMLMQAQKPFVIKYRGMATLNLKTFMQIMNASYSYLMMLKSTV*

>GrubOR50

MELPWKRKNLANVWTWPHIFLLNVFGWWGEEAETDFGRKWLTRFRSLSIVYLAIFMNSLMIQVYVKFAEGDIMQNLFTVFSAGPGTVGIFKLFNLVIHRKLLKSVMDRLSGLMSEVNDPILNIIARKSLKKTWIIFLLSLTVSYSIVLHWIMRPVLAAILHKEKTRIVESWPVFLDTWAQFILSYLFQLPGVIMLGHSFYIYDYLYFCTSDVILCHLNILKHKLNGLVLNESKKSTRELFSCVKYHSTILSVCNDFRDATSKVIVWQSINTVIMLCTGIFILTYLGKNINSIVLMNLGEVSFTLFTCLYFYCWFSNEITLQCTNVSNAVYMSNWIRAKSSDKKTMLITMTRAMHPVMFGGIMKINLTTFINVLKTTFSFYNFLIAVQVSSTKNA*

>GrubOR51

MAIMQLETIEEATKNLKLILIVAAVLFSNEYYSLVPFVHATLDFGLLGYSVYHFRNSPEEVAQVLFYMGLSVAVMISIYAGYMPYYKIRILVEDSYYTYDYQSSIMEIKLDDLKVQSAEDLKQFFKTIVFLIAWVISNLTGVTILVGLIKQEPIYLFPCWLPFDINNFIIQILIILWQQYGMFTMIFMAFSGGSILYIPYTYIKKEVALLKYALQEIEPRAYEMARNRYDFIDDQHKSKVLSACYIECINMCVEHHLMILRYFSNGKGIAGLAYSTAFCAGIIACTFGGYNINSANLELRFKNLAIIFFILGYLFAMFHIADATSSELLTIAETVFTMEWYNLPKECQSTLQLMLLMSNQPLFYKLLLGQKVDMEAYMALVKATYYYLNFLTA*

>GrubOR52

MAKIKLDVIEEASRKIRLLLSLANIILSYKIHSLTTFVNSTLACCVLAYSIYHFRNSPEDLAGAAYNLGACASVPVACVSGYLAPVTLRNIVDGSCSSYDYRSSIMREKLNEVENRRAETLKGFFKTIEYLIYYSTFNLLGLTALLGIIQREVVYLFPCWSPFDQNNFFWQITILLWQVYLIISMTFMAFGGGVLFYISYSHIKSEVALLKCALVTVEERAHEMAKSIKSSQGESYTRILSLCYRECIRMCAQHHSEIIGYFNDGKYITGIYYSSGFLCGGVACTFSAYFIVSDNFALKVRYLGMTLLLLGYLFVLFDIAEETTNELLTVAETASTIEWYKLPKECHSTLRFMLLRSCEPMFYKLLLGQRVGKEAYMALVRATYYYLNMMTA*

>GrubOR53

MVKYFRYLFADLPSVLDNTSVISINPDKRFKHTFFLVFNAVMTTACGVSMYFLGVQKSLEGAAFYTTMATLNLGKQLIYFSRPTQVMKLLDTLRRLQNHHCEIWEKKTFETDSVETWNAVHTYAVIIVCYQTFILTMSVLSDFIIGIIFPKAPSLLLVQLPGQGIIELFEPRTFGWLVVTTPFIVWGYVSLMVIIGTESLTFIPIMYVKIELKMLRHKLSLLKEKLEKLGINNKNSDNLLKDIIKHHQRTIKALDVMKKTLGLPIFIQNTVFSIVICLDLYCIMSFNDVSSFGVKLNGLGLAVCVGFLLCGLCFFGESLENENYEIRNSIYDLAWYDEKKNFGRSVQIMIRQAQRPYVINYRLIANLNLMAFMEIMNATYSYFMMLKSLV*

>GrubOR54

MFSLLKRYKEEDILDDHCQKFCNEWLSFLGIWPHKTLTLHSAFNYLILIFTLVVWTYTYLEMKDGDTTEALHRICLSLLALGLFSIQFFKHEEIMSVAKQIDGCFSFSNSDVKPFFKRRQKELFTSGMKIYTDLFTILVFAIWGSILTSLYTQQLFVSDIKPPIPMILPIDSKFLYYFVFFSETCFVVIASIADVLMAHMFMIFTLQLTANFEVVCLNLKSMNIFVKNDLALVLDSEIMPKIKVNVRHHQEIFKSFNSLKKLFDQIFYILYFSMMSAIAMCRTLLVGDIDIKQLLPLFYLETGYIHIFCHFADMLAEESANVRIAAYSTPWYTFSSNICTSLRIMILRAVRPPKLYFFIGGSDISCATFTLILNASVTYFCISFMMENQD*

>GrubOR55

MFKCLRSFLGDLTRLLDHVFVISIDSDKRLKQTFFTLINAIMNIACVVSVFFVELEKRLEGAAVFSTLTLTVTVKQLIYFFRLAQVKKLLDMLQRLQNHHKEAWEKEMFQAGYLDTWNSVHTYCSTLISYLIFFLTLPTFSDFTIGIIFPKMPSLLVQLPGQGFIDFLEPRTFGWLVVTTLFILWCYQVTFIHMGTESLTFVSIMYVKTDLKILRHKLLLLKQHLENHENDNRNSDELLSDIILHHQRTIQILIVMKKTLGLPITFHNTAFSINLCFNFYCILTFNEISSLIVKLNGVGLVICVGLLLFGLCFYGESLENENHEIRKCIYDLPWYNQDKNFRRSVLLMLRQAQRPYVINYRLVANLNLTAFMQILNATYSYLMMLKSTV*

>GrubOR56

MALDVFKKHRYVLRFLGIVLPGELTNSWLRIPFHIWFRYLSISLPVLTLCFLFGITQLHDQYSVTACMYSFAGVVSTTSASFKLILLYTNRHDIKELIDFLKDFRADTTVGRITYVLIYFYEFLALCFRVIALIEIFAKRELKLIMPFWTPFPRDNVYVALCTYFFFQGTAIIKTICNFFIDTLFLLISNQACHRMLILRNTFRIIGLSEEEKLERLKNKHMLKLPPGMKPTDSNILKLCVEEHILLLKVVNKFSKIINRIFLPQSFNAFSSIVIVLFLVSQSENMIRESFKVIPWTTVIFLQLFVSCFAGELIKSRCYDFFDSSYGSEWYNCDQKVKSSILVIQTFTCRPKIIKGAFVFELSLHTFETAVREVFSTFTVLYQLFNTKQ*

>GrubOR57

MVKLFKFLLGDLPTVLDKTFVISIDPEKRFKQTFFTITNVIMTTGCVISIYMLGVQKSLDGAAYNAAMSILILGKQLIYFSNPAQVNKLLDSLKMLQNHHNKPWEKEMFDIGSLESKKAVNTYWTMVLSYQMFVLTLSAVSDFIIGNIFPQAPSLLVELPGRGIIDLFKPRTLGWLVVTTLFIVWGYTVVMIHIGTESLTFVPISYVKIEMKMLQHKLAVVKENLEKVGNNHRNSDMLLVDIIMHHQRTLKVLNVMKKTLGLPLFIQNTSFSVITCLDFYCIFAYNEISSLAVKLNGVGVVICIGLLLFTLCYHGESLEKENQKIRNCIYDLPWFNQGTKFRRSVQIMLSQAQKPYVINYHLIANLNLAAYMEIMNTTYSYFMVLKSMV*

>GrubOR58

MLKIKFASFETDPEKLTIFRNCLLYAGAVNDGTWKSKVFILYRAYCLIVGFLQYAIMGTKTSDLMTVVEVFHWLSDFTIMTSMTMTCLYYNPILLKMEARIKAGFFDYGSPLTSEQIKVRAVMNRNSQVMCKLYFSLCYSGFLATYCRVFFAKKEYTLPFPMWFPHEIRNNYQYVLTLIHVFIVAESLTICAFSQLSTFVALSSHLIAQYKILIMAIKEIDLIAVTKISSEKKIFAMQAKIKACIQHHIIINRYFDYLHKLYRIPLLTTALFICLSICTLGFLLLSPNVSVPVIGALIIVFLPETCIIVVYCVYGQKLADICEEFGQTIYYSQWYTKPLPVQRDLLMILIGSRKKRQLTSFGLYEFSMKGLSEIIKATFSYFNMLRAMN*

>GrubOR59

MKDNAKPYFVDREVVYDFDKEREIQMIMSCYKISNTSKLKRYISLLCVSILWLFLLYEMCLGFYSIPFTITTDSKTQMLETLHMALLTFYAISHLSLRLRSDFTPVLEIINKGFNTAKKYIENLYKNKPSDKSINPYFPIPFYAPFDTTSVGTFTVVYLFNVVVMYSICMVTICIDEICISLIEQLKAQFEILNLSISNVVERSLQRYLNGKVVPKPNLESLYKQKEFQDCLFKCLRENIRHHHVLLRFTGLIRNYIQITFFLVAMLSSLVLALAVLFITKSKSSDEELLGVGTFVIMLLTELVFTLQFCLYGQEISNESGRIFFTLWSTPWWCFDKRVKSVLFIMMLNTKKPITLKTTFLNVSMSLESFSNIISSAYQVGNVMVNLQ*

>GrubOR60

MPTKRDLSDFIKFLKLVGFWPYLGDRSNRIMRWFQIFRLIIFLKSVSLWILSAYIIGAAEFLKKIYAGIVLCTPYMVLQTIYIFCNGHHLLSFVTTMEATIAKRNEPWQIDILNSFLNPLWKIIRISTYYSLLLLTVAFYGPILYDTVSAIFMNAEPLNLNVTIDGLLPSMKRGNFFYYFLQWYNAFLEGLGLAHFIGLVSISPLLVNYIRVEIRILCRKIDEYSKKGNSEKEALLREIIDDHINIIRLTELTNKFLAAPLILQNVAAAAAITLFTYAMTNSISQGYDNVTALLFNLNSPIAMIMVVFLSCYSGEMLHLESLNIYDGFCQVEWYKMPKRRQKDILIILMQVQRPLRIQFRGNSVDLRAFKQVINATYSCFMLLKSFA*

>GrubOR61

MLPRFSGEKDLSDVLRLQQISGFWFHFSKKHVLLLQILRFLYYVSVFIFANIIAFRIGFKNALSGSYFFVSWGFYGIPQLVTYMLYRKRANDILEKLKILHRKRTEQWQWDIFEKHSKLVWMTIWFNGVAGGTFIVVYYSPPILHDLFRLIFNGAGDGENMFSFPYPLIETQNNNRDLSYYSIFLVSLAWSTTTIFYGLGTVGFHPIVCSYCCIEIRIICKIIKEGEKQKLLNNRIFYKQIIEKHNAILRIVEDAQLILGPAEGAQIATGGLLLTIFLFALMQIKGNIILAISHIACLCLMTLLNFSVCFFGEMLQIQGDKLFECLCGLQWDEMTPQTRKDFNLMLIQARRPIIISFKGLLPVNFRSFQFMLNTSYSCLMLLTSMQD*

>GrubOR62

MLERIKGKKNLADLLLLLKISGLWFDFEEKTTLILQKLRYCYFLTVFIIMNVSALQDGIKSAFNGPFFLVSGGYYVTLQVVIYYIYRGKIKDIIAKFVRFHEKRKEQWQWEIFEKHSNVVWIMVWFYGVLAITFILVYYTAPLLYDLCLLLFTDDYDDRYIFSFPYPLEEIRKHDRDFIHYGMQLLSLFWSMLLECYGMGSLGLQTIICSYCCAEIHILCRAIEDGKKQKLLNNKQFVKKLITNHKDILSVIADAQVVIGPAVATQIALGALLLAAFLFLVMETKDNPSLVITYILLFSVMASLNFTFCFYGEMLQTEGNKLFEELCCLPWEEMSPKTRRDFNFILMQARKPILISYKGLLPVNFQSFQFVMNSSYSFLMLLNSTK*

>GrubOR63

MWDWRQLKWLYYFGWWPAAATTETGYKINRIFGAFLFIWVFFIQTGPEFVALYIALAHGSLKGTVLNLNTVLMGVVCCLKVTGLLLNEGNVRWIIAKLEEMEIRGIDVLGAKEYKRISDYRDKRCKRLVVFVFVYLSGLVQWIIRPIYDLFNGRKTMIIEAWIPWDKDTIFGWFLVFILQFIHVTTAIMALITIYVLYLSILEMILCHIEVLHYALTKLDFSKPGVDYHSHITLRYCVKQHQDILSVCHKFYKVVKVMLFIFIMFSTLVLCLSVFELSSIKETTLFKVSNLLEFTLNTVFLIFLYCLYFHNTVDKLTLGTLRAAYSNNWYIGREEDKKSLDILCTMSRKHFEFGFIIPVNLDTFITVLKSAFSYYNFLKAISEEE*

>GrubOR64

MDVFKRHRYMFRCAGVYFDGELGRFGRAVVEVQRWVTSFLLLYMHSIGFFLGVYTRHSEFPLVPTLVMCCGMVSGPSTLLKHMMLYRRRGQVKELMDQLNGYKVDGGVGTFTKYLMYSYEGMALFFNASSILKNALENPDLTVPYPAWEAYEINTQGKRTLHFFTFYGAIALCWLVTASVDTVVLHLSNQLCHKLAILNNTLGLIGLGEEERESILKKKKVLPLKDYEDKTILKNSLDEHFHLMRLTKSLSQFLNEVFIYQGVNSMAVLLVCFYIFSNSKDLINDTVAFSGALVVVFLQVLTSCWSGELIKIRCEQIHFSLYNNQWYDADPKIKSSLFIMATYTAKPITLRGAYYVFELSLQTYETFIRQAFSFFTVMYQFLD*

>GrubOR65

MWDWRQLKWLAYFGWWPSVAKTKRGYKILRIYGIALFFYDFLQLGPELIALYLVMAKAKGLNEVIMTLNNNLIGLSCAWNIGWILFNEKNVQWIIDKLEEMEQRAKKMIGEEYDKIAEHRHKLSRNYVLVVYIFFAAICQLTIYALYWTCHGHVSFIVETWVPWNTDNLQSLIILYTLQLVHGFSGVVAYSIIYHLYMSIFEMILLEIEAFHIALSKLDFSPPGVDSHHPDLNLGQCVKFHQDLLALCRNYQKTINLVLFFFIIFSSLNLCLTVYELSSIQDRGKLIMQVAVIALTMSMSYFYCVGGQEAVEKMTVGTLRAAYNNNWYIGSVKDKKALHLLCTMAKKDFDFGFIIPANLATFITVIKSAFSYYNFLEAMNMQD*

>GrubOR66

MSVYWFFEKVAQDEEAVGFTLQKALFKLSGVFLWDSPSNMTTLYPILPMFFINIVAVLNFLQVFRFSIRTLHDFTDMVLTIIVALVFLASGLKLILLHWSKNSIKQLVDFLESLPRLESYKRIKHMEGVCMSYMYLLIFTAIPKYCLGIMKGELPFESAWPFDTNTTVGWWVAYTTDILSANFCWFAQSSFDTLLVMSVTLMAGHLARLRQILSNIGKNTAMDERIIKSAIKLHVDLLHGADLVNSSYGILIMFQSLYAIVHAVVIIFYLVKVPDILNAVALSFMLMGAYSQLLLYCSSGQLLTTEFEKVHGAVFNNRWYRSNPNIREAMVMMAVRSQKPVLVRNYSIFTALHTNFLQSCQESFSYFVVFRTLAANIKLS*

>GrubOR67

VLVQNQCLYFSLTKIYIEQIQEPTSKMAIDYMRLTRRLYVLAGIITVDNTPTGWRMKLLSNLVVSSILLFSLHAFCNGCVIAGLTKDCISQTIVFIVIHVQTSIKFGILVIKGSEDIKNILDFVEGCVFVNKSYAKHIYIASFYIIALTVIAYAMYPFVWHNLPFFYETVWGSGSFLAFASSYFMVFFDLYLIQFVSTLNDSTYLLCADALCYRLNNVKSLLESIEGEEDEGKLIRAIIEHQDILRMLRKLANTISPNFFLQVFSSLTIVIFSAFSAVTSNDPQHFSACLSSFATLLTYCWLGQMVRNATEDLHFACYNNKWYNCNRKMKMNFEILSLYTRDPFEIRGCSIVKMNLQTFKEVVTDSYSYLMVLMTMDDTS*

>GrubOR68

MFAKMMKLSGLWCYYDVSTSTKVLHKARAYTFQLINVMAQFSAFQVGLRKLYIESSVGTYITGMMIIHLQLVVFFKNNHLLNQFVYGMREILKKRNEPWQMNVFDQFCSKSWKFINFQYNVLVIFFFFFVICPVAYDLVLLLFFESPEPFFELNFIEGYFQAKIERSLRFYTTLFCTYYQVIVGGMHIIGLLGILPASVAYMCAELRVISMRLQQWSKDHTNPHRDLVLRQIIQEHNDILELIHQMKKYLGELYISQNIIVPLSLTLSLFSVTKALKAGNIDLAIRISYTFFGWTSLAFISCYFSQDLYDESVRLSETLYEIPWYKETPQVRRNLLVMMMVTKKPIVLDYKGVIPMNLRRFREIMNLTYSYFMLLQSMA*

>GrubOR69

MNFNAFRKHTICMCILGVRPPGGVRWKWLGVLLDIYLIVQLSCIAVWGYLFFQGAFLYLPDAFMDRIVGVSAAVSVLGSIAKLITFRRKGHVLKDLFEELGGFSVNTKRGRLVAKVTTIYEVSVAAIVVTHNMHIAIDPVLHRSSPYWTASQTSGTKMEIIRDYIFNLDSAIVALSANVIGDTLFLTLTNQICHRLLLLAETVSSIGKGIRPVEGLHVQEGATDREVIRKCVEEHNLLLKMTKQVQELYDRIFLVQLIYSFSDASLTLFTISQYDDFMEAMNELLPQFISLFLELFLFCWGGQMINNHFEKLHLASYDSEWYSLDKKDQSSILIIQTFTQRPVDITSTSIFHMNFHAYETICKEVFSYYTIMRQLFSQK*

>GrubOR70

MKVVKMSVPPSRWVRSPLEVSGMLASSGYPRLYYVFAIYNIVATTYCFGGSLAYFIIMEGSTKEKLQCIQITLNILSAVMKLCNAGLQQKKLRTLLLDYDGLWNQLYEELLNREIMDISAKSCMLLYTVFKYFMTLTISMNIILALVIHMVMGVDQLSYQIYMPFDHKKYYIPLQTTQFMLSASPFLINATSFFLYIIISEQLATCMRVIRRKLREEKLSKQTIREHQEIIRLVNNTNDLFSWLLYFETSATSVECSCSAYTFYKVHIKGGKRGNILANLINFGFCYFIPYVICYCGNKVTTESELLFRDAYNSMWYEHDLKAKKELRMVMLAASKTLNLQYKNIAVFGRELFRSIINVTFNVFNAIIWLDHADQHN*

>GrubOR71

MDAFRNEKIVLRLLAITLPKDLSSSLLKTFVDVYFWIQVAAAGYWSYLYFRGAYLYLHEQFLMRMVGLSAGISVSGTIAKLITFKLNSSLLQELFEQLGSFEANTKRGKLVKKVTMLYESAVAAILVTHNYEYFLNSVPTRSAPYYSQFRTGPYEEVALDYWYTLYCVFVVLTGNVIGDTLFLLIINQICHRLFLLSNTVAAIRKGDELASEGMNLRGAESDKEVIEICVREHNILLKFKNQLEQLYNYIIVVQLAYSFSTICLTLFVISQEADVYESLSQLLPQTVSLFLEIFLYCWAGQMISNHFEELRFAVYNSGWYNDCRPEDRSSVLIMQTFTTEPVQVTAVSVFHLNFHLYETICKETFTYYTVMSQLFNQ*

>GrubOR72

MGRRKEEPYSLFYKVLEYNGLALNEHSRNRKLFPVLSGILLISILVDYVVLILYVMEDHPFDDKTESFQCLISMLHISSKVFYFKFGVKEKLKRLLDDSITIWKEMRHMKKHGDFLDETRIAIDRRIKFIQGMFLLTTPLYGLIVFIRNLTLEPEKREFVFKIWDPLHFFIEKEYYYFSRLVYEAVLFTYALYTIGSVYLLYFIPCFMAATQMRFLIKMLSSKDLDVNACILFHQKILRFVRDINRLFTGQMFFEIVLSALPITFRSYQLIMMLTNYDPRALGVFFFLGLCFLVPLLVCFSGQMITDASEDLFLGTYQNEWYRLNVKDRRSLCIMINVASIPLNLCYRNTMTFNMVRYMAVMQGTYSYITILINYT*

>GrubOR73

MSTIWPQLSKLNWFGHWPTDKGIKVWLQRTASYTILLWTICLFGAELFSSQRTFYSGDLIGMFINLNEFFSGLQCYLATLVFVVYRRPLEEAMRLLEGLTQEVTNELEALAKEEFNRRRYKGKLAYYMFFGTFVMICHWAVRPLLNMAIYGESSLIVHSWVPFSMSSLAGFIGNYLFQIVPCLSFCLGFCLFASIFVCFSEMLLGHFAILGKKLSSMDYGKGPLDSRLANCIDHHTRLLKICSMFQDITSIPLLTQCIFTVLALCMAMFELTNVQGASSGRIISVFAEAQQELLFLATYCWYGNEITMQCLELLRPGYMSNWYQGTRSEKKMLLNLMTRTRKPIYLGGFIKMDMLTFINVSFFYSIVKQISF*

>GrubOR74

MPDNERNMSHPRVINESDVIEGLSIGYQKFFGVWSVIRDYRATGKRNMIIKIKALITVFLILPYMFCQYLSYFFINVDIKKGIFIFLHTLPHMQLCCKLLVFWFRLDSQSILYDLIRKDFLSIPESKRSMANEIYKNITKKSNILCRASFLINTSIIVTFIVYPEIPVDYILYHTGDINSVTTGKKKIMAGWYPLPLGKSPFYEIIFAYEIFVMIWLGMGLSVYICLFYQVLMCLYAQFAVLGYHLSTLKLEPGLKNNSIVHKNKSSLNKELLDIMNDHHKLLSYTNKLRSVYNPLVTMILGMAVFFLVAAVFQFSSVSGKSVSPIFLFRSFQNLAFQSIDVTMFCFGSSYVEHASSDLQFAIYSSDWYKA

>GrubOR75

MEPKQFMLFCLNFSGMVHVNGGLFLRAYVIYQTCAYVIFGTSLILFAYFEPKPIIAKLQTLHFLIALTQIFCKFLSITLNQRETVDILDNLESLSRDASKDQRNTEILKRVNKSSTKLPKIFMFLFCGYYPLRTINLLVRYFVFKSPQKFVEASWIPPFLASHYLTGLFYQVATCLCTGFTNAAYTGLIIFLTIQLSGQIQVLRRAMEEGYPIKQCVILHQKLLKLFFRINRLLNALMLAEYILSSLYSCISTFLILKSLSSGGKGVANNAYVLVLVLLRVFLICYCGNIIQTQGQKLHSSAYFSEWYRKSLPEQKSVHLMMALTMVQPFKYNYRRILTVDMGLFLKSINTTYSYLTMLLSFADHKQIA*

>GrubOR76

MMYQPPISDSVIMDGLYLSYLKLFGIWKVINDYRTTGKRNGIIKFQLVITLLYALPYIFTQYLSFFIIKVDIQKATFLNLHTLPAVQICCKIFVIWFRLDSLSRLFDLVRKDFLFIPERNSAAAKEIFLNISKKANFLCLAAFIVNTSVVVSSIINPGFSVDYILYHTGSMDAVTTGNKKILGGWYPLQMDETPYYELVIFYEITFILWGGILLAVYISLFYQPLMCLYAQFSVLCIHVSSIRVETDARKISGNESIDSEAYKELYAIIKYHKKLLRYANELRSVYNPLVTMILGIGIFVLVIAVFQFLFGSPGDPIFIFKSLLFLGYQGIEVCMFCFGSSYVETASSDLQFAIYSSDWYKADI

>GrubOR77

MMGWPFNRKEISEIWKWPHIFWLNIFGWWAEQARTEFLRRWLSRYRFVLCVYFTIVTSMMLIAVGVKCVQGGILDNMFTVFGCCPGIVGIFKVYCLILFSKPLKTAMEGIDELFIDSKDPNETRMIRRTLKRVWIIFTFYLIMGSCISLHWVVRPVISAILFGEKTRIVDTWPPFIDTWPQFFFSFCYQLPMILGLGHAFYIFDNIYFCISECILCNLDVLKYRLSRMKLKTEQERSKDLVHSIEYYSKILRTCTYLRDSSSAVIIFQCIITVTILCSGVFIITMAEHTEVNVLMNLGEESTVVIFVLYFYCWYSNEIRFQCQDVCNAAYMSDWINGTEENKKKLADSDDQDCKTCYVWWNP*

>GrubOR78

MEHRQPIQDSDVIDGLSIWYLKLFGFWKIINDFRKTGKRNPFFKFKFIMSILISFPYIACQFSSYFIIEVDIQKATIINFYCLPAVTMCSRILVFWFRAESQCRLFSLIKKDFLDIPENKKPQTRKIYRNVSKSCNLMCIVAFVFDFSVVLTTVGIPGIPVDYILHHTGSMFDVRTGRKKILSGWYPLPMAEYPYYEIIFVYEMMCVLLGGIFLPIYLSLFYQVAVALHAQFLALGYHVSTLKINTNKNEKKNSRSTGIKEELYKILKDHQKLLSYADELRSVYNPLITINLGGAIGILIASVFQSHVGETRDIVFVLKAIQYAASIIVELAMFCYSSSLIQAATELGSSLRSLQQRLVQG*

>GrubOR79

MRYRAIADSDLVDGLSVRYLKIFGLWKVINDYRTSGKRNAIMKLELAITSLLVVPYICFQYLSYFHIDVDIQKATLLNLHSITGAQMFCKMFVLWFRIDSQCRLFNLVRNDFLDIPLDKKAHAKEIYKNITSKSNVLCNIAFFINASIVTLSILVPGLPVDYILYHTGSMDAVRTGRKKILGGWYPFPMSQSPYYEAIFVYEAVILFLGGIFLGSYLCLFFQVLMCLYAQFTVLGYHLSTLKIGSEHGDIKRIIKNKQTYSRMYKELYGILKEHQKLLSYANELRSVYNPLVTMILGMGLLVFIISVFQFLFGNERNPMFVLKSLQFLAYQSIEACMFCFGSSFLETATELGSALRHLQQ*

>GrubOR80

MCHRAPIADSDLIDGLNIRFLKLSGLWNTINHYRTTGNKNFNLKFQVIITILLAAPSIICQYIAFIAIPIDVEKATILNFHSLPLLQVCCKHLLFWFRLDSLCRLSNLMMKDFLEKCTPGFELKKVVNLYRKTIKESNIFCWVIFVEMSIGLAVLCFHPDVPIDYVLYHTGSMFEITTGKYKITCGWYPIPMDQSPWYEVVLFYEYITMAWMFFFIFVFLCLYNQLLMCLCAQFVVLGSHITGLNIDSNYQKNTSRYKTMNLRMNNELYKILQDHQKLLSYTAELRSVYNPLVTLNLGIGISVLVVAVFQFLFGKANDPMFLLRAIAMLVYQGIEIVMYCYCSSFIETASSDLQFAIYSS

>GrubOR81

MINEGISESDIIEGLNMSYLKIFGLWKVIHDYRTTGKRNGIIKFQVFCTFVLTLPYVICQYISYFNIEVDIQKATFLNLYPLPALQMCCRIAVFWFRIDTQCRLYDLIKKNFLNIPEERKIEIEEIYRKNGKVSNLCCTATLIVNATIVSLYVVNPSISVDYILYYTGSMTEVKTGRNKILGGWYPLPMAESPYYEIIFVYEAVCIFWGGLFLAVYFSLYYHVLVCLYAHFTVIEFQISNLKLPSCGSKSCIKRDLYNNMTVSEDLTRIIQDHQKLLRYFEELRTVYNPLVTLTLGIGIVVLIIGAIQFLLGKSINPGFIFQISEVFALQGLEVCMFCFGSSAIQEASSNLRFAIYSSD

>GrubOR82

MADWELDDTLQTRSYDHLTDKCRACTVISIVSSPTMLTVYYAFYHMRIKRFFELSDELSERILNSELSQTENFRRNYLSVAKSNNSLTKIALTFIFWTPILYNLPTPLIDLYNQEYRKTHPVYLLYPFDVHKPGTYEITFILLTLGLLCGDMKKFASDCFFLTTFRTQIVYLKFLSASIRDLGQEFKKKEDFILKKKLIKWIELHNHFIRNFSELISLYTPVICIYYANLMSTVVLLLFTQLQEEKNGLIESLGLAGFVSANVFQLYMQCATNDDFSVEADNLALGIYNTPWHEVEKKNKVMILIMLIVASKPVEITAFKSPTLRLNKEAFLSFVASTITAVMSFKKMSDLQRD*

>GrubOR83

MALQPAIGDSDVIDGLNVWFLKLYGIWRAINLYRTTGKYSNIIRMHIFVSFFITIPPLIIQWQSIFVLEFNFQKAIFLYMHPLPALNLCCRILFLWFQMDKVCRLYDLMRKDFLNIPMELKEEVAELYKKTNKISNLCCAVLFLWNHGIEFWFIVFPKIPVDYIQHYTGSITAVTTGRKKVLSGWYPVPMDKYPYYELIFVYEAIILIWAATFLAVYFSLFYQLITCLYAQYVILGFRLSKIKFDFQNNNDLIMNKDLHEIILEHQKLLRYTDELKSVYNPLVTMTLVIGVLVLIIGAVQILLEQTSDLSFIFQLVQIFSTQFIEVSLFCFGSSNIEKVSSDLQFAIYSSDW

>GrubOR84

MVGLREGDLVDLLENSYLYSREGKTFKKFLNAFMGAYSVGTLLVCFGAAKEAGFRKCMEQTAFYIVIGIITSLQQVYLLCNQDQVKKIINVLTGYQERHVGKWQVDMFKRDSLRVWKIIRYYNIVMSSYNVFYFSLPFIVDLVGGLINSDFPIVRFPLPNQQYVEDYEPRSWGNVLYSLIGITSGILTIEMNKGIEALIYISIIYTNTELQLVKKKLQVIKEVMERKDINYVDVERLLKDVTRHYQSTLEGLKTIDTTLGFPIAVQNTIYSICFCIDLYVLTIFDSEIKPGPFIVSIFSLTIMATLLFLFCYIGESLENTVIKIIQF*

>GrubOR85

MKKLNDNTDPEDEKYFKTGFKNNYGVWLICGGMFLGKPLLPIGFLLSVLFMITFMCGTMTKCFKTDLVAAIESLHFFIFVIVESIAMITFLQKRSLIVSVYTIIGKGFFDYENTLDDECLELKREAYTKMNARKRLIHHTFVTVVMSACIAISVFRPAISILFPDENQGSPDDGVVRIALVPAWTPFDKSQWYATAIVLLIEYIVAWTTPGIVFGATFFVVFSLEELGIQLQLLKKGLNNLMKRAERLESHMEANIRLCLKYSIRHHQFLFEFIDMVNQVFSLPGFGLFVSISLMLCISGFIFTLGNSRK*

>GrubOR86

MNAGLGHSDFFKETYLQVAKNNNKFTIKVLFLIFWAPGLFFIPTPIIDLYNGEYRKNEALNIRYTFDKHSPGFYELMFILESLTIVFGNWKKFANDCFFLAVFRNLVVYLKYLSASIRTLGQDFEKNSDDGTKRKLIKWLKLHEQVIRNTNELISLYTPVICIYYANLICMVVFSIFTQLKHESESWQSIGLGAFCILNFFQLYMQCSTNEEVGTEADKLVFEIYNTPWYEMSKSNKDIIRMIQRMAYKPVDITAFKSPTLRLNKESFLTFVSSTITAVIAFSQMSDLHH*

>GrubOR87

MIWNLMYNYCIILITYMATYQILPFMIDTLVGIHYPYFPSLRVHLFAQGLIDFSKPRTLINITLSIPSITWCCLEILSHIGAHALLLVSMAYLRVELKIIQQKIQIIKNIVKKKRKNIAEKLLIDVILHHQAILHGLDTMKSILGFPVALENTTISICFCLNLYGIIVLEGKESIAAKFEGISGMVSLLLILYWSCSLGGSLEDENSNIFQCLYDIPWYYEDTKIRKLLIMMLRQTNKPFVINYHQKADLNQHTFMQIINSSYSYFMVLKSTTS*

>GrubOR88

MMRRLDFADQEDEKYFNKGFKENKGMWLKYGGLFPGPLWIPVIFIFTGFVGIWLMAVTVFKIYKSDLIVAVENVHFIIFVSVELLAMISFLRKRKLIVSIYTTIGKDFFDYENTLDEECLEVKRSVIEESTKRMKIVYNTFITVVLSACVTITVLRPLLKNFYFDIDEEDAYDSFNRESMVTVWCPFDKHQWYAKIFIWHCECFITLNTPAIVFGATFFFVFTLENLGVQLRILKKSLANVIKRACKLNMPREEAIKLCLKFSIKHHQILFK*

>GrubOR89

LAPILDKYGTFDFDIETSINFNLPLPMVYVFDSSTVAGYLVALTHQLLSAMLCGLIFGGCGYMYIVMVSSLSLQMKILIHRLDTIEGRALSLYEANYGSKLKLNDYNLYRNKKFSKCMDICLRKCVEHHKVILSNINELNDFSSMPLFAIFFVAALIIALAMLGSSFWKVLPGNTIASLVLCGTEIAFLGTLSSLGQSITDLNEDIRTAIYGIKWYHCNKRFCMNIRILLVKTLEPVTFTACGLIKICYDTYANIINSAYSYYNIVSATGS*

>GrubOR90

MHWPWKRRNLSDVWKWPVISWLNMFGCWGEEEKTESGRKWRIRVRTLTMIFLPYLLMSMLIQGFLKFSEGDIMQNLFTVFAAGPGTVGLFKFFNVILYRKQLKSVMDRLSAMLSEADNPKLQQIARTSLKRTWILFIACLSVFTCSIMDWLMRPPIVAIIHHEKTRIVDSWPVFLDTWLQWFFSYLLQCPGILLLGHSNYMYEVLYFCTSDVILCHFKLLRHKLNHLVLSNNRKSTEELISCVKYYTMLLSVCEDFKKSYIKGSYMAVP*

>GrubOR91

IKQEPIYLFPCWHPFDINNTVFQVLILLWQQYLLSTMIFMAFGGGSMLYIPYIHIKSEVTLLKYALIKIDTRAQEMARKVLKDKSNKDKILLACYKECMKMCIEHHIEILGYFYRGKRLMGIIYTTGFFSGVIACTFGGYNINSENLALRFKNLAILFFILGYLFTMFWIADATTTELLTIAQTVFEIKWYILPKECHSTLQFMLFMSNQPLFYKLVLGQKVNMEAYMSLIKATYYYLNFLTA*

>GrubOR92

MSYHAVIEDSDVIEGLSIGYLKFFGLWMVIRDNRATGKRNVVMKFNLFIIIFLVFPYILFQFLSYFIINMDIQKASFIFFYILPYMQISCKMLVFWFRLKSLSKLFDLMKKNFLSLPECKRSAVNGIYKNIAKESNLFCKASFLVNISILFVFIFFPEYPVDYILYHTGDMKAVTSGKKKVMAGWYPLPIDKYPYYEVIIFYEAVSMIWGSMLISVYICLFYQVLMCLSAQFAVLGYHLSTL

>GrubOR93

MVSPASCLVTLIANMMQEPEERGKPFVVWDPVPESWYWTSCFIEGCCMMVVLSMLGTTLIACYGNCLQAAIQMRILQEMLKDSALDMRACIHLHQNILRYIEDINNYFSGQMFLEIVFSSLQTAIRGYICFKFLNAGNPKVISSFFFFSLCLLGPLIVCLSGHVITDSNEDLFRAAYNNAWYSASPREKSSLVLLMCQASKIKRLNYKNLLDFNMERYSVVVQGTYSYITLLQGADL*

>GrubOR94

MTDQDEKGYVEQYEMNFLHGIVIFASKPHIKIPIPVLILFGSTMFITITIHACMHAYTMDLMDELINYGFTALILSSLFCTFKYRREIHSLTRDVDSWGEFTYLEEVAKEMKLNAKEWVQRFSSIHMILMNVGSVAYCLVPIGKYLYLEPSDPMDLLIFHVPFPLDTDWGFYSTYFFQSVIIICSSLGISRVFGYKIICFLTIGRQMRLIGSALSSLEQRVGRLIIEKKLLQW*

>HhalOrco

MQKIKMHGLVGDLWPNIRLMQLTGHWLLEYHEETGGMVRLIRLAYCWLTTFLVVMQFAFLACFLILDTYDADQMAAATITTLFFLHSITKFAYFAIRSKYFYRTFGAWNQVNSHPLFAESNARHRATALSRMRKLLMIIGVITIMSVMAWTTVTFLGDPHREITDPEDVNSTITVEMPQLMVDAWYPWDAKTGFCFFATFIYQLYWLFISLSHANLLDILFCSFVIFACEQLKHLKEILQPLMELSATLDSVVPNSGELFRGGSGGSNMPLVENDGNDFDIRGIYSNRGDFSGFGQTAVSTIQTNGNGIGPNGLTKKQELLVRSAIKYWVERHKHVVKFVSSIGDAYGSALLLHMLTSTVTLTLLAYQATKIEGVDVYASTVIGYLLYTLGQVFVFCIHGNELIEESSSVMEAAYSCHWYDGSEEAKTFVQIVCQQCQKSLTVSGAKFFTVSLDLFASVLGAVVTYFMVLVQLK

>HhalOR1

YPPLQDSNIIDGLRVGYLKFFGFWKIINDYRTTGKKNTFMIFKFYTTFIISTPYIVPQLFSYFAINMDIEKATIINLHCLPAIMMCGRILVFWFRMDSQCKLYNLIQKDFLHIPEYKMAKASIIYRKITKNANLLCILAFVMDFSVAFAIASIPGVPVDYILYHKGSMFDVKTGRKKILSGWYPLPMAESPYYEIIFAYEMVCVLTGGLFQPIYICLFYQVLVALIAQFEVLGYHVSTFNISPNSDISQINYNPSAECSIIDSDSYNQNNINSNSNISHRKKITSEKSDSDISEDLYRILQDHQTLLSYADELRSVYNPLVTMNLGLAICFLIVSVFQYQSGETRDIVFVIKALLFMAAQMIELFMFCFGSSSLQAASSDLQLAVYSSDWYKADVKLRKTAQMLMVGAKKGVTLTAIKMYPVNKETLMSIFQFAYSTSALMSGMLEK

>HhalOR2

MVFGISPNKWLHDKVKKLNESVDNEIDVYAFIDKEYFRLLQISCIYVKLDKRNRLMSLIQWSFYVTVLFYHYILLTRSTIFMMDINMVLFSQSTHFTLLVQLTTILIVWLQTRHRQITLFHKLLSVDFYDYQEPPAKGADALRNGMVRERRLLSAIPVGAALAAGGVLIIAPIVDRNAGLFDFEEMSKEFSTHMPYPYGKYPYQTIEGISYYLTLGGQLIIGLLLTIIIGSGGFIFLNLAQNVSLQLKLLENSMDQIETRAEFMYLRKCGKTRRDTPSLYNDHQFIYCYNKCLRKNYEHHQVIMRAFHYMEETVSVPMFLAYMTGTIVIALSLISAGSADELPGTTLASMILCGTEIGYMFLFSVFGQRLGDLNMDLRFKIYNIKWYLCNRKVKSNMMIFQERTLKPLTMMAGGIVPANMETFATVMNSAYSYYNLVNAFGN

>HhalOR3

MSPKDKAGYLVEKSNIESDKLTEKFIDSNYYYLTQLSLLYMKMDKKHRIFSIAQFLTFYTIMGYHFILLIRSSVALFGINFIIFVHNSHIGLFSILVPTVVLDFQRHRSAFFHLHRLMFTGFYDYEEPELITVANVREDMTRQRRRLAVIPVSACLATGAVLVLAKFLDRLGTFDFNKTDGLVSDDLPYAFGVYPYNTKSGPGFYISYLLQVMHAAVLAAPIGLSGWTYIVLSQNIMLQLKILIASLESIEERSKQMCERTFPGRFDASSQKTFDSKAFMYCYNICLNKNFEHHQIIMSAFHSLKELFSVPVFLSYMAGTIVIALSLLSSGSGIELPGTTIASALLCFAEVGFMYLFSVFSQHITDLSLDLRFTMYSTKWYYCNKSTAQSIAIFQTMTLKPMTLTAGGIVPANMDTFGKVMNSAYSYYNVVNAFNITV

>HhalOR4

MWPVDKITHMFDAMSAESNDEVDDYVSAQYLHLIQYSAMYTRLTRKHRLFTLLEIIIYEFILIYHLIHLGKAALMLIDVSLVTFGETIHFCLLIQLDIILIIFIQTKHNKIALFHRAMAKNFYDYSESYQNNMEELQQDIRKERMLLAMIPITVVMAVPAVLVLTPQVDQYGTFDFSKISTEFNQQLPFPYIIYPYHTEEGIGYYTAALLQVVLATILGGSIGLGGFAYIVMSQNLWIQMEILYDSVNHIEERAEILRSDLFGAKNKLSGDKLYQQVQFEYCYNICLKKNFMHHQIIMRNFHLWADIFSVPTFLAYLTGTLVIALSLLSLGSWVELPGTTISSLILCAGEVGFMYIFSVFSQRLTDLNEKLRFEIYCIKWYLCNKEVKTSLKIFQEVTLRPMTLIAGGLIPANMDTFSKVMNSSYSYYNLVTAFNVA

>HhalOR5

MWLVDKVTQIVDAMSAESNDEVDDYVSAQYLHLIQFSAMYTKLNRRRRLFTLLQIIIYEFILIYHLILMVKAALMLIDVSLVTFGETTHFCLLIQLDIILVIFIQTKHNKVALFHRYMMKNFYDYSEFYELNKEGLQQNIRKERMLLAMIPVTVAISVPAVLVLTPQVDQYGTFDFSKISGDFNQHLPFPYIKYPYQTTEGMGYYVASLFQVIGACILSGSIGVGGFAYIVMSQNLWMQMEILYDSIKHIEERTEILQFKLFGARNKLSGDKLYYQTQFAHCYNICLNKNFRHHQIIMRNFHLWADIFSVPTFLAYLTGTLVIALSLLSLGSWVELPGTTISSLILCAGEVGFMYIFSVFSQRLTDLNEKLRFEIYCIKWYLCNKEVKTSLKIFQEVTLRPMTLIAGGLIPANMDTFSKVMNSSYSYYNLVTAFNVA

>HhalOR6

MAGMFSQFIDFLNTGGDEKTDLFIEKTFMKLTTVSAIYPILDKQRPLVCLGNFALFLFHSASLTYSWILTAKTSVILFDINFVPFVHEVHLTLLLILSVIILWHSWLKRPNVAKLFRFIDKDLFDYEEELEDKLKLLRSDRIKERRRYILCVIMIGVAASVAVILVPTVNKLGTFNFNATLYEVNFELAVPVTYPFTSMEPVPFFFENAYIMLGATAIALMNCTKSFMAIESNLTLQMHLKLLIYQIENIQVRSAKLYRQLYGVKPKLGGLGSYTPKFLKCYNICLKKTIQHHHVISEVVVEFNEVFGFYVFFYYLTGTLDIAMSMLATSSTDEFPGTTIGAMIICLVEVCFVALFAHMGQRLTDLSVDLREVIYNTPWYRCDQGVANNIKILQIATLKPISFSFYSLLNINYDSFATVMHSAYSYYNLVNAYNK

>HhalOR7

MGVLERLRNFLQDWNRWSDKYSDIMIQKEYRGSMHIIGLYPNVNKVGLFWGSFRALLVGVLLLFHTFGLVVSAVLLYDINFIYFSTALHYVLIVGIAFLYVLYFNTNRHRFARFHTKMYFNFYDYHEEITDEIVDLIKKGEKQKMRFTILPMFAAFIAVCILLVAPILDKYGTFDFDIETTINFNLPLPMVYVFDSSTGAGYFVALTHQLSSAMLCGLVLGGTGYSFIATVSTITIQMRILINRLDTIESRALSLYEANYGLKPNMNDYDLYRDEKFSNCMDTCLRRCVEHHKVILSNISELKDFFSLPLFSIFFVAALIIALAMVGSSNWKVLPGNTIVSLMLCLVEIAFLAIVSSLGQSVTDLNEDIRNSVYGIKWYHCSKGFCLNIRILLSKTLIPVTFTALGLIKICHATYANIINSAYSYYNLASVTSS

>HhalOR8

MGLTTSVEGLLRKWNDASDEYSDIVMQREYRNYVHIIGLYPNINKVGYFWSCFKIMIIGASLLFHSIGLVVSTVLLYDINFIYFSSALHYVFVVGISFFYNFHFNKNRKFLARYHSRMYFNFYDYKEETTDEIRELVKSTEVQKKRFMILPIFSACAAVCILLLAPILDKYGTFDFDIETSINFNLPLPMVYVFDTFTVAGYLVALTHQLLSAMLCGLIFGGCGYMYIVMVSSLSLQMRILIHRLDTIEARALSIYEANFGPKPKLNDYDLYRDRRFSKCMDYCLKRCVEHHKVILSNIAELNDFSSMPLFAIFFVAALIIALAMLGSSFWKVLPGNTIASLVLCGTEIAFIGTLSSLGQSITDLNEDIRTAIYGIKWYHCNKRFCMNVRILLVKTLEPVTFTACGLIKICYDTYANIINSAYSYYNIVSATGS

>HhalOR9

MNIIDKFTDYFESIDTSDDETNRFMEEKYKGWLQITLIYTKLDRKNIHKTCIQYFFYVLVVFYHWIFVVVGLIDTMGINFILYSKGAYYLGFIQMACCGIITTTVYRDKLVIFHRSMSKNFYDYKEKDTEFIERFKKECDRDVKLFSIVPVSTVIGAVIGFVLSPVLDKYGTHTPEEISSLNINLPAPIIAYQFSTHNNMGFFFAYFLQVCTGCVVIFIIAGGGYLYIIMVQNVCCQLKILIYSVEKLEERADECYYQMSGLKMKHTEKEKFSDQLYDYCYNQCLKKNFEHHQIILRLFDCVRHFFSVPVFMAYLIASVVIALSLLSNNIRELPGTSITSSVLGCIEVSYVAFISFLGQEIIDLNDELRVKMYETRWYYASNQVKNSIKIFQQMTLKPLVIMSASIVPINLDTFAMVMNSAYSYYNLINAFSD

>HhalOR10

MWFLDGSKEAEDSGPVEAFIDKQYYRIMQVSALYLRMDPKNRVLSFLQLITYVVILAYHWSLLVNGTLVLFDINMVLFSQSVHFSLLVQLAMILPICFQIKHQKMYQFHKMLSYEFFDYEDPPIIGEDHLRSAMAQENRKMFMIPVGVVAAAGAVLIMAPIVDMNAGSFDFNKTANIFIDNLPYRYAPYPFPVEDGFGYYFALLGQMLCGLLLTLIIAGGGFLFLNLSQNVVLQLKILHSSLDNIEGRTEHLIIKLFGNFDKKKDSYGDSRYAYCYSICLRKNFEHHQIVVRAFKILEELASVPVFLAYTTGTIVIALSLISAGSMKELPGTTLASMILCGVEVGYMFLFSVFGQRLSDLSSDLRFKLYSIKWYLCNNKVKSNMLIFQEMTLKPLTMTVGGVVPANMETFSTVMNSAYSYYNLVNAFDGS

>HhalOR11

MWQYHEDSEANRVVDTYIFEHYYYLPQISGLYPKMDGRNRIFTTLQLIISVLTLLHQMAILIKSTIALYGFDMVLLCQNVHFCLLIQIALITVGNCLWKHFSLYDIHRLLSYDFYDYQEPPGIGEELLRSTMETERRRLNCIPIGVAIAAGVVLALSPIIDFNAGSFDFNRTASVFSHHLPMPFTQYPYHVKDGFGFGFALFGQLGTGFLLTAIIGGAGFLFINLTQNISLQLKILNNSLENIDSRIEHLYSKLFGKMDRNIMKSLRHDSRYAYCYTKCLRKNFEHHQVILKSFHLLEDICSLPMGISYLTGTIVIALSLISTGSAKELPGTTMAAMILCVVEVSYMFLFSAVGQRFTDLSNELRCKMYNTRWYMCSKEIKSSLMIFEEMTLKPMTMTGARIVPANMETFTTVMNAAYSYYNLVVAFDIK

>HhalOR12

QGIEASPSELPTMFGQEKVRSILKNYFTTEGKREDSLAFEAVRRFIHGMVFYKTYVIPLPFFIVLNSIMVVLLLTENIIQIAHINWFGKLVEYNLSYPAVFYSLLVIKNRKDLIYISESIDVWWNYPFLEDKTKMIKKKAEAWLKRFTSAYYTLMILGFLCIFFQPLGKFIWNGRTDLKDLLLFKCWSPVPLDKTWGLVTIMVFEIIAQFSPFFMYSAIGSYMITMTILFYHQTNLIGVAFTTIEKRVWMMVRARTETSNGAYQIYSKNLQQEIANCARNYQNLYEYLKKMCEIFNVTTNILYYTGMCLLVSSGLQIVTKKTNALLLFQAVMLITSVIVNQYLNSFITETLSEAQSSLRISVYNCDWYNMPKKCQININLMLILVSYSPKLTTILGARCDKEFLSKVINASYCYFNALHSMRADRTGSLE

>HhalOR13

LRVHQFSGVMRHLAAITDSDVIDGLNIGFLKIFGVWGIINDYRTTGKKNFVSKFQVFITLLLAVPYIFCQNLSYFMIKVDIQKATFLNLHALPALQMCCKVMVFWFRLDSQSRLYSLVKKDFIHVPEEKKDEAAKIFKKITTKANLLCLAAFLVDLSITAFSIADPSISVDYLLHGTGSMTAVVGGKQKIMGGWYPLPMADWPYYEAIFIFETVLMIWSGVLLALYVSLFYQVLICLYAQFVVLNLHVSTLKNHFSIDCDRKNINNREVHSSLNKEEIYAVAKDHQRLLSYANELRSVYNPLVTMILGMGVFVLIIGVFQFLFGKSTKPMFIFKFMLLLAYQGVEVSMFCFGSSALETASSDLQFAIYSSDWYKKDIQFRKAVQMVMVGARKGVTLTAVRMYPVNVETIMAMLQFTYSVATLMSRMTE

>HhalOR14

MTVKKDELDEEFFYDFRETRKWYKIFAGSSMSKQMKPTIFLEIYRIFAFLFLIMMTFMFLFTALLTTEYLAEFSEAFHYFCILLTMAIYIPVERLAKNDIDKSFLKLKKCFYLYNGELNNTQKKIKRQTIEYIKFTDRVFFWMLIFVCVLYSILTPLKDYFYPHLRLERSEIIDRKLPIPFYIPFEDSYGITFIIVFLMECCCNMMVHSLITSIHEAYISLTGQIYGEMKLINYSLSHIEERAIKLYLSKQQKRVHRLKCIYRLPAFQKCFKKCLKEDMIHHQTLLSSIKLVRPFTGRIIISVFTLCSIVWAVDVYVISKMIEQASAMDKVLEFLLMLFTEAVYVFDMCHFNEKIRSENDELFMQIYSTPWYNYDRKLGQNVHLMLMNTIKPTKLNSNLFNVSASFETLMPIISATFSYFNVLRKLKN

>HhalOR15

MGKMFDVTEDIEIDKFIDKQYFYLMQISCIYIKLDKRYRVFSIIQWIFSFGNLVYHYLLLARSTMLMMTFNMVLFSQSSHFALLLQLTMILTFYFQTKHRELMLFHKMLSHGFFDYEEPPPPGDKELRSAMLRERRILIMIPTAVALAGGVILVGSPIIDRRLGSFDLEVLDREFSTHLPYPYGKYPYQTKEGFSYYAALAGQIFNGSLLTVFIGTAGFLFLTLAQNVSLQLQLLQNSLQHVEVRAEEMYFRLFGKTKNDGTSLYEDPQFSYCYSTCLRKNFKHHQIILRAFEYTEGIMSAPMFLAYMTGTIVIALSLISTGSVQELPGTTISAMILCGVEVGYIFLLSVFGQRIGDLNMELRFKIYSIKWYLCNRALKSNLLIFQEKTMKPLTIMAGGIVPANMETFATVMNSAYSYYNLANAFGS

>HhalOR16

MQLSEKDYLLKGYQGSQCFLLRIGAMYVLQDENEVYNWIFVGIFHMHYYVFLFWILPYDVSHTIPMGHMTATLQALHYYCTFLGYSIVNYLYHFNRKSVNFCLTLIGKSFFEYEEGGTEIEIKAAQESDKNQKEYIKKLTRYVMVIVFATAFAVLMLPPFAELLTRDGPIPDDEIMNPYLPIPLVLPWDSFSIGGYLLIYVFLFLVSYNICTELLALSMGYTSFLIIYINQFKILNNSVLKIEERATKRYKKVAVKQLKGMEKFDDPIFQECMILCLKQNIKHHQILLKWMDEACHFLGWGVLCTIFTTSFLLAASGFLITMESDSSLLKSLILVQVQILELLHAYLFCWWGENLATESAKLYHSLYKTPWFYCGKRFNKLMQIMMNRASKPVIPRDPLFKINASLEVYMSILSTGYSYFNLLRSMN

>HhalOR17

MKLLISDYDVEIDGVIEKTYSGLNTISAIYPVMDMERPRLCLINLFLFLFHSVILTVMTSSLVQASLELFYINFVPFVHELHLAFLGLLSIAILWHSWLKRSNVTRLHRIITQGFFDYKEDLEDKMKVLGEDRIKERKRHILYVILIGFAASVVVILIPAVNQFGTFRYNSTAYKVNFDLPVPLPYPFMGTEPLDLLPGYTMIVVTASSIALMNCTKSFMAIDCNLHIQMQLKLLLHQIEIIKSRAGRLYIKLYGTEPKYNGLKLYDKKFMKCYRICLRRSIQHHQIIVRALNEFNEVFGFYVFFYYLTGTLDIAMSMLATSSTKEFPGTTIGALIICIVEVGFVFVFAHMGQNITDLSVELREVIYDMPWYRCDQETKTTMRVLQIATLKPLSFSFYSLLHINYDSFATVLHSAYSYYNLVNAKN

>HhalOR18

MDTIKLSAEDIPIDRQITHDYETDRKNLMYMSGFKVNNDSNLKYILSKIYVYAIIFFYSFLVSMGSVAAILIHDKKLKLEAVHWTFLNLVGLIFMVIRYTYVLDEPLKTIRTGFFTYENEFIDDRYLERKKYHVRRIRIVSNMFLGLAISNGILLSFIKQVKKSSEVGNYGKSINIGLPIPIFLPFDTTTTNGFVLGYLINVVAFFYLVVITSSAQQIFLSFMEQGIVQFEILNISISNIEKRAYYIYSKGKACAEGISIELYRTRKFQNCISQCLRQNVRHHQTLLRFRNDIKTYVEAVFVLLILASIVVFAAIMFVILELQDLSEASTLFWLLSTEIIYVFVVCIYGELFSQESAKMFYALWDIPWWNFDKRNKVILHIMLSNSMNPVVINAPFFSSNMSLESFGDIMARAYQFMSVVRNVDRK

>HhalOR19

MLKDIKAILKEKIEQAVGKALMENQGIYMVISGVYPVNIYYSLTAILCQLFTLSFFMYTCIYFYYNEYNLSMFSEAAHFVVVVFCSTSFYIIFWFRKDKVHNITKLLEQEIFKYKGTLDLETRSEIDEMTLKMKNRKNKISNIMTIVYIICGLFGVILLPIIFKKSAITVKEERILLTVPVATYYPVRGDFGWYLCSAQQYLTTFFIISALNGSDVAFSCYCEEGCNQLRILCHAIRHSLRRARYLHTHIFGKEPLTLRDETFAICLKMCLDESVIHHYRLIRYFKEIQSLFSWCVMIVMMAGASMLCLSGIYFFFDVVGFGTKISFAFYLSGELMHTFIYAWYGQQISEMSAEIREVLYEIDWEDCSKTVKPYILIMQAYTNNPIKLKGGDFMEYNLNTFGNVCSTAYSYFNLMSAAVPSSS

>HhalOR20

RLRAARAMKYQAISDKDIIDGLNVKYLKMFGLWKVINDYRATGKRNAILKFEVGLITLLVIIYSICQFMSFFNIQFDVQKFTFLNLYTLPGFQAVFKILVFWFRIEDQCKMYNLVRKDFLKIPSHKEAHVKRIYENISAKSNMFCNAAILINTSTVILWILYPGLPVDYILYNIGSTDVVRTGRNKILGGWYPMPMGETPYYEIIFVFEAVMIIIGGLNLAVYICQFFQVLMCLYAQFAVLGYHLSTLKFNAVDGDDRIEDSENNAMYKELNEILEDHQKLLSYANELKSVYNPLVTVILGMGLLVLIFSVFQFLFGSLGNMMFLFTSLQTLTFQAVEVGMFCFGSQFLETASSELQLAAYSSDWYKADIKFKRAVQMMMVRAKKGETLSAVRMYPVNVETLMAMIQFTYSMITLLSRMTE

>HhalOR21

MGYEAVIKDTDVIEGLNIRFLKLFRMWNAVNDYRSTGKRNIIIKIYIFGSFLLTVPAAIFQIESVFTIEFDIQKATFMYMHTLPAISLCCRIIVFWFRMDSQCKLYDLMRNDFFDIPERLRGKVKEVYKETNKLSNICCLIPFIWNTGIEIWFIAFPGVSVEYIQHRTGSMSAVTTGSKKILSGWYPFPVEEYPNYQLVFAYETLCLLWAANVMAMYFSLFYQILMCLYAQFVVLGFRLSNLKVEFRDDDIATQKTSGQEKKNPRINEELNAILRDHQKLLRYTEELRSIYNPLVTMTLGLGILIITIGAAQLLLGKTSDPSFLFQLFQIFSFQFIEMSLFCYGSSLIETASTDLHFAVYCSDWYRADTKFRKSAQMMMIRARKSMNLTAIVMYPINMETLGSICQFTYSTAALMSGMVE

>HhalOR22

MVVTLWSFLKSYFLTQEKPEKAGYVFNTAHYFLYGLTVYKKKTRLAIPMEFILLLSSVVMFSSILQSLLDFWKTKWIELLCIYITLVTPILCSLTVILHRERIRAVSEQVDRWWNYPLLDKETKKMKTLAAVTMDRFNRYYFSAMYVAAAICGVLPFQQNNLSAEKMKPMDKLIFPMWTPFSIDGSWGFFAASLLQVLAIWFAYFSFGRLFSYFMSLSFTISNQMKLIGRACQTRLRRVEIMMKDLDITDNRSLDQLYIEMMMKDINCCIIHYQILYGHLKEIRRAFSLITNLTYHAGMWAMCLFGVSFVKEKENPYTLIRLASAIVSLFFHQYLISYVSEVLTDELENLRLAVYSSQWYNVPQKLKKNIHLFLTLLLYIPTLRTIMGTKTNMENLSQILNTSYCYFNIILTLKSRFSVE

>HhalOR23

MELLAWPRSLLSKYVAAVIKDEPRGYVYTSSVTIRFGIAIMEIRKNEYLVVRYPLLMLFSITTILWILIDHILAFSDARWFSKILDYNYSGGSLLFTYTLKVKRAEISVFTQVLDQWWSYSFLDQKQEELKERLQRKLKKFNNFYNRFMVTMGIGFGMMPLGRYFYETEKSSTNLLLERCWSPFPLDTWWGFSITYWVELTSMLSLFYSWAYIVCFLISVMETIGKQLQLIGISLVTVEERVKKAMSLFKVKDKEEWYELYVRMMQKELKNSVKHYQKMYRLSEEACSVFSTPINVCFHSGIIALVASCVKLATERENKFVIFQAIVMVVNVLVNQFIVSCINQILTDQLDSLRDTIYESSWYKLPIPCRKTIHMTQVMLLRPPSIRTVMGTKTDMEFFATVVNTSYFYLGALLSMNVKL

>HhalOR24

RTMGDKAAITDSDVIDGLSIRYLKFFGLWGVINDYRTTEKRNSVLKFQILITLLLMLPSIICQYVGFFVIEMDIQKATLLNFHSLPPLQLCCKHLLFCFRLDSICRLNNLMMKDFLKESIPEYQREKVISIYRKISKQTNIFCLVAFVVITIGVSLICIPEVPVDYILYHTGNMFAITTGRCKISTGWYPLPMDKSPWYEIVLVYECIVVLWASSFVTAFMFLYFQILMSLYAQFVVLGSHISDLNIDSDYQKNSIEYRILNLQNNRELHRILQDHQKLLSYAAELRSVYNPLVTMILGVGMSVLVIAVFQFLFGQTGDLMFIARAFFYVLYQSVEVSMFCFGSLFVQTASSDLQFAIYSSDWYKADVKFRKAAQMMMIRAKKGETLTAIRMYPVNAETIMAILQFTYTVATLMSRLVE

>HhalOR25

LSGAMEAMRDSDVIDGLSIRYLKIFGIWKIINDYRTTGNKNMILKIQVIITVMVTLPSILSQFLALLVIEVDIQKATILNFHALPPLQALSKQLVFWFNIDSICRLYNLMRKDFLEKTVNDIEREKVEFIYRKFSRETNTTCLVAFIVINIAGSFVLLFPGISVDYILYHTGKLSAVTTGRRKISTGWYPLPMDTSPCYEIVVLYEGILVMWCGYAILVFMCLYYQLLKCLHAQFALIVSHVSTLKIDYEYEKNSIEYRFTNQQVYSRMYRILQDHQKLLSYAAELRSVYNPLVTMILGVGMSVLVIAVFQFLFGQTGDLLFMVRAFFYVLYQCIEVSMFCLGSLFVQTASSDLHSAIYSSDWYKADVKFRKAVQMMMIRAKKGETLTAIRMYPVNAETIMAILQFTYTVATLMSRLVE

>HhalOR26

MGNEDAIKDSDVINGLSIRFLKLFGMWNAINEYRSNGKRTAVINVHTFGTLFITVPYVVCQLQSFFMIEFDIQKLTFLYLHPLPASSLCYRILVFWFRMGTLCRLYNLMRNDFFNIPEHVKTGVKELYKKTSKMSNTCCMYVFIWNAGIEILYLFFPGMSVEYIQHHTGSMAEVKTGKKKIFGGWYPVPMSEYPYYEIIFVYESICLLWAATLLAVYFCLFFQLLMCLYGQFVVLGYRFSNLKVDDADRKIRKNNPYNRNNSSRIYEELHQILLEHQKLLRYTDELRSVYNPLVTISLGIGVIVLIIGAVQILMGKTSDPGTIFQIIQVFSLEIMEVSLLCFGSSLIGTASSDLQFSIYCSDWYKADVKFRKAAQMLMVRSRKSSTLTAIVMYPVNLETLGAILQFTYSSTAVTVGMIE

>HhalOR27

MTYGKSIKGLFFAEEGNKEMGFISNIARFVLAGVTMWQSHSRFVIPSALVGFFGVLMLLSNLIHTIYNFDKLNWINKVVDYGWTSVYLTSSYIVVYHKEEIKSLSEELDRWWQYTYLERETAKLKKKSEDWMRTFNSYYVTCGCISWVFYVGLPAVKFFYKTPEQRNEALIFVAWTPFQLDTTWGYFITYGLEVVMMAFAQCIYIQIFQLLMTFAIVMGHQMRLIGTSLLTIFKRIKRMMKNMTFESRIEFYEVRDLLLEKELGNAIMHFQNLYRCSSRLSRIFASITNVTYHGGVWVMCSIAVKAATEMSFVAVLQAFMMIGAVTLSQYVYSFFNECLIEEVQKLRNEVYDSPWYEMPTKSRKTFQIFQSMLDSDRFPTLRTIMGIRTNMENLSKVINASYCYFSMLITIKSSSETTE

>HhalOR28

CLGGTKRLERFVTKRSYSSMGKIWLKNIEEGLKGPRMFLSLAGLGVVFKGRFAFASFVFANNVLALCLLAYSIYYYLDQIEKVSNIVHHAVLIMDICVALVLGYFRHDEFIAILSDPSSSYHYESQMMSKKIEEVKGIACKNYTGMFKYIVLSMVYLHVLMNVLAICQRYFNDEEILLLFPCYFPFPIDNYPVHIAVIIWQELVVSNIGILVFSGLLFIHCIYTHVKSEIDILKFAMVNIEERAYEMAMNQKESRDPTSHPETLSRCQVKCTRMCAEHHSIIISYFHNGGFLIETVYFLVILTGLVVCSCTGFALISENTSLKIKFVGIMIIQIIFLYIMCWLAEETAEQSQSVGDVVYGMEWYRLPKECQTIFLIMMIRAGKPLLMRMLTGQKVDLAAYMALIKASYSYFNMMLATMQ

>HhalOR29

MIDGGIEDSDILEGLNVRYLKIFGLWKVINDYRKTGKRNGIIKFEVFCTFILTLPYVICQYMSYFNIEVDIQKATFLNLYPLPALQMCCRIAVFWFRIDIQCRLYDLIKKNFLNIPEGSRAQIEEIYKRISKVSNMCCMATLIVNASIVSLYVVNPGISVDYILYHTGSMTEVTTGRKKILGGWYPVPMAESPYYEIIFVYEAICILWGGLFLAVYFSLYYHVLVCLYAHFTVLGFQIENLKMKSVKSGGSKSCINRDQNNNSIVYENLIDMIRDHQKLLRYFEELRTVYNPLVTLTLGIGVIVLIIGAIQFLLGKSINPGFIFQISEVFALQGLEVCMFCFGSSSIEAASSGLQFAIYSSDWYKADVKFRKAAQMLMVRANRGVSLTAIRMYPVNVETLMAILQFTYSTATLMMRMTE

>HhalOR30

MGYEIKDSDVIDGLSIRFLKFFGMWKAINDYRTRGKKNIIIKIHLLGSLMITIPYVVFQVQSFFTIQFDIQKITFISLHALPALQMCCRLLLFWFQLNRVCRLYDMIRNNFINIPEDMKDQMRDLYINTNRTLNLNCLVVFIWNIGIVLIFSISPRVPIDYIRYHTGSIAEVKTGRMKILSGWFPFPMDEYPYYEIIYVYEVFCLSWATTLVSVYFCLFYQVLMCLYVQFTVLGASLSNLKIDCPDFRQSKRHKNIIQNRNSNLYEELYECLQDHQKLLRYTDDLRKVYNPLVTVTFGIGILVLFMGALQILLGKTCDLSFLFQLIQMLSSQFLEVSLFCFGSSRIETVSTDLQFAIYSSDWYKADVKFRKAAQMLMVRTRKSSTLTAIVMYPVNLETLGAIAQFTYSAAALLSGMVN

>HhalOR31

WLPIMSLQFETTHSSMIHLLRRSGLKLPWVKHRSYWNQFCYFAYDILLFTIGLYQFICSACSIIFVPTFQDMCTLGIVTSVLATGGSITLFYFFYQNRLEKFTENWNALNDNILNSDLDKKDFFRQMFLQVAKNNESFTKTILFVVFWTPIIYCTPVPVVDAIKQSYRTNLPLPILYLYDDRQPVVYELTFFLHMMGLVISVMKKFGNDCFFLALFKIHIAYLRYLSVAIKCEGTKFSKCNNKIIKQNLISWIKIHQQIVKNAQDLIRLYTPIIIVYHVNLICIVVFGLFTQIKNDRDSSVQRIGTAMFCTVNIFQLYMQCSSAEELTNEAEKVSQEIYNTPWNEVDECNADIIRLVLKMASRPVEVTAFKAPTFLLNKQSFVAFVVNTIRAFMTFSKMSDLRTSISDNNLNLFTESI

>HhalOR32

MAPERVMEKDLMDGLSIFFVKYLGFWGTVNTYRTSGKVNLLFKIQWFLTFLCVPFPIFQFMSPAYIKFDLEKATIIILNTTSFFQMTFKQAVYSMNIKEHAILLEVMTKDILRSLPEYKKPHAKRIFEKISKRCNFWCFIAVVITFTAVSLWTMNPCISSEYIANHVGNMKDVTTGPKKILGGWYPVPFTRSPWEEIVYAYEFLWFFWIGYNVAIYELVITMEVLTLHAQMSVLNYHVSTLSKKEIVQHSGKKGLTQREVEDLFYQELLAIIRDHEMLLGYGNRLRNCFNAYITMLLATGGLLLIASIFQFLFGAKDAVVSVNYMLYLLYEVAEFIFLCTATTMLETSSTNIAFSIYSSEWYTSDKRSRDTIQMIMIRSRKPMSLIAVKMYPVNVETLMSVFQFAYSASALISRMVE

>HhalOR33

MSEPIRESDIFDGQTTFYMKYVGIWKIVNTYRTSGKMSLVFRLEWYLTLLLSVPFQVLQVISPSYIDVDLEKATILILNTVSFLHMVAKHGTFWWNIKGHAELFRLMTKDLLTSIPQYKAAEAKKIYQDATKRCNFYCRMIVTITYSVWSLWTFNPTVKSDYIQFHTGNMKDVTTGPKKILGGWYPLPFSQSPWTEIIYFYEAILLLWCAVIVSIFDTVVTQEVMGLYAHLSVLNFHISTLKKEEIIFHSKREIHTEQEAEDLMHKEFVAIIRDHQYLLRCGKIIKECYNTYITALLLAGGNLMIITVFQFFYGKKDIPTTINFVFYLSYGVMEISLLCWTTTLLETASTNIAFSIYSSDWYTCHKKLRNTGQMMMLRSEKPLSLAGFKVYHVNLETLMNIMQFTYSSSALMSRMVE

>HhalOR34

MALPTVDKKPIDEYYIGKCMKNNYGLEFWLKAGGVFSIGKNGRFIYLISGLYVIAFFMVFPLLVITAYKAAEGKELDIVCEAIHFAIFTFLEMIFLITCIVKNRVLAKIFTIMGKGFYDYENTLDEECLSVAAKTLNESKRRKKIIAKVFIVVVMCACLSITILRPIMKFLLGEHLLGKPDDGILRLIPVNMWTPFNKDSWYAMVIFYLSQDVIGYVTPGLVFGCTLFVVFACEDVGSQLIILGQSLKSVIRRAERLDMPTDEALKLCFSHSIRHHQTILMCVKTLEKLLYIPGLGLLFGSTILMCICGFIFVSKEVPFPSKFVFGMFLLSELMLIFLVCWCGENIQKTSTLIFDMVYSSEWPDNMSSMKNYVLIIQLRTIEPIKISFGGLMDASFETYSNICSSAFSYFNILIAVN

>HhalOR35

MIAVNEAKGSDKPKEWKPVSRSDLLEGLSVIHLEVFSLWTALNTYRKSSKKSYRLLAYIIFTVVVVSPYLISQILCAFYIKMDLQMAIYLILNTMPPYQAFTKMGVFWFRMEEMATLFDLLREDFLTCIPLHKKSKAKEIYRSITKRSNLFCFLAFFANTMTVVTWIAMPGFDTNLEGTGRKKIIAGWYPFPYSETPYYEVVVTYESVLMVWFGLSLCPYECFLVQLLSGLCAHFTVLNHHLATLTKEDVFGKIPENHGGVNAVMNEELKKIFDDYNKLLRYGDILKDVYNVFVTIILGMVMTDLITASLHLLFTPSDALFTVNLILFFLHSLVEIALICFTSSYVERVSFQIRFSAYSSDWYTADKKYRFTAQMMMLRAQRPLTLVAVKMYPVNLETLIAILQFIYTTCAVLSKMK

>HhalOR36

GYQVIEDSDIIDGLSVRYLKIFGIWKVINDYRTSEKRNYILKFQLIITLVLAIPSIIPQYFSFLVIQVDIQKATILNFHSLPSLQVLCKLLVFWFRIDSLCKLYNLMTKDFLDKTIPDCEIESVKHIYTKMSKRTNIIVLTACIVINCGVFLLVLFPSISVDYILYHTGNMYEVTTGRKKISTGWYPLPMDKSPWYEIILVYEGLVVLWAGSFIFVFMCLYYQLLMCLHAQFIVLGSHVSTLKIESLFEELEATDRIQNVEMYKKLYRILQDHQKLISFADELRTVYNPLVTMILGMGISVLIIAVFQFLLGKTGDPMFILRSFMFLLYQCIEVSMFCYSSSFIETASSDLHFAIYSSDWYKAGTKFRKAAQMMMIRTRKGVTLTAIRMYPINLETIMAILQFTYTVATLISRFTE

>HhalOR37

MIKEGIKDSDIIDGLSLRYLKFFGIWRIVNDYRTTGKKNAILKLHLLGTLLLGIPYVIFQFMSYFVIKVDIQKATILNFNAIPALQLCCRMFVFTFCMESQCRLYNVLRKDFLNIPKQNLEVKEIFMSISKTSNFCCTMSLAVNGSIVLFYIIYPGVSVDYILYHTGSMAAVRTGRKKILGGWYPVPIDQSPYYEIVFAYEAIVLLWGGFFLAVYFVLFYQVLMCLYAQFSALGLQMSSLKIEQYRSDINTSLNHNYISSTVYEELYKLLKDHQKLLRYTEELRNVYNPLVTMTLGMGILILIIGAFQFLFAQTGDPVYIFRFLQLLAYQGIEVSLFCIGSSSLQTASSDLQFAIYSSDWYKADTQFRKTAQMLMVRANKGVTLTAIRMYPVNVETLMAILQFTYSVSTLMSRMTE

>HhalOR38

MDYEAVIKDSDVIDGLSIRFLKFFGMWKAINDYRTRGKKNIIIKIHLLGSLMITIPYVVFQVQSFFTIQFDIQKITFISLHALPALQMCCRLLLFWFQLNRVCRLYDMIRNNFINIPEDMKDQIRDLYIRTNRAFNVTCFVVFICNGGIQLIYIMFPKVPIEYLQHHIGSVSAVKTGKKKILSGWYPCPMDEYPYYELIYAYETFGLSWSSILVSLYFCVFYQVLMCLYAQFAVFGARLSNLKIDFGQSKKHKNIIQNNLYEELYECLQDHQKLLRYTDELRKLYNPLVTITLGIGILGLSMGAVQIILGKRCEISFLFQLIQMLSSQFLEVSLFCFGSSCIETMSTDLQFAIYSSDWYKADVKFRKAAQMLMVRSRKSSTLTAIVMYPVNLETLGAIVQFTYSTAALMSGMATN

>HhalOR39

MSHEPFIKDSDVIDGLNIKYLKFFGLWKVINDYRTTGNRNLFIKFKVFLTLFLAVPYVLCQYICYFFIKADLQKATILNLHSLPALHICWKILIFWLRMDSQCRLLGLVRKDFINVPKEKKKAAKEIYEKITKKANLFCVAAFILDSSVIIIAILFPGVSVDYILYHTGNVFDVTTGRKKILAGWYPLPINESPYYEMVLVYEAVLVGWGGMMLAVYDSLVCQSLMSLYAQYKVLGYHVSTLKIDSHSRRTKNGENNDSEMLKELKVILQDHQRLLSYANEMRSVYNPLVTIGLGIGIGVLIIAAVQYLFGKTGDPMFVFKSLQFLASAGLEVSIFCFGSSYLETASSDLHFAIYSSDWYKAGTKFRKAAQMMMIRSKKGVTLTAVRMYPVNLETIMAILQFTYSVATVMSGVTE

>HhalOR40

MSYLPPITESDVIDGLYLRYLKFFGLWGVINDYRTTGKRNRIIKFQLLITLMFAVPYIFTQYLSFFVIKVDIQKATFLNLHTLPAIQICCKIMVIWFRLDSQCRLFNLVRKDFIYIPEYNREAANKIFKEISNKANILCIAAFIVNTAVIISSIAVPGISVDYILYHTGNMDAITSGRKKILGGWYPLQMDESPHYEIVFVYETTFILWAGILLAVYISLFYQVLMCLYAQFSVLCIHVSSLRSDPDAEKKYRNRKVDSEIYKELYIIIRNHQKLLSYANELRSVYNPLVTMILGIGIFVLIIAVFQFLFGSPGNPTFIFKSLLFLAYQGIEVCMFCFGSSYVETASSDLHFAIYSSDWYKADIKFQKAAQMMMIRTRKGVTLTAIRMYNINVETMMSIFQFTYTVSAFMSRMNE

>HhalOR41

LLQHLALDRSTTSMEPPRYVDQYGELFKWQRRCGFSTPWLEKPYFYFRFLDLAYDTITISMVLYILLDYSYTMLTTSLSFQDICLFGIGFGSSACSMFITTYQILYSYRLKEITDKMDSIGKKIMENDLGGKDFFKQEYVKNAKFLSIFTRCSMTSIFTTPFSYFLSVPVVEWFEGNYREHLPLPLANVFDDRQPVVYEIVVIVLSAGISIATAKKAALDSLFISFLSIQTTFLKYLSVAKDEMSKDMRFADDGRSRRKLLTWVKLHQEVIKNIEELVEYFSPIVVVYYIVVIEIVVCGAFVELKKDNDSIVQSISVGSYVMLTVIFYYLLSNKADELTTEVQKMVAAEYNLPWYAMKKSEVSIIKVVLMMCNKPIHITAYQAPVLRLNRETFSQFIVRAISALVTFFQMKDIFG

>HhalOR42

MDPTHFLPKLSTSMEPPRFVPHFRALLRWLTWCGMATPWIKKSFSIWRLLNLLYDLMLMALVTYIVFCYVYTIMTISVGFQDLCGLGVSASNYMCGFFATIHQCLLKDRLKVLTDKLDKIVLDIIRSGLGEEQAFLELYNNNSKTMAVLVNNSVILAAIGTLIYCLSVPAMDWYADQYRSHFPVLIESPFDERVPVAYEIVVFLVAACMIVSIAKKIVTDCLLISLFKIEIAFFKYLSLSLASMKKGFLKGDNAFIDRKLKLWIGIHQSVLRSVDELILISSPMVIMYYVTVISIVVCGTFVQIMKDNENIFQSLSITVFISITLLYYFLLANTADQLTAVAQNVAHAAFDVPWYQMEKKHSTMVRMVIAMANRPIRLTAYRAPIFVLNRENYAGFVVSAISAFVTFCQMKALYG

>HhalOR43

MIYQDAIKDEDIIDGLDVRYLKFFGLWKIINDYRTSGKRNVVVKLHLLLTILVSVPYVFLQYMSYFFIDVDMQKATYLNVNTLPAIQACCKVLVLWTRLGSICKLSGLMKKGFIEISEENKAAASEICKKITYKSNILYKTALCMNAGSAVVYLLSPGISVDYILYHTGNMAAVTTGRKKHLAAWYPLPFDNSPYFEIVFAFEALLISWDAIILVVYICMYYQILTCLYVQFTLLGLQMSSLKNENIKKKDNIIKQANRTTYDKLYRALESHKELLSYTNELKTVYNPLVTMILGIGIFVLIMSVFQFLFSKTGDIMVIIKSLQFLGFHGLEVSMFCFGSSAIESASSELDFAIYSSYWYEGDNRFKMAVQMVTMRAKKGMTLTAIRMLPINLETMVSIFKFTYSTAAFMSSVTE

>HhalOR44

MEPPRFVDYYSGLIKCLMYWGMPMPWWVQKPSKTVWLLLVTYDTLTLAVTCFALYLYVYTVTLDDVEFEEVNVLPPAICFKLCVLGISLLQFLDRGRIKQLSDDLDAVVRTIIETDLDQDESVKESFIQMYSKKSKFLVNYCRTMPFFAVSYYVIYFGSVPLIDWSEGIYRSHYPLPLLTPFDGRKPGIYEFLVFVVLISLTMVSGKQINNTCIFLAFFNVLRSFLHYLSLTMSEMQKISYKERNDPSIRRKIRVWIQIHQEVNRNLQVLLKIFSPVVIIYSIYLLLYLITAIFMQMQKKEENIYQTAAEGLAVIGMVRQIYVIFNTADQITTEAQKLANAAYELPWYQMDNSMRSTINMIMMMCNRPVHITGYRAKSFIVNRETIAGLMTSAVSGYLTLCQMTDAFGPKESSRQ

>HhalOR45

MDNQGAILDSDIIDGLDMKYLKFFGLWKIINDYRTTRKKNAILKFKVITTLLLTIPYIVSQYLSYWMIEVDIQKATFLNLHSLPALQICCKVLVLWFRIDSQSRLFDLVKKDFFGIPKSKEDEAKSIFSKMTSECNKLCSAAFLINTSVVILSIIDPGISVDYIMYHTGNMHAVTSGKKKILGGWYPLPIDKSPYYEAVFVYEILLIIWGGILLAVYVCLFYQVLMCLYAQFSVLALQVSTLKYSYIQDGKGRKSVNSKLYKELYEVIKEHQKILRYAEELRSVYNPLVTMILGVGIFVLIIAVFQFLFGSTGNPMFIFRSLQFLAYQGIEVSMFCFGSSYIQNASSDLHFAIYSSDWYKADVKFRKAAQMMMIRAKKGETLTAIGMYPVNRETLMTILQFTYTTSTVLSRITE

>HhalOR46

MQYQQPLRGPDVIDGLSIWYLKLFGFWKIINDFRTTGKRNLFFKFEFIMSILISFPYIACQFSSYLTIDVDIQKATLINFYCLPAVTMCSRILVFWFHADSQCRLFNLIKKDFLCIPENKKAETRKIYRRVSKSCNMMCMFAFVLDLSVVFTTVGIPGIPVDYILYHTGSMFDVTTGRKKILCAWYPLPMAEYPYYEIIFVYEMMCVLLGGIYLPIYASLFYQVAVALHAQFLVLGYHVSTLKINPNIKQKKKNMSSGITEDLYKILLDHQKLLSYADELRSVYNPLVTINLGGAIGILIVSVFQSHMGETRDIVFVLKSILYAASIMIELLMFCYSSSLIQAASSDLHFAIYSSDWYKADTKFRNTAQMMMVRAKKGVNLTAIRMYPVNLETLMSIFQFAYSTSALMSGMLEE

>HhalOR47

QQGIADSDVLDGLSIRYLKFFGLWSVINEYRTTGKRNGIIKLKLFITLLLSIPYIFSQYLSYFIIEVDLQKATFLNLHSLPALQICSKVLVFWFRIDNVCKLYNLIRKDFLSLPEHKRDGAKCIYMKITKTSNMLCKAAFIVNSSIVALYVMQPGISVDYILHHTGNMAAVKGGRQKIMHGWYPLPIDRSPYYEAIFVYETMLIIWDGILLAVYDSLFYQLLMCLYAQFTVLGFHLSTLKIVASQDPNSRLNDSNSPIYRELYKIIKDHKKLISYANELRSIYNPLVTIILGMGIFVLIIAVFQFLFGGTRSPLFIFRSLLFLVYQGIEVSMFCFGSSSIEKASSDLQFAIYSSDWYKADIKFRKTAQMMMMRARKGVTLTALRMYPVNVETIMSILQFTYSVTALMSRKAEIK

>HhalOR48

MSYPAEIRVSDVIQGLDIRFLIRSGMMRFINDYRTTGKMNPMIKIHLIGTFIISLPYMVFQCLSLFKVQYDIKKGTFVILHPMAAFQIYCRILVLWFNIERQGKLYNIIRKDFLNIPKEMSHDASELYKKQNRTSNLCCNATFLWNASIELVYIFFPGVSVDYIENREINKKVVNTGKNKIFSGWYPVPMSEYPYYEIIYIYEAMCLLWASTLLGLYFCMYFQLLMCLCTQYVALGYRVANLKIDPVIYKLDKKYKSSIYQELCQIVKDHQKLLSYTDELTSVYNPLVTMTLGIGIAVLIIGAIQFLLGKTSDPEIIFKLIQMFSFRTFFEVSMFCFGSSRIEEASSDLQDAIYSSDWYKADSNFKIGAQMMMIRAKKRVNLTALFLYPVNLATLGSIVQFTYSCSALMSGMAE

>HhalOR49

MFSWCSRKNASLSENCDEKSEKYHIKKAFEENYGFWMIFGGFYPCVGILPCIYIPSAVFLSIIFIMTTVNISNSEIAIKSESVHFIVFITIELTAMTVFIFKKKTIDEIYRAMGRRFYDYENTLDEECYDVIANAYKSGRSRKKIFHDMFVGCSMSTLLTASIVRPLLSYFKGDPDPNDGILRVLPVPLWTPFKTKTWYVNLLFFLAEDVIAYMTPGIVFGCILFVVCASEDVGAQLIILGHTLKSVVRRAEGLDMPREEALKLCFVHSIKHHQMLLKYIKSLEAIIYLPGFVLLFGSTILMCMSGFIFVSKEVAFISKVEFFLFLLSEMAVIFLICWCGEFIQTTSTQIFDMVYSSEWPDNMESMKNFVLIIQLRSIDSIKLNLGGFMVASLETFGNICSSAFSYFNLMLAVN

>HhalOR50

MSLSSVFGNMLQIFLNEPSDPGKGQVYEDSRSFRRGLAIWKSKTRIVIPSLLILFMEFIMFLSVIMDAVTRPNLDWVDKIAEFCFIINTLIFYSITYFLRDDMDRLSESVDFWWSHTFLKQTREDLKKSSIQWMETFNNYFMSLLALTCTGYAVLPLAIFAISESKSYSELNIFKMWAPFLQQHLWTILLLYAFQVACIFFQFFGYGVLTAYMMSVSVACKYQTRLVEISCLTIKDRVLALVRKEQDISERKTLFCTIFLKEIAESAKHYQHVYRNWKEMCRLLSRMADVVFYSGMCIVIMFGVRVATQQENSSVILNSFLFIVVVICNQYLFTIINGTFTNQVISIQNSVYNCPWYTLPVSCQKSINLFQIMVSYIPTLSTFMGVEASREFFGRTINATYCYMSALVSMNRK

>HhalOR51

MNQQAPITDSEAIEGLNIKYLKFFGLWKIINDYRITGKRNIMLKVQLVVGLMFTVPYVVFQYISFFFIDVDVEKMAFLTLHTLPGTQMCCEILLIYFRIDSECRLFNLIKKDFIYIPENKRDLAERILTKIGKNSNILCIGVFLVNLITLIFAVNFPVASVDYILYHTGNMDAVTTGRKKIFGGWYPLPMDKTPYYEIIYFFEAAFHLWAGMLLAAYISLFYQVLMCLYAQFSILSLRLSSLKVEKENSQDRNGDSKIYKELYMIIKEHKKLLSYSNELRSVYNPLVTMVLGIGIIILIASVFRSLFGTFGNPISMFLSVMFIAYQGIEVCMFCFGSSYVETASSDLQFAIYSSDWYKADIKFRKAAQMMMIRARKGVTLTAIRMYPINLETMMSIFQFTYTVSTLMSRMME

>HhalOR52

MSDHTPIKDSEIIDGLNIWYLKLFGLWKVINDYRTTGKKNSIINLQLTITLAYSIPYILCQLMSCYFIKMDLKKITFIFLLTLSKIQICIKVLVVWFFLRIQCRLSNLMKKDFIDLPEHKKSEAKQIFKKIALQTNLLSIAAFIINTSYYIVSIGFPDTPVDYILHHTGNTFRATTGRMKIVGGWYPIPFDKTPYFEIIFFYEASMMMWVGTFLAVYVSLFYQTLMCLYAQFAVLGIKLTTLDSGDGKEKIWKSDSERYNELLAIIKYHQKLLRYADELRLVYNPLVTMILGTGVFVLILAAFQFLFGTTTSTIFIVKSLIFLPYQAIEVCMFCFASSYLETASSDVLFAIYNSDWYKADIKFRKSAQMMMIRAKKGETLKSVSIYPINVETMMSIFQFTYTVSALMLKTTE

>HhalOR53

VEMELEEGMYRSGFNRIVSFVTCMRGPLENPGKGLWYGKLYIAYITYIEFAVIVNWVTIGTVFLKPGMTVEVRCFTGFPVVASAFCCIRRVDMFLNRQRYKKLMEDYLSLYDNSPELNKDVLHYAKIIGNIPRILFFITALPMIIMGVIPILVAIVGGVRVIPMLAVFPFDPTEYFFVFCATVLLQITGGYTCTLRALCFENMFNMFACRQLALIRQLSRELRRILKIPHVDDSGELKYQNDEGILYSPEEAKNVVIEELKQWVKNHQKSMRMAKELQDMYSISLCIQFAFTGLLLCTTAFVMANKVGGMMNLFFCGAYLIGLFIELLITCRIGDLILYESNMLERTVEGTHVYVLPSDVYKNWLRLILTRAKVPTRLSALGVFPLDMETYKSFIVLTYSFFTLLKELKHET

>HhalOR54

MEAMRDSDVIDGLSIRYLKIFGIWKIINDYRTTGNKNMILKIQVIVTVMLTVPSILSQCLALLVIEVDIQKATILNFHALPALHALARHLVFWFNIDSICRLYNLMRKDFLEKTVNDIEREKVDFIFRKFSKETNRTCFLVFMVINVAGSFILLCPGISIDYILYHTGSMSAVTTGRKKLSTGWYPLPMDTSPCYEIVVLYEGILVTWTASSIIVFMCLYYQLLMCLHAQFAMTVSHVSTLKIDYEYEKNNIESSFTNHQVYSRLYRILQDHQKLLSYAAELRSVYNPLVTMILGVGMSVLVIGVFQFLFGQTGDLVFVGRAFLYVLYQSVEVSMFCFGSLFVETASSDLQFAIYSSDWYKADIKFRKAAQMMMIRARKGVTLTAIRMYPINLETMMSIFQFTYTVSTLMS

>HhalOR55

MGCLGPIADSDIIDGLSIRYLKFFGLWKVINDYRTTGKKNSIMRFTVIISFILAVPYVLFQYLSYSSIKVDLQKATFLNLYPLPALQMICRILVFWFRMDRQCRLYNLLKKDFLHIPENKRVLVDKVYQKICKTSNICCTASMIVNFSIIGLYIFNPGISVDYILYHTGNMDAVTTGRKKILGGWYPLPMAQTPYYEIIFVYEATCVSWAGILLAVYFCLFFQVLISLYAQFTVLGVHISTLKFQSNKKDRKCDTKMFKELSQILRDHQKLLRYTDELKSVYNPLVTLTLGMGILILIIGAIQFLLGKSNSPGFIFKLLQVFIFQGVEVSMFCFGSSFIEMASSDLHFTIYSSDWYMAGTKFRKAAQMMMIRSKKGETLTAIGMYPVNRETLMTILQFTYTTSTVLSRITE

>HhalOR56

MFGPSQMNVFFEERPHNHEGLTSWKLRRAVSYFIAFVYFPTFCLIETTGILFGRNSELEEVIFAIAYISFMVQMVIKLSYFHWKIEDFRELCLQFEYFHTSRHRPDFAKGYLEEASDSLRRTAKVYNFVIYVNLILWNLNPLVVQPVRFVLNLTGFTDDKDATLIPDIFPVVYMFDETKTWYLRTLTGCLEWIVLNAGLCHAVALELFFMSLFLMLAAEVEVINKSAQSTEELGREIERDYSTLLGPRDDLDRIDITLLIEDHRIVLKKINKVSDIMNPLLGLAIGYCMVVLPTLGVVITKAIRDAKSTTEAFLNIITWIGATITELLVLFMYSWICAKLKDSEEGISEAVYSTNWYERDIKYKKTVLFIMMKSLRPKKMRMLYCGDMDRETFVVGLKGIYSFYNFMIGLA

>HhalOR57

MSYQDVIEDSDIIDGLSVRYLKLFGLWKVINDYRTTGKRNKIIKITVLVSFILIVPYITFQCISYFKIKVDIQKATFLNLNSMFALQLCCRLLVFWYRMGSQCRLVNLLKKDFLNIPTQKRKAVDEMYKKISKTSNICCTTYMISNVIIVVLLIAKPDTSVDYILYHTGNMDAITTGRKKILGGWYPFPMNESPYYELIFIYESICSIWTGLLLVVYFCLFFQVLFSLYAQFMVLGLHIGTLKIEDQDQVHKNDIQMYETFHGILKDHQNLLRYSEELISVYNPLVTMTLGMGVLVLIIGALQFLLEKASPTFIIKILQVLMFQGVEVCLFCFGSSFIETASSDLQFATYSSEWYEASKRLKTSVQMMMIAAVRGVRLTAIRMYPVNRETLMAILQFTYSTSTVMSRMTE

>HhalOR58

MFSYSHLNVFLDEHPKGDVYFWKLKRYVTYFFLFLYFPIWLVIEVSGMFLGRKGDLAQVAFDLSYVAHIVQLVIKMGYFLYYIEDIRCLCLRFERFHTSKHRPIFSRRLLGERGQFLRRLASTYYTVIYMNFLFWIITPLFIQPIIYGLTQAGFIAPAGPQNIIPKFFPVRYPFDETSTRNRIIIACMEFTVLSAGFTYFIPIDQFFVSVIVMVCSEIDVICKSVMTSKELTKELDRDYSLLLGSEDTRDRIDLKLFIEDHQRALRTTKKIGEVMNPILGLVVGNCMVLLCTLALVITTKMKTASSFSEVFREVFGFIVVMTTSLITLYLYSWMCGELKSSEEAVFGAVYSSDWYNRNKDYRDNVLIVMRQSYTSRPLRMMSMGDMDKETFIKGLKGIYTYYNFLTHFE

>HhalOR59

MSDQAIKDSDVLDGLNVKYLKFFGLWRVVNDFRTTGKRNKILRVKIFITLVLVLPYILCQYLSYFVIKVDIQKAIFLNLHLLPGTQICCKIVVFWFKIESQCKLFDLLKKDFLSVPEEMRPEAAEIFKKITRRTNKLCLAAFIVNISIIISSIADPAISVDYILYHTGDMAAVTTGKKKMLGGWYPVPMAETPYYELIYVYEAVAGTLGGFLLAMYVCLFYQVLMCLYAQFTILCLKTSALKIKSDNGRINSSIYKELNEILKEHQKLLSYAKELRSVYNPLVTLIIGIGLFILIIAIFQFLFGGKSDFMFIFKSLQLLVYQCVEVSMFCFGSTYIETASSDLQFAIYSSDWYMTGMKFRKEAQMMMIRATKGETLTALRVYPINVETIMSILHFTYSASAVVSRMAE

>HhalOR60

MLKLNDYADPKDEKYFKTGFKKNYGIWLLYGGMFLGNPLLPVAFILSGFILIYFMLGTITKFYKTDLLCAIENLHFLIFVTVELVAMMSFLQKRAVLVSMYITIGKGFFDYENTLDDECLELKRDAYDKTDSRKRLVHHSFVTVVMSACITISVFRPAVLILFPKENMGNPNDGMIRVALAPMWSPFDNTQWHGIVIVWLLEYIVAWTTPGIVFGATFFVLFSLEELGIQLQILKKSLTNVIQRAERLEQGMEENIKLCLKYSVRHHQLLFEFHDKLNEVISLPGLGLFVSFSIMLCMSGFIFTLKEVPLVSKSVFGLFLLSECAMLFALCYFGENIIELSEEIGDALYNSDWAIYSKVMQNYMLIIQMRSRRTMRLTLMDFMDVSRNTFSNICSTSYSYLNLMNEFN

>HhalOR61

ESNLFQHSGSDRVKLLTMVKIIKELLGDLPRILVKTFLISRDSKKRTGQTFVTISNAIMTLVCISSMYFLGLEKSLEGTASFSAYAFIIATKHVIYYFRQDEIKRMLNIFARLQKEYKEKWEREIFKRDYEDTWNMVYKFCLIMISYQVFYLMFTISVDYIIGNIFPNFPSVRVHLPCDGFIEFFEPRTLGRFIITIPVLMWTAEAITIHVGSETLVFVLMMYTKSELKMIRYRLIIIKKQLNRNKSNRSVNAEMLLWEVIQRHQRALDVLDVMKDTLGLPLAIHYTSVSVTLSVIFYCLMTFDERGTLTAKFNGITAIICIGSLLFALCYFGESLEEENNEINKRIYDLPWYNESIFVKRAIIIMLRQTQKPFVINYRLTAQLSLQTFMQVINTAYSYLMVLKSTVR

>HhalOR62

MAKLNGYTDPEDEKYFKIGFQKNYGNWLIYGGMFLGSPVLPITFLFCATTLIYFMFGTAIKFYKTDLVTTIENIHFLIFVAVEIGAMIVFLRKRALIVSIFITIGKGFFDYGNTLDDECLELKRDSYHKTDSMKRLIHHTFVTVVMSACIAITICRPVIIMMFPKENEGSPDDGMVKVALLPAWTPFDKSRWYTTVILWFIEYAVSWTTPGIVFGATFFVLFALEELGIQLLILKKGLNNVIQRAERLEQTMEENIRLCLKYSIRHHQVLFEFRDLLNEVVSLPGLGLFVSFSIMLCMSGFIFTLNEVPLVSKFVFGLFLLSECAMLFALCYFGEKIIELSEEIGDALYNSDWINYSQVMKNYMLIIQIRSRCTMRLSLMGFMDVSRNTFSNISSTSYSYLNLMNEFN

>HhalOR63

MEPRRPLQEASILEGLATMHMKFYGLWYCLNVTRTTGKVSRLFILVCALIVIIASTYVMFQIIYMFTIHLDLQKIAFVYLIVAPCMQDFYKVIFFLAKMQEICLIYDTLLVDFLESIPKHKMPVVKEIYRRTAKKCNQVCSFAFSGLIIAGSIWLFVPGYDTDDPTSDRKKVLNGWYPFHYSESPRYELVYAYECIMTLWCGGWYCIFECAILMPLICLCGHFDVLSYHIATLKKSDMVHVLGRSSASHLESNAFLNDQLKYILKDYEKLIRYGDTIRETYNLVITIILGVEIGNLTVIVLHLIFEDKDAMFLVKTGTYMSFQLIEVILICFSSDMMGEASSGIREALYCNEWYTTDRKLATSQQLMMVRASVPLTLTAVKMYPVNLETLLSIFQFIYSTAALLSKMK

>HhalOR64

MDPPRFTDYYRWMLKSFLYWGMPTPCLPKLSNPVWLLLVVYDFLTLAVILFALFVYGFTMARGNIGFQDVTLFLPGFVLYSFALYLSLYQFLIKGRLEKIIAEMDALARDIIESKLGDEEFLQVYSDNSKNIINHCQTLPFLYVSISFIFFCSVPIVDWYEGKYRTNFAIRIETPFDYRQPGIYELVVLLMSLALSISTSKQLNNALLFLAFFNTLRSYLKYLYISMGELKRKTIKNDNINNAFSRQDIRTWIKIHQEINRCLQVLLQLFSPVVIVYCLYMMFFLVSALFLQMQEKRNSIYQTFSAFIGVMVMLIQFYMVFNIADHVTFEAEDLANAVYGLPWYEMDKRTKYEVQMIITMCNRPINITGYRTKSLILNRETLAGILTSAMSAYLTLCQMKDAFGPKE

>HhalOR65

MADLDDIIVDERKTRKFLRLVCCLRYTQGKNSISTFNLLYIISVYTLLLIVVVQGVVLAYSTDDIVQKVESIHYVLTIIIVISYMSNELYNNSNLDQAWKLINLAYNEFSKDRDENRQIHVEINVSSVKTNKLFCFLMSSSCVGYLLFAPMRQLFTEEDSSARKLPVPLYMPFDTSDNFGFSLGMAWEAISLFYICGVSTSIHQSFRGIMGRLRGELKLLNNSIKGIHVRAEKYQGKVDDTEKLEAHFQFLVYKCLREDIVHHQMLLEYYSLTKIYLGTILLLFIFLSSIILGAVGFLITKPNSNTEDIIKFLAIVTAELFFVYQLCWEGERVAEESGQIFNNLFNIPWNAYDRNVKGCIRIMLCGTIKPIRLKTSIFNVEASLETYNWVITTAFQYFNLLRNIKSG

>HhalOR66

WLHIMSLQFETTHSFLIRVLRRSGIKMPWVHLDSNLSTFLYSVYDSFLVFIGFYQFIFAAGSIVWVGNFQDMCNLVAIAVTLLTGILISLFYLLYQERLALFVENWNKLNIKILKSRLDRGFKMMYLKIAQSNVSFSKKILFLVFWTPFIYCSPVPIIDVIRGLYRANLPLPILYPYDDHQPGLYELTFCLHVSGLVISVMKKIGNDCFFLSLFKIHTVYLRCLSTSINDSKDKFNMKSDLFIKKKQIVWIQLHTEIMRNANELVSIYTPIIVIYYFNLIMIVVFGLFTQIKNDRDNFIQGFGIGGFGIINIFQLYMMCSSAEVLATESERVALAIYDIRWYQMNRSNGEMLRLMLFMAKNPVQVTAFRARTFLLNKESFIGFITSSLTALMTFTKINDIRQSQSSS

>HhalOR67

MVYLQRLQKSDLFDGLNIGHLKFYGLWNGINDYRSTRKTSCIFKFNMTVSALYVFPFVVFQFICIFIISVDLKMATFVYMNGVSAAQVLFKIIVFWYRFKDQCDLVDLLRVDFLSSIPDSKTRHVNEIYKKNSLRCNIFTILAFTGNVLTIITWTILPGFNTEKTGTGRKKILSGWYPVTYSESPWYEIVFVYEVILICWHGSLVSLYESFLLMLLVGLYSHFVVLGYHLSTLKKNDKAVVKAGVDTKIDEAFNIELKKIMQDYNKLLRYSTLLRTTYNAITTVTLGLDIGVLILTIMFLMFGSSDGLSTFKMMMYFSFALIEITLLCVTSSIVGSASMSIHDSAYSSDWYVADKKFATTAQMIMIRSMIPVSLTALKMYPVNMETMIGIIRFIYSAVAIVSKMKE

>HhalOR68

MDTKNQAKRHNNGPGLVEETSQLFLNGLRRYKSESPLSIPIPLIVAVGNIMVCLMVTGGIVNYTKTDWVQLCINLIFIPVSVMVGTMILKYKRSFSWVNKEIDSWWSYKLLGKETDVMKKNAAEWMAWYGQYYYIGMYLAWVMNLVPFIKCHIWGKSEDPMNNLMYPCWTPLDLGTWWGFVLTYMFQFMSMYLSYFTFGNTCFYLTTAYISIGYQAQLIGLAILSVEQRCRKLSDENKDSSKREEIYESYVKEEIKSCIKHYQQLQRSAKELSNIFSTLAVLSYNIGIVVLSISGIRLTTETDKLMLVNSFVNTCVTLGNQFIISFICELLTEEVEKLRKIIYYSSWVSMPVSCQKYIYTFLMMVDYMPTPVTLTGLKTNYENFSKVVNSAYCYFSLILSMRNK

>HhalOR69

MDPPRFVDHYRALYIGMRTFGMATPWIQKPRSLWRRLPFLLWDTLLVVIVVYMLFCYAYSIMTISIPFQDLFGLGISTTNYICALSVTFYHALYGPRLKKITDNMDSIAAVICANNLGGAERFHKRYATNSKLMSVFTTLSIHFSAYLPFTYFFEVPVTDYFTGKYRSRFPVKINSPFYDREPGVYELVLGIMSLCVSSSIGKKTATDCLFMTLFMIQRNFLYFLSDSMKDLEKEFLTGDNKLFKKKLSIWIKLHQDIRRNAEELVLTFSPVIIIYYVSVIGNIVCAAFIQMKKDNDSMFQSICMSIYVMVALTYLFLLSDAAGRLTVEAEKLAFVVYSSPWYESNKTNTDTLRLVVTISNKPIHVTAYKAPVFLLNRETFLTFVASALSAFVTFSQMKDRFDQ

>HhalOR70

MDAPKFVDHYRSLLRGLRRVGLATPWIEKPTLLSRVPFLLYDGLLVAIVVYMLCCYGYSITTITIPFQELCGLGVSTSNFICALLVTFYQAHYSQDLKRITDNMDRIAERILTSDLRGADHFLQLYKRTSKLMAILTDYSIFFSFTLPIVYCFPVPVMDWMEGHYRSRHPVRIANPFNDKIPGVYELIAIVVACSIAFSTSKKAAMDCLFVTFFSIQSDFLKYLSVAMSELQKELRDEDSPLTRNKLITWFRLHQDIIRNTNDLIETISPVIITYYMTTIGIVVCGAFVQSMKENELFIQSISIGGYIMITLIYYFLLSNTADELTTEAQKLAFVVYSTPWYDMKKRNADMVQLVVTISTRPIEVTAYRAPTFLLNRETYAKFLVGAISAFITLCQMKFLYDDS

>HhalOR71

QLEGNHFSLHQRMKEVDIDSRYFRAIGLWQFVVDYKATYWVLFNFALASVFFVNISVQLMNTLTGGYEFSLLTEKLSVNLTVMESVIKIIYYCAKRRKLYSLSLCFRRDFLICRNHDREVADEVLNTGFSSVNTVTKGFVVMIFTTVGLWNSFPFLRCLTGDCSKWNIMPSWYPEAMDGLPAFIYIFEFFIMVFCAALLYNVNCFFSALALSLSSQFQLLTKSFSSIETNAERRKGCKIYNMNVLLRECLIDHQRLLRLVKEMEDMYNPIFLFQMLTSTFTICLVLVQLNDRTSSKGEMPVAMVCKFFMYLMFGSMELLVYSWGGQIIYDQTGEVHRSLYESGWASASHYFRKSVLIAQIRTLRPEYLTAGKFYAVDLASFTQIIKASYSYFTFLHGSGGSSR

>HhalOR72

HMPAPKFVDHYRALLTGLRRVGLATPWIEMPSLLGRLPYLFYNSLLVSIVIYMLCCYVYSVTTITIPFQELCGLGVSTSNFTCALMLTFYQISYSEELKRITDNMDRIAERILSGELAGAEHFLKLYKRTSRLMAILTNQSIFFSFALPLVYSFPVPLMDWMEGHYRSRHPFRIASPFDDKLPGVYELIMLIMTCSISYSTSKKAAMDCLFVTLFTIQSDFLKYLSVAMTELQKELKIGNSTQVRNKFVLWFRLHQDITRNTEELVEAFSPVVIIYYMTTIGIVVCGAFVQSMKENEMVVQSICMACYILITLVYYFLLSNTADELTTEAQDLAFIVYSTPWYDMTKSYADMMRLVITISTRRIEVTAYRAPVFLLNRETFAKFIVTAISAFVTLCQMKMVYG

>HhalOR73

MDPPRFADHYRPLLGWLRTCGLSTPWGEKPSFALGVIITCYNTLLVIMVCYTLLLYFYSILKNSIPFDELCLLGVGFGLYGCNVAVTLYLFLFQGKLKVIINKIDSIAATIQQNEIGGSEFLQEMYKKHAKLMVVLANNSIYFGYLTPMIFCLSVPTMDWYAGNYRANLPIQIDAPYDYHIPLVYELMVLLLSCCLAVSTTKKAATDCLFMSLFNIQITFLKYLSITKSYIQDDFKSTNKTFSKRKLIIWIRLHQQVNLNIQQLVSIFSPLVILYSVGTIMIVVCGTFVQIMDDNNNLMQSISIGIYVAMTCLNQFLMSRTADELTQEAMKLAFFAYDLPWYEMKRADADMVRMVILRSNRPIQVTAYCAPIFMLNRETFRGFMVTSISAFVTFCQIKDRYG

>HhalOR74

MDPPRFIDHYRGLLKWLRICGLASSWHHQSNFFSRFSFFIYATFLVIMVSYVILCYIYTAMMTSLTITDVCTFGVSGGCYVCGLLVSSYLIRYKDRLKKITDEMDIITKKIIESELGEREFLLNEYNKNSKLMAVLTDGSLYLSFATPIFYCVSTPVLEWYNGMYRSNLPIQTLSFFDEKAPGCYELMVIFTACSIAISTSKKSANDCLFIALFRIQTIFIKYLSVSKGALEKKLLADDSLRGQRKLLAWIKLHQDIMKNVEELIIYFSPVVIIYYVMVVEIVVCGAFVQLEKDNDNIIQSISVGSYVATTVLYHFLLANTVDELSDEAQKLAFVEYCLPWDKMNKKNISMVKFVLTMCNKPIKVTAYRAPIFLLNRETFAGFLLSAISAFVTFSGMKHNGS

>HhalOR75

MDPPRFIHHYRPLIGWLRKCGLPNPWAETPSTPRRLLLFCYDSFLVIMVSYMISVYIYSIASISISFADLCSLGTSGCCFMGSLFITLYMALFRKRIKMVTEGMDSIAEVIFQNELGGARLLQEMYQKNAKLMAILTSNSICISFLAPCTYCWSVPFVAWLSGTYRDELPLPLDTSYNYRLPVIYELMVMLISCSIGISTSKKSAVDCFFISLFNIQIDFLKYLSVSKRYLQEEFNSGKDIYIRKKLIMWIKLHQDINMNIQQLLTVFSPVIIVYNLTMVIIVVCGAFVQIKSDSNNLIQSLSIGAYVAIAGLYYYLMAHTAEELTTQAENLAFSAYDLQWYSLKKADIDMLRMVILRANVPIQVTAYCAPRFILKRETFANFVVTSISAFVTFCQIKDRYR

>HhalOR76

DPPQFVNHYRTLIRGLRLSGLPTPWIEKPSKLLRIPYLLYDLLMLTIALYMLWCYLYTTIQGKLAFDELCALGVGISLYTCSLLVTFYHFLFGHRLQNITENTDRLAAALLRSGLNRGHSLHQLYLKDSSAIAVLTKNTILFSFFCPVLYCLSVPVMNSIAGRYRCEQPLPITSPFDDKKAGIYELVALVLAISNAISCSKKGVNDCLFLALFRIQSSFLQYLSTSLENLQKEFLIESNDWNRKILIQWIGLHQEVLRNIQELVRIFSPVVIIYYLNVIGIVVCGLFTQTMNDSGNVMQSIGVGSYVLVTVLLMLLLSNTAEDLCTKAQRIAFVAYDVPWFEMDKTNADMLQMVINNSSKDIHVTAYRAPIFLLNRQTFAAFIINCIKAFVTLVQMKRCFG

>HhalOR77

MDPPRFIDHYRPVLGWLLKCGLSTPWAEKPSLCRRLLLLCYNAVLVIMVCYMLFLHLYSLTQISSPFPDLFHFGISGTLYGCSLSITVYLALCRERMKTITDGLDSIAETIFRNELGGADFLEQLYKKNAKVMTVLTNNSLFISFITPVTFCWSVPFISWLAGKYRSRLPIPIESPYNYRVPIIYELMVVLMSSCLIIASSKKAVADCLFMSVFNAQITILKYLYVTKRYLQVGYDNDIMVDRKKLVLWIKLHQKINENIRQLIETFSPLLIIYSVGVITIVVSGASVLVMNDNNNLIQPISIGTFIAITVLYYYLLSNTAEELSTEAQKLAFMAYNLPWYQMKKADADIVRMVILKTNRPIHVTAYCAPVFLLNRETFNAFMVTTISAFLTLSQIKDRFD

>HhalOR78

MDPPRFIDHYRVLISCIRRSGLPTPWLEKPHSISRVLLLIYDSIIIIMLLYSVVCYMYSIMTSTISFQDLCSLGLSGGCFVCALLINFYQIQYRKELKHITDTMDSIAEKIIESGLADKDIFVKEYTTNAKFTNNLIKYTLMSMMTTPFIYFLSLPIFEWYAGAYKAHFPVPIENFFNDRLPGVYELIVITIAASISYSSAKKASNDCLFICLFKIQTTFLRYMTLSKEVIEKELLAGNKNSQRKLLIWVKLHQEIIKNTKELILIFSPVLIVYYVMQIEIVVCGAFVEIKKDNDNLIQSISVGSYVALSIIYYYLLSNTADELTTEAQKLVFMEYNLPWYEMNKRNASMVKMIMTMCNAPVEITAYRGPTFLLNRENFAGFMFATLSAFLTLCQMKDIYG

>HhalOR79

MENQKRRMAKINLDIIAEATRKLRIIIGLSNIMESYKLFSLITFINSIGGCCLLIYSFYHFRHSSDDLAGVAYNFGLLNGVVVGSVSGHLGKIKLFKIVDDSYFTYDYQSSIMKKKIEELEKPRAKALKRFFKTLLILTYAAQANLTGLTVLIGMIKKEEIYLFPCWHPFDLSNILSQIFLLAWQQFIILGITFVAFGCWGILYITYSHIKTEISLLDFAIRNISSRAREMMQHRESVEKGEDYDKILLACYKQCTRMCAEHHSEIIRFFTNGQDFIGIFYTTAFISGGSACTFGGYYISSENIELQFKYLAMTTFILTYLYILFWIAEAISGKFFTIAQTVYTLEWYNLPKECQSTLRLMQIRSNHPLFYKLVLGQRVDMEAYMSLVKATYYYLNIIIT

>HhalOR80

MSLQFVTAQRTVIQGLRNTGISLPWVEDDSLLTNALRKFYNFFAFFIVTFQLVDTAISAIMLENFDDKCRAGTVTSIESNPTMLSVYYVFYHVRIKKFLEHSDALSQRILNSELGQKEFFEKSYLENAKKNNFFTKIVLIFVFWTPILYALPRPLIDLYNQEYRKTLPMYLVYPFDDHKPGFYEMTFLIQTLGLVCGDLKKFANDCFFLSLFRTQTVYVKYMSASIRDLGEEFKKTGDLNLKKKLIKWIEIHDHFIRNFNELLSLYTPVICIYYANLICTVVLCIFTQLQEKNFGIIEGIGLGGFFSANVFQLYLQCAANDDFIVEADNLALEIYKTPWYEIDKTNKDIIRTMFLMASRPVEITAFKSPTLRLNKEAFLAFVASTITAVMTFKNMSDLHQ

>HhalOR81

MSLQFETTHSFLIRVMRRSGLMVPWVRMDSSLLAAFYFFYDNLLVLIGFYQFIFSVGSIVRIGNFQDMCTLGIVIAVLATGVMLSIFYIHYQQRLEKFINNWDNLNTKILNGEPQLAKYFQRLYLEITKSNETFAKRILFIAFWTPFIYCSPVPIIDVIRGSYRTNLPLPILFPYDDRQPGLYEFTFCLHVFGLLISVMKKIGNDCFFLGLFKIHSVYLRYLSESIKDAKKKFANNDWFIRRKQIEMIKLHTEIIRNAVELVSIYTPIIVIYYGNLIIIVVFGLFTQIKNDRDSVVQGFGVGGFCLINIFQLYMMSSSAEELASEAEQVVLAIYNIPWYQMSKRNADMLRLMLLMARNPVKVTAFRAPTFLLNKETFISFIVNTITALMTFSKMNDRRQS

>HhalOR82

PDFDEMELYWNRKNLADVWTWPHIFWLNIFGWWGEEAKTEFGRKWLTRFRSLSIVYFACLITSMMIQVYIKFAEGDIMQNLFTIFASGPGMVGIFKFFDLVIHRKILKSIMGRLSGLISEVNDPILNTMTRKAFKKTWIIFLSTLLVFDSVVLHWALRPIIAAILHKEKTRIIESWPVFLDTWTQFFLSYLFQVPGVILLGHSFYIYDNLYFCTSDVILCHFEILKHKLNRLVLNENEKSSKDLVSCVKYHSAILSVCNDFRDATSKVIIWQSINTVIMLCTGIFILTYLGKNINSNALMNLGEVSFTLCTCLYYYCWFSNEITLQCTKVSNAVYMTNWISAKSSDKKIMLITMTRAMHPVMFGGIMQINLSTFINVLKTTFSFYNFLIAVQVSSTKNE

>HhalOR83

MFDPVKIYFNILIKSFSFVTGEEKMSTWTFHKGLLRLLGNDWLVRKGEISLLRIIWLVLYPIAYITSMISITTLTIKYVLQREHPTMLDYTRAMNGVVACIAFVYAICKSLVLKLFGKNLWHLMDMVDDLGEVDEIAEPHRDSSVRHATLYLGLLCLIPATWTVCSMFYMHNIPFPTDWPWGDYTPFRYFISFTIDFIAATYCAVTHSTYDTIFPISAGAICGHIASISAKMENLGRTGDKEKDKKILNECYRLHVALLRISDHINNTFGLVFLIQSIYTVLHACVIIYQVMKVSDITLAVLNTAPILASSYAQFLLFCYYGELLTDYFERLRFGFYNNHWYQLDMELKKTLVIMTLAANRTVRLEAYGITFAGHKTYVSGLQDSISYYLILKTVTTDT

>HhalOR84

MSLFTGMRGPLDQPGRGKWYGHLYSANVTFINISLFTNVLTAGLVCFSPGVSVEVRCFTGFPITAGTLAIIRRIDMYLNSDKYKDLTERYLSLYPDSPELKADVQKYGKIIRSIPRIMFLFTAVPMVTVGLLPAVIAITGGPRDLGVPAIYPFDPAEYIFVFCFLCFFQSTAAFHSTLSSLLFENMFNTFACRQLALTKHLSRELYRILSTAKVDVKGVATFESHDGTLMSKEESNQTVLAELKQWVKNHQQSSRLARDLQDVYSISLFVQFVFTGSILCMSAFVVANVVGGIVKVIFCGFYVLGLMVELLITCRLGNLILYESDSLEAMIEGTHVYALPGNIYKEWLRMILIKAKVPTKIVAVSLFPLDVETFKSLLVTSYSFFTLLKTMQHLDLRDS

>HhalOR85

MSLQFETTHRTVIQGLRLTGIPLPWSDEASRSIKILNNAYIYFVVIINSYQFAFSLYTVMTIDDFEEICRLGIVLSILVNSVMLTLFYALYEPKLKKFYKDSNTLSYNIMNSELEHYDLFKKNYLKVAKSNNNFTRNVLFFVLCTPLIYCVPTPIIDLCNREYRKHLPFIVRYPYDEHRPGIYEITFFLQMLAILYGDVKKFANDCFFLTVFRIHTVYLKYLSASIRTLGQDFEEIGDAVIKRKLVTWLKLHNHLIRNANDLISLYTPVICIYYANLICIVVFGVFTQIKHESGSIESIGLSGFCIVNLFQLYMQCSTNEEVGVEVDKLAFEIYNIPWYQVSKANKDIIRLIQVMANRPVDITAYRAPSLRLNKEAFLAFISRTVTAVIAFGQMSEIHQ

>HhalOR86

MRAPIEESQLLEGMSIFLLKSTGLWNAINTYLTTRRRTIGLNILTAYSIFYALPYVLFQLVSMFVIHVNVEKLTFLFLNSFPCIQVFLKVSVFWYRIEEQCDIFTLLKQDFLSCIPPHKMPKVREIYKQWARYSNVACVMAFASMILCMSSWIIVPGIDGVDDTGAVSKKILGGWYPFPFSRPPWNYIVFYYEMLLMSSHGSLISLFECVMIQPLLCLCAHFTVLGHHISTLKISDVVYSKSKNELHYMNSELRAILLDYDRLLRYTAVMQDILNLLVTAILGTGIVILIIGVLQFKFGTLDPMFVFHFLTFLSYQATEVFLICTSSSALHSASSDICFSIYSSDWYLADREYARTAQMIMVRTYKPSTLTAIKMYPVSVEILVGLFQFTYTAAMVLSK

>HhalOR87

RFVDHYRPLLGWLRTCGLSTPWGEKPSFAWRLIIACYNTLLVIMVCYMLFLYIYPIMKNSIPFAELCLLGNGFALLGCSLAITLYLVLFKGKLKVIIENMDSIADIIHQNELGGAEFLQEMYKKNARLMKVLTNNSIYFGFLTPVIFCWSVPTMGWFSGNYRANLPIQIDSPYDYHIPLVYELMVLLLSCCLAVSTTKKAATDCLFMSLFNIQITFLKYLSITKSYIQDDFKSTNKTFSKRKLIIWIRLHQQVNLNIQQLVSIFSPLVILYSVGTIMIVVCGTFVQIMDDNNNLMQSISIGIYVAMTCLNQFLMSRTADELTQEAMKLAFFAYDLPWYEMKRADADMVRMVILRSNRPIQVTAYCAPIFMVNRETFRGFMVTSISAFVTFCQIKDRYG

>HhalOR88

RKKKTCRNMFSLLKRYKEEEILNDHCQQFCSEWLSFLGIQPHKTLTLHSAFNSLILIFTLAVWTYTYLEMKQGDSTEALHRICLSLLALGLFSIQFFKHEEIMSVAKQIDDCFSYSNSDAKPFFKKRQKDLFTSGMKIYTDLFTIIVFTTWGSILANLYTQQLFVSDVKPPIPMILPIDSKLLYYFVYFTETAFVVIASIADVLVAHLFMMFTLQLTANFEVLCLNLNSMDRFMKNDLAMLPDSEIIPKININIRHHQEIFKSFNSLKKLFDQIFYILYFSMMSAIAMCRTLLVGDIDIKSLLPLFYLETGYIHIFCHFADMLAEESANVRLAAYSTPWYTFSSNVCTSLRIMVIRALKPPKLYFFIGGSDVSCATFTLILNASVTYFCISFMMENQN

>HhalOR89

MAKSYIQWPWEKENITEIWKWPIIFWLNIYGWWAEEASSTFWRKWLSRLRVICFINASACFWSMIFAIIVKFSQGDIMVNLFSVFGAGPGVVGTFKIVQCIRHRKPLKKAMDLLDVMMSEIDEPELEPIVRNGMKRCWIAFFLCLFFGSCISLHWLSRPLIILLFYGERTRIIDTWPVLDDDWFQWSITFLFQGTNVCLCGHTFYIFDNVYFCISESLLCQLRVLKYRLTHLKLDGSVGSDAALEICIKQHTQILKVCDKLKFASEGVIIFQCINTAIMLCTGIFILTLMEHINFNVLLNLGEITLVIIIILYFYCWYSNEITFQCSELATYAYMMDWTDGTFDQKKKLLNLMTMPMHPVIFGGIVEINLNTFINVMKTAFSYYNFLAAADAGGDHAK

>HhalOR90

MNGEDEKENVCLYESLFLDGIVLTSSKPHIKLPIPVVIFLGLVMIITVTLHAFMYAETINALDKLIDYGFPAILVSSMLCTLKYRREIHNLSRDVDSWGTFRYLEKETEDVRMNAKLLAQRFSRFYMFTIIMGTGAYCLAPLGKFLFLEPSDPADLLIFPCWTPLPIDTNWGFYTTYFCQSVIIICANIAFSRVFCYKIICFYTIGQQMRLIGSALSTIEQRAERQMREKKNLIVKGKTNIHFIMEQEIKQCARQYQTAYRAVKDTTSIFRPVTSVVYHYGMWVICATGVKIAIEEQLFMKVQILMLLNVILVNQYLYSVSSENLKNEVELLRIVTYSCPWYKMPIHCQKSILLMITSMSHIPSFNTIFGTETNMENFGKILNGSYYYISLLLTILSD

>HhalOR91

MARIMLENIKAATDKMVFLVTLSGFYLSPRHPLVQELFLFTNGFLATSLLIYSLNYYWGRIEKTGAVAYYLILCADVCVGSSMGYFMKSSVYSMIKGSIWSHDYESSIITIKRRELETARERDIKMLYKLLVVGIFYMMLDVILMAFVEARINNDLFLLFPCWIPFDMNQISTHISLAIWEIFLVLMVALCMFGAMGIICLFYSHISTEFNILEYAMDKLEERALEMASDYGGFDTRNAEVMKLCYKRCLCMCVDHHAEIIRYFRNATLFLKVSYFAAFGTGVVIFTFAGFFVISDNYAVKIKFTLISLAQLFFLYIISLIAEMIVEKSMTVREKTFDINWYDLPMECQTILVVMRTVSNKPLETKLLSGQTVDLAGFMSLVKVSYSYVNMLLAITH

>HhalOR92

STLPMNTERAESLVYLLKIGGLWINFTNHQFGSLIAVFQIFRLIYLVVGWFLMINVIFLKGVKFLLTPSAVFIPLGFNVVGTSVLYFCKIRMMEKLILQCDKNLSSYNEYWEKKLIQTDTEKVKYIANCFSYGLTCFGIVYTIMHVIVNIVRSITGNPERYTVPLPFDGYLDDTEERNFNFYIYTLLSDLWLAFGVPNTVAFQCTIFYVVSCTTTEINIIKEYLRILQIGQNNNATILPDWNLNQIILKHGQILRFNDLQNQSIALPTLVQCRIIFTLSICFVMFLILQLFGKNTFVMLLAAVFILILFALGLVLCSAGEYLEEKSDELFYAVCSLPWYKQSLTLQKSYFLLLLQTSKTLVFDYAFTSSLNLRCYMALVNNAFSYLMIMKSLSPADE

>HhalOR93

LIPLKVLLKGTWPAMERFCTSFLEYFEGDKLRLGFAPQRVMFKVMGCFWWKKPSLLQKMYSCGIWIGIQIVSLICIVLYILRTEMKTAEDFTKIINSLVMLIPFLMGILKVGILHINSANFKAMVDFLESFPHDGTIFPAGYKFFMNISWNLITIACWSINGFIRGNLVWYAVLPWDTSTPTGYKLGIASQLVSAVPAGSTHVMIGSVMVAAVERLRPQISKLKVVFSKIGPNHEDNKHIIKEAVDLHSAIKRGVNLINNAMGIIFMVKVLSLVPLICINAFVITKVGDIHYIVINLLPMSLCVCGELFMFFGSGQILATEMENMDHVCYDNEWYLAPVEDRRKLSIIIECSRKQLTINAYGIAYANNGTLLAVVQQGYSYFLFLHTMADIFDKN

>HhalOR94

MAKIKLESIEAATKNLKQILTVSTHILSNRFYSLLTFIHATFGFCLLGYSVYYFRNSPEELAQVVYYMGICVAVIVTTIIGYVPSYRSRNVLEDSYHTYDYQSEIMRKKIDEVKESCVKSLKGFFKTIVFLMVWVSFNLTAMTILVGLIKQEPIYLFPCWHPFDINNIIFQILILLWQQYFLSTMIFMAFGGGSMLFIPYTHIKSEISLLKYALKKVEARAHEMARTRKASNVGSVKSKVLSECYKECIKMCVEHHIEILGYFYRGKRLTGIIYTTGFFSGVIACTFGGYNINSENLALKFKNLAILFFILGYLFAMFWIADATTTEFLTIAQTVFEVKWYELPKECQSTLQFMVFMSNQPLFYKLVLGQKVNMEAYMSLVKATYYYLNFLTA

>HhalOR95

AMSDQAIEDSEVLDGLNIRYLKFFGLWKVINDFRMTGKRNKIIKIKVFITFAFALPYIFCQSMSYFVINVDLQKAIFLNLHLFPGTQMCCKIVVFWFRIGSQCKLFNLLKKDFLSVPKGMQSKANKIFTKITRRTNLLCMAAFIVNASIVILSIVDPGISVDYILYHTGDMAAVTTGKKKILGGWYPAPMAETPYYEIIFVYEAIAGTWAGILLAVYVCLFYQVLMCLYAQFTILDLKASSLKIKPINSRRKYKINSQINSSMLKELYEILQEHQKLLSYAKELRSVYNPLVTLILGIGIFVLIIAIFQFLFGGKGNLMFIFKSLQFLAYQCIEVSMFCFGSTYIQTASSDLVFAIYSSDWYKADIKFRKAAQMMMMRARKGEALTAVRMYPI

>HhalOR96

MQWPWKKKNLSDVWKWPLASLVNLFGYWAEEAKTESNRIWKKRFRALTMLFLPYIMMSMVIQGFVKFAEGDIMQNLFTIFAAGPGMVGLGKFLGIVFYRKQIKSVWDRLSAMISEADNPKLEHIARNSFKRTWILFIVYLSIFTASVTDWLMRPPAAAILYHEKARIVDTWPVFLDTWLQWFLSYLLQFPAIVLLGHSNYTYEILYFCTSEVILCHFKLLNYKLKHLVLRDNKESTEKLISCVKYHQNLLSVCNDFKNSTSKVLIWQFLNTVIMLCTGIFILTVLIKQTPYVAVINLFEVCTFEIMSLYLYCWYSNEITFQCSDVSNAVYMAEWINAKPSDKKTMLITMSKAEQPLLFGGILEIRLETFINILKTSFSFYNFLLAFQVNSTEK

>HhalOR97

MANIKLDVIEDASRKIRLLLSLANIILSYKLHSLTTFINSTLACCVLAYSIYHFRNSPEDLAGAAYNLGACASVPVACVSGYLAPVTLRTIVDGSCCSFDYRSSIMRKKLDEVENKRAETLKGFFKTIEYLIYYSTINLLGLTALLGIIQREVVYLFPCWTPFDQNNFFWQITMLLWQVYLIISMTFMAFGGGVLFYISYSHIAAEVKLLKYALKTVEERAHEMAKSLKRSQGEPYARILSFCYKECVRMCAQHHSEIIGYFNNGKDITGIYYSSGFLCGGVACTFSAYFIISDNFALKVRYLGMTLLLLGYLFVLFDIAEETTNELQTVAETAYAIEWYKLPKECHSTLKFMLLRSCEPMFYKLLLGQRVGKEAYMALVKATYYYLNMMTA

>HhalOR98

MDPPRFIDHYRGLIAVLQRCGLPEPWLSSSPSRLRTLYQLFVVFSITYIIFSYVYTFIISSMPFQDFCAIGIAGGCNVCGILVFIHHKRYSTRIHELTDTMDAICKQIMTSDLNKKDEFLKEYERNSKIMTALANYSFYLNFTTTCSYFLSLPAIEWYTGNYRANFPLAISNSFNDRIPGVYELIVLAIAGSTSISTVTKSTYDCLFVSLLKIQTTFFKYLYETIDNLKGDVQSQHHFLEWIRLHQEIMKNNEELLQTFSPVVIIYYLLVINIVVCGAFVESKKENDHLIQSISVGSYVVVTILYYFLLSNAADELTNEAQKLTFAGYALPWYQMKKRNSSMIKLILTMSNKSIEITAYKAPVFLLNREMFVGFMVAALSAFLTFCKMGGS

>HhalOR99

RQTVSSASYREYYSKKVDMSVPPTRWVRSALEVSGMLAPSEYPKLYSIFSSYSTAATAYCCGASLAYFIIMEGATTEKLQSIQITLTILSGLMKLCNAVLQQNKLNTLLLDFDGLWDEFYEEQLNREIMDKSEKFCRLLFKLYKYFVTVTPITNIVMALIGYLATGADQLILQIYMPFDNKKYYIPTQIVQMMLVISPLLANTTSFLLYLVASEQLTTYMRVLRRKLREEKICNQTIRQHQEVIKLLNNTNELFSWLLYFETTAISVECSCSAYALYKVHIKEGQRENVFIDLINFVFCFFTPYVISYCGSKITTESNLLFREAYNNAWYEHDLKAKKDLRILMLGASKILNLQYGNIVVFGMEHFKSIIQVTFNVFNAIIWLEHADQHH

>HhalOR100

MVKILKSLLQELPGVLEITFVISRSPVRRFIQAFVTITNVVMSIANAVSLYFLGLERSLGGTASFAAYGIIICIKHAIYYFRENEIKQLLGFFVEIEKRHKKGWEKEMFEKISKDAWNVVYKYWMIILPYEILYINVLTLLDFVIGYVIPNFPSIRVDVPCEGFIEFFEPRTLKRFIITVPILIWTMASMTVHLGSETLVFISIIYTKIELRIIRKKLEMIKNYLEKKEDDYKIKSEKLLWKVIAQHQRTIEVLNNMKGILGLPVAIHNTAISVTLCVIFYCLMTFDERGSLSIKFHGITLIMCCGSLVLGLCYFGESMEEENNEVICSIYDLPWYSEGKNFRRTVAIMLMQAQKPFVIKYRGLATLNLKTFMQIMNASYSYLMMLKSTV

>HhalOR101

ASLVTMLPRFSGDKDLADVLRLQKISGFWFHFNEKYVIFLQMLRFLYYVTFFIFANVIAYRIGMKNALSGSYFLVSWGFYGIPQLITYMLYNKKANNVIERLKLLYGKRTEQWQWDMFHKNSKFVWIIIRFNLIAAFLFIVVYYLPPLLNDLFRLIFNAGGENMFSFPYPLKEIQSESRDFKYYGIFFVSLFWTTTTIFYGVGNITFHPIVCSYCCIEIQILRKTLEDGRKKKLLNDKTFLKKIIANHNEILRIVEDAQVVLGPAEGAQIATGGLLLTAFVFALMEIKGNIILLIAHISCLFVMTLLNFSICFFGEMLQLQGDKLFECLCGLPWDEMTPKTRKNFNLILLQARKPICISFKGLLPVNFESFKFMLNTSYSCLMLLTTMKE

>HhalOR102

MVKVIRSQLGDLPTVLEDTFLISSNPRARFRQTLIMITNAIMSLTCAVSIYFLGLERSLEGSANFAVLTVTNAIKHIIYYFRKNEVRELLNVTVKLQEEHKKEWERKMFEDGSNDAWNDVHKFSVILTCYVIFVLALPSILDFVIGIFFPDAISIGVHLPCEGLMDFLGPRSLERFVFTVVVLLWCFEAITIDIGTESLTFIPIMYTKIELRIIINKLLIIKNLMNERDIGYRDESEKLLREVISQHQRILEILNSMSKTLGLPLAIHNSTFAITLCFSFYCIITIDESGSMAAKVQGTSAIIIIGSLLCALCYFGEQLETENQELLKAIYDLPWYNQNLNFRRAVLVMIRQSQKPLVINYRLISNLNLQTFMQIINKTYSYLMMLKSTV

>HhalOR103

MVKYIRSLLGDLPTVLDKTFLISIDSDKRFKQIFFTICNAIMTTACVGSMYFLGLEKSLEGAAIFSALSTTISVKQLIYFFRLAQVKKLLYTLKKLQNHHRETWEIEMFEAGSVDTWNAVHTFCLTLLCYLMLFLVLSAVLDFTIGIIFPRAPSLLVQLPGQGFIDFFEPRTLEWLLVTTLFLMWAFEAMVIHIGTESLTFVPIMYVKIELKILRHKLLLFKEELEKLGKNTRHADKLLTDIILHHQRTIEVLNVMKKTLGLPLAVQNTAFSITLCFNFYCIITFNEGGSLAVKFNGVLVVICIGLLLFGLCFFGESLEKENHEVLNCIYDLAWYLQDKNFRRSVLTMLRQAQRPYVINYRRIANLNLTAFMQIINTSYSYLMMLKSTV

>HhalOR104

MGWPFNRKEISEIWKWPHIFWLNIFGWWAEHAKTEFLRTWLSRYRFILCVYFTIVTSMMLIAVGVRCVQGGILDNMFTVFGACPGIVGIFKVYCLILFSKPLKNAMEGIDELFLDSKDPNETKMIRNTLKRVWIIFTFYLIMGSCISMHWVVRPVISAIFFGEKTRIVDTWPPFIDTWPQFFFSFCYQLPMILGLGHAFYIFDNIYFCISECILCNLAVLQYRLNRMKLKEKQGRSVDLVHSIEYYSKILRTCTYLRDSSSAVIIFQCIITVTILCSGVFIITMAEHTDVNVLMNLGEESTVVVFVLYFYCWYSNEIRFQCQDVCNAAYMSDWTNGTEENKKSLLVLMTRTAKPVMFGGILEIDLSTFFTVLKATFSYYNFLIACVSNK

>HhalOR105

MIGLRVGDLVHLLENTFLYSEKSKTFRKFLNMFMGAYSVGTLVVCLLAAIEGGFKKTMEQTAFYIVIGIITSAQQVYLLCYQDQVKKMVDILKGYQEQHVGKWQTEMFERDSLGVWKVIRYYNMVMGSYNVFYFSLPFVVDLVGGLINPNFPVVRFPLPSQQYVEDYEPRSWGNVLYSLIGITTGILTIEMNIGIEALIYISIIYTKTELNMIKKKLSIIKEVMERKGDNYRMDVVRLMKDVTRHHQSTLEGLDMMDNILGFPIAVQNTIYSICFCIDLYVLTIFDSEVKPGPFIVSIFSLTMMAIILFLFCYIGESLENTNNEVFEALYDVPWYQEGPQIRKHLTMMIQQANKPFVINYHGMSNLNLHNFMQVINTSYSYFMMLKSTM

>HhalOR106

MLGIKLASYETDPAKLTVFRNCLIYAGALNDGTLKSKFFIFYRAYGLIVGFLQYAIMATKTTEFMTVVEVFHWMCDFTIMTSMTMSCLYYNPILLRMEARIKAGFFDYGGPLTPEQIKVRATMNRNSQLMSKLYCSLCYCGLVATYIHVFFAKKEYLLPYPMWFPHEIRNMYQYVLTLIHVFIVAETMTICAFSQLSAFVALSSHLIAQYKILIIAIKEIDLIAGTKATPEEKILKMHAKLKTCVKHHVIINKYFDDLHKLFSIPLLTTAIFICLAICTLGFILLSPNVSVPVIGALIILFLPEMFIIVVYCVYGQKVADVCEEFGHTIYYSDWYTKPLSVQRDFLMILIGSRKKRQLTGFGLHEFSMKGLSEIIKATFSYFNMLRAMN

>HhalOR107

MAIDVFRKHRYVLRFLGIVLPGELADSWLRILFHIWFRYLSVCLPVLTLCFLFGITQLHDQYSVTACMYSFAGVVSTTSASFKLILLYSHRYDIKELVDFLKDFKADTTVGRITYVLIYFYEFLALSFRVIALIEIFMKAKLKLLMPFWTPFPRDNVMVAICTYFFFQGTAIIKTICNFFIDTLFLLISNQTCHRMFILRNTFRIIGLPEEEKLERLKHKHMLKLPPGVRPTDSNILKLCVEEHILLLKVVDKFTKIINRIFLPQSFNAFSSIVIVLFLVSQAEDMVRESFKVIPWTTVIFLQLFVSCFAGELIKSRCFDFFDSSYANQWYNCDQKIKSSILVIQTYTSRPMIIRGAYVFELSLHTFETAVREVFSTFTVLYQLFNTK

>HhalOR108

MLERIKGKKNLTDLLLLLKICGLWFDFKGNIVGVLQKFRYLYFLTVFIFMNISAFQNGIKSAFSGTFFLVSGGFYVSLQVVIYYLFRRKTWDIIEKLRMFNKERNEKWQWKIFDEHSNVVWIVFWLYGVAAITFILVYYTAPLLYDLCLLIFNTDDDGYIFSFPYPLEEIRKNNRDFVHYSMQLMSLFWSLLLVGYGMGSLGLQTIICSYCCIEIRILCKTIEDGGKRKLLNNKQFLKKIIKNHNDILSVIADAQEVIGPAVAAQIALGAILLAAFLFSVMEIKGNISLTVTYIILFNIMASLNFTFCFFGEMLQTQGNELFECLCCLPWNEMTPKVRKDFNVILVLARRPILISYKGLLPVNFESFQFVMNSSYSFLMLLTSTK

>HhalOR109

MWDWRQLNWLYYFGWWPSAAKTEVGYKINRVYGIALFIWDFIQIGPEVAALYIALSNGSLKGTVLNLNTVLMGGVCFMKITGILLNEANIKWIIAKLEEMENRGKNMLGLNEYKMIADYRDRRCKLLVIIVFTYLSGLVQWIVRPIYDICNGRTSLIIEAVIPWDKDTIYGWTIVFILQFVHLTTAIMALIIIYVLYLSIMEMILCQIDVLHYSLKKLDFSPPGADYHSSITLRYCVKQHQDILSLCYRFHKVVNVQLFVFMMFSTVVLCLSVFELSSIKDTTLFKVSNLLELTLNTVFLIFMYCLYCHNTVDKLTEGTLRAAYKNNWYMGREEDKKSLDILCTMSIKPFEFGFIIPVNLDTFITVLKSAFSYYNFLKAIADEE

>HhalOR110

GMDVFRRHKYMFRCVGVYFDGELGRSGRVVVELQRWITTFLLVYMHSFGFILGVLYRHSEFPLIPTLVMCCGMVSGPSTLLKHMMLHRRRGQVKELMDQLNGYKVEGGVSTFTKYLIYSYEGMALFFNASSILKNALENPDLTVPYPAWEAYEINTQGRRTLHFFTFYGAIAICWLVTASVDTVVLHLTNQLCDKLAILNNTLQLIGVDQEQRESVLTKKKVLPLQDYKDETILKTSLEEHCHLMRLTKSLSQFLNEVFIYQGVNSMAVLLVCLYIFSNSSDLVNDTVAFSGALVVVFLQVLTSCWSGELIKMRCEQIHFSLYNNQWYNADHNLKRSLFIMATYTAKPITLRGAYVFELSLQTYETFIRQAFSFFTVMYQFLD

>HhalOR111

MWDWRQLRWLSYFGWWPAAAKTKRAYRLLRIYGIVLFLYDFLQLGPELIALYLVICKGSMNEVVLTLNNNLLGLGSAWNIGCVLYNEKNIMWITSKLEEMELRVKKMIGNEEYDKYAAYKYKLCRNYVLTIFIFLAGLIQLTIYSLYWTYHGHVSYIVETWVPWNTDNLQSLIILYTLQVVHSYTGLTAYATVYHLYMSIYEMILLEIKAFHIALSKLDFSPPGVGSHPPVSLDLCVKFHQDLLLLCRKFNETINVTLFLFIMFSSLTLCLSVFELSLIYDRGKLTMLIELIMLTMSMTYFYCVCSQDVVEQMTVGTLRAAYDNNWYVGCAKDQRALHLLCTMAKKEFQFGFIIPANLATFITVIKSAFSYYNFLTAMDLQE

>HhalOR112

MERFCTRVLEYFEGDKLRLGFAPQRLMFKVMGCFWWKKPTLLQRIYGSFFWIGLQLLTLISVISFILNTNIKTAEDFTKIINSLVMLIPFLMGILKVGILHINSANFKAMVDFLESFPHDGTIFPAGYKFFMNISWNLITIACWSINGFIRGNLVWYAVLPWDTSTPTGYKLGIASQLVSAVPAGSTHVMIGSVMVAAVERLRPQISKLKVVFSKIGPNHEDNKHIIKEAVDLHSAIQRGVYLINNAIAIIFMVKVLSLVPLICINAFVITKVADIHYIVTNLLPMSLCVCGELYMFFGSGQILESEIEDMDHVCYDNEWYLAPVEDRRKLSIIIECSRKQLTINAYGIAYANNETLLAVVQQGYSYFLFLHTMADIFDKN

>HhalOR113

MSNIWPQLSKLNWFGHWPTDDGLKGGLQRTASYTILFWTLCLFGAELFSSQQTFLSGDLIGMLGNLNEFFSGLQCYLATLVFVVYRQPLEEAMRLLEELTQEVTTELEEVAMEEFSRRRKNGRRAYYVFFGTFVMVCYWAVRPLLSMAIYGESSLIVHSWVPFAKDSLLGFFGNFMFQIVPCLSFCLGFCLFASIFVFLSEMFLGHFAMLGKKLNSMDYGKEPLESKLANCIDHHTRLLKICSMFQDITSVPLLTQCILYVLGLCISMFELSDVQGASSGRLISVVAEAQQVVLFLATYCWYGNEITIQCLELMRPGYMTNWYQGTRIEKKMLLNIMTRTTRPIYLGALIKMDMITFINVLKAAFSYYNFLAVVAAVNAEE

>HhalOR114

MKFNAFRKHTICLTILGVRPPGGVKWKWLGVLLDIYLIVQLFCIATWGYLFFQGAFLYLPDAFMDRIVGVSAAVSVLGSLAKLITFRRKGHVLKELFDQLGGFPVNTARGRLVAKVVTIYEVSVAAIVVTHNMHIAIDPQIHRSSPYWTASMTSGTKQDIIRDYLFNLDSAIVALSANVIGDTLFLILTNQICHRLLLLAETVASIGKGIRPLEGLSVPEDATDEEVIRRCVDEHNLLLKMTKQVEELYDRIFLVQLVYSCSDACLTLFTISQYEDFVEAMNQLLPQFISLFLEIFLFCWGGQMINNHFERLHLASYDSEWYSLDEKNRSSILVIQTFTGRPVNVTSTSIFHMNFHTYETICKEVFSYYTIMRQLFSQK

>HhalOR115

MDTFRNEKIALRLLGITFPKDLSSPVLKTMVTLYFWLQIAAEGYWAYLYFRGAYLYLHEHFLIRMVGLSAAISVSGTIAKLIAFKLNSGLLQELFEQLGGFVANTDRGKLVKKTTILYESAVAAILVTHNYGYFINSTPTRSTPYYSRYQTGPYEEVALDYWYTLYCVFVTLTGNVMGDTVFLLIINQICHRLLLLSNTVSVMGKQEKNSTMEKMDMRGGESDQEIIQMCVREHNTLLKFKNQLEELYNYVIVVQLAYSFSTICLTLFVISQEANVMEAMSELLPQTISLFLEIFLYCWAGQMINNHFEELRLAVYNSGWYDSCREEDRSSVLIMQTFTTVPVEVTAVSVFHLNFQLYETICREAFTYYTIMSQLLGQ

>HhalOR116

KDPMRPKKEIKDPILFLERLLEWMGVVSIAEDQKPWYYTPMVVLQHISHVNGLLATSLYVLGDNPFFSKMESAQYFISLLHMVAKYYNLHYNNPPYRRLIANAKYLWQEAKKRPRMNNLLAELSQDVDKKMKFFFFIFAMVSPASCLVTLIANLMQEPEERGKPFVVWDPVPESWYWTSCFIEGWCMMVVLSMLGTTLVACYGNCLQAATQMRVLQQMLEDSPLDLRACVQLHQNILRYIEDINDYFSGQMFLEIVFSSLQTAIRGYVSLKFLNAGNPKVISSFFFLSLCLLGPFIVCLSGHVITDSKEKLFISAYNNAWYSASPREKSSLVILLCQASKIKRLNYKNLLDFNMERYSVVVQGTYSYITLLQGADL

>HhalOR117

MERDEPYAVFYKVLEYNGIKENEHSKHRKWFTVLEGILLISLCFDNTFPLIYVIEDHPFNDKAESLQCVTSMLHISAKVIYYRFRGREKLKRLLDDSVKIWKEMRNMKKYEDYLDETWLAINKRVNVITGMFSLCTPIYCLIAFAINLNLDPEDREPLFKIWDPLKYFFKIEDYFFIRMFYEFMMFSYALSTIGTLYLIYLIPSFMAASQIRFLIKMLSSKDLNVTACIEFHQKVLKYVKDINSLFTVHMFFEIILATLVITFRSYQLIMIITNYDHGAVVVSYYLILCFMVPLLVCYSGQMITDASEDLFHWTYQNEWYRLNTKDRKSLCIMMTAASIPLSLCYRNTVTFDMNRYMAVVQATYSYITILINNN

>HhalOR118

MVMIPPFHYTRKQMVMLGILELPNGPKFLRNIITVVIIALSFNSNLSSFLYFLLEDKPFFEKTESILNCIGILHVFSKALVLPFKRNVLLSVMSDLDEMSVDSNRFQEFRKGYNLNMMLSRKIPQTVLLSMCTLMMCGTIVSIVRYTTQGTVTPPVQLYIPFKDNISLTILYNYFLVLPTTFVFSVMISFLISLSLNISIQISYLIMKLENLGMDLSSKEIDSCIILHQKIMRVMTKVNSLMSGLLFFEYLLTSMQCCLSGYQLLADKKNDGIAGFFYHCTFFAISVTFSSVNCYCGDIIKLKSENIFEAAYCNNWYSLSNVERKKLLVLQLASSKPFTLSYRHLITFDLALYGIILKGAYSLVTVLQTMETV

>HhalOR119

RYLKFFGLWQVINDYRTTGKKNNIIKTTVLISLLLAVPYVVFQFLSYFYIEVDIQKATFLNLNSLGAIQLCCRVFVFWIRIDSQSRLVNLMKKNFLVIPIQKQEAVDELYNEISKTTNFWCLTYIIVNISVVILYIADPGVSVDYILYHTGNMDAVTTGRKKILGGWYPFPMNESPYYELIFLYESVCVLWAGILIAVYFCLFFQVLCCLYAQFVVLGYDAQTFNFDLENGEMLHHYHLRLSDTFRRILQDHQNLLRYSDELRSVYNPLVTLTLGFGILVFIIGALQFLLGETMSPAFMFKMSKVFVFQGVEVFLYCFGSSFIEAATELRLAVRHLQQSVVQGEQRDREGSADDDGRSEKWCSADSYKDVSG

>HhalOR120

AQDEEAAGLTLQKALFKLSGVFLWDPPNKMNAIYPILPMFFINIVAILNFLQVFRYSIHTLHDFTEMILTLIVALVFASSGLKLILLHWSKNYIKELVDFLESLPRLESYKKVKHMEGVCMLYMYILIFTAIPKYCLGIMKGELPFESAWPFDTKTTVGWWIAYSTDILSANFCWFAQSSLDTLLVMSVTLMAGHLARLRKILSSIGKDTVMDERIIKTAIKLHVDLLHGADLVNSSYGILIMFQSLYAIVHAVIIIFYLVKVPDILNAVALSFMLMGAYSQLFLYCSSGQLLTTEFEKVHGAVFDNRWYRSKPSIRKSMVMMGIRSQKLVIVKNYRIFTALHTTFLQSCQESFSYFIVFRTLAANIKLS

>HhalOR121

LIQEPILKMAIDYMKLTRKLYVLAGILTEHNTPTSWKVKLISNLAVCSVFLFNLYAFCNGCVVSGLTKDCISHTIVFIVVHIQSYIKFGILVTMRGTRMKYILDFVEGYVFVNDSYAKHIYLTSFYIIALTVLAYAIHPLISHSLPFYYETPWGSESFSAFASSYFVMFFDLYLIQFVSTINDFTYLMCADAICYRLNRVKSLLESIEGEEDEGKLIRAIKEHQDILRTLNMLADTISPNFFMQVFATLSIVIFSAFTAVTSNESQHFSACLSTLATLLTYCWLGQMVTNATEALHFAAYNNKWYNCNRRMKMNLQILSSFTREPFEIRGCSIVRMNLQTFKEVVTDSYSYLMILITMDDTS

>HhalOR122

YPPLQDSNIIDGLRVGYLKFFGFWKIINDYRTTGKKNTFMIFKFYATFIVSTPYILPQLFSYFAINMDIEKATIINLHCLPAIMMCCRVLVFWVRMDSQCKLYNLIQKDFLHIPEYKMAKASIIYKKITRNANSLCILSFIIDVTLVLTLVSIPGVPVDYILYHKGSMFDVKTGRKKILSGWYPLPMAESPYYEIIFAYELVCVLTVGLFQPLYISLFYQILMALYAQFVVLGYHVSTLNISRNSDTRNKIASEKTESGINEDLYKIIQDHQRLLSYADELRSVYNPLVTMNLGLAICFLIVSVFQYQSGETGDMAAVLKALLFLGALLVELFMFCFGSSSLQTAIELESSACSL

>HhalOR123

QIFRLVIFVKSVSLWILSAYIVGAADFLKKIYAGIVLCTPYMVIQTIFIFCNGHHLQSFVTTMANTIAKRNEPWQKEILNNLLNPLWKIIRISTFYSLLLLTVAFYGPILFDTLSTVFTSAEPLNLNVTIDGLLPSVKKGNFFYYFLQWYNAFLEGLGLAHFIGLVSILPLLVNYIRVEIKILCRKIDEYSRRGVAEQEALLREIIEDHINIMRLTELTNKFLAAPLILQNVAAAAAITLFTYAMTNSISQGYDNVTALLFNLNSPIAMTMVVFLSCYSGEMLHLESLNIYDGFCQIQWYNMPKKRQKDILIILVQVQRPLRIQFRGNAVDLRAFKQVINATYSCFMLLKSFA

>HhalOR124

MKVIDVLSHHRVYRWFLNYLAVACIFIDREWIWKKYYVFMTIFSVFGSGVMLYSLIVFIRDIDSVAVIMHHGTINIDIIISTQVCFIHRHTIRELVKGMSKSFDYESPIVSQFLDDLQEKRVKEMEKVFKYIMFTAVFMQINLIIFSIVEKYMKNLDYMLLFPCWFPFDLSYLPYHILAYFWQHINVEAIGLLICAGMAFSYIVYSHLTSQIILLKFAIEKLKVRAYELACSTLGDNTKEIFEDRLRKCYIKGTIQCVKHYSMIIDYYTQAKNLFKILYLIIFLTGLIILTSTGFALVSENTSLKIKFIGVNAVQMVYLYIFCWLAEEISDLGESIRLSIYGIDWFEMPK

>HhalOR125

ERSRLGLGEYLSNQLGMEGEAKPNLVDRDVVYNFEQERELLMMISCYKVSKSSKLNHFLSLSYVSIVWLFVLFELCMGLYSVVLTIDSPKTQLLETLHTVVLTFYVIAHMTNRLQSGFDAALEIINKGFYTYDENMGETHYEIRKEYVRRIRLVNKWFRIIIIYSGISFLMFNTAKKYLENVYKTEPSKIPINPYFPIPYFMPFDTSTVVTFTSAYLLNVALEFFICSVTICIDEIYVSLIEQLKAQFVILNLSISNIVERALRRYQDGKAGSVPNVEELYQQKEFQDCVLQCLKENIRHHHALLRFTALIRNYVQRTFFPCRDNGRSGPCSGGVSHY

>HhalOR126

ALQMCCRMLLFWFHMDRVCRLYDMIRKDFINIPEYMRDSVRELYIKTNRTFNVTCLVIFIWNAGIEFIFIVFPKVSVDYVQHHTGSMAAVKTGRKKILSGWFPCPMDEYPYYEIIYVYEAFCLLWAATLLNVYFCMFYQVLMCLYAQFTVLGARLSNLKLDFPDFGLSKRHKNIIQNCNYNLYQELYESLRDHQKLLRYTDDLRKFYNPLVTVTLGIGILLLFMGAVQILLGKTSDPSFLFQLFQIFSFQFIEVSLFCFGSSRIESASTDLQFAIYCSDWYKADVRFRRAAQMLMIRTRKSSTLTAIVMYPVNLETLGAIVQFTYSAAALMSGMVN

>HhalOR127

LWKVINDYRTSGKKNKIIKFELAITSLLVVPYICFQYLSYFHIDVDIQKATFLNLHSITGVQMFCKMLVFWFRIDSQSKLYNLVRKDFLDIPLHKRTVAKEIYKNITKKSNVFCNAAFLVNASIVTIAIVCPGLPVDYILYHTGNMDAVTTGRKKVLGGWYPFPMAQSPYFEAIFVYEAVILVIGGIFLGSYVCLFFQVLMCLYAQFAVLGYHLSTLKISPERGDTRVTENRRDDSKMYKELYGIVKEHRKLLSYANELRSVYNPLVTMILGMGLLVLIISVFQFLFGSTGNPMFIFRSLQFVAYQGIEACMFCFGSSFVETAAELGPAVRHLQQ

>HhalOR128

DYLQGGEMAKMKLEIIEAGTKNLKLILTVSTHLLSNIHYSLFTFVHATFEFSLLGYSVYYFRNSPEEVAQVLFYMGLSVAVMVTSITGYVPTYRTRELVEDSYCTYDYKSLIMRIKLDEVKGQCAESLKGFFKTILFLIVWVISNLTGMTILVGLIKQEPIYIFPCWHPFDMKNIVFQILILLWQQYVMFTMIFMAFGGGSILFIPYTYIKSEVALLKYALEEIDSRAYEMARNSKFYNDDLDKSRVLSSCYMKCVKMCVEHHLEILRYFSNGKRMVGLSYSTAFCAGIIACTFGGYNINSENLELKFKNLAILFFYTRLSLCNVLDSRRNYK

>HhalOR129

MDPPRFTDYYRWMLQSFIYWGMPTPWLPKLTKSAWWLLVVYDFLTLLLICFALCIFVFTIACGNLGFQDVTVLLPGFVFYTSALYLSLYQYLIKGHLEKIAADVDAIAREIIESKLGLEKELLQVYSDNSKNIIYYCRLLPCLYVSSSTIFFCSVPIVDWFEGNYRTNFAISIVTPFDYRQPGIYEFVVLLMTTALFISTCKQLNNALFFLAFFNTLRSYLKYLYLSMDELQNNIIEEDYTLTRQSIRTWIKIHQEINRCLQVLLQLFSPIIIVNCMYILVYLVGALFLQTQEKKKQHLPDIFSIHWCYGNGYSSVYDLQYS

>HhalOR130

MKVIAPTTIPVKSWRKKREEVVEKKAKTFKRNFDLIFYLTCYTFINLTLYGLIEGILRGEDVYLLFPCWLPFNLDNTFLYIVVILWQAILTANMQFMAFGGLGILYIPYEHIKAEIAILKFALQKMQERSKEMAEERGDFRKDKDKKVDQLLLYSSHLSCMRSCAEHHVEIMQYYNAVTGILTIIYSIAFGTGILCCTFSGYLIVSDNNDLKVKFTGLCCVILLHLFVICWVAEATTNELLSLGNTIYSLEWYHLPKECQSTLRLMLMKASQPLYYYLIFGQRVDIEAYMSLVKASYYYINMMTAK

>HhalOR131

MLISILAKSSLFLKMQATVKSGFYDYEGNMSEEQIIARAVANRDIQVSTSIFSACCMAALLFSYFKAPLFMRTHSLPFPIWLPYKIKSFWSYLPSMIWMWFIAEAMVTCANSNWVALVTLAGHLIGQFKILIMAIKAIDSLAKDKDAEHKVLERIKCCVQHHLLLINFFFDLQSYFNISLLVAAFSTGLILCTLGYLVISPETSLAVAGSLMFVLIPEMCIAGYYCVLGQKIADVTEEIGIAIYNLEWYTMPLPVQRDLLMMLVGSKKTLQLTGFGLHEFSIKGLSEILQASFTYFNMLMALSGGK

>HhalOR132

GTTMTYQDVIEDSDIIDGLSVRYLKLFGLWKVINDYRTTGKRNRIIKITVLVSFILIVPYITFQCISYFKIKVDIQKATFLNLNSMFALQLCCRLLVFWYRMGSQCRLVNLLKKDFLNIPTQNRKAVDEMYKKISKTSNICCTTYMISNIIIVVLLIGKPDTSVDYILYHTGNMDAITTGRKKILGGWYPFPMNESPYYELIFLYESVCVLWAGILIAVYFCLFFQVLCCLYAQFVVLGYDAQTFNFDLENGEMLHHYHLRLSDTFRRILQDHQNLLRYSDELRSVYNPLVTLTLG

>HhalOR133

GTITLSVYIYLVLNQKKLKKFLVQLKVLQRMRREEWEDEMFHKETDKFWRLYFLYSDFIAFYCIVYMTVSFLMDFIVGYFNPDAPSSRLPSAGQGFIDSTEPRSLNNFLASSISILYIAAIYPPFIATEGFLVFSMIYTRTEQKLLNKKVSILKYALENGELDVNKKIKEIINQHQIFLKVLKSLKETIGFPITIKYATTSVILCLNLFTVSTALTTSEYSGTIIAVTAIGSLGTLLLILCTAGESLEAENSELQFNLYDLPWDQFKPQDRKTLVMVLRQVGKPCCINYQGI

>HhalOR134

QDTIRDSDVIDGLNIRFLKLFGMWNAINEYRSTGKRPPVVKIHVFGTLIITIPYIVCQLQSFFNIDFDLQKLTYLYLHPLPAASLCCRILIFWFRMESISRLYNLMRNDFFNIPQHAKAGVKKIFKKISRLSNICCIFLLLWNAGIEILYLIFPGTSVDYIQHHTGSMAKVKTGRKKIFGGWYPVPMSEYPYYEIIFAYEFVCLFWAATLLAFYFCLFFQVLMCLYAQFLVLGFRLSSLKVDNDDSDSRKNNTYNKNNNTRIYEEL

>HhalOR135

SSEYIANHVGNMKDVTTGPKKILGGWYPVPFTRSPWEEVVYVYEFLLFFWVGYTVMIYELVITMEVMTLHAQVSVLNYHVSTLSKKEIVQYSGKKGLTQREVEDLFYKELLAIIRDQEMLFGYGERLKNCFNGYITMLLATGGLLLIASIFQFLFGAKDAIVSINYMFYLLYEVAEFIFLCFATTMLETSSTNIAFSIYNSEWFTSDKRSRDTIQMIMIRSRKPMSLIAVKMYPVNVETLMSVFQFAYSASALISRMVE

>HhalOR136

VLSHHLTTLKFKLNQKTNQRNNSLLYNQLRDIIKDHQKLLSYANELRSVYNPLVTMILGMGIFVLVIAVFQFLFGKTGNPMFIFKFFQFLAYQAIEVSMFCFGSSYIETASSDLQFAIYSSDWYKADVKFRKAAQMMMVRSRKGETLTAIRMYPVNMETIMSILHFTYSVATLMSRMTE

>HhalOR137

LEERALEMASDYGGFDTRNAEVMKLCYKRCLCMCVDHHAEIIRDLVDLWPPELAFLVQEPKRRKKADNYAVKIKFTLISLAQLFFLYIISLIAEMIVEKSMTVREKTFDINWYDLPMECQTILVVMRTVSNKPLETKLLSGQTVDLAGFMSLVKVSYSYVNMLLAITH

>TpapOrco

MQKIKMHGLVGDLWPNIRLMQLTGHWLLEYHEETGGMIRLIRLGYCWLTTFLVVLQFAFLACFLILDTYDADQMAAATITTLFFLHSITKFGYFAVRSKYFYRTFGAWNQVNSHPLFAESNARHRATALSRMRKLLMIIGVITILSVIAWTTVTFLGDPHRKITDPEDENSTITVEMPQLMVDAWYPWDAKAGFPYFMTFLYQLYWLFISLSHANLLDILFCSFVIFACEQLMHLKEILQPLMELSATLDSVVPNSGELFRGGSTGSNMPLVENDGNDFDIRGIYSNRQDFSGFQTGVSTIQTNGTGIGPNGLTKKQELLVRSAIKYWVERHKHVVKFVSSIGDAYGSALLLHMLTSTVTLTLLAYQATKIEGVDVYASTVIGYLLYTLGQVFVFCIHGNELIEESSSVMEAAYSCHWYDGSEEAKTFVQIVCQQCQKSLTVSGAKFFTVSLDLFASVLGAVVTYFMVLVQLK

>TpapOR2

MDLEEGMYNSGFDSLISFLTGMRGPLEKPGKGRWYGHLYTAYVAYTDFALFVTWITILIRFLSPGVPVEERCFAGFPTGTMSLAMVRNIDMYIKKPRYKKLMEEYLSLYGSSPELNADVKSSGKIIRNIPRVIFFFSTMPMVTIAGAPVFVALAGGERVLCVPSYFPFDPSVNLFLFCCLSFFQGTAAFVSTLRSVLFENTFNMFACRQLALTRHLSRELARILGKVRISDAGTVAFLFEDGRSLSKENTRKKVLDELKQWVKNHQQAIRMSKEIQDMYSISMFLQYAFTGVILCTNAFVVANVVGGMVTVFFCALYVFGLLVELLITCRLGDLILLESDSIERVTEGTHIYSLPGDIYSKWVRTILTRCNVPMKLAALGVFPINIETFRQFVLTTYSFFTLLKTMKH

>TpapOR3

MLKSIVGDLPKFLEKTLLISYNSNKWSKYKQIFCICYFFILNICGFIAIYFSGFQKALKGPAYFSVIGVITSCQQIFTLVNKDKVNQMLNVFLRFQQQRKHQWEKDTFIKESKMTWNFVYFYSLGLIMYMICYQTLPIILDALIGYIYPNFPSLRVPLFAQGFIDFTDSRSVTNFLITVPSIIWCFIELLSHVGGQALIVVSVTYMRAEMKVVNKKLLIVKKILDKNEELQNMANVLLKDVIIHHQKIIKGVEIMNSVLGFPIVVESIILSFCICFNLYVTVAYEGPGSTPVKLQGTYATLALVGILFWACSIGESLEDENSKIFNAIYDLPWYNEKVDIRNTVKIMMRQTKKPFFVNYHLRANLNLETFTLIINTSYSYFMMLKTTMH

>TpapOR4

MVILGVRPAGGVRWKLLAVLLDIYLLVQLSNILLWAFLFFWGAFKYLPSYFMDRIVGVSAAVSVLGSIAKLFTFRRKGQVLKELFDELGSLEVDTSRGKLVSKVTTIYEVSVAAIIVTHNMHIVYDFRRASPFWTSSPTVGTSSEILRDYLFNLNCAIVTLGANVIGDTLFLTLTNQICHRLLILSNTVSSIGKDMRSEDTIQIPDGATDGEIIKTCVDEHNLLIKLTKQVEVMYDRVFLVQLIYSFSDASLTLFTISQYENFMEAMKELLPQFIALFLEIFLFCWGGQMINNHFESLHLATYDSSWFALKREDRTSIFIMQTFTGRPVNLSSTSIFHLNFHTYETICKEVFSYYTIMRQLFSLK

>TpapOR5

MIKQAPITDSDVIDGLFVKYLKIFGVWKIVSDYRRTGKRNAFIKFQVIISIIYYIPYIIFQCMSFFVIQVDIQKATFLTLNTLATLQVYNKLLVFWFRMDNVCRLYNLIRKDFLRMIPEYRRAQDREIFMKISKNCNMVCMIVFIANASLLMCIFNTGVPIDYILHHTGDMAAVTGGKKKILGGWYPVPMSESPYYEAIFIYEMIGVAWGLILLIVYVCMFYQILMCLFAQFTVLGHHISTLKMESLKTGKRRRINDQEKSDSRMYKELYAVLLGHQKLLRYATELRSVFNPLVTMILGIGIIVLIISLFHFLFVGFRSALLIRSLQSVVYEAVEVCIFCFGSSALETASSDLQFAIYSSDWYKADTKFRKAAQMVMIRARKGMTLTAIRMYPVNMETIMAIFQFTYTVTTLMMRTAE

>TpapOR6

MGQLRFINHYRVLVRGLRIIGLPSPWLEKPSILVRIIYHLNDVHLLANSVYMLWAYLYTIFYENIPFRDVCSLGVGASSFQCGILLTIYHLLHERQLKHITDNVDSISQRAIDSQLGDEEVFEEIYMEKAKPVSTLTNYLIYLPFLSLFIYAFPVPLIDWLSGTYRSHHPLPLRSPFDDKQPGVYEVLFFLQATSNISNAKKGATDCLFIAMFKSHTAIIQYLASVMEAMKNDFIINGDHVRNHNKLVKWVSLHQDIIRNVEELLLVYSPVIIINYVNVVGIVVCGLFVQVKKDNDSFVQSFGMGFFIGIIIVQLFILTEAAEQLISEAQKLALTAYDLPWYEMRKPDVKTIQIVVAMSNRPIKITAYKTLLFLINRETYAGLLFSSLSAFVTLCQMKDHLD

>TpapOR7

MGYRSEEAIKDSDVIDGLSVRYLKIFGVWKVINDYRRTGKRNAAINIQVIITILYYTPYIIFQCISFFVIKIDIQKTTFIVLYALIGLQICSKIYVFWLRLKYICKLCTLFRRDFLGSIPEQEKTRVKQIFLKITKKCNMLLLVILIANLSLLIVIVSDPLVPVDYIAHHTGDMAAVAGGKKKILSGWYPGTMAESPYYELIYAYEAIGTAWGALAFTVYLSFYYQVLVCLNAQFAVLGYCVSTLEVGSVNTGTTMRVNNDKVINTVMYEQLYAILQDHQKLLNFSYELKSVFNPLVTIILGMGVLVLVIFFFQFLFGGSRSLISFVRCFQQVAYQAFEVCLFCFGSSSLEAASSDLHFAIYSSDWYKADIKFRKTAQMLMVRARKGETLTAMKMYPVNMETIMAIFQFTYSVSALMSRTAG

>TpapOR8

MMKLDLQKATFMNLYFLPSLQLCSKMAVFWFRMPNLCRILNITRKDFLTIPDYKREGVKEIFKKITKRGNIHCLASFLVNVSILIRSVVYPTIPVDYITHHVGNIDEVTGGRKKIVGSWYPLPMAKSPYYEAIFVFETILITGGGLFLAVYLCFYYQVLICLCAQFNALGYHISTLKINDKNSLKNENLNGHKKGSSMYLDLYVILKDHQKLLRYARELSSFYNPLVTIVLGMGIILLIASIFQFIFGKTGNHVLISTFLQFLAYQAIEVSMFCYGSSSIETASSNLQLAIYSSDWY

>TpapOR9

MQLMSTKHVLKGLFSTDVTGSAENFVQKNSQNFVNGLALAGQRAVLPLFVSFALEALMFLLVLLDNVKNISEVNWFMKMMDYNFSLCGTTFIFIMMIHKEEILLLNEEIDRWWCYISLDRETDKMKKKTGEWLSQFYVYYNNLMRMGTVTYMLMPIGKFFYIGHYDTDDLLLYQMWTPFNLRTRWGFLLTYLFQLITLYISFLSFIFVSSYLLTATSSIGFQMQLIGKAFLTVEQRAKGMLEGSTPESWEKQFEANLLKEIKCCAMNYQNLYWKAKKLFRVFSAVPTFCIYAGMCLLVISSVELVSNSVNNYIRIQAFIMVTLVIMNQYLYSLISENLTDEMEKLRTTIYSSSWYGWPITCKKSFHIMQTMLFYIPSFTTVLGKKIDFDFFSKVVNASYYYISMILSMNSRYGNF

>TpapOR10

MSLQFETTHRFLFRIMRGSGLRMPWIRPQSTLTAISYTINDIFLNLVGINQFVFCTISAFRVTKFQDICALGVTGPLEGTGVAISIFYIIYRKRLLKLVDNWNNLSLRIVNSDLGEADYFKQTYLEVTKNNLSFAKKILFLGFWTPIVYCMPVPIIDAVNHAYRTNLPLRILYPFDDKQPGIYELTLFLHVLGVTTSVIQKIGNDCFFLSLINIQSVYLKWLSASIRSMGEKFKSGDSVIKRKQTEWVKFHMAVLRDVNEVISLFTPTIVVYYFYMIIIVVCGLFTPIKNDHDSPIQGIGVGGFCLVNIFQLYMHSSSAQELTIEAEKLAQQIYNVPWYEVNRSNADMLRLMLLMAKKPVQVTAFKAPTFLLNKETFIGFVGTALTALVTFTQINDLNNAQAKVTT

>TpapOR11

MSLGAMKETKMDSRYFRAIGLWQFVVNYETSWWVIFNFCLGCVFLINISVQLINTLSGGYEFSLLTEKIAVNLTVMESVIKIIYYCAKRRRLYSLSDIFRQDLLICRNHDSDTAGRVMSSGLSDVNAVTKGFVVMIFSTVGVWNALPFFKCLMGDCSRWNIMPSWYPESVDRLSAVVYVFEFFIMVYCAALLYNVNCFFSALLLSLSSQFQLLTISFSAIEANAERIGGNKIKNMERLLKECIIDHQGLLRLSKEMEEMYNPIFLFQMLTSTFTICLVLVQLNERTSSSGDLPVAMACKFLMYLLFGSMELLVYSWGGQILYDKTGGVHRSLYESGWPSGSQQFRRTICIAQIRTTRPEHLTAGKFYMVDLSSFTQIIKASYSYFTFLHGSGGNSH

>TpapOR12

MMMKKEYFSDIPTFLENTYLLSSDNNNSRLKNIMFCVICFLLLCFVNVSLYFSSFKESLQASLFFSIIGTITVSQQIYILLNQRQTKLFLHSLKLLQKRQTRKWEKDIFKTDSQRIWHFIYIYYCIIFVYSCTYLLLPVVIDCLVGYIKPNFFSLRVPIPGQGFVDYTEPRSLNNFFVTMFSLIYCHFIIITYVGTEGFLLSSVTYTRIELRLLNEKILKFKDILNQKKEHKMNNTYKIFQETIIHHQLILEALKNLNSSIGFPVAVKNTTISLCICLNLYAITTFLTAGDYGAIISSSFSICSVNVLLFTLCNIGESLETVNSEVLYNLYDLPWYQLKTDTRKNLNMMIRQAKKPIAVNYHRRANLNLKGYMQIINTSYSYFMMLKSTVR

>TpapOR13

MDRAFVRLIQYLAGERLESGFVVQRAMFKCMGMFWWEKPSLAKRIYSIFLVLIQIISVVTVTSYIINTKINTVEAFTQVINCLIVSVSFIVGIMKMLCIHLSGRDFATMIEFLEELPPDDVVWPARDKIIMYSVCSLTTIGGWLMYGIVTKGWPWDADFPWDKTTQVGHILGISLQIISAIPGGLTHLMIDSILYASVEKLRPHINRLKFKFSTIGPCFEQNKAIMRDAVELHIAIQRSVYLINKAFSRMFMLQVVSLVPLICINAFVITQIDDIRYILTSVLPMSLTLCMEIFMIFGAGQLLASEVVQQGYSYFLFLHTMAEAIEN

>TpapOR14

MTEYKSPLHYLKKYMTVTGVLDLPGVPWPLNKGVTLVSCFVQFALPLAAVLYCLTESEPFFDKMEGFLIFLCNSHLASKIFTFVLRREKLLSIMAMIEEMSEFSYNNGLKEAHKKDLWYSKRFADGVVGSFTVLHFLCNMKSVVKHFFNSDTIPLQIYVPFQKEGNLYWVCVYCIVICVFPVINLATAMSTMVTFILNLSTQMSYLSKKLQSLGDESSDMKSCIILHQKVLKVVRDVNDLLSPLLYFEYLLSSIQCCLSGYRLFLAKEEFSSKMVGVTIFVLTILVPGVNCLSGEVIKNQSEQIFLSAYDSKWYQMSISEQKDLNLLMLVAARPQTLHFFNLLVFDMILFGGILKGAYSLITALQTISST

>TpapOR15

MEKKSDDTGYVFLYEFNFLYGIALWTRPRIIIPLPIVTFFGIFMLTTIIVHNYLYIETINWLNKMLDYGFTTINLSSMYVTIRYRKEIIQLTRDVDTWGSFVYIERETEEMRQNTKRWAERFNFFYLCTIYLGVTSYCFFPLGRFLYQEFTEEMDLLIYRCWSPFPIDTDSGFRITYAYQSVTLLSAYVAIGRAFSYKMICFVTISRQMRLIGKALLTIEQRVTRMVSENVSPENWDTIYNYTLEQEIKKCAIQFQSMYRAAKDTTTIFKPMTSVVYHVGMWVMCAAGVNLATEQQFFMLIQTFMLIFNVMITQYIYGVFSENLINEVEKLKIATYSCPWYNMPLSCQKSIHMMIISMEEIPSFVTYLGVKTNMENFSKILNGSYYYISLLLTMQGE

>TpapOR16

MMFSLSYERDPSKLQTNSIYPLIAGALYDGTRKTVLIILLRILFLTSGILQYITKLLVSDEEESQPAIIEALHWIIVYSSVLAMLVSCLRKSSLFFQMRSFVKSGFFDYGGTLTEDQIKVRAEANRDIQLYTILFTISCTLPLLFSFFKAPLFMGKFSLPYPIWVPLKIKDWWRFFDDLQSYFDIALLVTAFGTALILCTIGYLIISPQTSTAVIGSLLLILVPELCIAGVYCVFGQIIANVTEEVSITIYNLEWYTMPLSVQRDLLMMMIGSKKIKQLTGLGLHEFSTKGLSEILQASFTYFNMLIALSGGK

>TpapOR17

MTYQPPIRDSDVVDGLNVRYLKIFGLWRVINDYRTTGKKNTIIKFIMISTFLISVPYVICQCLSYFVIPVDVQKLTILNLHILPGIQIICKVLVFWFRMENQCKLFDLITKDFLGDIPKNKVNEVKNIYINITKKCNIFCTIAYYVFASNLILSIVNPEISIDYITNHTGSMAAVTGGKMKILGAWYPVPMSESPYYEAIFIYEMIVFVMCGILVSVYMSLFFQVLMCLYAQFTVLGYHISTMKLGSVNTNERTTNKYDQKNEEVMYQEIKVVLKAHQKLLSYTNELKDVYNPLVTLILGIGISVLIVAVFLFLFGEKESITFVLRSFQFLVYQGVEVSMFCVGSSYLEDASSSLQFAIYSSDW

>TpapOR18

MDPPRFAHHYSELIQGLRRCGMPTPWLDKPSRFLQFLYLTYDTFAIVLICFTASIYIYPIATKSRSFQDMSVLIHGLFLFWGVLAVSCYQLLYRHNLKVLTDSLDGVARDILESQLGQEEAFLQLYSKDSKYIGSLSKTMPTFQVCCAWLYCTSVPLMEWYSGDYRSHQPLPVGTPFGEKQPGVYEVLFLVVVGTLSVCCSKKGLNDCLFLSLFKILTGYLQYLSVSMKKLQSEFNTGDNTHIRRKIIRWIKLHQEVDRNIRSLLDLFSPVVIIYYVYMIGFLVTAAFVQMKKENDNIFQSAGAGGSGGVIFMQMYMMSNTAGKLNAEAKMLSQVAYDTPWYLMNKDNRNMVRMIIAIANKPIQVTAYRAQAFLLNRETFANVMLSAVSGFVTLCQLKDRYE

>TpapOR19

MSAAPSRSVRKTLELYGMMLPSGFRYPRLYRAFSFSCLFAIVYTFVGSFAFFVLMEGAITEKLQSLQISLTILNGLLKFLNTTFQEKKLDALLKGYDGLWEKLYDEPRNREIMDKSGRWCRKLYNFLNYIVIFTPSFNWVVAMLFYFVLGVDDLAFPIIMPYNGKRFFIIAQTVQIFLIMPPSLAHSSSFLLYLTLSEQLAACMRVVRRKLREERISNETIRAHQDIIKLVNGVNDVFSWLLFFETCAISLECSCSAYAMFKVHIKEGKRNTMFSSLTNFVVTFFIPYIICYCGDKVTTESNLLFREAYNGMWYEHDLQAKKHLKMFMLGASQSLNYQYRHITVIGMELFKAIINMTYTFLNAILWLE

>TpapOR20

MKISKGSNLFCSTTFLVNSVGIFLSIVDPLISVDYIAHHTGDMAAVAGGKKKILGGWYPVPMAESPYYEIIYLYEAVGIILGGTLLTLYTCVFYQMLVCLYSQFTVLGFYMATLKVNSVDTENRRKNNSYEGKDSTIYNQLHTILLEHQTLLGYTDELRSFFNPLVTMILGFGIFILVMSIFQLKAANEISMVISSVQYLAYEAVEVCIFCFGSSAIEAASSDLHFAIYSSDWYKADIKFRKTAQMLMVRARKGETLTAGKLYPVNMETIMAIFQFTYSVSALMSRTAG

>TpapOR21

MFQKLKSLIFETDPLKMATFKNCLIYQGAHYDGSWKSKFFVVYRTYAIFIGLVQYSLKSFEAAQSGDFLKLVETLHWVADFFIVFNMNLSCLYHSHLFVEMDTTINTGYFNFGGQLTKEQIKVRIDMNKQIQLFSNTYLPLSYCALFATFLKVIFASEEYPLPYPMWMPHQITSVYVYIGTLLHVWIVAETLAFAAFVHWSTFLALSSHLIAQYNILFLAIQEIDLIGRNEDLKEKETIMLEKLKGCAQHHIIIIKYFEDLQKFFNVPLLSTAFCITVTICSVGFVVLAPNASLPFIGGLLILLVPESFVVGVYCIYGQKIANACEKLGDTIYYSEWYTKPLSVQKEILMLLIGSKKIRQLTGMGLHEFSMKGLSEIIKATFSYFNMLRAMQQI

>TpapOR22

MSYQEAIRDSDVLDGLSVRYLKIFGLWRVINDYRTTGKKNAIIKFQLVVTFLIAVPYTFCQYLSYFVIEVDIQKATFLNLHSLPALQMCCKILVFWFRMESQSKLYNLIKNDFLTSIPDHKRAEAKEIYKIITKQTNNLCMAAFIVNLSITSLSIADPGISVDYIAHHTGSMLAVTGGKKKIMGGWYPVPMAESPYYELIFVFEVIMVIWGGIFLAVYVCLFYQILMCLYAQFSVLTLHISSLKIDSKARNYSILNKELYTILQDHQKLLSYANELRSVYNPLVTMILGMGVFVLIIAVFQFLFGNTGNPIFMFKFVQYLMYQSIEVCMFCFGSSAIESAIEFRSAVRHLQQRLVQGRYKVQEDCTDVDGQS

>TpapOR23

MDPPRFINHYRPLFTWLRRCGLPTPWLQEPSRFLELLYLGYDMIMVLVVSYMLWIHVYSIAKISITFQDLCVLGISSIGYVCGLLLTMYHMLFRSRVKKITDGMDSITKTILQSELGGAEFLQQIYQKNAKLMAILTHCSVILSLITPFLYCCSVPLMDFLVGNYRANPPLPLVSPYNDKLPVVYELTVLLMSCSIAISTSKKSVTYCYFISLFNIQTTFLRWLSVSKSSFQKEFLNIDNVLIRQKLTIWTRLHQDILRNIENLIAISSPVLIIYSVLVINIVVCGAFVQIKKDNDNLIQSISIGSYVIITIIYHFLLSNTADELSTEAQKLAFDTYNVPWYEMKAVNVDLIWMVLIKSNRPIRVTAYSAPIFVMNRETFGGFMVSAISAFVTFCQLKDRYA

>TpapOR24

MSHQPPLVESDFFDGLIVKHLKYFDILKRINFYRSTGKRLRLHSFSVAFYFSSLLPHIFFQFISLFVLKIDLQRVAITLMIFNPMLQTLCKIWSFWFYISEISTLFNLLKKDFLDSTLLHRQAHAKEVYRQTARNCNVFLFIAYAGIILIIPIWILMPGYDTEPPVPGVKKVLSGWYPFNNADSPGYEIVYVYESFITITIGPLFPLYNSVFLLHLMGLYAQFVVLSHNAASLRKEGDVTGDDLHEELRLVLKDYNKLLSYSDKLKDVYTALVTVILVTGPVTLLVTVLQLLFENTGTRSQMSTWKCFIYMSYEAVQVTLICFCSSLLQTASAEVHFAIYSSDWYEADKKFAYTTQMMMARAMKPVTLTAMKMYPVNMETLQSISQFIYSTAAVLSNVNQ

>TpapOR25

MDVFKRHRYMFRIIGIRFEGELGRLGKILVDLQRWVTYFLLIYMHFICLLLGVLWRYKEYPLIPTLIMSCGILTGPANFLRLRMLYRRRKQIKELIDQLNGYEVVGGVGKFTKCLMYGYEGMAHFFVAASVMKNVIVAPDLTVPFPVWEPYEVNTPERRALHFFTFYYAIMLCWIVMASVDSLLLNMANQLCHKLAILNNTLNLIGGSEANRSWVLKKEVLPLQDYEDSTILKTSLREHFHLLRLTRYLSETLNEVFIYQGINSMAVLLVLFYIFSNSKDWIADIMAFSGALVVMFVQVLTCCWSGELIKTRYEQMHFSIYNNQWYEADPKIKSTLFVIATFTAKPVTLRGAYVFELSLQTYETFIRQAFSFFTVMYQFLN

>TpapOR26

MDAFRKEKIALRLMAVTLPKDVPLFWLKATVDLYFCLEVLALGLWSFLYFRGAYLYVHENFLSRMVGLCAAISVSGSITKLVIFKFNSALLKELFEQLGNLVADTDRGRTVRKVTSLYQSAVAAIIVTHNYEYFIRSTPTRSSPYYTRTQTGSAGEVALDYWFTLYSAIVTLTANVIGDTVFLIITNQVCHRLLLLSNTVSTIGTDESKVTLEAEGIKSDQGLIKKCVQEHNALLIIKNKLENLYNYIITVQLASSFSTICLSLFVISQYGNVYVALQELLPQTFSLFLEIFLYCWAGQMLNNHFEDLRIAVYRSGWYNCRAADRSSMLVMQTFTSKPVQVTSMSVFHLNFHLYETICREAFSYYTIMSQLFSQ

>TpapOR27

MWDWRQLRWLSYMGWWPDICKTKTTYNMVRAYGIALFAWDVVQIGPECMAMYFGIINNSLKGSIFNLNTSLLGTLCFWKIGGVMLSKAEIKWLIAKLEEMDERAKKVIPRQVYEKMSNKRNKNCTRISILVYMGTLAAFQWSLRPLYLLFYGSKVLIIDAWVPWSTDTIQGWSVLYISQMVHVPSCFVGNLVIDILYMTILEMVLGQIEHLKEALHHLDFAPNTLVVDNHRPTTTLRFCITFHQDILSICHKFNKMLGVTLFLAIMLSTTSLCLFCFEMMSLKNADLFRIINLTEVVIVLLLIIFFYCWYCHRIVYQMSEGILRAVCNNKWYLGRHQDKKVILIICSMAMKPIRFGYIVPVNLDTYITVMKSAFSYYNFLKALSVEE

>TpapOR28

MDPPLFVNHYSALIQGLRVSGLPTPWIDKPSLIARLPYFLYDALMIIVVLYMLWCYLYTTVQGNIAFDELCALGVGISLFTCGLLETVYHGFYRHRLKRITDSMDDLARSLLRSELGPEDFLQQLYQKNAKSMTVLTKNTILFSFLCPFLYCVSVPVIDSVAGSYRFRQPLPIRSPFDDKKAGIYELVALVLAVSNGISCSKKGVNDCLFLSLFKIQESFLQYLSTSMEGLEKDFPQNPQQKLFSTNLENRRKLIQWIVLHQDVLRNVQELVVIFSPVAMIYYLNVKVIVACGLFTQTMNETGNVVQSIGVGSYVLVIIIHMLLLSNTADDLSTEAQRMAFVAYDVPWFEMKKSNANMVRMVLAVSNKPIHITSYRASIFLLNRETFAGFVISCISAFVTLVQMKKRFG

>TpapOR29

MKFPWNRQNLADVWKWPVIFWMNIFGWWAEEAKTEFSKKWLMRFRSLSCVYLVVLLTSMWIQIGIQFSEGNMLQNLFTIYTSVPCFIGLFKIVDCIVHRRLLKSVMDRLSALTPEINNHVLDGMARKYFKRAWLILFFMLLILDSFCFHWIFRPLIVAVFQHEKTRILETWPLFLDTWLQFFFSFLFHFPSLLIVGHALYMYDIMFCCTSEVILCHFAVLKYRLNHLTLNDSKASSKELISCVKYHSNIISICNDFKDATANVLLWHSINNVILLCTGIFIITYFGKLIPLQASINLAETSLSTTFCFYFYCWYSNEITIKYIDVSNAVYMINWVNAKATDKKTMIITMSRAMKPVMFGGILEITLETFINVIKTAFSFYNFLIAVQVSSTEND

>TpapOR30

MFSLLKRYKEEDIRDDHCLHICNQWLILLGIKPHRTLTLHSAFTFLILAFSLAVWVYTYLQIKTGDSTEALHHICLSLLASGLFSIQFFKHSEILRVAKQIDDCFSYENKELKYFFRQRQKKLFTSKMKIYTDIFTITVFMTWGCLLTNIYVEQLFVTDIEPPAPMILPMQSKALYYFVYFTETIFVITACTTDVMVAQLFMMFTLQLTANYEVLCLNLKSIQKSSNCSSEIVPDSKINAALRVIIKHHQEIFKALNSLKSLFDQIFYILYFSMMSAIAMCRTLLVGDIDIKQLLPLFYLETGYIHVFCHFFDSLIKESAEVGFSAYSTSWYNFSSNIGTSLRIVMLRAMKNPKIYFFIGGSDVSLATFSLIMNASMSYFLISFMMGNQN

>TpapOR31

MAVDVFMRHRYVLRFLGIVLPGEVPNKFLRALFYLWFRYLSISLPVLTFCFIMGLLQMHKQYSVTACMYSVAGVISSASASYKIYLLYSYRYNIKELVDFLNGFKADTTVGKITYLLIYLYEFLALYFRVVALIEIFRKGKIKLHLPFWTPYPRDNVAVELSTYIIYQGTAIIKTICNFFVDALLLLISNQTCHRMYILRNTFRIIGLPEEEKIERLKGKHILKLPPGKKPTDENVLNLCIKEHILLLNVIDRFANMVNKIFLPQSFNAFASICVLLFLVSQADDMVRESLYVLPWAIIIFLQLFVSCFAGELIKTRCYDIFDSSYGNAWYNCDQRIKSSILVIQTFTSRPKIIRGAYVFELSLHTYEKAVREVFSTFTVLYQLFNTK

>TpapOR32

MVTFQWPWNKEKITDLWNWPIIFWLNIYGWWAEEAQTPFWRKWLSRLRVICFLNASACFTTMIIAIIIKFSQGDIMANLFSVFGAGPGVVGTFKIVQCIRYRKTLKKAMDLLDVMMSEADEPEVVPIVRNGMRRCWIAFVTCLFFGSCISLHWLSRPLLILIFYGERTRIIDTWPVYNDNWTQWFFTFLFQGSNVCLCGHTFYIFDNVYFCISESLLCQLEVLKYRLTHLKIDGSVCSDAGLEICIRQHTLILKVCDKLKDASEGVIIFQCVNTVIMFCTGIFILTLMEGINLNVLLNLGEITLVIVVILFGYCWYSNEITFQCSQVATFGYMMDWTDGTLTQKKKLHNMMTMAMQPVIFGGIAEINLNTFINVLKTAFSYYNFLVAANAGGGK

>TpapOR33

MAESPYYELIYVYEALGVIFGGTILTVYVLMFYQVLVGLYSQFTVLGYYISSLKINSLNNFKNRNLNNSQSYDSKIYKELYEVILHHQKLLSYANELRSVFNPLITIILGMGIFVLTISVFQFGGARKIAVVIRSLQYVAYEGVEVSMFCVVSSAVETASSDLHFAIYSSDWYKADIKFRKTAQMLMVRARKGETLTAIRMYPVNVETIMAILQFTYSVAALMSRMS

>TpapOR34

MEYTGVVKTDKSRNEKLYIFFSSYLMVMLVLQVATCLLYVLEDHSFDDKIETIQCLVSLVHLVGKVLNLRLRKTKFKRLIDDSMELWREMGAYKGNEDLLQDIRKGINIRIKYIMGIFVATVPAYIVFWLITIFKSEYWENLFKIGDPFKAILGDNYYFACSTYQAVIVEHALIIFGVVYLSYLLPCFMATGQMKVVTRMLSSKDLNVTACVKFHQKTLKFVKEITELFSGQMFLETVLSAFPITLRCYQLMQMLSSYDPRALGVLFFLTLCLVAPLVICISGQVIRDASENLFLGTYQNEWYNLSHKDSKSLCIMMTRASVYLNLGYRNIMMFNMERYMLVIQATYSYITILINY

>TpapOR35

METKPFHYTRKQLVLTGILELPNAPKYLRNAITVLSITLVFHDLTAAFLYFLLEDKSFFEKTESLLDAMGVAHIFSKVLVLPFKRKILLSVMSDLDEMSLHSNRQEELRKLHRRNVMISRKIPETVILLVCLLVVSGTLASVVRYSTSGTVSPPLQLYIPFKGRHILFTVLYNYFTIFFPTCSYAVITSTIIVLSLNISSQISYLVWKLKMLGANSNPSDVDSCILLHQKILRVVRKVNELVSGLLFFEYLLTTSQCCLSGYQLLADNKNDNTATLLYNCTFFVVALTTLSVNYHCGNMVKLKSEQIFEAAYYNNWYELKTAERRQLVVIMLISSKPLTYSYRHLITFDLVLYGGILKAAYSLMTVLQTMEK

>TpapOR36

MEPPSFTSHYSALFLGLRRCGLPTPWLEKGSFAAKLLYKLYDFFLLAVVFYILFCYGYTIMAIYIPFQDLCGLGVSTSNYMCGALVTIHQLLFRHRLKRLTDNLDSIAAAIVKAGLGQEVFFIQLYNKNSKLMSTLVNISVLFAALAPSMYCFSVPTIDWFSGEYGVRFPVLIVSPFNDRQPGVYELIFFLVACCMCVSIGKKVTTDCLFISLLKIEITFFKYLSRSMSGLKKEFLNGKNALVGKKLILWIRLHQSILRTTEELITIFSPVVVIYYLAVICIVVCGAFVQIMKDNENIIQSLTIGSFVMTTLVYHFLLANTSDELTTEAQKLTLVAYDLPWYQMSKDEATLVQLVMSMTNRPVLLTAYRAPIFLLNRENFGSFVVSAISAFVTFCQMKTLYD

>TpapOR37

MGQIKLDKIEIATYKMNICLLLADFIQAHKLYSIFKITNTLIACCLLTYSMYYYLNELEKFAADIYNLVMCIDIVLASLIRLYLPVQVNTLVNGSYYSYDYQSELMRNKLDEVEEKRAKTLRKSFNLITYLCCYSFFNMFLMGIIESVLRGEAMIFPCWLPFEADSIQLKILVILWQSVITADLQFMAFGGMGLFYIPYEHIRAEFTLLKHAMRNLEPRALEMARNLGRGEKESVDDRILYHCKIKCMRMCAEHHSEIIKYFNTSSPVPNVAYSITFASGIICCTFTGYFIISDSLTLKIKFMGIICVVLTHLYVSFWIAESTSHEFTKVGDAVYTLEWYKFPKECQSILRLIQVKSSRPQYYHLLLGQKVDIDAFMSLIKASYYYVNILIAN

>TpapOR38

MTVITWVAMPGFDTNLEGSGRKKIIAGWYPFPYSESPYYEVVIAYESMLMIWFGLSLCPYECFLVQLLSGLFAHFSVLNHHLATLTTGANQKRGEDGKSYMNKELDKIFVDYNKLLRYGDVLKDVYNFFITILLGMVMTDLITASLHLLFTPKSASFTVNLFLFFVHSLVEIALICITSSFVESGSSQMRFSAYSSAWNLADKKYRVTLRMMMLRAGRPLTLVAVKMYPVNRETLVSILQFIYTTC

>TpapOR39

MLAVTGGKKKILGGWYPVPMAESPFYEAIFVYESVIAIWNATLLAVYVCLFYQLLMCLYIQFTVLALHVSSLKLRSAECRKKNVNDQRDDSELYKELYAILKEHQKLLSYAGELRSVYNPLVTIVLGMGIILLIIAVFQFLFGGRGSPMFIFKSLQYVAYQFIEVSMFCFGSSSIEAASSGMQFAIYSSDWYKADIKFRRNAQMLMLGAKKGVTLTAIRMYPVNIATIISILQFTYSTVVLMSHMRSKG

>TpapOR40

MVCQCVSYFVIKVDVQKATFLNLHFLPALQICTKILIFWFRMDSQCRFYNLARKDFLDIPGDRLAGADKISKKITRVSNLFCLAAFIVNGSSAFLSVVDPGISVDYILHHTGSMAAVAGGRKKILGGWYPLPMAESPYYEAIFFYETILTTWGALLLAVYECLFYQVLMCLYLQFAVLGHQISTLRVDAVKKRSDIDEKGSDSEIYRELYSILRKHQKLLSYANDIRTVYNPLVTMVLGMGIMVLIIAVFQFLFGGTANPMFIYKFFQFLLYQGIEVSMFCIGSSSVEKASSELQFAIYSSDWYKADIKFRKTAQMVMLRAGKGVTLTAIRMYPSSIKTTSSSSSLKWLSLLLLLSKRVCGQFRSSPPRPF

>TpapOR41

MWFIDKLTQVLDDMNSESDENTEAFIDKSYYLMTQVSLLYLKMDKKHRLISIYQFLIYYAIMAFHFVLLIQTSLFLLDINFIIFTHNTHIGLLTFLVGTVVCDFQRHRKAFVRLHRKIATGFYDYNEPKLEAASLLRAEMLEQRRRLTVVPLSAMVATGAVLLLAPVLDSYGTFDFDKIRGRTSESLPYTFGKYPYNPNEGIAYWISLILQMCLGVVLSSPIGTAGYTYIVITQNIMLQLKILSASLENIEKRTTDLCTRIFPGYYKVPGKRMYHSEESAYCYKKCLRKNFEHHQIIIRA

>TpapOR42

MSQAREIYSKSAKMCNMFCILAFAGMILCISSWILIPGIDGIDDTGAKATKVLGGWYPFPFSQAPWYQGVFCYETVLMTSHGLLISFFECVMIQPLLCLCAHFTVLGHHIATLRMNDVIYSKTHNSIQYLNDELKAILSDYELLLRYAARMQDILNILVTVILGTGILVLIIGILQFMFDARDFMFTFHFLTYLSYEATEVSLICLGSSALNTASSDIRFAVYSSDWYLADKKFAKTAQMMMIRTLKPQTLTAIKMYPVSVEILVGIFQFTYTAAMVLSK

>TpapOR43

MPIMKNIDDIILDERKTRKVLRLVCCLKYIRGQNSINAVNLVYIFLVYMLLSAVVGQGIVLAYFTDDIVEKVESIHYTLCIIIIISYISNELINNSKLDKAGKLMKFAYKTFNENNEENREVHVKITSNIMKSNGVFCCLMSGSCVAYLLFTPMRELFAKDNLPIKKLPVPLYMPFNTNSRLGYTIGTVWEAVTLFYICGVSTSVHQSFRGIMGRLRGELKLLNKSIKQIHERAAKKYKRPTSLMAYSDPVFKSLLFNCLREDIIHYKLLIQYYNLTKKYLGIILLLFIFLSSIILAGVGFLITRPNSNTEDVIKFLAIVSAELFFVYQLCWEGEKVAEESGKIFRSLFNIPWYHCDKNFKIAVNIMLSGTIKPILLKTNVFNVEASFETYNLVITTAFQYFNLLRNVKSA

>TpapOR44

MVKIKLEVIKESTQKMRTLLSLSCLIFTNELYSITIFINTLLACGLMGYSLYYYRNSIENFASIFYNMILCIAVIVAVVGGYTWQVSLHRLLNDSYRSYDYESSMMRHKLEEVETERAERLKNVLKAIVYISCYSMSNLTLLAFAEAVLKNKDTYLLFPCWFPFDVKIIPLQILAILWQEYIILNIQFMAFGGLALFYTSYSHIEAEITLLKYAMENIEVRAYEMAKKHGNRESANARILSRCYLKCLRMCAQHHSELINYFDNATNSMGIFYTTTFTVGLLACTFAGFFIVSDNMALKFKFMGMTLVILIHLYAMFWVGEATTKEFLSISETVYTMEWYNLPKSCQSTLRLMQLKSNEPLFYKMVLGQRVDMEAYMTLVKASYYYVNMMIAK

>TpapOR45

MGDVEDFHYINRTYWRMFSYSHLNVFLDERPTDDVAFWNLKRHVTHFFLFLYFPVWLVIEVSGMFLGRKGDLAQVAFDLSYVAHIVQLVIKMGYFLYYIKDVRSLCLEFERFHTSKHRPNFSRRLLGDSSEFLRRLSNTYYTVIYMNFLFWIVTPLFIQPIIYGLSQAGLVAAAGPQNIIPKFFPVRYPFDEGSTRNRIIIACMELTVLSAGFTYFIPIDQFFVSVIVMVCSEMDVICKSAKSAKELSRELDRDFSLMFGPGDSRKRIDIKLLIEDHQRALRTTKKIGQVINPILGLVVGNCMVLLCTLALVITTKMKTASSFSDVFREVFGFIVVMTTSLITLYLYSWMCGELKTSEEAVFEAAYSTDWYHRDKKYRDTVLIMMGQSFKSRPLRMMSMGDMNRETFIKGLKGIYT

>TpapOR46

MSYTGFKSLLLRDFGFNLHSLLNLMYYLSAIVSTTTFDVWHIITFDETTNFAEAMFQVAIIVHVSSLFVRFGTVFAYRKEIDYLMLLDYREFNSNLHRPKSSLKALKESYVLLKKLDSVTKRIYLAFTIAWYITAGIISRQFVGFLTGSNDFEEVLWVLPTTLPGEASSIAIHMWRWAFHSLFCLVYIYILAATDVLCFSVAVLLKKQYQILRSSLFRGPGESSDSANPLALKLFVQDHVKLIRMTEILRDVMSPIVAAQVSHTIIITACCTYLTATPTANNGAQLSGNLISYATISILELFALCNLSHMANTANEELAVGIYESSWYESSGSKMKGWSLPLLMAQKTIHFKALGLMDINMETFLRVLQISYSYFTLIQAQI

>TpapOR47

MSTKIPKIFMYVFCGYYPLRIINLLIKYFIFRSPQKFLEASWIPPILASNYFTGLLYQVATSMCTGFTNAAYSGLIIFLTIQLSGQIQVLRRMMEDGSLTKHCVIRHQKLMRLFYRINRLLAGIMFTEYILSSLYSCISSFLILKGLSSGGKEAANNVYVLILVLLRAFLICYCGNIILTQGEKLHSSAYFCYWYRKSLGEQKSVLLLMKVTTVQPFKYNYHQIITVDMALFMKSLHTTYSYLTILLSFADHK

>TpapOR48

MKALVFLLKLGGLWLNFSDYPYGIFVTAFQLFRFVYLTVGWIFIINLIFLKGLKFLLTPSAVFIPLGFNVVVTSMLFFLKIGKMEVLILECDKNLSSYYEVWELKLIHNDTQKVKNLADCLNYGMSFFAVTYMIFPIIINIARAVTGNLECYTVPLPFDGYLDEAKQRNINFYIVVIASNLWFVFGMANSVSFQCSVFYIISCATTEISIIKQYIKTISIGDGSSSNILPDWNLSHIIKKHTQLLRINELQNQSIALPNVMQCRFMFTLSICFVMFLILQLFGNDTAVMILAAMFVLILFALSFIHCSAGQYLEEKSDELFYAVCSLPWYRQSVKSQKDYLMLVLQTSK

>TpapOR49

MEATSTVKSFREKYIFKVVKDDKGDYVYKRSVYVRHGVTVWSQDDAYWVVRYPVFLTISFIVLVLVFIHHVLNLGRLVLTTKMLDYVYPTGAILYAFPFYIYEDKIGNVSLTLDKCSEYPYLEKMQKELKRRAEDRVNKFHYIYFNFMKFGCVFYCMMPILRKYLSATTDGSTLLFPCWSPFSLTTWWGFSVIYLLELLTLLTVFIAFGSIISYLITIVVMTGYQCQLMGTSLVTINERVSRMMEKMGVLKGGSDWLEIHTRTIHNELRIIVNHFQRIHGLTTDTCAFFTVPISMCSYVGIFVLAMCCEKLATARFNKSVLFQVSYLGMAVFLNQFLIAFVNQIITYQIEDLQFAVYDSPWHTFQTPCRKMVHIMQSMMLKKPTIKTILGTKADMEFFANIVYATYCCLSTLLSMKVNY

>TpapOR50

MDFGSTNWFGKILDYNYCCGSLVFVYTLWIKQKEVGDLSEALDQWWSYDFLEDDQERMKENLQKKLDRFHRYLYPFIMGSGIGFCLMPFGRYLSESQKSSTSLLLFQCWSPFPLRTWWGFLVTYLMEMLTMLPLFYSWAFILTYLITVMETIGNQLEFVGISLLTVENRALRMMGWTKAYHDYRRNLNYKWMLQKELRNSAIHYQKLYGLAKDACKLFEVPINICLYSGMFALVLSCVKLATERNNKFVLFQAFLMFITVLISQLAVSCINQILLDQMERLQETVYDSSWHNMPIPCRKVLFMFQVMVMKPPSITTIMGVNTDMKFFATVVQTSYFYLGALLSMNVNF

>TpapOR51

MINTYRTSSTITLNIRLQWYFTLLLGVPFSLSQFVCPAYIRVDMEKATLITLNLVSVFQMIVKPWAFLRNIEAHKTLFSTMAKDLLSSVPQHKQAHAKKIYSKIARRCNLFCYVAMIVTASTASLWILLPGIRSEYILYHVGNMNEVTTGPTKILGGWYPLPFDKSPWTEIIYAYEFIVLLWCGIMVSLYEVTLTMEVMTLHAHLAVLNFHITTLKKEEVVFYSGKSAVSAREIQELLSEQFLSIIHDHQKLLRLGKMLKESYIMYITVLVIDGGLILITSIFQFLFGAKDALVTVHFLLYLAYEVTEFSLLCFTTTMLETSSTDIEFSIYSSEWYTCDKKLRNTAQMVMVRSQRPLS

>TpapOR52

MVCMILLIVMFNIVTIIAAYPQMSIDYIAHHTGDMAAVAGGKKKILSGWYPGTMAESPYYELIYAYEAIGTAWGALAFTVYLSFYYQVLVCLNAQFTVLGYCVSTLKVGSVNTGTTMRVNNDKVINTVMYEQLYAILQDHQKLLNYSYELRSVFNPLITMILGMGIFVLVVSVLQFLFGGTRSLFLFARSIQQVAYQAIEVSMFCFGSSALEAASSDLQFAIYSSDWYKADIKFRKTAQMLMLRARRGVTLTAVRMYPVNVETMMAILQFTYSAAAFLSKTAE

>TpapOR53

MFYTLHQKRLQRFIENWNNLNLKILNSELGETEYFRQIYLDASKNSQSFAKRIIFLAFWTPILYCIPVPIIDASKQAYRTNLPLPILYPYDDRQPGVYELTFCLHVVGLVTSVTKQIENDCFFLSLFKIQSVYLKWLSASLKRLGEKFKKNSDVINGREQIVWIKFHVELIRNANELVAIYTPIVVIYYFHLIIIVVCGLFTQIKNDRDTLIQRVGVGGFGLINVFQLYMISSTAEELATEADMVAIEIYNVPWYEMSKSNADMLRLMLLVAKNPVKVTAFRSPTFLLNKETFRGFVASAITALMTFSKVNDLMKRNLQT

>TpapOR54

MSLQFETTHRFLISVMRRSGIRMPWARPASTLSAIAYNATDVLLPLICFYQFVLTASSAFRTRNFQDVCAIGVAAPLEGTAVVINIFYAVYKKRLQKFFDNWNILNMKIHKNKLGQAEHFKEIYLDVTRNNLYFSKRILFIAFCTPIIYCIPIIINDAVNGAYRTSFPVPIIYPYDFRQPGIYELTFALHVIGVTTAVVVKIGNDCIFLALVNIQSVYLKWLSASIKSLGEQFKNGDNALLNRKQVVWIKFHTEFLRDVSELIAIYTPIIVLYYFYLTIVVVCGVFTQIKSDHDDAIQGMGIGGFGVINMFQLYKHSSSAEELAIEAENLAQEVYNFPWYKMKKSNADMLRLMLLMSKKPVQVTAFKASTFLLNKETFIAFVLSSLTALMTLAKINDLHEHV

>TpapOR55

MVKLIRSFSGDLSMFLEKTYLISNNSITRLIQTFFTISSIIVTILCIASLKILGLEKSLEGTACFTTLAIISSTQHIIYYCRQNEIKQLLDVLLRLRKQHKEKWEKEMYEKYSNDAWNAVYRYCVMLIAYMTIYLMLPTIMDVIIGNIFPDTPSLRVDLPCEGFIAFFEPRTAGSFAATIPVLMWCSEALMQHAGSETLIFISIMYTKTELAIIKHKLSIIKNATNEKENNHHKIIEKLLWEVISQYQMALKALENINNTLGLPIVAQNIVISMCLCFTFYGIVTINDRGSLSGKFNGLATASCIGALFFCLCYFGESLEEENNEVLISIYDLPWYYQKRNFRGLLIIMLQQAQKPFVISYRLTAKLNLHTFMQVINASYSWFMMLKSTI

>TpapOR56

MTYQAAIEDSDVLDGLSVRYLKLLGLWSAINDYRTTGKRNIIIKVKLAVTFLLQFPFICSQYLSYFVIDVDVQKATCLNLHCLPGLQVCCRILIFVFRMDSQCRLYNLIRKDFLTIPEFKRNEVEKIFKDITKKCNRLCMAAFIVNMSTVIVSIIDPGISAEYVAHNTDRMTVLTGGKKKILCAWYPLPIEESPYYEVIYVYESMLITFGGVFLATYACLFYQVLMCLYAQFSVLGYQISTLDVDTVCNSEGDSDANGKTTDSAMYRQLYAIIQEHKKLLSYANDLRSVYNPLVTMVLGIGILVLIVGVFKFLFGETLNPMFIFKALQFVVYQGIEVSMFCFGSSYIQEASSDLQFAVYSSDWYKTDTKFRRAAQILMIRSRRSVTLTAIRMY

>TpapOR57

MKTFNICYYGLGMTNTALYSLLSIVKFFYKDPSERVGALVFPVWTPLPLHTTWGFFIVYVWEVMTTCMIILFYSEVFALLMSFSIVIKHQVKLVGIAFVSIKERMSETMVRKDLGPEEWHEEYIRLIDKEIRSGVIHFQHLYSSSRQLSRAFSAVTNVTYLGGMWVMCMIGVKAASEMTTVNVVQAIMLVTVIILNLYVFSFVSECFIEEIQKLRNSVYDSPWYEMPNKTKRSYHIFLTMLDADRFPTLRTIMGTKTNMENLSKVINASYCYFSTIITIKSS

>TpapOR58

MNGVSAANVLLKIILFWYRFEEQCDLFNLLSVDFLTSIPPDKKAHANKIYRKNALRSNAFSILAFAGNVLTIVTWIILPGFDTESTGKGRKKILSGWYPVPYSESPWYEIVFGYEIILISWHGSLVSLYECVLLMPLIGLYAHFVVLGYHLSTLKEDKYFNEEACGNPDSKTFLNRELKKILKDYEKLLRYADKFKNTYNAVITLTLALDTGALIMTILYILFGSSDALSTLKLLIYFSFALIEIVLLCVSSSFVEEASSEIQYSVYSSDWYLADREFATAGQLVMLRAMRPVTLTAFKMHPVNMETLITIFRFIYSTVAVLSKMKE

>TpapOR59

MIGRLLMMTWPWDRKEISEIWKWPHIFWLNIFGWWAEEASSEVRRTWLKRYRFVLVLYFTMITSSMMVAVGVRCVEGGILDNMFTLFGSCPGIVGVFKIYCLILYGRHLKSAMNGIDEAFLDTRGVEEQRMIRTTVKRCWIMFSFYLTLGSCISMHWIVRPIINAIFFGEKTRIVDTWPPFIDTWPQFFISFFHQLPVIMGLGHAFYIYDNLYFCISECILCHLRILKYRLNNMRLDRNKGRSDYLVHCIKYYSKILRICILLRNSTSAVIIFQCIMTVTILCSGVFIMTMSERTDVNVLMNLGEEGIVVIFVLYFYCWFSNEIRFQCQDVCNAAYMSNWIGGTEQNKKNLQVLMTRTAKPVLFGGILEIDLSTFVTVLKATFSYYNFLIACVSNKQK

>AlucOR109

MKRELKAEAVRHFTRLVVKKPMDLLPRLHRGRGVFNLEETSSGRSDSESKLPRAQHNVFYQQFRPMILVLTAFGRLAIQRGSDGEYRWKWFSWLSLFCLLNYAVQTYFAVAICRQRIKAVFESSNYDEFIFAIHILAYIQMHFHVPVSYWVQGPLYAQYLNQWSQFQNEWFLVTGEELKFRHKKAALTFVLVMLPFLALVLVMEKYSTLHDPFEYLLPHFFTIGSTVCVLGTWYIACLEIAFISKDLTKHLIKKLHSNPDPTFLQKWRALWMNLGSLVTNLGTNHFAIMSSIFLTFSVTFLLGLYNALSKIIIGDFSLKTIGYLTASGMSIIIIYIVCDSGHQATSRVESYTSQCILRAHLPAARDDVKYEVDLILRVVQTDPPQIQLAGFVTLNRPLFISFVANTITYLIVILQFKG

>AlucOR107

MEESWLVRYYGGGLGQAEYERVRDFAVSEFTPLVLFLGIFPPTDKMALMSIVVSLSIAVYAFYIVLFTITCSFATDDFVLWSELIHHTSLMYLGIFIRSVLILEAKEMIKLARDYLDGIYHYEEGYVDPIFQQLQDKSRKLQRKLFMLPLFIVLVTGIALGLKPLLDDVNEVEPHPKLLENGITYRSLIPIFYPFNNENTYQVLLMNGALLYFAFLVVVTVIAADLLFIRVSCRISLEIAILVESLNLIDKRAKRLYARKYGLNKKNESWPLYQDCIEECIKENVKHHQKIIIFYEQFSAVAAPAIGGGFFTCTIVLGLGMIVVNMDNVNISDIIAFVGTVFAEMMNAFMISWMSEKIGEQNYELYNAVYNLKWFKWRQSNKKLVITFLDGTRQPLFLNAFGMATINMEAFGSVVNTAYSFLNLVNASETLEEKK

>AlucOR106

MKEKDHSKRKDMLSVDYRKYMYCKLIWIDDGLADRGLTTPLLFIMIWMVFMVFFGLVSFVMSTQNKVRLDNLRSFLLESMVTMSIFNEYMSRKSLARLHHFMDESMKTPRTGLPKEEEILKDAKSLAKKHLTAYTVIFTFNLAAMILSQPVAEWIQGNSWKKLPYPWVIPPSHNEFLFWIVFMFQSIGLYFSHCLGVVIMSFSSITIQITALFEVLLLSLSHIEDRAKVMSEREGADYTTCITECLKEDIIHHQRLARELISATPHLRRTFFALSVTISMIMACEAYPLIMGNFTLGELIKGLLFLVVQFMCWGQMCTRMETMADQNTAVFNALYGTPWYDSGVKYKKLMLTSFTFSREPMYIISPLFIEMKATMSTFYSFVVSSFNILNLIRKMN

>AlucOR105

MGFFTSVDMTDVQRLRVVEDSKRRSGLSDLIGRCGGYRGPLYNEHYSKNILFRIYVHLTDLAVWINYITMIAAAMKSQSVLEFAMVGFPISAESLSLFLSYYSGYKNAEMTEVLLGFDDCFDDDPYPQHLEMEIRKSADYYHHFSRSLLWLQVFTMQIYCFLFPVTNELMHDFFRPRALPLPSLYPCDWKESRSCFIMIIFIHFLGATYVNWKIIGFGEVFFSMVSRQVALFRHLNHNLNQILTSVHVSTDGTVIYRVDKEIDHIYIKSALRKWIKHHQNVMTQYDRLQTLYSWPLFVHFGLVSGALCCSAYATSDETLDFDANLLCGGFLVGQMLELFYLCRMGDWITIETNELTLALTGSYTFVLDRIESQMLRIILSRVHRPSVMRAVGLYPLNTSTLKMLIQSTYSYYTMLKKVNRG

>AlucOR104

MFQERYKHEPDENFETELELCYYECIVQNIKHHHNLLREFNDMVTVADYAVAVPFFAGALFLGLCGLNLISEGDPRYSPKCVSLCLGATEGLNMLLICNYGEAFQQESEKLFGTIFGMKWHKRSMKCKRALMLLQLGSYKPLKITVGKMIALDMKTFSNLVNSAYSIFNLSAVSKGND

>AlucOR103

MGTSTLGARPTMRPNRNLFSLSLLLMKIQGLETPSHATLKLVSFVWKYWMLYTALHFVMVCLLGVTIGDNPYYLKLETCSGMIAGMSMVYRHFVLAFNRKEVHRLMDRINALVDDVVSVYGEETIAPWEKMCCGIMILSTCVVSFTTIPAYAYSYLKFYTDGEVTAPYEVYMPFERDAAHIHHVVIFQMLSFLDQAITLIVSNTFIGTIVVIVSGVTEKIAKRYKEINRNNFHTLKVTTNWHSEIIKIVEDTNALLGSAIMVDCLLSVVHISVSGYLLVKVGFESGTNLHKYIFLNLLCVTIPSYFCLCGHIISVGRDKLHEAVYQNEWYELTPSDRKTLILPTWMADKGLSLHFKKAVEFNLPTYLAIIKQSYSLIAMLKLMDG

>AlucOR101

MWDMRQLRMMNLWGWWPKMIKDPKKRKIMRVYGYCSFGLDSITMIAEIISLYLAVVNGSFRGAIINIVTTTLGTMAAQKIYTMLVHHEFISHICDTLEDLDNRAIELMGEECQVTMKDRERRCLLTFVFVGSCMFTVCHYNVRPIIVYFLYGERTIAMDMWTPWDEQTSETGWIVVLIYEWIHIFAAMYGMTVFDSLFLSIFEMILAEFDVLKIALRKINFAAEKKEVTLEFCIKFHQDLLLLVARINEFLIPIQTIQCVMFTFTICFSGFELLSLSDGSLNKMANLVEVVGAATYITFGYCYQCHCITEECEEVVRAACDNNWYEGSVEDQKKLLIILERAKNPISFGNIIKFDLGCFIAIFKTAFSYYQVLQAFDI

>AlucOR100

MESVAMGEGEKRKTNRSEGCVDYRKFVFCRLMRTDDGLVKRGITGSLLIIMVTTVAMETCSFVSIFMSKQLQSCLDCVRSFLLGNLVTMVLFNEFANRKRLARLHDFLNSSMSSPRTDLPEAQEILNKAREEASSSLRMYIIIFSGNIAPMILAQPLAEWISGHSWKKLPIPWAFPPSDSDVKFGLVFTFQCIGVCIANCLGMVFMSFSSITIQMTAMFDVLLLSLRRIENRATIRTQREEMDYKTSLLYCLREDVIFYQQLVREMTSATPHLRNTFLAFSATVPMIMACEAYPIISGNFTIGDLIRSFVFLAIQFTCWAQTCTKLETMTDQHEAVFREIYQTPWSDSGPVYKRLVYTTLLFSTQPKHIKARLSNEISATTSTFYSFVVSSFNWLSIIRKMN

>AlucOR99

METKEDEYMKPLVQLLKFGGFWFDFSGHKHAMALKWCNIARNVIAFSVWAYQMAYFMGGVSYLLTEAGVFVPICFDEGAVAVIVLCNQSTIRETIRIYRKRFEVFGSTPWAKNIIDSEMNKFNRIFQLPKLMLSVFFLFYSVVPLVYDAVLAYSGKSPYVVPLPLNFLLETPMQRTPAFYMTIYLSYLYFMIIVPRFIAFEALMMYIVAFVVIDVKILVQKMKNLSEKDDGSLFLQEEWNLKDVIDHHSTVVRVVEEHFWLVGLAMMVQNLTFSISSCLVIYLTKTSFNNGDIVLALFCGNFVVLLMILNLMFNGAGVLIENQGEMVLTAIYDTEWYKQPPKVRKEINAMLRQGLHVLKISYWSNTVNFETAMVVLNRAYSFFTLINTGE

>AlucOR97

MVKLFDELAEQEDEELMGVYEKLYGPALQLSLLFPSWKRENLYKTFGIILVYTVTLLVHFYVLSVSVVMLRDDFEAACLAFHYWLIFVMVFLSLALINMDRRTFSFAHRCLARDMGNYAAGRIYSESKPLALEARKKKELFRFLVLPGMVVILAAALLVVPYLKKINNPPHYNAYGVNMNLPLATHYPFPTDHGILHGVVVLGQLSAAFSLAVIVVSLELLLFRVSQAIIFEFKILQYALETLFERSERLFFQLHPDYYGKLSHMNSNYQKCITRCIQDCVKHHYKIQELLQAYEYVLKWPAALGYGIGTGVIGLGLVTLLMAKEKGNLENVVIFSLLIVAEVLNMYIVSVFGEDITTESAAVRDELYFIEWYKLNIPNRRMMLNFQVGITNPVIVKAGGLVALCMDTFSSIMNTSYSFFNLMNANPLDGNK

>AlucOR96

MAKLFGFHIINADSRKCLSYRMSFPMTTLLMAFNLCMLSGNAIGCLIAALLDSNFDRRLMNVKGMMLLIMIILLAINESFISRNRVNRILDYINRIKSITRYGFKEEEDIMNKAIDDCYKSTKYSTTFFFTNSMLMVTLPPTMAIITGESWKQLPYPWVITQTNDWLYYSSLVLQVVATALCHGVGAAGFSLLTTMKPLAAAFDKVILGINRIEERAARKMSEEGITYQESMLSCLKESIAHHQEIVDELLMEKPHLEIMFFAQVTFISIVMACEAYPIIMGIVDVSGLIRGVLFLFIQVMCCGFLNLEFDTIANKNVEVSEALYGTPWYALGVEYRHVVLNSMTFSQNPIWICGMGFFGLRASRATFYSAMVSACNMLNMFRKFA

>AlucOR94

MSNQDDVGDDVSVRRIPRSAQKRPQITVKNEKEFDGGCTDYRKYIFCRLMYIDDGLVSRGLTLPLIIIMVHTVTMEVCSFLSIFGSSQIKDGLDCVRSFLLGNLVTMVLFNEFANRRRLGRLHVFLDKSMRTLRTGLPEEEEILKRARDQASSNLKMYIIIFSNNIAPMVFAQPLGEWFAGRSWKRLPIPWSFPPSDTDLAFLLIFLFQFIGVIMANFLAMVFMSFSSITIQITALFDVLLLSLRHIETRAALRSKLEGTDYRQSLYNSLREDITFYEQLVREVGSATPHLRNTFLAFSATVPMIMACEAYPIMLGNFAIADLVKSSVFLAIQFLCWAQTCMRLETMTDQHAAVFQALYNTPWYEADLKYRKLIFMSLTYAAPSKYIKARLSNEITATTATFYSFVVSSFNWLNLIRKMS

>AlucOR93

MTLKSYIKKTLKWEEPLGLTTMIAVISGAWNTMAPPQSIQRFIYWQSWFQTSTYVLFMMSAGINIFVSTDFFGECLESLHFLVTAFHVFIKYMTMRFRERDFLELFDDIKRVWSGYRIHNEKFLSSTLASVNRTTVIISVCIINVMFVNIGAAVLKNILEPDKIHFPIQIWIPSFCRDSFMYGTIAQVVLFSWPLFIVAQSTTFLNSISVHVEALGLSLAKDIGRQKVWKGDSARRFYKKHQEVISIVSRVNALMAGNWGFEMLCSSLQLTLPAYRTLRALKRNEIEVFNHAVILSLNFMVIYIIFGSGNRILSMGEEINDRLYESDWYKLPVKEKKNVLFMLFRATKPVEYRYKMIHFDLPGFMKVVNTVFSYMALLRFLDGGNDGGNGGLL

>AlucOR90

MVASTSKNRKIKNEQAVRGFTRSEEKQIEDHLFSIFNILPIIGGIFGYHQSPKWSALTYTLNIGMYTSVSLTALNLLYCSYLLRDNLSQVTMAFHCFLISCVVMTASISLTLQRNKLIEFLLKLQFRGPLAEYHDSDYFQALEGKTRQRIFRMLVIFLSCYGACGLIAVIFPFVDLYLKNADQVTNVPEIYWKGLPFAVWWPYDVHNSTSAWILCFLSQGIWALFAPVIVTTAVVLCFYGAELILNHFKLLIFSVKNLDQRTKAMYERKYKENSRTQLENVYEDCFYECIVQNVKHHHIILKIVEEFLALANYAIAVPFFGGALLLGLAGMNLLSTDDLRIGPKIFCASVGATEATNMFLLCVYGEKFQHEGEELFNSIICTRWYKRSMKCRKALMIMQCGSFRPPKITAARMIELNMATFSNLVNSAYSIFNLNSVASATEDK

>AlucOR89

MAFGLEQMDCLTKEEIGIKAHFMRLMNLSGTFSRRKQTRLRSAIIWSVIYVPIILTLVATCIHFRKNFDLSSYALHHAALITIGFIVNVMTVCMYWKEFHDIMDGSTMSYNYDSGLVKNFAQQTIHERFKLSGLLVKLVSYGSVGVIIEVQIFFAIEAFYLQTYKTIFPMYVPMDLDDPFVFTSVVIWQELVVIYTTYLPLMLAVLYYNAWSHLDIEIKILTFAVANIQRIVEEESQNFRHEGIHRETLEAALYETYSYHFAKHHAHITSYFELFSKCVKLITLLLFTMGPVCLVTVGLSLLSDNIGIRLKLFWFLVIQLIMTYAICWIGQYIADVSTGISEVLVTAPWWLMPKSCRSTFLLIMTRCRKPLQMTTDYGVPANMESFMDLLKGVYQIISVVIQMRDG

>AlucOR88

MSFLMKFIDSLAENEDDALWDQLHKFYGPILEIAFIFPSWRRSKLPISLAVMSFYAFIFPVHIWLLTVGIQCVRDDFNLASLEFHYWLIFMFSLVALLMMNGNRNFMISFHRTLTSDVGKYRAGRIYDEKRPLEWEHNKKKQLLKFLSLPTLVLVLAGFSLLVPYLQKMDGTVEYNERGANMKLPIPAWYPFPTHEGILSLLAVLGQFMAAGGLATTVATLDIVVFRLTQSLLFEYQVLRYALETLMPRAKRLYTLKYPAEDMRKLRTNDEAFQRCIGKCLEDCVIHHQDIIKLIKDYKTLVKWPGFMAYGFGTGVIGLSLVNMLSAKEQGRYEDIVLFFLLALAEVLNMFMLSTFGESITTESKELREQLYFIDWHLLNTSNRKLVLNFQIGVTHPVIIKVGGLVNVSLDTFSSIMNTSYSFFNLMNAQ

>AlucOR87

MWKCWKKSSAPPPSLKDEAWKWPHESMMNWFGWWAEELERPLVVKLLQVMRAILIPSHLIFYGSLLYQTSNEFRQGTIISTVKSAFVSGPSTVACFKLYVIVRHRKSLKEITNSMDVMMKGILSRHIPEDLEKEMRSRWTGCRKLYKCCVYFGCSVTTHASVTPLLQTIAGALLTDDPLPFDSWPYFLMGYYFWALNTFCIGHVLYMFDATWFAMADNLQIHFAVLKNYLENLDLTKRSDVDLNLCLKNHMELIRLCRIFRRISRTVIVTTRMCSMLLLCAGTFVLTSAGDEFTSNDRGNLLSTLIYIAAVFFNYCRCADNIAHQLDELTTDCYSAQWVNAEKSQKTSILNMMTITRMEPKFCGIASIDLDTFVNVMRGVYSYYNFLTAVDVGDESETSRTEVNEPL

>AlucOR86

MWLREWFKNRSSKTASKEKQVVPVNIDGTPDFASFATCLSYQKFVGLYLDGSVINYLKISIPILFLTTGCISFAMADILNYKNKNIIWLIENGHWCIVYIAAIFWDTQMGIKSPLLLRMSRSVKSGVYKYAKYDTINREDLEKTNSQVVTTSRFCVFVYVVAVLATLVKPTILEEYDPYRHFFNGWFPFEVNSLWRVSIVRVYELGCAWSAASGVCTFFVTFMAYSYHIEAHLKLLIQKIEKVFDPESSEYDYIPQLDKKIRECLGHHREILRVFNDFSEFCDPTIGCATLMATFMVCTLLYLMTNPDFDVSVIVTFSGVVAPEFSLLISFRLRGQRITDLSNKINEAIYKLKWLDQDVKVQKNVLMWLRLTSKPLELKSFGYRNVSNSGIKEVLQTSYTFFNMLKAST

>AlucOR85

MSKAKKSAVGTSSYKELEGLERAEALKKGYDENGGAYIRLGGQYVVSRSEIWRPVLFYADTALAMFELVTASYFSVLQGDMEAASECFHFIQMIFNMMVISANLQYYRKNIDELFTAIGAGFFDYGDTIDPQTKEKMDKHMMDMRANKKFRFKVFVLVVWVLGGCMFIKVIVAHFRFGDTIDGEGGSVSRKHIVAIWLPGIDEWPSYIAMVVAAYLCQVLIMNSIWGFVLPVICFAEELNAQLHIVGIGLRHTTARARHILYRKYGEQKSGNLKFKYEESLREALKSSVQHHNVILEACKCASTLLNLPLMTVMFGTAVLLCMSGFVMVEDSVPIIANIISLLFIGGEVIYAYLFCYYGEMITATSLEIGDELYNDDWWEGRDVFRPYMAMISLRSNRPIKLSAGGFTDVNNAAFSNIISTSYSYFNLMFTSKS

>AlucOR84

MYYSPWMANNCIMDTQEYKTARKKDYWYLFEMSGMILDWRPGFYIINLAYVVVMVINAVYTCACLIASIFKVDDLIDVCQYLNFVGLLFVSLSVLASLNFQRERTLETCAIGSTEFFDYGVSFSRSEEIEQYRKEGRRRMKILFMVIPPWLTIIALSLMMSGPIDAAFSYPKINATYVNGIYQMAPLKMYYLHPIDNEFIRWLTVLSQAACSGNTALVIGCADLIMFNAGQNVIIQLEILNLAVLDTDKRASKLYELKFGREPPSDPVDKTNDRPLMNIYGFCLKQIVDHHKIILRRAEVYHKIVNWPCGIVVVNGSIVVAMSLLSIMQGGGKPSVLVLSFFLIIAEVASIFMVCEIGQSITSQCERLFDSMYAFKWMDCSVEVNRAINIMKCRFRKPIIMTAGSLTPINRDTFGTMMNTAYSYINLVAASGKEDAD

>AlucOR83

MMGEWWKDLKLPAGRHPESAPTLKKIYDDYIRRFDHLKMFKPIFCDTQFKWHTLLAYFGLFLHNTFIGFSYLVTCILNMDDISQASFPANLVIIHLIVLAILYQLVRLSWTENVTIIDNLVTIVKGREEYCKYTGTQYDVYLINRRSKILRRVTQVFTYVFGFQISWLILPVINSVFGEKRVPNEVSNGVAVTLGVPIWTPLDADHSWFYYCIVCSMQLFFFGSSGLFIGLGVFYNMMSQMSILDEMKLLIRSVGELDSRTSRLLTDKYPKLSIDKYSEKYDKCYFQCLRDNIIHHSFIQRTFSEYQSLVSVTLAIPFFFSSIIIALFFADITSGSLTLVELSIEVIHMCMNIFLMAMMCYFGQLITLKNEE

>AlucOR81

MGSFYQGITSPSENALKKLQISADTYVENGFFIARFSGMYRWSLLYSISYFSCMTFGIVAAVGYILNVSTTDEWDKFLENIHITLLIVNMEAQGVAYHYDQNGYIEIWRAIDKGFFDYEGTLDEETDEEIAIMKSELRNFKKVFQHNYTMLMCITTVLQFSKKPITRYLIGGGTVDGKNNLIWEAPFGLYFPFADYWIPYLLGLFLGNACGLLILITALGSVLQYIYMSEALIQEFAVVKKTMSKCIERAEQIYRNRSNSNQVEHQQWTMDDCIIHCINQSVKHHQITLRMMNVFKKLMYFSLFAIIFDGGLILCISSYILINDEVGITFRLPMPCVIAVEASLALVFCYYGGKLTDANTDVGNGIYECKRWMDHSKILCPYALIVKSYCNVPNELSAAGFTNVDVRTWGNLLSTAYSYIGFLLST

>AlucOR80

MVVEVNSRTYYGIEVVYFKFIGFWQFLTNGLGKDKLVISSIVYGLMFTFFIIVQILDMFIKDYDFSIFSEKLSVNLTCFESVIKIGYYCFKRSSLLELLPLYRLDLLLSAKHSPVISTEILMANRRFVNGATKSFVVMIFSTVGIWNCLPLLKCFTSGGCSTLQIMPTWYPGDVSYVPLNLFVYIFEFFIMIYCAALLYNVNCFFSSLALTASAQFELLSNNFANIESNAERRIEDHASSTDEDTKKATMYVLLRECLIDHQTLLGILQKMEDVFNPMFLFQMLTSTFTICLVLFQLNFHTASGDDLPIAMACKFVMYLVFGSMELLVYSWGGQIIYNKSEEIYWSLQKCGWEVGCDKFKTNVQIALQRSQFPVTLTAGKFYVVNLASFSQVIKASYSYFTFLHGSISNEE

>AlucOR79

MTVVQTNKRTAMTDVHHYHSLLLTMLEIAAVFKKREGSIFSPTGFKMFRVANLIVCFLFVTSCARYVFHEKGAQFFTVAIGTGSIEFCIINMILVSKSDIIDRMLATSAKIFYQLPQNEETRDVLETYRTKGYTFMRAFGMLIGVNEVLGLIKPFWMARLTGQLGLPFDISCLGIPTVPCWIFQIICTSHLIVTVAFHVIIVKTLMYLTWGHSIVITKIMNRRPVHDDEENDRKIIELYCDFSRFSTSFSSLFGLTTFIEVTFTSTRCCFLIYHAIKSLSNNDMEQAIVSVTALIASIAISYVMCSCGEDLVEINQMMRDGFYNSKWYESSPQSRRRMLPMLVLSRVPIRFQYRYYMYFNYEILMKIMHSTYSLSAALIQFL

>AlucOR78

MSQRPDPEGPPSKRTYLSKGLKLKSSVRLDAWRHIGYRLLLLGMTPENFMEKFSVRHALVYLIFFLTHSLYSAHELTVALFFSRSLLERVTHGYILTYIFTFNLQWYYMLRHVGNFHRHEMSLENFASTQAHLDFAEEVFNKNINAFLKYLFISMLWWATNTTTHVFGPLVETVLMYVRSGDFKLATVLPQVFSLPMWAQVIMYIHNATATILLFVYCLASYLVLGTRVLKVKTQCDILNEALRRDYDEESNIRSYIKDHINIIKSAKLLNGHMQMLNGIIFTACYLEFATQMFALTLFEPSGAYFFALAFDLMSIMLILVMQCWFASIVTISLQSVTDAVYETNWYRRDKDNSLNVLMMLQMAQQDYIQRIWFKSFKIERAAILNLIRSSYAVYTALLIFQE

>AlucOR76

MPNIGWLPFAITSLPRYALGCLCQAIIGVNTVSIVIGTFMSFATLFIHYGAQFKLLRARLRGCFPENVALEKAQEDIYKEKTIRKLKDCYNHHLAILRFHQELLKYYGVLLLVFRVAIVFWLCTLAYVSIIVDVNAHTILNMLSFASAELLYVLLFSIRGQDVTEWSYELHDELYSIQWWEQ

>AlucOR75

MDILHYSDQTGFKFIPVYDHMLRSIGVHSERGETQATAIKRYFGNFLILVAIVQGWSSAVAAYDSLKEEDFRAVTNVMSYLSVQLSSFSKFHIARTHMEVTHRLGNWIVEAKKNRPRDMKQPQLEFLVLKVNPAFFYFGLFATFFWCSVPLTNNLQAFIPTQYPFLDKQTSNSISFPLIQVPLYIFFTVTITYTATSLLHFLAIFTTEVKLLSKKWEQVFYDKRRPDNYMELMKDCIQQHIKLLDVMKDLNIIHDSMFAFQVFIFIVHFVSFNFCLVMTSGSNALSSVGPLTMSSLMEFGLLCWMGEEITDALQQFHRSLYMTNWYEASLEDKKNMIVVLEVLKKRHALTGTKVFVASLETFVEAARQAFSAYTLMKGLTTTE

>AlucOR74

MVEDLTVKDLTGIYLLPHSVAYMHFTGHWIGAVPGPTPFRVKMYRAFGGTFTWLIVIATAIASLNGMLHGSGMSDISMNLIIISTSISSLHKYSVFIHQEQGLGRLGRWMKRANEQNKISKNPDTTTDRILKKSLVSFYYSGIVAASLLLVKIVVTGYTYNALIPGLDQRYQPLILIFMEAFSFSSLSLEVIMDALILMNSLFVFRRELMKNVDEWRKMNYKSDNPQQFRQQLKTNVQNHVELLTIFQDVKNYCNSMFGYQVFAIVF

>AlucOR73

MNTEVTERFEYASKAYWGALGFTGLDAFLYEKPPKHNVLRWYAFRIIYIYFVFVHYPIFITMQFWGIVTAESHTLMQISFDISLMGYNIQNIIKLLIWMIRIKTVRSLRLNFSKFNVNKYRPKLSSWIIKRDAENALKFTGRCYWISYANLLFWVVLPTATAIINYSTYLAGYADWQENDFPRYSNTRFPFDLSQHRSQVIVSFLEVILFTLGFMSFQSMDMFFSVIIRMAQAQFSVLNSALFALDGELDKFWGTEVPVNDCKPPMKIHLIVQDHQRMIRYGVRLRKFLSPILGLETLNCITIICNMTIVASSQVSGGGEFLEVALAAFASSLVVITCLVVFFTFTSMTGQLKDAEESVFYAMYSSKWYERDVSHRKSIILMQKQAMTSRRIKMFGLGDMGRSTFIDGLRMVYTYYNFMQRFK

>AlucOR72

MSVNWNRKLNSLNHTRIAFDVGKYKDGRIYSHKACIRMEKELQKETILYLCIPLLIIILGGAILIVPYGSKLVRGYGMMYTACGVDLFLPIPLYHPFPTHEGIHHFLALISQVLLVFCLMNGIIAGVLNFLQYSQRVKLEYRVLSYSLDTLFARSKRVYLRHYPDKKANFTIRDPEFQHILGSLLRDSIIHHQTLVDMMNNYHGLITYPVAVGYMTGAGGIGLGLLSILRALQKR

>AlucOR71

MAPQVNLFKAWMIWMKIAGADPPSVNFPYALALLWKLIMLYGSVHYVIIMFLAIVVGDSAFHLKLEAGLFLLAGIPCSYKHFVFVIRKNKLHEVIDRLNTLLEEVEDVYGTETLAGWQRICNMVMYFYSTQFTMLVVPVFSFFYYIYYWEGVEATPYEVYIPFEKENHIHRVMLYELLSFLGPAAGLITGNIFFGSLTVAVSGVLRKIQEQFSQVSPSNAQFLLHRTIRWHSEIISIVGETNRLLGTVFVVEYLLAMVYICFSGYMLLKVGSATEDVNLNKNIILCIVCIVMPLFYCLCGHVIVLEYDKMSDSIFQNDWVSLQPVDRKKLILPALLAKRGLSLHYKKLLKFDMTTYLKIVKQSYSFLTMLKLMENT

>AlucOR70

MARTDLSDVIQLLQFTGHYFSFKGSRREKTYETLQKLRVVFMVICNPFTLSSLFIGGLKKSMGVELFFGLMGFLTAMQHVYAYRHRKTTEDIIRSILEIRRKYQQGSDIEFQQNTRAIWKVVYIYFSAMTSLLVFYITIPKFVDILYGILWDDPVALRLPQSMDAYLDEHQHRNLKYATVALVSSSWSFVSTYSHFGLDTLLSLVGFYYSSLVKTFCNRLKLNTHLTSKELEGHIKILAAHHHELFKLSLKMRSIFGCPYAMQNNFGAFCIVSLVYALLSDDSSGLLIKVANLFNLMILAGMLTSTSYIGQHVTNEISAIFDALYDLPWYELSPSNRKYLVTMICVARDPFTIHFHGRAPLNLANFMAILNTSYSYFMFMRSTL

>AlucOR69

MGSVTAKMDKWEKDEQEEVMKLFKEKYGPLIQLALIFPSWKSSSRSSTIMIFVLHSVVLLFHWMMIMISIKRSLESEWNFEMLTIWIHFAFIVFFVFIVIFCVNNQRSTYFRQYQIMSNDIGHHRGADGSIYETDGCVSEAKNIKREMLLYMIIPVLIFLFASTIYGLPYISKWLEGMENPYTLAMVNMNLPVPAWYPFPTHAGLGHFTAMAGQALVALSVGVVLITIILLFLTNALRIKFEFRVICYALQTLFTRSTTLFLQMQHDMKDIGNSEHSYQRVIGSCLVDIVVHHRAVSELISIFEKQVFFTCALGYMVGTLGVGLSLVNILEAMKVGNYVSVLIFSMMASMETLLMFTISQIGETITEESVKLRHQVYDIEWHKLDTQNRKILLIFQTAITEPIIVKAGGVINMCLDTFSNIMNLSYSFFNLMTNTN

>AlucOR68

MSDRHPDIAKYIKLMQATRNWYFAEDSTSHPAVDLLKRCYYHVRPSLFVFALLANGYGLYYREGFGALDGNLALFPQALASLVTSSTIYFNRRHHRKLTMLLNQRFLDKNEPWMVEIKNKYVSAVWKFIKAVILYQEFVKIFYVLAPVIVDSILHHIFDYLETPFFFPLTFSTFLTDDDKWTGRYYAVMFLNIWSGFEIVANLQGFIICYTVMTVFSVVELVILTEQIKSLDFYRSNGEINEQIRMVVKSHNDNIALNRELKAFLGPACAFLSLFTSLVLTLIVFTTTVTNDLMVILAYAVGAYFYFVAGLLYCSLGQLLDNQSSEVFDELCNLPWYRSSPDVRKSLNMMIRQAHNSLIIDYHGHYMMNLANFMNIMKSAYSYFTILQSVTGSD

>AlucOR67

MPVSQSLWNMIEGKMEITEEFEQKVTETPVERTAEDVFSTQIKALQIVAMWPNFKQNSNMELFTRALLKINTFVLAYCTLALFVKGLLTQDLVDRSEAMDIFTLTTSALYKMIFFYTHHKEMDDMVNWGAALAHQVPPKWMQYTTFFSCFHNFMGIFSITFWGLCPIFKWIFGETDLDGMTLPINVYDPIGVTGAMYSVFYIVCDYGLLSAVQIYMASDAYLFTAIHLAIGGFETLNNKLRKMGQINFNKGPTVNDSMNEYLKDCVKLHTHILIYIRKIDRLFRSMIMADVLHAIISLSFAMLQASESKGIFENMKMAMFVSYCIVHQYLNNYFGQNLIDQQEILNKELLISVPWNDGSKEMKKSYQIMMAGCLKSVRLSAWSVYTLQYATFLEFVKSMISYSMVLRQVQDQTVKQP

>AlucOR65

MSFIVGYFEKILFHFDKLEEPRSEEITKLHRKHFSILLLVSSVDLNLGRKYLFWTLLHAVFNYVVLAAQTCVLMYSTFLLRNDFEIGSGVLNYGLLMIVAIGILMNMQYFRHEVLHISGIMCTGLFRYSDKTMETEDMIKFRKHMKFQRQLLIALAVYVATIGGIVVLGPIIDEKLGMGFDGTFDENGVNRRLPVPLNYPGIDTSKIFGFLLALGMIFQSGVETTLIYGGATLLFATACQFILTEMKTLSVSIQTIPHRAAKKYCRIHNVSKKSLDLKTIFDDSEFQDCITDCLKENIQHYLEIYKFTKVLETYVKVPLLLAVLVITLAIGLTMMKLNEDIVRIGATISFTSVALGELCIMFLIAVYGEYYLTMSQEVNWEIYFTPWYKFSVKNQKLIRQFLISTRNELCIFAWIVRMDMEMFASVMNSAYSFFNFLNISKTLEEDELN

>AlucOR64

MSGFHGFWDKWWYGDPPMDDKIFDAIHQEFNQILYFCGLFPDPRPIRRLLTFGILILNIVICVTYMYLLGATAVLQNEDFFTASQTAHFASCDLLSLICIISLLLNRNRMIEMFRASAHNYYDYDDDAKISEMREEYMTTKKQKTIILIGLPSYLSLIGMVCLMSRTIDEYLGGGSNETNIDGVYQLTPVPMWYPFSIQSEPMHWIAVSSQAIAMFSTATSIGGTAAILVIFSQSISLQFKIIIYRIRKAEKHAYQLYRMNGGKKLKNVQLYSDPSFLGFYNSNLNKLAEHHSILIRQFDSLYQVVKWPAGCALLLG

>AlucOR63

MAEDDYTLTDMAGVYLLPHSRAYMFWTGHWVGAVPGPTPVPIMVARALGGTLTYVAVFVVCWGSLNGIMHGSGTNDMPMNLIIISCSISSVHKYCVYTNQEQGLGRLGRWMKRVSNREKKNKIPKTITDHILKVCLLIFYYSGTLAAFMLLTKLALTGTTYNVLVPGLENVLLLKLAIALSFMSLGFEVVVDALILMNSLFTFRRELMRSFEEWRKLNFDSENPNQYKEELKERVQKHIELLTIFQDLREFNNSMFGYQVFAIVFTTCALLYGMAKETENSHKVFVQTMPTATASFLEFFILCWCGEDIKFGFEQIHRSIYDTNWYEASLEDKKSMTIVLEFSKNPIILTGFTVFKANLETFVESMRQSFSLYTILSEMV

>AlucOR62

MGWRKYLTEMREFEDEDVRKAIRDNYSILPRLNNTFSYIDEGWVPLAIIHSIAFIIFVDLYLYLFFVTCYLLKDDFVLVGVQFHYLLLALFGLVFQFHLYNSRKEVCVLHKIMAQEFFAYENNEILAEEKTKLKKHMIKQRLQLIPFMILIGMIGLFIVGVGPLIDNMVGAGHDSDYLNGVYMKTPIPMYFPFEIVDFTTHYVATGFQIITVAMLALTISGVVS

>AlucOR61

MDTFCGGFMGKVYYLKKHNIYINVYLSCRVNRDNFSSFYRDFRYFLSQFYHLMAALSAALLGGSVCIVFIAIAEHLLAQLEILCISFRNAIGFIPAPGDRVGEKLAYQRVKSCLQHHNIILKFFDEFQKYYSIPLFCMLAGTTVAMCTIAFVVTDPSSTFGVSAAFLSLMAPEVAFCICYCTYGQKITDMSDILRDTVYNAPWYYQPKPVKMALLMALNKTRTPMTLSAAGLKDCSIKSIGEITQTTYTYFNALQLFRGKPAYHRE

>AlucOR60

MNDIGGLALAKSGLNNMMSILGGFRGPREVRFKGTIYEHIFIAYSYFGLLVSHYHVICCYLTPIFMPDMSFKDAMFFAVPCITTTFSHLRIYYMAWNRSKFIQLLEMNEEASKDDYYEDELQKEIDGWAKQVRILQPILYFAVSAPIVPWGVTPIVNEVLGNPWGPRKAPIISWYPYNVQETHFWVFTIFIQTMAGCHATLSNVMFDAVFICISTRQLALLIHLKNSFSKIFQVIHVDPKGISWYTNYRAEAVEKEEIENDLTQRLKYGIRKHQTTLRLSKTIVFFLATRFWIICLIYELHMYLFFMEVVQVRKS

>AlucOR59

MFVFIREKELKRDPNAMIGRRLVQARFAIFAGIYPDFYGWRHYYFVIFLWIIHPGLYSYFVMVYVLSFVEGLRYMDVELLGQVLCLGTITVIYCLVSIYYIAKKTDVDELMMMAGKGLSNYSRPTTQEEKTILDSKEKSTYKYAIGSSIMFVSVSLLHMGFLPIRRGLKGQYTSITNDTAPINKYTPLPVWTPYVCDDLTTFLISYFTQLIPGCMEISIINACCILYIGLCQQLTGNLEILVNSLRQLPERGLHMFEAERGIVEKFTPELYQNEYFLRCLNTCIGENIEHQYNIIKFYKKIQSVVGFSILAIFSGTGLIISTAAYSMLLIAERERDTEIIITNSFVWSFNLFVYTVLLTLYCYYGQKVTDKNEEVLEALYDTPWIEADMAFRKSVIIAMSYSQLDMTLSAMGLISASLATLLDIIKTSFSYLNMLLAAR

>AlucOR58

MSFLLKFIDELAEEEDDELIEVLKNEYGHFLFLAMIFPRWKKPLASFGLVLFYLSTIVLHHAMLSYAVYLSLLEHNWEQVSFLTHLVILLSFAIFQPVNFNWVRRVVAHVHRTLAKDVGIYCSGRIYDDPVCIAFREKIRLEKKFYMTMSAFCLMMGGILWSGLYISKSFSDIEQSYSSSGLSLKLPLALYYPFPTDRGVLHYVILGSQLLVCLVIGFLYLICEVLLINLFLKIKYELQVIGYAIDSLVSRSINAVGVNENNPQKDVLVIEDTKLQRSVEKCLKETIVHYQKILGLLLIARANLDAPLAIVMMLGLLVIGISLLNMLAALKANYIGMFLTFGMLVCAEIQAQLLVCLLGSSITEQADILREKLYSIEWYCFDMKNRRILLNFQACFTKSFVVTAGGIAEINMVTFSWILRAAYRFFNLMRSTS

>AlucOR57

MTPEKFMGTFSVQHAILFSALFIFHTMYAIYEVTITAFFARSLLETVSHFYIATYLFAFNFQWYFMLYSIRTFHENEIFLEDFKCTQTHADFATRELDENVYIFTRVLFVSCLCWTVNSSTHIIGPAIEALISLIKTGQIDNVLFILPPVFSTPWWAQIIIYFCNAITMFGLLLYCLASYTIMGFKVLKLKTLCDILNEALRNDVEEESNIKAYIKDHQIIIKAAKLLNAQLRTLNGFMFTACYLEFAVQLFALTLIDPSGSYYFALALDLSSIFLILVFQCWMGTMITHSLETVANGVYESLWYSRGNNNRSEVILMTQMAQKPFVQTIWLGTLKVERATSLSLVRSSYAMYTLLNFFQDK

>AlucOR56

MSRYGKIEDDELVNSIDIWYLKRSGLWEVFNHYREHGVRNRRFTLWKIITLILFVPIGFFSLCGPFFTETDLEGMTLVILNPMTSSQTVIKFAILWYGIETQCRVLELFKRDFLTCVPPSMQAKASEILTKAAKKANKLANLGILTDVITVSFWNILPLLRSEYFRIELGITAFGTPLRHNKILGFWYPVDYDETPYVQFVYCYEFLSCVWAGFVIALLEGLVIHLVILLTANIKVMHHLLEELKTSNGTLNSETLLTYIKDHQKLVKISNDMRNLYNMMITMELSTGLIILIITIFNFFLSSGNGDLVIMFKFMVYLMYTLVEVTVYCYIGSDLETTSEDLGFAAYSSQWYKVGKKFRKTLQMLMVRTRYSLALKFGRMYPINLMALTNILQTAYSTSMLLYRATSQDEQKEEAQILM

>AlucOR55

MLHEWTLFQKTGSSNSSDKEHYDIKETKHFATCRKVLVMMGFVNDGSVIYKLRQPVFVFLLYSAPVHHLVPAFIDKTAPSDQILMSWSISMLYVLLCIAWPFMIIRSSEIFQLWATVRKGFYHYSDPFTAAERTILSRTNDIVIKSTRVSIIAYFCAGFGTFLKEMQPESLRQYKAPYPGWFPWTINSNFRFALALLYQCAICLNTTFALEAVFVLFAYHTIHFEGQLRLLTRHFGDTFPPGLPATVTYSAEYKKRTLRRLYECVSHHLIITGFHKQILSYFGICLLVYRVIVTIMLCILCYLTTTGISLDKFVQLLCFALALLYLCFIFCLKGEKVTQLSDGWRLTVYEVDWWNHPVEVQKTILMMQMGASKALKVYGVWKPAMYSHEGISVIGQETFSFFNMLRAM

>AlucOR54

MFEPSEEILRELKISRETYLENDFFLARLSGLCRWNRLFSLFYFLSMTYAVASAVGFVLAAFSEKDRDQVLENIHFVPLIFNMASQAASYHYTQKEYLQLFRAVDSGFFNYDGDLDIATELEITEVKSVAKNRKKKFGHFYSMLMLVAGLGQILKKPLLYVLRGGGTKPVDGENNLVWEAPYGMYVPYADHWISYLTGMFLGYSACTFVSITAVGSVLSYQYMSEELLAEFKVVEITFSKCLRRAQTMYENRKNVLKANGKTSQITMKDCIIHCMNLSVKHHQHTLRMMNVFKDLMFFPLFMVIFDGALVLCISAYLTISDDVSLNLRITMPSVITAEATLAFIFCYYGEKLTEATEDVGDSIYNSDGWVQHSDIIRPYALIVKSFCNIPNELSAAGFSSVNHNTFGNVRT

>AlucOR53

MSKFNEYWDNWWFGDPPMNDKVYDAIYEEFNIILYVSGLFPDPRPIRRLITLGILIFNVVNCAAYVYFLGVTATIQINDFVTASQTVHFASVDLVAVTCMISIVASRRHMIDMFRTIANKYFDYGDDFEIPEMVEEYRTMKRQKIIILVVLPSYLALNAFVCMMGRTIDGYFGRASNETYENGVYMLTPEPMWYPFTIHNELMHWMIVMSQATGAFALASAVSGSAAIMVLLCQSINLQFKIIIYRIRKAEECAYHLHRKNGGEKLKKSELYSDPSFMGYFNSNLNKLAEHHSILIRQFDSLYQVVKWPAGCALLLGSLLIAMSLLALLSGDGKPSILLLAALLIVAEVMNMALLCGQSESVQELGQSLHEELYNMNWIELNPAAKKTMMMMILQSKRPLVLMAGGLQPLNWEAFSGIMNTAYSYVNLLLAADV

>AlucOR52

MGFSSWIAQQVVTEDEFQIRLKKYGWLHYLFQFSLVNSCYRSMTSLLMYYISLMFSLSIVIFHLFCYIKTALNAYSFGRADMSVANVHSVVLGIFIISVLTSYAIDKTKTSAIEQLYLESFLSYENEYPAPVTLFQKLMTLAGKMGIASGGMGLFFNVYVAAPMIDFRFWKESCVVKGINFCLALPHYYPYNSEDGWSFYLTELFQLMFGVYRISVFCAVQVTLTVWPLHLIQELTRLKTSIEHLEERIKKRYHRITMRNVEKVNLITMCMDKTFNECAKFCINENISHHHNILKYHERIDGIMALPSFLAYTTGTLTMGIAMVKLLSVEGDTTLGGNLAYVTVLAAEIGFMLLISTMGEAVTIQAEEIFNEVSHIPIENYDLEFRRNVIIFMEGTIKPIALSSSKFNKCNMEAFGNVLNAAYSFYNVTSASAKLEK

>AlucOR51

MSVQGEVIASKAFKDNFGRYMVWSGMYSESKIYSGAIHVFFLVHTVFLAYTVILSLDDEKLMGESAHFTAFRVSAFMLMINAVLNKDNLEILFIKLGQNQVHDYQNTLSDQCKKEIQAVRKNCDERKDFYGTNFLRTVSAALVIFWVRSLMEYYRGHMDNPKSDNGVNKNLPVPTYLPYESHDWPGYQFALLSEVALVMMSYFLVLGHDCSFICFSEEVLRELEIIIITLREVEQRSDHLRKEMLYKISQKESVHICLKHSVIHHQKLIKIFGSFKRYCFYSLFFMLSGGAFLICLSSLMFTSEKISHQDKSVFMMFLANELFHIFIFCYYGEHIMDRSIEVGNSLYNSSWIRIAQYVKPAFIMVKLRCQVPLSLSAGGFITAGFDTYGNVLRTAYSYLNLLQATN

>AlucOR50

MGEKPYTMDKNGEMEIDWLTQEDKNHINFFDQFHCWSGMWRSKKSIQWTYFWLSQMIAFSLVYFYSFYFLLSELELLAHLIHHMMVAADDIMYIYLLNIHRIRVETIHDYASKTYNYDSGIVREKHKKLMTEQLKLYPKMSKFIFLTTVATAMSLEVNYLLEATYLKSYVTMYPMYLPIDLNHPVTYTIVVFLQHLQVFISMILCGGLMSILFVVWSHLKVELDTLTFAITHVDELVEEKLRHFSYTNPADKEKAKAEFYNGFCYHFARHHAAIKRYFGAFQISCKVTMTFVLISGLICFACVGITSVTENIGIKLKFFVVMVIQTLIIYSWSWVGQDISDKNAALQNIIGGTHWWKMPKTCHSTLKLMLVGTSRPMLLYTLIGQPNNIDSFMDMTASSYKIFNMVYQVKFSS

>AlucOR49

MIRNWQEEEEGTTLLERMGQKFLNGHSIYLGGWVLRFPVRLPPFFYLTCAFGLIVKMILNYDNLTLIIDCAHMMIHMIVGLQTTLLALKQKGNIMRLKTQLDNFGYVKDLETAGEKIKEECEQEAMELYKPFSRCILITINVYILFPICKLFTEAGRAKLSRVLVWQMWMWVPDDTWWGFTIIFLFELVTSLFLLLSVMYTVPYLACLGKMTVGHCKLLALQLESISKKATQAAFTSGSFKAALNHEIDECARRLHETHMLANEVAEVYKYYLSSFYYGGMFALCMSGLQAVSATENIEESLKFMGVLTGELIAIGLATYVSEGMIQAFADVRSSI

>AlucOR48

MSESVAVFKSLNLALKAVGFVDSASGIKSYLWTTWNFIIIFIGVEFIAITIPLNIIQDNDIYLKLECVLCIYLNLSMMFRFTVLTIRKKRLFSLINRIESLLQQQMEREFDKAFITALARRCGNWAYLFTFAMTATCIEPVVMAYVKYYFRGVEDPVPFEVNLPFEKKNNIHAVVWYEVFQFLGCSMIVISTNILFSTLSETTSELVKKIAENFEKIDETNADYLLKQTIKWHTEVIGITKETNDILGITIFADAFFALQYISIAGFLLIRVGLSNTTSFSKYFITYICVLTNPIYYCYSGHRVSLMGDVLYDSIYNNKWYRLAPKTSKNLILPLMVARRGLSWNYKALNFDMALYLEIVKQSYSLITFLKMMK

>AlucOR47

MSMSVWALTIGGLRSSLGIEMTYACLTIMTTVQHFHMYRNRTRTEAIIATFQEIRNTYQKGTDIEFKNYTRFMWKVVKVYIVMIVGICSAMSLPFFADLVVWFVWETPTAFRIPIGMDSMVDKEPVRDATYFAVVLFSNCWTILGGVTQMGVDTFLFVSCYSLSSMVKTFCKQLKVPPNSTPEETTVHIRLLAAHQQALYKLQTEIRRTFGFPFFVQNLLGSFCICSLLYVMSENGATLLSQFIYVFNMIAVLMILASTAHVAQHVKNTTSEVFEALYEMNWYTLRPSDRKYLVTMLGVARNPLCIHFYGLLPLDMENFMSMLNTSYTYFMFLKSIG

>AlucOR45

MLIHSDIDKYIKFMKGYCVWYGKSVTWDDTRFDLCRKYYTESKCYLSAVLLCVVSFALYSTDDFGLQDGTFIYWPICLMMLVLTSIATATRHQQDVLTMSLNDNFLENTESWMRAIKDQNINRLWKMLRFYTVYNNTISALYMLVPLVVDSILHYGFDYLQTPFTLALPLTPLLKYSNTWNAQYYVLTAFNFWSCAEMVFMLEWFLGNYLLLTTFFLTELIILKHQVKSLDFGKNEEWDQQVESIVNKHSKIMKLNVELRDYLGLPGAFISFFSSILLTFTAFVSFTSTSIPLRVSYGSGFVLYFGAALLLTTIGQKLENESDELFKAFYSLRWYQYSPNARKSLNMMMRQARTPLIIDFHGRYKMNLANFMQILRSSYSYFTLLREVAKE

>AlucOR44

MKSKVFYFTDDAPKSTWDKSTRIYHRLRPVLLVVLLIENAIGLYFLEGFGVMDGSIIYLPICLLLFVINSTAYFSRKAQDRLIATLNKHFTESNEPWMLTVSHKYTTKVWKYIKLILIYDKACKCMYLAAPLICDTVLHYVFDMLEKPFYLPTFITPWLPKDVTWGPHYYLILVFGWWGMLECLNGIMGFMIGYTLLVTYVLIQVVIFKEKVKSAKIDGSQEEVDAEFRTLLSWHVDNLQLNRDLKAYFGFTCAFQSIFLSVGLTLTVFTAVSSKVIAVKIAYGVGFFFYFGTGLLYCSLGQLLENEVQSKHSEKFEYDYATVSHTAHTRLPRSMQNELRKFHADFETVLFIFYLTTISNIVGLKFFLPSSPTFITVGTKVVGTKVVGTKLAS

>AlucOR43

MRGTMELDDEEVMQTLQESGLRTWIGVIAGFRFAQQPRFKGTLKGRIYWWYEILTDLSVAINGISQFAALLTPEFRMIDRCLMCFPAASCLLCLFMSNYPRFKRKNFRSLVEEYENSFSDSQYRHHLEEQIRKGAKHTRSVIMCLVLLEFISMFIFCLILPVLNEATGFAFGPRRLAVPSLWLWDPLAGFWNYMAVVFVQLCGSVFVSLKKIGFLESFFVYASRQICMFTHLRYNLGKITDPLIVNDDGKVDVKEFTGSNRYLMKRKLIGWVKNHQNCLRLFEDLVKLYEWPLLVYFGATILILCTATYVTSDNSIDAQTCVICGVFNLGIFFELLFICRTGDRIKHESEKLLGALNGKNTFLLKSDEYKYLKMILTRCQSESVINASGGFPLTITTFIAIIKSSYSYYTLLKKVNGQTD

>AlucOR42

MRMTENGKNSDPDSLEIAEEKATKYLYQRFFVLPVVGGVFGFHQTRWWSIFTKTVFTAYYVSVLSTIVTLSYSSYLNRDNMAVASGCIHILITAFVVLGISLTLQRLRKDVVQLLSLDEIICEYQCSEYLSNLIRKSDKKLRFLLIFWVALYGSSAWIAVIFPFIDVYLGYELSLSNVTDVYWKGLPFASWWPMDADNSNLAWTTCFMSQGLYAFFAASSATSGMLCFAIFSEDIFNHIKLLVNSIERLEKRAKLMFKMLHPGKSLRNSLDEYDECYYNCILQNVKHHQKIVIKKDLLMKIANIPVAVPFFGGAMLLGLAGINLLSDGDIRIAPKVLFTCLGVTEAAQMFLLCHYGEQFRTQSELLFNATFYTKCYRRSMKCKRAMMIFRLGVSRPMAISAAKLIVLNMGTFANLVNSAYSIFNLQSITTQED

>AlucOR41

MGCLQAKIEEWSDAEDEEMMNSIRRRFGLFCQLSLAYPSWKPGMRRWTLLLFFIHTVLLTTHSVLLGISGVLMVLEWNMELASLTIHFSVILFFAIFIVYWMNSQRPLYTRQNMLMVTDVGSYKSGRIYDDDFCVEERRKNKRELLYYITCPVFISLTAGYVLTVPYIQHWLYSSGESPYTANMVNKHLPLPCWYPFPTHEGVLHLMVLLLQLAAALCGAIVLVAILLLLIFNTQRIRYEMRVVGYSLTSIFHRAKKMFLEQNPHRKGDNLRDDPGYQKVIGICLKDTIIHHHAVSEILSLFTKQADMPAALAYTIGTGVIAMCLFNILMALRDENYTSVVLFSMLVFVETLVMFVMSLCGESITSESVNLRHELYFTKWYNLDIENRKTLLNIQTNLVEPVIVSALGLIELNMN

>AlucOR40

MIPFVFKRDDSVDHEVVKGYQSTYNYIMRFCGLYPDFRGFWYYISGAHLNTVHLAYIWFLAAYMISTYYAFAYRDMELLSYELCYGLVTLIWFTVTHYTIYKRDQLDSLFRKVGRGFFTYEKPIDSEEEAIIDECNTNCRKTFQKTLALTTILAFWTCIIPPLPKAVMGDYSSIVEGGVPVNKHLALPTWNPYPTDTHLTYWTMWMYQALAGCTEAYIIGATCILYCNFCTIINRELKLLRFSLGNIKNRAIHAFKMRGYSLQLGQKYENSQLYQVCLVHCIDESIKHHIELKQFHGAIQNLLGFPIFAIFSGSALTISSPMFMFLQMIGEHEESSFTLVMNIFQYTIIIFGFTYFLANYCLFGQSITDESALLHFAFYDTPWPEAGLNFRRKVLMGMIHSRKPFVLTAHGLASASSETLVDMLKTVYSYFNLLAAT

>AlucOR39

MGYRVYPQQDLSDPSHMFSFQLNALKSTTMWKPDNQKYYIPFMILFAVNVFVLAICTVGLLLKGCSTKDLVDRSEAMDIFTLTGSALYKMVFFLYHYEQLVDMVTCGLALVRNLPEGWTKNCGLLSRIHYTAGFLVLLIWGLAPILKVMYGETTWAEMKLPINTYDPFDSTGFLFFLFYITGQYVLVLSAVIYMAADCYLFTSIYVAVGALQYIVDQFENMRDLNNNNKHTVADTMHDCLQECIEIHVHVLDYLRKTDKLFKSMILADVVHAVISLSFAMLQTSESKGIFEGVKMVLFVQVCFVHQFLNSHFGQELIDKQDNLAKQIITDIPWTDASRKFKKSYYIMLTCVREPFKLSAWNVYFLQYATFLEFSKTMIQYYMVLQEVQDEAEVS

>AlucOR37

MTPKVGKSEYVIKDTKVFATCRIPLVSMGFVDDGTVMSKIRKLITVFLMYSAPVHHILPAFVDETINLDGVLIGVSLLMLYILICVSWPVMFRRSNDIIKLWKTVQKGFYQYSDPLTKEERTLLSETDDLVIKTTRISIIAYFCAGFGTFLKEITPSSLRAYKTPYPGWFPWTINSNFRFSMALLYQLALCLNTTFALEAIFVLFAYHVIHFECQLRLLSQHFKDTFPTGLSATVTYGAGYRKNTLRRLNECVRHHLVIKRFHEQILSYFGICLLVYRVIVTIMLCVLCYLVTTGISANKFLQLLGLALALLFLCFIFCLKGEKVTLMSDQWRQTIYEVDWWNHPVGVQRTVLMMQLGATKPLRIYGVWKPAMYSHEGISVIGQETFSFFNMLRAMK

>AlucOR36

MLTEYNLRFLKVLHYYGFWITFVGWKHDKIKKICLPVRGVVIGLSSILAIYKLGQEGIQCIINGTVVYFPFMVYTFTIPILLFKNRKHFLSLLLDFEDCWKFFCEEKERKVLERHYKRIWKVANYVHICFFFEIAFYAVMQLALDSVLHYVFDYLSKPHVLTYPHMGYLPTNRTWDGLYYVVAVVGCYNLVETLSVQLGWVILFVVIVAYCYPILLLTEKAFKNLLQYDGDPDSAALKKAVQSHQLLVKLNKDLKAFLGLPCAFETLFVSIILTLTAFTSVTSTDALVVGAYSSGFILHFLAALLYFSLGQLLENKSEELFTILYDLRWYTFSPAVRKDLNMMIRQARKPFVIDFHGNYKFNLENFMQILSTSYSYFTIIQTLTDNH

>AlucOR35

MGLLFGRTPFYIKSGWLRFSYKSLPFVYALILAFANWFCLLYYIDHLHNTWNQTLGSNVSFSGVLFAILVFSQPFSTIFMVYSWAFEVPAIVRGYNSTAVLEEKISIVFPLYKQKPSKRNLVTFLIAFLFVVDLIIAYFLLKTRFTETPLILILLIIVNVVVTFSYCVLWCFNCYFISDLAVKLNKYMLQCLQVRENCAFKIKTCRKIWISVWKQSQMNSQSIAVSLSFALVLYGMIFVVGCYGILTSIRNQNILETLEMSPYVVVTFTIIACVFETSYQASHKLGATFLDTMIILDKDRVDHECVEEIDKFVDTINRTRIAAITLKDYMTMDRTLVVSFLSYSITYLIVLIQFQDKNEEPSVNISTPMRNNTL

>AlucOR34

MVTVAVLTLTYVFTHEVVTQTDFIIAINSYIVSMTFCAAAFKILIFLVLQKEFKKLFLMVEELGDLDMYAPSTKDHFFNCYMYVTLVLTNPCTWSLWHLIAHNDIPFKSQYPWGDDGVGYLLSFFFGIMAAVFCGLSHILVDTSFMMVIAGITLHVDKLSQSLSLLGKHRFKDSKIMSAGIDKHAQLLRVSQHLSTCYSNLFVGQSVYTVGHSCVLLFGAVHVESKVEVVMSLGTMLVTSYCQLLVYCYYGELLTSKFSDLVFDSYNNAWYDSDLQVKRALPKFSLMCHRHVSLRGFGKVIPSKSNWLHSLQESVSYFLFLKTISGEE

>AlucOR32

MSGDISRFKAKSTTTNESLMREEYIRKGIDENNGLFLIIGGMYTGYLPISILHAILTAVHIPLLLAAVIIGRNDYVVVSETIHFIILLSLAFVISMRYLSVRKKLDNIFEAMGRGYYNYEGTLDPGTEKEFAIHLKESEKRKSVLKYVFVGGCIGALICVSILRPVLQYYLKKYIKSKKLPHGLNGAKNTFIYYPWDSSNTWLNFIGYFLQDAYTLMTANVVFGFVLMFVSTAESVVVQLDKLKLSLKRIKIRAAFIASINHEDPATNNNKDFRRALHICIKHSIKHHQLISRIFDDFKSINYLLLFYLIGSLTFLLCMSAVLFAADDVSLISKATFVFFITSELVATFLVCMYGEHIAGMSSSLPMDLYNTEWYHFSNELLIYYRMLAMRCTRPCQLTAGGFSQINRNTFLEVLKTAFSYANLLQASKQK

>AlucOR31

MNGNGAVLNGVTESSMPIKRPRKLKKVPNKRSIQFETDRNEKKVEKFLSDEEALKKGFDENQGIYLVLGTLYRTSLWSWVHTVLFSLTALFMMVCLGRVAVLISDDFSLLFETIHYITIIGGVLVILPPMMRDEFRFEKIFKTFGRNVYSYDMLDEETAQSVQKLRAQGNREKQLLTKAFTVMLLGTFAGFSVLLPGMYIINGKFFERQRDDGIIMGIPCVLWFPARVGDDWIIFVRVFLLLIEEYAAFTVVAFIIGQQTSAICIGHTLLYEFKVLSLTMEKFVRRATKLAEGKKFEGIRINSEKQLYEKLTACLKDSVKHHDILLDVSEQYKSIFYVPELVILLSSTMVICLSAISLTSDDIPLEAKALSLILTGAEMVNVFVNCYYGQVLLDAHDELGDAIYGSGWTSCSTTVRQHILIILSRVQRPLSLSAGGFAAVNLDTFAQVVKSSFSYFSLLQALKE

>AlucOR30

MVEKSNFHVKRAQLFKAYNSIHWLTLTKWFYEDYPVEKLWSDKRLWIHLSIVIICQSSITMFKVFHLISEENFFIFLTSLTSFLVIVLVAVRTYILYQFPTFRQLYFKPEVFNCNLHRPTRSLALLTEAITHSRKVGMWCLVLFITFDVAFLVLPIVPPILEIIDGTNKTYDELIPQYPSINPVSLSWLSKELKYAFDLVMAVFNTIPWVGFVVVYYTVVQLFRAQHKIMMTAMLPGPPVPGDGREPLELKLWIQDHALIRKLVYKLRNTISPALAGTICVNVFTVGLNMLALVSSPIGSDAPMFTRYLYYFSFGTYSALSIFDIFIHCWLASEITNCGEDLSYALLKSDWQNDLKRSHHHYVLPLMLCKKQIRFTGLGLIPVTLTTFTETIRVSYSYFTLLRKTDD

>AlucOR29

MTEAEVKDGTKKVDDKLGCIDYRKYTFARMIMIDDGLAARGLTFPLLLIMVVNVGMQTCSFISIFTSTQTSVSLDNIRACLLGTSVTMSLFNQFISRQAIARLHGFFDKSFRTISTNPNFPEEKIILDDARKAASSQLEMYVKMFSCNAVAMMFAQPLGELMSGHSWRKLPVQWTFPPSDDEFSFWLIFAFQFTGICIAHCVGIMIMSFTSITIQMTALFDLLIFSIQHIEERATARGRQSGGDRHACLLACLTDDVDFYQQLIREMDSVTPHLRNTFLIISAAVPMVLACEAYPIMQGNFSFADLVKSFLFLSIQILCWAQTCSRLGTMTDKHAAVFAALYDSPWYEAGMKYKKLVLNSLTYAVQPKYIKARLSNEVTASMATFYSFVMSAFNLLNMIRNIG

>AlucOR28

MAGYGRLEDGDIVDGLSIWYLKASGLWEMFNHHRETGGRSKVLKFWMAGMIIAYSPVFVVSVVGPFFAEKDLEGMSLVVLNPMSTVQMVVKFGILWFHMEKQSRLLDLMKKNFLACVPPDKEAEVSRILGDAVKEANIYTFFGTRINIITVLLWSILPVLRSEYFRITLGITIFGTPLRHNKLLGFSYPFDYDASPGNEIVFVYEFLLVLSAGLIITVMECLVAQLVVLLTAYLKVFQYFMEELKSTHDPKFDKEQLLLYVKEHQKLMRVGDEVCDLYNFLITVQLSTGLFILIIAIFNFFLSSGNGDVVVMIKFVVYTLYTLVEICVYCYAGSNLETTSEDVCFAAYSCEWYEMNPDFRKTLQMMMVRSRSPVVLKAGKLYPLNLITLTNIVQMAYSTSMLMYQQTHN

>AlucOR27

MDGNIERHFDHMEKFLKYYWQWYGYPDTFKGRLITIFNIFRIFLLMVLVGIAASQAYFYGMSYLVDGSAIFLPLGIMSLVLNSHQSWNFGSIVKTAKQFELFLSSFQDEEERNLIASRMKERKRTVLQIVLIIEFYLMAPLMCHAISMTLHYYGFLKKPVLIPLLFEMFLEGNYELGPKLIATAVVSVFYVHLVANIVTMILLNVHFLGLVVACLEVLTERLKGFAEKTQEGYGKLKEELDLRDTIQQHADLLHIINSFNSWNGFLVTFCLAACSITFCLDALTTKRALEQEIYSGACLWGSFLLVMMVLSYLICDSGSQIETKSEELLRAVYNLPWYRGSSETRKAVWMMLTQGNRLIILNYKELMDLNMVTYLEMLKRAYSYFMILSSIE

>AlucOR26

MGYVWSKLQLKIKLWDSWEDEFSIDVMRHRYRGFHRIGFIVLDLSSKYAKLSIITALLGTSFLLMATLSLLATCAKMSDDFEECSGVCNLGFLSTLALAFLVNHNYFRKTILSAHHMLGKGFHDYQEPEYSSTEFEKYKTMLRKQNVALIILACYVALIGILVVVVCPLIDESLGFGWTEPYDEYGVNRQLPVTLWLPFYSHEGINHWFTFLFIEGLGGAMICLSIGGSALLFTSLTIGTMLEQKRLVLSIRDIEQRARHRFQTQYKGKPGVDDEGKNVAL

>AlucOR25

MSFLFKFFAYLAEGEDDELWDTYYNFYGPFLEISFVFPSWRRSRIPVSLTVLGFFAFTFPVHLWLLTLGIEGVRDDFNLASMEFHYWMLLMFSMISIFLMNSHRQNMIDFHRRLSRDVGFHGPGRIYDEDYPMKLEHNKRKQLLQFLFLPGLVLVLAGATLILPYLSKMDGTVLYNSREVNMKLPIPLWYPFPTHEGILSIMAVLGQFMAAGGLSAVIMTLDLIIFRATQAMIFEYKVLRYAIDTLVPRAKRLYAQEYPMMDLESVKMSDDAFQICIGKCLRACVIHHQDINRLLNLYKVMLKWPGFMAYGFGTAVIGLSLINILSAKQNGDYENIVLFFGLSMAEVLNMLMMSVFGELISTESKELRQELYFIEWHNLNTFNRKLMLGFQMGLNNPVIVKVGGLVTVSLETFSSIMNTSYSFFNLVNAQ

>AlucOR24

MISLTIGVRKQLQSTTNIMDNMFSFFAATPSFLGMGKIICLLRQRRALRRIWKSLDDLLENVLKRDVDEQLEKELRWRLKRCWAMYSIFLTVGTCITLHWLLRPIVYALYGERTSIVSTWPTYLESWIQWFTTYIFQAMNISSIGHALYIYDNVYFCICENLLIHFAVIKHHLHQMDISKGKPGGVTMKFCISHHVKLMDICMELRECSKYVIMQQVFWTIFILCPGVFELVSGRQTDTTILFNLMEITTIMTCILFFYSWYSNEVTLQSSQVFNTCYMSNWVEGTPNQRRTMMTMMTRSMKPMIFGGLVNVDLGTFISCFFRC

>AlucOR23

MWLKYPSDEELNRLGICRSTFDGNVFFKAQLGGLCWFSNSWSVFYFISTIFGIFSGCGFAYGVFMEKEWEALYEALHYIPLVVNITSTAASYHYTQDEYLQVFRSIDKEMFDYEGTLDEQAVEEIARMKTEARARKKKISMIYTKLMIVAFICQTLRKPLNYIIDGRGKKDVDGENNLIWDVCPFGIYMPYADYWAPYLIGHFLCWSCSAFSAITAVASALTYQAICEELLADYSALDLTLSTIVQRAERLFSNMNRGFTGNASGTVTFDYCLEKCLKVSIKHHHEIIRLFNIVKKLLYIPLFFTIFDTGIVMCFSGFIMISDDFSPKFKLLLSPVMLAESGIAFMFCYYGEELTEMNKNIGNRIYFSQDWMKHFKSIKPYALTVKSYCDIPNELSAGGFTKVNRLAFSNILSAAYSYVGLLLTTS

>AlucOR22

MIRLRLPPTINQDTSQKNIKTIYEELDSQFHTGIRFLLMGASPEKFLGKLSLTYVLIYLSIILFFMLYALFEIIVPFFFPGDFLEFMNHIYFGSYCLAFGYQWIYLLMNLNNIYLNRLNIESLYSTQAVSGIAEAALRKNLRPFKIAIKCSIALWSITILAFGLGSLIEIAVEYLLTGEVESYAIMSTFPFPPWGQMLVTALNFVTLNMGFAEVVAMAYISSLLALEIETQCEILCAAMLQDRDDWFKFRGYISDHARIIKNAKWLIGILESLNAPTVFSAYILMAIEMVVLTLVEPDGLFFVAMASDLMGVVIILIFQGWISSKITLSLQSISFAAYQTSWHEQDKNKALDLMIVTQMAQRTYVQKILLGTVVIERGTVLQIARSAYSFYTLLMVLQSNKTL

>AlucOR21

MFLVFMVTVGILFQGAKLLWNKNETTFDLLFSLVHGRENHCTYTGTPYDEFLKSRRHTFLQYGPTLFTYTNITVMSMYVWLPPLVDCIMGVTHHPNDPHNGIATTLGLPMWTPLDADHSWTSYFIVIYMQITFFRIVTVSWAFGIFYTSIFQLTLLDEMRLLRKSLQEIDTRATQLFTLKYQRQPVNRKTKEYDDCYYECLKENIIHHQFIRKIYGEYQKRVGWTIAIPIYLSSVVLALSSSYIMLDNRNALKRSMDVLRIIGDSLLTFLTCHAGEMIAYENDELRNELYNTEWYDRSKKVKQAISICLHITYMPMRLYGGYMVFNHELLSTIVNSAYSVFNFFRVIQKSAK

>AlucOR20

MVIQVRNMEQGLKEITKQYYAAAGISGISAYLKNDKPFFLIRLWVWISTWIILPQVTLAHIYTALFTGIPLATRFLSLSLGLDQLQTTIKCHYMLHNLNRFRSIMLDLETFNCVNHLGTEALTTMLKMCKLVKWLRSTYNFACYFTFIAWTLVPVVSTPKALFYAEDGVSMMKVLPPEYPFSTDYFPMVQLIYALESFSTFVILTYFASTNLIMVTDILLICQLFRVLNESVAPSKGRKAMTLRMFAVDHQKLLKICAEVRDLLSPLLALQLGISVMTITLAVFEITMVNQASSEGGINQVVLMSRKTSYTFIIFVELLLYCWLSTELELSCLSVRNGLYNSEWYKYEQLGTKDYRNYIMICMRALKPVRLTAMNIVTLSLTTSMEVLRMAYSYYTYLKKLR

>AlucOR18

MSFSFVEKYQLSPETEKTMVTEYSYLLYVGGLLINYRPKVWIISIAQTSIFIGLITSYTIIFIISTAKSSNFVAFSQNLNYASLCCICLGLYFAGLSHRSAFVRLMEIIHDDFYDYGDSFDNAEVAMWKSSLRTFKIIIVVGIPTYLIIIAVSIVLGDYIDTALGYDSTDEDYLGEIYQKAPLNLWYPFVVTNMFLRVAVTLSQMTTAAILATTLATGDVMMLFLGQTVALQLRILCLAATKMDQRANLMYEKGLARSSSGDKEDLDGCYKLCIKQLVQHHLIIKEFYKTYYTIAKWPTAIAFMNGSLMIAMSIIVAMNGNEETPSTYISTYLLLVAEVLSMWLLCETGQNVNTWSEKLFMDTYEFNWNGLSVPNKKMLLIFKENIKKPLLMMAGGLTPINRDTFATIMNTSYSYVNLLRASERRSND

>AlucOR17

MSKLKNLPFITILWREFKFLSVMGGTYGFYHTKAWTAVTVINYIVMYSAMLFTMSVLVYTTYLLRDNIGYMSQALHLLIVGCVTTTASFTITINRYKVEKFVVFFEDPWSLCEYSRNDFFEELMKETQKKKTRLIVTWILIYGICGVIGLLQSGINTVFGTQSELTDVNGAWVILPFIMWWPEDITASTGAWMRAFLVQSLYLYFCVIMVISGVVFAFSAVERILDQVKLLIYGIKTLDRRAKDMFQQKFPGSDMKYMEKEYDDCYYECLVQNVKHHHKMIKWIETFLDMASLPIAVPFYGGAVLLGMALITITEKDDPRVGPKCLAACLAFSEAYNMYLLCDVGQRLENLSQELYDTLYFSRWHTRSPKVKRAIQIMKIGCQKPIVFTAAKLLVLNMSLFADLVNSAYSIFNLKAASEKFEDK

>AlucOR16

MTLLSFFKDKFKWNEPLGITETTATLFGAFVNYAPSPGYRKFFRFFGWYIIFMFIIFNINVVLTIYFASDFFEESLEAVRLFVTAIHILAKLLTMRAMEKQYMELIEQIRRAWRTYEYSSGDMLNKTLAAANKGTVIVFVAIGNTIPINVIVAALKNLGNPPEIQFSMQCWVPPSLRTSFLAGSFYQLTPYFFPVMLYCMTISFLNSITLHVEALGLALAKEIRSQKEWRDEAARSLYIKHQEVVRIVGRVNDLMASNWGFEMMCATLQLTLVSYNALRTLKKNDVAFFNQANLMLVNFLVIYFIYGNGNRIIKMGEELHNSLYDTKWYTSTVKERKNVLFMMFRTSMPMEYRFKIAHFDLPSFAKLVNTVFSYITLLRSVDEPEEQGAF

>AlucOR14

MLTVGSSAHNSLSRILEVIGITRYKEASFFSENSFKLFRVVQWALNVLMIVSCLNFIFSESVDDSPDKLQSLALCTADIQFLTTQLILISRQSLVDDLVAHLRSVYHGIKEIPGSMDILAEGDRQARLFIMSYGVVIFSNVPSSMFFAGIKMILTGETAYPFPMSIFGLPTAVGWALQMLMIANAANILWGFYCVLKTVIYILGAYSNVMAHMLRERPIDVEAKNDRKMLKLHCDINSLSLKLVNVYGLISFLEVTVASGRCCFVAYHILLAVQEGDYKNLGVALSTLLTSVAITYVLCSCGEEISMQSVTIRDGVRDSKWYAVSPPARKTLLPVLLFTQRPIQFHYRRFVYFNLETFRNVLKTAYTMTTALAQV

>AlucOR13

MAFIMRLIDKCAAMENDDLDRLLDNYYGPMFKLGLVFPSWKRSALVFTIPWFILNVSTFTWNLILLGITVYKAFLCDNDMDLFSLSTHYFLLLLCGSLIIFFMNWNRKLNGLNHTRISVDVGKYKDSRLYSHKDCILMEKQIRVESYRYLCLPLLIVLICGAVLIVPYASKLFRGVGTMYTTCGVDMFLPIPLYHPFPTHEGLNHFLALISQVLVVCCLANVIVAIMLNFTQYSLRVKLEYQVLGYSLDTLFARSKKVYLKNYPNEKASFHIRNPDYQRIVGSLLRDSIVHHQTLVDMMDKYHGLITYPVVFAYLTGSGAIGLGLLSIVRALQKGDTETLLLFSLLMLGEVISMLTMSLIGESVTEATIMLRYKLYDIRWYDMDIPNRRSLLNFQTFITEPLVLTAGKGLVNLTMETFSSIMNSAYSFFNLVNIQQSE

>AlucOR12

MKFIDKLAEEEDDELIEILKGNYWHFLFYSMTFIRWKRPRIAIALISAYAIWIIVHLVIGIYSIYLAADERNWAVVGLVTHHMVLGALAIYLPIFCNTGGFREVMADMHRTFTTDIGQYSGGNMYAEQACIDIKKDVRRQTFVYYINPALVAAAGSLALAGPFLTKWFSGMENPYSPNGLSLKLPTALYYPFPTDSGVVFYAIVLTQVISGTILGYLILAPQLVFINLSQNLKRELRFVGYSMETLVRRAMRMTFENNVWKRKVTELDVDDTEFQQNVELSIKETIIHHQKASKLLSTAQVSVKGPLAASYIFGLVTIAISLYNITLALKTNDIGSLTTFLLLLSSEVIGTFINCGLIGSELTEQSEDVTEKLYFIEWYNFSVKNRKMFFTFQTAITQPYEIKAGGVTPMNMETFSDIMNSAYSFFNILQTIE

>AlucOR11

MFRGKEKERPYVIKDPKDFDTDRLVFLWLGFVYDGSFFSKVRLTVFVLLLYSAPIHHMLPVILDKSTTTDEVLIALSINMLYVLLCIAWPFMIYRSPEIIRLWSTVRQGFFHYSDPLTSYERTILSKANDLIIKSTRMSLIAYFCAGFGTYLKEMSPNSMRLYNPPYPGWFPWTINSNFRFAMALLYQLSICLNTTFALEGIFLLFLFHTISFEGQISLLKQHFEDTFPPGLPTEMTHVPAFKERTLKRLKECVRHHLVIMDFHKRILSYFGICLLVYRAICTIMLCILCYLTTTGIALNKFLQLACLAALILYLLFIFCLKGQKVSKMSEIWRETLYEVDWWNHPVEVQKAVLLMLVGAGKTMTVYGVWTPAMYSHEGISAIGQETFSFFNMLRAMK

>AlucOR10

MDRHEGPLSTHFRRLFRLVGVYNGKYITPHSCLFFFSGFVNIIYLSYILIFTDCNMTKVAHFIILQYFYIGTLWTVVYKGNDIIWIANECDKFVGLDGHHFERLYDEVREQEKTSPATKGKTIIDRIASIICTWTCIEPFINAWTGKAELEFPFTGTNADTTKFIFIYLMQCSLMFIVAIVCSVIFKSLMGIALDLVIKYKVIGLVLSSLNEQMMNHRNVYKSDLHQTIRKCVQSHHHVLRIFEKYRDICTYGFRYSYVGLIGATSLSRSLLSSDDPDLGTIPHIIAELSYMGFFCYILNQVEQEHDKLKDAVFAADWPWLPKPATSSLRLIMMRTAKTPRVILVKGGGPANLETFYKLLNGTCGYLIFGLVLDQAAF

>AlucOR9

MNLIGLPPREDDRKAVDYRKCTFFKILYTENETGGKVSMARGVFVIVTLSIMATCCLVSVTRSQTAEQLLDNLKGMHLEVMVLMVAINECVSRKRMRRFMSYVERFRANPRYDLPGEEAILVQARSNAIRDITFLVIIFGANFPLMMLTKPVTEALGGGSWKQLPFPWTVLPDDEDTTFVAFLLLHTLGVFFSHCLGVVGMCFSTITTQITALFDVLLLSIERIEERAARKMKQLGLSYQESMLICLKESVAHHQELVQEVRSEKPHLESQFFSEIVNISTIMACEAFPLVRPNLTISIAIKGLVFLVVQVLCTAVLCDRMEIMADQNTEVFAALYNTPWYKCGVEYRRILLNGMTFCRHPLTIRGKSFLGLIATRATFYTAMVNTFNLLSMIRKMG

>AlucOR7

MLFGAGSKELGFRKETIEEEDVGFMQLAGLYPLTRGYTAYYFISFILCTTLMEGMIVGSYLEGEVDTSLETAHVLLISLNMFTQICTHRYYYDIVNKLLRAIDDNFFSYGDTMDEDTKQVIRKLAKEKTARKKMFGKVFKIQVSSAAIAITFQRPILYVLNGRGVKDVDGENWLIYQSPFGILVPFSNYWVPYLFGMVLVVNQVITTSITAMATATSFVRFSEELLHQLEIVKLGLGNFMFRARHLHSLRYNQSKESGEQDKHLDKCIITCLSKSVEHHAIIIKLFGDFKNMMYIPLFTVIFDGSVLICMSAVQLITSKNPIVRMSMPPFIVAELYYTYLYCSYAEKLTNMVLRTAYSLLNFLATRK

>AlucOR5

MKIVTSDSNVQPVGSTSESKMVERKPQNLTKQLSLSRNPVRTRKERQKRTKTIYDQLDTLFHVGVRCLLLGISPDKFRGNKGYERFLIYFVVILFFFAYSLFQLCAPYFFPGDFLQGMRHFYFGSYCLTFGYQWIYLLRNFDKINQNRVNLKCFNSSRAVTEIAEVILEGKLKPFTRRIVCIAMIFSIASFSHGLGELIQFGEQYFIMGEVKTMLTYFPFPIWGQLIIVLLNTITISLLLAGTVSALIITGLLTLEIETQCAVLCAAMVRGTDDGSQSFTGLIADHVQIIKNARWLIDLMESLNSPAFSCFYFHIAIGLVIMKLVETNGYLFYVSMAFGFMSVILQTGAQGWFSSAITHSLESIAFAAYETNWYERDKNYARDVLMVIQMGQLRFVQKIFFRSIEIDRATVLRIVRSSYSFYTILMIIQ

>AlucOR4

MVLVPYLKPQKERNAAVDRGYDITGMFYARLAGLYPDLEIGWRYWFFGSYQILVVVAYFYYVLAYVIANVIAIKYMDVELIGSTLCFGSYTYTYALIALTFYIKRSKIDKLLEIIGNELYIYQCPLSQKQLKIRNEETTRAKNFGRYSFFVPCLVALTHMSVVPAIHGFKGEYSSIVNGSAPINKYTPLPVWTPVQATSGMSFFVVFWCQLCPGFVEFLIFHGSCTFFVGVVCVLVSEIKILLESLNSITDRAKYLYHVKGGRGSDIDNLYDDPIYQQCMVDCLKENVKHHIKIKEFRNLFQDIISYCIFFIFGGAAVTISTPPYTILKIMESGDTDKLYSAGVVMMGHTFLSVYLLSRYCKFGQNFESENSKLLEAFYCTPWYNTNMDYRKILIIAMSNSQKTLQIKGSVVGVSLSAAAFLDVIKSSYSLLNFLATAGS

>AlucOR3

MSAQTIQSDGGRALLNRDDVKGLNMGLNTFGAKTFWHTLEHFHATGKRHWVMATYIVLLHLVGFTYCLLGFSAVFFIKMDIKRGTAAIMNPICGLQTVFKCWTFSWSTAEYLKLFEILKKDFLTCVPPQKEGGANDVLAKNVVATNEFVKNAMRWNFLTLCMVSTMPYLRSQAFREFFRLGEGAIVPNKICENEYPFEWNSTPIYEIIWIYEQIAVILAIVTSSAYQAILLFLVMALVGHLRVLGYVMENLRASDFRGETYQLMDKSAKANAYQQLIRCIRDHQKINAAGDALAERYNFFLTFHLGTAIIVGIIAIFNCTAADELADKIKFAIMCGYGLLEVAIYCYCGQLLENASEDVLRQVYQCEWEEMEPKFRKAAQLMMVRANNPIALRAGRLYRVNLETLGAIQQLVYSALTMLSSMIDGS

>AlucOR2

MLLCWKKKKEKQKPVIKEQHDFANNYRLFKYIGMIQDGSLFSRIRVFLATFLLFYAWFHHLIPLVNSTEEYSFDELMDLIHLEMVYFLWCIVWPSYIIRAPLFTSLASKIQNGLYTYSDPLTLEEKTILSTANDAVVRMTKISVYVYVCGGIGIFLKGMNKERMRKLQLPNIGWFPFAINSLSRYAIGCLCQAIMGINAISIAIGTFMSFAIFLIHYEAQFKLLRTHLKRSFPKNVPLRIAQTDKYKKVSLRRLKDCYRHHLAILGFHQEIMKYYGILLLVFRVAIVMWMCTLAYVTVMVDVNAHNLLKMLSFASTELLYVFLFSFRGQDVTDWNYQWREELYSIQWWEQPKEVKTNIGIMVLGTTQPLLLYGVWKIALYSHEKLSDIGNESFSFFNMLRAIN

>AlucOR1

MLPGFILLVANCVIMVPYISKWVVGMKNPYTPRGVNLNLPVACWLPFHSHTGFWHVVAVSNQLIAVSCLAVIIITLLFMFLKFSQKVRYELKVLHYGIETLFKRSKRLYFKMYPERKAIRFHWTDPVYQRVVGICLKDSILHHKTIVNILDVFMTMVSIPAALAYVIGTAVIGLSLLSILNALNQGNYPNVILFALLCVGEILNMLVASLIGETLTHETIILREELYFIEWHKLNLSNRKTMLNFQTAITEPLSMKAAGLVDMNMDTFSSIMNSAYSFFNLVNAQ

>AlucORCO

MQKVKMHGLVGDLWPNIRLMQLTGHWLLEYHEENGGMLRLLRMAYCWMTTFSIYIQYAFLVCFLILETYNADEMAAVTITTLFFLHSVTKFTYFAFRSSYFYRTLGAWNQVNSHPLFAESNARHRATALSRMRKLLMIIGTVTILAVFGWTTVTFLDEPVWDKTDPDNVNETISVEIPQLMVYAWYPWDARYGMTYFMTFVFQLYWLFITLAHSNLLDVLFCCFVIFACEQLKHLKEILQPLMELSAALDSVVPNSGDLFKAGSAGSDIALIGNGENGNDFDVRGIYSSQRDFSGFQGGVVNGGTVGPNGLTKRQELLVRSAIKYWVERHKHVVKFVSSIGDTYGSALLLHMLTSTVTLTLLAYQATKIEAVDVYAASTIGYLVYTLGQVFVFCIHGNELIEESSSVMEAAYSCHWYDGSEEAKTFVQIVCQQCQKSLTVSGAKFFTVSLDLFASVFGAVVTYFMVLVQLK

IRs

>GrubIR25a

MCWILLLLLLEHALSQTLQTINIMFINEAKNQLAEKSFDVVLNYLKKNPKLGVKVEAVVRVTISGTDAKAILESICEAYNGTVADGKPPHIVLDSTMDSIPSEAVKTFTDALALPTMSSSFGQEGDLRQWKTLDVEKQKYLIQINPPADIIPEIVKSIVQLQNITNAGILYDDSFVMEYKYKSLLINMATRHIIVNVNNAESIVKQLTRFRDLDIVNFFILGGLPTIKIALDTASAKQYFGKKFAWHVITQDKGQLSCSCSNATILYVKPEPEPGMKERLDALRNSFNLVEEPEITSAFYFDFFLHSILAVKSLLDEDEWPKDFNYTLCDDYRADREIVRKDIDLMKHLKTVSEPYSYAPFLLERNGNSFPEFVMKLEKVTIVNSQSESAESLGTWKAGLNSPIVLKDAAAMNNFSAVTVYRVVTVKQKPFVIEYEEEGKKKYKGYCIDLIDEIKKLVGFDYEIYVAPDNQFGNMDDNGNWNGMIKELVDKRAEIALGSLAVMAERENVIDFTVPYYDLVGITILMKKQTTATSLFKFLTVLENDVWLCILAAYFFTSFLMWVFDRWSPYSYQNNREKYKDDEEKREFNLKECLWFCMTSLTPQGGGEAPKNLSGRLVAATWWLFGFIIIASYTANLAAFLTVSRLDQPIESLDDLAKQYKIQYAPLNGSVAMTYFQRMANIEKRFYEIWKDMSLNDSLSEVERAKLAVWDYPVSDKYTKMWQAMKEAKLPNTLEEALERVRNSKSSSEGFAFLGDATDIRYQQLVNCEFQMVGDEFSRKPYAIAVQQGSPLKDQFNNAILQLLNKRKLEKLKEKWWSENPERQKCEKQDDQSDGISIHNIGGVFIVIFVGIGLACVTLGFEYWWYKYKRPADGGVGPMVVKPTAGGGRNVEKLSVTGLADFGHHAAFRSRNTHNSNMRRGNLGQIPTSQW*

>GrubIR93a

MVKFIFLQYLFISIFAYEYYYTPSDRKTTDNLLVLIDPNFLHPYYKGLNNNIRQMVSSIANKYLKGESLLITFTNKLNYRIKDDVTAIFSIINCEDTWDLFLEQQETSILFITITEPNCPRLPKEFGITIPLYEPGLEFSQILIDLRTEDFLEWETVNLIFDNSIDSKLIDNILEAQTKSIPLSKKSASVSLYKINGTVNKWIRKKEILRVMELIPRSGTFFNYLVVVQFELVPTIIEIAKSIGLMDPRNRWLFIRLKTDDSKANNVKNYIHLLEEGENVAFLYNVTNSKYPCKYSLYCHANELLDHLIVSFDKSMREEKLLSEQVSEEEWDLIRPSKSQRRNSIINFIKARQKEMSWCDNCTWWKIISSETWGTDFLENKELLESGDWTPRSGPVLVDQIFPNVAHGFRRKTIPLFTFHNPPWQIVEYDENGKPFQAKGVIFEVVDHLAKSLNFTYEIILMANTSLTTNQTNSNRFNESAGDLILGQSKEFLAWEQVVHLIQNKKVLLGAAAFTVTEKRKKSLNFTLTIRTENYAFLVARPKELSRALLFIQPFTPDTWQCIVAAVLIMTPLLNFVHRVSPFYEHYSQREKGGYMKMMNCFWYLYGALLQQGGGIMPEANSGRLVIGTWWLVVLVLVTTYSGNLVAFLTFPKMDKIISNVDQLMERRGEVTWGMPEDSTLHIILKSTDNDKLNDLSESAQLHPKVTQEIINQIRKGEHVYIDRKSILLYIMKQELQTTNQCSLSIGDEEFLAEQLAMVVSPSSPYLELINKQIYKMHQVGLIDKWMIDYLPTKDRCWSNTLSSESQTHTVNLDDMQGSFFLLFLGVALGFILIIGELLFKKWKKTQEKQVIHPFVA*

>GrubIR40a

KNIRIEMTRCNKLFAYLVVSILINGCLSALEMAEKKRDFRSEMLITALHDMMVAIPTKHVAFIYDDLTDEYLLHLIAKVFSDNGIMLAHFILESFPQETSSIDLIVEDIKKTTTFSGNAVYITIASPSSAEGIIQMIDAENLAKRNIIYFFYAGKQRLSNNFFSYLNEAMRICLVLNPRYGIFQVLYSQARVKEKSLVLINWWSRKEGVFRFPLLPSAYDMYRNFQRRVFKIPVLHKPPWNFVTYLNDSFEVKGGRDDKVLRLLASKLNFRYEYFDPPDRSQGSAVVNGTMQGVLGLIWQRVADIFIGDLTVTYERSLAVDFSFLTLTDNEAFLTHAPGRLNEALALVRPFNWKVWPAVIATFLLAGGLVYLFFLAANDWKPHKRKLLYQCVWITISIFLKQNISANLKYDRVRFLVILLYLMSTYVIGDMYSANLTSMLAKPAKEKPISNLEQLDTAMSTRGYQLLVEKHSASHTVLENGTGLYKSIWEKMKTQQTYLLNSTEEGMLMVRDKKNIAVIGGRETFFYNTKRFGIHHFHLSEKLITRYSAIALQMGCPFIDNFNEILMQLFEAGILTKITEDEYQKLGELGATVSEKENTEDNNIERNHQKEDDKKLRTMSMKMLQGAFYVLLSGHALAAFSFLLEMVFNYFNTRGNIKLLFSLKLSS*

>GrubIR41b

MRFHFIWLWFSSFCNKKCLSISIISINGTTINSYLEVLANEVANKYFSDHRCVVVMSDPGLLRNFKINTTVVRIEMNPAGENCVEEVANLLRITYRDNCDGYIIQSRNPSCAFDGWLDIYFHASVARHNPKYFFLPVYPDLLNYSDELLSRRESDQTSNFLVSELYENSTDWSVKISTNNFYLHPTVVGRDPKIYLDEWHYEIGFKENVDLYPDKVLDLRRKPFRIATAKYFPLCSSYPLDGSEVRISLYFCEKYNCTPQPVTDNALWGTIYENLSADGVLGNIYNDRADVGVLAVYLWLKEWYYIDYSTSYMFADVTVMLPKPKKLSAWFLPFLPFKSDVWIAFVISLFLSASSLYLITRASIKFTRFRDQLIKKVQFTTFEDSSMRAIGLAILQQPSSRLIGDSPNRYLFTSFEFLYLVLSAMYSAELASFLTVPLYYPPIDTFHDFAQSGIHWFATNLAWIFTLQGVDEEDAKLIVSHFEALSMDELKKKVKEGKYGFSVERMPGGSIGEQDYQTSEVIPMYHIMKEKLYGSPLVASIKKGSPYLKHFNEVVLKVVEHGHYLYWENEVARQYLGSSIQAAYEEAKTVHVDNSPAVIQVSNIQGVILIYAFGIIISIIVFFIEIMRNRKIKTSSFREKKKLVIYKNSIVNKSPSLQHFNLPCFYL*

>GrubIR41c.1

MLLCGKSRPYASQINFKIRKMKLVLLLILLICRLSTGIEFTEDVASKSLSKLVSKIFLDMPECVQVVLSDFTFLKLPSNFVYLRVQYNSSNLFSSLRASYKMKCMGYLIDENSANDFLTTLGEARSSALERHTPRIVVVPNRRGNYSANVFMHPESKYIPDMIVIQIIADSKCETGVEMELMTNSFHNKSIFPEGERLGVWPVDEIVFFPDKLSNLNGKELIIAALHYPPYVLLPEFDGLDVRIVYEFCQLYNCKLSPLADDHKWGEIFTNGTGTGVSGNVYMDYADIGIGALTIWANEYKYIDFSHPYLESKAAVLVPKPAQKAEWRIPFLPFSLTLWVLQVLSIILAAVILILVNNVSSRIAEGLMLGGEFATRRGIFLRAYAMALLQPPPSRLPASSPLRRFFTIFEVLFLFFTTVYSGALSSVLTVPLYYPPIDSPLQLYEAGIPWAADHMAWIFSIQDATEQPLKGLVERFEVHSHETLISLLKKSDYGFIIEIMPGGHLTQTEYLSGEAILNHHVMKHDLYTASLNMCFRKGSPYTEKLNKLIGKCLDAGLSLLWETDVTLTYLSTRIQTALKISEGTKVEAHHPTKLKLTHIQGAFILLVFGLFVSFGVFMLEKLGDGPSSLMKIHALWKKP*

>GrubIR41c.2

GIWPDDDIVFFPDKMGDLKGKELYCSTVHYPPYVLAEDQVDGVEVRPQLEFCRIHNCSLKIRTSKYMWGDIWPNGSGNGIFGEVFLDQSDFGVGGLYTWLEEFQYLDYSTPYGIGRINVLVPKPTRVDEWKTVFMPFSPAMWLLLVFSIFAAATIMHCANIVAGIISDELILGGEFATWVGILFRAIGMSLLQPPPTVLSASPLRRFFTIFEVLFLLFTTIYAGAIASVLTIPKFNPPIDSALDLYESGLHWVALHEVWILSLVESTEPYIKSLIDRFETQDAETMARYAKEGVYAIGIELMETGQMIEASYLSVETLANHHTMKNDLSWSHVIMLHRKGSPYLEEMNRVIGRSRAAGLFLLWATNITANHLSSRFQIATQISKASTKIEMDQPIKLKLLHIRGAFMFLAAGMSLSSLVLFMEIIKFKLSNIKSIRPN*

>GrubIR75c

MVYSSFYFDIIKNYFNFLHIQSVVMIICPEEALKVNVLKEMSSQSFLLSFSVNSIQDNPIIRTGNVLDISCPNTSVILQKMSARKMFNSDMEWLMLEEVNTTDSFEITAKNDALKNTLALPGSCVTLAQISENEKRVKLYEVYRTAMWEPMKYKFLEEYFLNGSTKFTFRRGYRKFEGVILRSASAIFYPELFLGWESQENKEVDTISKAAFAFMNDIADHLNYSFTLKFLNFYGYETNGSFNGVVGYLQREEIDMSANGLMMNVDRMPYVDFVGDILVLRSPLIFRQPALSSVSNLFVLPFHQTVWILIVVVTMLYSMVLFLNLFLKMRLFRLKETDDTSVLEILTVIMAYVCGQGTGLELKPGAGRITLCILSMFCFFLSVSFSAKIVALLHSSAHTIKSLPDLTRSPMAVGIQNVVYNKHFFSISKDKEVRELFQKKILPQGDKAIIKPDVGIQKVKEGLYAYKVETPWAYTLITRTFEDKEKCDLDELNPFVMPTLAVGLQEKSGYREPIARSFSRLSEVGIKRRTMNLYYPQKPFCNLHNIGYSSVKLTDFKPALDFVSYGLALSLILFLFELLSKTRYLVRRILKS*

>GrubIR75d

MILFKHPRLPVMKNIYVLPFHRNVWFCCVALFFLCFGLLLAATISYPGVFEAHTYSTPLDLLTLLIGVASQQGIDLGARSLSTRMVLLLFSVCSLFLSTSYSANIVALIQSGAETITTISDLIQSPLNFAVEDVPFVKIYMKESSKEVKDLYEKKVSKGNSNPFISAERGIEKMKNEVFAFHVETSLAYKIMSKTYTSQEKCGLSELHVYTYPRYSIPVRKDSGYRDLFASRLSRQRECGLIKRAEQLFLERKPACVAKDSGFVSVTFEDFLPGLLVLCWGMAAAFVALLLEITTSKCALLVRWEKKKEK*

>GrubIR75d.1

FLYSLLDTRSFTMREILCFSLLFVLILPARSSVDLPVKIIQDYFDVLSIKSLTMLLCSAPEKKISILRQLSAEGFLVSFSLDTVKDLPIVRSGGVLDLTCNFSLDVIHQFTRQKTMGVHTEWLLLDDGSSLDVIKDAYILPGSYVTIAQVLGEEVVYLDVYRTSPYRSLKYSVLDNQTLEQFFQLPDRQTRNNFEGISLLGAAVLYFPHMFCGFDCRDHPEVDTIAKTGFPISQHLQEQLNFTLTFQILNDYGWKINNTFSGLMGLLQREEIDMGVIGIFMRPDRIEVVDFTGDTFEIKSLLIYKQPALSAVSNIFVLPFTRMVWLCCAALSVLTGLFLLADVATSFGNRQTFEESVTTSDVVTLIIGCICQQGTALVPKTLSARIIIFLFSLCCLFFYTSYSANIVALLQSSSATFRSLSDLTSSPLGIGVQDVIYNKMFFGEATDKDVRDLYNKKIAPQGPKAYLSPVEGIKKLRSGMFAFDVEIHWGYKIISDTFRENEKCDLDEMRIFLLPKLSIPVVKKSGYREHFTRMNTWQRDVGLHSRIRQRWLPKKPVCDNSGRGYVSVGLTDFRPALLVMVYGYIFSIAAFLIEIIFRYRFGFLNKTKRRRKKS*

>GrubIR75e

MLKLYLLLLMLLGFFWENIDSYKFPISSFFKNKYVGSVTLVACSEKDLIQEPLEVNSKHETGILMGLKGNISSLHPTFDSRHYRTGVYFDFSCDISKQVFRKMSREQLFNMSFTILILSDDYNETIEYLEHSTISFDSDVTLAVGYCLYDLYRTHVSKPLTSNMVGTYDTMTITYVKRDKRNDLKGITINTGIVWTRGFPLNITPEGIEKFLDFNHEPEEEFLSRYGFAIHKILEDGYNFRMNISIYDDWGYFNEKLKIFDRGMFHGLSLGDIDLGTAISRIYGQRLDVSFYFPPFLRFRTCFIFKHPSRLGKFTALVKPLSLGSWVCVLTIVALSGFILWFIKSFDTADIPPNEHDLGASLLSSLGTLCQQGLSNDSLRLPVRVLYIFLLIASLVIYMFYGAAVVGFLLLPSPKTIDTVEKLIASPITSYAENLAYHRTYFHGNFSEKAAKAYVAIKEKKTEKERWIELMNGIQKVKQGGAALYTQDTNLYRAIENSFSNSDICSLAEIEIVSLWVSTVIRKKSPYRELLCQGMILIHENGLLNRAMKNWQAQRPTCFAQNESPTVSLEATMIAHIIYILGLSLSLVLIAFEIITNKMKKFSSKTRKTK*

>GrubIR75g

MEQHRIILFILCCGGAWSTSYCIVPEGIHEVIEYYFRELFGAHLYFCKLEDAVKAFKRFTPGGPKYNVRHNHDFREPQELLMSYWQPSRLGIFLDTSCDQGLFFFNYTKDLFNASYSWIVWSEEENFTIFEDTRLSADSDVKVVKPGLEIYDIHRIHISIPLRTKMVASWNVTDGLVMISTSMDRRDFGGFTFTASLMMIDIKFNESNLLEPLLDKTYEPGKDMVRRYGLAVFLHVAELYNFTYNYILTNAWGDPQPDGSWTGMVGQVARGEAEFGLAPAKYVVKRFDVIDFINSMHIVKCCFTFLQPKLFGSVKALVLPLDEIVWVCLAVLGILSVIVFRILAKYDNTGLSNDSWGGSALLVVGAITQQGIPDNTEKVSTRIVYIFLLIVSFFIAVYYNTAILNGLLLPAPNAIQDIEQLLKSDIKLGMLDIPYLRNELIQNDTMTIRVREKIAKAKPKQIFYSVPDGVRMIKKGKFALFTEDEAIYTEILHQMTDAEVCSVSEVLKYNPFHVGAVAKKNSPYKELFNRAFAVMRERGILKRQLEHWLVKKPECNWKQDALSLSMEPLALAYALFSFGAIFSFLILGFEIIYCKKTNDQQAA*

>GrubIR75h

MVGSEDGRVWDVYGKPLKRTIFNEAGTWRPANIFMPKIKFRYDLGGVILKGVLVELDLQFYANNITHKIADHNYFPDMEMTQRVSYIFMTYLAKFYNFGFSIVPTDTWGANLANGSCTGMIGILQRREADIGLSACSFRVERVEVVSYAQRSQELRFICIFMEERIHGTYSSLLIPFSFQAWLCVFFAIVVGATIFSFLQKRDLTEGIVFVTAILSQQGLQKDFRSFSARVYSISLLMLGLVLYSYYSAAIMNGLLSPAPGSIRSIEDVIKSPMKASLARVPYMIPKVNQKAYVTPGLLEKTKKQEKSEQMLDAFVGVDRIRHERLTLIADDMSLYAIINEKYTDAEKCNLMEIEVIRSFPFGNTLQKNSPLREMMCQGTLRLKENGIARREMQMWYHPKPQCLGSTTYTHVTLEAVGLAFTLFLTGVALSCFILLSEIMYKKLFKANFSSDKFIKPQVRPFLR*

>GrubIR75i

MHSLVFLLLFIGSTYSLRHLNSTLEIITTYFNFRATNVINIYSCNHKEGVWLLKHLNALGFMIQIKHHTIDEKEESRPPYVRGWFVDLNCTKNIDFFRTINGLDGIWLGYPVKSLNLNLTKIRLDSDIVLGTEDGFLWDLYNNTKQQLKVLSAGTWRPPKISMPNSKYRYDLNGTVLKGVLLELELQFFKNNMTEKIGDYSYYPEWETSERLAYVYIQNLANFYNFEIEIVPSETWGYVLPNGSFGGMVGVIERGKADLGLTACAMREDRMRAVNFVPQLQDLRFICLFMEERIRGTYSALILPFSFNVWLCIFVAIVIGAAIFSLVQNKDVTEGIIFVTAILSQQGLLHDKNRMSARVYSISLLFLGLVLYSYYSAAIMNGLLSPVPPSIHNELELQKSTMKASIAKVPYLIATFSQKAYLTAGMFAKTREHEETMLDPFVGLDRVRKERLILISDDLALYGILKTAYTDLEKCNVREIETMRPFPLANPIRKDSPFREMIAQGMLRLQENGMGKRDRRVWYPPRPHCFASTIYTHVTLEAVGLAFTLYVLGIVLSFTILLSEVVMKRFKKFKKTDPDSEFQGYY*

>GrubIR76b

MQGLYYLLLALCSNYPPPPSETEFSCKLRKGKSEKGILKGKTLKIVTYDDRPFSGATANASGDLEGYGLVFEVLETLQEKFGFEYELQKESRVMGDESSGLLGKLVAKEVDMIAAFLPILPGTHNYVTWGTQLWQAHYYVLMKRPDDSATGSGLLAPFDNKVWILILISLTSVGPIIYLIMWLRIKLCPNDTKQLFPLSSCIWFVYGALMKQGSTLSPLSDSARLLFATWWIFILILTAFYTANLTAFLTLSLFTLPIKEVEDVAKPPHKWFTTQGSSVEYAIKNKDDGDLNILLSSVRRGNGRFIDTSSESHVLELLYDGWLYLDTSDTLNRLMYDDYKRKTLEGEDENKRCTFALTQHPFLVRSLAFAYPKGSSLPELFNPVIQVFVESGILKHLLNEDLPDTTICPLNLGNKERKLRNTDLFTTYVVVLAGFSGALIVFCIELLWTYCATRSFNSKSNKSHRLKNNYNKFVIANEFGKTATLKNQVQTKINGREYFMITAKEGDKRLIPLRTPSALLFQYGLNYPTMF*

>GrubIR1

MSSWAEIFLILAVVGISSGRRGGVYDMTKKVYTDRRILSHSEADVNMLFKFVMSDTRCLQVETDGTPFGMSFQEDLHQSDAVPTLVTGGRWSMVEGCKGFVIIASVFSAAEALLQKMPRWADHRILVILKGVGSNPLLQPIMADISIFMDAEVALVSSTDLTTGYLMTQNNRYYVRFRSVSNTPWKRLKEGPRDFFGREISVKTSNCSMFSQVGPINELGEPAWTGGAEMIMFRDIAQRLNLKPTFSATKSIHAGWFKQPLTDKNPSDIAFCGILVSSRTLDIKEITVSQPLALLCLKLLVPRPQRVHDQWDEIFEPFSPDLWLLIAVVTLCTTFLLQRFTTATRRLVYTKKIKKYELLGESFLQIVAILVLSNVPKSAKEQGAIRHILTWWSIFTLVMTTSFSSGLISHLTNKEYTKKIETFDELIKNGFSWAMKTRPEFSDLINLKDSSQVRWKYRFIKVNSTREHIRLIRKDKQVLWGYQVYGGFSLPDSDLPNYILRKFEVSSHCFMEYFIGFAIRQGSPFESPINRYIRWYNEGGLISAIVKSVHAERYLKYPYVFNVLRQTKPRPGEAERLQLSHLTGIFIVWSMGTAVSIFVFLLEIAQKCRI*

>GrubIR2

DEADVGFSGLWIANNKINTGVEMSVSLKRICMMYLVPRPVPINSEWYGLFSPFAPYLWLAIFLAYLSVCLLLPFLAYLDSIISVYGECHYLTFKGSWILLAGMLLQGNWMRSTHSQGPCRHLIAWWTVFSLLIGTVFSSSLASHLTRAVFTWKPETIEDLVKTDYSWTTTRVPVVETLFNLNDPVHQEWVRRIRVVRTQDELHAALRKMDNVVMGIYTFGIFSLMDEDIADQLLEGYEISSYCFTEFYLGFAFRRGSPYLKFFNEQLKNMIETGFIQYFMKTSMERMMKEKRYMYEVTRLSKNNGRGTQLTLDSLKGIIQFWAGGLLVSTAVFLAELVYNPLTDYLSASKFCQSRNSLSVLQGKITER*

>GrubIR3

MWLGVMLTLAVSSLVFYLLANFHKYYENGRTFLEPEFIFGRKLKFWRKKKSLPKLLPMNVKRGVPLGLYLFDGIGNSLINAYSMLLLVSLPKMPPGWSLRMLTGWWWLYCILVTVAYRASMTAILANPAQRVTINTLEELASSPILCGGWGEQNKEFFTTSLDSAGQRVGHKFEVVYDSNASISRVAEGNFAYYENIYFLHYAKITQKVMTGFESFTREKSSHNLDGKKDLHIMSDCVINMPISLGLQKNSPLKPRVDRFLQRVIEAGLVKKWLADVMLTTVVAEAPLEKTNINAVMDLKKFVGALVALGIGYGLGIIALIIENIYWYYIVQKNPLF

>GrubIR4

EKTKMVVAVSSPLEESEIYELLFFMFLRGDLDVIFVGIVNSTITVFTFFPFGKDRKSCPRNNNSTEVLDSWVNGTFIGNENLFPEKLPHKFNSCLMRVGTYHNPPYIIVDQEGRPSGGIEHGVVEIIAEHLGMKIEYCIYNRSDARLWMEEDGPKGLSTDLRLGHVWLTVSGDKNLVYTFPSLIIPHPFMTEHICFFFKNPKDVATWKLIFVGFNEVLWIILIATAFAFPCCLFLLARFQNYQHPFQKFSISLMSSYALLLSFPSTVDPRTIIFRLAFATWLFYTIHINLAYSAALKSLLTAGKTEPKMDSFEQLLEE

>GrubIR5

MDRMEATITNVEDLANQNKIKYGVLKGGSSANFFRDSNVSLYQKIWSQMESARPSVFTKSNDEGVERVLRGKRAYAFFMESTTIEYQKEKHCSLMQVGGLLDSKGYGIAMPFNSPYRIAISGSVLKMQESGRLQQLKDKWWKHSEDKEDCPKTEENAVSSSELGIANVGGVFLVLLIGCVAAFLVAILEFLWNVRKVAVEEKISPRDAFFLELKFAIQCYGTTKPVRKKPEESVAEEDLAECPEIEDEEEEEEEDRGFFGDENMEQEYLRMNGFNNNIRAKSTQSYS*

>GrubIR6

SGSVLKMQESGRLQQLKDKWWKNQKNKCPEEDKRKDSSELSIAHVGGVFLVLLIGCVVAFFVSILEFLWNVRKVAVEEKISPGEAFLLELKFAIQCYGTTKPVWKKREDSVVGKDFAEIREMEEVEEEEEESVSEAQPSAAEETIASEASELPQDEPPPPQEPEPEPEPLPPPPPKTEEDLWLFLREAVTQANPMSIDELLHKLLDTVNAVPQDFLEPVVEASIKRSQSMQSMDLSHAESLPADGEPPKEGEPGEQPPAAEGVAGVPTEGETK

>GrubNMDA

MLGVHFDTSSDRLLNEISTAIKVYAHGVEDFVSDYNNREYSLNTQLSCEGVGDSRWSTGDKFYKYLKNVSVEGEPGKPNIEFTADGALKAAELKIMNLRPGVSRQLVWEEIGVWKSWEKEGLDIKDIVWPGNSHTPPQGVPEKFHLKITFLEEPPYISLTPPDPVTGKCNMNRGVICRVAREVDIAEVDIPMAHRNGSYYQCCSGFCVDLLEKFAEELGFTYELVRVEDGKWGTLENGKWNGLIAELVNRKTDMVMTSLMINSEREAVVDFSVPFMETGIAIVVAKRTGIISPTAFLEPFDTASWMLVGVVAIHAATFTIFIFEWLSPSGFNMKCFQQQSTVAGAGHRFSLFRTYWLVWAVLFQAAVHVDSPRGFTSKFMTNMWAMFAVVFLAIYTANLAAFMITREEYHEFSGIDDHRLAHPYSHKPTFKFGTIPWSHSDSTLKKYFKEMHAYMRPFNRTTVTSGVEAVVNGDLDAFIYDGTVLDYLVAQDEDCRLLTVGSWYAKTGYGLAFTRNSKYLPMFNKRLLDFRENGDLERLRRYWMTGTCKPGKQEVKSSDPLALEQFLSAFLLLMAGILLAAALLLLEYLYFRYVRKHLAKTDRGGCCALVSLSMGKSLTFRGAVYEAQDILRHHRCNDPICDTHLWKVRHELDLARIRIRQLEKEMESHGIKPSRSPAYERFLNNRDVLRARDMTRNHITSTEIEGRFCASTPQLYSGRTEIAEMETVL*

>GrubGluR1

LAIEIWLYVLAAYVLVSITMFIVARFSPYEWQNPHPCDMENDLVENQFSIANSFWFTIGTLMQQGSDLNPKATSTRIVGGIWWFFTLIIISSYTANLAAFLTVERMITPIENAEDLAGQTEIAYGTLDSGSTMTFFRDSMIETYKKMWRFMENKKVFVSTYEEGIKRVLEGNYAFLMESTMLDYIVQRDCNLTQIGGLLDTKGYGIATPMGSPWRDKISLAILELQEKGEIQMLYDKWWKNPGDTCVRKDKTKESKANALGVANIGGVFVVLLCGLAIAVCIAICEFCYNSKRNAPIERRAGSQSLCAEMADELCFALRCRGSRQRPALKRQCSKCASSASYIPTIPPAPAPPQLPAYSNAQGGSLGIPLDLPHHAMNMKI*

>GrubGluR2

STRMVAGIWWFFTLIMISSYTANLAAFLTVERMDSPIESAEDLAKQTKIKYGALQGGSTVAFFRDSNFSTYQRMWSFMESARPSVFTDSNSDGVERVIKGKGNYAFLMESTSIEYVIERNCDLTQIGGLLDSKGYGIAMPPNSPYRTAISSAVLKLQEEGKLHILKTKWWKEKRGGGACRDDALKSSSAANELGLANVGGVFVVLMGGMGVACVIAVCEFVWKSRKVAVEEREASFCTGMASEIRNAIQCKNNDPTKEALESKTPEKKDVPVFLPQGTYSQYGFLSNGPL*

>AchiIR1

HLENQFTLLNCMWFAIGSLMQQGCDFLPKAVSTRMVAGMWWFFTLIMISSYTANLAAFLTVERMDSPIESAEDLAKQTKIKYGALRGGSTVAFFRDSNFSTYQRMWSFMESARPSVFTDSNSDGVERVTKGKGNYAFLMESTSIEYVIERTCDLTQIGGLLDSKGYGIAMPPNSPYRTAISSAVLKLQEEGKLHILKTKWWKEKRGGGACRDDALKSSSAANELGLANVGGVFVVLMGGMGVACVIAVCEFVWKSRKVAVEEREASFCTGMASEIRNAIHCKNNDPTKEALESKTPEKKDVPVFLPQGTYSQYGFLSNGPL*

>AchiIR2

MLDNPDVMMPNNDAGVDRVVSEEKYAFFMESASIEYEVQRKCQLAMVGDLLDSKGYGIVMRQNSTFRNALNKNVVRLQENGKLTQLKDKWWKEKRGGGACTSGTEEGEASELNLDNVGGVFVVLVAGCLIGVLLSFCEVLWDISQRDEKISFKHELIEEIKFIMKCKGTVKPVRKNSLTIANCNNLSKSSSKSSTTSKRTSKNSFSFRSPYS*

>AchiIR3

MLIKILLISFLLITKSYSSENEGNEEGIDNEEIVKTEVRIGALFDKDDETLIKAFNSAVELVNSDEELLPNMTLVPITFTGIPEYDSMEVGKKVCELMSYGVAAIFGPQSPFTSYHVQSLCDTMEMPHLSTKWDLSQRRSSCLLNIYPHPSTLTQAVTDIVTAWNWKGFTVLYDDFDALKKIQGIIKLADDKGYLVTVRQLEAEDGNYRAVLKEIKHSDETNIVIECAVEKLYDLLVQAQQVGIMGSHYSYIITTLDFQTINYEPFMWGGTNITGIRLVDPDDPYVINATQTEQRGEDGMESTTTNLDYIPTTEPPIEEEEEEGSSIPTVEAALLHDAVRLFVKALHHLSPLNIKPLTCGPHSSLDFGYTVINYMRLSEIKGLSGVIKFNHEGFRTDVQLDVISLTEEGLKKTGTWNTTTGLVMDPPDTNEGLVVDAGEDLRNKSFIVIIALTKPYNMLKEDSKTLTGNDRYEGFGVDLIHELSLMSGFNYTFVEQYDKNSGSPTTLQNGSRIWNGMIGEVQAGRADLAIADITITRERERDVDFTHPFMNLGISILHRKPSKAPPNLFSFLSPFSNDVWSCMLGAYFGVSLLLFVMARLSPYEWTNPYPCIEEPEHLENQFSLLNSLWFTLGSVMQQGSDVAPISVSTRMVASIWWFFTLIMVSSYTANLAAFLTVENNVSPFSDVKELAGQTEIEYGAKNNGATANFFRDSKEELYQKIYKFMLDNPDVMMPNNDAGVDRVVSEEKYAFFMESASIEYEVQRKCQLAMVGDLLDSKGYGIVMRQNSTFRNALNKNVVRLQENGKLTQLKDKWWKEKRGGGACTSGTEEGEASELNLDNVGGVFVVLVAGCLIGVLLSFCEVLWDISQRDEKISFKHELIEEIKFIMKCKGTVKPVRKNSLTIANCNNLSKSSSKSSTTSKRTSKNSFSFRSPYS*

>AchiIR4

EINSHNLEGKKDLHIMSDCVINMPISLGLQKNSPLKPSVDKFLQRVIEAGLIKKWLADVMLSTTVAEAPFEKNNINAVMDLKKFVGALVALGIGYGLGLIALIIENIYWYYVVQKNPLFDKYKQECNRKC*

>AchiIR5

ELTKNGFRKIGTWDPVKGISYTRTGSQMENEMFQSISNKTFYVVSRVGEPYLKEVDKNAEGNARYAGYSMDLIDEIAKDLKFSYKFYLAPDGEYGSFNKETKQWTGLIKELRERRADLGICDLTINYERRSAVDFTMPFMTLGISILYSKPMKQPPELFSFLSPFSVDVWVYMATAYLGVSLLLYFLARCTPDEWDNPHPCNPDPTELECIFSLHNCLWFSIGSLMAQGCDLLPKALSTRVVAGMWWFFVLIMISSYTANLAAFLTMDRMEATIESVEDLANQNKIKYGVLKKGSSANFFKDSNVSLYQKIWSQMESAHPSVFTNGNDEGVERVLKGNRGYAFFMESTTIEYQKEKHCTLMQVGGLLDSKGYGIAMPFNSPYRIAISGAVLKMQESGRLQQLKDKWWKNQKNKCPGEDKKKESSRLSIAHVGGVFLVLLVGCVVAFFVSILEFLWNVRKVAVTEKISPGEAFLLELKFAIQCYGTTKPVWNRREDSVVEKDFVENHEVEGEEEEEEDRGFFGDDNMEEEYMRMNGFNKNIRAMSTQSYS

>AchiIR6

VDKNAEGNARYAGYSMDLIDEIAKDLKFSYKFYLAPDGEYGSFNKETKQWTGLIKELRERRADLGICDLTINYERRSAVDFTMPFMTLGISILYSKPMKQPPELFSFLSPFSVDVWVYMATAYLGVSLLLYFLARISPMEWKNPHPCNKDPEELENTLAIYNAIWHNIGSLMQQGSDIAPQALSTRVVAGMWWFFVLIMISSYTANLAAFLTMDRMDATIESVEDLANQNKIKYGVLKGGSSANFFRDSNVSLYQKIWSQMESARPSVFTKSNDEGVERVLKGKRAYAFFMESTTIEYQKEKHCSLMQVGGLLDSKGYGIAMPFNSPYRIAISGSVLKMQESGRLLQLKDKWWKNSADKQNCPVEEAGTSSSELSIANVGGVFLVLLVGCVAAFFVAILEFLWNVRKVAVEEKISPSDAFFLELRFAIQCYGTTKPVRKPREESVAEEDIAECPEIEGAEEQEEEEEDRGFFGDENMEEQYMRMNGFSNKIRAKSTQSYS

>AchiIR7

IFSLHNCLWFSIGSLMAQGCDLLPKALSTRVVAGMWWFFVLIMISSYTANLAAFLTMDRMDATIESVEDLANQNKIKYGVLKGGSSANFFRDSNVSLYQKIWSQMESARPSVFTKSNDEGVERVLKGKRAYAFFMESTTIEYQKEKHCSLMQVGGLLDSKGYGIAMPFNSPYRIVMSCSVLKMQESGRLLQLKDKWWKNSADKQNCPVEEAGTSSSELSIANVGGVFLVLLVGCVAAFFVAILEFLWNVRKVAVEEKISPSDAFFLELRFAIQCYGTTKPVRKPREESVAEEDIAECPEIEGAEEQEEEEEDRGFFGDENMEEQYMRMNGFSNKIRAKSTQSYS

>AchiIR8

LTITYERRSAVDFTMPFMNLGVSILYSKPTKQPPDLFSFLLPFSIDVWTYMATAYLGVSLLLYFLARCTPDEWDNPHPCNPDPTELECIFSLHNCLWFSIGSLMAQGCDLLPKALSTRVVAGMWWFFVLIMISSYTANLAAFLTMDRMDATIESVEDLANQNKIKYGVLKGGSSANFFRDSNVSLYQKIWSQMESARPSVFTKSNDEGVERVLKGKRAYAFFMESTTIEYQKEKHCSLMQVGGLLDSKGYGIAMPFNSPYRIAISGSVLKMQESGRLLQLKDKWWKNSADKQNCPVEEAGTSSSELSIANVGGVFLVLLVGCVAAFFVAILEFLWNVRKVAVEEKISPSDAFFLELRFAIQCYGTTKPVRKPREESVAEEDIAECPEIEGAEEQEEEEEDRGFFGDENMEEQYMRMNGFSNKIRAKSTQSYS

>AchiIR9

ARISPMEWKNPHPCNKDPEELENTLAIYNAIWHNIGSLMQQGSDIAPQALSTRVVAGMWWFFVLIMISSYTANLAAFLTMDRMEATIESVEDLANQNKIKYGVLKGGSSANFFRDSNVSLYQKIWSQMESARPSVFTKSNDEGVERVLKGKRAYAFFMESTTIEYQKEKHCSLMQVGGLLDSKGYGIAMPFNSPYRIAISGSVLKMQESGRLLQLKDKWWKNSADKQNCPVEEAGTSSSELSIANVGGVFLVLLVGCVAAFFVAILEFLWNVRKVAVEEKISPSDAFFLELRFAIQCYGTTKPVRKPREESVAEEDIAECPEIEGAEEQEEEEEDRGFFGDENMEEQYMRMNGFSNKIRAKSTQSYS

>AchiIR10

VDKNAEGNARYAGYSMDLIDEIAKDLKFSYKFYLAPDGEYGSFNKETKQWTGLIKELRERRADLGICDLTINYERRSAVDFTMPFMTLGISILYSKPMKQPPELFSFLSPFSVDVWVYMATAYLGVSLLLYFLARISPMEWKNPHPCNKDPEELENTLAIYNAIWHNIGSLMQQGSDIAPQALSTRVVAGMWWFFVLIMISSYTANLAAFLTMDRMEATIESVEDLANQNKIKYGVLKKGSSANFFKDSNVSLYQKIWSQMESAHPSVFTNGNDEGVERVLKGNRGYAFFMESTTIEYQKEKHCTLMQVGGLLDSKGYGIAMPFNSPYRIAISGAVLKMQESGRLQQLKDKWWKNQKNKCPGEDKKKESSRLSIAHVGGVFLVLLVGCVVAFFVSILEFLWNVRKVAVTEKISPGEAFLLELKFAIQCYGTTKPVWNRREDSVVEKDFVENHEVEGEEEEEEDRGFFGDDNMEEEYMRMNGFNKNIRAMSTQSYS

>AchiIR11

VDKNAEGNARYAGYSMDLIDEIAKDLKFSYKFYLAPDGEYGSFNKETKQWTGLIKELRERRADLGICDLTINYERRSAVDFTMPFMNLGVSILYSKPTKQPPDLFSFLLPFSIDVWTYMATAYLGVSLLLYFLARISPMEWKNPHPCNKDPEELENTLAIYNAIWHNIGSLMQQGSDIAPQALSTRVVAGMWWFFVLIMISSYTANLAAFLTMDRMDATIESVEDLANQNKIKYGVLKGGSSANFFRDSNVSLYQKIWSQMESARPSVFTKSNDEGVERVLKGKRAYAFFMESTTIEYQKEKHCSLMQVGGLLDSKGYGIAMPFNSPYRIAISGSVLKMQESGRLLQLKDKWWKNSADKQNCPVEEAGTSSSELSIANVGGVFLVLLVGCVAAFFVAILEFLWNVRKVAVEEKISPSDAFFLELRFAIQCYGTTKPVRKPREESVAEEDIAECPEIEGAEEQEEEEEDRGFFGDENMEEQYMRMNGFSNKIRAKSTQSYS

>AchiIR12

WFSIGSLMAQGCDLLPKALSTRVVAGMWWFFVLIMISSYTANLAAFLTMDRMDATIESVEDLANQNKIKYGVLKKGSSANFFKDSNVSLYQKIWSQMESAHPSVFTNGNDEGVERVLKGNRGYAFFMESTTIEYQKEKHCSLMQVGGLLDSKGYGIAMPFNSPYRIAISGSVLKMQESGRLLQLKDKWWKNSADKQNCPVEEAGTSSSELSIANVGGVFLVLLVGCVAAFFVAILEFLWNVRKVAVEEKISPSDAFFLELRFAIQCYGTTKPVRKPREESVAEEDIAECPEIEGAEEQEEEEEDRGFFGDENMEEQYMRMNGFSNKIRAKSTQSYS

>TpapIR8a

MWLIFKTLMVTIILLEDTVGSVKLLILSEVNGTVWTEVTTEENWEEDYVVLNRDDSEESFKRVCEQLESGVWLVLDLTWSGWGIVAEIPGVRYLRADIGITPFMRAVEHSLIKLRNSTDAALIFQHSRHFEQSLWYLVRESSLRVTVLQGLDDKSAETLLNMRPSPSSYVIFGDHGAVKYILKQAVEKNLVYLDDRWALAFMDLGPTSLNVTVLKKRIMTIYMSQKMCCFPGQKLPCHCTEPFDIEKEGAKQLKKVIGKAIALSLNSGLSPEPLNTSCSSSPMSFTNNTIFYDKLNEALNDSWLLHMVNNRVELRLQLEVKNVDQQKTQELGRWDTGIGIRTAAMVQRVKRFFRIGTGFSTPFAYPTDQINPDGSQKWEGYALDLIERLAKNMQFEYQLVPVPNEMFGTRLENGSWDGLVGMLATGRVDMVIGSLTMTSEREEVIDFVAPYFEQTGFTIVLRKPMKKTSLFKFMTVLRLEVWLSILGALCLTALMIWFLDRYSPYSARNNKEKYPYPCRDFTLKESFWFAVTSFTPQGGGEAPKSLSARTLVAAYWLFVVLMLATFTANLAAFLTVERMQSPVQSLKHLARQSRINYTVVKNSDAHNYFKNMKFAEETLYKYWKEITLNSSTDQSKFRVWDYPIKEQYGHILNAIEKSGPVATIEEGLGKVIENEQAEFALIHDLLELRYYVYKNCNLTLIGEPFAEQPYAVAVQQGSHLNEEISRRILDLQRERFFESTASKYWNSSFKSKCDNVDEGEGITLESLGGVFIATLVGLFIALLTLGAEVWYHKKKSKNEIIIKNQQKLQSIIRDEIFTTKEFGHHNFINRKASLLTTKPKVKQITVYPRGQLY

>TpapIR25a

MCWILQVLLLYHATVTVLSDLQNINIMFINEASNQLAKKSFDVVLNYLKKNPDLGVKVDALVPVEIPTKSERDAKVILETICEAYNGTVSKGKPPHIVLDSTMYNVPSEAVKTFTDALALPTVSSSFGQEGDLRQWKLLDSDKLKYLIQINPPTDIIPEIVRSIVQIQNITNAGILYDDSFVMEYKYKSLLINMPTRHIIQVTNLRTIDSQLKRFSKLDIVNFFILGRLPMIKIALETANANKYFGRKYAWHVITQDKGQLSCSCNNATVLHVKPEPDLGQKERLDYLKNSFNLAEEPEITSAFYFDFFLHSILAVKNMLDADEWPKDFNYILCDDYREDREIVRKDVDLMKHLKSVSEPYSYAQIILEKNGKSFQEFIMKLEKVTIVNSQPESAESMGTWKAGLSSPIIVKDSTAMNNFSAVTVYRVVTVKQKPFVMEYQEDGETKFKGYCIDLIEEIRALVGFEYEIYVAPDNQFGNMDDKGNWNGMIKELIGKRAEIALGSISVMAERENVIDFTVPYYDLVGITILMKKQTTATSLFKFLTVLENDVWLCILAAYFFTSFLMWVFDRWSPYSYQNNREKYKDDEEKREFNLKECLWFCMTSLTPQGGGEAPKNLSGRLVAATWWLFGFIIIASYTANLAAFLTVSRLDQPIESLDDLAKQYKIQYAPTNGSAAMTYFERMANIEKKFYEIWKDMSLNDSLSEVERAKLAVWDYPVSDKYTKMWQAMKEAKLPNTLEEALERVKNSKSSSEGFAFLGDATDIRYQQLVNCEFQMVGDEFSRKPYAIAVQQGSPLKDQFNNAILQLLNKRKLEKLKEKWWSENPERQKCEKQDDQSDGISIHNIGGVFIVIFVGIGLACITLGFEYWWYKYKRPSDSGGVPVAVKPSIGGGRKVEKLSVTGLADFTHHPSFRARNTYNANLR

>TpapIR76b

MQGLVHYLLLSLCSNYPSPPGVSNFSCELQEGKQEKDVLKGKTLKIVTYNDRPFSATENNANGILEGSGIVFEVLETLQERFGFEYKLYKESSVIGDESSGLLGKLASGSIDMVAAFLPILPGTHDFITWGTQMWQAHYYVLMKRPEDSATGSGLLAPFDTKVWILILISLTSVGPIIYLIMWLRIKLCPNENNQQFPLSSCIWFVYGALMKQGSTLTPLSDSARLLFATWWIFILILTAFYTANLTAFLTLSLFTLPISEVEDVAKPQHKWFAAEGSSLEYAIKNKDDGDLNILLSSVYRGSGKFIDKSSENKILELLHEGWLYLDSSDTLNRLMYDDYKKKTIEGTEESKRCTFALTQNHFLVRSLAFAYPKGSNLPDLFNPVIQNFIESGILKHLLNEDLPDSPICPLNLGNKERKLRNTDLITTYYVVLSGFSCALVAFCIELLWIQCSKKCFNKSHLQLIVNDNNTKFKIDNEFEKAIAMQNQTQTKINGREYFMFTAKEGDKRLIPLRTPSALLFQYGLNYPTLF

>TpapIR92a

MNEPVELLTAYSQRRYIIQFTNPMCALDGWLDIYLHSSIARHNPKYFFLPVYPQTLTYGDELLARRESDLTTDFLISEIYENSTDWFVRLSTNNFYVHPTVSGREPKLYLNEWHYQTGFKENVDLYPDKVLDLRGKAFRVATGKYMPLTSSEPLEGSEIRIALYFCKKFNCTPEAVTDDILWGSIYDDLTANGVLGNVYNDKADIGAMAIYLWHKEWYYIDYTNSYLSSDVTVMIPKPTKLSAWILPFLPFKFDVWIAFFMSLVLSASSLYFITRVSISYTRFRDRLLKKVQFTTIEDSSMRAIGLAVLQQPSSRLIGDSPNRYLFTSFEFLYLVLSAMYSAELASFLTVPLYYPPIDTFHDFAHSGINWYGTDVAWVFSLKGVDEEDAQLIVDHFTMLPVDQLIEKAKEGKYGFTIERLAGGAITEQDHQTADVIPMYHIMKEKLFGSPLVASIKKGSPYLKHYNEVISKVLEYGHLLYWEGDVARKYLNSRIQSALHEAKSVHVDRAPAVVQISNIQGVILIYIFGTVVSIIIFVFEILRYRKQRDSLVKLISNHT

>TpapIR93a

MSNASLQMNKSHIYKFDQSVGDVVLDQITEFSAWDQVVQLIQNKKVMIGAAAFTITEKRKRDLNFTLTLKTENYAFLVSKPKELSRALLFIQPFTSGTWQCIVAAILIMTPLLNFVHRVSPFYEHYSDREKGGYTKMMNCFWYLYGALLQQGGGVMPEANSGRLVIGTWWLVVLVLVTTYSGNLVAFLTFPKMDKVISNVDQLMERRSELTWGMPEHSTLHIILKSTDNEKLNELSNSAQLHPKVTEDIVSRIRKGQHVYIDRKSFLLYLMKQEFRTTNSCALSIGDEEFLAEHLAMVVSPTSPYLHLINKQIYRMHQVGLIDKWLEDYLPNKDRCWSDPLSSESQTHTVNIDDMQGSFFLLFLGVALGFILIVAECLFKKWKNTQEKQVIHPFII

>TpapIR75d.1

MFCGFDCRENPEVDTIAKTGFPITRHLQEQLNFTLTFQMLDDYGWQTNMTFSGLVGMLQREEIDMGAIGIFMRPDRIVTVDFTGDTFEIKSLLIYKQPALSAVSNIFVLPFTQMVWLCCAALSVITGLFLMFDVAASFGFRQTFDEPVTTSDVVTLVIGCICQQGTILVPKTVSARISLFLFSLCSLFFYTSYSANIVALLQSSSANFRSLTDLTNSPLSIGIQDVVYNKIFLGEATDKEIRELYDKKIAPYGPKAYFQPEDGIKKLRSGLFAFDVNNNFL

>TpapIR75d.2

MSEVLVGHLSQQLNFKVKRRNASDCESLDRFLSNGQVDFSLTAVMMTKKRMEYSRFTGDLYELKSKILFKHPRLSVMKNIYLLPFHEDVWLCCLALFLLCFALLLVANLTVQGAFEKNMYSTPLDLLTLLIGVASQQGIYLGAESLSTRMTLLLFSVSSLFLCTSYSANIVALIQSGAETVTSVADLTASPLRVAVEDRHYNKIYLQEGDGYIKELYEKKILKGKQPFISAAEGIETLKSELIAFQ

>TpapIR84a

MDRRDFHGFTFTASIMMIDIVFNETNLLEPLLDKTYEPGKDMVRRYGLAVFMHLAEFYNFSYNFILTNAWGDPLHNGTWTGMVGQIARGEAEFGLAPAKFIVQRYDIIDYITSMHIVKCCFTFLQPKLFGSAKALVLPFHDAVWVCLAVIGAISVVVFRILSRYDSTSLSNDSWEGSTLLVFGAISQQGIPDNTEKVSTRIVYIFLLIVSFFTAVYYNTAILNGLLLPAPNAIQDIEQLLKSDTKLGMLDIPYLRNEFIQNDTMTVRVREKISKAKTKQIFYSITDGVRKIKKGKFALFTEDEAIYTEILHQMTDAEVCSVSEVLKYNPFHVGAVAKKNSPYKELFNRAFTLMRERGILKRQLEHWLVKKPQCQWKQDAISLGMEPLALAYAIISFGVIGGLIFLSLEILYSRRTRKHFKKNA

>TpapIR7a

MIFFAVFITLAHYGSCAIPYKYSLEEYPALSVCLLKILEGEVHTKNLVFIKEEVDKDKNFANLLKLNWDRMIVSTYNVSNVSGLFLFLPNINMTISYEKIMRIKHSDYNSKYIICTDNMKNIIDLFDIAWEYGVIDVIALLPSRRTGRISVYTYYPYGPFGCSTTDPVRINIYDPEDDNFESNESVFSKTKKLYNLYKCPVQITVSDRPAEVISNDNIESHNGISVSLFQFFQEHMNFTPQVTLIDREANTYAAAYYNFTSAVVLTIIKGKSDIGLGRFSQLMDYDNDVEYPAQTGMDCFTWAVPMKAGTKPSVWTTSVFEFDYITWIFIILSIIIAILMFRFLNKLPCFLERHDGLTIFASFLSIASNMNIKSQSTKIFLSSWLLYCFVITAAYSASLGSLVTVPPDSSDIETSMQLLDKDFVLAGEPKMYHILSASSETSPLIKSILNKFVILLPGEFKEIIHKIYSTRKLAVFYTKSKLIQEQNRVIEETQCGNMVHIVPKCLITSHTSPIIMKKGSVLMEPVKEIVVRLLESGILHYWDHKEEDDLLLNVTTTVQKFTVSQLRGALIVTITGYVISSIAFAYEILSAKFDHSTKSITKGEDIKILPHMKNGYIP

>TpapIR7b

MIFFAVFITLAHYGSCAIPYKYSLEEYPALSVCLLKILEGEVHTKNLVFIKEEVDKDKNFANLLKLNWDRMIVSTYNVSNVSGLFLFLPNINMTISYEKIMRIKHSDYNSKYIICTDNMKNIIDLFDIAWEYGVIDVIALLPSRRTGRISVYTYYPYGPFGCSTTDPVRINIYDPEDDNFESNESVFSKTKKLYNLYKCPVQITVSDRPAEVISNDNIESHNGISVSLFQFLQEHMNFTPQVTLIDREANTYAAAYYNFTSAVVLTIIKGKSDIGLGRFSQLMDYDNDVEYPAQTGMDCFTWAVPMKAGTKPSVWTTSVFEFDYITWIFIILSIIIAILMFRFLNKLPCFLERHDGLTIFASFLSIASNMNIKSQSTKIFLSSWLLYCFVITAAYSASLGSLVTVPPDSSDIETSMQLLDKDFVLAGEPKMYHILSASSETSPLIKSILNKFVILLPGEFKEIIHKIYSTRKLAVFYTKSKLIQEQNRVIEETQCGNMVHIVPKCLITSHTSPIIMKKGSVLMEPVKEIVVRLLESGILHYWDHKEEDDLLLNVTTTVQKFTVSQLRGALIVTITGYIISSIAFAYEILSAKFDHSTKSITKGEDIKILPHMKNGYIP

>TpapIR7c

IFFAVFITLAHYGSCAIPYKYSLEEYPALSVCLLKILEGEVHTKNLVFIKEEVDKDKNFANLLKLNWDRMIVSTYNVSNVSGLFLFLPNINMTISYEKIMRIKHSDYNSKYIICTDNMKNIIDLFDIAWEYGVIDVIALLPSRRTGRISVYTYYPYGPFGCSTTDPVRINIYDPEDDNFESNESVFSKTKKLYNLYKCPVQITVSDRPAEVISNDNIESHNGISVSLFQFLQEHMNFTPQVTLIDREANTYAAAYYNFTSAVVLTIIKGKSDIGLGRFSQLMDYDNDVEYPAQTGMDCFTWAVPMKAGTKPSVWTTSVFEFDYITWIFIILSIIIAILMFRFLNKLPCFLERHDGLTIFASFLSIASNMNIKSQSTKIFLSSWLLYCFVITAAYSASLGSLVTVPPDSSDIETSMQLLDKDFVLAGEPKMYHILSASSETSPLIKSILNKFVILLPGEFKEIIHKIYSTRKLAVFYTKSKLIQEQNRVIEETQCGNMVHIVPKCLITSHTSPIIMKKGSVLMEPVKEIVVRLLESGILHYWDHKEEDDLLLNVTTTVQKFTVSQLRGALIVTITGYIISSIAFAYEILSAKFDHSTKSI

>TpapNmdar1

MIIVASYTANLAAFLVLERPKTKLSGINDARLRNTMENLTCATVKGSAVDMYFRRQVELSNMYRTMEANNYNTAEEAIHDVKIGKLMAFIWDSSRLDFEAAQNCELVTAGELFGRSGYGIGLQKGSAWADAVTLAILDFHESGFMESLDNKWILQGNTQQCEQYEKTPNTLGLKNMAGVFILVAVGILGGIALIIIEMAYKKHQIRKQKRMELARHAADKWRGVIEKRKTLRATIAAQRRLKSNGLPETGVSLSVEALPRGVIDLPSPVRAWSGMRQRGVSELPNLP

>TpapNmdar2

MSSALWVCWGLLCGHLVAFKAPKSWPNKFLINVWGGFSVIFVASYTANIAALIAGLFFHSATATPNDNSLLSQRVAAPEGSAADYYVQRANRMLWEHMRRIKVPDVSVGVQMLKNGSLDILIADSPILDYYRATDQGCKLQKIGDQINDDMYGVGMIKGFPLKDSISSVISKFTSNGYMDLLQDKWYGGLACFTVDNDMSQPKPLGVSAVAGVFLLLGLGLLMGFIILMVEHLFYKYTLPILRHKPKGTIWRSRNIMFFSQKLYRFINCVELVSPHHAARELVHTIRQGQITSLFQKSVKRKEHEQRRR

>TpapGC3822

MWFAIGSLMQQGCDFLPKAVSTRMVAGMWWFFTLIMISSYTANLAAFLTVERMDSPIESAEDLAKQTKIKYGALQGGSTVAFFRDSNFSTYQRMWSFMESARPSVFSDSNSDGVERVIKGKGNYAFLMESTSIEYVIERNCDLTQIGGLLDSKGYGIAMPPNSPYRTAISSAVLKLQEEGKLHILKTKWWKEKRGGGACRDDALKSSSTANELGLANVGGVFVVLMGGMGVACVIAVCEFVWKSRKVAVEERASFCSGMASEIRNAIHCKNNDPAKEALENKTPEKKDLPVFLPQGNFPPQYGFLSNGPL

>AlucIR 25a

MLSPAATTTGIATLTFFILWVCFQTAHSQSATTINVMLIKEERNDIARMAFEVTQDYIKRNSKLGLEMNVYKVTESGSDAKLLLENLCETFNASAQAGKPPHMILDTSVVGVTSEAVKTFSKALGIPTLSASYGQEGDLRQWRALEGEIAKYLLQINPPADIIPEIVRSIVKLQNISSAGIVFDESFVMDHKYKSLLLNVPTRHIMGQVRSIQEIRNQLTRFKELDIVNFFILGSLSTIKNVLTEANGMKFFDRKYAWHAITQDKGPLKCDCSNATILHVKPEPDPGSKERLDNLKTSYNLMEEPEITSVFYFDFFLRGLLAAKSLLEKANWPKDYNKTSCDDYDENHDFVRKDLDLRSSLREVKEPYSYAPFLIESNGKSFMEFNMKIEKVVIVNSIAESAEAIGTWKAGLNNPIQTKDQASMRNFSAVTVYRVVTVKQKPFVIETFENGKPKYSGYCIDLLEEIRSFVHFEYDIYVAPDNAYGNMDASGNWNGMIKELIEKRADIGLGALSVMAERENVVDFTVPYYDLVGITIMMKKQTTQTSLFKFLTVLENEVWLCILASYFFTSFLMWVFDRWSPYSYQNNREKYKNDEEKREFNLKECLWFCMTSLTPQGGGEAPKNLSGRLVAATWWLFGFIIIASYTANLAAFLTVSRLDTPIESLDDLAKQYKIRYAPVNGSEAMTYFQRMADIEERFYEIWKDMSLNDSLTEVERAKLAVWDYPVSDKYTKMWQAMKEAGLPATLEEALERVRSSQSTSEGFAFLGDATDIKYLEMTSCDFQIVGDEFSRKPYAIAVQQGSPLKDQFNNAILQLLNKRKLEKLKEKWWTENNDRKECDKQEEQSDGISIHNIGGVFIVIFVGIGLACITLGMEYWWYKYKKPVSPKQVGQPAQTIPGNATNKQLSVTGLMDYGSREPRARYPIRRANANGPLDYDRPSFPAQQERLSHW

>AlucIR41a.2

MKSPRVGLIFLITTSVCNICFGIMRTRSDDEEFNLNMVHLARQVAKDYMSDRRGCIVVISDEGLLDDFTGYIDTTVLRVLFNGSKTECDPTMHKYILNAFYEKCTKYIVQISKPTCFFPAWFLARNGSTYEKHNPRVLFLPVKPHATEYGEEVLAMNQTNISHDVLIAETSPQNLSGPIGMPNELDPNRPITLYTNNFWQYVGEPGRIGRIYIDEWSLELGLRKGVDLYPDKVRDLRGKVLRLSAFPYLPYGNNEPMDGSEARILLEFCVVYNCTVVDVDDGHLWGQIYPENGTGVGEAGTIYMELSDFGVGANYLWLEFWPYLEFSNCYLYGALTVMVPKPELLSGLLTPFLPFPLSLWLVIVLCVFVSAVGLHWVTEATIKFAPHFLEEIYMNHKFITYTDSMIRSIGMLVLQQPQRLVPGSPVRHLFTAFEFTYLVITSAYAAELYDFLTIPRTTKPINSVVDLAESNIIWMTDHEVWVFGILHAEDPNIKKAASNFRAYPTPQLIEMSESNTPYGLGIERMAGGHYTELPYITDKFINKSRVMKDNYYVSPLVVNMQKGSPYANRLNDIIGRMQNGGLYYAWEADCVRKYLNYTKQLDMQWSSRPIKYPPKILNVADLEGAFLLYFIGTAMSVGFFVLELYLKKGLKLKDSFLNPTPKWFDDYLMGEGEPHG

>AlucIR41a.5

MRNFFLISFLLILTNLMQISCYEDIEHEELTSRLAEEIAEKYFSIDTQNTCITIVADLGVLQKFSAVNFSLVRISSNSPNDSCDTSMAEFIATSLREKCARYIVQIARPECFLPAWFEAIKNTIFERHNPKIIYLPVNDQENSSYGDALLATNETNISPNILVIENSEEEEEWPLKIYTNNFHQLVHEPERSPKIYLDDWHPSVGFRYGVNLFPDKMRNLQQKTLRLFTVPYVPYSNNDPIDGSEVRMVKEFCNVFNCKVEGVYDGMQWGEVYKENRTGIGQVGALYTENADIGVGANTYWLEYWPYIDFSDCYLGGAVVMIAPKPQVLGGWLTPFLPFPMDLWLLVMGVVIASAVGLYLLTNLTMKVSPSLAKKT

>AlucIR41a.1

MEGIRIVMILEVALLFSGSTKALNFTQNTESIEDALTTLAEEISTKYYVQISKGCIVLMTRPGFMGTFSVPGAAIIRLVSDNFCNDTDALNAMVTVFDEMCYNFIVQISEVHCFFKVWLDAQWKTIQRWHPEILFLPAYPEQTETADDVFTLKESDYSSSILAINIDETNSDWPFSIYTSNFYERVDKPGRNPKIFLDQWSSYASFRHDANLMIDFIQNLQNKTLKIMTFDYDPYTHFEPLDGTEIKLIQEFCKKHNCSLVAVDDGHYWGDIFENGTSDGLAGMVYDGRADFGAAAVYLWLPYFYFVDYSTSYLYSASTLLVPKPHPVSGWRTPFLPFDMLTWISYGLSVLMAAVFMYVITYLTVKYTRFAEAVQKRRMFLDKLDCIFRALGLAVLQQPSTPLVPHTPIRHLFTSFEFLFLIASSIYAAELASYLTVPRYEKPIDTLIEYSDSGMIWIGEHESWTYSLRGMTDPEIVNIVNNYLICSHEKLMKLAPTGEYGLIVERLPGGHYTEQDHVTDDIVAQSHMMAENLFGSPPVIAVRKGSPYRKYFNKVISNVLCGGLYLYWEGEMSRKYLHSRRQLALREADHPHYKDIPKNLEISHIQGGLFLYAIGATISIFVFMFELFFHYRKMSRKFIRKFT

>AlucIR41a.4

MFLQFCFKVALTVTLVANTAGDESHNHLLDDVNSLVREVTDKYMKSFNCIVWVEENDPFLNLSPSFSNSMIFIDGYDSENILLQVFFKKCQGWILQLSDPSKFMKKWEEARLGSLVRFNPRVLFIPWEVSTYATALFQTPEFDYISNAVAVELTRSNGSLQLTVVANNFSANIDEKNETMKVHLGNWPLNSSVEIYPHNLGNLQGKELRVATLQYLPYSQLYPDLDGVEVRILKAFCKHCNCVIKPVIDDFLWGERFDNGTSNGIVGNVLQDKADVGVGAVYLWYYDHLEFSYPYMPSRVTVLLPKPSPMPKWRIPFAPFDLALWIALIASIITVALVLFYMNYYLQRFSTHYVAPNEFQSWSGVVLRAVGMAVGQSPQNAFSTGSTLRIVFTTFELLFLLYGTIYSSALASVLTIPKYYPPIDNMRELSASGLPWTADHIAWVQNLLAADEPHIKVLLRKFEVHDDNVLSQLAKKGGYGFTIELTTGGHVSEASFLSADMINDLHVMKEALYYTYSTSITRKGSPYVEELNKLLHKCFDTGLLQLWESEMISKHGSSSIQTAFKQSKSAKASNEPHNLEKLKFKHTQGAFILLFLGNVIAIIVFMCEYYCKRVKRIDKPTKLH

>AlucIR41a.3

MGLQRSSCISAYSFLMGAIVLSTAQADVSKESLTITDVLENIIITKYFDSDKCIVHIVDEPAQQLSTINNLSHPSIIRISSFDVCDDPSLSDKIVFAIDSCCFSFILRVSKPVCVIKAWGKAQLSYEIDTFQRKSPKMFVTVFPVNDSIGDVDEFLSSPETELSSSIVVAVLGGGDPDWPVTLYTNNFYEPVSSAGRQPAIFLDRWNQEKGFQLGTDLYADKILDLQGKEIKVAVVDSLPYSQLRPFIGQEASMMTEFCLSRNCTIKGINDEWFWGEIFENGTGNGLVGMVFDGRADFGIAGVYLWASVFKHVGFSETYLQSGVTLLVPKPEKVGGWLIPFLPFSPQMWISYLLSVIATGISMHLITKATIKYTRFAEAVEKRGMFLTEVDTTFRALGLSVLQQPSTPLVPHTPIRHLFTSFEILYLVFCTVYAAELASVLTAPIYSKPIDSLEMFAGSGMTWLGEHNGWVYSLLTVETPIIKTIVNNFKLVTFEEMDELAITGKYGLIIEKLAGGNYAERQYLTEKIVSQSHVMAEYLYDSHVTTIMSKASPYFEHFNQLIRRLLENGLLLRWEEEASRLYMSYRLQSALHSARKINTEDEVRPLTFSDCLGMLLLYLAGIFISTAVFIGEVWTNKKKREREARFN

>AlucIR21a

MRIEFFLLVVIANLAVPTKISKLLESLAVVQHESIACMPPDGVPYMVPLLNAIARRYLKDHVTVVLYDDYFYFHPRLKSMLDHILLNYAYPLRHGRINTTMAKPKVPPGILEARENEQMAFIVFTKETEIGAESIREYTSRNTMTLLIAPTSVYMVRQFLGTKLAGDITNLLVIVDPMIRVKLLSQKVGQVFKECDILIYSHEFASDSLGSTTPVIVTAWRRTQFTRQVQLFPSKFKQGLGGIHLTVAASEIAPFVFRKRGQESGAGYTITKWDGIEIRLLNIVSQMLNFTVEYKEPELIEEEDVAQAVIKEVLAKKANLAVGGVYLTPERINGLLFSIPHTRDCASYISLSSTALPKYRAIMGPFLWDVWLALIAVYLFAMFPIAFSVWHSLKPLLDDLWEMENMFWYVFGTFTNCFTFSGENSWGKSERTATKLFIGTYWVFTIIITACYTGSIVAFITLPVFPKTIDTSKQLLEEDYKISVLSSGGWERLYNETDDPVAVKLYKSVNLVPDLSAGLLNVTRNVHSWRQSAFLGSRRLLEHTVRTNFTPDEDSKRLLFHLSDECFVPLLVSIVLQKRTHYLEEINGALERALQAGFMTKVTQELEWEEYRSATGKLLKVHKGLKGAPEDRELNLDDTQGMFLLLGAGFAIGLFVLIIEVSVWSTGQVKHRQFGDLTLKQRAFNKLKEHGEALYNCLLAPAKSGIIYFRERRVSSAFGEYVTRPYTPWPRSSIPSPTVQSPSPPPPNGMVSSAPPIDVGADSLSMDQLPLPRERPVRLMSF

>ClecIR25a-X3

MLKLAVQVLLLYVPSVVPQTRSINVMFVNDVRNELADLSFQVALEYVKRNRKLGVHVEGITKVVTEGSDAKAILEDLCHKLNESSAEGKFPHLILDTTMTGVTSESVKTFTAALNLPTISGSFGQAGDLRQWRHLESEQLKNLIQINPPADIIPEIIRNIIQIQNITNAGILYDDSFTMEHKYKSLLLNMPTRHIIADISDARSVRQQLNRFRELDIMNFFILGRLAMVKSVLEIANILKVFGKKYAWHAITQDKGNLICACNNATILHVKPEPDPESKERLDNLRTSFNLVAEPEITSVFYFDLFLHAFLAISTEVDRNWPKDYNKTSCENYREEALSGWKTIDLRTALKSVQEPYTYAPFVVESNGLSYPEFNMRLEKIQLKDSNGMINISAITVYRIVTVKQKPFIIEYFEDGKTKYKGYCIDLIEEIRSMIPFEYDIYVAPDNSFGNMDENGNWNGMIKELIEKRAEIALGSLSVMAERENVVDFTVPYYDLVGITILMKKPTTTTSLFKFLTVLENEVWLCILAAYFFTSFLMWVFDRWSPYSYQNNREKYKDDEEKREFNLKECLWFCMTSLTPQGGGEAPKNLSGRLVAATWWLFGFIIIASYTANLAAFLTVSRLDTPIESLDDLAKQYKIRYAPVNGSAAMTYFERMASIEEKFYEIWKDMSLNDSLNEVERAKLAVWDYPVSDKYTKMWQAMKEAVLPTSLDDAVQRVMDSSSSGEGFAFLGDATDIKYKVMTSCDFQVVGDEFSRKPYAIAVQQGSPLKDQFNNAILQLLNKRKLEKLKEKWWTDNPERVECEKQEEQSDGISIHNIGGVFIVIFVGIALACITLGFEYWWYKFKKPAVDVQQGAVMVGKQTKTGAGGGIDKLSITGLADYTSQPYYRPKFHARRTQYR

>ClecIR25a-X2

MLKLAVQVLLLYVPSVVPQTRSINVMFVNDVRNELADLSFQVALEYVKRNRKLGVHVEGITKVVTEGSDAKAILEDLCHKLNESSAEGKFPHLILDTTMTGVTSESVKTFTAALNLPTISGSFGQAGDLRQWRHLESEQLKNLIQINPPADIIPEIIRNIIQIQNITNAGILYDDSFTMEHKYKSLLLNMPTRHIIADISDARSVRQQLNRFRELDIMNFFILGRLAMVKSVLEIANILKVFGKKYAWHAITQDKGNLICACNNATILHVKPEPDPESKERLDNLRTSFNLVAEPEITSVFYFDLFLHAFLAISTEVDRNWPKDYNKTSCENYREEALSGWKTIDLRTALKSVQEPYTYAPFVVESNGLSYPEFNMRLEKVSIVHSLSESAESIGTWKAGFNSQIQLKDSNGMINISAITVYRIVTVKQKPFIIEYFEDGKTKYKGYCIDLIEEIRSMIPFEYDIYVAPDNSFGNMDENGNWNGMIKELIEKRAEIALGSLSVMAERENVVDFTVPYYDLVGITILMKKPTTTTSLFKFLTVLENEVWLCILAAYFFTSFLMWVFDRWSPYSYQNNREKYKDDEEKREFNLKECLWFCMTSLTPQGGGEAPKNLSGRLVAATWWLFGFIIIASYTANLAAFLTVSRLDTPIESLDDLAKQYKIRYAPVNGSAAMTYFERMASIEEKFYEIWKDMSLNDSLNEVERAKLAVWDYPVSDKYTKMWQAMKEAVLPTSLDDAVQRVMDSSSSGEGFAFLGDATDIKYKVMTSCDFQVVGDEFSRKPYAIAVQQGSPLKDQFNNAILQLLNKRKLEKLKEKWWTDNPERVECEKQEEQSDGISIHNIGGVFIVIFVGIALACITLGFEYWWYKFKKPAVDVQQGAVMVGKQTKTGAGGGIDKLSITGLADYTSQPYYRPKFHARTQYR

>ClecIR25a-X1

MLKLAVQVLLLYVPSVVPQTRSINVMFVNDVRNELADLSFQVALEYVKRNRKLGVHVEGITKVVTEGSDAKAILEDLCHKLNESSAEGKFPHLILDTTMTGVTSESVKTFTAALNLPTISGSFGQAGDLRQWRHLESEQLKNLIQINPPADIIPEIIRNIIQIQNITNAGILYDDSFTMEHKYKSLLLNMPTRHIIADISDARSVRQQLNRFRELDIMNFFILGRLAMVKSVLEIANILKVFGKKYAWHAITQDKGNLICACNNATILHVKPEPDPESKERLDNLRTSFNLVAEPEITSVFYFDLFLHAFLAISTEVDRNWPKDYNKTSCENYREEALSGWKTIDLRTALKSVQEPYTYAPFVVESNGLSYPEFNMRLEKVSIVHSLSESAESIGTWKAGFNSQIQLKDSNGMINISAITVYRIVTVKQKPFIIEYFEDGKTKYKGYCIDLIEEIRSMIPFEYDIYVAPDNSFGNMDENGNWNGMIKELIEKRAEIALGSLSVMAERENVVDFTVPYYDLVGITILMKKPTTTTSLFKFLTVLENEVWLCILAAYFFTSFLMWVFDRWSPYSYQNNREKYKDDEEKREFNLKECLWFCMTSLTPQGGGEAPKNLSGRLVAATWWLFGFIIIASYTANLAAFLTVSRLDTPIESLDDLAKQYKIRYAPVNGSAAMTYFERMASIEEKFYEIWKDMSLNDSLNEVERAKLAVWDYPVSDKYTKMWQAMKEAVLPTSLDDAVQRVMDSSSSGEGFAFLGDATDIKYKVMTSCDFQVVGDEFSRKPYAIAVQQGSPLKDQFNNAILQLLNKRKLEKLKEKWWTDNPERVECEKQEEQSDGISIHNIGGVFIVIFVGIALACITLGFEYWWYKFKKPAVDVQQGAVMVGKQTKTGAGGGIDKLSITGLADYTSQPYYRPKFHARRTQYR

>ClecIR93a-X2

MTFNVVIVDPFFMYPKYKGIENKLRSILNEALLNNLKLNAVDVVYHMSTKVSLKHDTTAVLSITSCKDLWNFYQSQGKDNGILYLSFTDPNCPRLPAHVGLTLPLHRVGKEISQILLDLRSQKILDWRNTAVIYDDTINEDIVTEILKVLTQPIPLSTSYNSIAKYFLTNIHEVEWKRRKYIFDVLVHLPPTNLMSNFLVVVNPEIIPIVIEVAKSLNLLEPTTQWLFINSRRFRTKANNISNYIQLVEEGENLAFMFNTSKIDQNCNFGLECNMVEIAESFMLALARTIEVESKLANDVSEEEWEVMKLTKVERREMLLNIMKKAQKNLGKCDNCTVWKFKASETWGMDFLKNKRKIDLLDVGFWSPRPGPMLTDSLYPNIAHGFRGRVIPIATIHYPPWQIVKYNENGQPKEFKGVTFEIINQLALSLNFTYEIILLSNGTSTNNSIRTYKFDESLGEVVLDASVEFIAWDQIVRLINEKKIMLGGVAFTITEDRKEYVNFSSYISLESYAFLVSRPKELSRALLFILPFSTDTWLCIIAAILVMTPLLNFVHRITPYYDHFSHRGKGGFLKMMNCFWYLYGALLQQGGGVLPDADSGRLVIGTWWLVVLVIITTYSGNLVAFLTFPKMDKAISNVDQLMAKRDSLTWGLPKITTLHKLLKSTDSLKFNHLSDAAELHTEITPDIVARIQAGKHIYIARKSVLLFIMKQEFLRTNRCDFSIGEEQFLPEKLAIALPTNSPYLNIINKQIYKMHKVGLIEKWLVDYLPKKDRCWSSTLSSESNTHTVNMDDMQGSFFLLFLGVACGILLIIGEYFYNRWKISKEKSVIHPFVS

>ClecIR21a

MSICLILLFFQLYIQIKGADVNADIKGYDLLYEKYELLEAETDKCMATDGTPYITSLFNEICKLYLTNQVPVLLYDNDYSVNLKFQRILNRFLKIYPKSLRHGLINVTKAVPQVPNGILDPGQNEQLAFIIFSKEIEIGALAIVDKVEIVTKTIFIPKSSLYQIKEFLTRDIARKFHNILVLVDPTLKIDIYFQKKGQVHKECDINMYTHQLASDSLGGSKAIILTSWRRNNFTRNVNLFPDKFKTGFNGMHMVVSTSENAPFVFKSKGHDSGEGYTLNKWDGLEVRIMNLISQMLNFSVEYKEPDSDPKYANLSWTENSLRQLALKQVDYAIGGIYLTAERYKLFTFTHPYSQDCASFISLASTALPKYRAIMGPFLWDVWLALTTVYLLAIFPIAFSVWHTVKPLIEDISELENMFWYVFGTFTNCFTFTGQNSWTHASKTATRIFIGSYWAFTIIITACYTGSIIAFITLPVFPEVIDSSKQLVEEEYKISTLEKDIWLELLTGSVDPVAHELYKELDFVPNLIEGLRNVTRNIHSEHKSAFFGSKYLLEYTVRTNYTPEDSNKRLLFHLNKECFVPLFLGIALPKKSMQVEKINLALSRLLQSGFMFKVSREVEWAVSKMSTAKLLKASMSSFRIAPEDRELTLDDTQGMFLLLGAGFAIAIAALMAEILVWCVKEFKQTKFGQPSLKERSLAKIKQNLGEIKRCLFDPIDQLKRSFYESRERNVSSLFGSGVSIPEHQNEALWIINLKPPQEKPIRLRSF

>ClecIR40a

MSSLKTIFVVALFLMICESKTAHKNLMLIKCIKDMAAALPTKELSLVFDYDYDEDFINDFIISLFNSGLKAVVYQLDNPKQEASYLNKLLDDMKMRMDGYTSAEYFIFAKKSVAEDILQMISSENLARRNVIFLFFWKGKKISYKFSINIQEAMRICVITSPRYGLFKLGYSQARPDGKNTLDLVNWWTENYGLFQQPLLPKASKVYKNFKGRVFNIPVLHKPPWNFVTYNNDTIEVIGGRDDKLLRLLSSKLNFEYDYFDPPDRSQGSAIINGTMQGVLGLIWQKEVEMFIGDLTVTYERSQVVEFSFLTLADNEAFLTHAPGRLNEALALIRPFQWQVWPAVIVTIFISGPFLYMLILASTEWKITSISKLWDCIWITTSIFLRQSITDCFKVDRVRILIATMYLVSTYVIGDMYSANLTSMFARPAREKPITTLEQLDKAMAFKGYQLLAERHSASHAILENGTGLYKSIWDKMKGQLEYLLDSTEDGMKIVKKEKNFALIGGRETFFYDTRRFGVHYFHLSEKLFTRYSAIALQIGCPFIHNFNEILMRLFEAGVLTKITEDEYQKLKEKQPSKAPKKQTEVGEGKVQKENDTRLKAMSMKTLQGAFYVLIVGYIMAGAALLGENIMKHQMEKPKRTKRTSNAKD

>ClecIR93a-X1

MLKLLAFLAINLDIFANCYYQNSIVGRSNDTLVVIVDPFFMYPKYKGIENKLRSILNEALLNNLKLNAVDVVYHMSTKVSLKHDTTAVLSITSCKDLWNFYQSQGKDNGILYLSFTDPNCPRLPAHVGLTLPLHRVGKEISQILLDLRSQKILDWRNTAVIYDDTINEDIVTEILKVLTQPIPLSTSYNSIAKYFLTNIHEVEWKRRKYIFDVLVHLPPTNLMSNFLVVVNPEIIPIVIEVAKSLNLLEPTTQWLFINSRRFRTKANNISNYIQLVEEGENLAFMFNTSKIDQNCNFGLECNMVEIAESFMLALARTIEVESKLANDVSEEEWEVMKLTKVERREMLLNIMKKAQKNLGKCDNCTVWKFKASETWGMDFLKNKRKIDLLDVGFWSPRPGPMLTDSLYPNIAHGFRGRVIPIATIHYPPWQIVKYNENGQPKEFKGVTFEIINQLALSLNFTYEIILLSNGTSTNNSIRTYKFDESLGEVVLDASVEFIAWDQIVRLINEKKIMLGGVAFTITEDRKEYVNFSSYISLESYAFLVSRPKELSRALLFILPFSTDTWLCIIAAILVMTPLLNFVHRITPYYDHFSHRGKGGFLKMMNCFWYLYGALLQQGGGVLPDADSGRLVIGTWWLVVLVIITTYSGNLVAFLTFPKMDKAISNVDQLMAKRDSLTWGLPKITTLHKLLKSTDSLKFNHLSDAAELHTEITPDIVARIQAGKHIYIARKSVLLFIMKQEFLRTNRCDFSIGEEQFLPEKLAIALPTNSPYLNIINKQIYKMHKVGLIEKWLVDYLPKKDRCWSSTLSSESNTHTVNMDDMQGSFFLLFLGVACGILLIIGEYFYNRWKISKEKSVIHPFVS

>HhalIR41c.1

MSIYHFYFYKTLRSEEIYKQMKLVLLLAPLIFHPSGGIELTEDPTSKSLSSLISKIFLDMPKCVVVVLSDFSSLELPNNFVYLRAQSNSSNLFSSLRASYKMKCKGYLIDENSANDFLTTMGKARSAAPERHTPRIVVIPNHKGNYSVDIFMHPESTYIPDMIVIQIIDDDKSEKGVELEVITNSFHNNSIFPEGEVLGIWPIDDMIFFPDKLKNLNGKELITATLHYPPYVLLHPIFDGMETRMVNEFCRLYNCKPIPLTDEYLWGEIFSNGTGIGVSGNVYMDRADIGIGALYLWENEYQYVDFAYPYLNTKITLLVPKPAQKAEWRIPFLPFSLTLWVLQVLSIVLAAAVMFVVNKVSSHIAQGLMLGGEFATGGGIFLRALAMSLLQPPPNRLPSGSPLRRFFTIFEVLFLFFTTIYSGALSSVLTVPLYYPPIDTPQQLYEAGIPWAADHYAWVFSIQGATEQPFKALTERFEAHSHDTLISLLKKGGYGFGIEIMPAGHVTQTKYLNGEAILNHHVMKHELYTTSLVMNLRKGSAYTEKFNNLIGKLLDAGLLLLWETDVTLTYLSSRLQTALKISEGTKVEVHHPTKLKLTHIQGAFMFLVFGLFVSFAVFMLEILGHSPSSPLKVYVLQKNP

>HhalIR92a

SSLCLEKCLSSSIIAINRTSVNSYLEVLANEIGEKYFSGHRCVVVMSDAGLLRNFKINTTVVHIEMNPTNESCVQEVADLLRITYRDNCDGYIIQSSNPNCAFDGWLDIYFHSSVARHNPKYLFLPVYPHLLTYGDELLSRRESDQTADFLVSEINENSTDWAVIISTNNFYVHPTVPGREPKIYLDEWHYETGFKENVNLYPDKVLNLKGKPFRIATDKYFPLTSAYPLEGSEVRISLYFCEKYNCTPEAVTDNSLWGTIYDNLSADGVLGNVYNDRADVGVLAVYLWLNEWYYIDYCTGYLAADVTVILPKPTKLSPWILPFLPFKYDVWIAFFISLFLSASSLYFITRVSIRFTRFRDQLIMKVQFTTIDDSLMRAIGLAVLQQPSSRLIGDSPNRYLFTSYEFLYLVLSAMYSAELASFLTVPLYYPPIDTFHAFAHSGIHWYATGSAWSNALKNSDEEDAKLIVNRFGVLSMDQLKEKIKEGKHGFTVERMAGGSIGEQDFQTSDVIPMYHIMKEKLFGSPLVGAIKKGSPYLKHFNEVVLKLVDYGHYLYWENDVARQYLGSSIQAAYEEAKTVQIDDSPAVIQISNIQGVVLIYTIGIIISIIVFFYEIITYRKNENSLLIEKKK

>HhalIR75b.2

PLKVNLYLCIYYIKVFWSSMVNYAFYFDIVNKYFNFLHIQSVVMLICPQEAVKVNVMKEMSDRSFLLSFSVDSIRDNPIIRTGTVLDISCHNTSLILQMMSARKMFNSDMEWLLMEEVNTTESFQQTLENDVLKDTLALPGSCVTLAQFSENERRVKFYEVYRTAIWEPMKFRFLTDYFMNGSKEFSFRRGYRSFEGVVLRTASAVLYPGLFLGWESQELKEVDTISKAGYAFMKDIADHLQYNYTLLFLDFYGYETNGSFNGIMGYMQRGDIDVAANGLMMNQERMPYVDFVGDILVLRSPLIFRQPSLSTVSNLFVLPFHKTVWMLTVVVTLLYSMALFLNLYLKMRLLRLKETDDSSVPEIISVIMAYVCGQGTGLELRPGAGRITLCILSVFCFFLSVSFSAKIVALLQSSATTIKSLSDLTHSPMSVGMQNVFYNTHFFSISTNIEVRELFQKKILPLGDKAIMKPDIGIQKVKEGLYAYKVETPWAYTIITRTFEDKEKCDLDELNPFPLPTLAVGLQEKSGYKEPIARSFSRLLEVGIKRRTMNIYYPQKPFCNSNNIGYTRVKLTDFKPALDFILYGLLSSFILFLFELLFKTRYLFRRIVRKEWPAHKEVI

>HhalIR1

MKVLLFYFSLFFVFFCTKGNIELNFNFNNDYLFPIDSTYLIEMMNYTFRDNYCVQLVADESIIANRIREAMFNVNFLRGVSDKRGRFQCINCVFIASTVEIFINILKTRPVDTDKFLSIIININDENVLYELSHKDVTNYLTNWHGVVIDLKAPHFLYRYIPLHKTFKTHNLSDIRNREIGINYGSFGGRKLRVGTFNCSVNSQIGPLDAKGRPSWFGGVEMIFLDAITQKLNFTYEIILPPDGEGVGGRLDKYGNLSKGLVGLVLDQSVDVAFCGIWQTNYINTRSLSISSPIQEVCITYLVPRPLPFNQMGLGLFTSFEGKVWMLIFISIITTAISSQLIAFMAVKHGFSNFQLIKFLKPNNSLLQLWSILCNNAPKSLATFGPLRHVLLWWAVYSLLLNAMFSSSLVSHLTATLYDKPLDSIQALVENDYYWSLNGMPYLGSILNSSNEWGKKWVKRFHFKSEKSLCSILQVDNNKLAMFAVNYDYGVMLEENIECKHLLRKYQTLPDCISKYRTVFLLRHRSPYRELFSDYITRYRENGMFQKQYRELFYKKILKDNYISEVRLPLNINSAKKVKLRLSNTLAIFYIYGLGIMISFVVFLLELLWYRKSYMLRIFV

>HhalIR41c.2

MIRLKNMFHAIMVYLLLHCSYGLIKQEDTRSQKLNKLISQIILEMPDCLVIVMSDSNDLKVPKDIIQMRFSPSSDELPAFLKEAYELKCDGYLVDQESAGYFFISLKIARLHANVRLVPKIAIIPNKIGEYSEALFRDKETAFIPDLTIIQIDNSTTEERFMVVTNDFQGTNRKLKGDFIGIWPDDKIVFFPDKMADLKGKELYSSVVHYPPYVLAQDPVEGVECRPQLEFCRLHNCSLKIRTSEYLWGDIFPNGSGNGIFGEVFLDQSDFGVGGIYTWLEEFQYLDYSTPYGIGRINVLVPKPTKVDEWKTVFMPFSPTLWLLLVFSILVAATLMHCANIAAGKISDELILGGEFTTWVGIFFRAIGMSLLQPPPSILSASPLRRFFTIFEVLFLMFTTIYAGAIASVLTIPKYNPAIDSALQLYESGLRWVALHEVWILSLVESTEPYIKTLVGRFETQDEETLVRYTKEGVYAIGIELMETGQMIEASYLSVETVANHHIMKNDLSWSHVIMLHRKGSPYLEEMNRVIGRCRAAGLFLLWATNITANHLSSRFQIVMQISKGSSKVEIDEPIKLKLLHVRGAFMLFIAGMSLSSLVLMVEIIKFKIFNTKSRKLI

>HhalIR84a

MKLLRMIFFIFCYEGTWSSSRCYIPDGIHDVIDFYFRDLFGAHLYFCKLEDAVKAFKRFTSGGPKYNIRHNRDFREPHELLMSYWQPSRLGIFLDTSCDHGIFFFNYTTDLFNASYSWIVWSEEVNFTMFEDTRLSADSEVKVVKPGLEIYDIHRVHISSELKSKLVASWNATDGLVQISPSMDRRDFGGFTFTASIMMIDIKFNESNLLEPLLDKTYEPGKDMVRRYGLAVFLHVAELYNFTYNYILTNAWGDPQPDGTWTGMVGQVARGEAEFGLAPAKYIVQRFEIIDFINSMHIVKCCFTFLQPKLFGSAKALVLPLDEIVWVCLAVLGILSVIVFRILAKYDNTGLSNDSWGGSALLVVGAISQQGIPDNTEKVSTRIVYIFLLIVSFFVAVYYNTAILNGLLLPAPNAIQDIEQLLKSDIKLGMLDIPYLRNELIQNDTMTIRVREKIAKAKPKQIFYSVPDGVRMIKKGKFALFTEDEAIYTEILHQMTDGEVCSVSEVLKYNPFHVGAVAKKNSPYKELFNRAFAVMRERGILKRQLEHWLVKKPECNWKQDALSLSMEPLALAYALFSFGAIFSFFILGFEIIHSKKSKKKAFD

>HhalIR75h.1

MRLQVLLIFALFSTCTCKNMTDYFEIINIYFSYRNVKSIDIFPCTESEGYWMFKRLLKDGYSTRVNRNLLDKEFQELPYIKGWFVDLTCSKNIIFFSNNVVQITGLDGIWLGYPSVASDLNMTSLRLDSDVMVGSADGRLWDLYGKPLERMILNGAGTWRPANISIPKIKFRYDLGGVVLKGVLVELDLQFFANNITHKIADHDYFPDMEMTQRVSYIFVTYLAKFYNFWFDIVPTDTWGANLKNGSCTGMIGILQRKEADIGISACSFRVERVEVVSFAQRSQELRFICMFMEERIHGTYSSLLIPFSFQAWLCVFVAIVVGATIFSFLQKRDLTEGIIFVTAILSQQGLQKDYRSISARVYSISLLILGLVLYSYYSAAIMNGLLSPAPGSIRNVDDVVKSPMKASLARVPYMIPKVNQKAYVTPELLAKTKRQEKSQQILDVFVGVDRIRHERLTLVADDMSLYAIINEMYTDAQKCNLMEIEVIRSFPFGNPLQKNSQLREMMSQGTLRLRENGIAKREMRVWYHPKPQCLGSTTYTHVTLEAVGLAFTLFMTGVVLSIFILLTEIALKIFLKYNLFSKKFSKTQARPFLR

>HhalIR75h.2

ESMYRLVILLLIGSTHSLRYMNNTLEIITTYFNFRATNVINIYSCNHREGVWLLKHLNALGFMIQIKHHTFDEKEESRPPYVRGWFIDLNCTKNMNFFKTINGLDGIWLGYPVKSLDMNLTKIRLDSDVVLGTEDGLLWDLYNNTKRNLKVSSAGTWRPPKIFMPNSKHRYDLNGTVLKGVLLELELQFFKNNMTEKIGDYSYYPEWETSERLAYVYVQNLAQFYNFEIEIVPSETWGYVLPNGSFGGMVGVIERGYADLGLTACAMREDRMRAVNFVPQLQDLRFICLFMEERIRGTYSALILPFSFNVWLCIFVAIVIGAAIFSLVQNKDVTEGIIFVTAILSQQGLLHDKNRMSARVYSISLLFLGLVLYSYYSAAIMNGLLSPVPPSIHNELELQKSTMKASIAKVPYLMATFSQKAYLTSGMFAKTREHEDTMMDPFIGLDRVRKERFILISDDLALYGILKVAYTDLEKCNVREIETMRPFPLANPIRKDSPFREMIAQGMLRLQENGMGKRDRRVWYPPRPHCFASTIYTHVTLEAVGLAFTLYVLGIVLSFTILLSEVVMKRFKKFKRTDPDTEFQGYY

>HhalIR8a

ELLWDLLRQMSMIWRAIFAIGTALFLASDVGSVKLLILKEENGTIWENVKTEEVWEEQHVILNRENEEESFNKVCEELSSGAWMVLDLTWSGWDSVAEIAGIRYLRADLGISPFMRATELTLIKLRNSTDAALIFQHSHHFEQSLWYLVRESSLRVTVHLGLDDESAESLLDMRPSPSSYVIFADHDKANTILKKAVDKKLVYLDDRWAMVFMDIGTAPINKTMLKKRIMQVYMPKSICCTDPGQMLPCHCTEPFNFEIEGAKQLKIALSKAISSSLTEGLSAEPLNYSCSSSALSPKNDSIFYDNLDKALSDSWLLHKTYGEVRLNLRFEIRNIGENRAQQLGNWDPIAGIRTAAMPRVKRFFRIGTGFSTPFAYPTNQISPDGSARWEGYALDLIERLAESMKFEYQLITVENHMFGQRFENGSWNGLVGMLATGRVDMVVGSLTMTSEREEVIDFVAPYFEQTGFSIVLRKPLKKTSLFKFMTVLRVEVWLSILGALCLTALMIWFLDRYSPYSARNNKEKYPYPCRDFTLKESFWFAVTSFTPQGGGEAPKSLSARTLVAAYWLFVVLMLATFTANLAAFLTVERMQSPVQSLKHLARQSRINYTVVKDSDAHNYFRNMKFAEETLYKYWKEITLNSSSDQSQFRVWDYPIKEQYGHILNAIEKAGPVANIQEGLRKVIESEQAEFALIHDLLELKYHVFKNCNLTLIGEPFAEQPYAVAVQQGSHLNEEISRRILDLQRERFFEATASKYWNSTIKSKCDNVDEDEGITLESLGGVFIATLVGLFIALLTLGAEVWYHKKKSKNQVTMKGQQQLQSIIRDEIFTTKEFGHHNFANRKASLLATKPKVKQITVYPRGQLY

>HhalIR75d.2

SQCNTMLGQIHTIIDYFKALKISSLSLLLCLSEEMKILALRQFSQEGFQISFGFSSDSRGTVLDLNCNNQTGGRMDGEWLLLGKAEDATELRALPDSKVTLLTEEGVLMDIYRPTMSSLLSFANYTGQRDHSDLKGETLKAVTVEDEGTSVRLEEGGDLFGHYGCLCKNAGGWSPQMSEVLVENLKQQLNFKVKRKKVTDCNALYSTLSEGAVDFSSTAIMILPEKMEYSSFTGDLFEYKSMILFKHPRLPVMKNIYVLPFHRYVWFCCVGLFFLCFGLLLAAARSYPGVFEEYTYSSPSDILTLLIGVASQQGIDLGARSPSTRLALLLFSVCSLFLSTSYSANIVALIQSGAETITTIADLVQSPLSFAVQDVSFVKVYLKNGEKEVKDLYETKIMKGNSNPFISAERGVEKMKNEVFAFHVETSIAYNIMSKTYTSQEKCGLSELHVYTYPRFSIPVIKDSGYRDLFASRLSRQRECGLIKRAELLALERKPACTAKDSGFVSVTFGDFLPGLLVLCWGMVAAVIALFAEITMSKCTLLAKQEKKKEK

>HhalIR76b.1

DIAEGRLPAMQGLYYLLLALCSNYPPPPSEAEFTCKLRKGHPEKEILKGRTLKIVTYDDRPFSGATANASGALEGHGLVFEVLETLQEKFGFEYELQKEKRLMGDETSGLLGMLVAKDVDMIAAFLPILPGTHNYVTWGTQLWQAHYYVLMKRPDDSATGSGLLAPFDDKVWILILISLTSVGPIIYLIMWLRIKLCPNDNKQLFPLSSCIWFVYGALMKQGSTLSPLSDSARLLFATWWIFILILTAFYTANLTAFLTLSLFTLPIKEVEDVAKPPHKWFTTEGSSVEYAIKNKDDGDLNVLLSSVRRGNGRFIDTSSENHVLEMLYDGWLYLDTSDTLNRLMFDDYKRKTIEGEDENKRCSFALTQYPFLVRSLAFAYPKGSRLPELFNPIVQVFVESGILKHLLNEDLPDTTICPLNLGNKERKLRNTDLFTTYIVVLAGFSGALIVFCIELLWTYCATRSFNSKSNKLQRLKNNYNKFVIPSEFEKNAAIPNQVQTKINGREYFMITAKEGDKRLIPLRTPSALLFQYGLNYPTMF

>HhalIR75d.1

MGVHTEWLLLDDGSSLDVIKDAYILPGSFVTIAQILGEEVVYLDVYRTSPYRPLKYTVLDNQTLEQFIQLPDRPSRNDFEGISLLGAAVLYYPHMFCGFDCRDHPEVDTIAKTGFPISQHFQEQLNFTLTFQILNDYGWKTNNTFSGLMGLLQREEIDMGVIGIFMRPDRIGTVDFTGDTFEIKSLVIYKQPALSAVSNIFVLPFTRMVWVCCVALSVLTGIFLMADVATSFGNRQTFEESVTTSDVVTLIIGCICQQGTTLVPKTLSARIIIFLFSLCCLFFYTSYSANIVALLQSSSATFRSLSDLTNSPLGIGIQDVIYNKIFFGEATDENVRELYDKKIAPQGPKAYLNPVEGIKKLRSGMFAFDVEIHWGYKIISDTFHENEKCDLDEMRIFLLPKLSIPVVKKSGYREYFTRMNTWQRDVGLHSRIRQRWLPKKPICDNTGRGYVSVGLTDFKPALLVMVYGISFSIAAFFLELFTRSKLFYRCKFLNKSKRKRRKFNDWDRVM

>HhalIR93a

DFLEKKELLESGDWSPRSGPVLVDQIFPNVAHGFRRKIIPLFTFHNPPWQIVKYDQNGKPSQVKGVIFEVVDHLAKSLNFTYEIILMSNTSLPANQTKFYRFNESVGDVVLDQSTEFLAWEQVVRLIQNKKVLIGAAAFTITEKRKKYLNFTLTIRTENYAFLVARPKELSRALLFIQPFTSDTWQCIVAAVLVMTPLLNFVHRVSPFYEHYSQREKGGYMKMMNCFWYLYGALLQQGGGVMPEANSGRLVIGTWWLVVLVLVTTYSGNLVAFLTFPKMDKVISNVDQLMEQRGEVTWGMPEDSTLHIILKSTDNDKLNELSDSAQLHRMVTQDIVSQIRKGEHVYIDRKSILLYLMKQELLTTNRCSLSIGEEEFLAEHLAMVISPSSPYLELINKQIYKMHQVGLIDKWLTDYLPTKDRCWSNTLSSESQTHTVNLDDMQGSFFLLFLGVTLGFILIIGELLFKKWKKTQEKQVIHPFVT

>HhalIR2

KNLAIIGRYNDEKVTLSRLHSFSEDKETITLASWSWKEEFRGTLFPKDEIAPAFKSKQLTVLLRHDPPFVIVKSYLNGSKYADGYLIDLWNCITERLDLSFEIKYFPTMEGGDFWNFANNGLRVEMERSEMDVALYASADPVDIYSNYTGVHTGVHLRLCTYEHKKRINFYQFIKTINSSCFAVLTFIGVLSAFLMYIAGNNLADSFLYFLAISFNQGPGEEPPTTSSVRIVIISFSISVLVFFYVSSASMASLSVNNQDLESTTIEEVVDHSYIRRSIIVEYTSAHVMMRYSDLATTFDKLRRVKMAKLPSVRDALTVTSKLKWFSFIERERVWPYWEEFKCCVFESDLRIVRPTHFMVRRNLSYTQVFRQESARLMENGVVSLLIKKWWPIVDASRKEFIPISLADVSIIFDLFAFGALVSLLIFFIERHVGILFH

>HhalIR68a

MFILLIFIILTVDCSLVSCTLSSSLIKRLWEYKKDDDFGWLLEDLIQRVLFDAKCLTIISDQFYNDMFTTRMFQKLSVIPIFVIFINENEDLLSPNYKTLSVILQARRKGCNGYIILIANADETMRFLRFGDRHRVIDTRARFFLLHDVRLFHKDYFYLWKKIVNVIFIRKFFEVNRYELMTVPFPSPIVHNWKPIRIDSWKSGKYQKNNELFIDKTSDLRGEIVHVAVFEYMPSVLKKVVEDDQSGNIMPIEYSGLEVLILRSLSDAMNFQILIYEPPNSKTEAWGKQQLNGSYTGLLGEMVSGRADFALGNFLYTPKNIKLFDLSIPYITQCFTFLTPESTTDNTWKTLILPFKKFMWLGVMLTLVVSSLVFYVLANFHKYYENGRISVRPVSIFVKKFKL

>HhalIR3

WTRVLLLLAVTGGSWGRKRGISDMAKRINEDNKHLNHSDAQIHRLFNYVMPDMRCYQVETDGTAFGQSFQEDLHQSNAVPTLVTGGSWSVVEGCKGFVIIATFFSSVIPIVQKMPRWAEHRILVILKGTGANTLLVPIMVDMRIFRDAEVAIVSSSDLTFGYRLTWTERYYVSFRSEPNATWKRLKEGPNDFLGREIWVQTSSCSMFSQIGPINAKGQPAWTGGAEMIMFQDIAERLKLRPRFNYTKFLHAGWFKEPLTSENKSDVAFCGILVSSRTLELKNIKVSQPLALLCLKLLVPRPQRVSDQWDEIFEPFSPGLWLLIATVTFFTTFLLQRFTTVTRRLVFTKRIKSMAGVTYIGWKIIFSFLRYKSLLLSRIRIVGGKFPSDRCYFGLIRRA

>HhalIR75f

MLDFNYEPEEEFLSRYGFSIHKILEDAYNFRMNISIYDDWGYFNEKLKIWDTGMFHGLSAGDIDLGTSISRVYGQRLDVSLYFPPYLKFRTCFIFKHPSRLGEFTALVKPLTLGSWLCILSGVVLSGLTLWFIKWFETVDIPSNENDLASSLLSSLGTFCQQGLSSDSLRLPVRVLYIFLLVASLVIYMFYGAAVVGFLLLPSPKTIDTVEKLIDSPIIPYAENLAYHKTHFQGNFSDKAAKAYEAIKETKTEKERWIDLTAGIQKVKQGRAALYAQDTNLYRAIENSFSNSDICVLAEIEIVLIWASTVIRKKSPYKELLYQGMILTHENGLLNRVMKTWQAQRPTCFAQNESPTVSFEA

>HhalIR4

NNDYLFPIDSTYLIEMMNYTFRDNYCVQLVADESIIANRIREAMFNVNFLRGVSDKRGRFQCINCVFIASTVEIFINILKTRPVDTDKFLSIIININDENVLYELSHKDVTNYLTNWHGVVIDLKAPHFLYRYIPLHKTFKTHNLSDIRNREIGINYGSFGGRKLRVGTFNCSVNSQIGPLDAKGRPSWFGGVEMIFLDAITQKLNFTYEIILPPDGEGVGGRLDKYGNLSKGLVGLVLDQSVDVAFCGIWQTNYINTRSLSISSPIQEVCITYLVPRPLPFNQMGLGLFTSFEGKVWMLIFISIITTAISSQLIAFMAVKHGFSNFQLISKYSKLYCIRYRVYYLF

>HhalIR75b.1

DGVTFTAATSVLYPNIFEGFSEENLNHPEADAYAKVGFAIERNVGQQFNFSFTIKIFFNSYGYLKNGSFTHVMGLMVKEQIDFTTGLMMRDERMDYIDFAGNTFATYSPLIFKQPSLSSVSNIFLLPFEAQVWLATGVLLFVSTIILFVEIIITSRLLFRTRYSFLEVFMGILEDAFLQGSTLQFESAAAKLTSLLFSIVSYFLFIAYSAKIVALLQLSTSSITSLSQLSNSHMAIAIQDVVYNRVYFQETKDPYVKEFYQKKIYPLGEKAYLPPKDGIMKIRSGFYAYKLETDWAYKLISETFNENEKCGLTEMNIFVLPMISPAFPKRSGLREHFSRSIIWQ

>HhalIR21a.1

TYWMFTIIITACYTGSIIAFITLPVYPEVMNTMHQLLHKNYRVTTMADEGWWTLLIGSDDDVASGLAGTAETVNNVLEGLSTVIKSSKEDKPVTFLGSSEHLKHILKSNYSASDVSKRQLFHISRQCFVPQMISMIMPHDSIYIDSFSRSLIKAIEAGFINKIQQDLEWNLYKSSARQTLLQGNLKLEALERQLTLEDTQGMFLLLGCGFAFAVCAFGFELGTWIKNRKGEKLTIVVKERILAASRRVSAAFLTPYKSHIRDHYPLYFRNVSRRKSSLFGSMIILEASDRQKPPTAVSEIHLMPPKVEKPIRLLSF

>HhalIR21a.2

AFKRILENGEVIWDGVEVRLLLLMKEILNFTMEFQDHIGKQSSDTLQILRQGNTDLVVGGFVMTKEIYGKTSMIYPHFMDCAAFISLTSIALPKYKAVMGPFLWDVWISITLCYILAIIPIAFSAWHTLGPLIQHPSEIENMFWYVFGTFTNCFTFRGQFSWTKSVKNSTKLFIGTYWMFTI

>HhalIR60f

LSTDLRLGLVWITAAGDKNLVFTFPSLIIPHPFMTEHICFFFKNPKEVATWKLIFVGFNDVVWIVLIATAFAFPCCLFLLARFQNYQHPFQKFSISIMSSYALLVSFPSSVDPRTIVFRLAFATWFFYTIHINLAYSAALKSLLTAGKTEPKMASF

>HhalIR5

EWYGIFSPYTTNLWLAIIVAYLSVSLLLPTLAYMDSLISVFGECRYLSFERSWILLAGMLLQGNWMRSTHSQGPCRHLIAWWTVFSLLVGSVFSSSLASYLTRAGYTWKPETIEDLLQTDYSWT

>HhalIR25a

MCRLLLLLLLEHALSQTLQTINIMFINEAKNQLAEMSFDVVLNYLKKNPKLGVKVEAAVRVSISGTDAKAILESICEAYNGTVSDGKPPHLVLDSTMNNVPSEAVKTFTDALALPTISSSFGQEGDLRQWKTLDNEKQKYLIQINPPADIIPEIVKSIVQLQNITNAGILYDDSFEMEYKYKSLLINMATRHIIVHVHNADSIENQLMRFRNLDIVNFFILGGLSTIKMALDTASKKQYFGKKFAWHVITQDKGQLSCSCSNATILYVKPEPEPGMKERLDGLRNSFNLVEEPEITSAFYFDFFLHSILAIKNLLDEDDWPKDFNYTLCDDYRADREIVRKEIDLMKNLRLVSEPYSYAPFLLERNGNSFPEFVMKLEKVTIVNSQSESAESMGTWKAGLISPIILKDATAMNNFSAVTVYRVVTVKQKPFVIEYEEDGRKKYKGYCIDLIDEIKTLVGFDYEIYVAPDNQFGNMDENGNWNGMIKELVDKRAEIALGSLAVMAERENVIDFTVPYYDLVGITILMKKQTTATSLFKFLTVLENDVWLCILAAYFFTSFLMWVFDRWSPYSYQNNREKYKDDEEKREFNLKECLWFCMTSLTPQGGGEAPKNLSGRLVAATWWLFGFIIIASYTANLAAFLTVSRLDQPIESLDDLAKQYKIQYAPLNGSVAMTYFQRMANIEKRFYEIWKDMSLNDSLSEVERAKLAVWDYPVSDKYTKMWQAMKEAKLPNTLEEALDRVRQSKSSSEGFAFLGDATDIRYQQLVNCEFQMVGDEFSRKPYAIAVQQGSPLKDQFNNAILQLLNKRKLEKLKEKWWSENPERQKCEKQDDQSDGISIHNIGGVFIVIFVGIGLACITLGFEYWWYKYKRPADGGGGPMVVKPTIGGGRNVEKLSVTGLADFGHHTTFRSRNTHNSNMRRGNLSHIPTSQW

>IR8a

MELPLLVLLLALRFAGSEVLKITFWIEPVQRAEFDTDIAMVLKELDALRLDVKVDDTTLTLTRSEDGLDMQRFCEILSTVGASAVIDLTYSHWEEGYNLVRSLGIGYVRLERIMRPFLDMFGDFMRQKRANNVAMVFMNARDAVEAMQQMLVGYPFRTLIMDASQTDPGQHFLERIRSLRPAPTYIALFARAAAMNGIFEKVQKADLFQRPLEWHFVFLDTRDRVFKYRRQAELCTRFTLNPRAICRSMPMPDLYCGSGFTMQRAMLLNVLRSLINAAQVSPGYPLAIYQDCNATASSSEVSDPLEKDDYNWLDMVHWSNFLAYAPPLPHIQDQFQSPVPGLTFAVNISAGYYSSEHEAKTDLAAWSSVGEMRLLNETISPARRFFRIGTAESIPWSYLRREEGTGELIRDRSGLPIWEGYCIDFIIRLSQKLNFEFEIVAPEVGHMGELNELGEWDGVVGDLVRGETDFAIAALKMYSEREEVIDFLPPYYEQTGISIAIRKPVRRTSLFKFMTVLRLEVWLSIVAALVGTAIMIWFMDKYSPYSSRNNRQAYPYACREFTLRESFWFALTSFTPQGGGEAPKAISGRMLVAAYWLFVVLMLATFTANLAAFLTVERMQTPVQSLEQLARQSRINYTVVKDSDTHQYFVNMKFAEDTLYRMWKELALNASKDFKKFRIWDYPIKEQYGHILLAINSSQPVADAKEGFANVDAHENADYAFIHDSAEIKYEITRNCNLTEVGEVFAEQPYAVAVQQGSHLGDELSYAILELQKDRFFEELKAKYWNQSNLPNCPLSEDQEGITLESLGGVFIATLFGLVLAMMTLGMEVLYYKKKQNALEITQVRPVNDSSGSGGNSSTAPPTATSTTKQAWHIPVLEAEEKPAKVSPPPSFETATFRGKKLPARITLGDGKFKPRHGLYARRNLGASDSHSGYME

>IR25a

MILMNPKTSKILWLLGFLSLLSSFSLEIAAQTTQNINVLFINEVDNEPAAKAVEVVLTYLKKNIRYGLSVQLDSIEANKSDAKVLLEAICNKYATSIEKKQTPHLILDTTKSGIASETVKSFTQALGLPTISASYGQQGDLRQWRDLDEAKQKYLLQVMPPADIIPEAIRSIVIHMNITNAAILYDDSFVMDHKYKSLLQNIQTRHVITAIAKDGKREREEQIEKLRNLDINNFFILGTLQSIRMVLESVKPAYFERNFAWHAITQNEGEISSQRDNATIMFMKPMAYTQYRDRLGLLRTTYNLNEEPQLSSAFYFDLALRSFLTIKEMLQSGAWPKDMEYLNCDDFQGGNTPQRNLDLRDYFTKITEPTSYGTFDLVTQSTQPFNGHSFMKFEMDINVLQIRGGSSVNSKSIGKWISGLNSELIVKDEEQMKNLTADTVYRIFTVVQAPFIMRDETAPKGYKGYCIDLINEIAAIVHFDYTIQEVEDGKFGNMDENGQWNGIVKKLMDKQADIGLGSMSVMAEREIVIDFTVPYYDLVGITIMMQRPSSPSSLFKFLTVLETNVWLCILAAYFFTSFLMWIFDRWSPYSYQNNREKYKDDEEKREFNLKECLWFCMTSLTPQGGGEAPKNLSGRLVAATWWLFGFIIIASYTANLAAFLTVSRLDTPVESLDDLAKQYKILYAPLNGSSAMTYFERMSNIEQMFYEIWKDLSLNDSLTAVERSKLAVWDYPVSDKYTKMWQAMQEAKLPATLDEAVARVRNSTAATGFAFLGDATDIRYLQLTNCDLQVVGEEFSRKPYAIAVQQGSHLKDQFNNAILTLLNKRQLEKLKEKWWKNDEALAKCDKPEDQSDGISIQNIGGVFIVIFVGIGMACITLVFEYWWYRYRKNPRIIDVAEANAERSNAADHPGKLVDGVILGHSGEKFEKSKAALRPRFNQYPATFKPRF

>DmelIR21a

MSYYWVALVLFTAQAFSIEGDRSASYQEKCISRRLINHYQLNKEIFGVGMCDGNNENEFRQKRRIVPTFQGNPRPRGELLASKFHVNSYNFEQTNSLVGLVNKIAQEYLNKCPPVIYYDSFVEKSDGLILENLFKTIPITFYHGEINADYEAKNKRFTSHIDCNCKSYILFLSDPLMTRKILGPQTESRVVLVSRSTQWRLRDFLSSELSSNIVNLLVIGESLMADPMRERPYVLYTHKLYADGLGSNTPVVLTSWIKGALSRPHINLFPSKFQFGFAGHRFQISAANQPPFIFRIRTLDSSGMGQLRWDGVEFRLLTMISKRLNFSIDITETPTRSNTRGVVDTIQEQIIERTVDIGMSGIYITQERLMDSAMSVGHSPDCAAFITLASKALPKYRAIMGPFQWPVWVALICVYLGGIFPIVFTDRLTLSHLMGNWGEVENMFWYVFGMFTNAFSFTGKYSWSNTRKNSTRLLIGAYWLFTIIITSCYTGSIIAFVTLPAFPDTVDSVLDLLGLFFRVGTLNNGGWETWFQNSTHIPTSRLYKKMEFVGSVDEGIGNVTQSFFWNYAFLGSKAQLEYLVQSNFSDENISRRSALHLSEECFALFQIGFLFPRESVYKIKIDSMILLAQQSGLIAKINNEVSWVMQRSSSGRLLQASSSNSLREIIQEERQLTTADTEGMFLLMALGYFLGATALVSEIVGGITNKCRQIIKRSRKSAASSWSSASSGSMLRTNAEQLSHDKRKANRREAAEVAQKMSFGMRELNLTRATLREIYGSYGAPETDHGQLDIVHTEFPNSSAKLNNIEDEESREALESLQRLDEFMDQMDNDGNPSSHTFRIDN

>DmelIR31a

MNLLISMFILILAAGEGEIIPSMEESVVTNFVKSLVKTKQAIVFSCLFKDFKEISLALMRINQFVSVVNLNQSYSLTSILTRENYARTSVMVNARCSGSSELLFEASENRYFNKTYQWFLWGVDLEVQSLFPLNLNYVGPNAQITYVNETADGYAYWDIHSKGRHLKSNLEINLIATLINDTLNIARDIFHLQSIDFRGQFNGLTLRGASVIDKEDIISNEQIESILSRPTKDAGVAAFIKYHYELLGLLRERFNFTVNFRNSRGWAGRLGNTTFRLGLLGIVMRNEADIAASGAFNRINRFAEFDTIHQSWKFETAFLYRYTSDLDTHGKSGNFLSPFSDRVWLFCLLTLGAFSIIWVLFEIIDYKILRIRVNSQKLEHLNQKSSVICIKTTCIERILQTFGACCQQGLDPNPVDRSVRFLVMTLFLFSLVMYNYYTSSVVGGLLSSSDQGPSTVDEITASPLKISFEDIGYYKVLFRESQNRSITRLIEKKLSSSRSLNELPIFSHIEDAVPYLKAGGFAFHCEVVDAYPVISEYFDANEICDLREVSGLMEVEILNWILHKNSQYTEIFKTAMCNAQEKGFVERILRRRQIKKPACQSLYTVYPVSLSGVLPGFVILICKSINKFS

>DmelIR40a

MHKFLALGLLPYLLGLLNSTRLTFIGNDESDTAIALTQIVRGLQQSSLAILALPSLALSDGVCQKERNVYLDDFLQRLHRSNYKSVVFSQTELFFQHIEENLQGANECISLILDEPNQLLNSLHDRHLGHRLSLFIFYWGARWPPSSRVIRFREPLRVVVVTRPRKKAFRIYYNQARPCSDSQLQLVNWYDGDNLGLQRIPLLPTALSVYANFKGRTFRVPVFHSPPWFWVTYCNNSFEEDEEFNSLDSIEKRKVRVTGGRDHRLLMLLSKHMNFRFKYIEAPGRTQGSMRSEDGKDSNDSFTGGIGLLQSGQQADFFLGDVGLSWERRKAIEFSFFTLADSGAFATHAPRRLNEALAIMRPFKQDIWPHLILTIIFSGPIFYGIIALPYIWRRRWANSDVEHLGELYIHMTYLKEITPRLLKLKPRTVLSAHQMPHQLFQKCIWFTLRLFLKQSCNELHNGYRAKFLTIVYWIAATYVLADVYSAQLTSQFARPAREPPINTLQRLQAAMIHDGYRLYVEKESSSLEMLENGTELFRQLYALMRQQVINDPQGFFIDSVEAGIKLIAEGGEDKAVLGGRETLFFNVQQYGSNNFQLSQKLYTRYSAVAVQIGCPFLGSLNNVLMQLFESGILDKMTAAEYAKQYQEVEATRIYKGSVQAKNSEAYSRTESYDSTVISPLNLRMLQGAFIALGVGSLAAAALNNTINVRSLNSRDKFICGGPVKIWYYLVLLLWYYFNRGLVGIYQLWHKTSIRNTGKGMPFLGE

>DmelIR64a

MHWWLLVFLPLSCQGLPEHELLELELDYGLAEPQRTSLLQSSLILQFSQDYKHIPRITYFTCQKPHLQTPNQIPNAAEHRDAFAAKNFQLIKSLYESELFVRIVLLDVLAQSPTSGRPNRPGNGPTGGFSQTPSQAQSNSEWLEGVLRMEALRQIAVVDLACGAVSRRFLELASAKMLYSEKFHWLLIEDFAWHGRTQTAEGSGKRDDGEMEEEEPPGQQIQATDDEDLPSIESFLGGMNLYMNTELTLAKRMSEAAHYTLFDVWNPGLNYGGHVNLTEIGSFTPTEGIQLHTWFRTTSTVRRRMDMQHARVRCMVVVTNKNMTGTLMYYLTHTMSGHIDTMNRFNFNLLMAVRDMFNWTFVLSRTTSWGYVKNGRFDGMIGALIRNETDIGGAPIFYWLERHKWIDVAGRSWSSRPCFIFRHPRSTQKDRIVFLQPFTNDVWILIVGCGVLTVFILWFLTTIEWKLVPHDGSALIKPKGGAPPRHHYQQQQQQEQVEAPVRPITAVSVVVSKEKVEEKQEEYEDSTPIDAGTLWQRCYQKLNKYIKDRKAKQKKAPERVGLFLESVLFFVGIICQQGLGFSTSFVSGRCIVITSLLFSFCIYQFYSASIVGTLLMEKPKTIKTLSDLVHSSLKVGMEDILYNRDYFLHTKDPVSMELYAKKITSVPTTKENEADEDEPVDPNPVSTDPAKSYRDIVHSHETGAHAKDNAASNWLDPETGLLRVKHERFAFHVDVAAAYKIIAETFSEQDICDLTEVSMFPPQKTVSIMQKNSPMRKVISYGLRRVTETGILTYHFNVWHSRKPPCVKKIETSDLHVDMDTVSSALLILLFSYAITLMILGTEILYSKWHNRIQLKWVGAT

>DmelIR75a

MQLVQLANFVLDNLVQSRIGFIVLFHCWQSDESLKFAQQFMKPIHPILVYHQFVQMRGVLNWSHLELSYMGHTQPTLAIYVDIKCDQTQDLLEEASREQIYNQHYHWLLVGNQSKLEFYDLFGLFNISIDADVSYVKEQIQDNNDSVAYAVHDVYNNGKIIGGQLNVTGSHEMSCDPFVCRRTRHLSSLQKRSKYGNREQLTDVVLRVATVVTQRPLTLSDDELIRFLSQENDTHIDSLARFGFHLTLILRDLLHCKMKFIFSDSWSKSDVVGGSVGAVVDQTADLTATPSLATEGRLKYLSAIIETGFFRSVCIFRTPHNAGLRGDVFLQPFSPLVWYLFGGVLSLIGVLLWITFYMECKRMQKRWRLDYLPSLLSTFLISFGAACIQSSSLIPRSAGGRLIYFALFLISFIMYNYYTSVVVSSLLSSPVKSKIKTMRQLAESSLTVGLEPLPFTKSYLNYSRLPEIHLFIKRKIESQTQNPELWLPAEQGVLRVRDNPGYVYVFETSSGYAYVERYFTAQEICDLNEVLFRPEQLFYTHLHRNSTYKELFRLRFLRILETGVYRKQRSYWVHMKLHCVAQNFVITVGMEYVAPLLLMLICADILVVVILLVELAWKRFFTRHLTFHP

>DmelIR75b

MNFSVLESHFKEAQIFVDADVTYVTHDPFSKNFLLYDVYNKGRQLGGELNITADREIFCNKTNCRVERYLSELYTRSALQHRKSFTGLTMRATAVVTALPLNVSIKEIFDFMNSKYRIQLDTYARLGYQARQPLRDMLDCKFKYIFRDRWSDGNATGGMIGDLILDKADLAIAPFIYSFDRALFLQPITKFSVFREICMFRNPRSVSAGLSATEFLQPFSGGVWLTFALLLLLAGCLLWVTFILERRKQWKPSLLTSCLLSFGAGCIQGAWLTPRSMGGRMAFFALMVTSYLMYNYYTSIVVSKLLGQPIKSNIRTLQQLADSNLDVGIEPTVYTRIYVETSEEPDVRDLYRKKVLGSKRSPDKIWIPTEAGVLSVRDQEGFVYITGVATGYEFVRKHFLAHQICELNEIPLRDASHTHTVLAKRSPYAELIKLSELRMLETGVHFKHERSWMETKLHCYQHNHTVAVGLEYAAPLFIILLGAIILCMGILGLEVIWHRHCTLH

>DmelIR75c

MTSWPLYRLIVFNLLEINLSNLMVFHCWSIKEAFPLVEMLNQNGIFSQYIDVQNPDNLANVHKEYLDSDLVSLNADVTYVSREDEERFILHDVYNKGSHLGGKLNITVDQTLQCNRSHCQVKEYLSELHLRPRLQHRMDLSSVTFRLAALVSVLPINSSEEELLEFLNSDRDSHMDSISRIGNRLIMHTQEILGFNVQDAFGGAIGMLTNESAELCTTPFVPSWNRLHYLHPMTEQAQFRAVCMFRTPHNAGIKAAVFLEPFMPSVWFAFAGLLIFAGVLLWMIFHLERHWMQRCLDFIPSLLSSCLISFGAACIQGSYLMPKSAGGRLAFIAVMLTSFLMYNYYTSIVVSTLLGSPVRSNIRTIQQLADSSLDVGFDTVPFTKTYLVSSPRPDIRSLYKQKVESKRDPNSVWLSPEEGVIRVRDQPGFVYTSEASFMYHFVEKHYLPREISDLNEIILRPESAVYGMVHLNSTYRQLLTQLQVRMLETGITSKQSRFFSKTKLHTFSNSFVIQVGMEYAAPLFISLLVAYFLALLILILEICWARYAKKKFSTIIPQNQ

>DmelIR75d

MKVQVAHWLPLIFFLLVSGTPRVAGSWRSEYSRQDPDPKTRWGNQLPDMLVAYYRHHGVHSLMLVVCHTDIADFRLWKLWQHFNLNNFYVQVSTESSLRDLQHVDALDEHKDAPPPKSFHANNSTHWETSFLLPALPYKMGILLLEFSSECALNLLRWSAASEHNYFTTNRFWLLLTEDPGDIDLLEDPEIFIPPDSELRVLHYENVGNFSCSLIDLYKVAAWKPLKRTLVGHNIRNSRHVIHALQHFGSAITYRQDLEGIVFNSAIVIAFPDLFTNIEDLSLRHIDTISKVNHRLMLELANRLNMSYNTYQTVNYGWRQPNGSFDGLMGRFQRYELDLAQLAIFMRLDRIALVDFVAETYRVRAGIMFRQPPLSAVANIFAMPFENDVWVSILMLLIITTVVLVLELFFSPHNHDMSYMDTLNFVWGAMCQQGFYVEVRNRSARIIVFTTFVAALFLFTSFSANIVALLQSPSDAIQSLSDLGQSPLEIGVQDTQYNKIYFTESTDPVTKNLYHKKIASKGENIYMRPLLGMEKMRTGLFAYQVELQAGYQIVSDTFSEPEKCGLMELEPFQLPMLAIPTRKNFPYKELIRRQLRWQREVSLVNREERKWIPQKPKCEGGVGGFVSIGITECRYALGIFGCGAAVSFVLFLFEFIFRHFKQVYRIIKGYREVQR

>DmelIR76a

MENLLVESYYFSTVLSFFAQQFFADSHATCIFWHPAFDFRLETVHPMPLIIMDWHRWANRSDQDVYDYKIKEDEFEGKGIPYNDWTLRLTVAIERSHCETFIAFQEQIPEFARYFYHASIYSIWRSLRNRFMFVYTKEFEDKKDSYLSGYIFQDQPNILVITSQYLNSSTFEIKTNRFVGPRNFNKNPEPVEFYILQRFDAKGTKATWETQSAMSSKMRNLKGREVVIGIFDYKPFMLLDYEKPPLYYDRFMNTTDVTIDGTDIQLMLIFCELYNCTIQVDTSEPYDWGDIYLNASGYGLVGMILDRRNDYGVGGMYLWYEAYEYMDMTHFLGRSGVTCLVPAPNRLISWTLLLRPFQFVLWMCVMLCLLLESLALGITRRWEHSSVAAGNSWISSLRFGCISTLKLFVNQSTNYVTSSYALRTVLVASYMIDIILTTVYSGGLAAILTLPTLEEAADSRQRLFDHKLIWTGTSQAWITTIDERSADPVLLGLMEHYRVYDANLISAFSHTEQMGFVVERLQFGHLGNTELIENDALKRLKLMVDDIYFAFTVAFVPRLWPHLNAYNDFILAWHSSGFDKFWEWKIAAEYMNAHRQNRIVASEKTNLDIGPVKLGIDNFIGLILLWCFGMICSLLTFLGELWRGQG

>DmelIR76b

MATGIELLVAAALCVACPPLNDSPPTNLIQMGENGTLSPVTELPMDVDASEAGFDADAPVETLETINRKKPKLREMLDWIGGKHLRIATLEDFPLSYTEVLENGTRVGHGVSFQIIDFLKKKFNFTYEVVVPQDNIIGSPSDFDRSLIEMVNSSTVDLAAAFIPSLSDQRSFVYYSTTTLDEGEWIMVMQRPRESASGSGLLAPFEFWVWILILVSLLAVGPIIYALIILRNRLTGDGQQTPYSLGHCAWFVYGALMKQGSTLSPIADSTRLLFATWWIFITILTSFYTANLTAFLTLSKFTLPYNTVNDILTKNKHFVSMRGGGVEYAIRTTNESLSMLNRMIQNNYAVFSDETNDTYNLQNYVEKNGYVFVRDRPAINIMLYRDYLYRKTVSFSDEKVHCPFAMAKEPFLKKKRTFAYPIGSNLSQLFDPELLHLVESGIVKHLSKRNLPSAEICPQDLGGTERQLRNGDLMMTYYIMLAGFATALAVFSTELMFRYVNSRQEANKWARHGIGRTPNGQSVAPSRWLRGWRRLNSGHGQLLGASTHGQNVTPPPPYQSIFNGGSHGDPLNRWRRPLANGNALGNGVLLGGDSEGGVRRLINGRDYMVFRNPNGQSQLVPVRSPSAALFQYSYTE

>DmelIR84a

MIKLQVKVISWPLIILTAFLRVLQIESINTNFLELAAFEDFLRSEHLSHVLVVRGDDADGDWKIECHQKLLANYRVQFYRPEMSANFEDLMFYGSPRTAVLVLNSEHVLVRRQVFGVASEAGYFNNSLAWFILGSGRESLPVEQLIDQLLSGYRMGIDADITVALRGPDNASMLFYDVYRISRQANTPLIIEKKGLWTHSGGYQKFGNFKNTWVIRRRNFLNVTLIGSTVLTEKPPGFGDMEYLADDKQLQQLDPMQRKTYQLFQLVERMFNLSLAISLTDKWGELLDNGSWSGVMGQVTSREADFAVCPIRFVLDRQPYVQYSAVLHTQNIHFLFRHPRRSHIKNIFFEPLSNQVWWCVLALVTGSTILLLFHVRLERMLSNMENRFSFVWFTMLETYLQQGPANEIFRLFSTRLLISLSCIFSFMLMQFYGAFIVGSLLSESARSIVNLQALYDSNLAIGMENISYNFPIFTNTSNQLVRDVYVKKICKSGEHNIMSLQQGAERIIQGRFAFHTAIDRMYRLLLELQMDEAEFCDLQEVMFNLPYDSGSVMPKGSPWREHLAHALLHFRATGLLQYNDKKWMVRRPDCSLFKTSQAEVDLEHFAPALFALALAMVASALVFLLELFLHWLPDFRRRLGTMST

>DmelIR92a

MLLQPLVMHLSQLLRIIVGQYFAEFPSILIVYNNSASTTPLQLEYLSALELVLRELSKPIRLQWINVAFLKDLNDLEDQVMGALNSSVTEGFITILSQTHHFIHARYYATRNANVRLKDKRYLFLCEDESPAELLCMDILQFYPHHLMVRPGTETAPTGPTGPHPDPRRGGGASVSTKNKDDGEGGAGNKTTSPYRDINFELWTQKFVGAVGNLDALLLDAFLPNETFANRVELYPNKLLNLQRRSLLVGSITYVPYTITNYVPAGQGDVDPIHPQWPNRSLTFDGAEANVMKTFCQVHNCHLRVEAYGADNWGGIYDNESSDGMLGDIYEQRVEMAIGCIYNWYDGITETSHTIARSSVTILGPAPAPLPSWRTNIMPFNNRAWLVLISTLVICGTFLYFMKYVSYRLRYSGTQVKFHHSRKLEKSMLDIFALFIQQPSAPLSFDRFAPRFFLATILCATITLENIYSGQLKSMLTFPFYSAPVDTIEKWAQSGWKWSAPSIIWVHTVQSSDLETEQILARNFEVHDYSYLSNVSFMPNYGFGIERLSSGSLSVGDYVSTEALENRIVLHDDLYFDYTRAVSIRGWILMPELNKHIRTCQETGLYFHWELEFIDKYMDKKKQEVLMDLANGHKVKGAPQALDVRNIAGALFVLAFGVAFAGCALVAELLIHRMDLSK

>DmelIR93a

MNPGEMRPSACLLLLAGLQLSILVPTEANDFSSFLSANASLAVVVDHEYMTVHGENILAHFEKILSDVIRENLRNGGINVKYFSWNAVRLKKDFLAAITVTDCENTWNFYKNTQETSILLIAITDSDCPRLPLNRALMTVECRINAVVFVDQTILEENALLVKSIVHESITNHITPISLILYEINDSLRGQQKRVALRQALSQFAPKKHEEMRQQFLVISAFHEDIIEIAETLNMFHVGNQWMIFVLDMVARDFDAGTVTINLDEGANIAFALNETDPNCQDSLNCTISEISLALVNAISKITVEEESIYGEISDEEWEAIRFTKQEKQAEILEYMKEFLKTNAKCSSCARWRVETAITWGKSQENRKFRSTPQRDAKNRNFEFINIGYWTPVLGFVCQELAFPHIEHHFRNITMDILTVHNPPWQILTKNSNGVIVEHKGIVMEIVKELSRALNFSYYLHEASAWKEEDSLSTSAGGNESDELVGSMTFRIPYRVVEMVQGNQFFIAAVAATVEDPDQKPFNYTQPISVQKYSFITRKPDEVSRIYLFTAPFTVETWFCLMGIILLTAPTLYAINRLAPLKEMRIVGLSTVKSCFWYIFGALLQQGGMYLPTADSGRLVVGFWWIVVIVLVTTYCGNLVAFLTFPKFQPGVDYLNQLEDHKDIVQYGLRNGTFFERYVQSTTREDFKHYLERAKIYGSAQEEDIEAVKRGERINIDWRINLQLIVQRHFEREKECHFALGRESFVDEQIAMIVPAQSAYLHLVNRHIKSMFRMGFIERWHQMNLPSAGKCNGKSAQRQVTNHKVNMDDMQGCFLVLLLGFTLALLIVCGEFWYRRFRASRKRRQFTN

>DmelIR7a

MFHHLWLLMGLRSLAMGALHPPQPEAMTPLVAAALEILAEQVSPSQSTLAVMDLTQDAEHRDERQEQLMTIILRSVGSEMALRTFQKPPAEVPASFVVFLVNSAQAFNTLGFHFTDIHSTREFNFLILLTHRMSSRAERLQVLRDISRTCVRFHTSNVILLTEKRDGVVLVYAYRLLNMDCDLSVNLELIDIYKNGLFRHGHEARSFNRVLSLSGCPLQVSWYPLPPFVSFIGNSSDPEERAQIWRLTGIDGELIKLLASIFDFRILLEEPCNKCLSPDIKDDCSGCFDQVIISNSSILIGAMSGSHQHRSHFSFTSSYHQSSLVFIMHMSSQFGAVAQLAVPFTVIVWLALVVSSLLLVLVLWMRNRLVCGRSDLASHALQVLTTLMGNPLEARSLPRSSRLRILYAGWLLLVLVLRVVYQGKLFDSFRLPYHKPLPTEISELIRSNYTLINQEYLDYYPRELTVLTRNGSKDRFDYIQGLGKEGKFTTTSLIATMEYYNMMHWSTSRLTHIKEHIFLYQMVIYLRRHSLLKFAFDRKIKQLLSAGIIGYFVREFDACQYRKPFEEDYEVTPIPLDSFCGLYYISLIWLSAAVVAFILELLSQRIVWLRRIFE

>DmelIR7b

MKYWLYILSCCSLVASTMESSSDWDLAEALAQVVANSEMGRFKTLYIYTHTNSQSTGGHLEELLDQVLMIVPNNLQARRLLLQQSMEYKPYVHAVLALVDGLPSLSAIYARIRATQDLSHTLIYMSMPTDAYGEEMQATLRFLWRLSVLNVGVVLRPPGDHILMVSYFPFSALHGCQVISANVVNRYQVGTKRWASQDYFPSKLGNFYGCLLTCATWEDMPYLVWRPDGSGSFVGIEGALLQFMAENLNFTVGLYWMNKEEVLATFDESGRIFDEIFGHHADFSLGGFHFKPSAGSEIPYSQSTYYFMSHIMLVTNLQSAYSAYEKLSFPFTPLLWRAIGLVLILACLLLMLLVRWRHHHELPRNPYYELLVLTMGGNLEDRWVPQRFPSRLVLLTWLFATLVLRSGYQSGMYQLLRQDTQRNPPQTISEVLAQHFTIQLAEVNEARILASLPELRPEQLVYLEGSELQSFPALAQQSGSSARVAILTPYEYFGYFRKVHPMSRRLHLVRERIYTQQLAFYVRRHSHLVGVLNKQIQHAHTHGFLEHWTRQYVSAVDEKDESVARIASTSYSTLDGIDGDPSLSESEEDQQVAPVRQNVLSMRELAALFWLILWANLGAVVVFVLELLLPRIKLRKILRKMKKSTRASATTTSTLSSPSTTKDIPFSCKDGFQDSWPKCSLLVS

>DmelIR7c

MLHSAVHNVSLVYALVWAIDNYYGMATSTPLAVVQFPTSRESRRLHNDLIDAALGRSSGTGRIQFLLEDDRVEMTETDTDPPPPSGLTGRPIAIWFLDSLRSYFRLEMYLNQLGSPYKRNGFFLVIYTGLEDQPMESLKIMFRRLLNMYVLNVNVFLQRDGTVHLYTYYPYGPHHCQSSLPVYYTAFQDLAAPANGFGLTKPLFPRKLTNMHGCEMVVATFEHRPYVIIEDDPKTPGGRSIHGIEGLIFRSLAERMNFTIKLVEQKDKNRGEILPDGNFTGILKMMVDGEVNLTFVCFMYSKARSDLMLPSTSYTSFPIVLVVPSGGSISPMGRLTRPFRYIIWSCILVSLIFGFVLICLLKITALPGLRNLVLGRRNRLPFMGMWASLLGGLALYNPQRNFARYILVMWLLQTLILRAAYTGQLYLLLQDVEMRSPIKSLSEVLAKDYEFRILPALRTIFKDSMPTTNFHAVLSLEESLYRLRDEDDPGITVALLQPTVNQFDFRSGPNKRHLTVLPDPLMTAPLTFYMRPHSYFKRRIDRLIMAMMSSGIVARYRKMYMDRIKRVSKRRNLEPKPLSIWRLSGIFVCCAGLYLVALIVFILEILTTNHRRLRRAFNVINRYAA

>DmelIR7d

MDIRCVVALLLGLCKVQAVVWPHQHLLEEQLASQISATLQKIFINGLAVYNFGVFISTSYEEMDRDRVILVHQVLNRNLYPPNFPVAVVLASKMNRKITAQVFTQLLFVQNAEQAIAIAEGVNRNGLCVIVLLTSQPERPIMTKIFTYFMQERYNINVVILVPRLHGVQAFNVRPYTPTSCSSLEPVEIDIKDGDLWDVFPRRLKNLHGCPLSVIVWDIPPYMRINWKSSDPMDGLDGLDGLLLRIVARKMNFTLKLIPNEPNGLIGGSSFMNGTFTGAYKMLRERRANITIGCAACTPERSTFLEATSPYSQMSYIIVLQARGGYSIYEVMLFPFEKYTWLLLSTILGLHWIVGSRWRMPSPILAGWMLWIFVIRASYEASVFNFIQNSPVKPSPRTLDQALSGGFRFITDHASYRMTLKIPSFQGKTLISAGQPVDVFDALLKAPWKTGAFTSRAFLADHLVRHRKHRNQLVILAEKIVDNMLCMYFPHGSYFAWEINKLLFNMRSFGIFQHHSQILAWDNLPTTTDTDTPGKRIHSSTESVATGFAESMSFVVAALNCLMGALCISIVVFGLELLSRRRHWTGLEWLFERV

>DmelIR7e

MNHINEFVARAVLHVVHHYILSVTPSLVLTLCCRSNHTCNFYNKMMSTLFREWGLAPLQIVNVLRGVPWHPVPGRRHFNVIFTDSFAAFEEIRMEYYSREYNYNEHYFIFLQARDRLLQGEMRLIFDYCWRYRLIHCSIQVQKSNGDILFYSYYPFGEHGCSDMEPQLINRYNGSMLVEPDLFPRKLRNFFGCPLRCALWDVPPFLTLDEDQEEVLRVNGGYEGRLLLALAEKMNFTIAVRKVHVNMRDEALEMLRRDEVDLTLGGIRQTVARGMVATSSHNYHQTREVFGVLASSYELSSFDILFYPYRLQIWMGILGVVALSALIQLIVGRMLRERMGSRFWLNLELVFVGMPLLECPRSHTARLYCVMLMMYTLIIRTIYQGLLYHLIRTHQLNRWPQTIESLVQKNFTVVLTPIVQEVLDEIPSVQHMRFRLLEANSELDPLYFLEANHQLRQHVTASALDIFIHFNRLSADKVHQRGEQGSGAHFEIVPEDIISMQLTMYLAKHSFLIDQLNEEIMWMRSVGLLSVWSRWELSESYLRNEQSFQVLGTMELYAIFLMVLVGLIVGLLVFILELVSMRSIYLRKLFT

>DmelIR7f

MQGEDANLYVARALRLVIENVLAQLSTTLVVTISTRHLGTAHWFEYMMNILMDSWRMVAVQLLRIRPDLVVNPVPGRKRVSLLMVDSYQGLLDTNITASNANFDDPDYYFIFLQARDHLIPKELQLILDHCLAHFWLHCNVMIQTAQVEVLVYTYYPYTADACQKAYPIPVNTFDGRKWKASQMFPDKLSQMHGCPLTVLTWHQPPFVELVWDPKHNRSRGSGFEIQLVEHLARRMNFSLELVNIALLRPNAYRLAEGSSEGPIEKLLQRNVNISMGYFRKTARRNQLLTTPMSYYSANLVAVLQLERYRIGSLALLVFPFELSVWMLLLLALLIHLGIHLPSARRGNEEDGGGGLQVVALLLGAALARLPRSWRHRFIAAHWLWASIPLRISYQSLLFHLIRLQLYNTPSFSLDQLLAEGFQGICTANTQRLLLEMPQLARDPDSIQSVDTPFDWDVLNVLTRNRNRKIFAVANQDVTLSFLHSSAHPNAFHVVKQPVNVEYAGMYMPKHSFLYEKMDDDIRRLDASGFIHAWRRASFASVHRKEQVHMTSRRYINHAKLSGIYMVMAGLYLLAGLLFAGEVLLRQRN

>DmelIR7g

MNVTSLLNFESMKYIGAQTQAASINHHVAQALRVFIEDFYQRIAPAFIVVLSCRRPSPMNFYRNIMQLLYESVDTMIVQLVLVELGRPRRIAGPRTHNLLLVDSLDALLDIEIHTYTAQSDTSEYYFIFLQQRDALIPHDMQGVFAYCWRHQLINCNVMTQSSGGQVLLHTYFPYAPGQCNDSQPTRINMFLGESWKHRDYFPSKLHNLNGCPLIVLARKVSPFLDLDEGQRELRGLEGRLLQELSRRMNFSIQFSGLQDQLKNRTTWTEKQLLQKLVQERIAHLAIGYVRKRIQYATNLTPVFPHYSNRVVGCLLLNAHNLTSLEIWSFPFQALTWICLVAGDRLALVLAVYAASLGLPIDPPERPSLQLLFASWLIFGLIVRSMYSALLFFILRYHLHQRLPGNLQDLTHGDYAAVMGRTTLQDLREVPSLQDLLGLKSVIVTSEREEEVLRTLDRCTLREGAGSHPLFFGLISQDALLHLTQRGHRAGAYHIIPQDVLEQQLAIYLQKHSHLASHLDHLVMSIRSVGLVHHWAGQMASERYFRSRFLYREKRIRQPDLWAVYILTAGLYLLSLVVFICELLASRRAGL

>DmelIR10a

MAVLGTVFLLFMLDLKTLNLTRLNGLLVEPTRDLPQLELWLRAGSDHQDAENPYVQWFLLRTEIPLSIVTYQENRYWMDDPFGRRNLVLVMSLDQLLTNRGAAAPIQKASTFFYILADQDKDLSADEQLRLEGSCRQLWTQHKVYNRFFLTRDGVWIYDPFKRRDSAFGRLVRYYGSETLDKLLFRDMAGYPLRIQMFRSVYTRPEFDKETGLLTRVTGVDFLVAQMLRERLNFTMLLQQPEKKYFGERSANGSYNGAIGSIIKDGLDICLTGFFVKDYLVQQYMDFTVAVYDDELCIYVPKASRIPQSILPIFAVGYDIWLGFVLTAFACALIWLTLRVINLKLRIVSLGNQHIVGQALGIMVDTWVVWVRLNLSHLPASYAERMFIGTLCLVSVIFGAIFESSLATVYIHPLYYKDINTMQELDESGLKVVYKYSSMADDLFFSETSPXWNRDLRADVIDEVARFRNKAGVSRYTSLILESSHFTLLRKIWVVPECPKYYTISYVMPRDSPWEDAVNALLLRFLNAGLIVKWIQDEKSWVDIKMRSNILEADAESELVRVLTIGDLQLAFYVVIGGNLLAFLGFLAEHFRWKLQKKGV

>DmelIR11a

MRFAILWLFSGCLLPGIQVGIWVVVRAQPTGRDVLLSRLGNQQNELNTRRLANASSYLTRNYIANRINTLVVREICVECPYELSERQRQLVDQILASLAPELSVLLHKGTAEETTWEYTLFVVNDHTAFTGQVFIFPDELLEREFFCIVVVSEIQSRQFVRQTVGSIVKSNLQMHFVNVVVVAQLEDGTVGTYSYKLFKANCTPGITVRQINHFDRITGKPQQSMPDLYPVRNGHLGDCPFNVGAAHMPPHLIYKRHKDPPPASNVSIPAEDLAGIDWDLLQLLAKALKFRIQLYMPQEPSQIFGEGNVSGCFRQLADGTVSIAIGGLSGSDKRRSLFSKSTVYHQSNFVMVVRRDRYLGRLGPLILPFRGKLWGVIIVILLLAVLSTCWLRSRLGLSHPIEDLLTVIVGNPIPDHRLPGKGFLRYLLASWMLLTLVLRCAYQARLFDVLRLSRHRPLPKDLSGLIKDNYTMVANGYHDFYPLELTCRQPLDFSARFERVQRAAPDERLTTIALISNLAYWNHKHPNISRLTFVRQPIYMYHLVIYFPRRFFLRPAIDRKIKQLLSAGVMAHIERRYMQYENKRKVASNDPVLLRRITKSIMNGAYRIHGLVIVLATGMFILELLAGRSNGRLRRWMEWVHQ

>DmelIR20a

MLASLNRSTGLSAELLDLYGLVVHFLLSGEHTTLVYFNPAGLDCSWGVLWQRNLTAHPQIVWQRNYSYPDLYYQFNAKLLVLACLPMDSRAAIQLEILANSLSHLRTVVRLLIEVAGPDQVTLARQYLSFCLRRSMLHVELYFRDYHHSLILYSFRAFPSFELVMRWISVGQGVKLFLHKLDDLRGHRLRVIPDLSPPNTFFYRDARGDNQVTGYLWDFLATFAGRLNAGLEVVRPSWRAGSASDSSYMLEYSAKGLIDVGLTTTLITKWNLWAIHQYTYPLLVSSWCTMLPVEKPLATPDLFGRIVCPTLAMTLLLIILVTWLVFRQLRCLTRLKNSRPARIVPHLLTLLLLTTCSAQLLSLLIFPPYHVRIASFEDLLRGDQKILGMRNEFYNFDGAFRARYAGVFYLIDDPNELYDLRNHFNTTWAYTMPYIKWLVIKTQQRHFSKPLFRWSKDLCFFDFMPTSVIVAPDSIYWESIKDFTFRIHQAGLMKHWIRKSFYDMIKAGKMSIKDYSDLETLKPLNIGDLEIVWRVCGAAIAVASAIFIMELLYFYINVFFNSL

>DmelIR41a

MFIDLSWSLVLSAIVGKYLNESTICIFWNDKFEFQLLHKSDYISFVGINIKSFDDNGGHYIIDTGLKKKELQNKHLFLDELVIKIIISIEVTHCETFVVFDKDIDRFVNAFNKASVYSIWRSLHNKFVFAHIANESPESRNHFFEDQPNILFVVRDHSSASSFDIKTNKFVGRKAENPSQMILVDRYLASEQRFQFGKSLFADKLNNLQGREVIIAGFDYPPYTVIKHNMSTNAQDMGVSGESDFKNVYIDGTETRIVLNFCEQFNCTIQIDSSAANDWGKVYPNMSGDGALGMLINRKADICIGAMYSWYEDYTYLDLSMYLVRSGITCLVPAPLRLTSWYLPLEPFKETLWAAILLCLCAEATGLVLAYKSEQALYVLPGYREGWWTCTSFGVCTTFKLFISQSGNSKAYSLTVRVLLFACFLNDLIITSIYGGGLASILTIPSMDEAADTVTRLRFHRLQWAANSEAWVSAIRASDEALVKDILYNFHIYSDDELLRLAQDQHMRIGFTVERLPFGHFAIGNYLGPQAIDQLVIMKDDIYFQYTVAFVPRLWPLLDKLNTLIYSWHSSGFDKYWEYRVVADNLNLKIQQQVQETMTGTKDIGPVPLGMSNFAGFIIVWILGSAIATLTFLLELSLTYILKQSNLK

>DmelIR47a

MRQIKLLVWLLVVGVVSSTEQLQFLKNFLEAVHKERSISTILLIQRKVHKNDFLHGLYPIFWPIICLDETKRVELVNNFNKDFLALVYMESEADTLLLSALAADLNHIRDARIMIWLQMSPSENFLDRIVFQASKQKFLNLVVIENTLKTRRFYPFPQPKVQVIDKPFEEKEIYPALWRNFMGKNAIAVPDLVPPRSFNSFDPKTGHRRESGSIYNVFKAFTQRYNITMLLKWPLIRNTTQEEIIGKSVRGEIDLPITGQLISFRHPNGSRSQPLLGMTALSIAVPCGPELPMFDRFFLFYGLATPITITGYYVLLNTIEIILGTLSDRIKRHPRRKKILNLVLNLRVFSCILSLPTPQGNRLRSVKGQLTMVMSITGLILSCIVAAQTSTILTMKPQYRHIKNFQELSDSNITVVCNHLNYLTIKQQMDPKFMAKFMQNIWIVNSIEQMKMIFDLNTSYAYQTFSYKKDPFTLLQMHTTRKAFCRTPGLDLVSGLAYTAVLEKNSIYALALQDYTLKAFSAGLVYYWAEESIRDLISTVGRTQFEKLPIVIGYQSLKLQDYNVCWKILLIGGALAFCVFIVEVVVGLINRRI

>DmelIR47b

MREAQIIIFLLTSAAAVTLKQYEFLXSFLKAGEQEQTITTLLMMQKHVHTKNLLQGLYPXPWPIIHFVETQRIKFIALLYMSSEKDIFLSSLAANLKFERLDKPFGKSNIFPVLWRNYMGXIALTLDHLVEPRSFYWTDPRTNIKRRTGYIYMLITNFAEQHNITLQLXSPPNEDMSQMVIIERTHKGPRSTHNWADDQLETFERXQDSLLPWHGSMAIVVPCGQEMSAYERFHAAHAFRAPIIFFGFHIFLSLIDFLLRTISDRIRCNPRRIQLLQTVLSLCVLRCILSTSLPNSNXLRSRLRDNSPXXXVLQAXSYSALWXLTGTAXQXHNRDFQSHKLHDYXTTDGSXHSIEVPGLLKARNXXIXLFHIFSSLGTKFDLRIGSAGSHTSGVEFRYYELLDRXSSLENNIVSQVFTILKLPYSRFRVLKLEDCRGCWQTLFVGFSIATFVFIVNVLMGFFRNINQKK

>DmelIR48a

MHLLITETYMIIGKTLHDILNELNERLIISTNIIFCKQFDNLIHFEAQTSRFVYSSLEAFNITSLWNHVGNDNKLFVIVGNVPPYELFAKLELSSPENCTQFILNNTVDMCADALVKNSKAFSVSRELRIAPANVIVPHGKPLLSYRYLAAPFNTKVWIALGTYVFLISGFLCLIHWLRSGKWDFSQNLLEVYSSLLFTVFHLKATNGIERYILFGVLFISGFVYSTSYLRLLKSMLIAETFEKQIQTFEELAESNIPLLINPYDRMIFQHHHIPKSLWTAVRTVSSETLLNHRSHGYVRLCPAILTASKIPSHTHRHLFSVCRFSHEQEVVPKGSSXXSLVPCIRKRNREXNHLGCLSGVSWPGISXFFHYGALGGEAFGSILLHDANYFPSPRLFRRLAELHYGSY

>DmelIR48b

MILQQSSNLLKLLLLLAISSVRTQGLNDIIIELNQRLLISNNFLYCNQSDKLNEYEIKYLQHMPPISLMIFTSIESMNFTQVEYNLGADNKLFLIMGNEEPPYDFLHALNLHFQFAEYIIVIDEPVDLKKSTKWLDFVNHLWQQGYVQLLIYTSYDEKLYHKIIFPETVIEETLVEQYISIRGSFNNLYGYPVRVAAYNNAPRSMLYVNRWGKHIFAGFYMRFLRAFIDARNGSFVPVLTPSNSPGNCTLNLVNETVDVCADALAANPAAFSLTHGFRIASANVLVTHAKPLHSYRYLTAPFQWSVWACLVIYVLLVVNFLSFIGWLRSGKWEFSKYLLEVFSSLLFSGFYLKEIRGRERYILFGVLFIAGFVYSTEYLGLLKSMLISEVFEKQIDTFEALVESNITLMVDPYDKILFAKYNMPEILSPIMELVSFETLLKHRNRFDQDYAYILFSDRMALYDYAQQFLKHPKLLRIPIDFSFLYTGIPMRKRWFLKHHLGRAWYWAFESGLTRKLALDADFEAVRVGYLSFLITEHVEAQPLNVDYFVMPAIALAIGYILALLSFVIEMTAWRIREFLGCRKATMTSTGCSEGGHVDVD

>DmelIR48c

MSLLRIILIIIFLRIVSSIPDTIISHLSAELQIKIQIYFGLGNDLYDFSRLDGNYQKIIISHNISEEFKTYHDEPVLIIIRLERDLNLNLATLDVLRSYLTDRQYNDILLIDNDEENLNSYVDIRKAYWNAGFSQVLIYNSQQRTWSIKPYPYLQIRPTSLKEYIENRNTRNLMGYPLRVLVTNDPPHCFVDKDELPGSPNRYKGSIVTMLKIFADQLNATFQANPFREFRRYSTADCVQMVSDDEIDACGSIFIRTYTYATSQPVRLNRVVIMAPFGNPIEKFYYFFRPFDLYVWIGTGIIVVYIAVMGSLLHRWHFKEWNVGQYLLLAVQTLLNRELSLPQSSSGSKFMLLLLLFAIGFILSNLYVALLSMMLTTKLYQRPIENLADLKAANVNILLQTHNIRPNSVYGSSEELRERFLLVEESQHLEKRNGLDPSYAYVDSEDRMDFYLYQQKFLRRRRMKKLSNPVGYTWAVQVIKQNWVLEKHYNDHVQRFFETGLQNKLVDDVHELAVKAGFLHFFPTQTQTIEPLRLEDIVMAAMVLGGGHALAVICFLVELFA

>DmelIR51a

MYNVLVLFLLLFTRAQMEPHRRGHNMTLLRSVLTVIRGRENWKNTPIFLGGHCNSDDLNNLMSWLQNTMEVTCHTVDTSTSAKNENALGHFNINADNSLGLLFCQSSHELIWFNMDKRLRRLRGIRLIVILSDKRSSSSKAIMSTFKRLWHFQFQXNFQGYVVSTPVENDIPRVFFVKDKKTGRKQIRGFGYRTFVEYLHRYNASLHVSNSQQEHAINSSVNMGRIINQIVDGQLEISLHPYVDVPENMGDNSYPLLIASNCLIVPVRNEISRYMYLLLPLNQSSWILLLGSVIYISGVLYYIQPGLLHRTWDQRIGLNILDSISRIINICSPSRIYNPSLRYFIVSVHLSILGFVVTNLYSIMLGSFFTTLVVGEQVDSMQQLIQXQQKVLVKYYEVSTFLRHVEPDLVDGVAQLLVGVNASEQVSALLGFNRSYAYPFTLERWEFFSLQQQYAFKPIFRFSSACLGSPIIGYPMKSDCHLQSSLNMFIMRIQAAGLLRHWVVSDFNDAMRAGYVRLLENFLGFHSLDVDSLRLRWAVLLCGWLLSTLIFLCER

>DmelIR51b

MCKVLTLLVVILLLALTNAAYNVTLLKSVLSLISTREPWINTPIFVGHNTQGGDLNDLIIWLHQTMGVTSLTMNLFLQPEHIRPLGHFKITRYNGIALFFCHDKHDIMWLTLDRNLRKLRRIRLIIILRNQRSGSQGAIKSIFNALWQYQFLNVLVLQRDQLYSYTPYPAMRFFKLDIHTEPLFPHAARNFHGYVVSTPAENDIPRVFHVHDPLTKSRKVLGYAYRTFVEYLDHYNASLRLTNPDENLDPTTSVNMNHIVQLIIDGQLEISLHPYVFTPPTATKSYPLLIYPNCLIVPMRNEIPRHMYLLRPFQLYSWYILLFAVFYITGILYCISPKLNKSSWPQRLGLNFLDAISKILFISPPITIYRPTWRHLIIFLQLSVLGFMSTSWYNIELDSFFTTIVVGEQVNSMDQLVHQQQRVLVKEYEINTFLRHVEPRLVEKVSRLLVPVNASEQVSALLSFNRSFAYPFTEERWQFFAMQQQYAFKPIFRFSSACLGSPHIGYPMRVDSHLETSLNHFILKIQDTGLLNHWVVSDFNDAMRAGYVRFVDNVLGYQSIDVDTLRLGWCVLGIGWILSALVFSCEYWHLYPWRFIA

>DmelIR52a

MALGWSVIILGFIGQLSAQILNYTQSRDLELLEGSLFRVLSRLNLEEEYNTLLIYGKECVFHSLLRKLEISAVTVPSGSTDYDWSFSTAILILSCGYDAENEENSYTLMKLQRTRRLIYLEDNSEPESVCMRYSLKEQHNIAMVKSDFDQSDTFYSCRLFQTPNYVEGHFFKDQPIYIENFQNMRGATIRTVADSLVPRTILYRDEKSGETKMMGYLGHMINTYAQKLNAKLHFIDTSKLGAKKPSVLDIMNWVNEDIVDIGTALASSLQFKNMDSVWYPYLLTGYCLMVPVPAKMPYNLVYSMIVDPLVLSIIFVMLCLFSVLIIYTQHLSWKNLTLANILLNDKSLRGLLGQSFPFPPNPSKHLKLIIFVLCFASVMITTMYEAYLQSYFTQPPSEPYIRSFRDIGNSSLKMAISRLEVNVLTSLNNSHFREISEDHLLIFDDLSEYLVLRDSFNTSFIFPVSVDRWNGYEEQQKLFAEPAFYLATNLCFNQFMLFSPPLRRYLPHRHLFEDHMMRQHEFGLVTFWKSQSFIEMVRLGLASMEDLSRKRNEEVSLLLDDISWILKLYLGAMFISSFCFILEILRCGERCKRLWRCRW

>DmelIR52b

MTWLVILLCFLGYMAAHIADISVQNQSLMDNELINLLLKLRNEEFYDTLLVYGKDCEFHSVIKNVDVAVVLVSDSMNFEWNFSSLTLILSCGPDIDNGGPNSTSIKLQRNRRLVLLKEDFQPSNICNIYTQKEQYNIALVRENFTKSKSIYTCRYFQDPNVDEVNLSGTKPIFIEQFQNMKGKAIRIVPDLLPPRVMLYQDANDGELKMIGYVANLITNFAQKVNATLQLDFLKPSTSITEISRMAKDDELDMGITLEASLNTSNLETSSYPYLLTSYCLMVQVPAKFPYNLVYALIVDPLVLGIIFVLFLLLSVLLIYSQKMSWQDLSVANILLNDKSLRGLLGQSFPFPLNASKKLRLIFTILCFASIMLTTMYEAYLQSFFTNPPSEPEICSFQDVGSYNRRIAMSALEVNGLIKTNNSHFREIRMDDLEIFDNMPECYELRDAFNLSYNYVVTGDRWRSYAEQQTLFKEPVFYFARDLCFSRLIFLSVPLRRHLPYRHLFDEHMMQQHEFGFVNYWMSHSFFDMVRLGLTSLKDLSRPLAYTPSLLMDDISWIMKIYLAAIVLCVFCFLLEIGVDKWKRWMKFRNLQILNTC

>DmelIR52c

MVWLIIILFCLGNSSSQILDVTNNSHLDFDYRLFGLLQRLQVEKSYDTLLVYGEDCAIPSLFERLQVPAVLVSSGSTNFDWNFSSLTLILSCNFQDEREENYRTLMKLQTSRRLILLKGHIKPESVCDFYSKKEQHNVAMVKENFYQLEVVYSCRLFQDQNYEKLNLFDGKSIYKDQFRNMHGAPIRTLSDKEPPRTIPYIDSKTGEEKFKGYVGMLISQFVKKVNATMQIREDLIKDDEEVSFVDITNFTSNDILDIGICEARTLEMSNYDAISYPYLMSSYCFMAPLPDSLPFSDVYMAIVAPSILIMFLIIFCICSVLIIYIQERSYRSLTIRSVLMNDICLRGFLAQPFPFPRQYNRKLKLIFMLVCFSSLISTTMYTAYLQAFLWGPPIEPRLTSFDDVKKSRYTMAINIYEREFLEALNVSLEDVEIYDYGKFSKLRSTFNTNYLFPVTALQWFTINEEQKLFKYKIFYYCDAFCLNQFDILSIPLRRHLPYRDIFEEHMLLQKEFGLTKYWIDQSYRDMIRANLTTFKDFSPLLENDYIEVHNLYWVFTMYFVGMGMGLCFFILEILRPLRYWRNCKIKCEYCYAFLKNFAK

>DmelIR52d

MVRIIIILLCLGYTKARILDATNTNHTDLEERLLSLLLRLQQEQFFNTLLIYGEDCAFSSLSRRLQVPTILVSSGSTSFEWNYSSLALILTCEFKAEREENYQTLKKLQMNRRLILLNGNIKPDSVCDFYSKKDQYNIAMVNNNFHQVGIIYACRLFQERNYEKVYLSEGNPIYVDQFRNMQGALLKSITFNLIPGSMAYRDPKTGQEKHIGYVANLLNNFVEKVNATLDMQVKLHKAGKKTSFYNITKWASEDLVDIGMSYAAYFEMTNFDTISYPYLMTSTCFMVPLPDMMPNSEIYMGIVDPPVLVVLIAIFCIFSVMLNYIKQRSWRSLSLVNVLLNDICLRGFLAQPFPFPRQSNRKLKLISMLVCFFSVITTTMYTSYLQSFMWGPPIDPKMCSFADLENSRYKLAIRRYDIEMLRPFNVSMDHVVVFDESSQLEYLRDSFDDNYMYPMSALSWSAFKEQQKLFAFPLFYYSEKLCLKPISFFSFPIRRHLPYRDLFEEHMLQQNEFGLSTYWIDRSFSDMVRLKLATMNDFSPPRLEDYIEVSDLSWVFGMYFTGLGISCCCFGLELLGLPSWTRRLRLTNWLRVRN

>DmelIR54a

MWTVITGIVLWAPVLVAGSAVDFIFRAAAEHSLSVIMIRIDYCPYNWAKDIFENQTIPVVVLSDSETFINIRMFSRPLHVACLPGHELQKDLALLENFTSSLMDFPSQKKIVYISNNFSDPTRMDYIFETCYHRRIWNIVGLLASDEHRYFYRYHLYPSFRTEYRSLESSTIFDKDFPNMHGHPLTVMPDQWLPRSVLYVDRRTGKQILAGSVGRFFHVLSWKLNATLQLSKKVTTGRFLNATALKELSESFSVDVPASLTIMERVEQLASTSYPMEVTHVCLMVPVARRIPIKDIYFILSSASNMFLAIVIVSSYGLALNLLRNMTHRDVRLVDFVLNDKALRGILGQSFNLPLSRSFSTRLIFLMLGIVGLNVSSIFGAGLDTLMAHPPRQFQARSFAGLRRTKIPLVTTEEDFPTWMKLRVPMLVVNVSEYNHLRNGRNTSNAYFASRLYWNLFSEQQKRFTRELFIYSTDDCLWSLALLSFQWPQNSLFTEPVSQLILEVNANGLYDFWVGMHYYDMTAAGLSGLEDPSLQLKEREHPTSLRIVDFQWMWQAYGTFMVIAILVFLLEVSWHRITSLFVSLVY

>DmelIR56a

MGSRFFIRNLILFGLLASSNMQIPFGELEKKFELDVDFLLGVTELVGHIQGLYSITVYADCIDIHPSIQQRIMDKFMVPVNTIGSNLSRPNYHKLDNSRIRIVLFTGLNDTILVNLNKTDVPYSDNFYMLAYASAIKNKCIELDFIEEVFTLLWKMSIQNAILLIRGEFMMEMWSYLYMGKIHKIKLTKPNSYLESLRKYNYRFSLEVINDPPAIFWYNSSEQADVTGGGNLSVSGPLGLIIINFLRHLNVTIDIVPIPGKQTSQYELFQQPDNLRAENGVNMVGSALLKYSPMVTQSRMCLLVSNRRMIPFSRFLDRLVSPGVHKLTFVSSIGIFVIKYFSHRPRSFVDAIFCTIRFFFAIPLPSIILNRLPVVDRFIEVFIIIFVQILLSSNISITTSALTTGFWEPPIINVETMRASGLHILTEDPTILQAFKENILPSSLADLVILVDEDTYFHHVTTLNNSYVYVVQAHNWQIFRLYQQQMTNEPFEIASEELCSKWRILGIPLNPKSPLRFMFKDYFYRILESGLREQWVHSGFKKFCEFNNLKKLPVDSVDSWQPLSIEFYSNVIRAYIIGLVIATLAFVAELLHNGYRRKNVKKT

>DmelIR56b

MLLDTDLASGVIRSPYSFDIPHAFIFNETQFVVPKFCGPYMEIVKHFAEVYHYQLFLDSLESLPKKSVVEQDIISGKYNLSLHGVIIRPEETSDFFNATQHSYPLELMTNCVMVPLAPELPKWMYMVWPLGKYIWTCLFLGTFYVALLLRYVHWREPGNATRSYTRNVLHAMALLMFSANMNMSVKLKHASIRVIIFYTLLYIFGFILTNYHLSHMTAFDMKPVFLRPIDTWSDLIHSRLRIVIHDSLLEELRWLPVEYQALLASPSRSYAYVVTQDAWLFFNRQQKVLIQPYFHLSKVCFGGLFNALPMASNASFADSLNKFILNVWQAGLWNYWEELAFRYAEQAGYAKVFLDTYPVEPLNLEFFTTAWIVLSAGIPISSLAFCLELFIHRRKQRRPQYERFECYDY

>DmelIR56c

MRSSFRICLFLLTTYHPSHGWNMQHLLNLLAPFGRMNVFQEIVWFVSPHQRLDQLDEFIMRIDEAFGKSATQTVVNNNTEMRMIYSSARRNHMSFVFTTGAEDPIMKVFSKVLLGRHFYVSMVIYVDKVGDMHPIYDLLTFAYNQQFFNSMVHFESMEGVNQLFGVSKFPVMSFENRTDFLKYMGKIWKQVQNARSDVGGFGFTTPLRQDLPHLFQSQGHYDGSTYRIIETFVRFINGSFKELIMPPDSLGGQVINMKDALQLIRERKMEFCAHAYALFMSDEELEKSYPLLVVQWCLMVPLYNSVSTYFYPLQPFDWNVWFFALGALLALVLLELMWLRMFGGWSGYRGAVLNSFCYIINVPIEGQLQQPCLLRFLLLATVFFHGFFLSAYYTSNLGSILTVNLFHAQINTMNDIVSAQLPVMIIDYEMEFLLNLNKELPQEFLELLRPVDSAVFSEHQTSFNSSFAYFVTEDHWEFLDEQQKHLKQRLFKLSSICFGSYHLAFPLQMDSSLWRDIEYFTFRIHSSGLLNFYARSSFGSALHAGLVQRMPDTQEYTSAGLQHLAIAFILLLVMSFLAGIVFVLETLSR

>DmelIR56d

MDNRAAELILRERNIFPTNGSDNITLLNNMFVLEMFYRITQLYHFKNFIFYISERLDLNNKDSQEFFHNFWTYFPMAPNLIITREHHLGIPMMQFISTPSLVMVFTTGKDDPIMELASHNQQGIHWLKTIFVLFPSLQSRDFETNPESLAQFTAEIKDVYDWVWRKQFINTFLITIKDNVFILDPYPTPSIVNKTGVWQAEEFFHKYAKNMKGYLVRTPILYDMPRVFKSDRPTNRYEKNFIHGTSGNLFLGFLEFVNATLMDTSANVTADYLNMTNLLDLVSQGVYETLIHSFTEITTKFVVSYSYPIGINDCCIMVPYRNQSPADQYMHEALQENVWVLISLFTLYITVAIYLCSPLRPRDLSAAFLQSICTLTYSVPTFIIRTPTLRMRYLYILLAIWGIVTSNLYISRMTSYFTTAPPVRQINTVQDVVEANLRIKMLAIEYERMAKSPLQYPESYLNQVDLVDKHMLDLHRDPFNTSFGYTVSSDRWRFLNLQQLHLRKPIFRLTEICEGPFYHVFPLHKDSHMRSVMTEYIMIAQQAGLMNHWERETFWEAVHLHRIHVHLFDDEPMALSLDFFSSLLRTWTLGLILAGLAFAAEMKWHEHVTFKRRPVIRITRKPRSFLRRFMKL

>DmelIR56e

ERXAFRNQWAFCFPRTXAIEVVLSAWSPXCPGQRSKPQPISXPHHXGSCWRKRKWKXKPRLLVVDKRTLVEHLNSLNDGYAYCIIAGHWQVGMM

>DmelIR60a

MWCNNPGLIIIIFLGQILNLCQGIVNLSNETANTVIFMLPEKDLGPDVWKAGVGCLDSFAQIFFFRNPKERFTRAYNLMLVHAFHLSSPADQIQEGFSKLINEAVTNPGPPDREELFQMRVASDYNITNGTEDKGELILADNYVIVVDSVDRLKELMKKKIVEMRSWNPGARFLVLFHNATCRNRPLGVASNIFKDLMEMFYVHRVALLYANSTMNYNLLVNDYYSNVNCRILNVQSVGQCHDGKLYPNNAVVKASMQDYVSGFSPRNCTFFACSSISAPFVEADCILGLEMRILGFMKNRLKFDVNQTCSLESRGEMDGPANWTGLLGKVQNNECDFVFGGYYPDNEVADHFWGSDTYLQDAHTWYIKMADRRPAWQALVGIFEAYTWIGFILILIISWLFWFTLVMILPEPKYYQQLSLTAINALAVTISIAVQERPICETTRLFFMALTLYGLNVVATYTSKMIATFQDPGYLHQLDELTEVVAAGIPFGGHEESRDWFENDDDMWIFNGYNISPEFIPQSKNLEAVKWGQRCILSNRMYTMQSPLADVIYAFPNNVFSSPVQMIMKAGFPFLFEMNSIIRLMRDVGIFQKIDADFRYNNTYLNRINKMRPQFPETAIVLTTEHLKGPFFILVVGSCWAALTFIGELIIHRWRTQLVSTSEQQDRRSDKRRRRRRRRKPEKDNRWQRQVQVAPVVRFTPVKRRKVFQGQTSQK

>DmelIR60b

MRRSLYLIIAIGLVDVHCVSLRYILNALENELQYRAILLVESASEIESCWEQKYIQGAVPILNFNANQSLYLKDALNTNILALVCLNENVESTMQALYENLEDMRDTPTILFVLSDSKVQDVFLECLRRKMLNVLAFKGLDRGFVYSFRAFPTFRVIERNVMDILQYFEQQLEDLGGHTLTTLPDNIIPRTVVYKSPDGSRQLAGYLYPFLRNYVSTINATLKVCWHLVPEDGMIQLGEVVRLSEIHDVDFPLGMHGIEHGSTSQNVPLEVSSWFLMLPMEPSLSRAQFFIMLGFEKVTPVLLLLTILLSTAHRIEMGLRPSWRCYVLGDRVLQGTLGQAFFLPRRLSVKLMLVYSLILLNGFTFSNYSITSLETWLVHPPSGHPIHSWEQMRTLNLKVLIVPSELDSMTKALGKQFTESNSDLFELSKSGNFQDKRLAMDQSYAYPVTCTLWPLLEHAQIRLPKPEFRRSREMVLIPLLIMAMPLPKNSMFHKSLNRYRALTHQSGLYEFWFKRSFNELVALRKIHYKVNGDHQIYRDFEWQDFSYVWLGFVGGTIASILVLLAEIGYHRWQLNQN

>DmelIR60c

MEMRLALFFTFACLAGAHDGSLRNMLKSLEDELGYRTILLLEGFVYSFKAFPTLRVVKRRVKDVRRYFEPQLEDLGGCVLKVVPDGIMPRTMVYQGEDGELQMGGYLSHFIRNYVSTINASLQIRWDLFPEDGDFDMDSLTGSNHVDFPLGLGSLSFQTLHKDVAMEISSWFLMLPMEPSLPRARFFIRFGISLYLIPLIILLAIVLSNAHRFEAGLTPSWRCCSMGNTVLRGVLAQAFVLPKGLSPKLMFVYWLLLVSGFFVSNYVIVYLTAWLIQPPTSDPVTDFDQMRRAKLKILMVPTDMDYLKSIRGAEYVDAHSDVFQTADSTDFQTQRMSMELHFAFSVTGTLWPLLRQAQVKLHRPIFRRSKEMVFLPFVIMGMTMPNNSIFLSSLKQYRLRTSEAGLYLLWFKKSFSELVAIHKISYKEDWVHDSYSDLKWEDFLFAWLGFLGGTTVSCLALLAEIGYHRWLWKRTHQ

>DmelIR60d

MRLAIYVAFLSSIGNRSGFLSSLLMSLGKELHYKTILLVGGSSTCWSLEPFETGVPILNLRGENNAYPQDTFNSQMLALACLQTESEDAVKLLYRSLKDMRDTPTLLFASSEEHIHDTLFLGCFRENMLNVLALTASSKEFIYSYQAFPTFRVIKRKLVEIHRYFEPQLKDLGGHIVSALPGNIMPRTMCYRNAEGERQLAGYLNTFIRNYVESINGTLRISWGLVPEDDMRHLTISRLSKIQHVDFPLGIIPLYNKTDKQHVYMEISSWFLMLPMETSVPRAHLFVKLGLERLLPIIVVVGAVLGNAHRIEVGLGPSWRCYYLADKVLRGALAQPIVLPRRLSPKLMLIYSLLLLSGFFLSNYYMASLTTWLVHPPASDRILEWDQLRYLHLKVLTIPEEFKYMSLILGTDFMTAYGSIFQLTNSTDFQRRRISMDPSYAYPVTTSLWPFLELSQVRLRRPLFRRSYDMVLQPFQVMSLPLPRNSIFHKSLLRYAALTRETGLYYYWFRRSYYELVALGKISYKEEEGNPYCDLKWNDFRIVWLAFLGGTIISCLALLLEVAHYRWHLGNSSL

>DmelIR60e

MVIKMISFLLVSVLLCLVGASDSESMQVQVLQDLNLALQTELNVFIDFECCATSEILHKLDSPRILLSSNSREARDLRIRGNFTESTLIIVSVMDSDLNPLVASLLPRLLDELHELHIVFLSNEEPGFPKQDLYTYCFKEGFVNVILMSGKGLYSYLPYPSIQPISLSNVSEYFDRARIIRNFQGFPVRILRSTLAPRDFEYSNEQGGLVRAGYLFTAVKELTYRYNATIESVPIPDLPEYDVYLAVAEMLHTKKIDIVCYFKDFSLEVAYTAPLSIIREYFMAPHARPISSYLYYSKPFGWTLWAVVISTVLYGTVMLHLAARGARVEIGKCLLYSLSHILYNCHQKIRVAGWRDVAIHGILTIGGFILTNVYLATLSSILTSGLYDEEYNTLEDLARAPYPSLHDEYYRSQMKAKTFLPERLRRNSLSLNATLLKAYRDGLNQSYIYILYEDRLELILMQQYLLKTPRFNMIRQAVGFTLESYCVSNSLPYLAMTSEFMRRLQEHGISIKMKADTFRELIHQGIYTLMRDDEPPAKAFDLDYYFFAFVLXTVGLISSLLVFFAELVSGHL

>DmelIR60f

MRFHLNIANSGLLGLHLCPTRSALPEQNPCFSKAGAVIXNLTLPWRRWRERCLLGALRPXTLPTPELQCXSKYLPXRKSQQENASSGLPGFCXGDXQTELHRGSRAIALPRSPYHDLYYVWIAYLGGTMIGIGMLAVEIACFKWDLLRRPPIXMY

>DmelIR62a

MYLQFLFALFLSRYQIVATENFDRAFELALFLDRIGRVHRLHAITIVNSLGSVDPSYLDDLHRGLMCNSSNHFYMLPQMTATDKDSSHVHFSSLQDEETIYLVFARDSKDAVIYLQAERARGRRYTRTMFLLRKQESQKDIKYFFELLWKLQFRSALVVVAARNFYQMDPYPTVRVIRMRRLSSYDPHHVFPPANRKNFRGYRMRLPVQQDVPNTFWYKNRRTKAWELAGLGGILINQLMMHLNVTMDLFRFEVNGSSLLNMAALTDLIVKGKVELSPHLYDTLQSNTSVDYSYPTQVAPRCFMIPLDNEISRSLYVFLPFSLTMWLCLLFVLLVVHFVYVRRLIPDGHFWAILGVPGAGQVRYGNRKPVRRFSTFLILFGIFILGQTYSTKLTSSLTVTLIRRPDNSLEELFLLPYRILVLPTDVYAIVDSLGHAEQFSTKFSCTDAENFSQKRISMHPEYIYPISTIRWRFFDMQQRFLRKKRFYFSKICHGSFPYQYQLRVDSHLKDALHRFLLHVQQAGLHDLWLDTCYRKAHRMGYLKDFSTLAELEEKLRLRPLALNLLVPAFSLFLCGMLGSGIAFLVEIRHSFGCRQKPPSINRNPGD

>DmelIR67a

MLPILVPVLLLFNETSWINPILTSIYKDRHHETVLLLQHSQHGNASGLERFPWPVFSFNEQMDFYVRGKYNSEMLVLIWQTGNSDWDLDLWQALDRSLLNMRKVRVLLLRKWEKIPTADVAATAEHLLFLHVAVIGQGNRIYRLQPYAPQSWLQVDPIESPIFIKIRNYFGRYIVTLPDQFPPRSIVYRNPKTDEIQMTGYVYKFLLEFIRIYNFTFRWQRPIVQGERMNLILLRNMTLNGTINLAISLCGFETPSXLGVFSDVYDMEEWYIMVPRAQEISIADVYVVMVSGNFLIVLIIFYFIFTILDTCFGPLLLKERVDWSNLMLNERMISGIMGQSFNMSARNTISSKVTNATLFLLGLVLSTLYAAHLKTLLTKRPTSQQISNFKQLRDSPVTVFFEEAERFYLKHAWDRPIRYIKDQLNFRETIEYNALRMGLNRSNAFSALTSEWMIVAKRQELFKQPIFTVQPELRVIQTSVLLSLVMQSNSIYEDHINDLIHRVQSAGIVEYWKHQTLREMITMGMISQKDPFPYVAFREFKVGDLFWIWLLWVSFLFMSFVIFLCELLVDCFISKTLIRNKRPH

>DmelIR67b

MELLYLNTLQSLSLLEGNRLVQTVQELNNIYQTELNVFLEFGNGADILESAQGTFVPTLWIKNPQNQKVMKGNFTSCTLTILYLEDEHLDRGLYYLANWLWEYHHLEVLIFFNGGSYDKLIQIFSRCFNEGFVNVLVMLPGSDELYTFMPYQDLKILNLKSIKEFYSLSRKKMDLNGYNITSGLVIAGAPRWFSFRDRQNRLILTGYMLRMIVDFTNHFNGSVRLMNVLTVNDGLELLANRTIDFFPFLIRPLKSFSMSNILYLENCGLIVPTSRPLPNWVYLLRPYAFDTWIAWLIMLIYCSLALRILSKGQISISAAFLKVLRLVMYLSGSRDMGTRPTTRRLFLFVILTTSGFILTNLYVAQLSSNSAAGLYEKQINTWEDLDKSDSIWPLIDVDIKTMEKLIPDRTKLLKKIVPTLEADVDTYRRNLNTSCIHSGFFDRIDFALYQQKFLRFPIFRKFPHLLYQQPLQISAAFGRPYLQLFNWFVRKIFESGIYLKMKDDAYRHGIQSGLLNLAFRDRHLEVKSNDVEYYYLIAGLWFGGLTLATVCFLLELLIGYAKIKVTISCKMNIM

>DmelIR67c

MFCWLIFLNIILLSDRSESWSAREVIHQFNHDQQLQLNIYLDCNDVELQIGQEVSNLFVNSTADKMKILGRFSSHSLIIACFKDSTRNRTLNGVKELLWGLQYLPILFVVDSNMDFYFQQALRHGFIHVLALNFMNGSLYTYKPYPKVEVHQIKDMQKFYKLTKLRNLQGQAVRTTVETMTPRCFRYRNRHGQLVYAGYMYRMVKEFISTYNGTEEHVFGNVDTVPYKEGLAALKNGEIDMMPRIIHALEWYYFYRSHILYNIKTYIMVPWAEPLPKSLYFIQPFRGTVWITIMVSFVYASIVIWWIRYRQQGNSSLTQSFMDVLQLLFQLPLSKIWHFNMGTHQVVSFIVLFVFGFMLTNLYTAQLSSYLTTGLFKSQINTFDDLFREKRTLLVESFDAEVLHNMTKEKIIQKEFESIILITSIEEVFKHRKSLNTSYAYEAYEDRIAFELSQQRYLRVPIFKILKEVYDQRPVFVALRHGLPYVELFNNYLRRIFESGIWIKLQEDSFLEGIASGEISFRKSKSREIKIFDKDFYFFAYILLGMGWCVSTIALFLELWSFKYSVTNVLHEG

>DmelIR68a

MRCLWILIVAFISLAMATSIPIPIANPAPLSGYEMQLKILLQKILWVANVKRCFAVITDDLHYPIYDRIFFESVGRRVIPFFVMRTNESDDLQRPSRQVELFVKAIKSSDCELNVITILNGWQVQRFLGYIYDNRSLNMQKKFVLLHDLRLFESDMIHLWSVFIDAIFLKRQLDNKYTISTIAFPGILSGVLVMKNIANWELGKGLNGRILFADKTSNLFGTSLPVAISEHVPMVLWANATKSFQGVEVEIMNALGKALNFKPVYYKPNQTENMDWTELDGGASVAYGSGNPDGYAQNGTHIDSMLVDEVAAHSARFAIGDLHLFQVYLKLVELSAPHNFECLTFLTPESSTDNSWQTFILPFSAGMWVGVLLSLFVVGTVFYAISFLNAIINGNVSSEFFRCLRPNRNVPMDPKIYRRISFRIAISRYRSSKGDRMPRDLFDGYTNCILLTYSMLLYVALPRMPRNWPLRVLTGWYWIYCILLVATYRASFTAILANPAARVTIDTLEDLLRSHIPPSTGATENRQFFLEANDEVARKVGEKMEVFGYSDDLTSRIAKGQCAYYDNEFYLRYLRVADESGSALHIMKECVLYMPVVLAMEKNSALKPRVDASIQHLAEGGLIAKWLKDAIEHLPAEALAQQEALMNIQKFWSSFVALLIGYVISMLTLLAERWHFKHIVMKHPMYDVYNPSLYYNFKRIYPQH

>DmelIR68b

MKFLVGLLLQWYLPGIYALAEIACRIAVEQNVQVTYLYRCASCPASFDADYSALELDLYRCVGSRLPVITRNMEAHELEPFRRTDSLSIFQIPAAEKGDSLVRRILDMLNPHQRRKHMHKYLFVWPNAGRHQLLRLFRGSWAKKLLYGLAITGRENGTFDFDPFAWGGLQVIQRLDGEVPYARKVKDLRGYPLRFSMFTDPLMAMPRSPVETAGYQAVDGVAARVVGEMLNASVTYVFPEDNESYGRCLPNGNYTGVVSDIVGGHTHFAPNSRFVLDCIWPAVEVLYPYTRRNLHLVVPASAIQPEYLIFVRVFRRTVWYLLLVTLLVVVLVFWVMQRLQRRIPRRGVIQFQATWYEILEMFGKTHVGEPAGRLSSFSSMRTFLMGWILFSYVLSTIYFAKLESGFVRPSYEEQVDRVDDLVHLDVHIYAVTTMYDAVRSALTEHQYGLLENRSRQLPLGIATSYYQPVVRRRDRRAAFIMRDFHARDFLAITYDSQAERPAYHIAREYLRSMICTYILPRGSPFLHRLESLYSGFLEHGFFEHWRQMDLITRVGASPDAEEFLEDLGDQTDTDSGSNELAIRNKKVVLTLDILQGAFYLWSVGIGISCLGFAVEHAHWFWRRQTLRNAVEARTS

>DmelIR85a

MSIQWLKHILLLAILVNLAGTRENHIPLDLKKSSIVMVKMSQILCKARIKVLFVYFENQTSHEHTGQILKEVTKCDISNQNTPLEAVKDDGILMYMVMITTNISQPLELSLIRKKSAAKHRSHVFLLVRDADTVSDAWMRASFRQFWKIWLLNIVILYWRDGRLNAYRYNPFMDNYLIPVDNKPNEVPTLEQLFPKTIPNMQRKPLRMCIYKDDVRAIFWRQGTILGTDGLLAAYVAERLNATMMITRPHSYNNHNLSSDICFLEVAKEYVDVAMNIRFLVPDTFRKQAESTVSHTRDDLCVIVPKAKTAPTFWNIFRSFGSLVWALILVSVLVANVFCYILKSEVGRVPMQLFAGALTMPMTQIPPNHSIRLFLIFWLYFGLLICSAFKGNLTSMMVFQPYLPDINQLGALARSHYHIIIRPRHVKHIQHFLTLGHKHESRIREQMLEVSDTQMYEMMRNNDIRFAYLEKYHIARFQVNSRVHMHLGRPLFHLMNSCLVPFHAVYIVPYGSPYLGFLDSLIRSSHEFGFERYWDRIMNSAFIKSGVKVVNRRRGSGNDEPVVLKLQHFHAVFALWLVGIGMACIVLAWEHLTHNYNLAVTKRRD

>DmelIR87a

MSTPEQRFWLAALLFLLSQHSEVRGFGINLMKVQTEDKGQEACILALLRKYFDSGDGLSGSVLCINRNYQLPNIEEQLLRGVNNYENYPWSLLITNSREGPSPAKFLMNEKPQCYFLIVDNLEDEDLDEVFEHWKGMVNWNPLAQFVVYLASLEETDEEMNDLMVELLLTFINKKIFNVNVIGQSEENQFYYGKTVFPYHPDNNCGNRVISVELLDACDYPSEETDSEDENDEDEGDGAQEEDDGPQEEGDGEQEEEDGPQEQEDGDQAKGDEGQENDDGGLENKVENEFRIGASDDDELENDLSSNSSEPEAIIEEFFRAKFEDKFPRDLSGCPLTASFRPWEPYIFRNSEEQPVDDYYYGLQGDEDDYNDTSPNYGESDDESYADPGEDGDGAIPDTETQSGGKLKLSGIEYEMVQTIAERLHVSIEMQGENSNLYHLFQQLIDGEIEMIVGGIDEDPSISQFVSSSIPYHQDELTWCVARAKRRHGFFNFVATFNADAGFLIGIFVVTCSLVVWLAQRVSGFQLRNLNGYFPTCLRVLGILLNQAIPAQDFPITLRQLFALSFLMGFFFSNTYQSFLISTLTTPRSSYQIHTLQEIYSNKMTVMGTSEHVRHLNKDGEIFKYIREKFQMCYNLVDCLNDAAQNEHIAVAVSRQHSFYNPRIQRDRLYCFDRRESLYVYLVTMLLPKKYHLLHQINPVIQHIIESGHMQKWARDLDMRRMIHEEITRVREDPFKALTFDQFRGAIAFSGGLLLVASCVFAFELCYVKYVYRTEKRERKTKKITKKVHNIKIQHD

>DmelIR94a

MALPKQLKFINIFLVLLIIYGSSDGTENQHEIFLNRLLQAVHNERSVETLFLLHHSNLANCSLQDWNPPRIPTIRSNELTVFNVEKTFNHNALALVCLMKNSYREILNTLAKSFDCMRQERIILMIHRKSDSKFIEDITHEVKNLQFLHLIVLIVQEKYNGQVFASTLRLQSFPEPHFKRIRNVFAIQRIFYRPINFHGKVLNAIPNDIPILFVALNEMFTEYARRYNSTLRIQNRTIKEDIEITEDNYDIDMKIQLHNSQNFLHHMNIAMDIGSNSLIILVPCATELRGLDIFKELGVRTLTWLALLFYIIFVLVEMLFVFISNRFNGRNFTMRYTNPLINLRAVRAILGQTSPISNRYSLSIQHFFVFMSLFGTLFGGFFDCKLRSFLTKRPYYSQIENFSELRKSGVTVVVDHTTRQFIEQEINANFFRDEVPNVRTTTIQELINHVYSYDRKFAFVANSIPWRTFREEMKSINQKILCDSKNLTILENVPLTFSIRRNAIFSHHLRNFIINAADSGMITCWFKMAGKVIRKHIKTTLRESEQQPSHLPLSFDHFKWLWAVLCIAYVMSFMVFVMEILWSKYQRRTRSVSIV

>DmelIR94b

MSLIFNLLFILILSQAVSQETEFLQLKYLNNIVRSMIKLHKMETLVIVKHHLDNNCSLQNWNAHGMGIIRTNDQGKLIMKDTFNSRTLAIICIGQNSHITLLRNVFETFGKVQQKKIILWTQMELKEKFFQEISKKSRDLKLLNLLVLKAVTKDKLLIYRLNPFPSPHFKRIENIWTPNDTLFMDTKFNFHGMTAVVKHDYNWTIQMGNIRKFPISRIEDKEVIEFALKYNLTLQFFNDVERFDIELRKRIILKSNSTQPIDSGIPMVFSSLLIVVPCGNYLSIQDVIKVSGIEKWIFYIILVYVIFVLIEITFLGVTILISRQSRHQMIPNTLVNLCAFRAILGLPFPETRRTSLSLRQLFLAIALFGMIFSIFINCKLSSMLTNPCPRPQVNNFEELKTSGLTVVMDHDAENFIEKEIGVDFFNQYMPRKVTLTFTERAKLLFSLKGNHAFTLFSESFAIIESYQRSKGLRAHCTSEDLIVAERVPRIYILENNSILDRPLRRFIRQMQESGITNHWLKNIPSSLEKNLMQITIPYDRERVHPLSIEHLTWLWCILILGYSISMIVFFVEMSLKRRKKNLENRAPNICIC

>DmelIR94c

MSKVFKLLVLPLIYLSLTKGSKNPQLKFLRELINVIEEGREIRTIMVIKHSRDEYCHLDQWNPRGSPILRTNEMGSIRISGYFNDQAVILACMGENSDYGLLKSLANAMDNMRQERIILWSEREPTKMLMDYISQQADRYNFAQIIIVTMNEDVDAVPSLHQLNPYPTPRFRQITNISNIRRTSFFGCGLSFQGKTAILKESVVSNIRFKVWSPSGPIPLSELKDYEIVQFAVKYNLSLKLYDQNESKSDHFDIQLGPLFITKDFPTQMAFVSPNTACSLIVIVPCSPKWRFMDVLHKLGVLKLIGCLLIAYAVFVLIETLILWLTHRISGREVRLTSLNQLLNPRAFRGILGLPFPEFRRSSISLRQLFLVISVFGLVYSNFVSCTLSALLTKPAQNPQVRNFKELRDSGLITIMDKYTHSFIEKHIDPEFFDHVLPHYLILQKKEALRMIWNFNDSYSYVMYTTTWKSLNTVQKSFDERVFCESESLTIAWNLPRMYVLGNNSVLKWMLSRYITYMPQTGIPDSWTEQLPKVLKLLYNVTSPRRIKEGAVPLSIQHLSWIWHLLFIGESIATLVFIVEILLQKSNQHTSNMRERSSEDDDFV

>DmelIR94d

MGQLHLLLVALVLLSPGGDSFYHSLIHHLNRELKIEYVLLLGNFDTTWLDILWQLPVSVLQIKEHSRETYSLLENPSHNVLTIAFVNDSPEDILEILYRNLRMLNTQPVLLVIRKSTIRVNSLLEWCWHHQLLKVVAIAQDFMESLIVYSYNPFPVLQFIERRLDNSTVIFEKRLENLHGYEVPIALGGSSPRLIVYRDLEGKLIFSGPVGNFMKSFEQRYNCRLVQPYPFDESAISPARDLIASVQNGSVQIALGAIYPQVPYTGYSYPIELMSWCLMMPVPEEVPHSQLYSMVFSPMAFGITIVAMVLISLTLSMALRLHGYRVSFSEYFLHDSCLRGVLSQSFYEVLRAPALIKAMYLVICLLGLLITSWYNSYFSTFVTSAPRFPQLTSYESIRHSNIKIVIWKPEYEMLLFFSENMEKYSSIFQLQEDYKEFLHLRDSFDTRYGYMMPMEKWSLMKEQQRVFSSPLFSLQDDLCVFHTVPIVFPMVKNSIFKEPFDRLILDVTATGLLSRWRDMSFTEMIKAGQLGLEDRGHPKEFRAMKVGDLIQIWRFVGWMLGLATIVFLLELICFWRHKMWQNMKYMFCRNKNI

>DmelIR94e

MDCPKWILSGLCLISLVSGATVIELLGTLKLELDFEYVLLMKNRNFSLSDQVWNGTSLTKDVMDEVQVPVLQFNENVSYFLHNSISRRLVTLGFMSDANLDEHRGLLTALVANLRHMTTSRVIFLVQSKASTDFLYELFRNCWRKKLLNVIVIFQDFETTSTFYSYSNFPILQIEERIYETSLQTLPIFPDRLRNLHGYEMPVILGGTAPRMIAYRNKKGNVVYDGTVGHFMTAFQQKYNVKFVQPLQAKNPLDFAPSMQTVGAVRNETVEISISLTFPTIPPFGFSYPYEQMNWCVMLPVEADVPPFEYYTRVFELAAFLLTLGTLVLISCLLASALSLHGYATNISEFLLHDSCLRGVLGQSFVEVFRAPTLVRGIYLEICVLGILITAWYNSYFSSYVTSAPKQPPFRTYDDILASKLKVVAWKPEYAELVGRLLEFRKYETMFLVEPDFNRYLALRDTLDTRYGYMITTNRWVLINEQQKVFSRPLFQKRDDFCFFNNIPFGFPLHENSVFMEPVQKLIMELAETGLYYHWITTGFSELIDAGEMHFVDLSPHREFRAMQIQDLQYVWYGYAFMVVLSSLVWLLENLAYTVKSKTIFPTHFMQRNKK

>DmelIR94f

MSGMWQQVLLAETSNWFRSDVLQRFWTHLRVEIRFRTMLNYRLESCDCWFDNVLGSDNSTALLWNDQTYPHYLRRRQDTDILVVSCLRFHQYQEVLLALSLMLDQMRSMPVVLQLCGDEDSMQELNSARLLLKHSQDLKMPNVVLLSSTFFTSATLYSYEMFPEFNVQKLVYQAYLTLFPYKLGNLKGHPIRTVPDNSEPLTIVRKTLNGSIAIDGLVWQFMIEFAKHINATLQLPIEPHPEKSIKLVQILDLVRNQTVDIAASLRPYSLNVQRSSTHIYGSPMMVGNWCMMLPTERVIGSHEALTRLMKSPWTWLILLLFYSVHRFLAQKTRLRSSLIHLIKLLINLSLICFLQAQLSAYFIGPQKVNHISNMQQVEESGLKIRGMRGEFMEYPIDMRSRYASSFLLHDLFFDLAQYRNSLNTSYGYTVTSVKWELYKEAQRHFRRPLFRYSEEICVQKLSLFSLIQQSNCIYCYRSRIFILRMHEAGLIRLWYRRSYYVMVTAGRFPIGDLSTVHRAQPIRWTEWQNVVLLHGVGLLFSVVVFVIELTVHYANVCLNNL

>DmelIR94g

MSTAVNSVHSKLVSLISRGQELTSIFFYAPAKEKCHLEDTISSATWGLPLVIWRTDRTVILNGFIGEGLLVLACLPGFHWRALLGSLARSLKYLRQARILIELMQDRDEFLVSEVLQFCLSQDMINVNAIFDDFPETENLSSFEAYPSFEVVNQTFTPDTQVSDLYPNKMLNLRGGVIRTMPDYSEPNTILYQDKEGNKEILGYLWDLLEAYAHKHNAQLQVVNKYADDRPLNFIELLDAAQSGIIDVGASIQPMSMGSLSRMHEMSYPVNQASWCTMLPVERQLHVSELLTRVIPYPTLALLLLLWIFYEVLRGRWRRHSRLQSIGWLVLATLVSSNYVGKLLNLFTDPPSLPPVNSLAALMESPVRIISIRSEYSAIEFTQRTKYSAAFHLALHASILIGLRNAFNTSYGYTITSEKWKIYEEQQKRSSKPVFRYSKDLCFYEMIPFGLVIPENSPHRAPLHSYTLLLRQAGLHDFWVNRGFSYMVKAGKINFTAVGERYEAKTLTITDLRNVFIIYVSVLLISLILFTCELFVSWVN

YWLGF

>DmelIR94h

MLSNISFSSAPELVDLYGLVLKFLVSSETTLFYFNPTGQKCSWETLPRTILSNHPQIIWFREETYPGLYKRHSSNLFVMACLSSTSYDGQLQLLAESLTRYRSVRVLIEVQDKEGSFLASQILLLCQQHSMLNVVLYFSRWTRTLNVFSYLAFPYFKLLKQRLSGSLRPKIFINQLKDLQGYKIRVQPDLSPPNSFSYRDRHGECQVGGFLWRIVENFSKSLKGDTQVLYPTWAKAKVSAAEYMIQFTRNGSSDIGVTTTMITFKHEERYRDYSYPMYDISWCTMLPVEKPLSVEILFSHVLSPGSALLLILAFILFFLIVPQLIKCLGITFRGRLIGMASRIFALVMLCSSSAQLLSLLMSPPLHTRIKSFDDLLTSGLKIFGIRSELYFLDGGFRAKYASAFHLTENPNELYDNRNYFNTSWAYTITSVKWNVIEAQQRHFAHPVFRYSTDLCFSSETPWGLLIAPESFYREPLQHFTLKINQAGLITQWMTQSFHEMVRAGRMTIKDYSRTNLMKPLRIQDLRKCWVIFAVGLGTSTVVFTIELLLIYTNVFLNSL

>DmelIR100a

MATTLQLIMLALVGGTLGQANNTDHKQVLTSIVKQLEGGLELHLRTSEDGGNDLVQFLMQEKSSIIISAKQEEVPSRAKIMRHHFFIFDGVHQMQEIRTSLFNTDGFYILALENNTIEDDVLLMEFAADVWLQHGHSRIYYVQLSKKSVLLFNPFLQRLVVVQDSKTYSRIYKDLEGYHLRIYIFDSVYSSVIGDGENKVLSVTGADAKLAKTVARQLNFTADFVWPDDEFFGGRLANGEYSGGVGRAHRGEVDIIFAGFFIKDYLTTHIQFSAAVYMDELCLYVKKAQRIPQSILPLFAVHMDVWLCFLLVGLLGALVWLILRAVNLILGIEGVPDGSRATRISYFGAARRIFVDTWVIWVRVNVGRFPPFHSERIFVASLCLVSVIFGALLESSLATVYIRPLYYRDVNTLRELDESGQPIYIKHPAFKDDLFYGHNSEVYRRLDAKMMLVAEGEERLIEMVSKRGGFAGVTRSASLQLSDIRYVMTKKVHKIPECPKNYHIAYVLPRPSPYLEEVNRIVLRLVAGGIVGLWTGEAKERAKWSIQRFPEYLAELDVGRWKVLTLSDVQLAFYALTIGCLLSAIVCMAEILLGRQRRLHSPK

>ClivIR1

MPGVLGLIWQGEVDIFIGDLTITYERSLAVEFTFFTLSDSEIFLTHAPGKLNEALALVRPFRWEVWPVIIVTVALSGILLFVLQKVRLGWHSVKLSELCDSVWIITTIFLRQSLVTKVHGDRARLTVILLYLVATYVIGDMYSANLTSLLTRPAREKPISTLEQLNDAMTNKNFKLLVARRSSSHGVLENGTGLYQIVWNKMKNQNQYLLNTVEEGIEQVKHFKTVALLGGRETFFYNAKRYGL

>ClivIR6

MSELFQSNIEEVSRVCVITNPRHYSYHMYYNQASADGQSELTRVNWWTRRKGFYTNLLLPKSSELYKDMKGRMFVIPVLHKPPWHFVSFENETVHVEGGRDEKLLSILAEKLNFRYVYFDPPDRSEGSSIINNTMPGVLGLIWQGEVDIFIGDLTITYERSLAVEFTFFTLSDSEIFLTHAPGKLNEALALVRPFRWEVWPVIIVTVALS

GILLFVLQKVRLGWHSVKLSELCDSVWIITTIFLRQSLVTKVHGDRARLTVILLYLVATYVIGDMYSANLTSLLTRPAREKPISTLEQLNDAMTNKNFKLLVARRSSSHGVLENGTGLYQIVWNKMKNQNQYLLNTVEEGIEQVKHFKTVALLGGRETFFYNAKRYGVRHFHLSEKLFTRYSAIALRIGCPFIDQFNKQILNMFECGILS

KITEDEYQKLRKKQLSKTGETPISPAESFIGIGEGGSQSENDETLRAMSMKTLQGAFFVLIVGCCFGALVLIFEVVYTINIRKR

>ClivIR5

MNNINRERRQILATLFENVRLENLATYCESASDSPFHFTARCLWFVAGTRRACLVDINAGYKWISDTFEEAEKCSLSQIKLFKTEMLAMPVAKRSGYREIIAQKLCWQQENGLYDKIAATWIPPKPRCVGDSGGFTSVGTTDFLPAILALIYGCSIATFILILELLYKHRTHFRSMISGPGKDSDVSTNKRDEMKRSV

>ClivIR4

MEYANRQDTLSERRVAVRVMKSWSDEVDRVLYSKLGIFVDLSCLKNAHFLKEADQSRFRLNHTWLVWDPNQQYMAMNTPQSPYSEVVVASKDTLHQITKFVDELPPELHEVGTFSNNITTLTSLTIKPNLNNKVFKVSYYVMQEIYDPANLTNQMLDPDFQPGFDPGNRFGYATMLLLGDMYNFSMEFFQEYDWGEHLPNGTWTGVIGSI

QSGRTDMSISPLFPKAERLDISYAPPVIHNYEFVIAFQQHRKLGTYKAQTMELTPTVWALVGLFALAGGVFFTFTFMQGSFIRALPAGMIQVVGSLASQGLCIDPESASSRIAGLSLMLIGLLLSNYYNAATMTALLSEAPPEIKSFDEFLKTDIPINMVDAAYSTSKLGRIHVFPQEAWPKTGLTEGSPPPKIMTLEDGVKSMKKGDAF

FAESYLIAKGVNEAFTDDDKCSLTIFPTMATVNRAFRFLKKDGHLLEMFSRGIIRSMEVGIISKVRDDWIAKPPSCFRNVPFYQVQLEAVTVAFVVLLGGMILSLLIYFCELIPCLKTANMKQPSTLTTSVD

>ClivIR3

MPLAFLGWILMILDLFGTSFGKLDRIRIDDDYFNRKMVYLAREVMNEYMADRRACIIVVSDEGLLDDLKDFRNASFIRVLFNGTKTKCDPNMHNHLIQAFYHKCPRYVVQISKPTCFFPAWFLARNGSTYEKHNPRVLFLPIKPHATVYGEEVLAMNQTNISHDILIAETSDSKEWPITLYTNNFWQYVGEPGRIGRLYLDEWSWETGFKRNVDLYPDKVRDLRGKVLRLSAFPYLPYGNNEPMDGSEARILLEFCVVYNCTVVDVDDGHLWGSIYPNNGTGVGEAGTIYMELSDFGVGANYLWLEFWPYLEFSNAYLYGALTVMVPRPELLSGLLTPFLPFPLSLWLILVFTVFMSAVGLHVVTDATRRFAPHFREEIYHNHKFITVTDSLIRSIGMFVLQQPQRLVTGSPVRHLFTAFELIYLVITSAYAAELYDYLTIPRTTKPIDTVEDLADSNLIWMTDHEVWVFGILHAEDPKIKKAASNFRALPTPELIKLGETGSIGLGIERMAGGHYTELPYITDKFVSISRVMRDNYYWSPLVVNMQKGSPYANRLNDIIGRMESGGLYYYWEADCVRKYLNYTKQLDMIWSTRPIKYPPKVLNVADLEGAFILYFIGTAMAVGLFFLELYFKRVLKLERSFLDPTPKWFDEYLEEKNKILK

>ClivIR2

MLSPALDGVEIRVMDAFCVAANCQLSPVTDDDLWGELYENGTSIGIVGNVLQDNADVGLGAVYLWYYEHIEFVFPYMPSKVTVLLPKPSPLPEWKVPIAPFDWVLWLSLLGSIVLAAIVLFLMNKCLLKYSRSHIPPREFESVSGIILRAVGMAVLQSPENIFTNGSTLRIALTTFQVFFLLYTTIYSSALSSVLTIPRYYPPIDNMRDLYLSGLPWAADHIAWVTTLKEADDPIIKGLLGNFAVFDQETLTVLSKKGGHGFTIELTNGGHVSEAAFLAADMINNLHTMKQPMYHTYSTMIIRKGSPYADELRKIVHRCIDTGLVQMWESMMLTSYGSSSIQTAFQLSKKSRSNEHTGLVKLKLLHTQGAFFILILGTVTGTIIFFMECFFHPRRGGFNGYRQDDVGGLGILVGLGYYA

>AlinIR25a

MPSFTARLPGTTSVAKVFTVFMFYVSLLQKVHSQSATSINVMFVTEDRNDIARLAFDVVSDYVKRNSKLGIEMEVFRVTESGSDAKFLLENLCETFNASAKAGKPPHIILDTSVVGVTSEAVKTFSRALGIPTLSASYGQEGDLRQWRALEVEIAKYLLQINPPADIIPEVVRSIVILQNISSAGIVFDDSFVMDHKYKSLLLNVPARHIMGRVRNIQEIRNQLTRFKELDIVNFFILGSLSTIRNVLNEANGMKFFDRKYAWHAITQDKGQLKCDCSNATILHIKPEPDPGSKERLDNLRTSYNLVEEPEITSVFYFDFFLRGLLAAKALIEKAPWPKDYNKTSCDNYDENHDFIRKDLDLRSSLRDVKEAYSYAPFLISTNGKSFMEFNMKIEKVVIVNSIAESAEAIGTWKAGLSNQIQTKDIASMRNFSAVTVYRVVTVKQKPFVIETFENGKPKYSGYCIDLLEDIRSFVHFEYDIYVAPDNAYGNMDPSGNWNGMIKELIEKRAEIGLGALSVMAERENVVDFTVPYYDLVGITIMMKKQTTQTSLFKFLTVLENEVWLCILASYFFTSLLMWVFDRWSPYSYQNNREKYKNDEEKREFNLKECLWFCMTSLTPQGGGEAPKNLSGRLVAATWWLFGFIIIASYTANLAAFLTVSRLDTPIESLDDLAKQYKIRYAPINGSEAMTYFQRMADIEERFYEIWKDMSLNDSLSEVERAKLAVWDYPVSDKYTKMWQAMKEAGLPATLEEALDRVRKSQTTSEGFAFLGDATDIKYLVLSSCDFQIVGDEFSRKPYAIAVQQGSPLKDQFNNAILQLLNKRKLEKLKEKWWTENEDRMQCEKQEEQSDGISIHNIGGVFIVIFVGIGLACITLGLEYWWYKYKKPASPKQVGPMAQIISTNATNKQLSVTGLMDYNTREPRARYPIRRTAVNATQDYSRPTFPAQQERLSHW

>AlinIR41a.4

MVNVSLVGSTQRFRWILQLSDPAKFMENWEKARVDSLIRFKPRILFLPWNESNYATTLFEAPELNYIDDAVAVELEDADTNRNKQLKLVTNNFFVDIGSKNETTEIYLGVWPLNNSIEIFPNKIGDLQGKELRIATLQYLPYSQVSPELDGVELRILKSFCKQSNCSLVPVTDDFLWGELFENGTSNGIVGNVLQDKADLGVGAVYLWYYDHIEFAYPYMPSRVTVLLPKPSPMPEWRVPLAPFDFALWVALIVSIATVAFVLFYMNHYLQRFSSHHVSPNEFQSWSGVFLRAVGMAVGQSPQNAFSAGSTLRIVFTTFEILFLLYGTVYSSALASVVTVPAYYPPIDNMRQLYASGLPWTADHIAWVKNLMDADEPFIKDLLSKFEVHDQEMLSQLAKKGGYGFTIELTNGGHVSEASFLSADMINNLHVMKEALYYTYSTTIARKGSPYVDELNKLLHKCFDTGLLQLWESDMISKHGSSIIQTAFKLSKSAKASNEHHELVKLKLKHTQGAFILLLLGNTIGTLVFLFEFYCKSMKQVVKPTKMN

>AlinIR41a.2

MLSSSSESLPKNCCYILTIIVSLCAADFRTKNEEDVFNAKMVYLARQVAQDYMSDRLRCIVVVSDEGLLEDFTGYNDTTVLRVLFNGSRDECDPTMHKYILQAFYHKCTRYIVQISKPTCFFPAWFLARNGSTYEKHNPRVLFLPVKPHATVYGEEVLAMNQTNISHDILIAETSPQTPIGAIAMPKEIDPNRPVTLYTNNFWQYVGEPGRIGRIYLDEWSWELGFKNGVDLYPDKVRDLRGKVLRLSAFPYLPYGNNEPMDGSEARILLEFCVVYNCTVVDVDDGHLWGEIYPENGTGVGEAGTIYMELSDFGVGANYLWLEFWPYLEFSNCYLYGALTVMVPRPELLSGLLTPFLPFPLSLWLVIVMCVVVSAVGLHWVTEATIKFAPHFLDEIYKNHKFITYTDSMIRSIGMLVLQQPQRLVTGSPVRHLFTAFEFTYLVITSAYAAELYDFLTIPRTTKPINSVFDLAESNLIWMTDHEVWVFGILHAEDPSIRKAASNFRALPTPELIKLGESNHPYGLGIERMAGGHYTELPYITDKFIEKSRVMRDNYYVSPLVVNMQKGSPYANRLNDIIGRMENGGLYYAWEADCVRKYLNYTKQLDMQWSTRPIKYPPKVLNVADLEGAFLLYFIGTALAVGLFFLEIYFKKGLKLKNSFLNPTPKWFDEYLLGQSGSHD

>AlinIR41a.1

MPDMVKNLQNKTLKIMTFDYDPYTHFEPLDGTEIKLIQEFCKKHNCSLVAVDDGHFWGDIFENGTSDGLAGMVYDGRADFGAAAVYLWLPYFYFVDYSTSYLYSASTLLVPKPHPVSGWKTPFMPFDVFTWIGYGLSVIMAAVFMYVITYFTVKYTRFTEAVRKRRMFLDKLDCLFRALGLAVLQQPSTPLVPNTPIRHLFTSFEFLFLITSSIYAAELASYLTVPRYEKPIDTLIELADSGMIWIGEHESWTYSLRGMTDPEIVTIVNNYRIFSHEKLRQLAPTGEYGLIVERLPGGQYTEQEHVTDEVVAQSHMMAENLFGSPPVIAVRKGSPYRKYLNKVISNVLCGGFYLYWEREMSRKYLHSRRQLALREAEHPHYKDIPKRLTISHIQGGLYLYSLGITISLFVFILELFHFKAKGPKNRPKDK

>AlinIR75d

MGALLPYSLITQYFINIHVSSIIVVSCCTTSQTAQLLRHLSQRGITASWAVDNLSPLEVRRSGIVLDLSCNQSKEILHDMSSRKMFGLEMEWLLMSEGSAPEEAELPDLYILPGSSVTLSVTSPSSISFYDTYRITRRLPYKFTLLGAVARDEDVLPQWKRPSRVNYEQNLLTTVSVIHSLDIRKLTDPDVAEEDRWPAIHFPVVVNVAYQLNFKFDLRLESVHGWKFPNGSFEGMIGVMEREEVDFGASGVIMREDRRKHVDYTVDYFEFKTGIIFKQPSLSSVSNIYLLPFSRHVWAACGGLLLFVLIILCIAVSSGDAQTFTPPATFLDMVNIVLGFVCQQGSYLAPVTISGRIVVFVSSLAALFLYTSYSANIVALLQSTSSVLKTLKDLTNSHLGLKVQINEYHLGYFLEAVDEDVITLYNKKVKNQPETFVNGTRGVEFMRTGDFAFCVEFDLAYKQISKTFQEEEKCGLGEMHLFFVPRLSIPVIKRSGHREHFTQTIIWQWESGMLDRISRIWLARRPRCESTGGGYLRVGLKDFNPALKVILVGIIISIWFFLCELITDRGFKAYYRKIKHNQEKIMGDDGHLIADLCFPSWKMLFKNKRF

>AlinIR41a.3

MNNPLNLCTDGLIISMWLFVIGSSTTSQANVLFDNKHTILLGSLEKSVITQYFHKDKCIVLIVEDDTWTTNKELEHYYSLLSLILVSSIDICRDPYLVESIVSAIDSGCYSYILRVAEPKCVLKSWGESQLSYNVHTFQRVSPKMFATISNHQKDGSKIVDEFYSLPEAELSSNIVVAVFGNESLEWPVTIYTNNFYEPMSSADREPKVFLDRWNELNGFELQADLYADKILDLQGKELKVAVVDLLPYAELRRFIGQEAQILKHFCAHRNCTIKGITDEWFWGEIFENGSGNGLLGMVFDGRADFGIAGVYGWASVFRHTEFSASYLHSGVTLLVPKPVKVGGWLIPIFPFSSEMWLAYILSVIVAGISMHIITMATIKYTRFAEVVLKRGMFLTAVDTAFRALGLSVLQQPSTPLVPHTPIRHLFTAFEILYLVFCTVYAAELASYLTAPQYSKPIDSLEDLADSGMIWLGEHYGWVYSLLDVDTPSILKIVDHFKVVTFEEMDKLAGTGMYGLIVEQLAGGHFSERQYLSEKIIAQSHIMAEYLYDSPVITIMRKCSPYREHYNELIGRLLENGLLLFWEAEAARVYMSSWLQTALKSAIKVNMEDEARALTLSDCLGMFLLLAFGLFTSSVVFIVELWIIRQKNNTKD

>AlinIR41a.5

MDLHSDWCNLMCFLIITQHIGSNWCVDHDEMTTGLAHEIAEHYFSEYNECITVVADLGALQKFSPPNNSFIRVSFDHTNDSCDPSVKESLRNSFREKCVRYVVQIAKPNCFFPSWFESMNLGYERHNPTVVFLPSINADDQNYGDDLLSKNETNISADILVAEISDAEEWAVKIYTNNFYQLTHEPERISKIFLDEWHPSKGFRFKADLLPNKLKDLKLKTLRVFTVQYLPYSSYDPFDGSEVRMVKEFCNVVNCTAVGLTNDGNWGTFDEETNTGTGQMGAIHSGEADIGVGGNVVWLEFFPHLDFSDAILGGASAIIAPRPKVLGGWFTPFLPFPLDLWVVVWAVVIASAILLYLFTTLTIRTIPHLAEKHRRNEKFVTFTDSLLRAIGMLISQQPSNLVTGSPVRHLFTSMEVIFLVITTCYCAQLYDFLTVPRTTKPIDTVKDVVENNLTWLAPSDLWIYALKHSDDPVVAKFVQLFQAYPPEEIIELSEKGEVGVTVEKLAGGHYVEDSYISYKFISQSRVSTGPDIYGMTPITLFMQKGSPYTEALNRFHGHMQNGGLHFAWEAGTARDHLNYTIQEGIAQSSRKQVYPPKVLRLADLEGAFLIHFIGVAVSIIVFFIESRVGKKKKSL

>AlinIR21a

MKLEVLFVTTLVHLTHSVKITKLLNSMAFADLETISCMAPDGIPYMVPLINSIAKRYLKDHATVILYDDYFYYHPRLKNMIDILISNYAYPLRHGLVNTTMAKPTVPAGILEARENEQMAFIVFTKESEIGAEAIREFTGHNTMTLLIAQTSVYHVKLFLQTKLAADITNLLVFVDPMIKIDHFVQKTARVLKECDILIFSHKVITDSLGISMPVIVTAWRRNHLTRQVQLFPPKYKRGLGGLHLVASASEIPPFVFRKHGHDSGAGYTITKWDGIEVHLLYMLSQMLNFTVEYKEPEFNEEEDVAQTVIKDLHTKKTTLAIGGVYLTPERIGGLTFSFPHTQDCASFISLASTALPKYRAIMGPFLWDVWLALTAVYLLAMFPIAFSVWHSIKPLLNDIREVENMFWYVFGTFTNCFTFTGKNSWSKADKTATKFFIGTYWIFTIIITACYTGSIVAFITLPTYPETIDSSKQLLEEDYKISLLGSGGWEGLFNDTEDPVASKLYESVERVPNLYSGLRNVTRNVHSWRQSAFLGSRRLLEYTVKTNFTPDEDSKRLMFHLSDECFVPLFVSIVMDKRTNYLEEFNNALERIIQSGFMTKIVREVEWQEYRSASGKLLTMHKGLKGAPEDRELNLDDTQGMFLLLGAGFGIGLLVLIIEISVWSSEQRKNRQFGELTLKQRAINKLKEHWETLYACLLAPANSGIIYFRERRVSSAFGEYVTQPYASWSITSIPSPTVEPPSPSPPNGEISSAPTVGNAASLSMDQLSFPPEKPIRMMSF

>AlinIR8a

MRTCLWFHVVLFVAHEVAGQGVKLLVVKDNNAGIWDSVSTSFFEQLPVTVDKEDTNSTINDLCEVLKEGVWGVLDLTWSGLDEIKAVCNTWGLPYVRLEYGITQYLRGADKSLATIRKAPDAALIFQTEEQLDQSLFYLIRESSMRVILFKGLSDKEAETLTTMRPTPNFNIIFADTPSMNVMFLKAVERNLVRYDDRWILVFLDNEHNSFDRKTLVKRVTLATPTIDANAAANFAENVAETLEEVAKTSGIDLSPVPAQCEGSSTAAKDLTVFQEKLSEIVEKKPWLDWRQQESTMALHLDMDWTAESSKGEKLFIGSWNSKKGLTIAGNVTKIPRFFRVATGYMVPFAYPVIDPSTGAPKLDDKGNEVWEGYCIDLINRLAEDMDFDYELTTSYNFGRKLPNGSWDGLIGDLASGRVDIIVAALTMTSEREEVIDFVAPYFEQTGFSIVIRKPLRKTSLFKFMTVLRVEVWFSILAALCLTAFMIWFLDKYSPYSARNNKDKYPYPTREFTLRESFWFAVTSFTPQGGGEAPKSLSARTLVAAYWLFVVLMLATFTANLAAFLTVERMQSPVQSLKQLARQSRINYTVVQDSDAHSYFRNMKFAEETLYRVWKEITLNASANQSQYRVWDYPIKEQYGHILISMEKTGTVNSTEEGFQKVRENEDAEFALIHDALEIKYEVYRDCNLTEIGEPFAEQPYSIAVQQGSHLNEEISRRILDLQKDRYFESLSGKYWNSTMKGKCDSSDEDEGITLESLGGVFIATLFGLVLAMLTLGIEIVYERKAKKNVIKVKSAKPEKSEKSEKKEKMMNNPFFNDDKLFSREFGSFPKKPSKLLAPKPKVSFITVFPRDQLY

>AlinIR76b

MSPFVHLMLVAMCANYQTNLLATDNQNFTCILKSEEQIKKEVYKGKVIKILTFDEMPLSGARKDGKGGMIGEGVAFELVETLKEKFGFDYTVERMAPIVGDESHGALGKLVSREIDMVAAFIPVLPDAHEFVKFGKDLSQAAYYVMLKRPADSNSGSGLLAPFDTVVWLLILVSLAVVGPVFYGVMWLRDRLCPGDIDQVYPLSTCVWFVYGALMKQGSTLNPLADSARMVFATWWIFILILTAFYTANLTAFLTLSISTLPIKEIDDVAKDNRHWFALQGGPIEHAIKDKEDEKLRKLRDSAASGRATFLETKQESIILQKITNDWYYLDDSYSLTRMMYDDYNRKSDMNAESSLRCAYVLTEKPFLVRSLAFAYGKDSPLPDLFNPILERFIESGILQHKLNLDLPDAVICPKDLGNKERKLRNADLWTTYLVVFSGVSVAFMIFLIEIIWRFYRKVKGSNQGVFNKQQMFQSRLNTDKLLAMRDQVQTKINGRDYYMVTNKGGNSHFIPLRTPSALLFQYG

>AlinGluR1

MIREWMNMFTAAAFLLACAHVASALPEVIKIGAVFEEGDEWSRYAFEAAIKIINEDESTLPGIKLEAAIAEEPIPQYDIVGVENHVCDMMKAGVVGIVGPHSTEMSNHVQSLCDTMEIPHISARWDGQQRRSSCLVNLYPHPSVLAQVAADMVRTWDWKGFTVLYDDFNALRKIGELVKVADDKGLIVSVRQIKGTEEEESNYRYVLQQVKHSGETNIVVEVSRERLFDVMMQAQQVGLVGGEYSYIITSLDFQSIDLEPFKWAGTNITGIRIVNPDQPHFKEVMKLIMEMKTQGEGGEGAGEEGGGEEGEKEEVGENNGDEAEEGADAEDELGEGEEEGGGKEEENKRRKRRTQQDDEENEVIGGEEEGAEGGEEEGGGEEEVGENGEGGGEGPDDGAEGNQEEENGEGGEEEAGGGAPEEAPAEEEEEYEIPPIEALLIYDAVNLVAEALHNLDIVEPREIDCRQNNAWESGYSVINFVKMSEQAGLTGLVKFDNEGFRSEVVLEIVELVQEGLRVKGNWTKQDGVTIHYIGGEAGPTDIGDDLRNTTFVVLIALTHPYGMLKEDSRQLMGNDRFEGFGIDLIHELSMMSGFNYTFRVQEDKSSGSPKTLENGTRVWSGMIGEVLAGRADLAIADMTITRERERDVDFTMPFMNLGISILYRKPMAMPPSLFSFLSPFSYEVWGYILSAYLGVSFLLFIMARISPYEWTNPYPCIEEPTELETQFSLSNSLWFTTGSILQQGSDVAPISVSTRMVAAIWWFFTLIMVSSYTANLAAFLTIEQKIEPFTDVEGLANSEGIKYGAKKGGATANFFRDSNEPIFQKMWTFMEANPDVMPTSNEAGVARVQENTDYAFLMESASIEYEQERKCELTMVGDLLDSKGYGIAMRQNSSYRNVLSRNVIKLQEKGKLTQLRDKWWKEKRGGGACNAEEEGGEASELGLDNVGGVFVVLLGGCILSVFLAFGELLCDIYGREDKVSFKDELIEEIKFIARCHGTVKPVRKGEPANSSSSSSKSGSRSGSKSGSKTRSSRTRTRSGSGGSPSVERHRFSSRLYSDPLSLD

>AlinIR93a

MSTGLFLVQFVLLMIMLKDHECNAAIKLHNDKRANDTLLIIIDESFVDSLDRNIEQQVRQIVSEISSRILKKGAVDILYHSNANIYLEPDVTAVFSMTSCLDLWTLFNRGKKFDILFISLTEANCPRLPPDSGITVPLYRRGWEIPQIILDLREDGSLTWETTAIIFDDNLEEDMLKSIIEVLNRPKKSSEMACSVVLYRLHSFPKDIQNKKKKNMDNLLENLPPIDMVNNFLVLIDQRKITPTLELVKKMGLVIPTAQWMFVVRNLNVMKRRTSPEKRYVDLIGEGENVAFLINSTRADSRCDMGLLCNARQLVEKLIIAIEKSIEQEIILADSLSDEEWDVLKPKKLERREHILDFIKKKTRDEASDCDSCTEWIIRSSDSWGMDFVQSKGSYGNKESNSTTGALLEVGHWQPRSGLVLVDHLFPNIVGGFRGRTLPIASVHFPPWQFVKYDEFGQPSEYGGVVFNVLNELADKLNFTYEIVLLPNGTSAANKFTLHKELGEVVIDSSVEFAAWDQVVLDLKSNKVFLGAVAFVETEERKADVNFTHPVATDAYAFLVSRPKELSRALLFIQPFTGETWLCIIATILLAGPLLWFVHRVTPFYDHYSHRGKGGYTRLYNCFWYLYGALLQQGGGVMPEADSGRIVIGTWWLVVLVVVTTYSGSLVAFLTFPKMDKVISNVDQLLERSAVSGDGMITWSFPKISTIHRLLKDTDNKKFNMFYEASEKLEQLTPEIIAKIQNGEHVYIQRKTMLLYIMKQEFLRTQRCDYSIGSEEVLKERLGLVVKSNSPYLKIINQHIHDMHKVGLINKWLEDSLPKKDKCWMSTLGSSSSTHTVNMSDMQGCFFLLFIGVFTSILLIGGECFMKWWKLRKQKSIIQPFIS

>AlinIR75q

MQQVGLVGKDYNYIITSLDLHTIDLDIFKWAGTNITGVRLVNTESEHYREIMELMLAIKKEEEEEAYRPFTYDEQRRKRRNISSTLKSEDLGSNKIMSATSGRKTYSKRELQNKEGSRIIPPVEAMLIYDAVVLAAQALHSLSHVNPKQINCLMRSAWESGYSVINYMKMSEFYGLTGEVKFDNEGFRTDVALDIIELTQSGLHVKGNWSTYGGVNIQYPEPETDLTEATDDLRNTTFVVIIALTHPYGMLKESKYTLVGNDRFEGFGIDLIHELSEMTGFNYTFRVQEDKSSGNPTTLPNGTRVWNGMIGEVLAGRADLAIADITITREREHDVDFTMPFMSLGISILYRQPRAAPPSLFSFLSPFSYEVWGYMLSAYLGVSFLLYLMARISPPEWTNPYACIEEPVELENQFSLSNSLWFTIGSLMQQGSEIAPIAVSTRLVAAIWWFFTLIMVSSYTANLAAFLTIEQKVSLFDNVQELADQEVIKYGAKRGGSTANFFRDSHDPTYKKMWEFMSSHPEVMTDSNEVGVDRVDSTTDYAFLSESTSIEYETERRCNLYKVGRELDEKGYGIAMRQNSTYRNVLSRSVVKLQEGGQLDELKKKWWKEKRGGGSCLESPSGGAEDLGLDNVGGVFVVLLGGCIFATFLAFGELSFAIYMMEDKESFKEEFKKELKFIMKCSGTSKPRKIPSISSSTSSNPSVRSKSRSASRSRTSTVRTPNFSLQFP

>DmelGluRIIC

MWQRILLLGCMWSAFFMCRSRGQQINIGAFFYDDELELEKEFMTVVNAINGPESEQTMRFYPLIKRLKPEDGSVTMQEHACDLIDNGVAAIFGPSSKAASDIVALVCNSTGIPHIEFDISDEGIQAEKPNHQMTLNLYPAQAILSKAYADIVQNFGWRKFTIVYDADDARAAARLQDLLQLREVHNDVVRVRKFHKDDDFRVMWKSIRGERRVVLDCEPNMLVELLNSSTEFGLTGQYNHIFLTNLETYTDHLEELAADNETFAVNITAARLLVNPDPPPYSLPYGYVTQRDNIVYESSDPPRTLIHDLIHDALQLFAQSWRNASFFYPDRMVVPRITCDFAASGGRTWAMGRYLARLMKGTSGVNNTNFRTSILQFDEDGQRITFNIEVYDPLDGIGIAIWDPRGQITQLNVDVKAQKKMIYRVATRIGPPYFSYNETARELNLTGNALYQGYAVDLIDAIARHVGFEYVFVPVADQQYGKLDKETKQWNGIIGEIINNDAHMGICDLTITQARKTAVDFTVPFMQLGVSILAYKSPHVEKTLDAYLAPFGGEVWIWILISVFVMTFLKTIVARISKMDWENPHPCNRDPEVLENQWRIHNTGWLTVASIMTAGCDILPRSPQVRMFEATWWIFAIIIANSYTANLAAFLTSSKMEGSIANLKDLSAQKKVKFGTIYGGSTYNLLADSNETVYRLAFNLMNNDDPSAYTKDNLEGVDRVRKNRGDYMFLMETTTLEYHREQNCDLRSVGEKFGEKHYAIAVPFGAEYRSNLSVAILKLSERGELYDLKQKWWKNPNASCFEEPDPDATPDMTFEELRGIFYTLYAGILIAFLIGITEFLVYVQQVALEERLTFKDAFKKEIRFVLCVWNNRKPIVAGTPISSVRTTPRRSLDKSLDRTPKSSRRVVIGRSSEEMREMAQGSGSSSGSNNAGRGEKEARV

>DmelGluRIIA

MRLCPVVIYAFIIIIGFLEGIIALGGDDRNEITVGAIFYENEKEIELSFDQAFREVNNMKFSELRFVTIKRYMPTNDSFLLQQITCELISNGVAAIFGPSSKAASDIVAQIANATGIPHIEYDLKLEATRQEQLNHQMSINVAPSLSVLSRAYFEIIKSNYEWRTFTLIYETPEGLARLQDLMNIQALNSDYVKLRNLADYADDYRILWKETDETFHEQRIILDCEPKTLKELLKVSIDFKLQGPFRNWFLTHLDTHNSGLRDIYNEDFKANITSVRLKVVDANPFERKKTRLTKVDQILGNQTMLPILIYDAVVLFASSARNVIAAMQPFHPPNRHCGSSSPWMLGAFIVNEMKTISEDDVEPHFKTENMKLDEYGQRIHFNLEIYKPTVNEPMMVWTPDNGIKKRLLNLELESAGTTQDFSEQRKVYTVVTHYEEPYFMMKEDHENFRGREKYEGYAVDLISKLSELMEFDYEFMIVNGNGKYNPETKQWDGIIRKLIDHHAQIGVCDLTITQMRRSVVDFTVPFMQLGISILHYKSPPEPKNQFAFLEPFAVEVWIYMIFAQLIMTLAFVFIARLSYREWLPPNPAIQDPDELENIWNVNNSTWLMVGSIMQQGCDILPRGPHMRILTGMWWFFALMMLSTYTANLAAFLTSNKWQSSIKSLQDLIEQDKVHFGSMRGGSTSLFFSESNDTDYQRAWNQMKDFNPSAFTSTNKEGVARVRKEKGGYAFLMETTSLTYNIERNCDLTQIGEQIGEKHYGLAVPLGSDYRTNLSVSILQLSERGELQKMKNKWWKNHNVTCDSYHEVDGDELSIIELGGVFLVLAGGVLIGVILGIFEFLWNVQNVAVEERVTPWQAFKAELIFALKFWVRKKPMRISSSSDKSSSRRSSGSRRSSKEKSRSKTVS

>DmelGluRIIB

MHGLQFLVLLALAIASGANEDTLVIKIGAIFFDTEMKLADAFSAALEEVNAINPALKLDAIKRYVTVDDSIVLQDISCDLIGSGVAAIFGPSSKTNSDIVEVLCNMTGIPHLQFDWHPQQSNRERMNHQLTVNVAPMELFLSAAFSDILASKTFDWKSFTIAYERSSHLIRLQHILAWKQLHKAGIKMQEFERGDDYRILWKRINNAREKFVLLDCPSDILVDVINASIGYNMTGSFNHLFLTNLDTHLSGIDGFYSRDFTVAVAAVRIRTYVPPPVHDEIDVFDNSVDTRFSSLGSQLVYDSIVLFYNALLEISQRPGFYIPNFSCGRGFWQPGPRLVEQMKQITPKMVKPPFKTQRLQINADGQREDFNLEVYNPIIDRVTHIWNKEFQLVDFEKLRENSTQALKQKRLQNKEDFSQKPIRYTVATRVGKPYFSWREEPEGVHYEGNERFEGYAVDLIYMLAQECKFDFNFEPVRDNKYGSYDANTDEWDGIIRQLIDNNAQIGICDLTITQARRSVVDFTVPFMQLGISILSYKEPPPKADIYAFLNPYNAEVWLFVMIAMMITAFALIFTGRIDQYEWDQPVENVNREMERQNIWHLSNALWLVLGSMLNQGCDLLPRGLPMRLLTAFWWIFALLISQTYIAKLAAFITSSKIAGDIGSLHDLVDQNKVQFGTIRGGATSVYFSESNDTDNRMAWNKMLSFKPDAFTKNNEEGVDRVKLSKGTYAFLMETTNLQYYVQRNCELTQIGESFGEKHYGIAVPLNADFRSNLSVGILRLSERGELFKLRNKWFNSNESTCDSNVPTIDDGQFDMDSVGGLFVVLIVGVVVGLVIGVAEFLWHVQRISVKEKIPPMLALKAEFYFVIRFWLTRKPLHTYRQSRDSTSTGYSSLEQITSASSAKKKKKTRRIEK

>DmelClumsy

MYSLFLTHFLLIALPVLADIDRSQFMVGSIFTSDKDESEIAFRTAVDRANILERNVELVPIVVYANTDDSFIMEKMVCNLISQGVIAIFGPSTGSSSDIIASICDTLDIPHIVYDWIPNESIPDREHSTMTLNVHPDNLLLSQGLAEIVQSFAWRSFTVVYETDKELQQLQDILQVGEPISNPTTVKQLGPGDDHRPFLKEIKLSTDNCLILHCAPDNLLKILQQANELKMLGEYQSVFIPLLDTHSIDFGELSGVEANITTVRLMDPSDFHVKNVVHDWEEREKREGRYFKVDPNRVKSQMILLNDAVWLFSKGLTELGIFEELTAPDLECRRKKPWPFGKRIIEFIKARSEETSTGRIDFNENGQRSFFTLRFMELNSDGFLDLATWDPVNGLDVLNDDEESEKRVGQKLSNKTFIVSSRLGAPFLTLREPQEGEILTGNSRYEGYSIDLINEIAKMLNFKFEFRMSPDGKYGALNKVTQTWDGIVRQLIDGNADLGICDLTMTSSRRQAVDFTPPFMTLGISILFSKPPTPPTDLFSFLSPFSLDVWIYMGSAYLFISLLLFALARMAPDDWENPHPCKEPEEVENIWSIMNTTWLSIGSLMGQGCDILPKAASTRLVTGMWWFFALMMLNSYTANLAAFLTNSRQANSINSAEDLAAQSKIKYGAMAGGSTMGFFRDSNFSTYQKMWTAMESASPSVFTKTNDEGVERVQKGKNLYAFLMESTTLEYNVERKCDLVQIGGWLDYKSYGIAMPFNSPYRKQISAAVLKLGELGQLAELKRKWWKEMHGGGNCEKSDEDGGDTPELGLENVGGVFLVLGLGLLSAMVLGCTEFLWNVKSVAIEEKISLKEAFKSEALFAARIWITTKPVHTSSESGSSNSSSSSSSRSKHSFKSQGLSMKSLKSSGYQDVEASVHSKLKKIGSMFSLKSQKTVTPPPEIGWKLDKSTQIDVVPTSDVDQELIPEVEPHLPHRHHHHHHHRHHHHHHQPDQEHDRNPSPPE

>DmelGluRIID

MHFCWISLIILSLSRVQAQFYGGNAYEASSGQSIRLGLITDDATDRIRQTFEHAISVVNNELGVPLVGETEQVAYGNSVQAFAQLCRLMQSGVGAVFGPAARHTASHLLNACDSKDIPFIYPHLSWGSNPDGFNLHPSPEDIANALYDIVNQFEWSRFIFCYESAEYLKILDHLMTRYGIKGPVIKVMRYDLNLNGNYKSVLRRIRKSEDSRIVVVGSTTGVAELLRQAQQVGIMNEDYTYIIGNLNLHTFDLEEYKYSEANITGIRMFSPDQEEVRDLMEKLHQELGESEPVNSGSTFITMEMALTYDAVRVIAETTKHLPYQPQMLNCSERHDNVQPDGSTFRNYMRSLEIKEKTITGRIYFEGNVRKGFTFDVIELQTSGLVKVGTWEEGKDFEFQRPPQAVNFNDIDDGSLVNKTFIVLISVATKPYASLVESIDTLIGNNQFQGYGVDLIKELADKLGFNFTFRDGGNDYGSFNKTTNSTSGMLKEIVEGRADLAITDLTITSEREEVIDFSIPFMNLGIAILYVKPQKAPPALFSFMDPFSSEVWLYLGIAYLGVSLCFFIIGRLSPIEWDNPYPCIEEPEELENQFTINNSLWFTTGALLQQGSEIAPKALSTRTISAIWWFFTLIMVSSYTANLAAFLTIENPTSPINSVKDLADNKDDVQYGAKRTGSTRNFFSTSEEPIYIKMNEYLNAHPEMLMENNQQGVDKVKSGTKYAFLMESTSIEFNTVRECNLTKVGDPLDEKGYGIAMVKNWPYRDKFNKALLELQEQGVLARLKNKWWNEVGAGVCSAKSDDDGPSELGVDNLSGIYVVLVIGSIISIIISILCWCYFVYKKAKNYEVPFCDALAEEFRIVIRFSENERPLKSAQSIYSRSRNSSQSIESLKTDSEENMPVED

>DmelGluRIIE

MFFNHFVILWSLFSIHISVNWAQYENFGGYDNYQSLESVPIGLLTDQNTEQMNIVFDHAIDVANQEVGTSLTSLKEEVNYGDAYQSYGKLCRMLETGIAGVFGPSSRHTAVHLMSICDAMDIPHIYSYMSENAEGFNLHPHPADLAKALYSLITEFNWTRFIFLYESAEYLNILNELTTMLGKSGTVITVLRYDMQLNGNYKQVLRRVRKSVDNRIVVVGSSETMPEFLNQAQQVGIINEDYKYIIGNLDFHSFDLEEYKYSEANITGLRLFSPEKMAVKELLMKLGYPTDQDEFRNGSCPITVEMALTYDAVQLFAQTLKNLPFKPMPQNCSQRTESVRDDGSSFKNYMRTLRLTDRLLTGPIYFEGNVRKGYHLDVIELQPSGIVKVGTWDEDRQYRPQRLAPTTAQFDSVDNSLANKTFIILLSVPNKPYAQLVETYKQLEGNSQYEGYGVDLIKELADKLGFNFTFVNGGNDYGSYNKSTNESTGMLREIMTGRADLAITDLTITSEREQALDFTIPFMNLGIAILYLKPQKATPELFTFMDPFSEEVWWFLGFSFLGVSLSFFILGRLSPSEWDNPYPCIEEPEELENQFTLGNSIWFTTGALLQQGSEIGPKALSTRTVASFWWFFTLIVVSSYTANLAAFLTIEKPQSLINSVDDLADNKDGVVYGAKKTGSTRNFFMTSAEERYKKMNKFMSENPQYLTEDNMEGVNRVKTNTHYAFLMESTSIEYNTKRECNLKKIGDALDEKGYGIAMRKDWPHRGKFNNALLELQEQGVLEKMKNKWWNEVGTGICATKEDAPDATPLDMNNLEGVFFVLLVGSCCALLYGIISWVLFVMKKAHHYRVPLRDALKEEFQFVIDFNNYVRVLKNSASIYSRSRQSSMSVASVAQESQ

>DmelCG3822

MRSSGVLVLPLLLLQLILNCRKAQSLPDIIKIGGLFHPADDHQELAFRQAVDRINADRSI

LPRSKLVAQIERISPFDSFHAGKRVCGLLNIGVAAIFGPQSSHTASHVQSICDNMEIPHL

ENRWDYRLRRESCLVNLYPHPNTLSKAYVDIVRHWGWKTFTIIYENNDGIVRLQELLKAHGMTPFPITVRQLSDSGDYRPLLKQIKNSAEAHIVLDCSTERIHEVLKQAQQIGMMSDYHSYLVTSLDLHTVNLDEFRYGGTNITGFRLINEKIVSDVVRQWSIDEKGLLRSANLTTVRSETALMYDAVHLFAKALHDLDTSQQIDIHPISCDGQSTWQHGFSLINYMKIVEMKGLTNVIKFDHQGFRTDFMLDIVELTPAGIRKIGTWNSTLPDGINFTRTFSQKQQEIEANLKNKTLVVTTILSNPYCMRKESAIPLSGNDQFEGYAVDLIHEISKSLGFNYKIQLVPDGSYGSLNKLTGEWNGMIRELLEQRADLAIADLTITFEREQAVDFTTPFMNLGVSILYRKPIKQPPNLFSFLSPLSLDVWIYMATAYLGVSVLLFILAKFTPYEWPAYTDAHGEKVESQFTLLNCMWFAIGSLMQQGCDFLPKALSTRMVAGIWWFFTLIMISSYTANLAAFLTVERMDSPIESAEDLAKQTRIKYGALKGGSTAAFFRDSKISTYQRMWSFMESARPSVFTASNGEGVERVAKGKGSYAFLMESTSIEYVTERNCELTQVGGMLDTKSYGIATPPNSPYRTAINSVILKLQEEGKLHILKTKWWKEKRGGGKCRVETSKSSSAANELGLANVGGVFVVLMGGMGVACVIAVCEFVWKSRKVAVEERLSAILNE

>DmelCG5621

MISTEASFPLGFILTSLLLAFPGCRGERTNVGLVYENTDPDLEKIFHLAISKANEENEDL

QLHGVSVSIEPGNSFETSKKLCKMLRQNLVAVFGPTSNLAARHAMSICDAKELPFLDTRWDFGAQLPTINLHPHPATLGVALRDMVVALGWESFTIIYESGEYLPTVRELLQMYGTAGPTVTVRRYELDLNGNYRNVLRRIRNADDFSFVVVGSMATLPEFFKQAQQVGLVTSDYRYIIGNLDWHTMDLEPYQHAGTNITGLRLVSPDSEQVQEVAKALYESEEPFQNVSCPLTNSMALVYDGVQLLAETYKHVNFRPVALSCNDDSAWDKGYTLVNYMKSLTLNGLTGPIRFDYEGLRTDFKLEVIELAVSGMQKIGQWSGEDGFQENRPAPAHSLEPDMRSLVNKSFVVITAISEPYGMLKETSEKLEGNDQFEGFGIELIDELSKKLGFSYTWRLQEDNKYGGIDPKTGEWNGMLREIIDSRADMGITDLTMTSERESGVDFTIPFMSLGIGILFRKPMKEPPKLFSFMSPFSGEVWLWLGLAYMGVSISMFVLGRLSPAEWDNPYPCIEEPTELENQFSFANCLWFSIGALLQQGSELAPKAYSTRAVAASWWFFTLILVSSYTANLAAFLTVESLVTPINDADDLSKNKGGVNYGAKIGGATFNFFKESNYPTYQRMYEFMRDNPQYMTNTNQEGVDRVENSNYAFLMESTTIEYITERRCTLTQVGALLDEKGYGIAMRKNWPYRDTLSQAVLEMQEQGLLTKMKTKWWQEKRGGGACSDADEDSGAVALEISNLGGVFLVMGVGSFFGIFVSLLEMVLGVKERSDENQEAPDSDASSLGFANLGGVYLVMFVGSCFGSIYGLVNCVVSVYLRARENKVSFKTELLDEIRFILQCSGNTKAVKYPKNSSRSNASSKSKGSSMSVDSLPEDTSEADASGKHNHGKK

>DmelCG9935

MLIASGFLLFQFLSYGLGVPPLVRIGAIFSNQPGMYNSELAFRYAIHRLNMDKSLLPETTVDYYVEYVNRFDSFETVQKVCKLIRVGVQAVFSPTDSVLATHINSICDALDIPNIGRSAHDFSINVYPSKQLVNYAFNDVIQYLNWTRFGILHEKENGIINLHQLSRSFHGEVHMRQVSRDSYVSALNEFKGKEIHNIIIDTNSNGISILLKNILQQQMNEYKYHYLFTSFDLETYDLEDFKYNFVNITSFRLVDTADVGVKQILKDIGLYSHHIFKKPYLNLHIKKSTILESEPALMFDSVYVFAIGLQTLEQSHSLTLLNISCEEENSWDGGLSLINYLNAVEWKGLTGPIQFKDGQRVQFKLDLIKLKQHSIVKVGEWTPHGHLNITEPSMFFDAGSMNVTLVVITILETPYVMMHYGKNFTGNERFYGFCVDILETISREVGFDYILDLVPDRKYGAKDPETGEWNGMVAQLMKYKADLAVGSMTITYARESVIDFTKPFMNLGISILFKVPTSEPTRLFSFMNPLAIEIWIYVLIAYFLVSLCIYIVGKLSPIEWKCINACDLENISIGNQFSLTDSFWFTIGTFMQQSPDIYPRAMSTRIISSTWGFFSLIIVASYTANLAAFLTTERMINPIENAEDLASQTEISYGTLDSGSTMTFFRDSVIETYKKIWRSMDNKKPSAFTTTYEDGIKRVNQGNYAFLMESTMLDYIVQRDCNLTQIGGLLDTKGYGIATPKGSPWRDKISLAILELQERGDIQMLYDKWWKNTDETCTRKNTSKQSKANSLGLESIGGVFVVLIAGIIVAAVVAFFEFWYNFRYNYEATPSQSVVNNKYNQDGILESERNYTPPDRSFWIEIAEELRYASWCMNKQKRPALTRTCSKCTIPKGQRINKL

>DmelCG11155

MVRKKREIVIKENIQGRSYLKKICCSYIILSILVISNALPPVIRVGAIFTEDERESSIES

AFKYAIYRINKEKTLLPNTQLVYDIEYVPRDDSFRTTKKVCSQLEAGVQAIFGPTDALLASHVQSICEAYDIPHIEGRIDLEYNSKEFSINLYPSHTLLTLAYRDIMVYLNWTKVAIIYEEDYGLFNLMHSSTETKAEMYIRQASPDSYRQVLRAIRQKEIYKIIVDTNPSHIKSFFRSILQLQMNDHRYHYMFTTFDLETYDLEDFRYNSVNITAFRLVDVDSKRYLEVINQMQKLQHNGLDTINGSPYIQTESALMFDSVYAFANGLHFLNLDNHQNFYIKNLSCTSDQTWNDGISLYNQINAAITDGLTGTVQFVEGRRNIFKLDILKLKQEKIQKVGYWHPDDGVNISDPTAFYDSNIANITLVVMTREERPYVMVKEDKNLTGNLRFEGFCIDLLKAIATQVGFQYKIELVPDNMYGVYIPETNSWNGIVQELMERRADLAVASMTINYARESVIDFTKPFMNLGIGILFKVPTSQPTRLFSFMNPLAIEIWLYVLAAYILVSFALFVMARFSPYEWKNPHPCYKETDIVENQFSISNSFWFITGTFLRQGSGLNPKATSTRIVGGCWFFFCLIIISSYTANLAAFLTVERMISPIESASDLAEQTEISYGTLEGGSTMTFFRDSKIGIYQKMWRYMENRKTAVFVKTYEDGIKRVMEGSYAFLMESTMLDYAVQRDCNLTQIGGLLDSKGYGIATPKGSPWRDKISLAILELQEKGIIQILYDKWWKNTGDVCNRDDKSKESKANALGVENIGGVFVVLLCGLALAVVVAIFEFCWNSRKNLNTENQSLCSEMAEELRFAMHCHGSKSRHRPRKRSCLNCSSVPTYVPSNVSTSNVGVYYNYFN

>DmelGlu-R1

MHSRLKFLAYLHFICASSIFWPEFSSAQQQQQTVSLTEKIPLGAIFEQGTDDVQSAFKYAMLNHNLNVSSRRFELQAYVDVINTADAFKLSRLICNQFSRGVYSMLGAVSPDSFDTLHSYSNTFQMPFVTPWFPEKVLAPSSGLLDFAISMRPDYHQAIIDTIQYYGWQSIIYLYDSHDGLLRLQQIYQELKPGNETFRVQMVKRIANVTMAIEFLHTLEDLGRFSKKRIVLDCPAEMAKEIIVQHVRDIKLGRRTYHYLLSGLVMDNHWPSDVVEFGAINITGFRIVDSNRRAVRDFHDSRKRLEPSGQSQSQNAGGPNSLPAISAQAALMYDAVFVLVEAFNRILRKKPDQFRSNHLQRRSHGGSSSSSATGTNESSALLDCNTSKGWVTPWEQGEKISRVLRKVEIDGLSGEIRFDEDGRRINYTLHVVEMSVNSTLQQVAEWRDDAGLLPLHSHNYASSSRSASASTGDYDRNHTYIVSSLLEEPYLSLKQYTYGESLVGNDRFEGYCKDLADMLAAQLGIKYEIRLVQDGNYGAENQYAPGGWDGMVGELIRKEADIAISAMTITAERERVIDFSKPFMTLGISIMIKKPVKQTPGVFSFLNPLSQEIWISVILSYVGVSFVLYFVTRFPPYEWRIVRRPQADSTAQQPPGIIGGATLSEPQAHVPPVPPNEFTMLNSFWYSLAAFMQQGCDITPPSIAGRIAAAVWWFFTIILISSYTANLAAFLTVERMVAPIKTPEDLTMQTDVNYGTLLYGSTWEFFRRSQIGLHNKMWEYMNANQHHSVHTYDEGIRRVRQSKGKYALLVESPKNEYVNARPPCDTMKVGRNIDTKGFGVATPIGSPLRKRLNEAVLTLKENGELLRIRNKWWFDKTECNLDQETSTPNELSLSNVAGIYYILIGGLLLAVIVAIMEFFCRNKTPQLKSPGSNGSAGGVPGMLASSTYQRDSLSDAIMHSQAKLAMQASSEYDERLVGVELASNVRYQYSM

>DmelGlu-R1B

MRFGLKLSCLWPSFLLWLTWSSGGGGGSGVGVSAQPSLTEKIPLGAIFEQGTDEVQSAFKYAMLNHNLNVSSRRFELQAYVDVINTADAFKLSRLICNQFSRGVYSMLGAVSPDSFDTLHSYSNTFQMPFVTPWFPEKVLTPSSGFLDFALSMRPDYHQAIIDTIQFYGWRKIIYLYDSHDGLLRLQQIYQGLRPGNESFQVELVKRISNVSMAIEFLHTLEQIGRFENKHIVLDCPTEMAKQILIQHVRDLRLGRRTYHYLLSGLVMDDRWESEIIEFGAINITGFRIVDTNRRLVREFYDSWKRLDPQMSVGAGRESISAQAALMYDAVFVLVEAFNKILRKKPDQFRNNVQRRSQTLMVAQAAASTSSDGYNYSASGGGGGNGGAGGGFAGSDSGGSGGMASRALDCNTAKGWVNAWEHGDKISRYLRKVEIEGLTGDIKFNDDGRRVNYTLHVVEMTVNSAMVKVAEWNDDAGLQPLNAKYVRLRPHVEFEKNRTYIVTTVLEEPYIMLKQVAFGEKLHGNNRFEGYCKDLADLLAKELGINYELRLVKDGNYGSEKSSAHGGWDGMVGELVRKEADIAIAAMTITAERERVIDFSKPFMSLGISIMIKKPVKQTPGVFSFMNPLSQEIWVSVIFSYIGVSIVLFFVSRFSPHEWRLVQQQPQQSQSPDPHAHHEQLANQQPPGIIGGAPLPAPPGPPTPGAQTAAGAAALQAALSAGSPGSGGSSSAVVNEFSVWNSFWFSLAAFMQQGCDLSPRSVSGRIAAASWFFFTLILISSYTANLAAFLTVERMVTPINSPEDLAMQTEVQYGTLLHGSTWDFFRRSQIGLHNKMWEYMNSRKHVFVPTYDEGIKRVRNSKGKYALLVESPKNEYVNAREPCDTMKVGRNLDTKGFGIATPLGSALKDPINLAVLTLKENGELIKLRNKWWYEKAECSTHKDGETSHSELSLSNVAGIFYILIGGLLVSVFVAILEYCFRSRDSRSASSGSGMGLGMGLGGGMSGGSLGKANGSMMLGPSSAVPGGMPSSHQRSTLTDTMHAKAKLTIQASRDYDNGRVGYLNCASLQYYPPAQLSATPPDAGDSLHMNAHGQV

>DmelNmdar2

MMPSRVKLKRGTDGPTPTPTPMPTTMRKHTPIATLNTASCQHNSTTSRRKRILTPPSGPISLLLLTVLTLLILDTRSCQGLRLTNGGGSLSKGAAANKEQLNIGLIAPHTNFGKREYLRSINNAVTGLTKTRGAKLTFLKDYSFEQKNIHFDMMSLTPSPTAILSTLCKEFLRVNVSAILYMMNNEQFGHSTASAQYFLQLAGYLGIPVISWNADNSGLERRASQSTLQLQLAPSIEHQSAAMLSILERYKWHQFSVVTSQIAGHDDFVQAVRERVAEMQEHFKFTILNSIVVTRTSDLMELVNSEARVMLLYATQTEAITILRAAEEMKLTGENYVWVVSQSVIEKKDAHSQFPVGMLGVHFDTSSAALMNEISNAIKIYSYGVEAYLTDPANRDRRLTTQSLSCEDEGRGRWDNGEIFFKYLRNVSIEGDLNKPNIEFTADGDLRSAELKIMNLRPSANNKNLVWEEIGVWKSWETQKLDIRDIAWPGNSHAPPQGVPEKFHLKITFLEEAPYINLSPADPVSGKCLMDRGVLCRVAADHEMAADIDVGQAHRNESFYQCCSGFCIDLLEKFAEELGFTYELVRVEDGKWGTLENGKWNGLIADLVNRKTDMVLTSLMINTEREAVVDFSEPFMETGIAIVVAKRTGIISPTAFLEPFDTASWMLVGIVAIQAATFMIFLFEWLSPSGYDMKLYLQNTNVTPYRFSLFRTYWLVWAVLFQAAVHVDSPRGFTSRFMTNVWALFAVVFLAIYTANLAAFMITREEFHEFSGLNDSRLVHPFSHKPSFKFGTIPYSHTDSTIHKYFNVMHNYMRQYNKTSVADGVAAVLNGNLDSFIYDGTVLDYLVAQDEDCRLMTVGSWYAMTGYGLAFSRNSKYVQMFNKRLLEFRANGDLERLRRYWMTGTCRPGKQEHKSSDPLALEQFLSAFLLLMAGILLAALLLLLEHVYFKYIRKRLAKKDGGHCCALISLSMGKSLTFRGAVFEATEILKKHRCNDPICDTHLWKVKHELDMSRLRVRQLEKVMDKHGIKAPQLRLASSSDLLNHHHLKERPPLLGNLSLAASAQDLYRWSYKTEIAEMETVL

>DmelNmdar1

MAMAEFVFCRPLFGLAIVLLVAPIDAAQRHTASDNPSTYNIGGVLSNSDSEEHFSTTIKHLNFDQQYVPRKVTYYDKTIRMDKNPIKTVFNVCDKLIENRVYAVVVSHEQTSGDLSPAAVSYTSGFYSIPVIGISSRDAAFSDKNIHVSFLRTVPPYYHQADVWLEMLSHFAYTKVIIIHSSDTDGRAILGRFQTTSQTYYDDVDVRATVELIVEFEPKLESFTEHLIDMKTAQSRVYLMYASTEDAQVIFRDAGEYNMTGEGHVWIVTEQALFSNNTPDGVLGLQLEHAHSDKGHIRDSVYVLASAIKEMISNETIAEAPKDCGDSAVNWESGKRLFQYLKSRNITGETGQVAFDDNGDRIYAGYDVINIREQQKKHVVGKFSYDSMRAKMRMRINDSEIIWPGKQRRKPEGIMIPTHLRLLTIEEKPFVYVRRMGDDEFRCEPDERPCPLFNNSDATANEFCCRGYCIDLLIELSKRINFTYDLALSPDGQFGHYILRNNTGAMTLRKEWTGLIGELVNERADMIVAPLTINPERAEYIEFSKPFKYQGITILEKKPSRSSTLVSFLQPFSNTLWILVMVSVHVVALVLYLLDRFSPFGRFKLSHSDSNEEKALNLSSAVWFAWGVLLNSGIGEGTPRSFSARVLGMVWAGFAMIIVASYTANLAAFLVLERPKTKLSGINDARLRNTMENLTCATVKGSSVDMYFRRQVELSNMYRTMEANNYATAEQAIQDVKKGKLMAFIWDSSRLEYEASKDCELVTAGELFGRSGYGIGLQKGSPWTDAVTLAILEFHESGFMEKLDKQWIFHGHVQQNCELFEKTPNTLGLKNMAGVFILVGVGIAGGVGLIIIEVIYKKHQVKKQKRLDIARHAADKWRGTIEKRKTIRASLAMQRQYNVGLNSTHAPGTISLAVDKRRYPRLGQRLGPERAWPGDAADVLRIRRPYELGNPGQSPKVMAANQPGMPMPMLGKTRPQQSVLPPRYSPGYTSDVSHLVV

**GRs**

>GrubGR1

MFISQKNAIFTKRVNAECYGKNFIRVGTKHSNIHHKFQSQQQPAQNIYFNEMKPIFVMMRIVGRFPYSFTKTEFVPFSFISWPFLYNLVFNLVFGLITTWSMQIIINDRIYPSQSYDETLFWFMLLLFALQSFTGPISCWWDASRIVRYFEKWKDFEDSWKCGSMYSAKRYRFTRIFSFLIMPLVTLFVIYQTLTLPRISIVIFLPHIPIVVALMMMMVHWWMTLHDLTLYSAQLLTSMLKVKDRRGIWYRRRIWLQISNLVTEIGNATGASGLNGAVTNFLGIVLATYGILINLLSPGSSNVSVFGLILPAIACAITMFLTANAASNATEWVGPRFAKKFLQIDLSSLQRNCLSEVDLMLACLSAKSPVIEYLGFIKITRNTFLQFLSHAVTYLVVLVQMKIQPKQLKSHQFLENNGNTTLYEELIS*

>GrubGR2

ESSICVNVREIYPHINQNENPIYINKYLQEMIPLLHILRLFGRFPFLFGDDQVIFKLLSWPVGYSIFVVVLQMLGTCYIMDMVFSLGFSSQGNFDNSIYFICLACSVVESLAIPFTYWNELSRTMKYINDWYHFQEEFGKDELDFVLTRPLKIAGIIQAPFFIIILFGGAYLFDDLRLIIFMPALTSISSLIASWLIFTATLYELRCAAQKLLKRFTAKDCRNIHKNRILWLKLSDLTSDFGDIVGHAGVVVLILLFTTFVLVTYGTLSLAIEQSFNKTFWLLLSASLFVICSQFVLCDAAHRTTKAIGEKFSSNLIELNTSFLSHKDKFEIKLLLETLKTNPPVIRFVGWLTIDRSLFVKLISNTVKYLVVLIQFRTSSGTTASIDSPKGGLIEHLK*

>GrubGR3

MNPTASILFNKYGIPKEYYNWREISLKKKIGIIRKSESAFYKIIKPMLYCLRLIGSFPYFEHKGTPHFKLFSCWTLYSIIFMTVEIILLQLRTKILFKNNENKFDNKLRSQINYFITMFQALGPITLWFEVAGIVKFISKWNSFQKDLNIHNIILDTRLRKWTYGFMVICFIYVIAVSYFEARIYGGIIHIPAIMVIHANLVLTLSLVFFSGLFSLYTATYLMKLIKDSKSSEEIVVCRKLWVKLSHLTTEIGNVLGPTMMEWLLIGSIVVIMSSYSLIYLLHNLVIHNTLDQSEINSGIFFLTVSSILLICLCEFGQRCTHSAGTKVQTELLNLSFTGRDKEFQQEVNLFIETIHFRYPNFVLCGFITLNRTYLVSLISGGITYLIVLVQFRSSQNA

>GrubGR4

MYFSSESVLNLIFLLTKLLGVFPYDHVRKRVSYQWFTYSLIIILVCTVEAAFVVFDPPRLAKNSLLRNILFRCQMLAMYACIVIFNGCILIHRRKISLVFLILDKLKNETRGQPSLLLVLSYLFWQSLAIAATAYMDWVGNTPLEGTVSLALVYYYGMISIIVAGIAPFVALVQLVSVKLEYLVIALKSRVTHSTGRLYSCVLLYDRMVSLLNSLNESFSLQLLLISFVSFFNLTINMFFIADYVVNEASINRSKMVPVYLGWITIFSSMIIFPVYSCHRTIKQAKEFNTQLYQLMIDDTTNDISNNKKLRLHIAMKREVVFTACGFFTLDYTLVHSMIAAATTYLVILIQFGQRQPTGSESLAYSTPFSNSTESPLSPATI*

>GrubGR5

INKDAINLGGSIFEDAIVGSHFIVHASSMLSARRLGFTRLLRIVFPASVIHGSCAMDSSFRFSRCIYCYCLVLGLSALLQDSLWAISNIQNETWSPGFVVDMLSYLLAYVMWAVMAVELVRQKDGFEQVIRELEKVDTFLQNPKYNATFYLIPFFFAVVANLIEKVIVGIDDNSFLQGIGYYVYMSPIVMFSSQYFFLMAVASDQFSYLTNLLRSNYRLWYFKDLMTVHKSLCDTAKKINSIYCLHLLIITSIPFIINALKIYGAIMCVIRPDSYDNSYLLAENIFHVIMNSIIFWCLVHSSSSVQGKARQFNIEFLMILLKGNTIGEESDFQQYLSMDWCVKPTAGGFYNIDYPLLTSTLTGYLTLVTYMLQLTGSTLK*

>GrubGR6

MYLALIEVRNFRFKWISWKVFYSTIIMSVNIILCLFAVNRYIRDRGVSYRSLDRLSGDIIFYGSTLLISQSFLKLVRQWKDLIFRWAALQSEMNSFESSAVQKKIKLLTNCLILSSIFEHILSNTQFMLNVEPNADRLKMFLDNGNITSVLGYNFWLVLFAYIGNFVITLIWNYTDIFLTAISLSLANMFEQFNKELTMTAEKVSQESALSYWRKKRELYNRLSIFVKSVEEKMSRLVLISFAVNLYFICIQLLYSIQPFSSLLRVAYFCGSFGHILFRTCSVCFAATSIYEHSKGSLPLLYSVPSELYNTEVQRLTDQVNNDTLALTGCKFFTVTRSLMLTIAGTIVTYEVVLLQLSNPSVESE*

>GrubGR7

MLRRFESILGTVLKFSLVFGTFPMKKYPMMTYQFSWKLYCYSILVGLIACVEGTYWILVEYHYYSHSVTIIIESIVFWFNIYNWVSVLIHSYLQRVNLPDIVSRLYTIQCHIGITSYKKWYATSIFLLTLLNALPSILYVIEDAIYFTDVLLVSIYFLFINVPVIMASQYSVLMNLLADQLTFLTKQLSLPTYTFDVRQLVENHHALCVQAARINKSFDMFLLQATTIPFVVIVINIYIVIVCILKPEEFDEDSKLLTSVMDAVVNVGIIILIVTAAKNAEDSAMEFNNQLWNNMLISKTLGKDNDLKMYVSMKYGVQQTAYGFFKLNYSLLTSMIASALTYVILLVQFTLLR*

>GrubGR8

MTNGAHNAMSLILAMSRAFGVFAMKPQGGTYVFSRKLHIYFITLTIAAMTEGVIESLTAKSKHAVSLVMVIDIFVYSFYIVTAMIVLFQHYQLRETLPAIIYELGDMERLLGNISYSRYYDHAVIFLATLNASPRIFAVIHKPFTWNHLLMRILYFSFTIIPVLISGQYAILLHILSRQLQTLSEHLNKFLFNLEVWSLMNLHHNLIVLAYKINRAFDVFLFHMITFSFVIVILKLYIVIVYIMKPDSYTDLELTTITFIDFLVICGSIIITVFAAMEATEKAKTFNAQLLKYLLVSKNFAKDEKIRTYLAMKHSIHQSACNFFSLDFNLLTSMAAGTATYVILLVQFTLL*

>GrubGR9

FQTSHIFILTVSILFGVFPLHRIQGTYSFSWKLYLYCLLVAAVTTGYGCWWSYLMLRDNQLSLLLIMDLLVFLLHCTTWVVILFLYYRHRFYMQSVILDLEVIHSRIGIASYHVESYMAGIAVILAVHTVPFLYKLLSNFGTMLFKSSVLLLLEYVYMSAPILLASQYAILLHMVSRELFTITAHMKGAIVDFEFRQILDVNNSLRLLAERINEAFDVYLFQSIAFCYVITLLRLYAVIVCIVAPNTYANDDLLFDSVVDTLRNSGIVVLVVSSAVDATNKAEDFNRELFTNIMVRQSLSKEINISIYLTTRREIKPNPCNFFTLDFSLLTSMVAGTVTYVTLLVQFTLLK*

>GrubGR10

KRTLSSALTISLFFGVFPMRRLHGMKYAFSRKLYFYGICLGLIATGQGIFVATMLIKELSLSMALLMDLFVFIFYCFTWIIVSVQNYLLRNTLPIIITELDDLDSRIAKTTYSRFFNASLILITILNAVPKLIDFSTRLFSWDYLFAGIMYFFSMNAPVLVAGQYSVLLYIISRQLKAITDQLSNANNKPAIRQVMDIHFTLCNLAERVNNAFNLFLFHVITLSFVVIVLKTYIIIICIVRPNLYADSIALVMTVVEVVHNASVIILIVSSAMKASNNAERFNAQLFKTLMMCNRDITDDNTLRTYLNMKHEIKQTACQFFSLDYRLLTSMAAGTTTYVILLVQFTLL*

>GrubGR11

MFNSPVIDKVIFLFDTSNWFGLFPFSINSAGYFQFSLKNSILSIMSLLLTILYNVLCFINYFLLINRDSWVMRGIIYFGVSFQCFYPVMSYFQLFFRLHEFNDFINRIKLASRVLFPVRSFQIPFSKIYLFGNLLLLTIIFVNDACSYLSVLFNIKIFLMLALRYWVVFLLVVLVEMYSVIVGILTSMFKLCNDQLLLLFSSAPRTIVKRLERLAWAHNQLCECAAIISRLQSFQILCVITMCYFVIIIAIYMVVNMAISGPWNSVLALDFFCWCYIYASGVGRILVTSVNCKNKVIIRVILK*

>GrubGR12

MASELVIQEIFYIPTILGTFPFGSQFSLSKKLLAYCSLVRFLSVFGAISAFIYNTEEEEGSFIGVALRSVAVMSALGDNFFTLLWWVLKRHKVSFLMESIVDIQRRKKIILEGPHIRYVHLLLILTLVTHVFSYYNNNTKENSLVNIINEFSYFLSSSSTICFVGQFWDILHLLGHLFRENTQLFDESSVINYERFLMLCEMINDIYGPPILLTITEYFVRIIIYFYELLLPMYPGFSSSNILIIVQAINCIMPIVSMILACNFYIKQVIIFLNYLITVSNVSVVSMIKILIPMV*

>GrubGR13

MDASRTVRYFQKWKDFEDIWECGTIYSVKRLRFIRIFTTLLPPLMIVVATYEALTLPKISAFVFIPYVPILVAAVLMLEHWWMTLYNLTLYSEKLLTSILKVKNRHDIEHRRRMWLKISQLASEIGDATGASGLSYTCTFFTGFILSIYAILINIASPGTSNVSLWGLFFPAVYSSVSYYLAADAAYRVTERIGPIFTKKLLQTDLSFFQQNVSNEVDLLVNCLSSKPPVIEYLGFMKVTRSTFLHVILIFLSYMNIYVIGATTSLVSL*

>GrubGR14

MDYPLIPFTDWVTAPVMIKYINLWHRYECQFKEEFASFPNKIMVQFMCYLFGPVSIYLAYFAGFKMFDSFLLFILYFPLIFAMCLYMLLWLLCQYYLLRQIRKLKGIVISEGLEHRVERIRKCWLLLSELTSCTGDSIGMVLAPCAMIGFLAFITFSYVFFTSLIDDVSSVDWTMGFIIILTLLSYFLLFECADRTTKEVGANFYSVVFDQRFSSLDEENLNQLVLLLATFSSHPPTIKFGGLMVVDRQLFVSLITNSLTFLVVLVQFK

>GrubGR15

MSAVQNCLNRYLLFPNIWGSFIFNFLGQIQHIRVARIILSLVAYVYYFTNEKIPPNFDFRYLIFFTENCLIFSAAGVLLRVSWLLKDIRISLKLMENVSDFFNGTEIKLIIWPSIVSNIVITAAVSYRAYKFSMPPLHFFSDLQILSLINAFTDEFSTMLLLARFLSKALNTHLASVTDRENLVSMLKAQNMLCSAVQHINNLYSVQILFVMALSFFCSISYTFLAIREILLSHIDGISLILWIIIFLQMSVKIILSASSLTNSVM*

>GrubGR16

MVLVICVCIFAIGVELKPLRWYIWCMGTILVTWMVFGLGCYSLDYVTFSTLFYTFSHYYLITLIVLVNSQICWIGGYIASAFSCLTVALEASGDLHQLVRWHRRLTVSCRMLSKVYSMEILILFGTSLVSCTVEAYLTYNILMTRTLVHIAVCVYWTCILYSICFKIIYTCVQAKSKAMEFDAAVYKSIVFNKQGDFSFYGPACRLYFHFQSRGGVTFDVLGFFQIDWQLARTMIGAGIAYLVILVQFS*

>GrubGR17

MYVMLTAVPLVFTNSYYQSIIHGSLIHMPSLFFLHMHTAMYFSFWMISVMFLHSTAKRIIRRLKKVVEDRDHVEIAACRRIWLTLAELTADLGNALGATLMESLLFCSTVLIVSCYFVMFFVRSIYQASTEQQRATLTARIVFIVLTSSTITVLCEFGQRCTNEVGSNLLQELLRMNVSPQEKQLHREVNMFIQSVSLRYPDMVLCGFLTLNRRLLVSLLSGGITYLIVLVQFRSSSDQSQ*

>GrubGR18

VKYSFLMRYCGAFPVNVKGSLFNIDLWKIFLSFLQILGLLAIFYHRTSLINFYLPLIYFLVVLHAFCTHSFLPLNLVWSVLKIKKLNKIMRGFLSIDISLLRMGLKCYNSVGFGFILAQSFAIACVIVAMAISGYSPYVFVFNFLPMLPMIATTQFFSFLMNLLQERFSKLTVSIRPVRYKLIFLTSLYQELFQLSRDINSLFSLQIINIIASSMCTVVIYIFYLIKLLHEGKG

>GrubGR19

RVGYKRTPQHRSSYYNQFRPVLLILRLFGRYSIQLSETGVWESKLLSWITLYCFINYGLQTYVAVIVCFRRVQALVDSQSDYDEFIFSIHILAYLNIHFHVPFTYWLQNSRMAHYLNVWNDFELNWESTFGQKLELIYKKTVLIYVAVMIPSVVLFLVFEQYSTLHDPWLYFLPYFLTIVSTALILALWFVTCIELKRLSEDLSQQLIAASILSESK

>GrubGR20

MRAFDVYWETSPLFSISKIFGIAPYNSFFRLSFVHTPITILFLSLILGDALYVGVYMEKVNDSYQLSGFSLILERFQLIMVILTVVVCMMKSINNVKLIRTVIETLGSVDDYLSGLNLEEKRERYSMYLTLFVRFFSLLSLIMVDFVITRMYRKLDSILVECILMYPLIIINTVETQFIVMLNIVKKRLMVVTKKLNEITKKGVRNINLEILQT

>GrubGR21

WPDLMKEWLEVERQMVGMPSVTAFRKIRIIFLSIFVYAIVEHILWQVYHLEKASLCNKNHFIRRYFLETYSSVFRHIRFSMSLGILLGIINSLLTLVWTFTDLFLICVCMALSARFEELQTSLKRIIGQDMSQEFWRESRKCYIKLSFLAKNLDEKCSLLMLFTFFNNVYSICCLLYKTLKLVSIDFACMLFLPFYSSVQL

>GrubGR22

SILVDICEDINSLYSHQILMIFSGIFILTTTNLYHVVEKLIVFVAKKNVRQLNFATITTYRVMVRAYEVWTVVRSCSKTNEKAKEFNTQLYQLMIDDRFNVISNDKKLRLHIAMKRDVVFTACGFFTLDYTLVHSMIAAATTYLVILIQFGKPNSNSTPDTSSNGTLLINTTDLPLMISTIATFQ*

>AlucGR66b

MVLLIYFAVFFVVVLKPIVLNPVFTVLHHSFHIVLALGISMSFYRIISRKNQLEEVISIIRSTHKFLGLPPSKDGRFGLLVFISVVVITSAELVHFTHNNNVPQKMTHAFFSLYTTIGVSISCQFLWFVNRILECYEGLNKILGREDDPFPQPNRLVIIQNDLVRSAELLNDCYSLQLFFVISSVFCSSTLMGVVLIRNPNMYLKISLAAWVIVNITVPVLIIRCCERLEKKAERFNKLLASCALKDKTERLLTNPHITMHFATQKPLKFTACNCFNIDYRLGCSMIAACVTYLVILVQIDGSKNTIPTLPLNDTTSTPLTQS

>AlucGR66a

MMMVANQFMGLVKHVDLSLSCLLKQLKSPDQTPSKELLKSYHQLYDVSKLIDDAFSLQNLILIIHHVFATTFSMYYFFEAIMHLYPSSSVKKRIWLNLATYILYFSCICLKFALCQTVQSKAKDFKETLYDKIFNDSQEAQTLALRPLYKKIKFSAFDVLDMDFKALKAIIMTIVTYQVILLQFTVSNPSTDKKDFHNNSSSLEQNRSRS

>AlucGR32a

MMGPKRGVAPHHVKVGSLFPGRDTKRTDLMTLLQMSKAFGLLPLDDGLNYSVMWIVYSIGFSVAFTFLSLGYKTYYMVDTDDFENNAWHKIIFYCRALVENIIIVSHLAQVLVHRKELPEVLKEIKKIGLRSTYARYFAPVGYLIIILNFVVIAVLEQNSSTSTRASLATAFCEVLCLIILVQFCAVLEVFKNEMDVLTSRISINQAKLEVLSDRRQVILEMAGSTNKVFSVQLLLICFKLFTDVIYFAYFTIIEIKEIATPKGNPIKVIRMPLYAVWQLFELYSISSSTEKLVESAENFNAELFQLMRDNKDLCKNKKLSLCLALKQTVNFTACGFFTLGYPLVTSIIAAATTYLVILVQFSL

>AlucGR28a

MSLGAHWVSIFRIRPHLPLLIDYLISIDSKLPILVKGLSLSKTYHSLAFLFFSKLAASFVVFGVYLLLHYDGDNTLWRVTLVAHNFVSLTCELQLLIFILMIKSRFQVVNRALSSMCHDMRSARISVKSRKILYPKIEKSSDLHHIELAHDCYLKLVDSWILLNKIYAVQVLFSTGGCFLKALFNVYFLSSSSIDDWPVLQNDLINSVVWMVFYFSRFAMLALVATSASHEFAKTKAITALMSHWYDDSTIREELDIFWTNTSCRKLKFSTCGLFTLDSGLIIKACVTGITYLVLLVQFKPQIAVN

>AlucGR63a

MMLSSVIFMLLYWVLSAFELINIGELIKLRISEELCSLEVKNVEASKIRDLRMAWLGLVEMIKLNTKSWSYIFTYMVLTLLFVIIMTSFGFISILVKENRFDFDLLFVSLSVAALSYIMFECGHRLTHKAGYEICEVLGNVNILNMDKAAKEEVKFFTKAAKMNYPLVTLGGVFIANRAAMTSMVSSIVTNLIVILQLPKDSSSFVHLSNASNNTNIA

>AlucGR22a

MTGNDLKIKLKAWVYTVLTIIPSLSLLASFTEQETLVIRNILQRLLYIMLFSGMSFVAMFWIMSHVVVQRIAVYLKGELMKLGVNSRNRSNVSAMEVHRLRYLWARATSLLHKSGECLGLSMMIISLTNSASFVIAAYGVIVGILEFNQEAVLHQSVNVVAGIAYIFAGTESGYQSTQKMGDDIMAALSAIDLSGINDVTSREINMFLQIMSLNPPIVTCAGVVTISRSFLTSVFSNTVTYLIVLLQFKTTDVEFNCNNETLTSILQKPEG

>HhalGR64f

MTHLFSFTDVVLTRKLILRQTNYMYFDKSLELCQANIRLDEANMDSIIRNGGSQFVQVATIPIADRHKTHKTIESQGVSNKGESVIQALRPVYPIARILALPDNDEKFTWRNWKTMTCLFLLTWGIFLSSATVIKVSGETFSYTAVGQITFYCIVTVYLILFLKLSTEWPDLMEEWLEVEQQMVGMPPVRAVHKIRIVFLSIFIYAIVEHVLCQLYNLERARMCDENNFIERYFLEIYDNVFRHFRFSMWMGIILVMMNFFLTLVWTFTDIFLMSVCIALSVRFEQLQTSLKRIMGQDMSQEFWRESRKRYIKLSFLVKNLDEKFSLLMFFTFFNNVYSICCLLYRALKWRDLTTIHTIYNCAAFILVTVRLYGICLAAGKIHDISKDILPVLFAVPSQSYNVEVHRLFLQIWQDPVSLTGSRFFAITRGLVLKITGTIFTYEIVLAQFNQINPENENTMPQTNCNSNPVKLNET

>HhalGR66a

MIAIGLTVALLEIAMSCGQFCFLVDTVSDFFDSGAKLNRVEQDNLSNTAELRGVPNKAGEIYEGIVCPIGGALGIKLGLEDLDFVDWKRPREKDFHPPPILVRFFRRAVSDELIRLRKVKRDFSTRHFDWDSDERINIGEEMTIACIKPPVPFEKLIITEKLVDVVNQLVSTSCKINAIYSLPLLTTVLASYISIICHLFFIYENGNNGELRLHNVPADCLMLLYRSLVVWRLSHSAAVAHRKSKKFNVLLYKLMIEDKTNEFLRNDKLKLHIGMKREVVFTACGFFNLDYTLVHSVIASATTYLVISIQFGELLN

>HhalGR28a

MRAFDVYWETSPLFSISKIFGIAPYNSIFRLSLVHTSITILFLSLILGDALYLGIYMEQVNDSYQLSGFSLMLERFQLIMVIVTLIVCMMKAINNVKLIRIVMETLGTVDDYLLGLNVEEKRERYSMYVTLFVRLFSLFSLIMVDLVITKMYRKLDSILIECMLMYPLIIINIVETQFIVLLNVVKKRLIAVKKKLNEITKKGVRNINLEILQTLATAHSTLVDICQDINSLYSHQILMIFSGIFILTTTNLYHVVEKLIVFVAKKDVKMLNFATITSYRVMVRAYEVWTVVRSCSKANEKAKEFNTQLYQLMIDDRMNDISNDKKLRLHIAMKRDVVFTACGFFTLDYTLVHSMIAAATTYLVILIQFGKPNSNSSPDASNNATLLLNTSTTSLPLMISTIGTV

>HhalGR68a

MMKPDVKRCLGILMGPAKMLGLFPIAWDERRCYRISVPFIAISSLKCLAYTVVTIVYLSVNFTASENIALGAEIDYISLILINAIPILSVCELVCTLHEFNECIFFLEAAELQLLQLGKFVDYDTSKRPLWINALLALGAFFARFAKNAIIDPAAVLLSGLQVLVTFSLISHMIVLVYWYFGVVGILTKLFAACNQEVRNYVRDFVVLKMRKVEKLARAHHTLCLCTTTLNDIHGAQLVAIFLSCFVLSVTEVYRCIIFLEEKVDVTFFLVIAVKLCCIILCFNLCLQIVTACKECSAEAKEFHTLLYQLMLDDKTNDLSNNKKLCLHIAMKREVVFTACGFFKLDYTLVHSMIAAATTYLVILIQFGQPRSMPTLPTNSLKSSEYTNATSPSTRII

>HhalGR32a

MFSLTSCLFQVDHVNRVFGFQLLVVFINIFTALVDELYHLIYLKSSGQFGDDILEVLFLLVSLAYTFFNTFAITSACDKTVVKAKEFNTLLYQVMISDKTSIVSKNKKMRLHASMKREIMFTAYGFFSLDYALVHSIAAAVTTYLVILIQFSQPTPGSD

>HhalGR63a

MAYEYLPSVYIRRKDAEESTTKYSKRRSEVSVLYQVLEPILLLMRLAGRFPYAINQKPDKEMHCGWAVYSAAVGVLQFAGVYFTSNLSYSLSLGNFDEQIFATVTTIVCFILGLNPFLTWFEAPWLSAYLDKWSAYQDELRSFDVILDTRLQKWLLLWILALFPYTAVVAYFLDVRRGDPTAFPVYLLLQIGSYLLLTLWFFMVFFIEDTASRLLARIGDASAEREVAILKKLWLTLANLTTELGHVLSLTLILFMISCSIIGIANCYSLLFFLRDCFLDNCDSSSYQQIPIISQVGTLIVSAIIIIAICEHGHRCTVSVGSNFLKEILKINFSLRNENTQRELHSLVQTILLRYPDMALGCYFTVNRRLLATMVTTAITYLIVLLQFRSTGIKT

>HhalGR43a

MQIRSGSLEVTTKIFETEIKSLPSCGDLHWDICEAIAYFNASFKTQLQLQFLALFIKLIDCPYFIFLTLSYGLNFQYIVSSLIYTLLQIAQILAIVSPCSAATKAGNDTSFILCKHLHSGIPMAMKKNIKSFLTLLEVHRTDFHCGIFTLNESVVSAIIGSVTTYLVILIQFQDEDFDS

>HhalGR2a

MDTRLQSNSLNDVFVEDIFIKIIFLLSKLIGIFPCRYRNGQFFASIPLCIWSFLIACAMSFTTVYFHINLFIPKSRIKLLVVDTLTQVIVSLWFCFCLWVMYLIAVTNSVARMKQMNRILSGLNEIDRQLIILEIRRCWRQRFMYFAKSYFAGLFVVILCITQSPLISLWTGLKGFLFLPACSVWLCAAQFTALADQVTHRLDEACIALNDIVLTRHFSISDKKLVTLSQVQDKLCDIYVILDSSNAWLITCIISVCFNGILIPFYMALTAFFLAEAAFNINVIVSALCWIFISLHTVWLIVSTTSGITMKLKGFNLTLYKLMMSDKRNEVLRNNKLRLHISMQREVVFTAKGFFKLDFTLIQWIIASATTYLVILIQFTPPGDETPTDTLKSDVNNTVLP

>HhalGR22

MFWCITLYELRTASDKLLNRIIASGCRNMYQYRILWLKLSDLISTIGESIGHTGLAVCALIFTTFVFTTYGLLSLAIDHAFTKSFCGLLCTSLLSMSLQFIMCDAAHRTTQAAGEKFSSKLLELSTSSLSQKDQFEMNFLLQTMMTNPPVIHFIGYMIIDRNLFVKFISDTVTYLVVLIQFKTSSLARSLVNASSEVFN

>HhalGR1

MINNNIMRDRIDVDLLGNITTQSILHYLSEEERKDNKKKKEKADSRSTIQLELRTPLLLAQFLSLLPIYGVSNPDYRKLRFEWKSWKVLYSFGIITFNIILCIFALNRYIRDRGVTYRSLGNPASIRDIMFFGSTLLISMSFLILVRQWKDFIGKWASLQGEMNNFESSNVQKKIKLLTNCLLFSSIFEHILSNTQFMLNVEENQDRLRMFLDDKNVSSVLGFNTWCYIFIYFGNFLITLIWNYTDIFLTAVSLSLANMFKQFNRDLRMTAAMVSHDNALSYWRRKRELYNSLSLLAKTVEEKISRLVLISFSVNLYFICIQLLNSIQPLTSFLQVAYFCGSFGHILFRTCSVCFAATSIYEHSKGSLPTLYSVSSEQFNSEVQRLIDQVTGETLALTGCKFFTVTRTLMLTIAGTIVTYEVVLVQFNNPTDDSGSDAHNSSRRA

>HhalGR2

MINNNIMRDRIDVDLLGNITTQSILHYLSEEERKDNKKKKEKADSRSTIQLELRTPLLLAQFLSLLPIYGVSNPDYRKLRFEWKSWKVLYSFGIITFNIILCIFALNRYIRDRGVTYRSLGDIMFFGSTLLISMSFLILVRQWKDFIGKWASLQGEMNNFESSNVQKKIKLLTNCLLFSSIFEHILSNTQFMLNVEENQDRLRMFLDDKNVSSVLGFNTWCYIFIYFGNFLITLIWNYTDIFLTAVSLSLANMFKQFNRDLRMTAAMVSHDNALSYWRRKRELYNSLSLLAKTVEEKISRLVLISFSVNLYFICIQLLNSIQPLTSFLQVAYFCGSFGHILFRTCSVCFAATSIYEHSKGSLPTLYSVSSEQFNSEVQRLIDQVTGETLALTGCKFFTVTRTLMLTIAGTIVTYEVVLVQFNNPTDDSGSDAHNSSRRA

>HhalGR3

MLTIYKNQIFAEGVYSRNHHKNVTNVGTKHRNIHFKIQPQLMAKDNIYFNEMKPIFIMLRMVGRFPYSFTKTGFAPFSFISWPVLYSLVFNLVFVLMTIRSMQIMINDKIYPSRSYDETLFWFLLLLFALQSFTGPITFWMDAPGLVSYFQKWKDFEDFWRLGTLYSVNRHRFTRFFSISIMPLVALFVTYETLTLPKISILIFLPHIPIVIALLLMQVYWWLTLHDLTQYSEKLLTSMLKVKDRRGMGYRRRIWQQISNLVTEIGNAIGASGLSYSITNFVGFILSTYGILINLASPGSSDVSVMGLLLPAVACAMTIFLVTDAAYKATECVGHKFTKNLLQIDLSTLSRSCLSEVDLMFNSLSANSPVIEYLGFMKITRNTFLQFVSHTATYLIVLVQMKTQPKHSKNG

>HhalGR4

FNVLWPNKTLNMMKPDVKRCLGILMGPAKMLGLFPIAWDERRCYRISVPFIAISSLKCLAYTVVTIVYLSVNFTASENIALGAEIDYISLILINAIPILSVCELVCTLHEFNECIFFLEAAELQLLQLGKFVDYDTSKRPLWINALLALGAFFARFAKNAIIDPAAVLLSGLQVLVTFSLISHMIVLVYWYFGVVGILTKLFAACNQEVRNYVRDFVVLKMRKVEKLARAHHTLCLCTTTLNDIHGAQLVAIFLSCFVLSVTEVYRCIIFLEEKVDVTFFLVIAVKLCCIILCFNLCLQIVTACKECSAEAKEFHTLLYQLMLDDKTNDLSNNKKLCLHIAMKREVVFTACGFFKLDYTLVHSMIAAATTYLVILIQFGQPRSMPTLPTDSLKSSEYTNATSPSTRII

>HhalGR5

MKQGSMSKAWITTSVSHNKPYKKVPVKKYFQEIKPLILLQRAFGKLPYSFNEEGFAPFKLLSFPVIYTIIFIVFQSTWTVYSLCIIIQEKIHKAPSYDVTLYWVSIGLFLLLNFTTPMTKWIDIRKFVHHVSSWQDFQNNHLDAELGANLSLTLMIASVLLLPIASVFIYCQSYLLTDLSLFVMVPYIFSFVETGVIIIHWGVVLYELRIASRTLLSKIIMDGCRQMSTYRRTWLELSKLVSGVGESLGHTGLVISIVLFTTFVLAMYALLSSLFEPAKTCNHVWGLLINAVLSLLCNLFLFNAAHRTTQEVGPDFSCKILASDLTHLSQVEMNEISLMVQTISANPPTVEYLGFVTVNRSLFVSLVSNAVTYLVVLIQFKASAPEKPVKEEVVQ

>HhalGR6

MYFSSECIFNIILLMTKLLGVFPYDHVRKRVSYQWFTYSLIIVLVCTVEAAFVVFDPPRLAKNSLLRNILFRCQMLAMYACIVIFNACILIHRRKISLVFLILEKLKSETRGQPSFLLVLGYFCWQSLAITVTAYNDWNGNTPLDGTVSLALCYYYGMISIIVVGTAPFVALVLLVSTKLEHLVIALKSKVTLGSGRLYSYVLLYDRMVSLLTSLNEAFSLQLLLISFVSFFNLTINMFFIADYVVNDASIHRAKMVPVYMGWITMFSSMVIFPVYSCHRTTKQAKEFNTQLYQLMIDDTTNDISNNKKLRLHIAMKREVVFTACGFFTLDYTLVHSMIAAATTYLVILIQFGQHQSTAPELPANPPDFSNTTALPLSTSTI

>HhalGR7

TYTSLQDSTMVKGVHNALSLVLALSRAFGAFVLRAQGRTYVFSYKLISYFIVLAMGTIAEGIIEASNLQSECSFSLLLIVDIFVFCFYLITAIIVLYQHYRLREPLPAIISELEDMEDHIGDVSYGRYYSYGVIFLSILNALPRIFAVLRRSFAWNYLLTRILYYSFTQIPLLIAAQYAMFLHILSRQLYTLSEQLNTAIFNLEVWRLIDIHHNLVLLADRINRAFDLFLIHMVTFIFVINILRLYFLIVYIVNPVSYTDLELTIISFVDILINCGSLIIIVSAAMEATKKAELFNKQLLKSLLISKTIAQDEKIRTYLGMKHSIQQSACNFFSLDYHLLTSMAAGTTTYVILLVQFTLL

>HhalGR8

KGVHNALSVVLALSKAFGAFTMSFQGQIYVFSYKLYAYFIVLVIGVLVQGIIDAFIASSEHPLSLVMFIDYFVFSIYIFTAIIISFQHYYLRESLPAIISELGDMDQLIGNVSYSGYYKFGVIVLFVLNALPRIFAVAERPFTWNHLLKRTLYFFITVVPVLVGGQYGSVLQILSRQLHSLSEQLNNFQSNLEVWTLIDVHHNLVLLADRINKAFNMYLLNTITFCFVIDILKLYFVIVYIVKPVTYTDLELTVISSMDILVNWGSIFTIVFAAMEAKKKAELFNKQLLKSLLISKTIAQDEKIRTYLGMKHSIQQSACNFFSLDYHLLTSMAAGTTTYVILLVQFTLL

>HhalGR9

QNALSFVLAMSRPFGASVLRVQGWTYVFCYKLYSYLILLVTTTIAEGIIETSFLISLYSLSFLFIIESSVFWINTTTAVIVIYQHYQLREELPAIISDLECMDSLIGGVTYSGYYNYGAIILSIVTATPRIIAILLRPLSWNNVFMRVLYLFITEIPILIATQYAILLHILSRQLHTLSEQLDAVMFNIKVLSLIDVHQNLVLLAGRINTAFDTFLMYMITSIFVINISTLYFLIIYIMKPVSAIDLPLSIVCALSFLVNCGLLMLMVFPAMEATKNAELFNKQLLKSLLISKTIAQDEKIRTYLGMKHSIQQSACNFFSLDYHLLTSMAAGTTTYVILLVQFTLL

>HhalGR10

GNFIRRMKQGSMSKAWITTSVSHNKPYKKVPVKKYFQEIKPLILLQRAFGKLPYSFNEEGFAPFKLLSFPVIYTIIFIVFQSTWTVYSLCIIIQEKIHKAPSYDVTLYWVSIGLFLLLNFTTPMTKWIDIRKFVHHVSSWQDFQDGCRQMSTYRRTWLELSKLVSGVGESLGHTGLVISIVLFTTFVLAMYALLSSLFEPAKTCNHVWGLLINAVLSLLCNLFLFNAAHRTTQEVGPDFSCKILASDLTHLSQVEMNEISLMVQTISANPPTVEYLGFVTVNRSLFVSLVSNAVTYLVVLIQFKASAPEKPVKEEVVQ

>HhalGR11

GNLLRRMRNRTLSEAWVTTSVFSIKPYTKVSVNKYFLEIKPLIILQRALGKLPYSFNKHGFDPFKIISFPVLYTIVFFTLQSAWTIHTMSVIIKEKIFNAPSYDMALFWVSLELILLLNIASPITKWIDVHKYVQFVNNWKDFQNGCRQMSTHRKTWLKLSKLVSEVGDSNAHTGIIMSVTYFTSLVVTTYALLSSFSRLADYNSHFWGHLVSTLIGFLSNFVLCDAAHRTTQELGPEFSSKILAMDMTHLSQSEVNEICLLLQTMSAHPPLIGYLGFVTINRNLFVSFMSNAVTYLVVLVQFKSTSPLNPIKEDITQ

>HhalGR12

PFSFISWPVLYSLVFNLVFVLMTIRSMQIMINDKIYPSRSYDETLFWFLLLLFALQSFTGPITFWMDAPGLVSYFQKWKDFEVKDRRGMGYRRRIWQQISNLVTEIGNAIGASGLSYSITNFVGFILSTYGILINLASPGSSDVSVMGLLLPAVACAMTIFLVTDAAYKATECVGHKFTKNLLQIDLSTLSRSCLSEVDLMFNSLSANSPVIEYLGFMKITRNTFLQFVSHTATYLIVLVQMKTQPKHSKNGPFPDSYGNSTDFENT

>HhalGR13

EVAILKKLWLTLANLTTELGHVLSLTLILFMISCSIIGIANCYSLLFFLRDCFLGNCDSSSYQQIPIISQVGTLIVSAIIIIAICEHGHRCTVSVGSNFLKEILKINFSLRNENTQRELHSLVQTILLRYPDMALGCYFTVNRRLLATMVTTAITYLIVLLQF

>HhalGR14

INRAFDTFLIHVITFNFVIGTMKLYFVVVYIVKPIAYTDLELTVISAVDFLVNFGSIVIIVTAAMKANKKAELFNKQLLKSLLISKTIAQDEKIRTYLGMKHSIQQSACNFFSLDYHLLTSMAAGTTTYVILLVQFTLL

>HhalGR15

VELWSVVDTCEAVIQKAKDFSAALYQFMIDESTESCKNKKIRLHLTVQKEPCFTAYGFFDLDFTLLHSIIAAATTYLVILIQFSQTTTSYPKRVLLNTTASYNSSYSNYTE

>ClivGR3

MKFLESIPTTATKTKNAGNDTKGFNLFVISKILGVFPFDQTYGYDTSWFSFSCALHLLSCASWIGVIILLILTDYPGSPGKYLNKALHALSQVLNFSCIISHLVIVKQNPLFVGKLAAFTAVYEHQVFGKAISVGALLVAGRSVPIAVFRLLNANYKKMTEEILHWFSSMVRLMVIFQFCTFLVILRRKLESHIKEMNQYKINTSAHFNMVEFMKKLNRIFGCQLLSTFCQIFIGTVANSYWFINSIVTPSEWTLRQILFSLSNLTAVIYVVFEMYVIVRTCSDALYQVELFNVELFRLMRGSKQLCENEKLYLYATMKNSVTFTACGFFNLGYPLVTSIIAAAATYLVILLQFSTASHKQF

>ClivGR2

MEIGPLFEESKDPGAVFTVSRFLGLICVNKTGELTTPLVVVSFLILSVSSAASVAAMLYSLLEHIDTNDQTDNFWSATEIIRCSLDIVAVFTHFTSILVNRTKFRRSYVRVSRLRVYKSKSVELKIVVGMFAFRTCCSLFQIHIAEQRDQIVDVISGHLTAFMYFNTTLIVLQMCTMTEAIRSKFDEFLKISPQHYLLTDRHSKTVDFLVEVLSIYEGTLLIFALRSLIEMTSMGFYSYVTFYKVVNDHSMWYSAYSAVNLISLVVVLTTMFIVVRTCEMASEQIECFHKELFRQMRGNPSLCTNKKLSLYVSMRKTITFTACGFFTLGYPLITSIVAAATTYLVILVQFSVPKT

>ClivGR1

MQVFIIGPTDKRGFLMSMYGVNKVSVNCFVKVFFPAILMGLMPYCPNRRISRFLLVYSLAFHILHRCFYIYVLTRYIGQYSDMDFVLMSLICTNCIFKTSCIVSHLFSIIRGREKFLSIVDVCLNEDFTRNRFRFITLAGVFVLPIPLIARQVVDTGWVSMMYCIGGIVDIVSIFLIAMQYCTLLEIATKRFIATKFSLHMQLNEIENATDMHNNILELTKKIHSIYGRTILVEVCYLGFDFIVAFYDAIQSVRVHQLYTWGVSTFFCSIGYILTIYILCNACETVMRMAEKFNAELFRLMRENKELCKNEKLQLYVTMKQTVNFTACGFFTLGYPLVTSIIAAATTYLVILVQFSMPAAPAKT

>AlinGR3

MRPVLLVSQFIGVFPICGVLGSDHTQLKYSKFSFKSLVSMTVMTATSVLAATTTLLVIREGLTYKAAGDIVYTLANASSMIAFYNLAKRWKSLMGKWAAVEDSMAGLPTVNVSRSIQILTAYVVCASMCEHTMAILYNRSPCIISLETYYKECFRHVFSFTKYALWKGILASLLGFYMVSAWNFTDLFLMTLSIALSSRFKQFNLELESIMHLDMDAKYWMKMRGIYNKLALLTKLVDANVSTLIWISFINNLFFVCLQLLHTTDPFQSIPKMIYSIISFSHLVFRACAVCMTAADVYHSSKAPMAILFSVPSSSYNIEVQRMTLQVSMENLSLSGCRFFFITRTLMLTVAGTIATYEIVLLQFRNISPDFSETTAINCTGLYSDDIYE

>ClecGR28b

MFPTLWTGVIYNVSNRLDTNDAMWLDFLLFGIRMFGVLFAETISIIWFIIYKKHFTSFLIELQKLEFIFKKLGFPCNMTMSYRKHVVSFLLITVAFIANFLIRRITVFRTVQQIFSYVVPFILSIMVLMFNEFVEILTYQFKTMCTIVSSSKECVYPHRKIEILLQCQDVLESAATQLNKAFSPLLLLLIVITFARATLGIYNFLIISGSKVMDITGTSFMIIVYIAMTINMINIITKLLRESEAFDKAIYSLIVFDDTNTHATNPKLSQYLTGRKKIRFTAFDLFEINFSVLGQMVATGFTYLIIVVQLDAGK

>ClecGR22

MASKRKHTDKPNDPFYLEIRPLLVVLRAFARLAFGIKDGRLVSFPRSCVSLIWFVLYGAHFYLAVDVFLIAFRRLKTETSFFLTIIMMISLILTSVHFYLPLSVVLESGKICSYVNSWADFQDLFFLVTGTRFRPKYRKLLNVCLFLTPLHQIGVLWLQQKIQYFEQWYHMSLFFSILLIANMNLLFWIISFLEMAHAANLIKEIIEKFQYNFQSKSVAKLTVLWISLVKLIGRLSDSMYITMLVFITVVHSCGVTSAYAVISSINSGSVEDVFIFLMLLLLSCVLILAVIEPVHLTKIKVHDEIYREIIKIDTKKIDPTISKEFERFGEVVQKMNLKVTLGGFITIHRNLMTSMIGTAVTHLVVLVQFSTRQENSSS

>ClecGR24-X3

MYDHGFSPSNPKQAWMDKLEKTVLKRPRKVTESAFYLEMRPLLFLLRLFGKISYMINKEGKMEARLFSISSLSCLAVFAGQTFLVARNIVTLVEVLKEEENFGRFVQGFLILTFMAFHFFLPFSLYLESGKICHFFNEWAVFQDLMEKTTGHKFSTNYDKWLRACLFMCPLGVIIIVLYERNILYNSVWYQLIFYAILLMIFQISLYLWIFSLIEIGYAAQVVQKELKKTTVESCTGLNIYNYRLIWLKLSKLLEIVGDAVALTMIAMTTVNHTCFIISAYMLISSFMHSLYDSIPFLTIMVITGLMITQTFEPGEFVSRKLGKQIADTLMETDISKVDSDCLKELNLFTQAVSGSNNVVTFGGFANVNRSALAGIVGSTVTYLIVLVQFNQSPES

>ClecGR24-X1

MLFLKMDYAVTYTGTKPGSNETVCEGYLGSSGSTVNIMSVSGLMYDHGFSPSNPKQAWMDKLEKTVLKRPRKVTESAFYLEMRPLLFLLRLFGKISYMINKEGKMEARLFSISSLSCLAVFAGQTFLVARNIVTLVEVLKEEENFGRFVQGFLILTFMAFHFFLPFSLYLESGKICHFFNEWAVFQDLMEKTTGHKFSTNYDKWLRACLFMCPLGVIIIVLYERNILYNSVWYQLIFYAILLMIFQISLYLWIFSLIEIGYAAQVVQKELKKTTVESCTGLNIYNYRLIWLKLSKLLEIVGDAVALTMIAMTTVNHTCFIISAYMLISSFMHSLYDSIPFLTIMVITGLMITQTFEPGEFVSRKLGKQIADTLMETDISKVDSDCLKELNLFTQAVSGSNNVVTFGGFANVNRSALAGIVGSTVTYLIVLVQFNQSPES

>ClecGR32a

MQFCAFLEYLRSLFIKLHLQVKYKKKITNLITQHSRVISMAMVANKLYNVQLLVIFIGLFVNLVSWLYLLVDEVQNTSRSPVAPGLLRLADSMWQICVIYFISSSCQLTKHEAEKFNQALFRLMTESSELCKNGKLQLHLTMNQTINFTACGFFTLGYPLVTSIIAAATTYLVILVQFSMPSN

>ClecGR21a

MTFLQHLYFQKSNNAFYLEVRPILLLFRIFARMTYDIKDGTLICRPRLIASLAWFILYGAQFYVTIDLLMGLTWHLKQEKNFITSIVMTVTLVFILMHLFLPISLILESDHICSYVNRWADLQVQFYKATGKKFIPKYRKLLYVLLVITPFHEMLAIWISQSVLYYDRWYHLVLYFSTFFVCQLNTLFWAISFFEMAHIASEIKEGLRTNFRSFGGYSNIANLRSLWVSLVDLCADLGRALWRTMIILMIVNFGSSVASVYAIISHFISGSPGIWTFVMVLFCNGTAILLMVEPVHMAMLKAGHGVYQELLEFNVTKLGGTSFVQVDKFLQVVKGINPRVTLGGFFTIDRSLLTTIAGASVTYLIVLIQFRIPSENM

>AchiGR1

MEILAAIKHSDHNVQGEIKKIKVEMNERLTSIEKHLEGQNKYIEEILAENQALKARVGNLEVRLNKSEQGLLSKCLEIRGIPIRAGETPSGLVASIGAGLGLKLNIEDLDTVQRRRAKNDDPRPPPIIARFTRQSVRDDLIQK

>AchiGR2

MIQLLKAKDFSATLYQLMIDENTLFCQNKKIRLHLTVQKEPCFTAYGFFDLDFTLLHSIIAAATTYLVILIQFSQTTTSQKGILLNATAAYNLSNSNYTE

>DmelGR5a

MRQLKGRNRCNRAVRHLKVQGKMWLKNLKSGLEQIRESQVRGTRKNFLHDGSFHEAVAPVLAVAQCFCLMPVCGISAPTYRGLSFNRRSWRFWYSSLYLCSTSVDLAFSIRRVAHSVLDVRSVEPIVFHVSILIASWQFLNLAQLWPGLMRHWAAVERRLPGYTCCLQRARPARRLKLVAFVLLVVSLMEHLLSIISVVYYDFCPRRSDPVESYLLGASAQLFEVFPYSNWLAWLGKIQNVLLTFGWSYMDIFLMMLGMGLSEMLARLNRSLEQQVRQPMPEAYWTWSRTLYRSIVELIREVDDAVSGIMLISFGSNLYFICLQLLKSINTMPSSAHAVYFYFSLLFLLSRSTAVLLFVSAINDQAREPLRLLRLVPLKGYHPEVFRFAAELASDQVALTGLKFFNVTRKLFLAMAGTVATYELVLIQFHEDKKTWDCSPFNLD

>DmelGR8a

MSGHLGRVLQFHLRLYQVLGFHGLPLPGDGNPARTRRRLMAWSLFLLISLSALVLACLFSGEEFLYRGDMFGCANDALKYVFAELGVLAIYLETLSSQRHLANFWWLHFKLGGQKTGLVSLRSEFQQFCRYLIFLYAMMAAEVAIHLGLWQFQALTQHMLLFWSTYEPLVWLTYLRNLQFVLHLELLREQLTGLEREMGLLAEYSRFASETGRSFPGFESFLRRRLVQKQRIYSHVYDMLKCFQGAFNFSILAVLLTINIRIAVDCYFMYYSIYNNVINNDYYLIVPALLEIPAFIYASQSCMVVVPRIAHQLHNIVTDSGCCSCPDLSLQIQNFSLQLLHQPIRIDCLGLTILDCSLLTRMACSVGTYMIYSIQFIPKFSNTYM

>DmelGR9a

MSLWLEHFLTGYFQLCGLVCGWSGSRLGRLLSSTFLVLILIELVGEIETYFTEENPDNESVPAYFAKVIMGVNMAYKMIHAWIALSALFECRRFRYLLEELPPVKATSFIYRHLILEIILFACNAFLVLSEYTIRGIYLENLRYAYSLQAVRARYLQMMVLVDRLDGKLEQLHHRVISGSSDYKTLRLDYAHLAKVTRSLSHLFGLSLLLLNVLCLGDWIIVCNVYFMVAYLQVLPATLFLFGQVMFVVCPTLIKIWSICAASHRCVSKSKHLQQQLKDLPGQTPVERSQIEGFALQIMQDPIQIDVCGIYHLNLQTLAGMFFFILEALVIFLQFVSLVRT

>DmelGR10a

MTSPDERKSFWERHEFKFYRYGHVYALIYGQVVIDYVPQRALKRGVKVLLIAYGHLFSMLLIVVLPGYFCYHFRTLTDTLDRRLQLLFYVSFTNTAIKYATVIVTYVANTVHFEAINQRCTMQRTHLEFEFKNAPQEPKRPFEFFMYFKFCLINLMMMIQVCGIFAQYGEVGKGSVSQVRVHFAIYAFVLWNYTENMADYCYFINGSVLKYYRQFNLQLGSLRDEMDGLRPGGMLLHHCCELSDRLEELRRRCREIHDLQRESFRMHQFQLIGLMLSTLINNLTNFYTLFHMLAKQSLEEVSYPVVVGSVYATGFYIDTYIVALINEHIKLELEAVALTMRRFAEPREMDERLTREIEHLSLELLNYQPPMLCGLLHLDRRLVYLIAVTAFSYFITLVQFDLYLRKKS

>DmelGR10b

MRVGKLCRLALRFWMGLILVLGFSSHYYNPTRRRLVYSRILQTYDWLLMVINLGAFYLYYRYAMTYFLEGMFRRQGFVNQVSTCNVFQQLLMAVTGTWLHFLFERHVCQTYNELSRILKHDLKLKEHSRFYCLAFLAKVYNFFHNFNFALSAIMHWGLRPFNVWDLLANLYFVYNSLARDAILVAYVLLLLNLSEALRLNGQQEHDTYSDLMKQLRRRERLLRIGRRVHRMFAWLVAIALIYLVFFNTATIYLGYTMFIQKHDALGLRGRGLKMLLTVVSFLVILWDVVLLQVICEKLLAEENKICDCPEDVASSRTTYRQWEMSALRRAITRSSPENNVLGMFRMDMRCAFALISCSLSYGIIIIQIGYIPG

>DmelGR22d

MFRPRCGLRQKFVYVILKSILYSSWLLGIFPFKYEPKKRRLRRSMWLILFGVVISSSLLILMVKQSAEDREHGIMLDVFQRNALLYQISSLMGVVGVVSICTVHLRTLWRSKHLEEIYNGLMLLEAKYFCSNAVECPAFDGYVIQKGVVIVVGLLAPWMVHFGMPDSKLPVLNVLVVSMVKLGTLLLALHYHLGVVIIYRFVWLINRELLSLVCSLRGNHKGSSSRVRFLLKLYNKLVNLYSKLADCYDCQTVLMMAIFLAANIIVCFYMIVYRISLSKMSFFVMLIMFPLAIANNFMDFWLSMKVCDLLQKTGRQTSMILKLFNDIENMDKDLEISISDFALYCSHRRFKFLHCGLFHVNREMGFKMFVASVLYLLYLVQFDYMNL

>DmelGR21a

MSFWAVSRGLTPPSKVVPMLNPNQRQFLEDEVRYREKLKLMARGDAMEEVYVRKQETVDDPLELDKHDSFYQTTKSLLVLFQIMGVMPIHRNPPEKNLPRTGYSWGSKQVMWAIFIYSCQTTIVVLVLRERVKKFVTSPDKRFDEAIYNVIFISLLFTNFLLPVASWRHGPQVAIFKNMWTNYQYKFFKTTGSPIVFPNLYPLTWSLCVFSWLLSIAINLSQYFLQPDFRLWYTFAYYPIIAMLNCFCSLWYINCNAFGTASRALSDALQTTIRGEKPAQKLTEYRHLWVDLSHMMQQLGRAYSNMYGMYCLVIFFTTIIATYGSISEIIDHGATYKEVGLFVIVFYCMGLLYIICNEAHYASRKVGLDFQTKLLNINLTAVDAATQKEVEMLLVAINKNPPIMNLDGYANINRELITTNISFMATYLVVLLQFKITEQRRIGQQQA

>DmelGR22b

MFGSSREIRPYLARQMLKTTLYGSWLLGIFPFTLDSGKRIRQLRRSRCLTLYGLVLNYFLIFTLIRLAFEYRKHKLEAFKRNPVLEMINVVIGIINVLSALIVHFMNFWGSRKVGEICNELLILEYQDFEGLNGRNCPNFNCFVIQKCLTILGQLLSFFTLNFALPGLEFHICLVLLSCLMEFSLNLNIMHYHVGVLLIYRYVWLINEQLKDLVSQLKLNPETDFSRIHQFLSLYKRLLELNRKLVIAYEYQMTLFIIAQLSGNIVVIYFLIVYGLSMRTYSIFLVAFPNSLLINIWDFWLCIAACDLTEKAGDETAIILKIFSDLEHRDDKLEMSVNEFAWLCSHRKFRFQLCGLFSMNCRMGFKMIITTFLYLVYLVQFDYMNL

>DmelGR22f

MKMFQPRRGFSCHLAWFMLQTTLYASWLLGLFPFTFDSRRKQLKRSRWLLLYGFVLHSLAMCLAMSSHLASKQRRKYNAFERNPLLEKIYMQFQVTTFFTISVLLLMNVWKSNTVRKIANELLTLEGQVKDLLTLKNCPNFNCFVIKKHVAAIGQFVISIYFCLCQENSYPKILKILCCLPSVGLQLIIMHFHTEIILVYRYVWLVNETLEDSHHLSSSRIHALASLYDRLLKLSELVVACNDLQLILMLIIYLIGNTVQIFFLIVLGVSMNKRYIYLVASPQLIINFWDFWLNIVVCDLAGKCGDQTSKVLKLFTDLEHDDEELERSLNEFAWLCTHRKFRFQLCGLFSINHNMGFQMIITSFLYLVYLLQFDFMNL

>DmelGR22e

MFRPSGSGYRQKWTGLTLKGALYGSWILGVFPFAYDSWTRTLRRSKWLIAYGFVLNAAFILLVVTNDTESETPLRMEVFHRNALAEQINGIHDIQSLSMVSIMLLRSFWKSGDIERTLNELEDLQHRYFRNYSLEECISFDRFVLYKGFSVVLELVSMLVLELGMSPNYSAQFFIGLGSLCLMLLAVLLGASHFHLAVVFVYRYVWIVNRELLKLVNKMAIGETVESERMDLLLYLYHRLLDLGQRLASIYDYQMVMVMVSFLIANVLGIYFFIIYSISLNKSLDFKILVFVQALVINMLDFWLNVEICELAERTGRQTSTILKLFNDIENIDEKLERSITDFALFCSHRRLRFHHCGLFYVNYEMGFRMAITSFLYLLFLIQFDYWNL

>DmelGR22a

MSQPKRIHRICKGLARFTIRATLYGSWVLGLFPFTFDSRKRRLNRSKWLLAYGLVLNLTLLVLSMLPSTDDHNSVKVEVFQRNPLVKQVEELVEVISLITTLVTHLRTFSRSSELVEILNELLVLDKNHFSKLMLSECHTFNRYVIEKGLVIILEIGSSLVLYFGIPNSKIVVYEAVCIYIVQLEVLMVVMHFHLAVIYIYRYLWIINGQLLDMASRLRRGDSVDPDRIQLLLWLYSRLLDLNHRLTAIYDIQVTLFMATLFSVNIIVGHVLVICWINITRFSLLVIFLLFPQALIINFWDLWQGIAFCDLAESTGKKTSMILKLFNDMENMDQETERRVTEFTLFCSHRRLKVCHLGLLDINYEMGFRMIITNILYVVFLVQFDYMNLKFKTD

>DmelGR28b

MIRCGLDIFRGCRGRFRYWLSARDCYDSISLMVAIAFALGITPFLVRRNALGENSLEQSWYGFLNAIFRWLLLAYCYSYINLRNESLIGYFMRNHVSQISTRVHDVGGIIAAVFTFILPLLLRKYFLKSVKNMVQVDTQLERLRSPVNFNTVVGQVVLVILAVVLLDTVLLTTGLVCLAKMEVYASWQLTFIFVYELLAISITICMFCLMTRTVQRRITCLHKVLKNLAHQWDTRSLKAVNQKQRSLQCLDSFSMYTIVTKDPAEIIQESMEIHHLICEAAATANKYFTYQLLTIISIAFLIIVFDAYYVLETLLGKSKRESKFKTVEFVTFFSCQMILYLIAIISIVEGSNRAIKKSEKTGGIVHSLLNKTKSAEVKEKLQQFSMQLMHLKINFTAAGLFNIDRTLYFTISGALTTYLIILLQFTSNSPNNGYGNGSSCCETFNNMTNHTL

>DmelGR28a

MAFKLWERFSQADNVFQALRPLTFISLLGLAPFRLNLNPRKEVQTSKFSFFAGIVHFLFFVLCFGISVKEGDSIIGYFFQTNITRFSDGTLRLTGILAMSTIFGFAMFKRQRLVSIIQNNIVVDEIFVRLGMKLDYRRILLSSFLISLGMLLFNVIYLCVSYSLLVSATISPSFVTFTTFALPHINISLMVFKFLCTTDLARSRFSMLNEILQDILDAHIEQLSALELSPMHSVVNHRRYSHRLRNLISTPMKRYSVTSVIRLNPEYAIKQVSNIHNLLCDICQTIEEYFTYPLLGIIAISFLFILFDDFYILEAILNPKRLDVFEADEFFAFFLMQLIWYIVIIVLIVEGSSRTILHSSYTAAIVHKILNITDDPELRDRLFRLSLQLSHRKVLFTAAGLFRLDRTLIFTITGAATCYLIILIQFRFTHHMDDTSSNSTNNLHSIHLGD

>DmelGR33a

MIQIMNWFSMVIGLIPLNRQQSETNFILDYAMMCIVPIFYVACYLLINLSHIIGLCLLDSCNSVCKLSSHLFMHLGAFLYLTITLLSLYRRKEFFQQFDARLNDIDAVIQKCQRVAEMDKVKVTAVKHSVAYHFTWLFLFCVFTFALYYDVRSLYLTFGNLAFIPFMVSSFPYLAGSIIQGEFIYHVSVISQRFEQINMLLEKINQEARHRHAPLTVFDIESEGKKERKTVTPITVMDGRTTTGFGNENKFAGEMKRQEGQQKNDDDDLDTSNDEDEDDFDYDNATIAENTGNTSEANLPDLFKLHDKILALSVITNGEFGPQCVPYMAACFVVSIFGIFLETKVNFIVGGKSRLLDYMTYLYVIWSFTTMMVAYIVLRLCCNANNHSKQSAMIVHEIMQKKPAFMLSNDLFYNKMKSFTLQFLHWEGFFQFNGVGLFALDYTFIFSTVSAATSYLIVLLQFDMTAILRNEGLMS

>DmelGR32a

MSPNTWVIEMPTQKTRSHPYPRRISPYRPPVLNRDAFSRDAPPMPARNHDHPVFEDIRTILSVLKASGLMPIYEQVSDYEVGPPTKTNEFYSFFVRGVVHALTIFNVYSLFTPISAQLFFSYRETDNVNQWIELLLCILTYTLTVFVCAHNTTSMLRIMNEILQLDEEVRRQFGANLSQNFGFLVKFLVGITACQAYIIVLKIYAVQGEITPTSYILLAFYGIQNGLTATYIVFASALLRIVYIRFHFINQLLNGYTYGQQHRRKEGGARARRQRGDVNPNVNPALMEHFPEDSLFIYRMHNKLLRIYKGINDCCNLILVSFLGYSFYTVTTNCYNLFVQITGKGMVSPNILQWCFAWLCLHVSLLALLSRSCGLTTTEANATSQILARVYAKSKEYQNIIDKFLTKSIKQEVQFTAYGFFAIDNSTLFKIFSAVTTYLVILIQFKQLEDSKVEDPVPEQT

>DmelGR36a

MFDWVGLLLKVLYYYGQIIGLINFEIDWQRGRVVAAQRGILFAIAINVLICMVLLLQISKKFNLDVYFGRANQLHQYVIIVMVSLRMASGISAILNRWRQRAQLMRLVECVLRLFLKKPHVKQMSRWAILVKFSVGVVSNFLQMAISMESLDRLGFNEFVGMASDFWMSAIINMAISQHYLVILFVRAYYHLLKTEVRQAIHESQMLSEIYPRRAAFMTKCCYLADRIDNIAKLQNQLQSIVTQLNQVFGIQGIMVYGGYYIFSVATTYITYSLAINGIEELHLSVRAAALVFSWFLFYYTSAILNLFVMLKLFDDHKEMERILEERTLFTSALDVRLEQSFESIQLQLIRNPLKIEVLDIFTITRSSSAAMIGSIITNSIFLIQYDMEYF

>DmelGR39b

MLYSFHPYLKYFALLGLVPWSESCAQSKFVQKVYSAILIILNAVHFGISIYFPQSAELFLSLMVNVIVFVARIVCVTVIILQVMVHYDDYFRFCREMKYLGLRLQCELKIHVGRLKWQSYAKILALGIGFLVTVLPSIYVALSGSLLYFWSSLLSILIIRMQFVLVLLNVELLGHHVSLLGIRLQNVLECHLMGANCTLDGNANRLCSLEFLLALKQSHMQLHYLFTHFNDLFGWSILGTYVVLFSDSTVNIYWTQQVLVEVYEYKYLYATFSVFVPSFFNILVFCRCGEFCQRQSVLIGSYLRNLSCHPSIGRETSYKDLLMEFILQVEQNVLAINAEGFMSTDNSLLMSILAAKVTYLIVLMQFSSV

>DmelGR39a

MSKVCRDLRIYLRLLHIMGMMCWHFDSDHCQLVATSGSERYAVVYAGCILVSTTAGFIFALLHPSRFHIAIYNQTGNFYEAVIFRSTCVVLFLVYVILYAWRHRYRDLVQHILRLNRRCASSCTNQQFLHNIILYGMLTILCFGNYLHGYTRAGLATLPLALCMLVYIFAFLVLCLLLMFFVSLKQVMTAGLIHYNQQLCQGDLISGLRGRQQILKLCGGELNECFGLLMLPIVALVLLMAPSGPFFLISTVLEGKFRPDECLIMLLTSSTWDTPWMIMLVLMLRTNGISEEANKTAKMLTKVPRTGTGLDRMIEKFLLKNLRQKPILTAYGFFALDKSTLFKLFTAIFTYMVILVQFKEMENSTKSINKF

>DmelGR36c

MDLESFLLGAVYYYGLFIGLSNFEFDWNTGRVFTKKWSTLYAIALDSCIFALYIYHWTGNTNIVNAIFGRANMLHEYVVAILTGLRIVTGLFTLILRWYQRCKMMDLASKVVRMYVARPQVRRMSRWGILTKFIFGSITDGLQMAMVLSAMGSVDSQFYLGLGLQYWMFVILNMAMMQQHMIMLFVRTQFQLINTELRQVIDEAKDLLLSPRHQGVFMTKCCSLADQIENIARIQSQLQTIMNQMEEVFGIQGAMTYGGYYLSSVGTCYLAYSILKHGYENLSMTLSTVILAYSWCFFYYLDGMLNLSVMLHVQDDYWEMLQILGKRTIFVGLDVRLEEAFENLNLQLIRNPLKITVVKLYDVTRSNTMAMFGNLITHSIFLIQYDIEHF

>DmelGR36b

MVDWVVLLLKAVHIYCYLIGLSNFEFDCRTGRVFKSRRCTIYAFMANIFILITIIYNFTAHGDTNLLFQSANKLHEYVIIIMSGLKIVAGLITVLNRWLQRGQMMQLVKDVIRLYMINPQLKSMIRWGILLKAFISFAIELLQVTLSVDALDRQGTAEMMGLLVKLCVSFIMNLAISQHFLVILLIRAQYRIMNAKLRMVIEESRRLSFLQLRNGAFMTRCCYLSDQLEDIGEVQSQLQSMVGQLDEVFGMQGLMAYSEYYLSIVGTSYMSYSIYKYGPHNLKLSAKTSIIVCILITLFYLDALVNCNNMLRVLDHHKDFLGLLEERTVFASSLDIRLEESFESLQLQLARNPLKINVMGMFPITRGSTAAMCASVIVNSIFLIQFDMEFF

>DmelGR47a

MAFTSSQLCSLLTKFTALNGLNTYYFDTKTNAFRVSSKLKIYCAIHHALCVLALAHMSYSTASNLRVSVTVLTIGGTMACCVKSCWEKAQGIRNLARGLVTMEQKYFAGRPSGLLLKCRYYIKITFGSITLLRIHLIQPIYMRRLLPSQFYLNVGAYWLLYNMLLAAVLGFYFLLWEMCRIQKLINDQMTLILARSGQRNRLKKMQHCLRLYSKLLLLCDQFNSQLGHVAIWVLACKSWCQITFGYEIFQMVAAPKSIDLTMSMRVFVIFTYIFDAMNLFLGTDISELFSTFRADSQRILRETSRLDRLLSMFALKLALHPKRVVLLNVFTFDRKLTLTLLAKSTLYTICCLQNDYNKLKA

>DmelGR47b

MQRDDGFVYCYGNLYSLLLYWGLVTIRVRSPDRGGAFSNRWTVCYALFTRSFMVICFMATVMTKLRDPEMSAAMFGHLSPLVKAIFTWECLSCSVTYIEYCLSLDLQKDRHLKLVARMQEFDRSVLMVFPHVQWNYRRARLKYWYGTVIVGFCFFSFSISLIFDTTRCTCGIPSTLLMAFTYTLLTSSVGLLGFVHIGIMDFIRVRLRLVQQLLHQLYQADDSSEVHERIAYLFEMSKRCSFLLAELNGVFGFAAAAGIFYDFTIMTCFVYVICQKLLEREPWDPEYVYMLLHVAIHTYKVVITSTYGYLLLREKRNCMHLLSQYSRYFSGQDVARRKTEDFQHWRMHNRQAAMVGSTTLLSVSTIYLVYNGMANYVIILVQLLFQQQQIKDHQLTSGKDVDIVGPMGPITHMD

>DmelGR43a

MEISQPSIGIFYISKVLALAPYATVRNSKGRVEIGRSWLFTVYSATLTVVMVFLTYRGLLFDANSEIPVRMKSATSKVVTALDVSVVVMAIVSGVYCGLFSLNDTLELNDRLNKIDNTLNAYNNFRRDRWRALGMAAVSLLAISILVGLDVGTWMRIAQDMNIAQSDTELNVHWYIPFYSLYFILTGLQVNIANTAYGLGRRFGRLNRMLSSSFLAENNATSAIKPQKVSTVKNVSVNRPAMPSALHASLTKLNGETLPSEAAAKNKGLLLKSLADSHESLGKCVHLLSNSFGIAVLFILVSCLLHLVATAYFLFLELLSKRDNGYLWVQMLWICFHFLRLLMVVEPCHLAARESRKTIQIVCEIERKVHEPILAEAVKKFWQQLLVVDADFSACGLCRVNRTILTSFASAIATYLVILIQFQRTNG

>DmelGR59b

MVYWMIKLYFRYSLAIGITSQQFSNRKFFSTLFSRTYALIANIVTLIMLPIVMWQVQLVFQQKKTFPKLILITNNVREAVSFLVILYTVLSRGFRDTAFKEMQPLLLTLFREEKRCGFKGIGGVRRSLRILLFVKFFTLSWLCVTDVLFLLYSTDALIWVNVLRFFFKCNTNNILEMVPMGYFLALWHIARGFDCVNRRLDQIVKSKSTRKHRELQHLWLLHACLTKTALNINKIYAPQMLASRFDNFVNGVIQAYWGAVFTFDLSTPFFWVVYGSVQYHVRCLDYYLIDNMCDVAVEYHDSAKHSWSEVRWTKEISSYVIYANSTKLQLWSCGLFQANRSMWFAMISSVLYYILVLLQFHLVMRK

>DmelGR58a

MLLKFMYIYGIGCGLMPAPLKKGQFLLGYKQRWYLIYTACLHGGLLTVLPFTFPHYMYDDSYMSSNPVLKWTFNLTNITRIMAMFSGVLLMWFRRKRILNLGENLILHCLKCKTLDNRSKKYSKLRKRVRNVLFQMLLVANLSILLGALILFRIHSVQRISKTAMIVAHITQFIYVVFMMTGICVILLVLHWQSERLQIALKDLCSFLNHEERNSLTLSENKANRSLGKLAKLFKLFAENQRLVREVFRTFDLPIALLLLKMFVTNVNLVYHGVQFGNDTIETSSYTRIVGQWVVISHYWSAVLLMNVVDDVTRRSDLKMGDLLREFSHLELVKRDFHLQLELFSDHLRCHPSTYKVCGLFIFNKQTSLAYFFYVLVQVLVLVQFDLKNKVEKRN

>DmelGR59d

MADLLKLCLRIAYAYGRLTGVINFKIDLKTGQALVTRGATLISVSTHLLIFALLLYQTMRKSVVNVMWKYANSLHEYVFLVIAGFRVVCVFLELVSRWSQRRTFVRLFNSFRRLYQRNPDIIQYCRRSIVSKFFCVTMTETLHIIVTLAMMRNRLSIALALRIWAVLSLTAIINVIITQYYVATACVRGRYALLNKDLQAIVTESQSLVPNGGGVFVTKCCYLADRLERIAKSQSDLQELVENLSTAYEGEVVCLVITYYLNMLGTSYLLFSISKYGNFGNNLLVIITLCGIVYFVFYVVDCWINAFNVFYLLDAHDKMVKLLNKRTLFQPGLDHRLEMVFENFALNLVR

NPLKLHMYGLFEFGRGTSFAVFNSLLTHSLLLIQYDVQNF

>DmelGR58b

MLHPKLGRVMNVVYYHSVVFALMSTTLRIRSCRKCLRLEKVSRTYTIYSFFVGIFLFLNLYFMVPRIMEDGYMKYNIVLQWNFFVMLFLRAIAVVSCYGTLWLKRHKIIQLYKYSLIYWKRFGHITRAIVDKKELLDLQESLARIMIRKIILLYSAFLCSTVLQYQLLSVINPQIFLAFCARLTHFLHFLCVKMGFFGVLVLLNHQFLVIHLAINALHGRKARKKWKALRSVAAMHLKTLRLARRIFDMFDIANATVFINMFMTAINILYHAVQYSNSSIKSNGWGILFGNGLIVFNFWGTMALMEMLDSVVTSCNNTGQQLRQLSDLPKVGPKMQRELDVFTMQLRQNRLVYKICGIVELDKPACLSYIGSILSNVIILMQFDLRRQRQPINDRQYLIHLMKNKTKV

>DmelGR59e

MDSSYWENLLLTINRFLGVYPSGRVGVLRWLHTLWSLFLLMYIWTGSIVKCLEFTVEIPTIEKLLYLMEFPGNMATIAILVYYAVLNRPLAHGAELQIERIITGLKGKAKRLVYKRHGQRTLHLMATTLVFHGLCVLVDVVNYDFEFWTTWSSNSVYNLPGLMMSLGVLQYAQPVHFLWLVMDQMRMCLKELKLLQRPPQGSTKLDACYESAFAVLVDAGGGSALMIEEMRYTCNLIEQVHSQFLLRFGLYLVLNLLNSLVSICVELYLIFNFFETPLWEESVLLVYRLLWLAMHGGRIWFILSVNEQILEQKCNLCQLLNELEVCSSRLQRTINRFLLQLQRSIDQPLEACGIVTLDTRSLGGFIGVLMAIVIFLIQIGLGNKSLMGVALNRSNWVYV

>DmelGR59f

MRSSATKGAKLKNSPRERLSSFNPQYAERYKELYRTLFWLLLISVLANTAPITILPGCPNRFYRLVHLSWMILWYGLFVLGSYWEFVLVTTQRVSLDRYLNAIESAIYVVHIFSIMLLTWQCRNWAPKLMTNIVTSDLNRAYTIDCNRTKRFIRLQLFLVGIFACLAIFFNIWTHKFVVYRSILSINSYVMPNIISSISFAQYYLLLQGIAWRQRRLTEGLERELTHLHSPRISEVQKIRMHHANLIDFTKAVNRTFQYSILLLFVGCFLNFNLVLFLVYQGIENPSMADFTKWVCMLLWLAMHVGKVCSILHFNQSIQNEHSTCLTLLSRVSYARKDIQDTITHFIIQMRTNVRQHVVCGVINLDLKFLTTLLVASADFFIFLLQYDVTYEALSKSVQGNVTRYK

>DmelGR58c

MNQYFLLHTYFQVSRLIGLCNLHYDSSNHRFILNHVPTVVYCVILNVVYLLVLPFALFVLTGNIYHCPDAGMFGVVYNVVALTKLLTMLFLMSSVWIQRRRLYKLGNDLMKMLHKFRFNLGNDCRNRCLCKGLLTSSRFVLLTQQLLTRDSVVNCESNSSLRQAMVPYQSAAIVYALIMILLMSYVDMTVYMVEVAGNWLLVNMTQGVREMVQDLEVLPERNGIPREMGLMQILAAWRKLWRRCRRLDALLKQFVDIFQWQVLFNLLTTYIFSIAVLFRLWIYLEFDKNFHLWKGILYAIIFLTHHVEIVMQFSIFEINRCKWLGLLEDVGNLWDINYSGRQCIKSSGTILSRKLEFSLLYMNRKLQLNPKRVRRLHIVGLFDLSNLTVHNMTRSIITNVLVLCQIAYKKYG

>DmelGR59c

MVDLVKTILLIAYWYGLAVGVSNFEVDWLTGEAIATRRTTIYAAVHNASLITLLILFNLGNNSLKSEFISARYLHEYFFMLMTAVRISAVLLSLITRWYQRSRFIRIWNQILALVRDRPQVVRGRWYRRSIILKFVFCVLSDSLHTISDVSAQRKRITADLIVKLSLLATLTTIFNMIVCQYYLAMVQVIGLYKILLQDLRCLVRQAECICSIRNRRGGVYSIQCCSLADQLDLIAERHYFLKDRLDEMSDLFQIQSLSMSLVYFFSTMGSIYFSVCSILYSSTGFGSTYWGLLLIVLSTASFYMDNWLSVNIGFHIRDQQDELFRVLADRTLFYRELDNRLEAAFENFQLQLASNRHEFYVMGLFKMERGRLIAMLSSVITHTMVLVQWEIQNDES

>DmelGR57a

MAVLYFFREPETVFDCAAFICILQFLMGCNGFGIRRSTFRISWASRIYSMSVAIAAFCCLFGSLSVLLAEEDIRERLAKADNLVLSISALELLMSTLVFGVTVISLQVFARRHLGIYQRLAALDARLMSDFGANLNYRKMLRKNIAVLGIVTTIYLMAINSAAVQVASGHRALFLLFALCYTIVTGGPHFTGYVHMTLAEMLGIRFRLLQQLLQPEFLNWRFPQLHVQELRIRQVVSMIQELHYLIQEINRVYALSLWAAMAHDLAMSTSELYILFGQSVGIGQQNEEENGSCYRMLGYLALVMIPPLYKLLIAPFYCDRTIYEARRCLRLVEKLDDWFPQKSSLRPLVESLMSWRIQAKIQFTSGLDVVLSRKVIGLFTSILVNYLLILIQFAMTQKMGEQIEQQKIALQEWIGF

>DmelGR59a

MKRIGQAYNVYAVFIGMTSYETMGGKFRQSRITRIYCLLINAIFLTLLPSAFWKSAKLLSTADWMPSYMRVTPYIMCTINYAAIAYTLISRCYRDAMLMDLQRIVLEVNREMLRTGKKMNSLLRRMFFLKTFTLTYSCLSYILAVFIYQWKAQNWSNLCNGLLVNISLTILFVNTFFYFTSLWHIARGYDFVNQQLNEIVACQSMDLERKSKELRGLWALHRNLSYTARRINKHYGPQMLAMRFDYFIFSIINACIGTIYSTTDQEPSLEKIFGSLIYWVRSFDFFLNDYICDLVSEYQMQPKFFAPESSMSNELSSYLIYESSTRLDLLVCGLYRVNKRKWLQMVGSIVVHSSMLFQFHLVMRGGL

>DmelGR61a

MSRTSDDIRKHLKVRRQKQRAILAMRWRCAQGGLEFEQLDTFYGAIRPYLCVAQFFGIMPLSNIRSRDPQDVKFKVRSIGLAVTGLFLLLGGMKTLVGANILFTEGLNAKNIVGLVFLIVGMVNWLNFVGFARSWSHIMLPWSSVDILMLFPPYKRGKRSLRSKVNVLALSVVVLAVGDHMLYYASGYCSYSMHILQCHTNHSRITFGLYLEKEFSDIMFIMPFNIFSMCYGFWLNGAFTFLWNFMDIFIVMTSIGLAQRFQQFAARVGALEGRHVPEALWYDIRRDHIRLCELASLVEASMSNIVFVSCANNVYVICNQALAIFTKLRHPINYVYFWYSLIFLLARTSLVFMTASKIHDASLLPLRSLYLVPSDGWTQEVQRFADQLTSEFVGLSGYRLFCLTRKSLFGMLATLVTYELMLLQIDAKSHKGLRCA

>DmelGR66a

MAQAEDAVQPLLQQFQQLFFISKIAGILPQDLEKFRSRNLLEKSRNGMIYMLSTLILYVVLYNILIYSFGEEDRSLKASQSTLTFVIGLFLTYIGLIMMVSDQLTALRNQGRIGELYERIRLVDERLYKEGCVMDNSTIGRRIRIMLIMTVIFELSILVSTYVKLVDYSQWMSLLWIVSAIPTFINTLDKIWFAVSLYALKERFEAINATLEELVDTHEKHKLWLRGNQEVPPPLDSSQPPQYDSNLEYLYKELGGMDIGSIGKSSVSGSGKNKVAPVAHSMNSFGEAIDAASRKPPPPPLATNMVHESELGNAAKVEEKLNNLCQVHDEICEIGKALNELWSYPILSLMAYGFLIFTAQLYFLYCATQYQSIPSLFRSAKNPFITVIVLSYTSGKCVYLIYLSWKTSQASKRTGISLHKCGVVADDNLLYEIVNHLSLKLLNHSVDFSACGFFTLDMETLYGVSGGITSYLIILIQFNLAAQQAKEAIQTFNSLNDTAGLVGAATDMDNISSTLRDFVTTTMTPAV

>DmelGR63a

MANYYRRKKGDAVFLNAKPLNSANAQAYLYGVRKYSIGLAERLDADYEAPPLDRKKSSDSTASNNPEFKPSVFYRNIDPINWFLRIIGVLPIVRHGPARAKFEMNSASFIYSVVFFVLLACYVGYVANNRIHIVRSLSGPFEEAVIAYLFLVNILPIMIIPILWYEARKIAKLFNDWDDFEVLYYQISGHSLPLKLRQKAVYIAIVLPILSVLSVVITHVTMSDLNINQVVPYCILDNLTAMLGAWWFLICEAMSITAHLLAERFQKALKHIGPAAMVADYRVLWLRLSKLTRDTGNALCYTFVFMSLYLFFIITLSIYGLMSQLSEGFGIKDIGLTITALWNIGLLFYICDEAHYASVNVRTNFQKKLLMVELNWMNSDAQTEINMFLRATEMNPSTINCGGFFDVNRTLFKGLLTTMVTYLVVLLQFQISIPTDKGDSEGANNITVVDFVMDSLDNDMSLMGASTLSTTTVGTTLPPPIMKLKGRKG

>DmelGR 64e

MARTTGDPAKRRRCMSRIKFWRRSRVGSEVVEKDTKRFKLSLIKAWLLRIRQEDYKYSGSFQEAIKPVLIIAQIFALMPVRKVSSKFAEDLTFTWFSVRSYYALVTILFFGVSSGYMVAFVTSVSFNFDSVETLVFYLSIFLISLSFFQLARKWPEIAQSWQLVEAKLPPLKLPKERRSLAQHINMITIVATTCSLVEHIMSMLSMGYYVNSCPRWPDRPIDSFLYLSFSSVFYFVDYTRFLGIVGKVVNVLSTFAWNFNDIFVMAVSVALAARFRQLNDYMMREARLPTTVDYWMQCRINFRNLCKLCEEVDDAISTITLLCFSNNLYFICGKILKSMQAKPSIWHALYFWFSLVYLLGRTLILSLYSSSINDESKRPLVIFRLVPREYWCDELKRFSEEVQMDNVALTGMKFFRLTRGVVISVAGTIVTYELILLQFNGEEKVPGCFEN

>DmelGR64f

MKILPKLERKLRRLKKRVTRTSLFRKLDLVHESARKKAFQESCETYKNQIENEYEIRNSLPKLSRSDKEAFLSDGSFHQAVGRVLLVAEFFAMMPVKGVTGKHPSDLSFSWRNIRTCFSLLFIASSLANFGLSLFKVLNNPISFNSIKPIIFRGSVLLVLIVALNLARQWPQLMMYWHTVEKDLPQYKTQLTKWKMGHTISMVMLLGMMLSFAEHILSMVSAINYASFCNRTADPIQNYFLRTNDEIFFVTSYSTTLALWGKFQNVFSTFIWNYMDLFVMIVSIGLASKFRQLNDDLRNFKGMNMAPSYWSERRIQYRNICILCDKMDDAISLITMVSFSNNLYFICVQLLRSLNTMPSVAHAVYFYFSLIFLIGRTLAVSLYSSSVHDESRLTLRYLRCVPKESWCPEVKRFTEEVISDEVALTGMKFFHLTRKLVLSVAGTIVTYELVLIQFHEDNDLWDCDQSYYS

>DmelGR68a

MKIYQDIYPISKPSQIFAILPFYSGDVDDGFRFGGLGRWYGRLVALIILIGSLTLGEDVLFASKEYRLVASAQGDTEEINRTIETLLCIISYTMVVLSSVQNASRHFRTLHDIAKIDEYLLANGFRETYSCRNLTILVTSAAGGVLAVAFYYIHYRSGIGAKRQIILLLIYFLQLLYSTLLALYLRTLMMNLAQRIGFLNQKLDTFNLQDCGHMENWRELSNLIEVLCKFRYITENINCVAGVSLLFYFGFSFYTVTNQSYLAFATLTAGSLSSKTEVADTIGLSCIWVLAETITMIVICSACDGLASEVNGTAQILARIYGKSKQFQNLIDKFLTKSIKQDLQFTAYGFFSIDNSTLFKIFSAVTTYLVILIQFKQLEDSKVEDISQA

>DmelGR64a

MKGPNLNFRKTPSKDNGVKQVESLARPETPPPKFVEDSNLEFNVLASEKLPNYTNLDLFHRAVFPFMFLAQCVAIMPLVGIRESNPRRVRFAYKSIPMFVTLIFMIATSILFLSMFTHLLKIGITAKNFVGLVFFGCVLSAYVVFIRLAKKWPAVVRIWTRTEIPFTKPPYEIPKRNLSRRVQLAALAIIGLSLGEHALYQVSAILSYTRRIQMCANITTVPSFNNYMQTNYDYVFQLLPYSPIIAVLILLINGACTFVWNYMDLFIMMISKGLSYRFEQITTRIRKLEHEEVCESVFIQIREHYVKMCELLEFVDSAMSSLILLSCVNNLYFVCYQLLNVFNKLRWPINYIYFWYSLLYLIGRTAFVFLTAADINEESKRGLGVLRRVSSRSWCVEVERLIFQMTTQTVALSGKKFYFLTRRLLFGMAGTIVTYELVLLQFDEPNRRKGLQPLCA

>DmelGR64d

MLSTKIVLNDGLQLYTMGSLSFSVICIFCFGSFIKLSRRWPHIIRETALCERIFLKPCYANQEGLNFTRFLRRWALILLVAALCEHLTYVGSAAWSNYVQIRDCNLKVGFVENYFLRERQELFSVFEYRAWMVFFIEWNTMAMTFVWNFGDIFLFLMCRGLKIRFQQLHWRIRQNLGKPMAKEFWQEIRSDFLDLDSLLKLYDKELSGLILVCCAHNMYFICVQVYHSFQVKGAFMDELYFWFCLLYVISRLMNMMLAASSIPQEIKDISNTLYEVRSSPWCDELGRLSEMLRNETFALSGMGYFYVTRRLIFAMAGALMGYELVLFRQMQGAVVQKSICSRGPGSSMSIFFS

>DmelGR64c

MQQSGQKGTRNTLQHAIGPVLVIAQFFGVLPVAGVWPSCRPERVRFRWISLSLLAALILFVFSIVDCALSSKVVFDHGLKIYTIGSLSFSVICIFCFGVFLLLSRRWPYIIRRTAECEQIFLEPEYDCSYGRGYSSRLRLWGVCMLVAALCEHSTYVGSALYNNHLAIVECKLDANFWQNYFQRERQQLFLIMHFTAWWIPFIEWTTLSMTFVWNFVDIFLILICRGMQMRFQQMHWRIRQHVRQQMPNEFWQRIRCDLLDLSDLLGIYDKELSGLIVLSCAHNMYFVCVQIYHSFQSKGNYADELYFWFCLSYVIIRVLNMMFAASSIPQEAKEISYTLYEIPTEFWCVELRRLNEIFLSDHFALSGKGYFLLTRRLIFAMAATLMVYELVLINQMAGSEVQKSFCEGGVGSSKSIFS

>DmelGR64b

MPQGETFHRAVSNVLFISQIYGLLPVSNVRALDVADIRFRWCSPRILYSLLIGILNLSEFGAVINYVIKVTINFHTSSTLSLYIVCLLEHLFFWRLAIQWPRIMRTWHGVEQLFLRVPYRFYGEYRIKRRIYIVFTIVMSSALVEHCLLLGNSFHLSNMERTQCKINVTYFESIYKWERPHLYMILPYHFWMLPILEWVNQTIAYPRSFTDCFIMCIGIGLAARFHQLYRRIAAVHRKVMPAVFWTEVREHYLALKRLVHLLDAAIAPLVLLAFGNNMSFICFQLFNSFKNIGVDFLVMLAFWYSLGFAVVRTLLTIFVASSINDYERKIVTALRDVPSRAWSIEVQRFSEQLGNDTTALSGSGFFYLTRSLVLAMGTTIITYELMISDVINQGSIRQKTQYCREY

>DmelGR77a

MPLPLGDPLALAVSPQLGYIRITAMPRWLQLPGMSALGILYSLTRVFGLMATANWSPRGIKRVRQSLYLRIHGCVMLIFVGCFSPFAFWCIFQRMAFLRQNRILLMIGFNRYVLLLVCAFMTLWIHCFKQAEIIGCLNRLLKCRRRLRRLMHTRKLKDSMDCLATKGHLLEVVVLLSSYLLSMAQPIQILKDDPEVRRNFMYACSLVFVSVCQAILQLSLGMYTMAILFLGHLVRHSNLLLAKILADAEHIFESSQKAGFWPNRQELYKGQQKWLALELWRLLHVHHQLLKLHRSICSLCAVQAVCFLGFVPLECTIHLFFTYFMKYSKFILRKYGRSFPLNYFAIAFLVGLFTNLLLVILPTYYSERRFNCTREIIKGGGLAFPSRITVKQLRHTMHFYGLYLKNVEHVFAVSACGLFKLNNAILFCIVGAILEYLMILIQFDKVLNK

>DmelGR89a

MLRFPHVCGLCLLLKYWQILALAPFRTSEPMVARCQRWMTLIAVFRWLLLTSMAPFVLWKSAAMYEATNVRHSMVFKTIALATMTGDVCISLALLGNHLWNRRELANLVNDLARLHRRRRLSWWSTLFLWLKLLLSLYDLLCSVPFLKGAGGRLPWSQLVAYGVQLYFQHVASVYGNGIFGGILLMLECYNQLEREEPTNLARLLQKEYSWLRLIQRFVKLFQLGIFLLVLGSFVNIMVNIYAFMSYYVSLHGVPLTISNNCLVLAIQLYAVILAAHLCQVRSAKLRKKCLQLEYVPEGLTQEQAMASTPFPVLTPTGNVKFRILGVFILDNSFWLFLVSYAMNFIVVILQTSFEHINHGEI

>DmelGR85a

MYSLIEAQLLGGKLVNRVMASLRRIIQRSLGYFCALNGILDFNTDIGTGNLRRYRVLFMYRLLHNFAVISLTLKFLFDFTDHFKYIESSTLITVNFFTYFTLVFFALLSSMGSCYQWQNRILAVLKELKHQRDLSRHMGYRVPRSKQNSIDYLLFALTVLLILRLSIHLATFTLSARMGFNHPCNCFLPECMIFSMNYLLFAILAEITRCWWSLQSGLKMVLLNRQLSTVAFNLWEIERLHTRFQCLIDLTSEVCSIFRYVTLAYMARNLWSGIVAGYLLVRFVIGNGLQDVELVYLVFSFITCIQPLMLSLLVNSMTSTTGSLVEVTRDILKISHKKSVNLERSIEWLSLQLTWQHTHVTIFGVFRINRSLAFRSASLILVHVLYMVQSDYISITN

>DmelGR98c

MEMEAKRSRLLTTARPYLQVLSLFGLTPPAEFFTRTLRKRRRFCWMAGYSLYLIAILLMVFYEFHANIVSLHLEIYKFHVEDFSKVMGRTQKFLIVAIATCNQLNILLNYGRLGLIYDEIANLDLGIDKSSKNFCGKSHWWSFRLRLTLSIGLWMVIIIGVIPRLTLGRAGPFFHWVNQVLTQIILIMLQLKGPEYCLFVLLVYELILRTRHVLEQLKDDLEDFDCGARIQELCVTLKQNQLLIGRIWRLVDEIGAYFRWSMTLLFLYNGLTILHVVNWAIIRSIDPNDCCQLNRLGSITFLSFNLLLTCFFSECCVKTYNSISYILHQIGCLPTAEEFQMLKMGLKEYILQMQHLKLLFTCGGLFDINIKLFGGMLVTLCGYVIIIVQFKIQDFALIGYRQNTSDTS

>DmelGR93b

MSGLLVMPRILRCLNVSRISAILLRSCFLYGTFFGVITFRIERKDSQLVAINRRGYLWICLVIRLLASCFYGYSYDAWSGQYEDMYLRAFFGFRLIGCLICSVIILVMQFWFGEELINLVNRFLQLFRRMQSLTNSPKNRFGDRAEFLLMFSKVFSLLFVFMAFRLMLSPWFLLTLVCDLYTSVGTGMITHLCFVGYLSIGVLYRDLNNYVDCQLRAQLRSLNGENNSFRNNPQPTRQAISNLDKCLYLYDEIHQVSRSFQQLFDLPLFLSLAQSLLAMSMVSYHAILRRQYSFNLWGLVIKLLIDVVLLTMSVHSAVNGSRLIRRLSFENFYVTDSQSYHQKLELFLGRLQHQELRVFPLGLFEVSNELTLFFLSAMVTYLVFLVQYGMQSQQI

>DmelGR92a

MFEFLHQMSAPKLSTSILRYIFRYAQFIGVIFFCLHTRKDDKTVFIRNWLKWLNVTHRIITFTRFFWVYIASISIKTNRVLQVLHGMRLVLSIPNVAVILCYHIFRGPEIIDLINQFLRLFRQVSDLFKTKTPGFGGRRELILILLNLISFAHEQTYLWFTIRKGFSWRFLIDWWCDFYLVSATNIFIHINSIGYLSLGVLYSELNKYVYTNLRIQLQKLNTSGSKQKIRRVQNRLEKCISLYREIYHTSIMFHKLFVPLLFLALIYKVLLIALIGFNVAVEFYLNSFIFWILLGKHVLDLFLVTVSVEGAVNQFLNIGMQFGNVGDLSKFQTTLDTLFLHLRLGHFRVSILGLFDVTQMQYLQFLSALLSGLAFIAQYRMQVGNG

>DmelGR97a

MRFLRRQTRRLRSIWQRSLPVRFRRGKLHTQLVTICLYATVFLNILYGVYLGRFSFRRKKFVFSKGLTIYSLFVATFFALFYIWNIYNEISTGQINLRDTIGIYCYMNVCVCLFNYVTQWEKTLQIIRFQNSVPLFKVLDSLDISAMIVWRAFIYGLLKIVFCPLITYITLILYHRRSISESQWTSVTTTKTMLPLIVSNQINNCFFGGLVLANLIFAAVNRKLHGIVKEANMLQSPVQMNLHKPYYRMRRFCELADLLDELARKYGFTASRSKNYLRFTDWSMVLSMLMNLLGITMGCYNQYLAIADHYINEEPFDLFLAIVLVVFLAVPFLELVMVARISNQTLTRRTGELLQRFDLQHADARFKQVVNAFWLQVVTINYKLMPLGLLELNTSLVNKVFSSAIGSLLILIQSDLTLRFSLK

>DmelGR98d

MEANRSRLLAAARPYIQIYSIFGLTPPIQFFTRTLHKRRRGIVILGYACYLISISLMVIYECYANIVALQKDIHKFHAEDSSKVMGNTQKVLVVAMFVWNQLNILLNFRRLARIYDDIADLEIDLNNASSGFVGQRHWWRFRFRLALSVGLWIVLLVGLTPRFTLVALGPYLHWTNKVLTEIILIMLQLKCTEYCVFVLLIYELILRGRHILQQISVELEGNQSRDSVQELCVALKRNQLLAGRIWGLVNEVSLYFTLSLTLLFLYNELTILQIVNWALIKSVNPNECCQYRRVGTCLLLSINIFLSCLYSEFCIQTYNSISRVLHQMYCLSAAEDYLILKMGLREYSLQMEHLKLIFTCGGLFDINLKFFGGMVVTLFGYIIILVQFKIQFFAQSNFMQNINSTELKAYTA

>DmelGR98b

MVAQKSRLLARAFPYLDIFSVFALTPPPQSFGHTPHRRLRWYLMTGYVFYATAILATVFIVSYFNIIAIDEEVLEYNVSDFTRVMGNIQKSLYSIMAIANHLNMLINYRRLGGIYKDIADLEMDMDEASQCFGGQRQRFSFRFRMALCVGVWMILMVGSMPRLTMTAMGPFVSTLLKILTEFVMIMQQLKSLEYCVFVLIIYELVLRLRRTLSQLQEEFQDCEQQDMLQALCVALKRNQLLLGRIWRLEGDVGSYFTPTMLLLFLYNGLTILHMVNWAYINKFLYDSCCQYERFLVCSTLLVNLLLPCLLSQRCINAYNCFPRILHKIRCTSADPNFAMLTRGLREYSLQMEHLKLRFTCGGLFDINLKYFGGLLVTIFGYIIILIQFKVQAIAANRYKKVVN

>DmelGR98a

MEQMSGELHAASLLYMRRLMKCLGMLPFGQNLFSKGFCYVLLFVSLGFSSYWRFSFDYEFDYDFLNDRFSSTIDLSNFVALVLGHAIIVLELLWGNCSKDVDRQLQAIHSQIKLQLGTSNSTDRVRRYCNWIYGSLIIRWLIFIVVTIYSNRALTINATYSELVFLARFSEFTLYCAVILFIYQELIVGGSNVLDELYRTRYEMWSIRRLSLQKLAKLQAIHNSLWQAIRCLECYFQLSLITLLMKFFIDTSALPYWLYLSRVEHTRVAVQHYVATVECIKLLEIVVPCYLCTRCDAMQRKFLSMFYTVTTDRRSSQLNAALRSLNLQLSQEKYKFSAGGMVDINTEMLGKFFFGMISYIVICIQFSINFRAKKMSNEQMSQNITSTSAPI

>DmelGR94a

MDFTSDYAHRRMVKFLTIILIGFMTVFGLLANRYRAGRRERFRFSKANLAFASLWAIAFSLVYGRQIYKEYQEGQINLKDATTLYSYMNITVAVINYVSQMIISDHVAKVLSKVPFFDTLKEFRLDSRSLYISIVLALVKTVAFPLTIEVAFILQQRRQHPEMSLIWTLYRLFPLIISNFLNNCYFGAMVVVKEILYALNRRLEAQLQEVNLLQRKDQLKLYTKYYRMQRFCALADELDQLAYRYRLIYVHSGKYLTPMSLSMILSLICHLLGITVGFYSLYYAIADTLIMGKPYDGLGSLINLVFLSISLAEITLLTHLCNHLLVATRRSAVILQEMNLQHADSRYRQAVHGFTLLVTVTKYQIKPLGLYELDMRLISNVFSAVASFLLILVQADLSQRFKMQ

>DmelGR93d

MKATKYSVGILRFMSFYARFLSLVCFRLRKQKDNNVWLEEIWSNRSRWKWISVTLRIVPLCIYAFTYAEWISNRMLITEKFLHSCSLVVSIPCYLSIIHLKICHGPEVTKLVNQYLHIFRLGTLDIRRRSQFGGGRELFLLILSVCCQIHEYVFILVIASRLCGFQHIIWWVSYTYVFIICNSIMCFGFIWHLSLGVLYAELNDNLRFESGFQTAFLRKQQRIRVQKSMALFKEISSVVTSLQDIFNVHLFLSALLTLLQVLVVWYKMIIDLGFSDFRIWSFSLKNLIQTLLPVLAIQEAANQFKQTRERALDIFLVGKSKHWMKSVEIFVTHLNLSEFRVNLLGLFNVSNELFLIIVSAMFCYLVFVTQCVIVYRRRYVI

>DmelGR93c

MIERLKKVSLPALSAFILFCSCHYGRILGVICFDIGQRTSDDSLVVRNRHQFKWFCLSCRLISVTAVCCFCAPYVADIEDPYERLLQCFRLSASLICGICIIVVQVCYEKELLRMIISFLRLFRRVRRLSSLKRIGFGGKREFFLLLFKFICLVYELYSEICQLWHLPDSLSLFATLCEIFLEIGSLMIIHIGFVGYLSVAALYSEVNSFARIELRRQLRSLERPVGGPVGRKQLRIVEYRVDECISVYDEIERVGRTFHRLLELPVLIILLGKIFATTILSYEVIIRPELYARKIGMWGLVVKSFADVILLTLAVHEAVSSSRMMRRLSLENFPITDHKAWHMKWEMFLSRLNFFEFRVRPLGLFEVSNEVILLFLSSMITYFTYVVQYGIQTNRL

>DmelGR93a

MFSSSSAMTGKRAESWSRLLLLWLYRCARGLLVLSSSLDRDKLQLKATKQGSRNRFLHILWRCIVVMIYAGLWPMLTSAVIGKRLESYADVLALAQSMSVSILAVISFVIQARGENQFREVLNRYLALYQRICLTTRLRHLFPTKFVVFFLLKLFFTLCGCFHEIIPLFENSHFDDISQMVGTGFGIYMWLGTLCVLDACFLGFLVSGILYEHMANNIIAMLKRMEPIESQDERYRMTKYRRMQLLCDFADELDECAAIYSELYHVTNSFRRILQWQILFYIYLNFINICLMLYQYILHFLNDDEVVFVSIVMAFVKLANLVLLMMCADYTVRQSEVPKKLPLDIVCSDMDERWDKSVETFLGQLQTQRLEIKVLGFFHLNNEFILLILSAIISYLFILIQFGITGGFEASEDIKNRFD
